# Sterically Demanding Flexible Phosphoric Acids for Constructing Efficient and Multi‐Purpose Asymmetric Organocatalysts

**DOI:** 10.1002/anie.202202189

**Published:** 2022-04-26

**Authors:** Fabian Scharinger, Ádám Márk Pálvölgyi, Melanie Weisz, Matthias Weil, Christian Stanetty, Michael Schnürch, Katharina Bica‐Schröder

**Affiliations:** ^1^ Institute of Applied Synthetic Chemistry, TU Wien Getreidemarkt 9/163 1060 Wien Austria; ^2^ Institute of Chemical Technologies and Analytics, TU Wien Getreidemarkt 9/163 1060 Wien Austria

**Keywords:** Asymmetric Catalysis, Aziridination, Cyclization, Epoxidation, Phosphoric Acids

## Abstract

Herein, we present a novel approach for various asymmetric transformations of cyclic enones. The combination of readily accessible chiral diamines and sterically demanding flexible phosphoric acids resulted in a simple and highly tunable catalyst framework. The careful optimization of the catalyst components led to the identification of a particularly powerful and multi‐purpose organocatalyst, which was successfully applied for asymmetric epoxidations, aziridinations, aza‐Michael‐initiated cyclizations, as well as for a novel Robinson‐like Michael‐initiated ring closure/aldol cyclization. High catalytic activities and excellent stereocontrol was observed for all four reaction types, indicating the excellent versatility of our catalytic system. Furthermore, a simple change in the diamine's configuration provided easy access to both product antipodes in all cases.

## Graphical abstract

The ability of counterion enhanced catalysis to promote the asymmetric functionalization of enones was demonstrated. Combining the use of sterically demanding phosphoric acids with simple chiral diamines allowed for the preparation of simple ion‐paired and highly tunable organocatalysts. The latter could be applied in four different asymmetric transformations, providing a highly enantioselective access to both product antipodes in all cases.

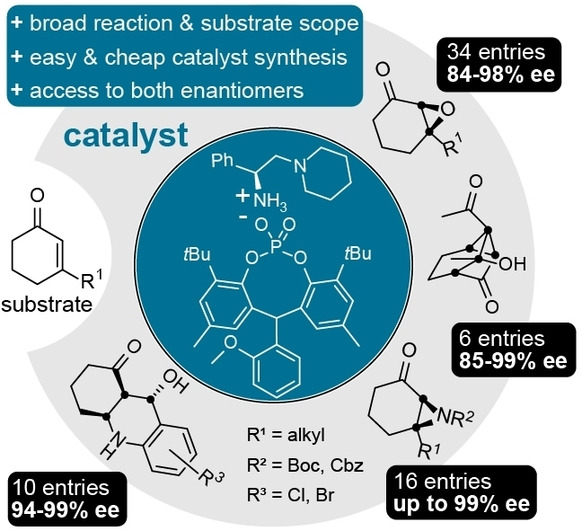

## Introduction

**Scheme 1:**
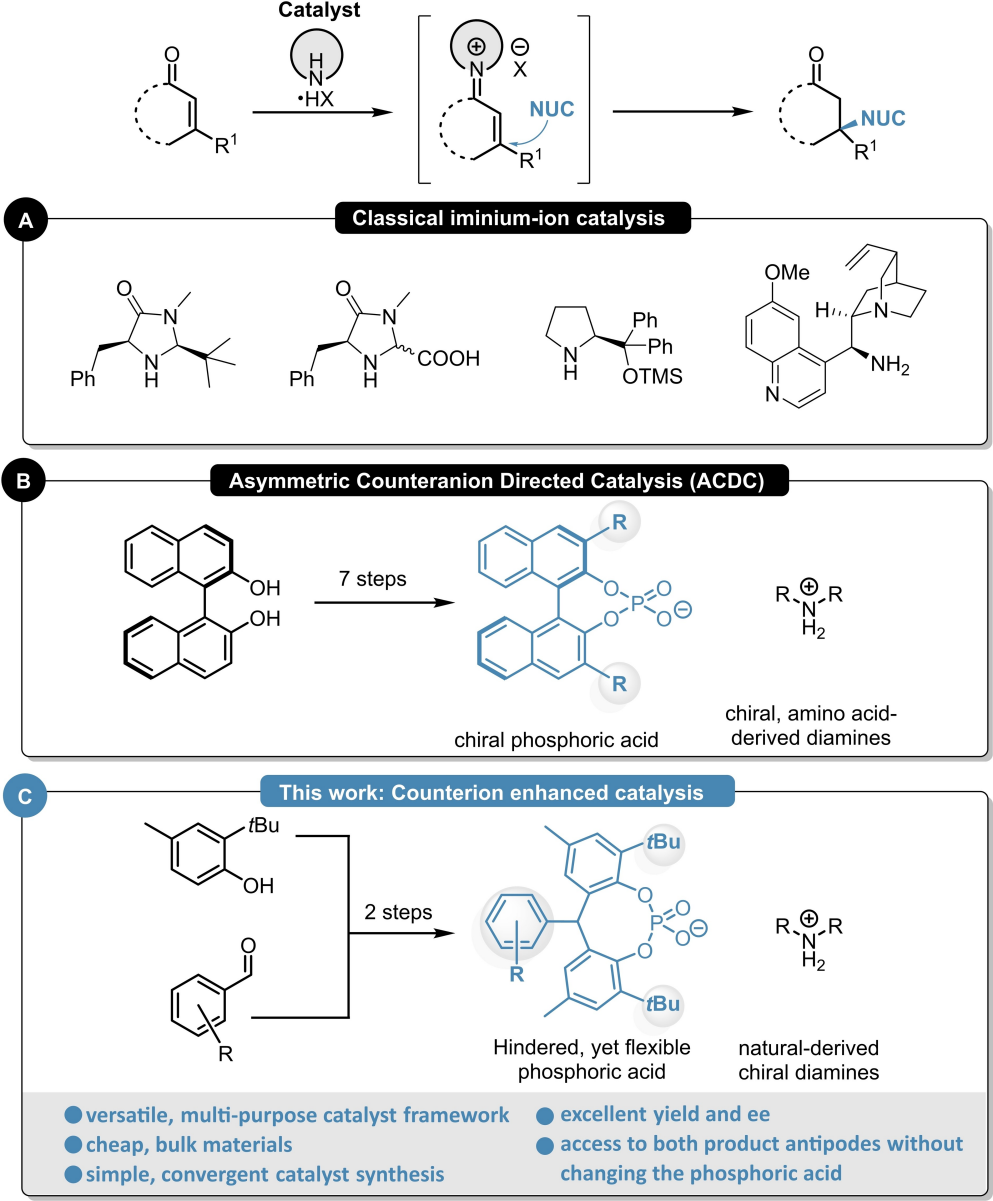
Different catalytic concepts for the asymmetric functionalization of enones: classical iminium‐ion catalysis (A), Asymmetric Counteranion‐Directed Catalysis (B) and the concept of counterion enhanced catalysis (C).

The synthesis of optically active products is one of the major challenges in modern chemistry and in this field, asymmetric organocatalysis is of special interest.^[1]^ As no toxic and/or expensive transition metals are required, such transformations generally feature great air‐ and moisture tolerance,[^2^, ^3^, ^4^, ^5^] therefore providing a simple and robust access to enantioenriched products. Iminium‐ion catalysis represents one of the most important organocatalytic activation modes and it has become a powerful and widely applied tool for the enantioselective β‐functionalization of α,β‐unsaturated aldehydes and ketones.^[6]^ Landmark discoveries from the group of MacMillan,[^7^, ^8^, ^9^] Jørgensen,[^10^, ^11^, ^12^] and List[^13^, ^14^, ^15^] resulted in powerful methods for various iminium‐ion‐catalyzed asymmetric transformations (Scheme 1A). Parallel to traditional iminium‐ion catalysis, the field of BINOL‐derived chiral phosphoric acids also emerged to be indispensable, providing elegant examples for the β‐functionalization of enals and enones using enantiomerically pure, chiral phosphoric acids (CPAs) via Asymmetric Counteranion Directed Catalysis (ACDC, Scheme 1B).[^16^, ^17^, ^18^] Despite these remarkable advances, most of the existing methods suffer either from their difficult tunability for obtaining the opposite product antipode and/or from multi‐step catalyst synthesis with tedious separations resulting in rather expensive catalyst systems.[^19^, ^20^]

Incited by these limitations and based on our previous experiences,[^21^, ^22^] we envisioned to apply our concept of counterion enhanced catalysis for various asymmetric β‐functionalization reactions of cyclic enones (Scheme 1C). By combining easily accessible chiral diamines with sterically demanding yet flexible phosphoric acids, simple and highly tunable multi‐purpose catalyst frameworks were obtained. Within this contribution we will report a set of different transformations which could be carried out with such catalyst system, resulting in excellent enantioselectivities for both product antipodes.

## Results and Discussion

### Asymmetric Epoxidation of Enones

Enantioenriched epoxides play a crucial role for the pharmaceutical and agricultural industries, both as intermediates and actual APIs. Based on the landmark discovery of K. B. Sharpless et al., the asymmetric epoxidation of unsaturated compounds is of special interest in this field. Seminal examples relying on (transition)metal catalysis,[^23^, ^24^, ^25^] phase transfer catalysis,[^26^, ^27^, ^28^] bifunctional base catalysis,[^29^, ^30^] and iminium‐ion/enamine catalysis[^31^, ^32^, ^33^] have been all successfully applied, resulting in various tools for asymmetric epoxidations. Despite these remarkable advances, the asymmetric epoxidation of α,β‐unsaturated ketones still remained a challenge. The current state of the art catalyst for this purpose was reported by the group of B. List. Their elegant strategy applied double stereoinduction by combining the chiral amine **AM1** with the chiral phosphoric acid **CPA1**, also known as (*S*)‐TRIP (Table 1, left).^[34]^ Instead of relying on such a double stereoinduction approach, we envisioned to use our concept of counterion enhanced catalysis, using simple and flexible phosphoric acids in combination with amino‐acid‐derived diamines as cheap and readily available chiral frameworks (Table 1, right). To test our concept, the asymmetric epoxidation of 2‐cyclohexen‐1‐one (**1** **a**) was chosen as benchmark reaction. Initially, we aimed to investigate if the use of a flexible phosphoric acid instead of other, commonly used acids can lead to an increased catalytic activity and/or enantioselectivity. For this purpose, the epoxidation of **1** **a** was performed by using the chiral diamine **AM2** or **AM6** in combination with the phosphoric acid **PA1**, and the results were compared to control experiments performed with TFA. When using **PA1** instead of TFA, a significant increase both in catalytic activity and enantioselectivity was observed (Table 1, entries 3 vs. 4 and 5 vs. 6), indicating a strong counterion effect. To our delight, the simple and cheap, unoptimized catalyst framework of **AM6** and **PA1** could already provide excellent results with only marginal differences to the current state‐of‐art (Table 1, entries 2 vs. 6).

**Table 1:** Proof of concept studies.

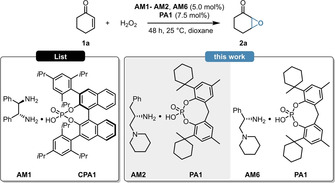						
					
Entry^[a]^	Amine	Acid	Conv. [%]^[b]^	ee [%]^[c]^		Δee
1^[d]^	**AM1**	TFA	95	90 (*R*,*R*)	**├**	**2**
2^[d]^	**AM1**	**CPA1**	98	92 (*R*,*R*)		
3	**AM2**	TFA	54	67 (*S*,*S*)	**├**	**22**
4	**AM2**	**PA1**	93	89 (*S*,*S*)		
5	**AM6**	TFA	91	79 (*S*,*S*)	**├**	**12**
6	**AM6**	**PA1**	95	91 (*S*,*S*)		

[a] Reactions were performed with 0.30 mmol 2‐cyclohexen‐1‐one (**1** **a**), 5.0 mol % amine, 7.5 mol % acid and 1.5 equiv H_2_O_2_ (50 % aq.) in 1.25 mL dioxane at 25 °C for 48 h. [b] Determined by GC analysis on a BGB5 column using *n*‐dodecane as internal standard. [c] Determined by chiral GC analysis on a BGB175 column. [d] Literature values performed at 50 °C for 24 h.

**Scheme 2:**
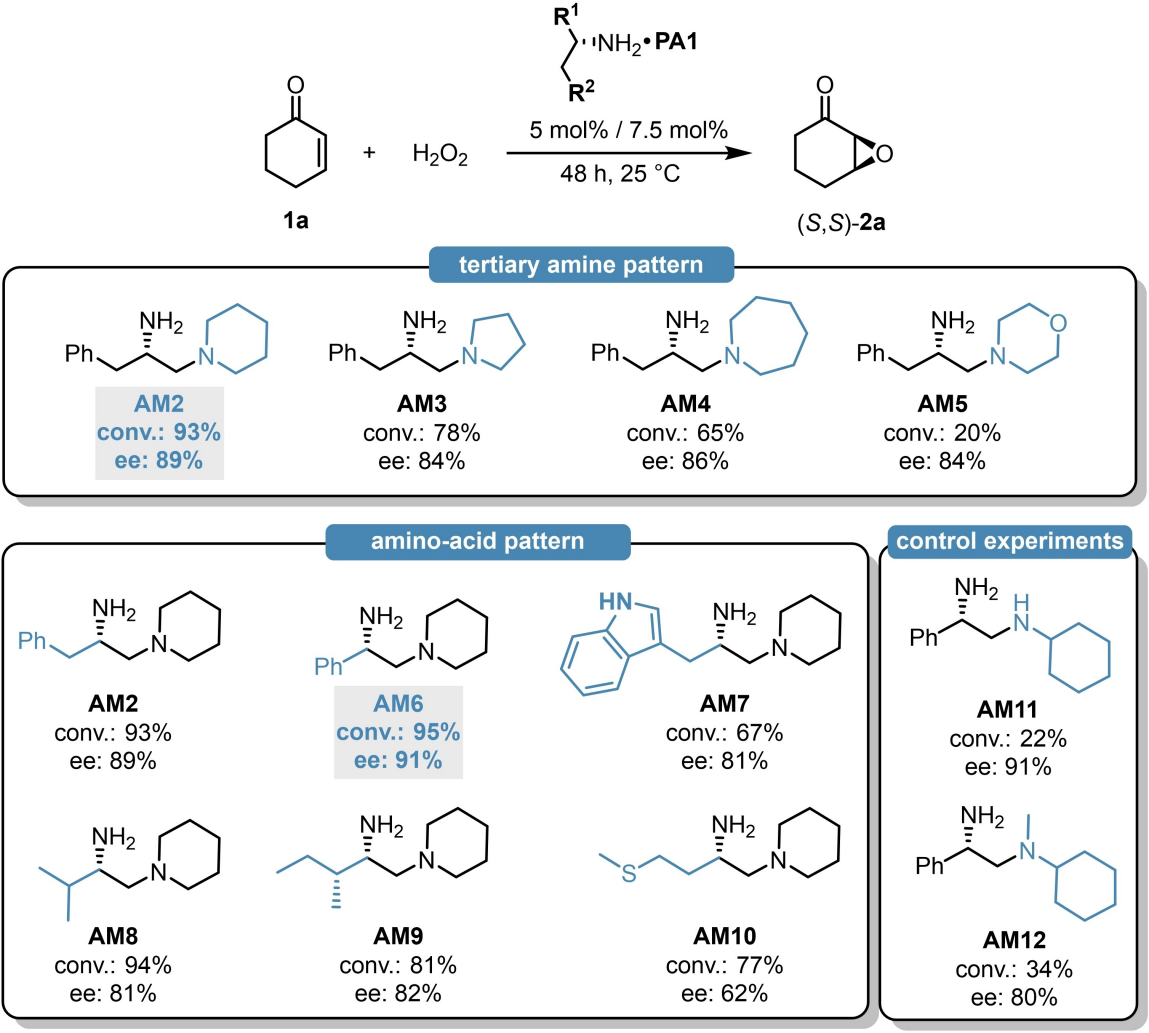
Screening of different amino‐acid‐derived diamines using **PA1** as counterion. The reaction conditions were identical to those in Table 1.

Encouraged by these initial findings, we then aimed for optimizing our catalyst framework. In order to find the ideal amine source, various diamines (**AM2**–**AM12**) were synthesized from simple and cheap amino‐acid precursors and their catalytic efficiency was evaluated in the epoxidation of **1** **a** (Scheme 2). At first, we sought to optimize the tertiary amine patterns of the diamines by using different, l‐phenylalanine‐derived amines (**AM2**–**AM5**).

Compared to the initially used piperidine unit, both the ring expansion and the ring shrinkage resulted in a significant decrease of catalytic activity and it also led to inferior enantioselectivity (Scheme 2, top). This was particularly pronounced when using the morpholine‐modified amine **AM5**, which provided only 20 % conversion. With the suitable tertiary amine modification in hand, we also aimed for screening different amino acids (**AM6**–**AM10**). This revealed, that the diamine **AM2** and **AM6**, derived from aromatic amino‐acids like l‐phenylalanine and l‐phenylgylcine result in excellent yield and stereocontrol; meanwhile, the introduction of either heteroaromatic‐ or aliphatic amino‐acids led to inferior catalytic activity and/or enantioselectivity (Scheme 2, bottom). Based on these findings, **AM6** was chosen as ideal diamine, resulting in 95 % conversion and 91 % ee. Even though l‐ and d‐phenylgylcine are non‐proteinogenic amino acids, they are eventually crucial building blocks for lactam antibiotics what makes them cheap and commercially available bulk materials.^[35]^ The cyclic tertiary amine pattern was still found to be crucial, as both the phenylglycine‐derived **AM11**–**AM12** resulted in significantly inferior catalytic activities.

We then continued our optimization by screening different solvents. While the reaction proceeded quite smoothly indeed in ether‐type reaction media, significantly worse results were obtained when using other solvents such as toluene or chloroform. We suspect that dioxane is an ideal solvent because it can fulfill two key requirements: (I) its low dielectric constant facilitates the formation of tight ion‐pairs, resulting in an effective steric shielding around the iminium‐ion and providing higher ee values; and (II) it forms a homogeneous system with the 50 % aqueous H_2_O_2_, which can improve not just the catalytic performance but it also leads to better reproducibility (for details, see Supporting Information page S24).

**Scheme 3:**
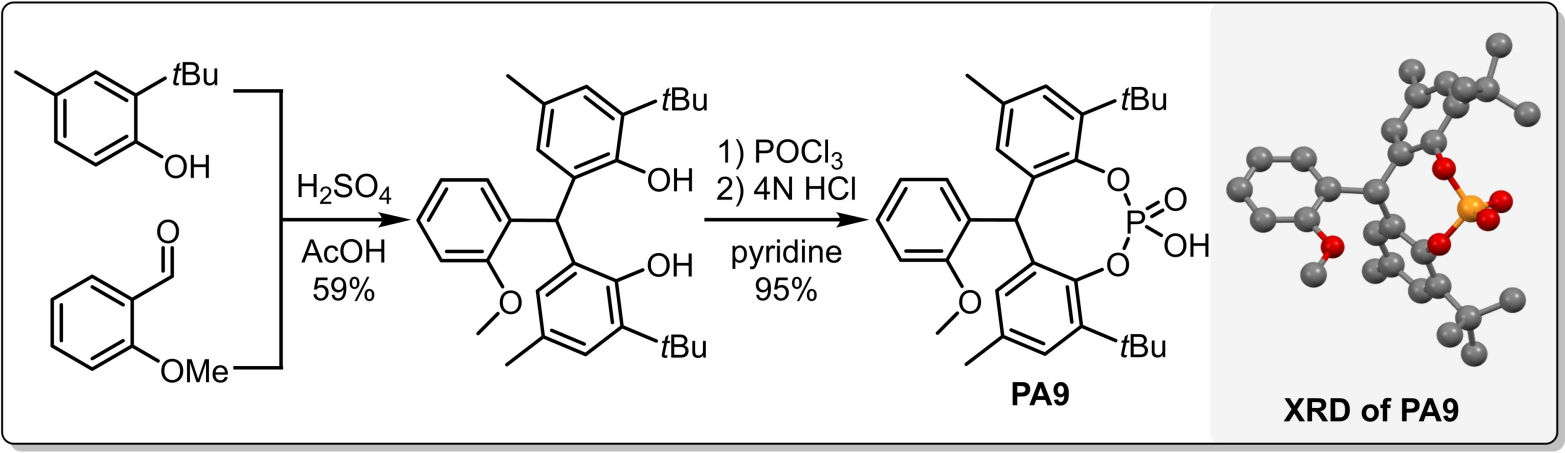
General synthesis of **PA5**–**PA9** with the example of **PA9** (left) and the XRD crystallographic data for **PA9** (right). The deposition number for the XRD of **PA9**‐MeOH adduct is CCDC 2112844.^[36]^

With the suitable diamine and solvent in hand, we continued the optimization by investigating the effect of different counterions (Table 2). For this purpose, a set of sterically demanding phosphoric acids (**PA1**–**PA9**) were prepared either via direct phosphorylation of the corresponding diols (**PA1**–**PA2, PA4**) or in a convenient two step procedure starting from simple phenols via Friedel–Crafts alkylation and subsequent phosphorylation (**PA5**–**PA9**, Scheme 3). The detailed synthesis of **PA1**–**PA9** can be found in the Supporting Information (Supporting Information page S11–S17).

**Table 2:** Acid screening.

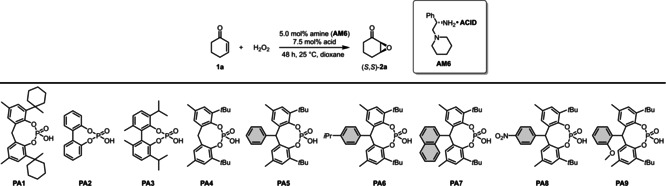								
								
Entry^[a]^	Acid	Conv. [%]^[b]^	ee [%]^[c]^		Entry^[a]^	Acid	Conv. [%]^[b]^	ee [%]^[c]^
1	no acid	0	n.d.		9	**PA4**	85	92 (96.0 : 4.0)
2	p‐toluenesulfonic acid	90	83 (91.5 : 8.5)		10	**PA5**	98	93 (96.5 : 3.5)
3	2,5‐dinitrobenzoic acid	94	67 (83.5 : 16.5)		11	**PA6**	96	94 (97.0 : 3.0)
4	TFA	95	72 (86.0 : 14.0)		12	**PA7**	95	94 (97.0 : 3.0)
5	camphorsulfonic acid	87	82 (91.0 : 9.0)		13	**PA8**	95	93 (96.5 : 3.5)
6	**PA1**	95	91 (95.5 : 4.5)		14	**PA9**	95	95 (97.5 : 2.5)
7	**PA2**	12	69 (84.5 : 15.5)		**15** ^[d]^	**PA9**	**96**	**95** (97.5 : 2.5)
8	**PA3**	97	90 (95.0 : 5.0)		16^[e]^	**PA9**	48	86 (93.0 : 7.0)

[a] Reactions were performed with 0.30 mmol 2‐cyclohexen‐1‐one (**1** **a**), 5 mol % amine **AM6**, 7.5 mol % acid and 1.5 equiv H_2_O_2_ (50 % aq.) in 1.25 mL dioxane at 25 °C for 48 h. [b] Determined by GC analysis on a BGB5 column using *n*‐dodecane as internal standard. [c] Determined by chiral GC analysis on a BGB175 column. Enantiomeric ratios in parenthesis. [d] Reaction was performed with 1.0 equiv acid. [e] Reaction was performed with 2.0 equiv acid.

While the simple biphenyl‐derived **PA2** provided poor solubility and therefore low conversion and moderate stereocontrol (Table 2, entry 7), both the yield and enantioselectivity could be improved with **PA3**, indicating that an increased steric bulk might be indeed beneficial (Table 2, entry 8). To our delight, compounds **PA5**–**PA9** all gave excellent results, providing the asymmetric epoxide (*S*,*S*)‐**2** **a** in 95–98 % yield and in 93–98 % ee (Table 2, entries 9–14). Taking into account the slightly higher yield of the phosphoric acid synthesis, but also the higher enantioselectivity, **PA9** has been chosen as optimal acid additive. Furthermore, the amine‐to‐acid ratio could be decreased to 1/1 without any loss of catalytic performance (Table 2, entries 14 vs. 15). The phosphoric acids **PA5**–**PA9** are sterically very demanding, yet feature no hindered rotation; therefore, the resulting ion‐pair might behave as a single stereoisomeric catalyst. This could be an important aspect not just to avoid the generation of matched/mismatched diastereomers between the amine and the phosphoric acid, but it also results in a simple catalyst system which provides a convenient access to both product antipodes. Indeed, after simply changing from l‐**AM6** to the diamine d‐**AM6**, (*R*,*R*)‐**2** **a** was obtained with the same excellent 95 % ee. To further test our concept, other classical Brønsted acids were also applied under the optimized reaction conditions (Table 2, entries 2–5). These provided the product (*S*,*S*)‐**2** **a** in good yields; albeit, significantly decreased enantioselectivity of 67–83 % ee were observed (Table 2, entries 2–5). This clearly indicates the superiority of our phosphoric acids, which might be a consequence of the more efficient steric shielding upon iminium‐ion formation.

In order to probe our hypothesis whether these phosphoric acids can eventually form stable enantiomers under the reaction conditions, ^31^P NMR spectra of the [**AM6**][**PA3**] and [**AM6**][**PA9**] catalyst salts were recorded at different temperatures between −30 °C and +27 °C. The formation of the catalyst salt [**AM6**][**PA3**] resulted in complete peak splitting already at room temperature, indicating the formation of diastereomers and therefore suggesting that—as a consequence of its hindered rotation—compound **PA3** is chiral and it can form distinguishable enantiomers in a chiral environment at room temperature. In contrast, no peak splitting in the NMR spectra of [**AM6**][**PA9**] could be observed, suggesting that such ion‐paired catalysts based on flexible phosphoric acids might indeed act as a single stereoisomer under the reaction conditions. The detailed NMR study can be found in the Supporting Information (Supporting Information page S153–S159).

**Scheme 4:**
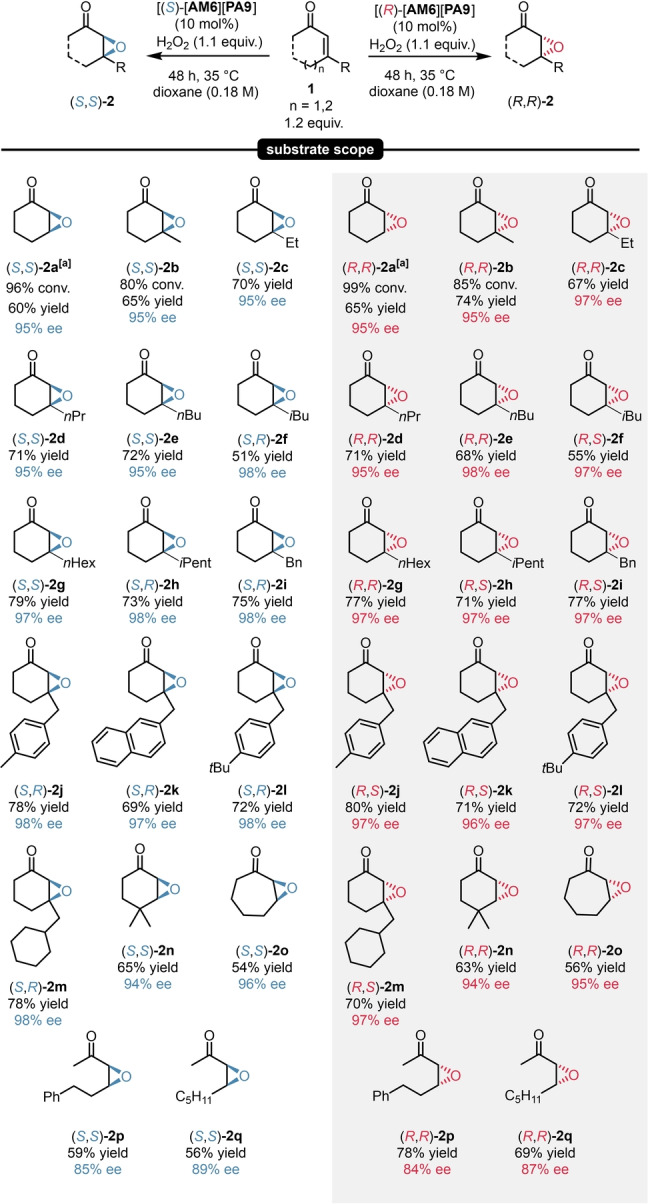
Substrate scope for asymmetric epoxidations. Reactions were performed with 1.0–3.0 mmol enone (1.0 equiv **1** **a**–**q**), 10 mol % [**AM6**][**PA9**] and 1.1 equiv H_2_O_2_ (50 % aq.) in dioxane (0.18 M) at 35 °C for 48 h. Yields refer to pure products after isolation by column chromatography. The enantiomeric excess was determined by chiral GC (BGB 175) or chiral HPLC (Chiralpak® IB or AS‐H columns) analysis. Products **2** **a**–**f** and **2** **o** were isolated with lower yields because of their high volatility. [a] Reactions performed with 5.0 mol % [**AM6**][**PA9**] at 25 °C for 48 h. For **2** **o**‐**‐q**, an additional work‐up step with 1 N NaOH was required. For details, see Supporting Information page S26.

Having all crucial reaction parameters optimized, we aimed for exploring the scope and limitations of our catalyst system. Cyclohexenones with various β‐substituents including alkyl, cycloalkyl and aryl groups were all well tolerated, resulting in a rather broad substrate scope as the corresponding epoxide products (**2** **a**–**2** **n**) were obtained in 95–98 % enantioselectivity for both product antipodes (Scheme 4). To our delight, the reaction scope could be expanded not just to other cyclic enones (**2** **o**), but eventually also to the epoxidation of acyclic substrates, still providing excellent enantioselectivities (**2** **p**–**q**). In accordance to literature—and for the sake of higher yield and enantioselectivity—a catalyst loading of 10 mol % was used for all reaction except when using the sterically unhindered substrate **1** **a**.^[34]^

### Asymmetric Aziridination of Enones

**Figure 1:**
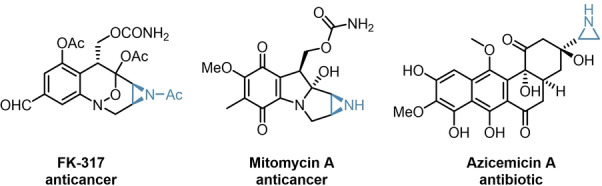
Examples of biologically active aziridines.

To investigate the robustness of our catalytic system, we envisioned to expand the reaction scope to asymmetric aziridinations. As structural analogues to oxirans, aziridines are important building blocks for the synthesis of numerous APIs as well, as depicted in Figure 1.[^37^, ^38^, ^39^, ^40^]

During the 1990s, numerous advances have been published for the asymmetric aziridination of olefins via Lewis‐acid catalysis in the presence of chiral ligands,[^41^, ^42^] as well as for the synthesis of aziridines via the addition of ylides or carbenes to imines.[^43^, ^44^] With the rise of organocatalysis, several metal‐free strategies emerged providing not just a less toxic and/or expensive alternative, but also expanding the reaction scope towards enals.[^45^, ^46^] As for the current state of the art for the asymmetric aziridination of enones, the group of Melchiorre reported an elegant strategy via simple iminium‐ion catalysis. The combination of a cinchona‐derived chiral diamine and a chiral acid resulted in moderate to high catalytic activities and high stereocontrol for various enones via double stereoinduction; however, rather high catalyst loadings of 20 mol % was required.[^47^, ^48^]

We started our optimization with the aziridination of 2‐cyclohexen‐1‐one (**1** **a**). To our surprise, dioxane, which was the most suitable solvent for asymmetric epoxidation, resulted in nearly no product formation; whereas the reaction in chloroform in the presence of NaHCO_3_ as acid scavenger proceeded smoothly at room temperature, providing the product (*S*,*S*)‐**3** **a** in high yield and in an excellent 97 % ee. Further attempts by screening different solvents and acid scavengers gave almost all inferior results. Nevertheless, changing from NaHCO_3_ to NaOAc provided even higher yields and an ee of 99.5 % when using the catalyst salt of [**AM6**][**PA9**], which was already found to be very efficient for our asymmetric epoxidations. The detailed optimization can be found in the Supporting Information (Supporting Information pages S35–S36).

**Scheme 5:**
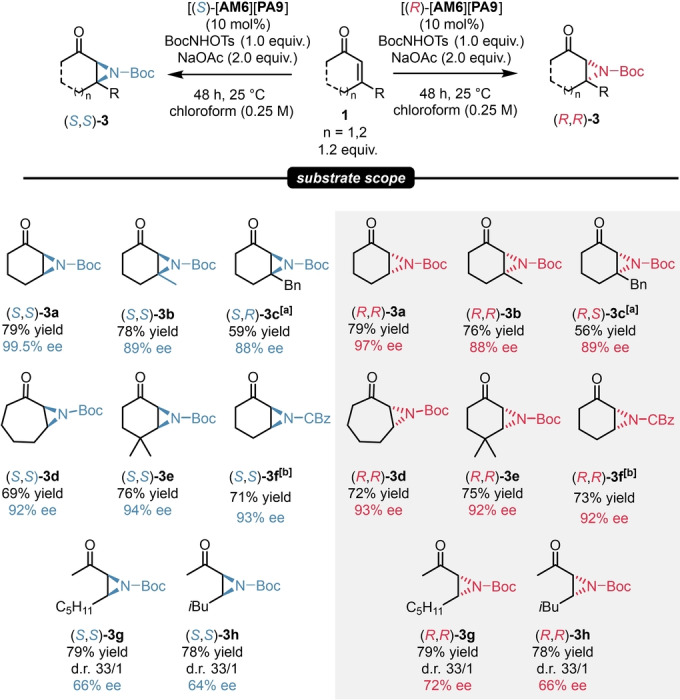
Substrate scope for asymmetric aziridinations. Reactions were performed with 1.2 mmol substrate, 10 mol % catalyst [**AM6**][**PA9**], 1.0 mmol *tert*‐butyl (tosyloxy)carbamate (1.0 equiv), 2.0 mmol (2.0 equiv) NaOAc in 4 mL chloroform (0.25 M). Yields refer to pure products after isolation by column chromatography. The enantiomeric excess values were determined by chiral HPLC analysis on a Chiralpak® AS‐H or on a Chiralpak® IA‐3 column. [a] Reaction was performed with 2.0 mmol (2.0 equiv) NaHCO_3_ instead of NaOAc. [b] Reaction was performed with 1.0 mmol (1.0 equiv) CBzNHOTs instead of BocNHOTs.

Gratifyingly, under these optimized conditions, the catalyst salt [**AM6**][**PA9**] was applicable for a set of enones. The reaction showed good tolerance for 3‐alkyl‐ and 3‐arylcycloalkenes; moreover, CBz‐protecting aziridines could be also prepared. The aziridines **3** **a**–**f** were obtained in good to high yields and in excellent enantioselectivity values of 88–99.5 % ee by using only 10 mol % catalyst loading. Furthermore, our catalytic system was found to be suitable for the aziridination of acyclic enones, as the product **3** **g**–**h** were obtained in good yields, high diastereoselectivity and moderate ee. To the best of our knowledge, this is the first example for the asymmetric aziridination of enones without the necessity of an ion‐paired catalyst featuring both chiral cation and anion. Similarly to our findings described in the previous chapter, our strategy provided an access to both product antipodes with essentially the same enantioselectivities (Scheme 5).

### Asymmetric Michael‐Addition‐Initiated Cascade Reactions

**Figure 2:**
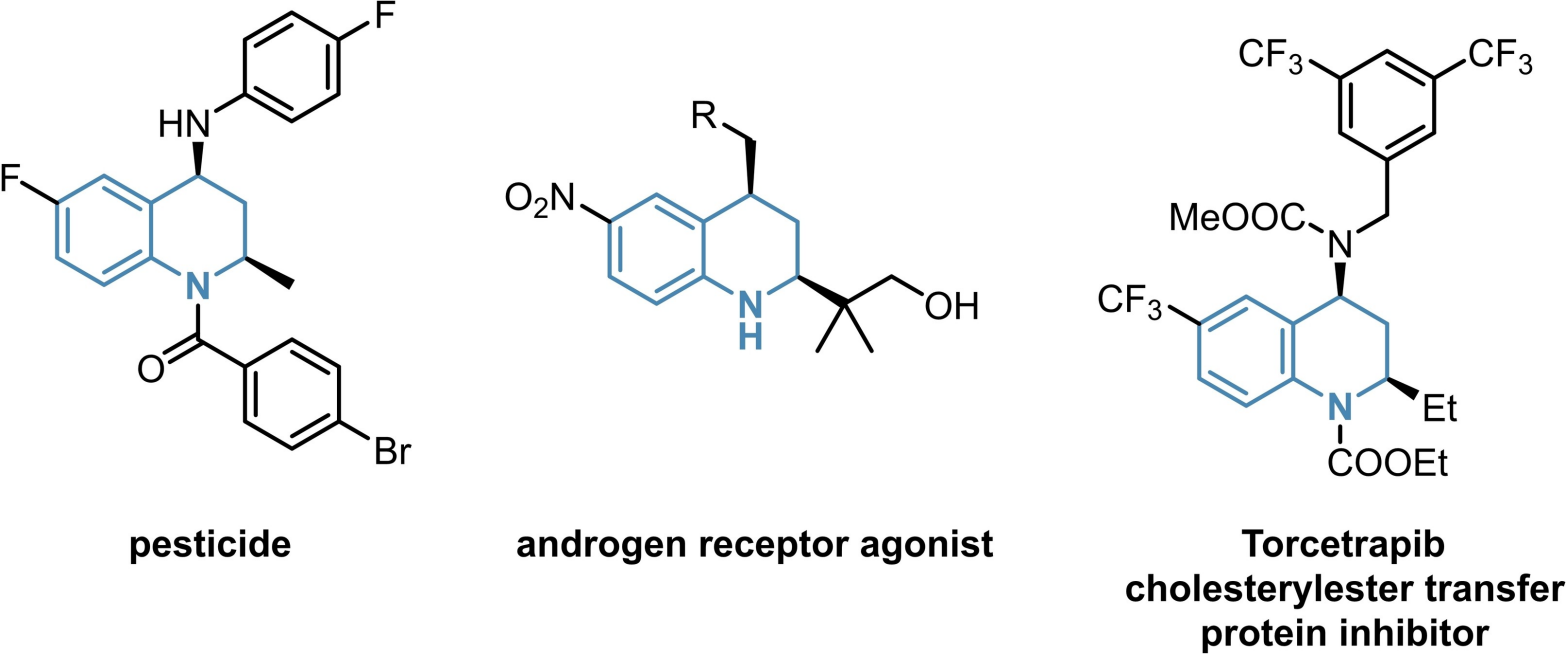
Examples of biologically active tetrahydroquinolines and acridins.

To further examine the versatility of our catalytic system, we also envisioned to expand our reaction scope to the direction of cascade‐cyclizations. We initially turned our attention towards the synthesis of octahydroacridines via a direct aza‐Michael/aldol sequence. While *N*‐heterocycles such as quinolines and acridines (Figure 2) are known to exert anti‐inflammatory, antiviral and anticancer activities,[^49^, ^50^, ^51^, ^52^] the application and activity of chiral octahydroacridins are not yet fully explored.^[53]^

**Scheme 6:**
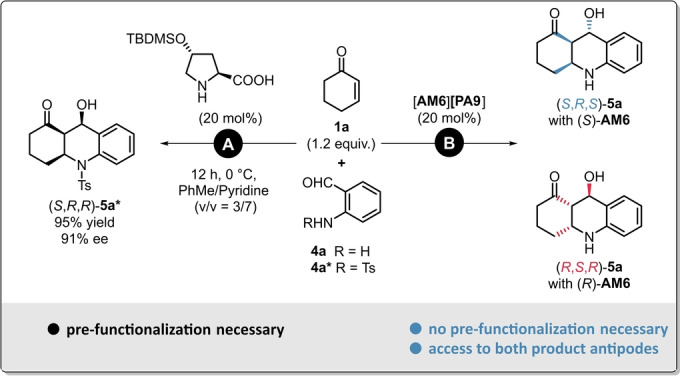
Different concepts for the synthesis of asymmetric octahydroacridins: Aza‐Michael/Aldol sequence by using pre‐functionalized reagents (**A**),^[39]^ and by our concept relying on a protecting‐group‐free reagent (**B**).

So far, the only procedure for the synthesis of such stable octahydroacridines was reported by the group of Yang in 2018. Even though they could use modified proline‐derivatives as elegant and reasonably affordable organocatalysts, the use of *N*‐tosyl‐protected reagents was found to be crucial for achieving high enantioselectivities (Scheme 6, **A**).^[53]^ Therefore, such method requires not just only additional pre‐functionalization, but it also leads to a less atom economic process. Consequently; to solve these aforementioned issues, we aimed for the development of a direct aza‐Michael/aldol sequence for the synthesis of octahydroacridines relying on protecting‐group‐free reagents (Scheme 6, **B**).

Initially, the reaction of **1** **a** and 2‐aminobenzaldehyde (**4** **a**) was investigated in the presence of our state‐of‐art organocatalyst [**AM6**][**PA9**] (Table 3). A short optimization showed that the amount of solvent was crucial and higher concentrations (>0.4 M) led to significantly lower ee values. The unprotected octahydroacridine **5** **a** product was found to be sensitive to acidic and basic environment, therefore they could not be isolated by conventional column chromatography. Nevertheless, when the reaction was performed in chloroform, the isolation of the partially precipitated product could be carried out by simple filtration, which provided the pure **5** **a** in 64 % yield and basically as a single enantiomer (>99 % ee, Table 3, entry 2). When the reaction was carried out in dioxane or toluene—resulting in homogeneous reaction conditions—**5** **a** could be still isolated in 96 % ee by a fast short‐path Pasteur‐column, indicating that the precipitation had eventually no effect on the enantioselectivity of the product (Table 3, entries 2 vs. 3, 4). Through additional control experiments we found that our catalyst [**AM6**][**PA9**] has indeed better performance for such transformations compared to state‐of‐art organocatalysts: the cinchona‐derivative (*S*,*S*)‐**AM13** and (*R*,*R*)‐**AM1** (DPEN) both gave high conversions; albeit, resulting in degradation products and in limited yields of 2–11 % for **5** **a** both with TFA and with **6** as counterion (Table 3, entries 5–11).

**Table 3:** Screening of different solvents and catalysts for the synthesis of octahydroacridine **5** **a**.

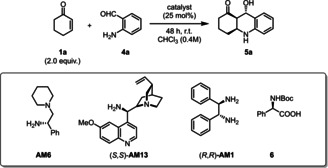					
					
Entry^[a]^	Amine	Acid	Conv. [%]^[b]^	Yield of **5** **a** ^[c]^	ee [%]^[d]^
1	**AM6**	none	trace	n.d.	n.d.
**2**	**AM6**	**PA9** (**1.0** equiv)	>**99**	**48** (**64**)	>**99**
3^[e]^	**AM6**	**PA9** (1.0 equiv)	79		96
4^[f]^	**AM6**	**PA9** (1.0 equiv)	90		96
5	**AM13**	**PA9** (1.0 equiv)	85	11	>99
6	**AM1**	**PA9** (1.0 equiv)	99	11	>99
7	**AM13**	TFA (1.0 equiv)	98	2	70
8	**AM13**	TFA (2.0 equiv)	88	n.d.	n.d.
9	**AM1**	TFA (1.0 equiv)	99	n.d.	n.d.
10	**AM13**	**6** (1.0 equiv)	>99	7	>99
11	**AM13**	**6** (2.0 equiv)	>99	18	>99

[a] Reactions were performed on a 0.2 mmol scale in 1 mL chloroform (0.2 M) at 25 °C for 48 hours. [b] Determined by GC‐MS analysis on a BGB 5 column. [c] Isolated yields after precipitation from the reaction mixture with *n*‐hexane/*i*‐PrOH. The yield in parenthesis corresponds to 1.0 mmol scale. [d] Determined by chiral HPLC on a Chiralpak® AS‐H column.

**Scheme 7:**
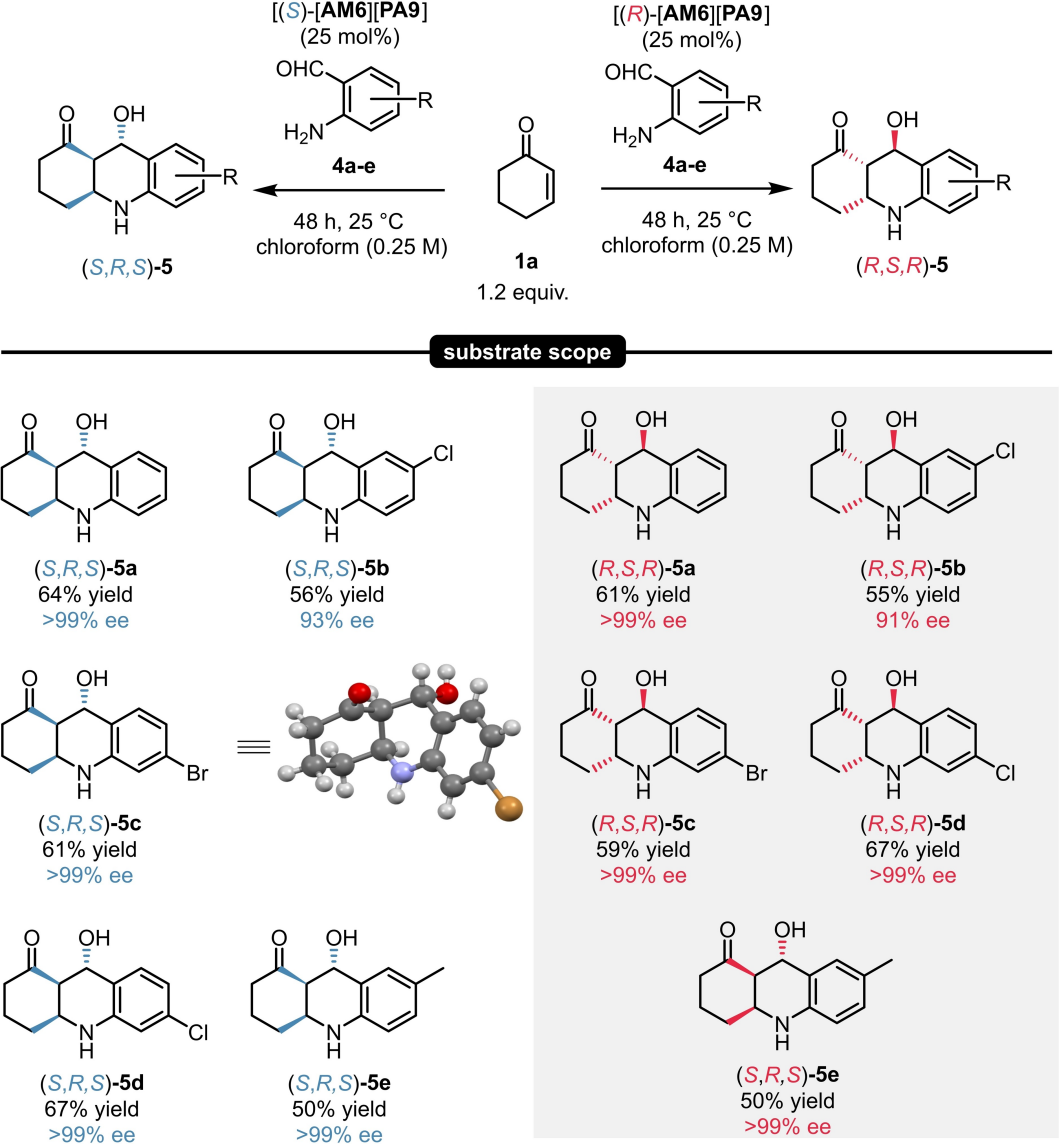
Substrate scope for the synthesis of octahydroacridines via direct aza‐michael‐aldol sequence. Reactions were performed with 1.0 mmol 2‐aminobenzaldeyhde (**4** **a**–**e**, 1.0 equiv), 1.2 equiv 2‐cyclohexen‐1‐one (**1** **a**) and 25 mol % catalyst [**AM6**][**PA9**] at 25 °C for 48 h. Yields refer to pure products after precipiation. Enantiomeric excess values were determined by chiral HPLC analysis on a Chiralpak® AS‐H column. The deposition number for the XRD of (*S*,*R*,*S*)‐**5** **c** is CCDC 2112845.^[36]^

With these promising results in hand, a small scope of five different compounds was explored. By using the catalyst [**AM6**][**PA9**], the octahydroacridine products **5** **a**–**e** could be isolated in 55–64 % yield and in excellent enantioselectivities of 93–>99 % ee. Similarly to our previous results, **5** **a**–**e** could be accessed in both enantiomeric forms (Scheme 7).

**Scheme 8:**
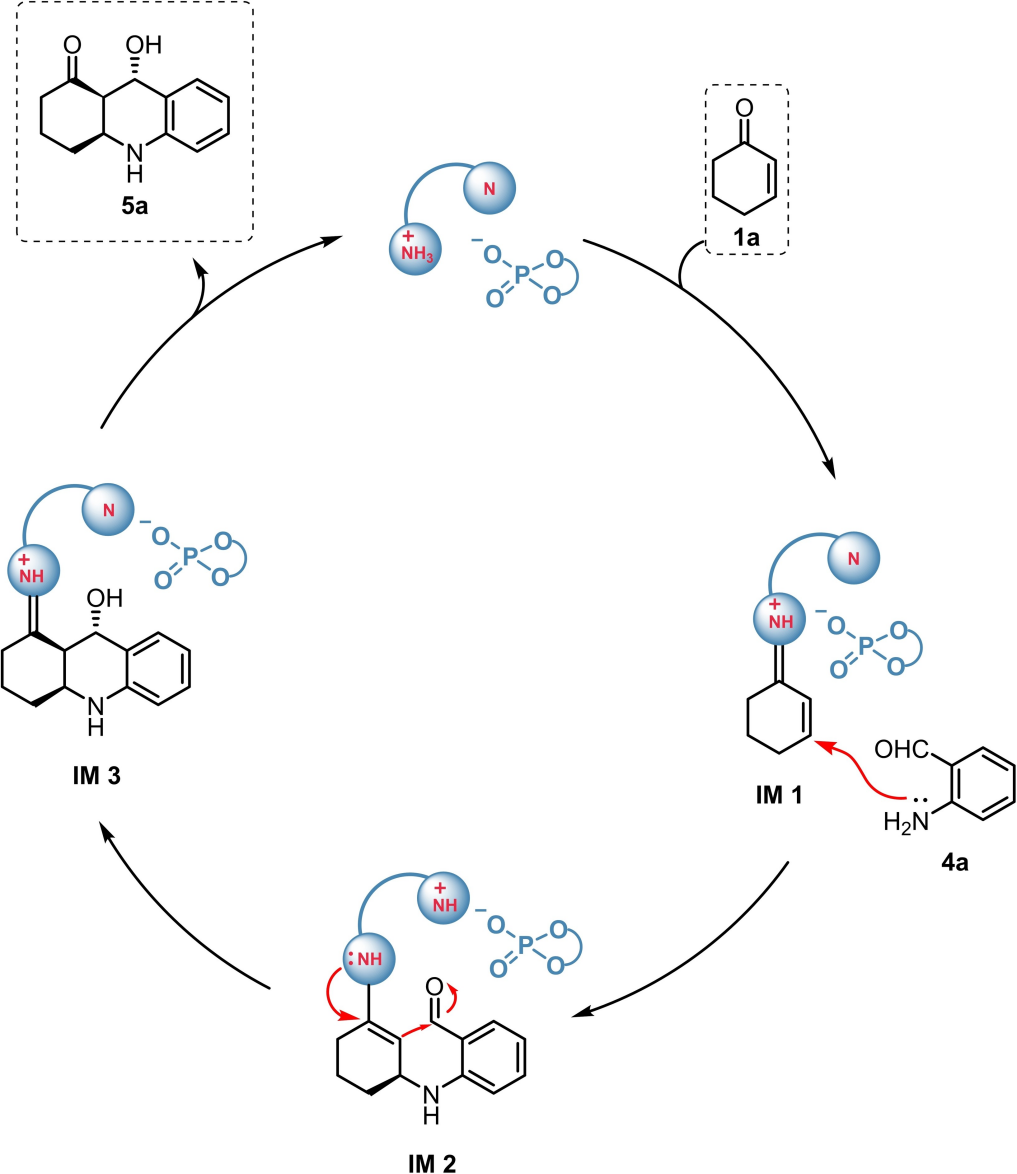
Plausible reaction mechanism for the aza‐Michael/aldol sequence, using **4** **a** as a representative reagent.

Herein, we propose the plausible reaction mechanism (Scheme 8). As a first step, the enone **1** **a** reacts with the aminocatalyst, resulting in the formation of the iminium intermediate **IM1**, which then undergoes a Michael‐addition with **4** **a**. The generated conjugated enamine (**IM2**) then initiates an intramolecular aldol reaction and the cyclization. Finally, hydrolysis of **IM3** leads to the desired product **5** **a**.

**Scheme 9:**
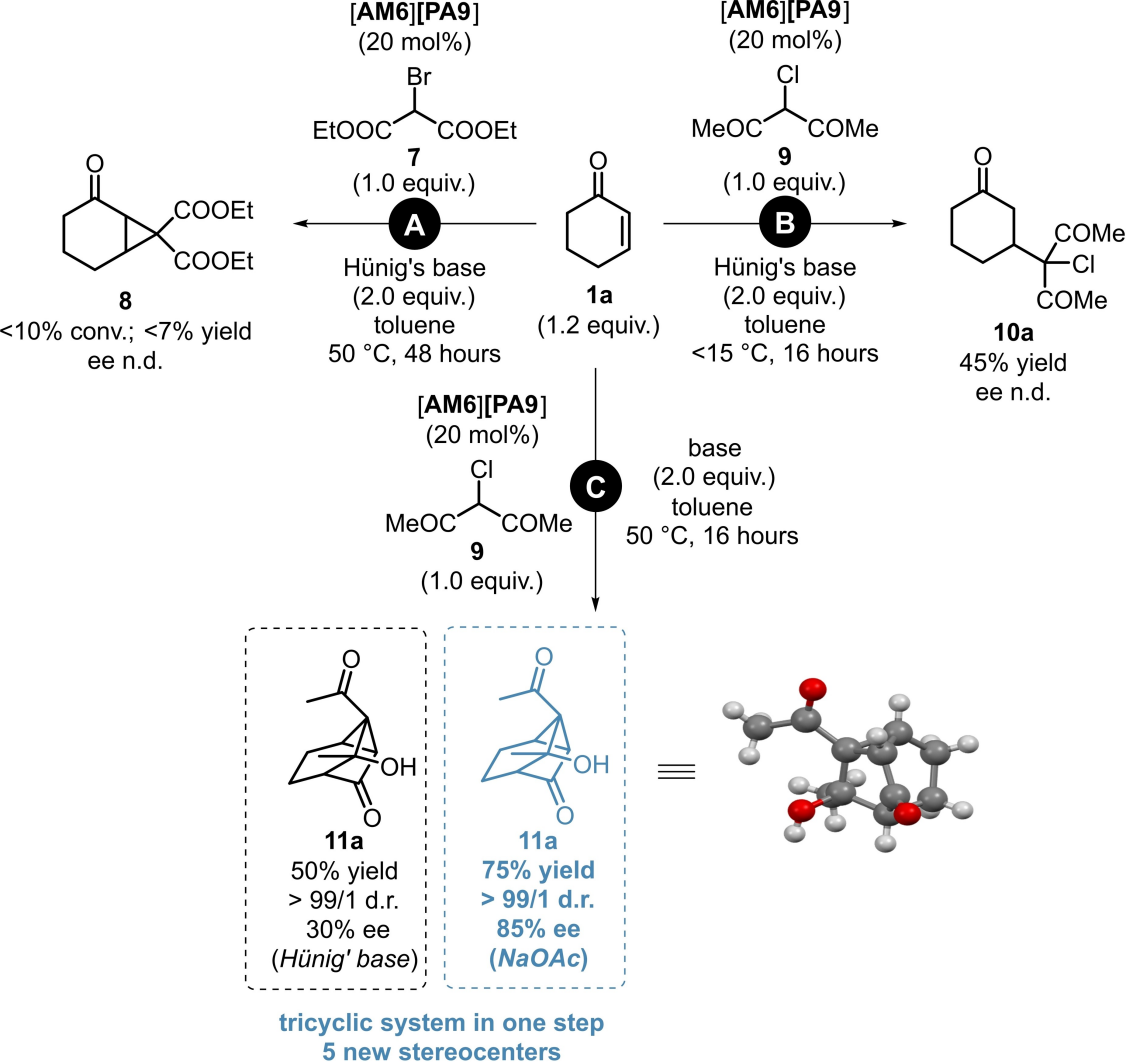
Towards the discovery of a novel Robinson‐like MIRC‐reaction/intramolecular aldol sequence: Initial trial for MIRC reaction (**A**), simple Michael addition under kinetic control (**B**) and the novel cyclization under thermodynamic control (**C**). Reactions were performed on a 1.0 mmol scale by using 1.2 equiv **1** **a**, 1.0 equiv reagent (**7** or **9**) and 2.0 equiv base in 5 mL toluene (0.2 M) at 15 °C or 50 °C for 16 hours. Yields refer to pure products after flash column chromatography. Enantiomeric excess values for **11** **a** were determined by chiral HPLC analysis on a Chiralpak® OJ column. The deposition number for the XRD of **11** **a** is CCDC 2112843.^[36]^

Being motivated to find yet another application for our powerful and multi‐purpose catalyst [**AM6**][**PA9**], we envisioned to apply it in Michael‐Initiated Ring Closure (MIRC) reactions of **1** **a**, by initially aiming for the synthesis of the cyclopropane derivative **8**. Despite several attempts using diethyl‐2‐bromomalonate (**7**) as reagent, the reaction was found to be extremely sluggish even at 50 °C and only traces of product **8** could be observed (Scheme 9, **A**). When a similar reagent, 3‐cloroacetylacetone (**9**) was applied, the Michael‐product **10** **a** was formed in moderate yields under kinetic contol; meanwhile, no cyclopropane‐formation was observed (Scheme 9, **B**). However; we discovered an unprecedented Robinson‐like annulation sequence when using 3‐chloroacetylacetone (**9**) as reagent in the presence of *N*,*N*‐diisopropylethylamine at 50 °C under thermodynamic control. The reaction afforded the tricyclic product **11** **a** with five new stereocenters in a promising 50 % isolated yield and in a moderate 30 % ee (Scheme 9, **C**, black). Moreover, this result could be significantly improved when using NaOAc as acid‐scavenger, as **11** **a** was obtained in 75 % yield, as a single diastreomer and in 85 % ee (Scheme 9, **C**).

**Scheme 10:**
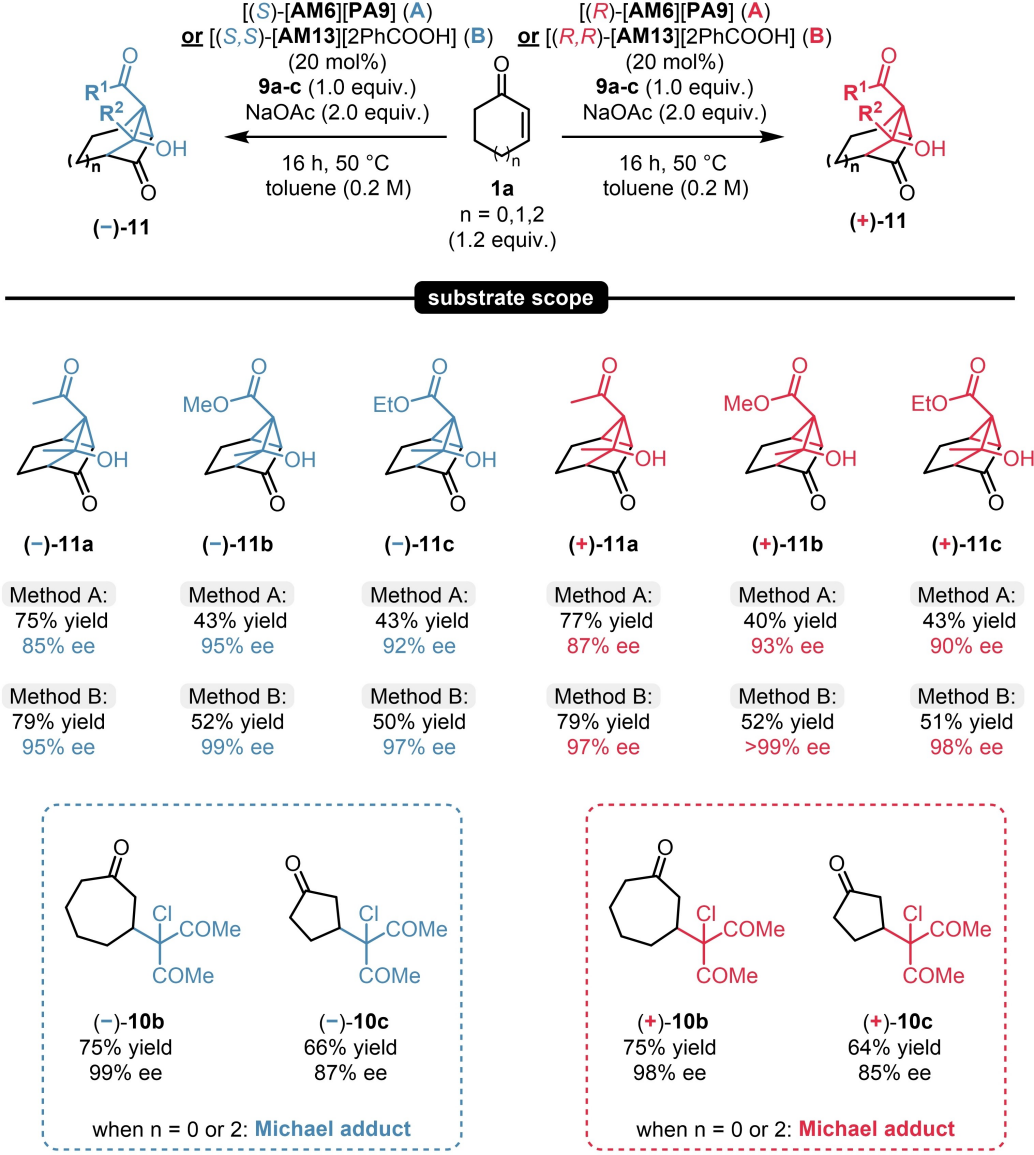
Scope of the MIRC/aldol sequence. Reactions were performed with 1.0 mmol chlorinated reagent (**9** **a**–**c**, 1.0 equiv), 1.2 equiv 2‐cyclohexen‐1‐one (**1** **a**) and 25 mol % catalyst [**AM6**][**PA9**] or [**AM13**][**2PhCOOH**] at 50 °C for 16 h. Yields refer to pure products after isolation by column chromatography. Enantiomeric excess values were determined by chiral HPLC analysis on a Chiralpak® OJ column.

Even though these results were already quite promising, we aimed for further improving this process. Gratifyingly, when changing to the classical quinine and quinidine‐derived organocatalysts [**AM13**][**2PhCOOH**], even better results were obtained: The product **11** **a** could be obtained in 79 % yield, as a single diastereomer, and in an excellent 95 % ee and 97 % ee for the (−) and (+)‐antipodes; respectively (see entries for **11** **a** in Scheme 10). We then aimed to explore the possible limitations and reaction scope of such MIRC/aldol annulation sequence. We found that the reaction was limited to the six‐membered substrate **1** **a**, as the reaction with other cyclic enones resulted in the formation of the corresponding Michael adducts **10** **b**–**c**. Nevertheless, the reaction scope could be easily expanded by using readily available chlorinated β‐ketoester reagents, resulting in **11** **b**–**c** in excellent enantioselectivities for both product antipodes by using both organocatalytic strategies with [**AM6**][**PA9**] and [**AM13**][**2PhCOOH**] (Scheme 10).

**Scheme 11:**
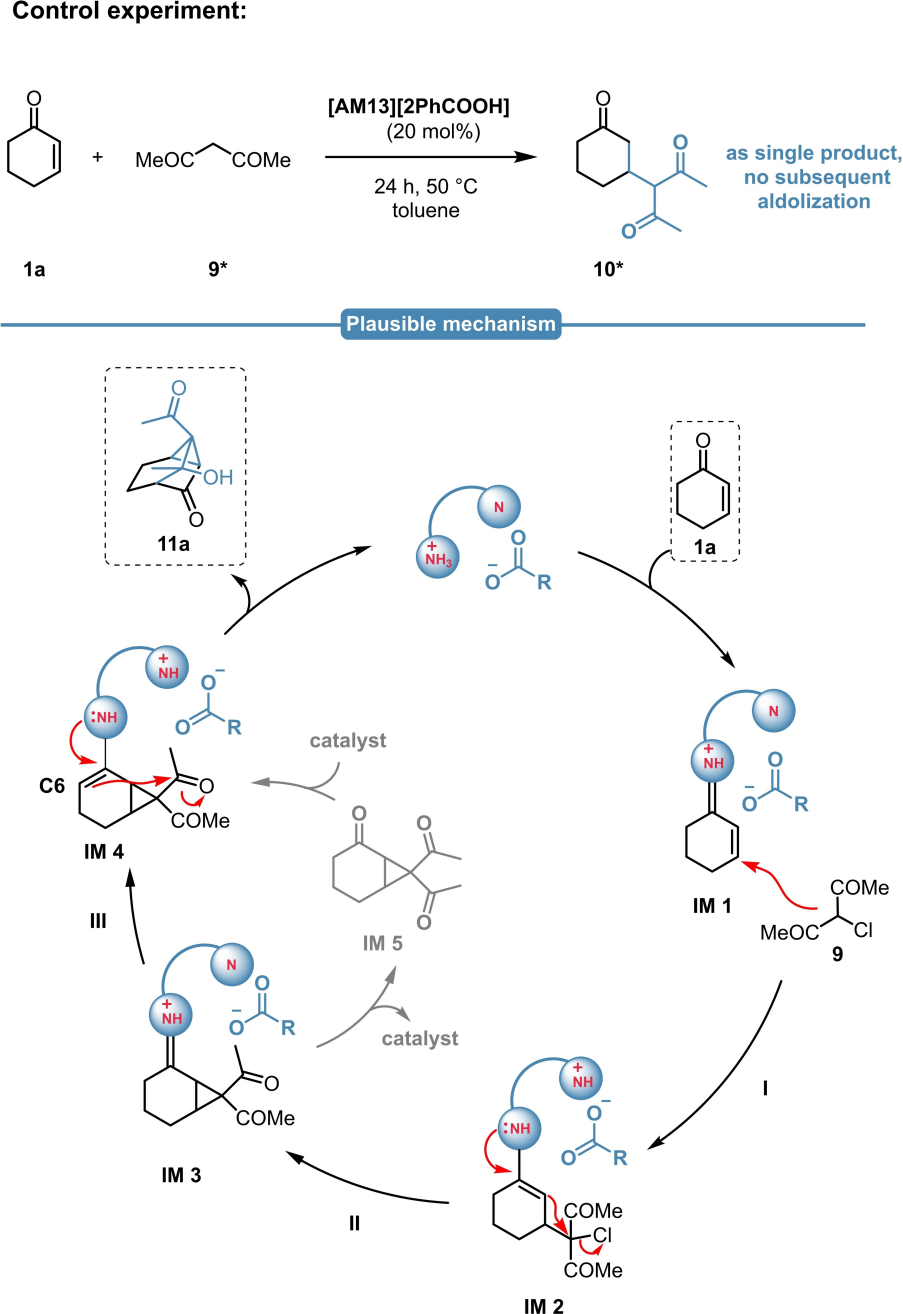
Control experiment with 9* (top) and the plausible reaction mechanism of the MIRC/aldol sequence (bottom).

The control reaction with acetylacetone (9*) resulted in the formation of the Michael adduct (10*) as single product and no sign for the subsequent intramolecular aldolization was detected (Scheme 11, top). This might suggest, that the cyclopropane intermediate is indeed required for an ideal geometry for the subsequent second ring closing step, which makes a concurrent aldolization/cyclopropanation pathway unlikely. Based on these experimental findings, herein we propose a plausible mechanism for the MIRC/aldol sequence (Scheme 11, bottom). At first, the catalyst reacts with the enone **1** **a**, resulting in the formation of the imunium intermediate **IM1**, which then undergoes a Michael‐addition with **9** (**I**). Through the conjugated enamine **IM2** this is followed by the subsequent cyclopropane ring closure (**II**), resulting in **IM3**. This step might be followed by a direct intramolecular proton shuttle (**III**) and the generated enamine **IM4** subsequently initiates the intramolecular aldol reaction, yielding the tricyclic product **11** **a**. This is in accordance with our experimental observations, as the corresponding cyclopropane (**IM5**) could be never isolated; however, its formation cannot be completely outruled.

## Conclusion

Herein we reported a novel concept of counterion enhanced catalysis for various asymmetric functionalization of enones. By combining sterically demanding flexible phosphoric acids with amino‐acid‐derived chiral diamines, simple and highly tunable organocatalytic frameworks were obtained. After a careful parameter optimization, a particularly powerful and multi‐purpose organocatalyst was found, which could be successfully applied in asymmetric epoxidations, aziridinations, aza‐Michael/aldol cascade cyclization, as well as in a Robinson‐like asymmetric MIRC/aldol cyclization. For all four reaction types, excellent enantioselectivities were obtained, providing an easy access to both product enantiomers in all cases. Further investigations focusing on broadening the scope of our catalytic concept, especially for complex cyclizations are currently ongoing in our research group.

## Conflict of interest

The authors declare no conflict of interest.

1

## Supporting information

As a service to our authors and readers, this journal provides supporting information supplied by the authors. Such materials are peer reviewed and may be re‐organized for online delivery, but are not copy‐edited or typeset. Technical support issues arising from supporting information (other than missing files) should be addressed to the authors.

Supporting Information

## Data Availability

The data that support the findings of this study are available from the corresponding author upon reasonable request.
