# Supplementary material for: Sterically Demanding Flexible Phosphoric Acids for Constructing Efficient and Multi‐Purpose Asymmetric Organocatalysts
Source: Angew Chem Int Ed Engl. 2022 Apr 26;61(26):e202202189. doi: 10.1002/anie.202202189 (PMC9324080; doi:10.1002/anie.202202189)
Supplement: Supplementary file 1 — Supporting Information [file ANIE-61-0-s001.pdf]

Angewandte Chemie
Eine Zeitschrift der Gesellschaft Deutscher Chemiker

Supporting Information

Sterically Demanding Flexible Phosphoric Acids for Constructing Efficient and Multi-Purpose Asymmetric Organocatalysts

*F. Scharinger, Á. M. Pálvölgyi, M. Weisz, M. Weil, C. Stanetty, M. Schnürch, K. Bica-Schröder**

Supporting Information

Sterically Demanding Flexible Phosphoric Acids for Constructing Efficient and Multi-Purpose Asymmetric Organocatalysts

Fabian Scharinger[a], Ádám Márk Pálvölgyi[a], Melanie Weisz[a], Matthias Weil[b], Christian Stanetty[a], Michael Schnürch[a] and Katharina Bica-Schröder*[a]

[a] Institute of Applied Synthetic Chemistry, TU Wien, Getreidemarkt 9/163, 1060 Wien, Austria

[b] Institute of Chemical Technologies and Analytics, TU Wien, Getreidemarkt 9/163, 1060 Wien, Austria

Supporting Information

TABLE OF CONTENTS

GENERAL REMARKS 3

1. PREPARATION OF ETHYL ACETAMIDOCYANOACETATE (OXYMA, 13) 4

2. PREPARATION OF DIAMINES 5

Step 1: Boc-protection 5
Step 2: DCC/DMAP/Oxyma coupling 6
Step 3: Deprotection 7
Step 4: Reduction 8

3. SYNTHESIS OF THE PHOSPHORIC ACIDS 11

Preparation of 1,1´-biphenols *via* Friedel-Crafts alkylation 12
Preparation of 23 *via* oxidative coupling 14
General procedure to all phosphorylations and hydrolysis 14

4. SUBSTRATE SYNTHESIS 18

5. PARAMETER OPTIMIZATION FOR ASYMMETRIC EPOXIDATIONS 21

5.1. Preliminary phosphoric acid screening 21
5.2 Amino-modification screening 22
5.3 Amino acid screening 23
5.4 Solvent screening 24
5.5 Final phosphoric acid screening, screening of conventional acids 25

6. GENERAL PROCEDURE AND ANALYTICAL DATA FOR THE ASYMMETRIC EPOXIDATIONS 26

7. PARAMETER OPTIMIZATION FOR THE ASYMMETRIC AZIRIDINATIONS 35

7.1 Solvent screening 35
7.2 Base screening 36

8. GENERAL PROCEDURE AND ANALYTICAL DATA FOR THE ASYMMETRIC AZIRIDINATIONS 37

9. GENERAL PROCEDURE AND ANALYTICAL DATA FOR THE ASYMMETRIC AZA-MICHAEL ADDITIONS 42

10. GENERAL PROCEDURE AND ANALYTICAL DATA FOR THE ASYMMETRIC MICHAEL-INITIATED RING-CLOSURE/INTRAMOLECULAR ALDOL SEQUENCE 46

11. CHIRAL HPLC AND CHIRAL GC CHROMATOGRAMS FOR THE ASYMMETRIC EPOXIDATIONS 49

12. CHIRAL HPLC CHROMATOGRAMS FOR THE ASYMMETRIC AZIRIDINATIONS 66

13. CHIRAL HPLC CHROMATOGRAMS FOR THE ASYMMETRIC AZA-MICHAEL/ALDOL REACTIONS 74

14. CHIRAL HPLC CHROMATOGRAMS FOR THE ASYMMETRIC MICHAEL-INITIATED RING CLOSURE/INTRAMOLECULAR ALDOL SEQUENCE 80

15. NMR SPECTRA OF CHIRAL DIAMINES 84

16. NMR SPECTRA OF PHOSPHORIC ACIDS AND INTERMEDIATES 93

17. NMR SPECTRA OF LITERATURE UNKNOWN ENONE SUBSTRATES 109

18. NMR SPECTRA FOR THE ASYMMETRIC EPOXIDATIONS 111

19. NMR SPECTRA FOR THE ASYMMETRIC AZIRIDINATIONS 128

20. NMR SPECTRA OF ASYMMETRIC AZA-MICHAEL/ALDOL REACTIONS 136

21. NMR SPECTRA OF ASYMMETRIC MICHAEL-INDUCED RING-CLOSURE/ALDOL REACTION SEQUENCE 144

22. EXTENDED 1D/2D-NMR EXPERIMENTS SUPPORTING THE STRUCTURE OF COMPOUND 11 148

23. NMR STUDIES FOR PHOSPHORIC ACIDS 153

24. XRD DATA FOR COMPOUNDS 5C, 11A AND PA9 160

Supporting Information

General remarks

All purchased chemicals from commercial suppliers were used without further purification, unless noted otherwise. Dry solvents were pre-distilled and desiccated on aluminium oxide columns (PURESOLV, Innovative Technology).

Column chromatography was performed on standard manual glass columns using Merck (40-60 µm) silica gel with pre-distilled solvents (PE: petrolether, EtOAc: ethyl acetate, Et_2O: diethyl ether). For TLC analysis, precoated aluminium-backed plates were purchased from Merck (silica gel 60 F_{254}). UV active compounds were detected at 254 nm. Non-UV active compounds have been detected using vanillin staining solution (5% vanillin in EtOH + H_2SO_4).

^{1}H, ^{13}C, ^{19}F and ^{31}P NMR spectra were recorded on a Bruker Advance UltraShield 200 MHz or 400 MHz spectrometer and chemical shifts are reported in ppm using TMS (tetramethylsilane) as internal standard. Coupling constants (*J*) are given in Hz. For assignation purpose, the following abbreviations are used: s (singlet), d (doublet), t (triplet), q (quartet), m (multiplet), brs (broad singlet), dd (doublet of doublets), ddd (doublets of doublet of doublets), td (triplet of doublets), dt (doublet of triplets).

GC measurement have been performed on a Thermo Scientific Focus on BGB5 column by using FID detector. Chiral GC measurements were performed on chiral BGB columns (BGB 173, BGB 175) by using FID detector.

GC-MS measurements have been performed on a Thermo Scientific DSQ II on BGB5 column (30m), equipped with a quadrupole MS detector DSQ II.

Chiral HPLC measurements were carried out on a DAIONEX UPLC equipped with a photodiode array (PDA) plus detector (190–360 nm), using a Diacel Chiracel AS-H, IB and OJ columns (all 250 × 4.60 mm, 5 µm).
Optical rotation was measured on an Anton Paar MCP500 polarimeter at the specific conditions and the results have been compared to literature values. Concentrations are given in g / 100 mL.

HR-MS analysis was performed using HTC PAL system auto sampler, an Agilent 1100/1200 HPLC and Agilent 6230 AJS ESI-TOF mass spectrometer.

Microwave reactions were performed on a Biotage Initiator Classic in 20 ml pressure tight glass vials.
Melting points above room temperature were measured on an automated melting point system OPTI MELT of Stanford Research Systems and are uncorrected.

Infrared spectra were recorded on a Perkin-Elmer Spectrum 65 FT IR spectrometer equipped with a specac MK II Golden Gate Single Reflection ATR unit.

1. Preparation of ethyl acetamidocyanoacetate (oxyma, 13)

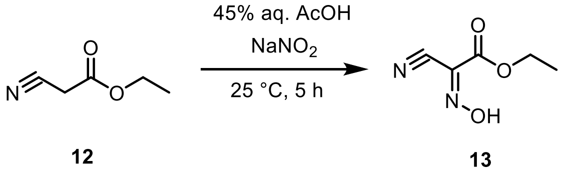

According to literature procedure.[1] A solution of ethyl cyanoacetate (**12**, 10 g, 88.0 mmol, 1.0 equiv.) in aqueous acetic acid (45 w/w %, 40 mL) was stirred at 0 °C for 15 min. Sodium nitrite (18.3 g, 265.0 mmol, 3.0 equiv.) was added portion-wise and the reaction mixture was stirred 18 h at room temperature. Et_2O (50 mL) and water were added until a clear solution was obtained. The aqueous phase was extracted with Et_2O (2×), the combined organic phases were washed with water (2×), brine (1×) and dried over anhydrous Na_2SO_4. Removal of the solvent gave **13** as a slight yellow solid (12.3 g, 98% yield). **^{1}H NMR (400 MHz, $CDCl_3$) δ** 4.45 (q, J = 7.1 Hz, 2H), 1.41 (t, J = 7.1 Hz, 3H). The O*H* proton was not detected.

2. Preparation of diamines

Diamines **AM2**-**AM12** were prepared according to a four-step synthetic procedure, as depicted in Scheme S1.

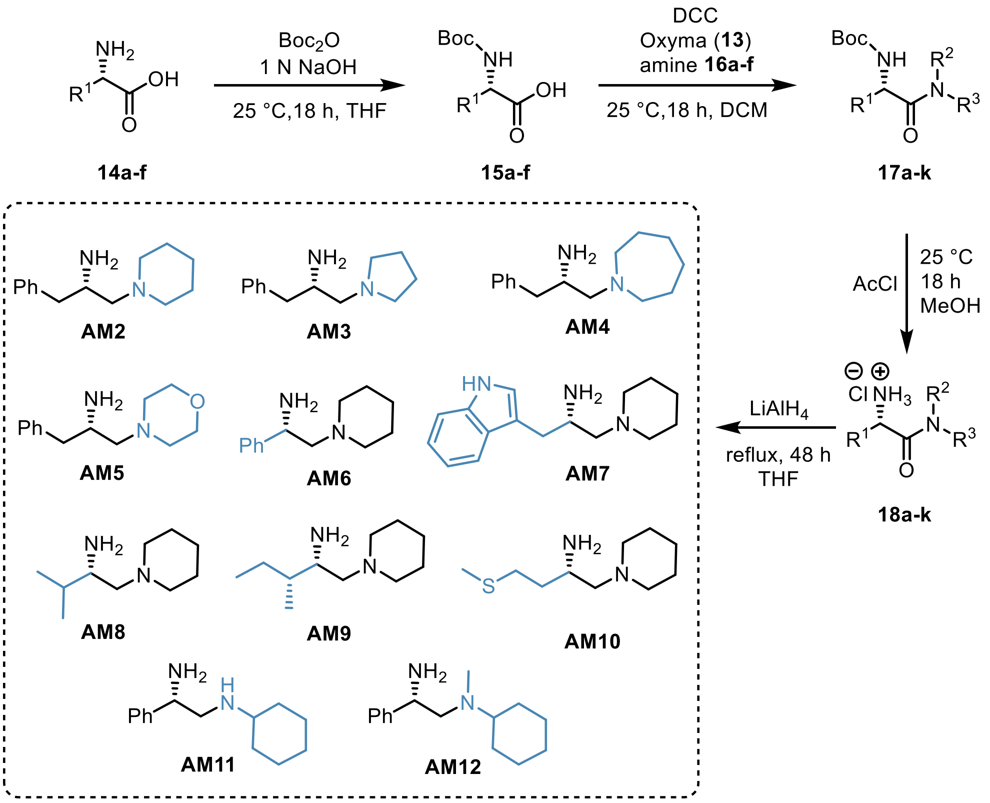

Scheme S1. General procedure for the synthesis of diamines **AM2-AM12.**

Step 1: Boc-protection

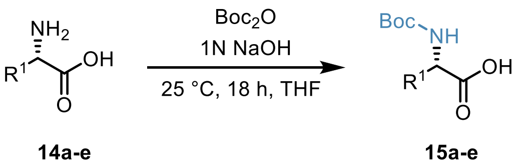

General procedure[2]: To the solution of amino acid **14a-e** (1.0 equiv.) in a mixture of THF / 1 N NaOH (1:1, 0.4 M), di-*tert*-butyl-dicarbonate (1.2 equiv.) was added. The solution was stirred for 18 h at room temperature. The THF was removed *in vacuo*, the resulting aqueous solution was diluted with DCM and it was acidified to pH 2 with a 1 N HCl solution. The aqueous phase was extracted with DCM (3×), the combined organic phases were washed with brine and dried over anhydrous Na_2SO_4. Removal of the solvent afforded the product **15a-e** as a white solid or colorless, viscous liquid.

N-Boc-L-tryptophan (**15f**) was bought from Fluka and it was used without further purifications.

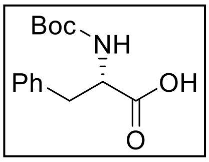

(*tert*-butoxycarbonyl)-L-Phenylalanine, 15a.[3] Performed on a 30.0 mmol scale. Viscous liquid (8.0 g, 99% yield). **^{1}H NMR (200 MHz, CDCl$_3$)** δ 7.18 – 7.31 (m, 5H), 4.94 (d, *J* = 8.3 Hz, 1H), 4.61 (d, *J* = 8.3 Hz, 1H), 3.09 – 3.25 (m, 2H), 1.42 (s, 9H). The COO*H* proton was not detected.

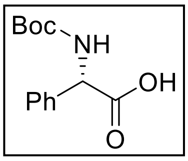

(*tert*-butoxycarbonyl)-L-Phenyglycine, (*S*)-**15b.**[4] Performed on a 17.0 mmol scale. White powder (4.1 g, 99% yield). **^{1}H NMR (200 MHz, CDCl$_3$) δ** 7.51 (brs, 1H), 7.29 – 7.43 (m, 5H), 5.13 (d, *J* = 8.1 Hz, 1H), 1.40 (s, 9H). The COO*H* proton was not detected.

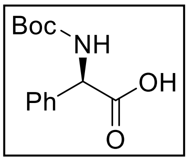

(*tert*-butoxycarbonyl)-D-Phenyglycine, (*R*)-**15b.**[5] Performed on a 19.8 mmol scale. White powder (4.79 g, 96% yield). **^{1}H NMR (200 MHz, CDCl$_3$) δ** 7.50 (brs, 1H), 7.29 – 7.42 (m, 5H), 5.13 (d, *J* = 8.1 Hz, 1H), 1.40 (s, 9H). The COO*H* proton was not detected.

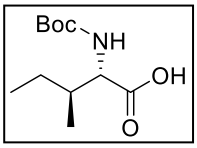

(*tert*-butoxycarbonyl)-L-Isoleucine, 15c.[6] Performed on a 24.0 mmol scale. Viscous liquid (5.5 g, 99% yield). **^{1}H NMR (200 MHz, CDCl$_3$) δ** 3.48 (brs, 1H), 2.40 – 2.73 (m, 1H), 1.83 – 2.05 (m, 1H), 1.48 – 1.60 (m, 2H), 1.43 (s, 9H), 0.90 – 1.20 (m, 6H). The COO*H* proton was not detected.

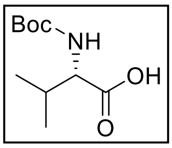

(*tert*-butoxycarbonyl)-L-Valine, 15d.[3] Performed on a 51.0 mmol scale. Viscous liquid (10.5 g, 94% yield). **^{1}H NMR (200 MHz, CDCl$_3$) δ** 5.05 (d, *J* = 9.1 Hz, 1H), 4.25 (dd, *J* = 8.8, 4.3 Hz, 1H), 2.09 – 2.26 (m, 1H), 1.44 (s, 9H), 0.99 (d, *J* = 6.8 Hz, 3H), 0.92 (d, *J* = 6.9 Hz, 3H). The COO*H* proton was not detected.

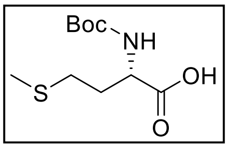

(*tert*-butoxycarbonyl)-L-Methionine, 15e.[7] Performed on a 14.0 mmol scale. Viscous liquid (3.4 g, >99% yield). **^{1}H NMR (200 MHz, CDCl$_3$) δ** 5.17 (brs, 1H), 4.43 (brs, 1H), 2.58 (dd, 8.4, 6.2 Hz, 2H), 2.11 (s, 3H), 1.97 (m, 2H), 1.44 (s, 9H). The COO*H* proton was not detected.

Step 2: DCC/DMAP/Oxyma coupling

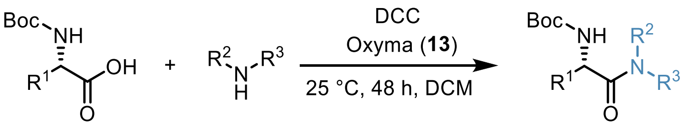

15a-f **16a-f** **17a-k**

General procedure:[8] To a solution of the Boc-protected amino acid **15a-f** (1.0 equiv.) in dry DCM (0.2 M), DCC (2.0 equiv.), oxyma **13** (1.1 equiv.) and amine **16a-f** (2.5 equiv.) were added at 0 °C. The

resulting turbid solution was stirred for another 15 min at 0 °C, allowed to warm up and it was stirred at room temperature for 48 h. Then cold EtOAc (50 mL) was added, the mixture was stirred for 10 min, the DCU was filtered off and the clear solution was concentrated *in vacuo*. The crude mixture was re-dissolved in EtOAc (25 mL) and it was washed with 1 N HCl (1×). The formed solid was filtered off and the filtrate was repeatedly washed with 1 N HCl (2×), 1 N NaOH (3×) and brine (1×). The combined organic phases were dried over anhydrous Na_2SO_4. Solvent removal afforded the crude products **17a-i** as white solids, which were used without further purification with the exception of (*S*)-**17b** and (*R*)-**17b**.

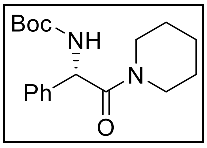

***tert*-butyl (*S*)-(2-Oxo-1-phenyl-2-(piperidin-1-yl)ethyl)carbamate**, (*S*)-**17b.**[9] Performed on a 17.0 mmol scale. Purified by column chromatography (30% EE in PE, R_f = 0.38). White solid (2.0 g, 79% yield). **^{1}H NMR (200 MHz, $CDCl_3$) δ** 7.38 – 7.16 (m, 5H), 6.08 (d, *J* = 7.7 Hz, 1H), 5.51 (d, *J* = 7.7 Hz, 1H), 3.66 (dt, *J* = 11.5, 4.2 Hz, 1H), 3.78 – 3.13 (m, 4H), 1.54 – 1.27 (m, 14H).

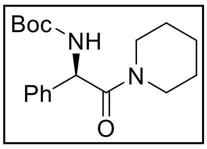

***tert*-butyl (*R*)-(2-Oxo-1-phenyl-2-(piperidin-1-yl)ethyl)carbamate**, (*R*)-**17b.**[9] Performed on a 17.0 mmol scale. Purified by column chromatography (30% EE in PE, R_f = 0.38). White solid (2.1 g, 80% yield). **^{1}H NMR (200 MHz, $CDCl_3$) δ** 7.37 – 7.17 (m, 5H), 6.08 (d, *J* = 7.7 Hz, 1H), 5.51 (d, *J* = 7.7 Hz, 1H), 3.66 (dt, *J* = 11.5, 4.2 Hz, 1H), 3.78 – 3.13 (m, 4H), 1.56 – 1.27 (m, 14H).

Step 3: Deprotection

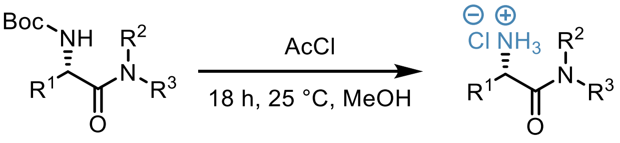

17a-i **18a-i**

General procedure:[8] To a solution of Boc-amide **17a-i** (1.0 equiv.) in dry MeOH (0.41 M), acetyl chloride (8.0 equiv.) was added at 0 °C. After 15 min, the ice bath was removed and the solution was stirred for another 18 h at 25 ˚C. The solvent was removed and the solid was dried under reduced pressure (1 h, 0.7 mbar). Et_2O was added to the solid and the dispersion was stirred for 15 min. The product was filtered off. The crude products **18a-i** were used directly for the next step without further purifications.

Supporting Information

Step 4: Reduction

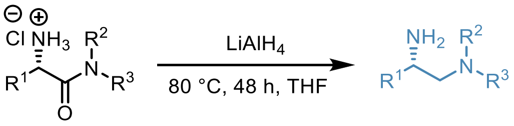

18a-i **AM2-AM10**

General procedure:[10] To a stirred solution of salt **18a-i** (1.0 equiv.) in THF (0.13-0.15 M) $LiAlH_4$ (5.0 equiv.) was added in small portions at 0 °C. The resulting dispersion was stirred for 15 min and it was subsequently stirred at reflux for 48 h. After cooling to room temperature, distilled H_2O (1 mL per g $LiAlH_4$), and 15 % aqueous NaOH (2 mL per g $LiAlH_4$) were added at 0 °C and the mixture was stirred for 10 min. Anhydrous Na_2SO_4 was added to remove the previously added water, and the product was taken up in EtOAc. The solid was filtered off and it was washed with EtOAc. The filtrate was washed with 1 N NaOH (2×) and brine (1×) and it was dried over anhydrous Na_2SO_4. Removal of the solvent – and if necessary, column chromatography – afforded the pure product **AM2-AM10** as yellow oil or yellow solid.

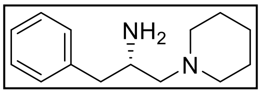

(*S*)-1-Phenyl-3-(piperidin-1-yl)propan-2-amine, AM2.[11] Performed on a 3.0 mmol scale. Yellow oil (0.60 g, 85% yield). **^{1}H NMR (400 MHz, $CDCl_3$) δ** 7.32 – 7.25 (m, 2H), 7.23 – 7.16 (m, 3H), 3.66 – 3.59 (m, 4H), 3.23 – 3.11 (m, 1H), 2.71 (dd, *J* = 13.3, 4.5 Hz, 1H), 2.54 – 2.41 (m, 3H), 2.33 – 2.15 (m, 4H), 1.44 (brs, 2H).

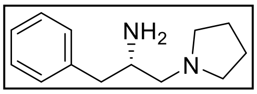

(*S*)-1-Phenyl-3-(pyrrolidin-1-yl)propan-2-amine, AM3.[12] Performed on a 4.0 mmol scale. Purified by column chromatography (15% MeOH in EtOAc + 2% Et_3N, R_f = 0.23). Yellow oil (0.44 g, 59% yield). **^{1}H NMR (200 MHz, $CDCl_3$) δ** 7.39 – 7.16 (m, 5H), 3.27 – 3.09 (m, 1H), 2.79 (dd, *J* = 13.3, 4.5 Hz, 1H), 2.65 – 2.42 (m, 6H), 2.34 (dd, *J* = 11.8, 4.3 Hz, 1H), 1.77 – 1.61 (m, 4H), 1.46 (brs, 2H).

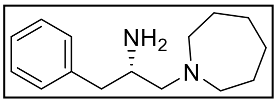

(*S*)-1-(Azepan-1-yl)-3-phenylpropan-2-amine, AM4.[12] Performed on a 3.0 mmol scale. Yellow oil (0.62 g, 99% yield). **^{1}H NMR (400 MHz, $CDCl_3$) δ** 7.34 – 7.15 (m, 5H), 3.13 – 3.03 (m, 1H), 2.76 – 2.42 (m, 7H), 2.32 – 2.24 (m, 1H), 1.71 – 1.49 (m, 10H).

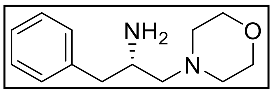

(*S*)-1-Morpholino-3-phenylpropan-2-amine, AM5.[13] Performed on a 4.0 mmol scale. White solid (0.35 g, 45% yield). **^{1}H NMR (400 MHz, $CDCl_3$) δ** 7.34 – 7.14 (m, 5H), 3.78 – 3.59 (m, 4H), 3.26 – 3.13 (m, 1H), 2.73 (dd, *J* = 13.3, 4.5 Hz, 1H), 2.59 – 2.45 (m, 3H), 2.42 – 2.20 (m, 4H), 1.51 (brs, 2H).

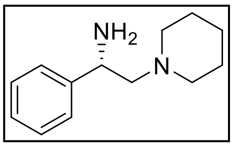

(*S*)-1-Phenyl-2-(piperidin-1-yl)ethan-1-amine, (*S*)-AM6.[14] Performed on a 6.1 mmol scale. Purified by column chromatography (10% MeOH in DCM + 0.8% Et_3N, R_f = 0.25). Yellow oil (0.99 g, 77% yield). **^{1}H NMR (200 MHz, $CDCl_3$) δ** 7.42 – 7.16 (m, 5H), 4.10 (dd, *J* = 9.6, 4.4 Hz, 1H), 2.67 – 2.19 (m, 6H), 1.89 (s, 2H), 1.73 – 1.50 (m, 4H), 1.49 – 1.33 (m, 2H).

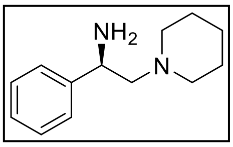

(*R*)-1-Phenyl-2-(piperidin-1-yl)ethan-1-amine, (*R*)-AM6.[14] Performed on a 6.1 mmol scale. Purified by column chromatography (10% MeOH in DCM + 0.8% Et_3N, R_f = 0.25). Yellow oil (0.95 g, 76% yield). **^{1}H NMR (200 MHz, $CDCl_3$) δ** 7.42 – 7.16 (m, 5H), 4.10 (dd, *J* = 9.6, 4.4 Hz, 1H), 2.67 – 2.19 (m, 6H), 1.89 (s, 2H), 1.73 – 1.50 (m, 4H), 1.49 – 1.33 (m, 2H).

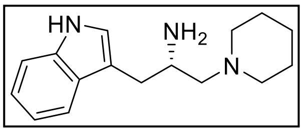

(*S*)-1-(1*H*-Indol-3-yl)-3-(piperidin-1-yl)propan-2-amine, AM7. Performed on a 5.0 mmol scale. Purified by column chromatography (10% MeOH in DCM + 0.8% Et_3N, R_f = 0.21). Yellow solid (0.37 g, 30% yield). $[\alpha]_D^{20}$ = + 11.60 (c 0.96, CH_2Cl_2). **^{1}H NMR (400 MHz, $CDCl_3$) δ** 8.44 (s, 1H), 7.62 (dt, *J* = 7.9, 1.0 Hz, 1H), 7.36 (dt, *J* = 8.1, 1.0 Hz, 1H), 7.18 (ddd, *J* = 8.1, 7.0, 1.3 Hz, 1H), 7.11 (ddd, *J* = 8.0, 7.0, 1.1 Hz, 1H), 7.06 (s, 1H), 3.38 – 3.26 (m, 1H), 2.90 (ddd, *J* = 14.2, 4.5, 0.9 Hz, 1H), 2.83 (d, *J* = 12.0, 2H), 2.66 (dd, *J* = 14.2, 8.4 Hz, 2H), 2.40 – 2.21 (m, 6H), 1.56 (pd, *J* = 6.0, 3.6 Hz, 4H), 1.42 (p, *J* = 6.0 Hz, 2H). **^{13}C NMR (101 MHz, $CDCl_3$) δ** 136.52, 127.85, 122.83, 122.00, 119.32, 119.09, 113.08, 111.31, 65.87, 55.23, 48.34, 31.55, 26.23, 24.59. **IR ATR (vmax/cm^{-1})** 3367, 2917, 2839, 1499, 1355. **HRMS (ESI)** Cald for $C_{16}H_{24}N_3$ $[M+H]^+$ 258.1965, Found 258.1972.

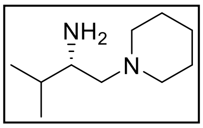

(*S*)-3-Methyl-1-(piperidin-1-yl)butan-2-amine, AM8.[14] Performed on a 4.0 mmol scale. Light yellow oil (0.60 g, 80% yield). **^{1}H NMR (400 MHz, $CDCl_3$) δ** 2.66 (ddd, *J* = 10.3, 5.6, 3.4 Hz, 1H), 2.16 – 2.10 (m, 2H), 2.46 (s, 2H), 2.06 – 2.00 (m, 2H), 1.63 – 1.45 (m, 7H), 1.44 – 1.35 (m, 2H), 0.89 (d, *J* = 6.8 Hz, 6H).

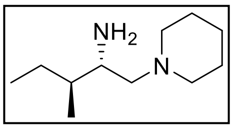

(2*S*,3*S*)-3-Methyl-1-(piperidin-1-yl)pentan-2-amine, AM9.[15] Performed on a 3.0 mmol scale. Dark yellow oil (0.48 g, 96% yield). **^{1}H NMR (400 MHz, $CDCl_3$) δ** 2.76 (ddd, *J* = 10.5, 5.6, 3.2 Hz, 1H), 2.53 – 2.35 (m, 2H), 2.23 – 1.85 (m, 5H), 1.57 – 1.35 (m, 7H), 1.32 – 1.07 (m, 3H), 0.95 – 0.80 (m, 6H). **^{13}C NMR (101 MHz, $CDCl_3$) δ** 63.46, 51.80, 39.26, 26.35, 25.42, 24.70, 15.20, 11.72.

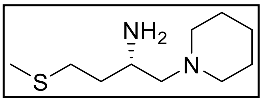

(*S*)-4-(Methylthio)-1-(piperidin-1-yl)butan-2-amine, AM10. Performed on a 5.0 mmol scale. Red oil (0.56 g, 60% yield). $[\alpha]_D^{20}$ = + 7.19 (c 1.0, CH_2Cl_2). **^{1}H NMR (400 MHz, $CDCl_3$) δ** 2.99 (ddt, *J* = 9.6, 8.4, 4.2 Hz, 1H), 2.65 – 2.38 (m, 4H), 2.26 – 2.09 (m, 4H), 2.07 (s, 3H), 1.68 – 1.32 (m, 10H). **^{13}C NMR (101 MHz, $CDCl_3$) δ** 66.29, 55.14, 46.79, 35.28, 31.29, 26.26, 24.59, 15.62. **IR ATR (vmax/cm^{-1})** 2928, 2852, 1439. **HRMS (ESI)** Cald for $C_{10}H_{23}N_2S$ $[M+H]^+$ 203.1576, Found 203.1576.

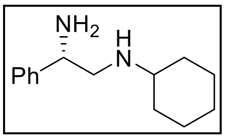

(*S*)-*N*^{1}-Cyclohexyl-2-phenylethane-1,2-diamine, AM11. Performed on a 5.0 mmol scale. Red oil (0.60 g, 58% yield). $[\alpha]_D^{20}$ = + 44.37 (c 0.93, CH_2Cl_2). ^{1}H NMR (400 MHz, **$CDCl_3$**) δ 7.26 – 7.21 (m, 3H), 7.21 – 7.12 (m, 2H), 3.90 (dd, *J* = 8.5, 4.8 Hz, 1H), 2.78 (dd, *J* = 11.7, 4.8 Hz, 1H), 2.63 (dd, *J* = 11.7, 8.5 Hz, 1H), 2.33 (tt, *J* = 10.4, 3.8 Hz, 1H), 1.77 (dddd, *J* = 10.8, 9.3, 3.5, 1.5 Hz, 2H), 1.68 – 1.58 (m, 3H), 1.56 – 1.47 (m, 2H), 1.19 – 1.03 (m, 4H), 1.01 – 0.91 (m, 3H). **^{13}C NMR (101 MHz, $CDCl_3$) δ** 145.17, 128.61, 127.23, 126.50, 56.89, 56.23, 55.12, 33.99, 33.78, 26.31, 25.17. **HRMS (ESI)** Cald for $C_{14}H_{23}N_2$ $[M+H]^+$ 219.1856, Found 219.1874.

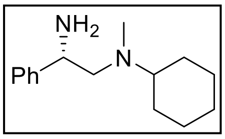

(*S*)-*N*^{1}-Cyclohexyl-N^1-methyl-2-phenylethane-1,2-diamine, AM12. Performed on a 5.0 mmol scale. Red oil (0.46 g, 42% yield). $[\alpha]_D^{20}$ = + 33.17 (c 0.75, CH_2Cl_2). **^{1}H NMR (200 MHz, $CDCl_3$) δ** 7.41 – 7.07 (m, 5H), 3.98 (t, *J* = 6.9 Hz, 1H), 2.48 – 2.32 (m, 2H), 2.26 (s, 3H), 1.89 (s, 2H), 1.80 – 1.46 (m, 5H), 1.33 – 0.92 (m, 5H). **^{13}C NMR (101 MHz, $CDCl_3$) δ** 144.74, 128.32, 126.99, 126.74, 63.77, 62.72, 53.48, 37.84, 29.17, 28.31, 26.39, 26.11, 26.10. **HRMS (ESI)** Cald for $C_{15}H_{25}N_2$ $[M+H]^+$ 233.2012, Found 233.2014.

3. Synthesis of the phosphoric acids

Phosphoric acids **PA5**-**PA9** were prepared according to a two-step procedure *via* Friedel-Crafts alkylation and subsequent phosphorylation (Scheme S2, **I.**). Phosphoric acid **PA3** was prepared according to two-step procedure *via* oxidative coupling and subsequent phosphorylation (Scheme S2, **II.**). Phosphoric acids **PA1**-**PA2** and **PA4** were prepared *via* direct phosphorylation of commercially available diols **24**-**26** (Scheme S2, **III.**).

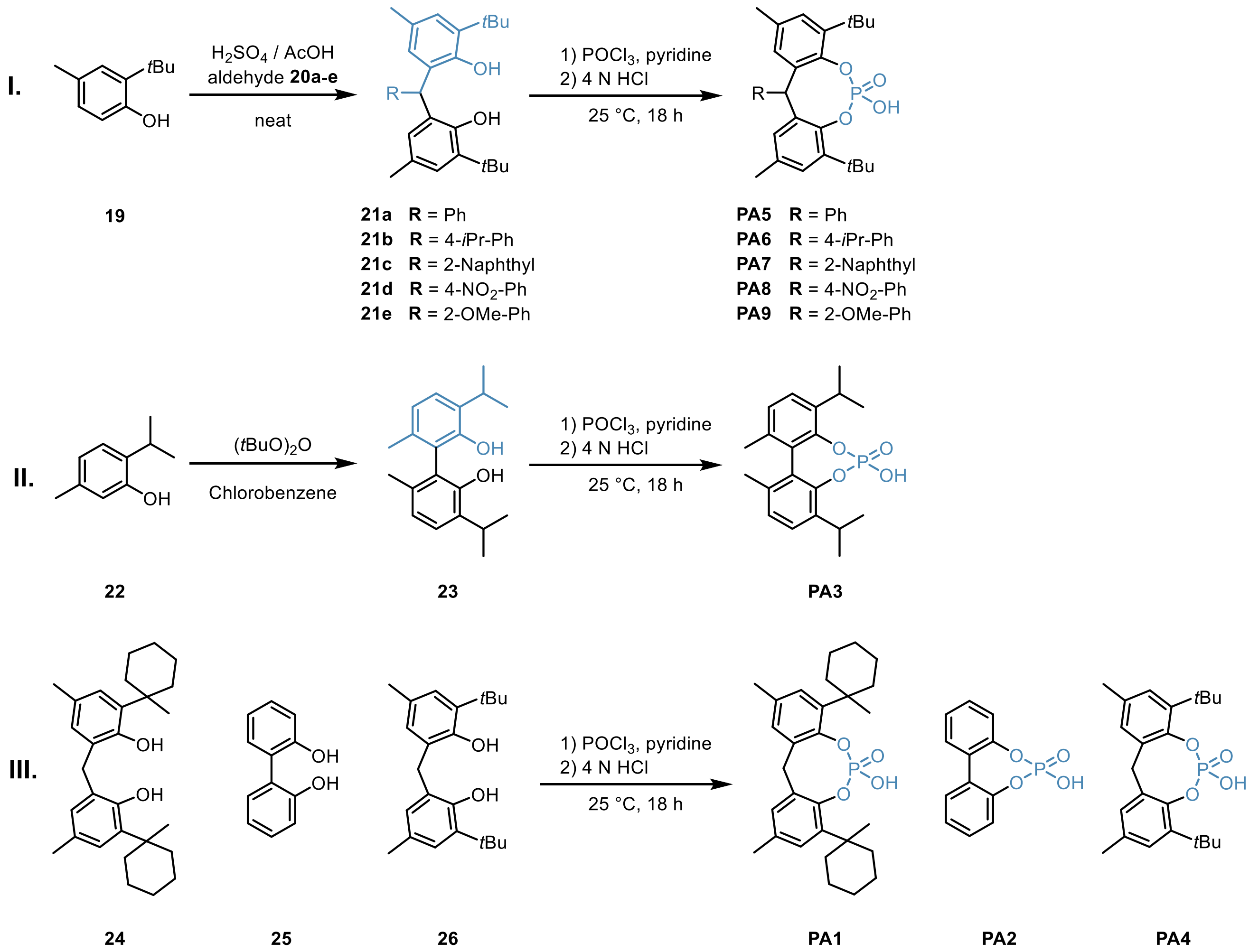

Scheme S2. General procedure for the synthesis of phosphoric acids.

Supporting Information

Preparation of 1,1´-biphenols *via* Friedel-Crafts alkylation

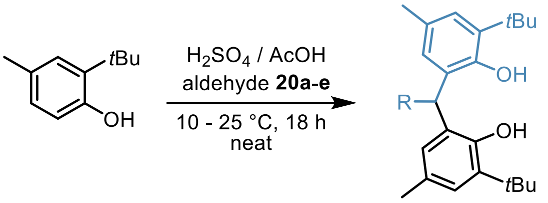

19

21a **R** = Ph
21b **R** = 4-*i*Pr-Ph
21c **R** = 2-Naphthyl
21d **R** = 4-NO_2-Ph
21e **R** = 2-OMe-Ph

General procedure:[16] To a solution of phenol **19** (2.0 equiv.) in acetic acid (0.58 M), aldehyde **20a-e** (1.0 equiv.) was added at room temperature. Then concentrated sulfuric acid (1.3 equiv.) was added dropwise over about 7-10 °C (*excessive cooling below this point might result in the freezing of acetic acid!*) and the mixture was stirred for 15 min. The cooling bath was removed and the solution was stirred for 18 h at room temperature. Distilled H_2O was added, the precipitate was filtered off and it was subsequently washed with distilled H_2O until the filtrate became colourless. Purification of the crude products (noted below) afforded **21a-e** as white or slightly coloured solid.

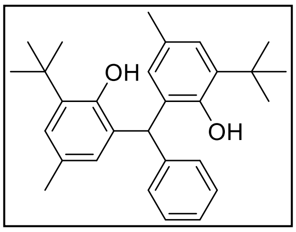

6,6'-(Phenylmethylene)bis(2-(*tert*-butyl)-4-methylphenol), 21a.[16] Performed on a 6.0 mmol scale. Purified by washing the crude precipitate several times with cold *n*-hexane. White solid (1.4 g, 55% yield). **^{1}H NMR (200 MHz, $CDCl_3$) δ** 7.43 – 7.29 (m, 3H), 7.25 – 7.15 (m, 2H), 7.12 – 7.03 (d, *J* = 2.2 Hz, 2H), 6.50 (d, *J* = 2.2 Hz, 2H), 5.61 (s, 1H), 4.83 (bs, 2H), 2.19 (s, 6H), 1.38 (s, 18H).

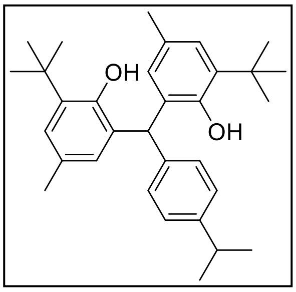

6,6'-((4-Isopropylphenyl)methylene)bis(2-(*tert*-butyl)-4-methylphenol), 21b. Performed on a 6.0 mmol scale. The crude precipitate was dissolved in DCM, washed with sat. $NaHCO_3$ (2×), brine (1×), and the combined organic phases were dried over anhydrous Na_2SO_4 and concentrated *in vacuo*. The crude product was purified by column chromatography (5% EtOAc in PE, R_f = 0.45). White solid (0.95 g, 34% yield). **M.p.** 177 – 178 °C. **^{1}H NMR (400 MHz, $CDCl_3$) δ** 7.21 (d, *J* = 8.2 Hz, 2H), 7.11 (d, *J* = 8.2 Hz, 2H), 7.05 (d, *J* = 2.2 Hz, 2H), 6.52 (d, *J* = 2.1 Hz, 2H), 5.54 (s, 1H), 4.81 (s, 2H), 2.93 (m, 1H), 2.19 (s, 6H), 1.38 (s, 18H), 1.27 (d, *J* = 6.9 Hz, 6H). **^{13}C NMR (101 MHz, $CDCl_3$) δ** 151.00, 148.07, 137.71, 137.64, 129.66, 129.46, 128.44, 127.95, 127.24, 127.21, 47.14, 34.77, 33.84, 29.92, 24.08, 21.20. **IR ATR (vmax/cm^{-1})** 3535, 3476, 2960, 1441, 1164. **HRMS (ESI)** Cald for $C_{32}H_{41}O_2$ $[M-H]^-$ 457.3112, Found 457.3114.

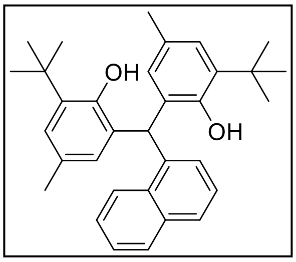

6,6'-(Naphthalen-1-ylmethylene)bis(2-(*tert*-butyl)-4-methylphenol), 21c. Performed on a 6.0 mmol scale. The crude precipitate was dissolved in DCM, extracted with sat. $NaHCO_3$ (2×), brine (1×), the combined organic phase dried over anhydrous Na_2SO_4 and concentrated. Purified by column chromatography (5 % EtOAc in PE, R_f = 0.41). White solid (1.48 g, 52% yield). **M.p.** 107 – 110 °C. **^{1}H NMR (200 MHz, $CDCl_3$) δ** 7.96 – 7.80 (m, 3H), 7.54 – 7.35 (m, 3H), 7.17 – 7.03 (m, 3H), 6.45 (d, *J* = 2.1 Hz, 2H), 6.31 (s, 1H), 4.73 (bs, 2H), 2.14 (s, 6H), 1.40 (s, 18H). **^{13}C NMR (101 MHz, $CDCl_3$) δ** 150.87, 137.77, 136.53, 134.24, 132.08, 129.79, 128.97, 128.52, 128.31, 128.10, 127.32, 127.18, 126.86, 126.02, 125.74, 124.14, 43.27, 34.78, 29.98, 21.19. **HRMS (ESI)** Cald for $C_{33}H_{37}O_2$ $[M-H]^-$ 465.2799, found 465.2796.

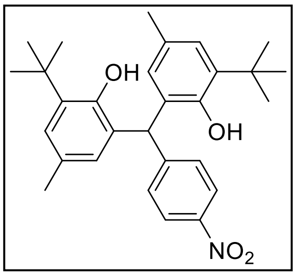

6,6'-((4-Nitrophenyl)methylene)bis(2-(*tert*-butyl)-4-methylphenol), 21d.[17] Performed on a 6.0 mmol scale. The crude precipitate was dissolved in DCM, washed with sat. $NaHCO_3$ (2×), brine (1×), and the combined organic phases were dried over anhydrous Na_2SO_4 and concentrated *in vacuo*. Pink solid (2.5 g, 89% yield). **M.p.** 212 – 214 °C. **^{1}H NMR (400 MHz, $CDCl_3$) δ** 8.24 – 8.11 (m, 2H), 7.34 (dd, *J* = 8.9, 0.7 Hz, 2H), 7.07 (d, *J* = 2.1 Hz, 2H), 6.52 – 6.43 (m, 2H), 5.82 (s, 1H), 4.91 (s, 2H), 2.20 (d, *J* = 0.7 Hz, 6H), 1.38 (s, 18H). **^{13}C NMR (101 MHz, $CDCl_3$) δ** 150.54, 149.79, 146.88, 137.33, 130.33, 130.00, 127.79, 127.65, 127.47, 123.80, 46.22, 34.49, 29.93, 21.05. **IR ATR (vmax/cm^{-1})** 3505, 2954, 1515, 1443, 1346. **HRMS (ESI)** Cald for $C_{29}H_{34}NO_4$ $[M-H]^-$ 460.2493, found 460.2491.

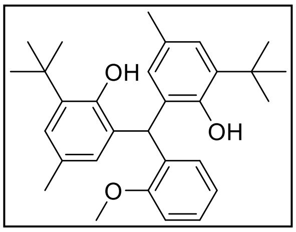

6,6'-((2-Methoxyphenyl)methylene)bis(2-(*tert*-butyl)-4-methylphenol), 21 e.[18] Performed on a 6.0 mmol scale. The crude precipitate was dissolved in DCM, washed with sat. $NaHCO_3$ (2×), brine (1×), and the combined organic phases were dried over anhydrous Na_2SO_4 and concentrated *in vacuo*. Purified by column chromatography (3 % EtOAc in PE, R_f = 0.40). White solid (1.6 g, 59% yield). The crude product could be also purified by recrystallization from either acetic acid or *n*-hexane, affording **21e** with the same purity. **M.p.** 172 – 174 °C. **^{1}H NMR (200 MHz, $CDCl_3$) δ** 7.37 – 7.21 (m, 1H), 7.09 – 6.86 (m, 5H), 6.55 (d, *J* = 2.1 Hz, 2H), 5.91 (s, 1H), 4.95 (s, 2H), 3.81 (s, 3H), 2.19 (s, 6H), 1.37 (s, 18H). **^{13}C NMR (101 MHz, $CDCl_3$) δ** 156.83, 151.17, 137.49, 130.77, 129.28, 128.73, 128.64, 127.63, 127.43, 127.10, 121.37, 111.05, 55.90, 39.73, 34.82, 29.88, 21.21. **IR ATR (vmax/cm^{-1})** 3537, 2949, 1486, 1440, 1178, 1106. **HRMS (ESI)** Cald for $C_{30}H_{37}O_3$ $[M-H]^-$ 445.2748, found 445.2765.

Preparation of 23 *via* oxidative coupling

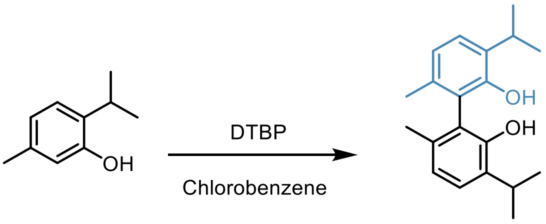

22 **23**

According to modified literature procedure:[19] In a 20 mL microwave vial equipped with a stir bar, the phenol **22** (0.73 g, 4.85 mmol, 1.0 equiv.) was dissolved in chlorobenzene (1.66 M). Di-*tert*-butyl peroxide (DTBP, 0.93 mL, 5.10 mmol, 1.05 equiv.) was then added *via* syringe. The vial was capped and the reaction mixture was stirred for 15 min at room temperature. The vial was then placed in the microwave reactor and heated to 160 °C (*high absorption setting!*) and stirred for 15 min. The reaction mixture was allowed to cool down and volatiles have been removed under reduced pressure. The resulting crude mixture was purified by column chromatography (2% EtOAc in PE, R_f = 0.30). White solid (1.1 g, 76% yield). **^{1}H NMR (400 MHz, $CDCl_3$) δ** 7.19 (d, *J* = 7.7 Hz, 2H), 6.89 (d, *J* = 7.8 Hz, 2H), 4.76 (s, 2H), 3.32 – 3.25 (m, 2H), 1.96 (s, 6H), 1.26 (dd, *J* = 6.9, 2.7 Hz, 12H).

General procedure to all phosphorylations and hydrolysis

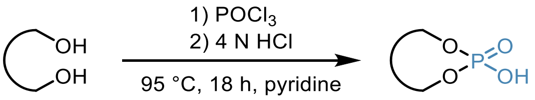

21a-e, 23-26 **PA1-PA9**

According to literature procedure:[20] To a solution of the diol **21a-e**, **23-26** (1.0 equiv.) in pyridine (0.34 M), $POCl_3$ (2.0 equiv.) was added slowly *via* syringe at 0 °C. After stirring the reaction mixture for 24 h at 95 °C, it was cooled to room temperature and H_2O (25 equiv.) was added slowly. The resulting clear solution was stirred for another 18 h at 95 °C. After cooling to room temperature, 4 N HCl was added slowly. The precipitate was filtered off and it was washed with 4 N HCl. The filtrate was extracted with DCM, and the organic phase was washed several times with 4 N HCl (3×), brine (3×), H_2O and it was dried over anhydrous Na_2SO_4. Removal of the solvent afforded **PA1**-**PA9**.

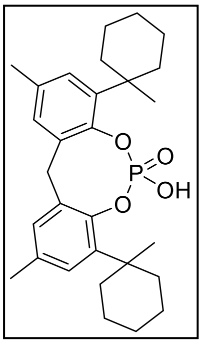

6-Hydroxy-2,10-dimethyl-4,8-bis(1-methylcyclohexyl)-12*H*-dibenzo[d,g] [1,3,2] dioxaphosphocine 6-oxide, PA1. Performed on a 7.1 mmol scale. White powder (3.4 g, 98% yield). **M.p.** 264 – 266 °C. **^{1}H NMR (400 MHz, $CDCl_3$) δ** 7.06 (t, *J* = 1.9 Hz, 2H), 6.96 (d, *J* = 2.1 Hz, 2H), 4.06 (s, 2H), 2.29 (s, 6H), 2.17 (dd, *J* = 13.4, 7.9 Hz, 4H), 1.69 – 1.52 (m, 8H), 1.44 (t, *J* = 9.1 Hz, 8H), 1.30 (s, 6H). The COO*H* proton was not detected. **^{13}C NMR (101 MHz, $CDCl_3$) δ** 147.79, 147.70, 139.60, 139.53, 134.26, 130.79, 130.76, 129.55, 129.53, 128.17, 38.62, 37.64, 35.39, 27.09, 26.62, 22.96, 21.15. **^{31}P NMR (162 MHz, $CDCl_3$) δ** −9.53. **IR ATR (vmax/cm^{-1})** 2922, 2856, 1604, 1440, 1194, 1018, 967. **HRMS (ESI)** Cald for $C_{29}H_{38}O_4P$ [M-H]$^-$ 481.2513, found 481.2514.

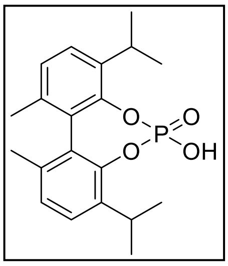

6-Hydroxy-4,8-diisopropyl-1,11-dimethyldibenzo[d,f][1,3,2]dioxaphosphepine-6-oxide, PA3. Performed on a 6.7 mmol scale. Brown foam (2.0 g, 83% yield). **^{1}H NMR (400 MHz, $CDCl_3$) δ** 8.35 (brs, 1H), 7.27 (d, *J* = 8.0 Hz, 2H), 7.15 (d, *J* = 8.0 Hz, 2H), 3.49-3.45 (m, 2H), 2.14 (s, 6H), 1.27 (dd, *J* = 25.5, 6.9 Hz, 12H). **^{31}P NMR (162 MHz, $CDCl_3$) δ** 2.14.

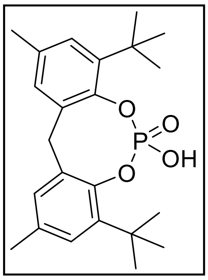

4,8-Di-*tert*-butyl-6-hydroxy-2,10-dimethyl-12*H*-dibenzo[d,g][1,3,2]dioxaphosphocine-6-oxide, PA4.[21] Performed on a 9.0 mmol scale. White powder (3.6 g, >99% yield). **^{1}H NMR (400 MHz, $CDCl_3$) δ** 12.35 (brs, 1H), 7.04 (d, *J* = 1.5 Hz, 2H), 6.96 (dd, *J* = 2.1, 0.8 Hz, 2H), 4.05 (s, 2H), 2.28 (s, 6H), 1.41 (s, 18H). **^{31}P NMR (162 MHz, $CDCl_3$) δ** −9.64.

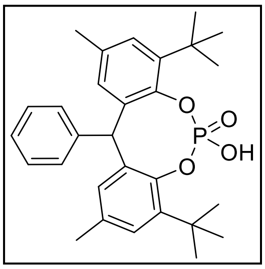

4,8-Di-*tert*-butyl-6-hydroxy-2,10-dimethyl-12-phenyl-12*H*-dibenzo [d,g]-[1,3,2]dioxaphosphocine 6-oxide, PA5. Performed on a 0.7 mmol scale. White powder (0.3 g, 87% yield). **M.p.** 254 – 256 °C. **^{1}H NMR (400 MHz, DMSO-d_6) δ** 7.26 (dd, *J* = 8.3, 6.6 Hz, 2H), 7.22 – 7.16 (m, 1H), 7.13 – 7.05 (m, 4H), 7.03 – 6.97 (m, 2H), 6.03 (s, 1H), 2.23 (s, 6H), 1.39 (s, 18H). The COO*H* proton was not detected. **^{13}C NMR (101 MHz, DMSO-d_6) δ** 146.64 (d, *J* = 7.8 Hz), 141.91, 140.86 (d, *J* = 4.7 Hz), 135.26 (d, *J* = 3.1 Hz), 133.37 (d, *J* = 1.7 Hz), 128.04, 126.98, 34.80, 31.10. **^{31}P NMR (162 MHz, DMSO-d_6) δ** −12.85. **IR ATR (vmax/cm^{-1})** 2959, 1739, 1602, 1439, 1011, 932. **HRMS (ESI)** Cald for $C_{29}H_{34}O_4P$ [M-H]$^-$ 477.2200, found 477.2201.

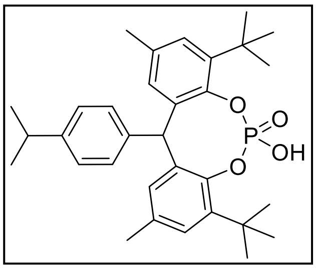

4,8-Di-*tert*-butyl-6-hydroxy-12-(4-isopropylphenyl)-2,10-dimethyl-12*H*-dibenzo[d,g][1,3,2]dioxaphosphocine 6-oxide, PA6. Performed on a 2.0 mmol scale. White powder (0.91 g, 89% yield). **M.p.** 212 – 215 °C. **^{1}H NMR (400 MHz, $CDCl_3$) δ** 7.20 (d, *J* = 8.1 Hz, 2H), 7.14 (d, *J* = 8.2 Hz, 2H), 7.03 (s, 4H), 6.27 (s, 1H), 2.89 (h, *J* = 6.9 Hz, 1H), 2.25 (s, 6H), 1.41 (s, 19H), 1.26 (d, *J* = 6.9 Hz, 6H). The COO*H* proton was not detected. **^{13}C NMR (101 MHz, $CDCl_3$) δ** 147.31, 147.22, 146.87, 140.84, 140.78, 138.09, 134.42, 134.06, 129.53, 128.16, 127.06, 126.13, 35.07, 33.76, 31.11, 24.15, 21.39. **^{31}P NMR (162 MHz, $CDCl_3$) δ** −10.48. **IR ATR (vmax/cm^{-1})** 2960, 1502, 1512, 1442, 1218, 1106, 1018, 939. **HRMS (ESI)** Cald for $C_{32}H_{40}O_4P$ [M-H]$^-$ 519.2670, found 519.2670.

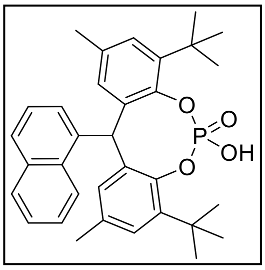

4,8-Di-*tert*-butyl-6-hydroxy-2,10-dimethyl-12-(naphthalen-1-yl)-12*H* -dibenzo [d,g][1,3,2]dioxaphosphocine 6-oxide, PA7. Performed on a 3.2 mmol scale. White powder (1.1 g, 67% yield). **M.p.** 284 – 287 °C (decomp.). **^{1}H NMR (400 MHz, $CDCl_3$) δ** 7.81 – 7.67 (m, 4H), 7.39 – 7.25 (m, 2H), 7.20 – 7.13 (m, 1H), 6.95 (t, *J* = 2.0 Hz, 2H), 6.86 (s, 1H), 6.75 (d, *J* = 2.2 Hz, 2H), 2.10 (s, 6H), 1.35 (s, 18H). The COO*H* proton was not detected. **^{13}C NMR (101 MHz, $CDCl_3$) δ** 147.42, 147.32, 140.81, 140.75, 135.20, 134.41, 133.93, 133.91, 133.58, 133.55, 132.75, 128.66, 128.60, 128.07, 126.93, 126.40, 126.37, 125.72, 125.53, 124.78, 40.43, 35.05, 30.86, 21.40. **^{31}P NMR (162 MHz, $CDCl_3$) δ** −9.10. **IR ATR (vmax/cm^{-1})** 2950, 1601, 1438, 1196, 1123, 1015, 958. **HRMS (ESI)** Cald for $C_{33}H_{36}O_4P$ [M-H]$^-$ 527.2357, found 527.2356.

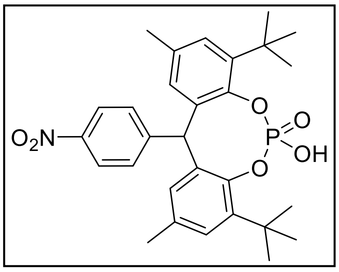

4,8-Di-*tert*-butyl-6-hydroxy-2,10-dimethyl-12-(4-nitrophenyl)-12*H* -dibenzo[d,g][1,3,2]dioxaphosphocine 6-oxide, PA8. Performed on a 5.4 mmol scale. Red solid (2.1 g, 74% yield). **M.p.** 199 – 202 °C. **^{1}H NMR (200 MHz, $CDCl_3$) δ** 10.83 (brs, 1H), 8.09 – 7.95 (m, 2H), 7.28 – 7.21 (m, 2H), 7.06 (s, 2H), 6.88 (d, *J* = 2.1 Hz, 2H), 6.02 (s, 1H), 2.22 (s, 6H), 1.32 (s, 18H). **^{13}C NMR (101 MHz, $CDCl_3$) δ** 149.27, 146.72, 146.63, 146.47, 141.80, 141.75, 134.91, 134.90, 133.20, 129.65, 128.20, 123.11, 35.16, 31.11, 21.30. **^{31}P NMR (162 MHz, $CDCl_3$) δ** −11.54**IR ATR (vmax/cm^{-1})** 2962, 1596, 1517, 1443, 1346, 898. **HRMS (ESI)** Cald for $C_{29}H_{33}NO_6P$ [M-H]$^-$ 522.2051, found 522.2052.

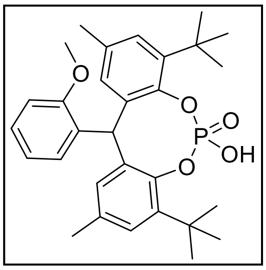

4,8-Di-*tert*-butyl-6-hydroxy-12-(2-methoxyphenyl)-2,10-dimethyl-12*H* -dibenzo[d,g][1,3,2]dioxaphosphocine 6-oxide, PA9. Performed on a 3.6 mmol scale. White solid (1.7 g, 95% yield). **M.p.** 174 – 176 °C. **^{1}H NMR (400 MHz, CD_2Cl_2) δ** 10.08 (brs, 1H), 7.64 (ddd, *J* = 7.8, 1.6, 0.8 Hz, 1H), 7.27 (td, *J* = 7.7, 1.6 Hz, 1H), 7.05 (s, 2H), 7.02 – 6.93 (m, 3H), 6.87 (dd, *J* = 8.2, 1.2 Hz, 1H), 6.47 (s, 1H), 3.52 (s, 3H), 2.25 (s, 6H), 1.42 (s, 18H). **^{13}C NMR (101 MHz, CD_2Cl_2) δ** 158.54, 147.36, 147.27, 141.03, 140.97, 134.70, 134.67, 134.30, 134.29, 129.75, 129.34, 128.36, 127.88, 127.86, 126.99, 120.27, 112.13, 56.08, 38.08, 35.25, 31.15, 21.33. **^{31}P NMR (162 MHz, CD_2Cl_2) δ** −10.56. **IR ATR (vmax/cm^{-1})** 2959, 1599, 1459, 1436, 1245, 1199, 1107, 955. **HRMS (ESI)** Cald for $C_{30}H_{36}O_5P$ $[M-H]^-$ 507.2306, found 507.2304.

4. Substrate synthesis

3-Methyl-2-cyclohexenone (**1a**), 3-methyl-2-cyclopentenone (**1b**) and 4,4-dimethyl-2-cyclohexenone (**1n**) have been purchased from commercial supplier (Sigma Aldrich). 3-Ethoxy-2-cyclohexenone (**27**) has been purchased from Acros Organics. All of these were used without further purifications.

1c-**m** were prepared *via* Grignard-reaction, as depicted in Scheme 4.

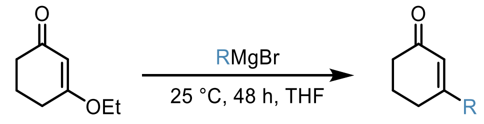

27 **1c-m**

General procedure:[22] A 3 M solution of the Grignard reagent was prepared freshly from freshly ground Mg (1.0 equiv.), alkyl/aryl bromide (1.0 equiv.) in dry Et_2O (2.0 M for alkyl halide, 1.0 M for aryl halide) and it was stirred for 1 h. After being cooled to 0 °C, 3-ethoxy-2-cyclohexenone (**27**, 1.0 equiv.) in dry THF (10 mL) was added slowly, and the reaction mixture was stirred for 18 h at room temperature. The reaction mixture was quenched with 1 N HCl solution (50 mL) at 0 °C. Et_2O was added and the organic phase was washed with 1 N HCl solution (3 × 25 mL), sat. $NaHCO_3$ (3 × 25 mL), dried over anhydrous Na_2SO_4 and concentrated under reduced pressure. The crude products were purified by column chromatography (PE: Et_2O, UV and vaniline TLC visualization) to provide the pure products.

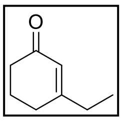

3-Ethyl-2-cyclohexenone, 1c.[22] Performed on a 14.0 mmol scale. Purified by column chromatography (25% Et_2O in PE). Yellow oil (1.20 g, 69% yield). **^{1}H NMR (400 MHz, $CDCl_3$) δ** 5.87 (s, 1H), 2.45 – 2.11 (m, 6H), 2.07 – 1.81 (m, 2H), 1.09 (t, *J* = 7.4 Hz, 3H).

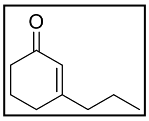

3-Propyl-2-cyclohexenone, 1d.[23] Performed on a 14.0 mmol scale. Purified by column chromatography (20% Et_2O in PE). Yellow oil (1.72 g, 89% yield). **^{1}H NMR (400 MHz, $CDCl_3$) δ** 5.84 (s, 1H), 2.36 – 2.31 (m, 2H), 2.25 (tdd, *J* = 6.2, 1.5, 0.7 Hz, 2H), 2.20 – 2.13 (m, 2H), 2.01 – 1.92 (m, 2H), 1.51 (h, *J* = 7.4 Hz, 2H), 0.91 (t, *J* = 7.4 Hz, 3H).

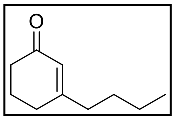

3-Butyl-2-cyclohexenone, 1e.[24] Performed on a 14.0 mmol scale. Purified by column chromatography (30% Et_2O in PE). Yellow oil (1.53 g, 74% yield). **^{1}H NMR (400 MHz, $CDCl_3$) δ** 5.86 (s, 1H), 2.48 – 2.09 (m, 6H), 2.06 – 1.79 (m, 2H), 1.61 – 1.15 (m, 4H), 0.91 (t, *J* = 7.0 Hz, 3H).

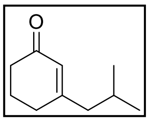

3-Isobutyl-2-cyclohexenone, 1f. [22] Performed on a 14.0 mmol scale. Purified by column chromatography (25% Et_2O in PE). Yellow oil (1.10 g, 69% yield). **^{1}H NMR (400 MHz, $CDCl_3$) δ** 5.84 (s, 1H), 2.54 – 2.17 (m, 4H), 2.18 – 1.56 (m, 5H), 0.90 (d, *J* = 6.5 Hz, 6H).

Supporting Information

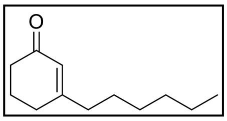

3-Hexyl-2-cyclohexenone, 1g.[25] Performed on a 14.0 mmol scale. Purified by column chromatography (30% Et_2O in PE). Yellow oil (1.96 g, 60% yield**).** **^{1}H NMR (400 MHz, $CDCl_3$)** δ 5.85 (p, *J* = 1.4 Hz, 1H), 2.33 (dd, *J* = 7.5, 6.0 Hz, 2H), 2.26 (td, *J* = 6.1, 1.5 Hz, 2H), 2.23 – 2.16 (m, 2H), 2.03 – 1.92 (m, 2H), 1.53 – 1.41 (m, 2H), 1.35 – 1.20 (m, 6H), 0.93 – 0.82 (m, 3H).

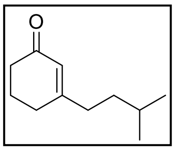

3-(3-Methylbutyl)-2-cyclohexen-1-one, 1h.[26] Performed on a 14.0 mmol scale. Purified by column chromatography (30% Et_2O in PE). Yellow oil (2.1 g, 89% yield). **^{1}H NMR (400 MHz, $CDCl_3$) δ** 5.85 (s, 1H), 2.33 (dd, *J* = 7.4, 6.0 Hz, 2H), 2.27 (ddt, *J* = 7.1, 5.5, 1.1 Hz, 2H), 2.22 – 2.16 (m, 2H), 2.00 – 1.91 (m, 2H), 1.54 (dp, *J* = 13.3, 6.7 Hz, 1H), 1.40 – 1.31 (m, 2H), 0.88 (d, *J* = 6.6 Hz, 6H).

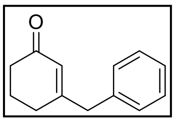

3-Benzylcyclohex-2-en-1-one, 1i.[27] Performed on a 18.0 mmol scale (1 M Grignard solution). Purified by column chromatography (35% Et_2O in PE). Yellow oil (1.8 g, 54% yield). **^{1}H NMR (400 MHz, $CDCl_3$) δ** 7.30 – 7.25 (m, 2H), 7.24 – 7.18 (m, 1H), 7.14 – 7.10 (m, 2H), 5.83 (s, 1H), 3.47 (s, 2H), 2.32 (dd, *J* = 7.5, 6.0 Hz, 2H), 2.25 – 2.17 (m, 2H), 1.98 – 1.86 (m, 2H).

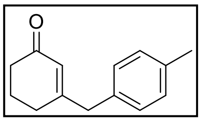

3-(4-Methylbenzyl)cyclohex-2-en-1-one, 1j.[28] Performed on a 14.0 mmol scale (1 M Grignard solution). Purified by column chromatography (30% Et_2O in PE). Yellow oil (0.91 g, 32% yield). **^{1}H NMR (400 MHz, $CDCl_3$) δ** 7.14 – 7.09 (m, 2H), 7.07 – 7.02 (m, 2H), 5.86 (s, 1H), 3.46 (s, 2H), 2.39 – 2.29 (m, 5H), 2.27 – 2.21 (m, 2H), 1.99 – 1.90 (m, 2H).

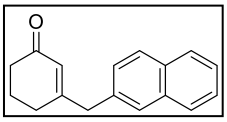

3-(Naphthalen-2-ylmethyl)cyclohex-2-en-1-one, 1k. Performed on a 18.0 mmol scale (1 M Grignard solution). Purified by column chromatography (35% Et_2O in PE). White solid (1.0 g, 24% yield). **M.p.** 75 – 77 °C. **^{1}H NMR (400 MHz, $CDCl_3$) δ** 7.64 – 7.54 (m, 3H), 7.44 – 7.39 (m, 1H), 7.31 – 7.22 (m, 2H), 7.13 – 7.02 (m, 1H), 5.73 (s, 1H), 3.46 (s, 2H), 2.15 (dd, *J* = 7.4, 6.0 Hz, 2H), 2.07 (td, *J* = 6.1, 1.5 Hz, 2H), 1.79 – 1.67 (m, 2H). **^{13}C NMR (101 MHz, $CDCl_3$) δ** 200.04, 164.65, 134.64, 132.54, 128.56, 127.84, 127.82, 127.66, 127.34, 127.24, 126.41, 125.92, 44.82, 37.46, 29.38, 22.80. **IR ATR (vmax/cm^{-1})** 3056, 1664, 811, 745. **HRMS (ESI)** Cald for $C_{17}H_{17}O$ $[M+H]^+$ 237.1274, Found 237.1228.

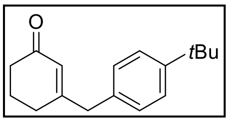

3-(4-(*Tert*-butyl)benzyl)cyclohex-2-en-1-one, 1l. Performed on a 14.0 mmol scale (1 M Grignard solution). Purified by column chromatography (30% Et_2O in PE). Yellow oil (1.7 g, 39% yield). **^{1}H NMR (400 MHz, $CDCl_3$) δ** 7.35 – 7.29 (m, 2H), 7.11 – 7.06 (m, 2H), 5.85 (s, 1H), 3.47 (s, 2H), 2.36 (dd, *J* = 7.4, 6.0 Hz, 2H), 2.30 – 2.22 (m, 2H), 2.02 – 1.92

(m, 2H), 1.31 (s, 9H). **^{13}C NMR (101 MHz, $CDCl_3$) δ** 200.15, 165.24, 149.92, 133.97, 128.92, 126.92, 125.73, 44.12, 37.46, 34.59, 31.50, 29.49, 22.80. **IR ATR (νmax/cm^{-1})** 2951, 1665, 812, 746. **HRMS (ESI)** Cald for $C_{17}H_{23}O$ $[M+H]^+$ 243.1744, Found 243.1749.

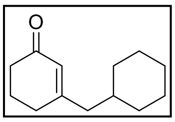

3-(Cyclohexylmethyl)cyclohex-2-en-1-one, 1m.[29] Performed on a 21.0 mmol scale. Purified by column chromatography (30% Et_2O in PE). Yellow oil (1.5 g, 71% yield). **^{1}H NMR (400 MHz, $CDCl_3$) δ** 5.83 (s, 1H), 2.37 – 2.31 (m, 2H), 2.25 (td, *J* = 6.1, 1.6 Hz, 2H), 2.08 (d, *J* = 7.2 Hz, 2H), 1.97 (dq, *J* = 7.7, 6.3 Hz, 2H), 1.74 – 1.59 (m, 5H), 1.52 (ddq, *J* = 11.0, 7.2, 3.6 Hz, 1H), 1.28 – 1.07 (m, 3H), 0.97 – 0.83 (m, 2H).

5. Parameter optimization for asymmetric epoxidations

5.1. Preliminary phosphoric acid screening

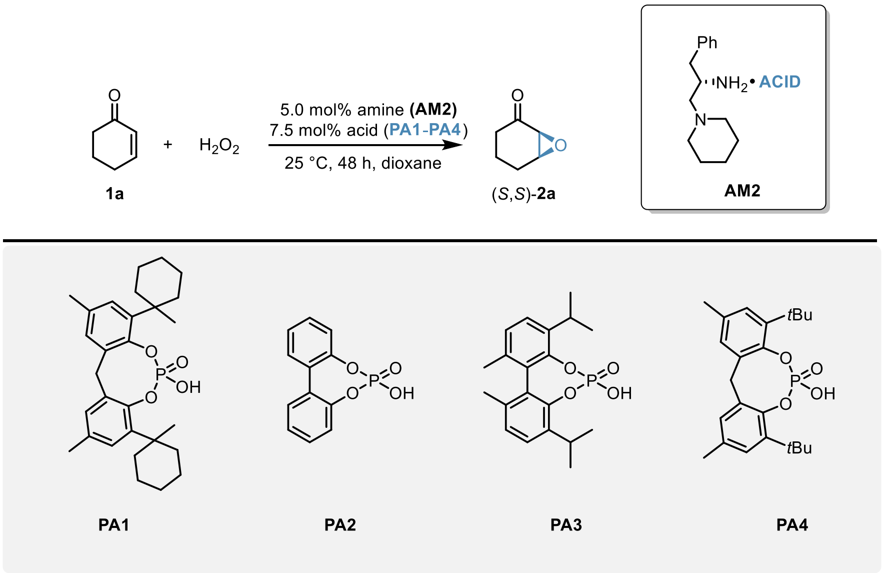

entry[a]	amine	acid	conv. [%][b]	ee [%][c]
1	AM2	**PA1**	**93**	**88**
2	AM2	**PA2**	7	25
3	AM2	**PA3**	93	79
4	AM2	**PA4**	60	88

[a] Performed with 0.30 mmol 2-cyclohexen-1-one (**1a**), 0.015 mmol catalyst (5 mol% **AM2**, 7.5 mol% **PA1**-**PA4**) and 1.5 equiv. H_2O_2 (50% aq.) in 1.25 mL dioxane at 25 °C for 48 hours. [b] Determined by GC analysis using *n*-dodecane as internal standard. [c] Determined by chiral GC analysis using a BGB175 chiral capillary column. Absolute configuration has been determined by measuring the optical rotation and comparing with literature data.

5.2 Amino-modification screening

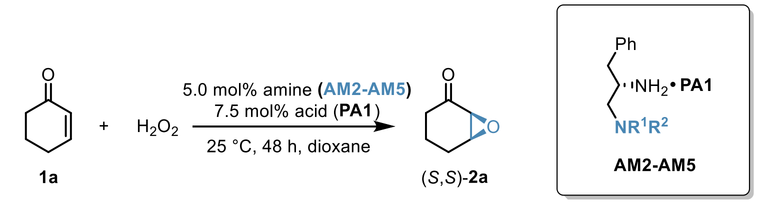

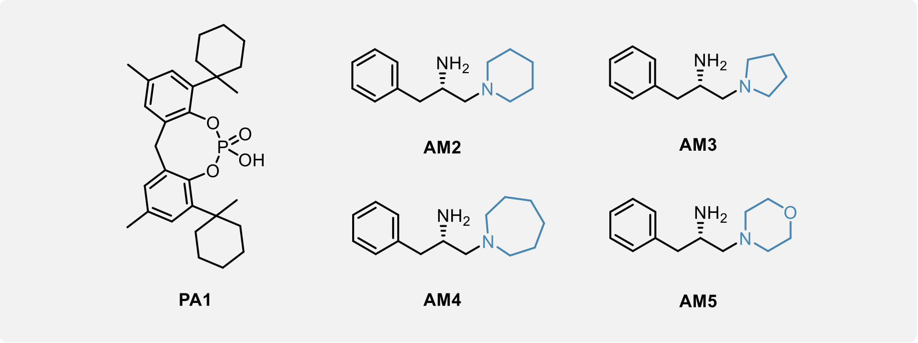

entry[a]	amine	acid	conv. [%][b]	ee [%][c]
1	**AM2**	**PA1**	**93**	**89**
2	**AM3**		78	84
3	**AM4**		65	86
4	**AM5**		20	84

[a] Performed with 0.30 mmol 2-cyclohexen-1-one (**1a**), 0.015 mmol catalyst (5 mol% **AM2**-**AM6**, 7.5 mol% **PA1**) and 1.5 equiv. H_2O_2 (50% aq.) in 1.25 mL dioxane at 25 °C for 48 hours. [b] Determined by GC analysis using *n*-dodecane as internal standard. [c] Determined by chiral GC analysis using a BGB175 chiral capillary column. Absolute configuration has been determined by measuring the optical rotation and comparing with literature data.

5.3 Amino acid screening

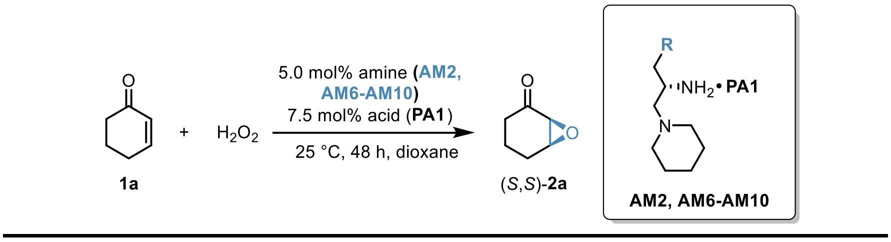

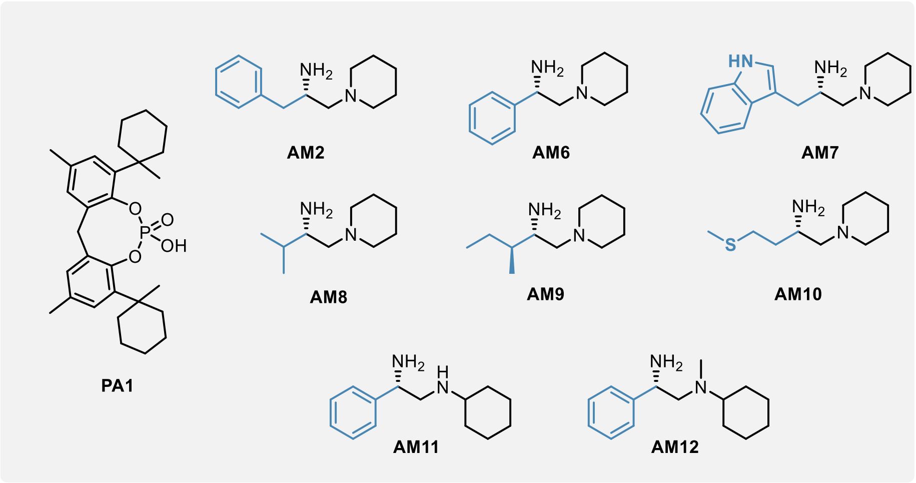

entry[a]	amine	acid	conv. [%][b]	ee [%][c]
1	**AM2**		93	89
2	**AM6**		**95**	**91**
3	**AM7**		67	81
4	**AM8**		94	81
5	**AM9**	**PA1**	81	82
6	**AM10**		77	62
7	**AM11**		22	91
8	**AM12**		34	80

[a] Performed with 0.30 mmol 2-cyclohexen-1-one (**1a**) 0.015 mmol catalyst (5 mol% **AM2**, **AM6**-**AM10**, 7.5 mol% **PA1**) and 1.5 equiv. H_2O_2 (50% aq.) in 1.25 mL dioxane at 25 °C for 48 hours. [b] Determined by GC analysis using *n*-dodecane as internal standard. [c] Determined by chiral GC analysis using a BGB175 chiral capillary column. Absolute configuration has been determined by measuring the optical rotation and comparing with literature data.

5.4 Solvent screening

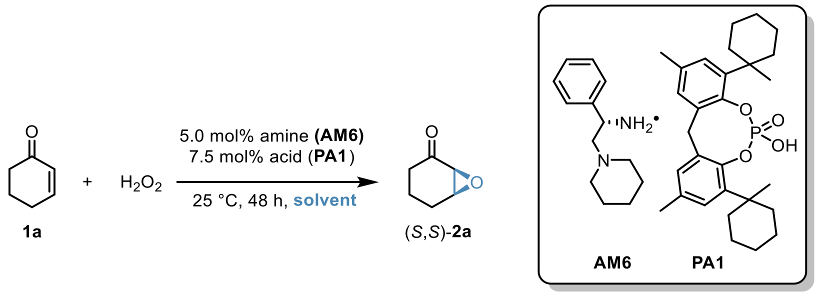

entry[a]	amine	acid	solvent	conv. [%][b]	ee [%][c]
**1**			**dioxane**	**95**	**91**
2			DME	73	87
3			2MeTHF	81	87
4	**AM6**	**PA1**	THF	18	87
5			MTBE	94	83
6			$CHCl_3$	42	84
7			toluene	42	60

[a] Performed with 0.30 mmol 2-cyclohexen-1-one (**1a**), 0.015 mmol catalyst (5 mol% **AM6**, 7.5 mol% **PA1**) and 1.5 equiv. H_2O_2 (50% aq.) in 1.25 mL solvent at 25 °C for 48 hours. [b] Determined by GC analysis using *n*-dodecane as internal standard. [c] Determined by chiral GC analysis using a BGB175 chiral capillary column. Absolute configuration has been determined by measuring the optical rotation and comparing with literature data.

5.5 Final phosphoric acid screening, screening of conventional acids

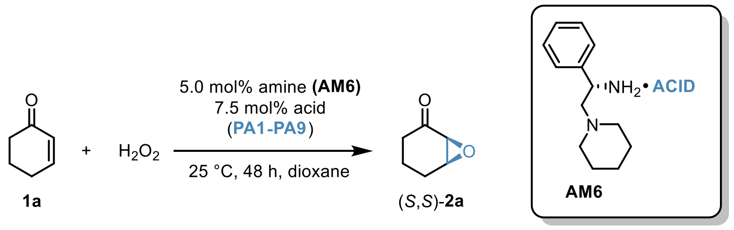

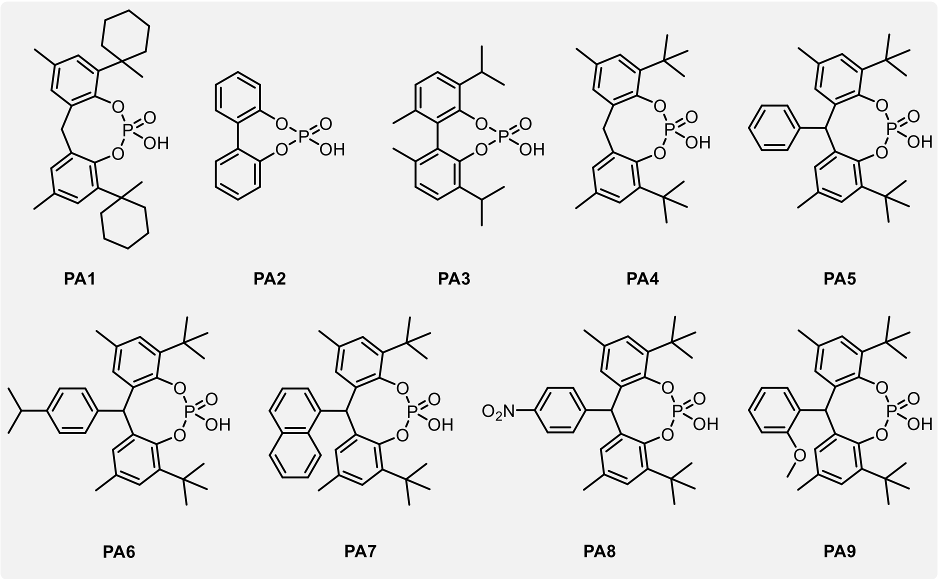

entry[a]	amine	acid	conv. [%][b]	ee [%][c]
1		no acid	0	n.d.
2		*p*TsOH	90	83 (91.5: 8.5)
3		2,5-dinitrobenzoic acid	94	67 (83.5: 16.5)
4		TFA	95	72 (86.0: 14.0)
5		Camphorsulfonic acid	87	82 (91.0: 9.0)
6		**PA1**	95	91 (95.5: 4.5)
7		**PA2**	12	69 (84.5: 15.5)
8		**PA3**	97	90 (95.0: 5.0)
9	**AM6**	**PA4**	85	92 (96.0: 4.0)
10		**PA5**	98	93 (96.5: 3.5)
11		**PA6**	96	94 (97.0: 3.0)
12		**PA7**	95	94 (97.0: 3.0)
13		**PA8**	95	93 (96.5: 3.5)
14		**PA9**	95	95 (97.5: 2.5)
15[d]		**PA9**	**96**	**95 (97.5: 2.5)**
16[e]		**PA9**	48	86 (93.0: 7.0)

[a] Performed with 0.30 mmol 2-cyclohexen-1-one (**1a**), 0.015 mmol catalyst (5 mol% **AM6**, 5.0-10.0 mol% **PA1-PA9** or other acid) and 1.5 equiv. H_2O_2 (50% aq.) in 1.5 mL dioxane at 25 °C for 48 hours. [b] Determined by GC analysis using *n*-dodecane as internal standard. [c] Determined by chiral GC analysis using a BGB175 chiral capillary column. Enantiomeric ratios in parenthesis. Absolute configuration has been determined by measuring the optical rotation and comparing with literature data. [d] Performed with an amine-to-acid ratio of 1/1 (5 mol% **AM6**, 5 mol% **PA9**). [e] Performed with an amine-to-acid ratio of 1/2 (5 mol% **AM6**, 10 mol% **PA9**).

6. General procedure and analytical data for the asymmetric epoxidations

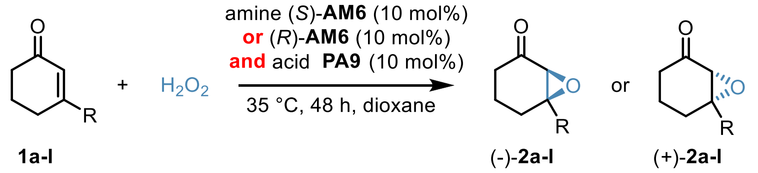

Reactions were performed on a 1.0 – 3.0 mmol scale. In a 20 mL screw cap vial the catalyst was prepared *in situ* by stirring the amine **AM6** (10 mol%) and phosphoric acid **PA9** (10 mol%) in anhydrous dioxane (0.18 M) for 20 min. Then the enone **1a-l** (1.0-3.0 mmol) was added and the reaction mixture was stirred for another 15 min at room temperature. Finally, 50% aqueous hydrogen peroxide (1.1 equiv.) was added and the resulting clear solution was stirred for 48 h at 35 °C. The reaction mixture was allowed to cool down to room temperature. Et_2O, distilled H_2O and brine were added, the aqueous phase was extracted with Et_2O (3×) and the combined organic phases were washed with brine and dried over Na_2SO_4, filtered and concentrated *in vacuo*. The crude products were purified by column chromatography (PE: Et_2O, vanillin staining agent) to provide the pure epoxyketones (−)-**2a**-**l** and (+)-**2a**-**l**.

For epoxides **2o-q** additional/modified workup is required: After 48 h at 35 °C, EtOAc was added and the reaction mixture was extracted with brine (2×) and concentrated *in vacuo.* The crude was taken up in small amounts of EtOAc, filtered through a silica filled pasteur pipette and eluted with EtOAc (5 mL). The solution was removed and approx. 3 mL dioxane and 1 mL Et_2O was added, followed by 1 equiv. 1N NaOH. After 30 min stirring the mixture was extracted with Brine (2×), concentrated and purified by column chromatography (PE:EE mixtures, vanillin staining agent) to provide the pure epoxyketones **2o-q**.

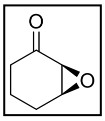

(1*S*,6*S*)-7-Oxabicyclo[4.1.0]heptan-2-one, **(*S*,*S*)-2a.**[27] Performed on a 3.0 mmol scale. Purified by column chromatography (25% Et_2O in PE, R_f = 0.2). Colourless liquid (201 mg, 60% yield, reduced yield due to high volatility). $[\alpha]_D^{20}$ = − 141.77 (c 1.0, CH_2Cl_2). **^{1}H NMR (400 MHz, $CDCl_3$) δ** 3.59 – 3.54 (m, 1H), 3.19 (d, *J* = 3.9 Hz, 1H), 2.56 – 2.47 (m, 1H), 2.24 (m, 1H), 2.11 – 1.99 (m, 1H), 1.98 – 1.83 (m, 2H), 1.70 – 1.60 (m, 1H). **^{13}C NMR (101 MHz, $CDCl_3$) δ** 206.04, 56.02, 55.20, 36.45, 22.94, 17.10. The **enantiomeric ratio** was determined to be 97.3:2.7 er (95% ee) by chiral GC analysis (BGB 175 column, 110 °C isothermal for 20 min, 30 °C/min to 220 °C, 25 min method), major enantiomer: t_r = 12.15 min, minor enantiomer: t_r = 13.43 min,).

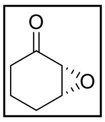

(1*R*,6*R*)-7-Oxabicyclo[4.1.0]heptan-2-one, **(*R*,*R*)-2a.**[27] Performed on a 2.5 mmol scale. Purified by column chromatography (25% Et_2O in PE, R_f = 0.2). Colourless liquid (185 mg, 65% yield, reduced yield due to high volatility). $[\alpha]_D^{20}$ = + 175.60 (c 1.0, CH_2Cl_2). The

enantiomeric ratio was determined to be 97.3:2.7 er (95% ee) by chiral GC analysis (BGB 175 column, 110 °C isothermal for 20 min, 30 °C/min to 220 °C, 25 min method), minor enantiomer: t_r = 12.49 min, major enantiomer: t_r = 13.29 min.

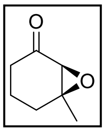

(1*S*,6*S*)-6-Methyl-7-oxabicyclo[4.1.0]heptan-2-one, (*S*,*S*)-2b.[27] Performed on a 3.0 mmol scale. Purified by column chromatography (25% Et_2O in PE, R_f = 0.33). Colourless liquid (246 mg, 65% yield, reduced yield due to high volatility). $[\alpha]_D^{20}$ = − 152.99 (c 1.0, CH_2Cl_2). **^{1}H NMR (400 MHz, CDCl$_3$) δ** 3.06 (s, 1H), 2.47 (dtd, *J* = 17.6, 4.1, 1.0 Hz, 1H), 2.16 – 1.79 (m, 4H), 1.68 – 1.58 (m, 1H), 1.43 (s, 3H). **^{13}C NMR (101 MHz, CDCl$_3$) δ** 206.83, 62.52, 62.09, 35.79, 28.50, 22.32, 17.28). The **enantiomeric ratio** was determined to be 97.4:2.6 er (95% ee) by chiral GC analysis (BGB 175 column, 110 °C isothermal for 20 min, 30 °C/min to 220 °C, 25 min method), minor enantiomer: t_r = 12.30 min, major enantiomer: t_r = 13.92 min.

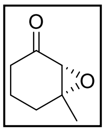

(1*R*,6*R*)-6-Methyl-7-oxabicyclo[4.1.0]heptan-2-one, (*R*,*R*)-2b.[27] Performed on a 3.0 mmol scale. Purified by column chromatography (25% Et_2O in PE, R_f = 0.33). Colourless liquid (244 mg, 65% yield, reduced yield due to high volatility). $[\alpha]_D^{20}$ = + 164.20 (c 1.0, CH_2Cl_2). The **enantiomeric ratio** was determined to be 97.5:2.5 er (95% ee) by chiral GC analysis (BGB 175 column, 110 °C isothermal for 20 min, 30 °C/min to 220 °C, 25 min method), major enantiomer: t_r = 12.15 min, minor enantiomer: t_r = 14.17 min.

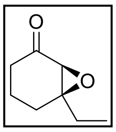

(1*S*,6*S*)-6-Ethyl-7-oxabicyclo[4.1.0]heptan-2-one, (*S*,*S*)-2c.[27] Performed on a 2.15 mmol scale. Purified by column chromatography (25% Et_2O in PE, R_f = 0.30). Colourless liquid (211 mg, 70% yield, reduced yield due to high volatility). $[\alpha]_D^{20}$ = − 141.97 (c 1.0, CH_2Cl_2). **^{1}H NMR (400 MHz, CDCl$_3$) δ** 3.07 (s, 1H), 2.53 – 2.45 (m, 1H), 2.14 – 1.81 (m, 4H), 1.78 – 1.60 (m, 3H), 0.96 (t, *J* = 7.5 Hz, 3H). **^{13}C NMR (101 MHz, CDCl$_3$) δ** 207.14, 66.24, 60.90, 36.09, 28.93, 26.28, 17.50, 8.79. The **enantiomeric ratio** was determined to be 97.1:2.9 er (94% ee) by chiral GC analysis (BGB 175 column, 110 °C isothermal for 20 min, 30 °C/min to 220 °C, 25 min method), major enantiomer: t_r = 16.3 min, minor enantiomer: t_r = 17.55 min.

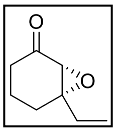

(1*R*,6*R*)-6-Ethyl-7-oxabicyclo[4.1.0]heptan-2-one, (*R*,*R*)-2c.[27] Performed on a 1.75 mmol scale. Purified by column chromatography (25% Et_2O in PE, R_f = 0.30). Colourless liquid (164 mg, 67% yield, reduced yield due to high volatility). $[\alpha]_D^{20}$ = + 139.87 (c 1.0, CH_2Cl_2). The **enantiomeric ratio** was determined to be 98.3:1.7 er (97% ee) by chiral GC analysis (BGB 175 column, 110 °C isothermal for 20 min, 30 °C/min to 220 °C, 25 min method), minor enantiomer: t_r = 16.44 min, major enantiomer: t_r = 17.09 min.

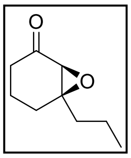

(1*S*,6*S*)-6-Propyl-7-oxabicyclo[4.1.0]heptan-2-one, **(*S*,*S*)-2d.**[30] Performed on a 2.15 mmol scale. Purified by column chromatography (20% Et_2O in PE, R_f = 0.31). Colourless liquid (237 mg, 71% yield, reduced yield due to high volatility). $[\boldsymbol{\alpha}]_{\boldsymbol{D}}^{\mathbf{20}}$ = – 117.34 (c 1.0, CH_2Cl_2). **^{1}H NMR (400 MHz, $CDCl_3$) δ** 3.06 (s, 1H), 2.49 (dt, *J* = 17.3, 4.6 Hz, 1H), 2.16 – 1.80 (m, 4H), 1.75 – 1.53 (m, 3H), 1.51 – 1.37 (m, 2H), 0.92 (td, *J* = 7.3, 1.4 Hz, 3H). **^{13}C NMR (101 MHz, $CDCl_3$) δ** 207.14, 65.43, 61.28, 38.15, 36.07, 26.46, 18.10, 17.47, 14.11. The **enantiomeric ratio** was determined to be 97.7:2.3 er (95% ee) by chiral HPLC analysis (Chiralpak® AS-H column, *n*-Hexane/*i*-PrOH = 90:10, 1 mL/min, λ = 210 nm, 25 °C), minor enantiomer: t_r = 7.47 min, major enantiomer: t_r = 11.6 min.

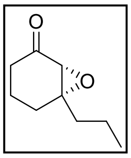

(1*R*,6*R*)-6-Propyl-7-oxabicyclo[4.1.0]heptan-2-one, **(*R*,*R*)-2d.**[30] Performed on a 2.15 mmol scale. Purified by column chromatography (20% Et_2O in PE, R_f = 0.31). Colourless liquid (244 mg, 75% yield, reduced yield due to high volatility). $[\boldsymbol{\alpha}]_{\boldsymbol{D}}^{\mathbf{20}}$ = + 136.06 (c 1.0, CH_2Cl_2). The **enantiomeric ratio** was determined to be 97.8:2.2 er (96% ee) by chiral HPLC analysis (Chiralpak® AS-H column, *n*-Hexane/*i*-PrOH = 90:10, 1 mL/min, λ = 210 nm, 25 °C), major enantiomer: t_r = 7.41 min, minor enantiomer: t_r = 11.85 min. $[\boldsymbol{\alpha}]_{\boldsymbol{D}}^{\mathbf{20}}$ = + 136.06 (c 1.0, CH_2Cl_2).

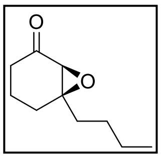

(1*S*,6*S*)-6-Butyl-7-oxabicyclo[4.1.0]heptan-2-one, **(*S*,*S*)-2e.**[27] Performed on a 2.0 mmol scale. Purified by column chromatography (25% Et_2O in PE, R_f = 0.28). Colourless liquid (242 mg, 72% yield, reduced yield due to high volatility). $[\boldsymbol{\alpha}]_{\boldsymbol{D}}^{\mathbf{20}}$ = – 126.95 (c 1.0, CH_2Cl_2). **^{1}H NMR (400 MHz, $CDCl_3$) δ** 3.05 (s, 1H), 2.53 – 2.44 (m, 1H), 2.14 – 1.80 (m, 4H), 1.76 – 1.51 (m, 3H), 1.43 – 1.26 (m, 4H), 0.89 (t, *J* = 7.1 Hz, 3H). **^{13}C NMR (101 MHz, $CDCl_3$) δ** 207.14, 65.56, 61.30, 36.05, 35.82, 26.88, 26.51, 22.71, 17.48, 14.02. The **enantiomeric ratio** was determined to be 98.6:1.4 er (97% ee) by chiral HPLC analysis (Chiralpak® IB column, *n*-Hexane/*i*-iPrOH = 98:2, 1 mL/min, λ = 210 nm, 25 °C), major enantiomer: t_r = 5.14 min, minor enantiomer: t_r = 5.53 min.

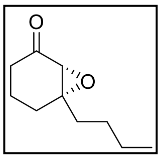

(1*R*,6*R*)-6-Butyl-7-oxabicyclo[4.1.0]heptan-2-one, **(*R*,*R*)-2e.**[27] Performed on a 1.0 mmol scale. Purified by column chromatography (25% Et_2O in PE, R_f = 0.28). Colourless liquid (114 mg, 68% yield, reduced yield due to high volatility). $[\boldsymbol{\alpha}]_{\boldsymbol{D}}^{\mathbf{20}}$ = + 111.66 (c 1.0, CH_2Cl_2). The **enantiomeric ratio** was determined to be 97.6:2.4 er (95% ee) by chiral HPLC analysis (Chiralpak® IB column, *n*-Hexane/*i*-PrOH = 98:2, 1 mL/min, λ = 210 nm, 25 °C), minor enantiomer: t_r = 5.17 min, major enantiomer: t_r = 5.57 min.

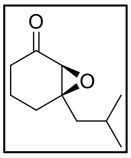

(1*S*,6*R*)-6-Isobutyl-7-oxabicyclo[4.1.0]heptan-2-one, (*S*,*R*)-2f.[27] Performed on a 1.5 mmol scale. Purified by column chromatography (15% Et_2O in PE, R_f = 0.27). Colourless liquid (130 mg, 51% yield, reduced yield due to high volatility). $[\alpha]_D^{20}$ = – 93.41 (c 1.0, CH_2Cl_2). **^{1}H NMR (400 MHz, $CDCl_3$) δ** 3.03 (s, 1H), 2.54 – 2.46 (m, 1H), 2.13 – 1.79 (m, 5H), 1.70 – 1.60 (m, 2H), 1.39 (dd, *J* = 14.1, 8.2 Hz, 1H), 0.96 (d, *J* = 6.6 Hz, 3H), 0.90 (d, *J* = 6.6 Hz, 3H). **^{13}C NMR (101 MHz, $CDCl_3$) δ** 207.20, 64.79, 61.66, 45.26, 36.07, 26.36, 25.19, 23.26, 22.66, 17.44. The **enantiomeric ratio** was determined to be 98.8:1.2 er (98% ee) by chiral HPLC analysis (Chiralpak® IB column, *n*-Hexane/*i*-PrOH = 98:2, 1 mL/min, λ = 210 nm, 25 °C), major enantiomer: t_r = 5.14 min, minor enantiomer: t_r = 5.54 min).

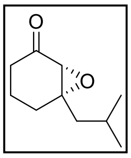

(1*R*,6*S*)-6-Isobutyl-7-oxabicyclo[4.1.0]heptan-2-one, (*R*,*S*)-2f.[27] Performed on a 2.0 mmol scale. Purified by column chromatography (15% Et_2O in PE, R_f = 0.27). Colourless liquid (181 mg, 55% yield, reduced yield due to high volatility). $[\alpha]_D^{20}$ = + 123.84 (c 1.0, CH_2Cl_2). The **enantiomeric ratio** was determined to be 98.8:1.2 er (98% ee) by chiral HPLC analysis (Chiralpak® IB column, *n*-Hexane/*i*-PrOH = 98:2, 1 mL/min, λ = 210 nm, 25 °C), minor enantiomer: t_r = 5.14 min, major enantiomer: t_r = 5.52 min.

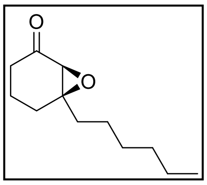

(1*S*,6*S*)-6-Hexyl-7-oxabicyclo[4.1.0]heptan-2-one, (*S*,*S*)-2g.[27] Performed on a 1.8 mmol scale. Purified by column chromatography (25% Et_2O in PE, R_f = 0.45). Colourless liquid (258 mg, 79% yield). $[\alpha]_D^{20}$ = – 96.77 (c 1.0, CH_2Cl_2). **^{1}H NMR (400 MHz, $CDCl_3$) δ** 3.06 (s, 1H), 2.54 – 2.45 (m, 1H), 2.15 – 1.80 (m, 4H), 1.74 – 1.55 (m, 3H), 1.40 (ttd, *J* = 8.5, 6.2, 1.6 Hz, 2H), 1.34 – 1.21 (m, 6H), 0.91 – 0.84 (m, 3H). **^{13}C NMR (101 MHz, $CDCl_3$) δ** 207.19, 65.62, 61.34, 36.15, 36.09, 31.77, 29.28, 26.54, 24.73, 22.63, 17.52, 14.15. The **enantiomeric ratio** was determined to be 98.6:1.4 er (97% ee) by chiral HPLC analysis (Chiralpak® IB column, *n*-Hexane/*i*-PrOH = 98:2, 1 mL/min, λ = 210 nm, 25 °C), major enantiomer: t_r = 4.83 min, minor enantiomer: t_r = 5.37 min.

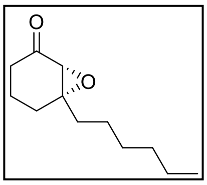

(1*R*,6*R*)-6-Hexyl-7-oxabicyclo[4.1.0]heptan-2-one, (*R*,*R*)-2g.[27] Performed on a 1.8 mmol scale. Purified by column chromatography (25% Et_2O in PE, R_f = 0.45). Colourless liquid (250 mg, 77% yield). $[\alpha]_D^{20}$ = + 83.40 (c 1.0, CH_2Cl_2). The **enantiomeric ratio** was determined to be 98.3:1.7 er (97% ee) by chiral HPLC analysis (Chiralpak® IB column, *n*-Hexane/*i*-PrOH = 98:2, 1 mL/min, λ = 210 nm, 25 °C), minor enantiomer: t_r = 4.89 min, major enantiomer: t_r = 5.47 min.

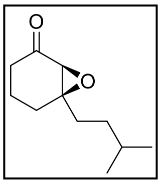

(1*S*,6*R*)-6-Isopentyl-7-oxabicyclo[4.1.0]heptan-2-one, **(*S*,*R*)-2h.** Performed on a 1.8 mmol scale. Purified by column chromatography (10% Et_2O in PE, R_f = 0.36). Colourless liquid (236 mg, 73% yield). $[\alpha]_D^{20}$ = – 106.04 (c 1.0, CH_2Cl_2). **^{1}H NMR (400 MHz, $CDCl_3$) δ** 3.06 (s, 1H), 2.54 – 2.44 (m, 1H), 2.15 – 1.80 (m, 4H), 1.74 – 1.47 (m, 4H), 1.32 – 1.23 (m, 2H), 0.88 (dd, *J* = 6.6, 1.9 Hz, 6H). **^{13}C NMR (101 MHz, $CDCl_3$) δ** 207.16, 65.78, 61.32, 36.07, 34.03, 33.66, 28.10, 26.61, 22.54, 17.54. The **enantiomeric ratio** was determined to be 99:1 er (98% ee) by chiral HPLC analysis (Chiralpak® IB column, *n*-Hexane/*i*-PrOH = 98:2, 1 mL/min, λ = 210 nm, 25 °C), major enantiomer: t_r = 4.85 min, minor enantiomer: t_r = 5.21 min. **HRMS** (ESI) Cald for $C_{11}H_{19}O_2$ $[M+H]^+$ 183.1380, Found 183.1329.

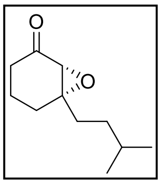

(1*R*,6*S*)-6-Isopentyl-7-oxabicyclo[4.1.0]heptan-2-one, **(*R*,*S*)-2h.** Performed on a 1.5 mmol scale. Purified by column chromatography (10% Et_2O in PE, R_f = 0.36). Colourless liquid (194 mg, 71% yield). $[\alpha]_D^{20}$ = + 106.85 (c 1.0, CH_2Cl_2). The **enantiomeric ratio** was determined to be 99:1 er (98% ee) by chiral HPLC analysis (Chiralpak® IB column, *n*-Hexane/*i*-PrOH = 98:2, 1 mL/min, λ = 210 nm, 25 °C), minor enantiomer: t_r = 4.85 min, major enantiomer: t_r = 5.20 min.

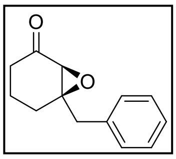

(1*S*,6*R*)-6-Benzyl-7-oxabicyclo[4.1.0]heptan-2-one, **(*S*,*R*)-2i.**[27] Performed on a 1.6 mmol scale. Purified by column chromatography (35% Et_2O in PE, R_f = 0.25). Colourless liquid (243 mg, 75% yield). $[\alpha]_D^{20}$ = – 103.00 (c 1.0, CH_2Cl_2). **^{1}H NMR (400 MHz, $CDCl_3$) δ** 7.30 – 7.11 (m, 5H), 3.06 (s, 1H), 2.93 (d, *J* = 1.5 Hz, 2H), 2.49 – 2.38 (m, 1H), 2.08 – 1.73 (m, 4H), 1.62 – 1.50 (m, 1H). **^{13}C NMR (101 MHz, $CDCl_3$) δ** 206.69, 135.58, 129.71, 128.70, 127.18, 65.42, 60.65, 42.38, 36.07, 26.45, 17.39. The **enantiomeric ratio** was determined to be 99:1 er (98% ee) by chiral HPLC analysis (Chiralpak® IB column, *n*-Hexane/*i*-PrOH = 98:2, 1 mL/min, λ = 210 nm, 25 °C), major enantiomer: t_r = 8.97 min, minor enantiomer: t_r = 10.1 min.

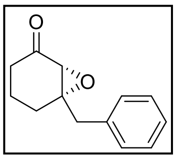

(1*R*,6*S*)-6-Benzyl-7-oxabicyclo[4.1.0]heptan-2-one, **(*R*,*S*)-2i.**[27] Performed on a 1.6 mmol scale. Purified by column chromatography (35% Et_2O in PE, R_f = 0.25). Colourless liquid (252 mg, 77% yield). $[\alpha]_D^{20}$ = + 107.82 (c 1.0, CH_2Cl_2). The **enantiomeric ratio** was determined to be 98.2:1.8 er (96% ee) by Chiral HPLC analysis (Chiralpak® IB column, *n*-Hexane/*i*-PrOH = 98:2, 1 mL/min, λ = 210 nm, 25 °C), major enantiomer: t_r = 9.10 min, minor enantiomer: t_r = 10.02 min.

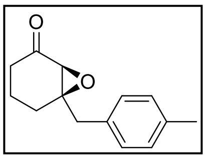

(1*S*,6*R*)-6-(4-Methylbenzyl)-7-oxabicyclo[4.1.0]heptan-2-one, (*S*,*R*)-2j. Performed on a 1.1 mmol scale. Purified by column chromatography (20% Et_2O in PE, R_f = 0.29). White solid (186 mg, 78% yield). **M.p. =** 54 – 56 °C. $[\alpha]_D^{20}$ = – 107.96 (c 1.0, CH_2Cl_2). **^{1}H NMR (400 MHz, $CDCl_3$) δ** 7.15 – 7.07 (m, 4H), 3.11 (s, 1H), 2.99 – 2.89 (m, 2H), 2.54 – 2.44 (m, 1H), 2.33 (s, 3H), 2.13 – 1.76 (m, 4H), 1.67 – 1.56 (m, 1H). **^{13}C NMR (101 MHz, $CDCl_3$) δ** 206.78, 136.78, 132.47, 129.58, 129.38, 65.55, 60.68, 41.97, 36.08, 26.43, 21.18, 17.41. The **enantiomeric ratio** was determined to be 99:1 er (98% ee) by chiral HPLC (Chiralpak® IB column, *n*-Hexane/*i*-PrOH = 98:2, 1 mL/min, λ = 210 nm, 25 °C), major enantiomer: t_r = 8.16 min, minor enantiomer: t_r = 8.98 min. **IR ATR (vmax/cm^{-1})** 2951, 1705, 817, 781. **HRMS (ESI)** Cald for $C_{14}H_{17}O_2$ $[M+H]^+$ 217.1223, Found 217.1208.

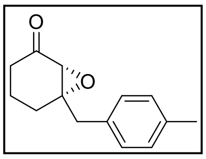

(1*R*,6*S*)-6-(4-Methylbenzyl)-7-oxabicyclo[4.1.0]heptan-2-one, (*R*,*S*)-2j. Performed on a 1.18 mmol scale. Purified by column chromatography (20% Et_2O in PE, R_f = 0.29). White solid (204 mg, 80% yield). $[\alpha]_D^{20}$ = + 93.69 (c 1.0, CH_2Cl_2). The **enantiomeric ratio** was determined to be 98.4:1.6 er (97% ee) by chiral HPLC (Chiralpak® IB column, *n*-Hexane/*i*-PrOH = 98:2, 1 mL/min, λ = 210 nm, 25 °C), minor enantiomer: t_r = 8.19 min, major enantiomer: t_r = 8.90 min.

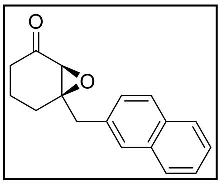

(1*S*,6*R*)-6-(Naphthalen-2-ylmethyl)-7-oxabicyclo[4.1.0]heptan-2-one, (*S*,*R*)-2k. Performed on a 0.9 mmol scale. Purified by column chromatography (15% Et_2O in PE, R_f = 0.14). Colourless liquid (158 mg, 69% yield). $[\alpha]_D^{20}$ = – 87.52 (c 1.0, CH_2Cl_2). **^{1}H NMR (400 MHz, $CDCl_3$) δ** 7.86 – 7.78 (m, 3H), 7.69 – 7.65 (m, 1H), 7.52 – 7.44 (m, 2H), 7.35 (dd, *J* = 8.4, 1.8 Hz, 1H), 3.18 (s, 1H), 3.14 (s, 2H), 2.55 – 2.45 (m, 1H), 2.17 – 1.80 (m, 4H), 1.60 (ddt, *J* = 12.6, 6.0, 4.1 Hz, 1H). **^{13}C NMR (101 MHz, $CDCl_3$) δ** 206.56, 133.50, 133.13, 132.56, 128.34 (d, *J* = 2.9 Hz), 127.76, 127.68, 126.32, 125.90, 65.43, 60.66, 42.49, 36.03, 26.43, 17.33. The **enantiomeric ratio** was determined to be 98.8:1.2 er (97% ee) by chiral HPLC (Chiralpak® OJ column, *n*-Hexane/*i*-PrOH = 85:15, 1 mL/min, λ = 210 nm, 25 °C), major enantiomer: t_r = 28.97 min, minor enantiomer: t_r = 35.43 min. **IR ATR (vmax/cm^{-1})** 3053, 2944, 1707, 811. **HRMS (ESI)** Cald for $C_{17}H_{17}O_2$ $[M+H]^+$ 253.1223, Found 253.1208.

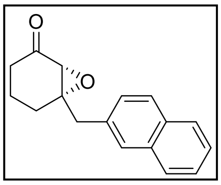

(1*R*,6*S*)-6-(Naphthalen-2-ylmethyl)-7-oxabicyclo[4.1.0]heptan-2-one, (*R*,*S*)-2k. Performed on a 0.9 mmol scale. Purified by column chromatography (15% Et_2O in PE, R_f = 0.14). Colourless liquid (162 mg, 71% yield). $[\alpha]_D^{20}$ = + 87.73 (c 1.0, CH_2Cl_2). The **enantiomeric ratio** was determined to be 98.2:1.8 er (96% ee) by chiral HPLC (Chiralpak® OJ column, *n*-Hexane/*i*-PrOH = 85:15, 1 mL/min, λ = 210 nm, 25 °C), minor enantiomer: t_r = 28.86 min, major enantiomer: t_r = 34.91 min.

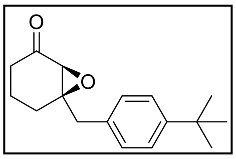

(1*S*,6*R*)-6-(4-(Tert-butyl)benzyl)-7-oxabicyclo[4.1.0]heptan-2-one, (*S*,*R*)-2l. Performed on a 1.22 mmol scale. Purified by column chromatography (20% Et_2O in PE, R_f = 0.28). White solid (227 mg, 72% yield). **M.p. =** 46 – 48 °C. $[\alpha]_D^{20}$ = –90.48 (c 1.0, CH_2Cl_2). **^{1}H NMR (400 MHz, CDCl$_3$) δ** 7.32 (d, *J* = 8.3 Hz, 2H), 7.13 (d, *J* = 8.5 Hz, 2H), 3.10 (s, 1H), 3.00 – 2.87 (m, 2H), 2.54 – 2.42 (m, 1H), 2.13 – 1.79 (m, 4H), 1.69 – 1.57 (m, 1H), 1.31 (s, 9H). **^{13}C NMR (101 MHz, CDCl$_3$) δ** 206.89, 150.07, 132.43, 129.39, 125.62, 65.55, 60.66, 41.84, 36.10, 34.60, 31.49, 26.54, 17.42. The **enantiomeric ratio** was determined to be 99:1 er (98% ee) by chiral HPLC (Chiralpak® IB column, *n*-Hexane/*i*-PrOH = 98:2, 1 mL/min, λ = 210 nm, 25 °C), major enantiomer: t_r = 6.15 min, minor enantiomer: t_r = 6.62 min **IR ATR (vmax/cm^{-1})** 2945, 1708, 836. **HRMS (ESI)** Cald for $C_{17}H_{23}O_2$ $[M+H]^+$ 259.1693, Found 259.1682.

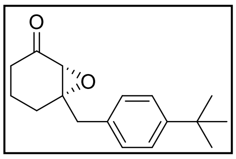

(1*R*,6*S*)-6-(4-(Tert-butyl)benzyl)-7-oxabicyclo[4.1.0]heptan-2-one, (*R*,*S*)-2l. Performed on a 1.05 mmol scale. Purified by column chromatography (25% Et_2O in PE, R_f = 0.33). White solid (194 mg, 72% yield). $[\alpha]_D^{20}$ = + 80.41 (c 1.0, CH_2Cl_2). The **enantiomeric ratio** was determined to be 98.5:1.5 er (97% ee) by chiral HPLC (Chiralpak® IB column, *n*-Hexane/*i*-PrOH = 98:2, 1 mL/min, λ = 210 nm, 25 °C), minor enantiomer: t_r = 6.25 min, major enantiomer: t_r = 6.79 min.

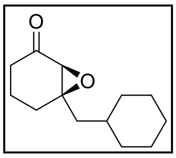

(1*S*,6*R*)-6-(Cyclohexylmethyl)-7-oxabicyclo[4.1.0]heptan-2-one, (*S*,*R*)-2m. Performed on a 1.57 mmol scale. Purified by column chromatography (25% Et_2O in PE, Rf = 0.31). Colourless liquid (219 mg, 67% yield). $[\alpha]_D^{20}$ = – 100.68 (c 1.0, CH_2Cl_2). **^{1}H NMR (400 MHz, CDCl$_3$) δ** 3.03 (s, 1H), 2.54 – 2.45 (m, 1H), 2.13 – 1.82 (m, 4H), 1.77 – 1.59 (m, 7H), 1.58 – 1.44 (m, 1H), 1.39 (dd, *J* = 13.7, 8.0 Hz, 1H), 1.31 – 1.06 (m, 3H), 1.02 – 0.84 (m, 2H). **^{13}C NMR (101 MHz, CDCl$_3$) δ** 207.19, 64.41, 61.60, 43.86, 35.94, 34.44, 33.82, 33.23, 26.38, 26.26, 26.16, 26.13, 17.31. The **enantiomeric ratio** was determined to be 98.8:1.2 er (98% ee) by chiral HPLC (Chiralpak® IB column, *n*-Hexane/*i*-PrOH = 99:1, 1 mL/min, λ = 220 nm, 25 °C), major enantiomer: t_r = 5.84 min, minor enantiomer: t_r = 6.58 min. **HRMS (ESI)** Cald for $C_{13}H_{21}O_2$ $[M+H]^+$ 209.1536, Found 209.1507.

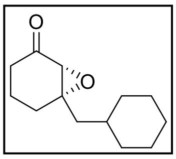

(1*R*,6*S*)-6-(Cyclohexylmethyl)-7-oxabicyclo[4.1.0]heptan-2-one, (*R*,*S*)-2m. Performed on a 1.57 mmol scale. Purified by column chromatography (25% Et_2O in PE, R_f = 0.31). Colourless liquid (230 mg, 70% yield). $[\alpha]_D^{20}$ = + 106.21 (c 1.0, CH_2Cl_2). The **enantiomeric ratio** was determined to be 98.8:1.2 er (98% ee) by chiral HPLC (Chiralpak® IB column, *n*-Hexane/*i*-PrOH = 99:1, 1 mL/min, λ = 220 nm, 25 °C), minor enantiomer: t_r = 5.76 min, major enantiomer: t_r = 6.43 min.

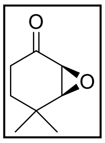

(1*S*,6*S*)-5,5-Dimethyl-7-oxabicyclo[4.1.0]heptan-2-one, (*S*,*S*)-2n.[27] Performed on a 2.83 mmol scale. Purified by column chromatography (20% Et_2O in PE, R_f = 0.29). Colourless liquid (258 mg, 65% yield). $[\alpha]_D^{20}$ = – 148.55 (c 1.0, CH_2Cl_2). **^{1}H NMR (400 MHz, $CDCl_3$) δ** 3.21 (d, *J* = 4.0 Hz, 1H), 3.16 (dd, *J* = 4.0, 1.3 Hz, 1H), 2.39 (ddd, *J* = 18.9, 6.4, 3.0 Hz, 1H), 2.18 (ddd, *J* = 18.8, 11.7, 7.0 Hz, 1H), 1.95 – 1.84 (m, 1H), 1.33 (dddd, *J* = 13.7, 7.0, 3.0, 1.3 Hz, 1H), 1.21 (s, 3H), 1.05 (s, 3H). **^{13}C NMR (101 MHz, $CDCl_3$) δ** 206.15, 64.28, 56.07, 33.29, 30.90, 29.93, 27.56, 23.02. The **enantiomeric ratio** was determined to be 97.2:2.8 er (94% ee) by chiral GC (BGB 175 column, 110 °C isothermal for 20 min, 30 °C/min to 220 °C, 25 min method), major enantiomer: t_r = 13.42 min, minor enantiomer: t_r = 15.45 min.

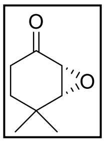

(1*R*,6*R*)-5,5-Dimethyl-7-oxabicyclo[4.1.0]heptan-2-one, (*R*,*R*)-2n.[27] Performed on a 2.41 mmol scale. Purified by column chromatography (20% Et_2O in PE, R_f = 0.29). Colourless liquid (213 mg, 63% yield). $[\alpha]_D^{20}$ = + 156.13 (c 1.0, CH_2Cl_2). The **enantiomeric ratio** was determined to be 96.7:3.3 er (93% ee) by chiral GC (BGB 175 column, 110 °C isothermal for 20 min, 30 °C/min to 220 °C, 25 min method), major enantiomer: t_r = 13.69 min, minor enantiomer: t_r = 15.00 min.

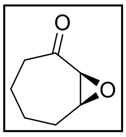

(1*S*,7*S*)-8-Oxabicyclo[5.1.0]octan-2-one, (*S*,*S*)-2o.[27] Performed on a 0.91 mmol scale. Purified by column chromatography (3% EE in PE, R_f = 0.4). Colourless liquid (62 mg, 54% yield). $[\alpha]_D^{20}$ = – 15.03 (c 0.66, CH_2Cl_2). **^{1}H NMR (400 MHz, CD_2Cl_2) δ** 3.31 – 3.25 (m, 2H), 2.57 – 2.50 (m, 1H), 2.21 – 2.16 (m, 1H), 1.78 – 1.56 (m, 4H), 0.95 – 0.87 (m, 1H). **^{13}C NMR (101 MHz, CD_2Cl_2) δ** 210.46, 59.40, 55.08, 40.52, 27.41, 23.52, 22.95. The **enantiomeric ratio** was determined to be 98:2 er (96% ee) by chiral GC (BGB 175 column, 110 °C isothermal for 30 min, 30 °C/min to 220 °C, 38 min method), major enantiomer: t_r = 15.80 min, minor enantiomer: t_r = 16.78 min.

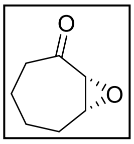

(1*R*,7*R*)-8-Oxabicyclo[5.1.0]octan-2-one, (*R*,*R*)-2o.[27] Performed on a 0.91 mmol scale. Purified by column chromatography (3% EE in PE, R_f = 0.4). Colourless liquid (64 mg, 56% yield). $[\alpha]_D^{20}$ = + 17.07 (c 0.71, CH_2Cl_2). The **enantiomeric ratio** was determined to be 97.5:2.5 er (95% ee) by chiral GC (BGB 175 column, 110 °C isothermal for 30 min, 30 °C/min to 220 °C, 38 min method), minor enantiomer: t_r = 16.00 min, major enantiomer: t_r = 16.47 min.

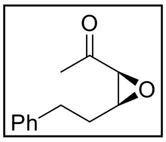

1-((2S,3S)-3-phenethyloxiran-2-yl)ethan-1-one, (*S*,*S*)-2p.[27] Performed on a 0.57 mmol scale. Purified by column chromatography (5% EE in PE, R_f = 0.45). Colourless liquid (65 mg, 59% yield). $[\alpha]_D^{20}$ = – 13.34 (c 0.62, CH_2Cl_2). **^{1}H NMR (400 MHz, $CDCl_3$) δ** 7.25 – 7.23 (m, 2H), 7.16 – 7.10 (m, 3H), 3.09 (d, *J* = 4.0 Hz, 1H), 3.03 – 3.01 (m, 1H), 2.74 – 2.67 (m, 2H), 1.93 (s, 3H), 1.90 – 1.85 (m, 2H). **^{13}C NMR (101 MHz, $CDCl_3$) δ** 205.63, 140.42, 128.64, 128.35, 126.35, 59.93,

57.51, 33.47, 32.01, 24.55. The **enantiomeric ratio** was determined to be 92.5:7.5 er (85% ee) by chiral GC (BGB 173 column, 150 °C isothermal for 40 min, 30 °C/min to 220 °C, 48 min method), major enantiomer: t_r = 13.92 min, minor enantiomer: t_r = 14.46 min.

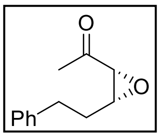

1-((2R,3R)-3-phenethyloxiran-2-yl)ethan-1-one, (*R*,*R*)-2p.[27] Performed on a 0.57 mmol scale. Purified by column chromatography (5% EE in PE, R_f = 0.45). Colourless liquid (60 mg, 55% yield). $[\alpha]_D^{20}$ = + 15.32 (c 0.62, CH_2Cl_2). The **enantiomeric ratio** was determined to be 92:8 er (84% ee) by chiral GC (BGB 173 column, 150 °C isothermal for 40 min, 30 °C/min to 220 °C, 48 min method), minor enantiomer: t_r = 13.91 min, major enantiomer: t_r = 14.46 min.

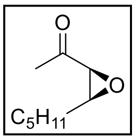

(2*S*,3*S*)-3-Hexyloxiran-2-yl)ethan-1-one, (*S*,*S*)-2q.[27] Performed on a 0.71 mmol scale. Purified by column chromatography (3% EE in PE, R_f = 0.3). Colourless liquid (62 mg, 56% yield). $[\alpha]_D^{20}$ = – 33.78 (c 0.71, CH_2Cl_2). **^{1}H NMR (400 MHz, $CDCl_3$) δ** 3.10 (d, *J* = 4.0 Hz, 1H), 3.02 – 2.99 (m, 1H), 1.99 (s, 3H), 1.57 – 1.56 (m, 2H), 1.43 – 1.37 (m, 2H), 1.27 – 1.25 (m, 4H), 0.83 (t, *J* = 6.0 Hz, 3H). **^{13}C NMR (101 MHz, $CDCl_3$) δ** 206.610, 59.95, 58.09, 31.74, 31.40, 25.45, 24.34, 22.48, 13.92. The **enantiomeric ratio** was determined to be 94.5:5.5 er (89% ee) by chiral GC (BGB 175 column, 110 °C isothermal for 30 min, 30 °C/min to 220 °C, 38 min method), minor enantiomer: t_r = 14.14 min, major enantiomer: t_r = 14.90 min.

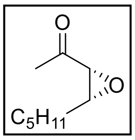

(2*R*,3*R*)-3-Benzyloxiran-2-yl)ethan-1-one, (*R*,*R*)-2q.[27] Performed on a 0.71 mmol scale. Purified by column chromatography (3% EE in PE, R_f = 0.3). Colourless liquid (63 mg, 57% yield). $[\alpha]_D^{20}$ = 42.09 (c 0.66, CH_2Cl_2). The **enantiomeric ratio** was determined to be 93.5:6.5 er (87% ee) by chiral GC (BGB 175 column, 110 °C isothermal for 30 min, 30 °C/min to 220 °C, 38 min method), major enantiomer: t_r = 14.09 min, minor enantiomer: t_r = 15.00 min.

7. Parameter optimization for the asymmetric aziridinations

7.1 Solvent screening

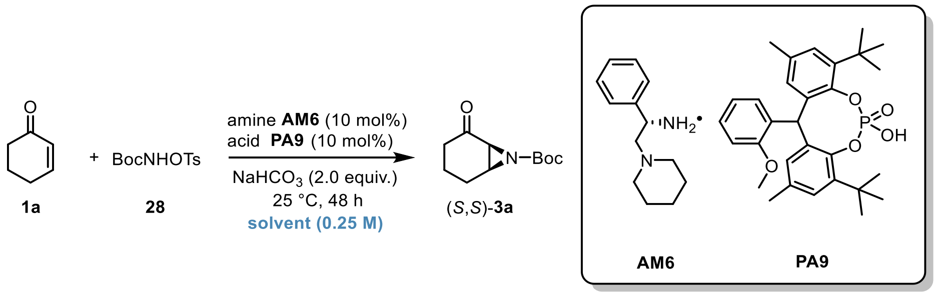

entry[a]	amine	acid	solvent	conv. [%][b]	ee [%][c]
1			dioxane	11	n.d.
2	AM6	PA9	MTBE	7	n.d.
3			DCM	99	64
4			$CHCl_3$	98	98

[a] Performed with 0.30 mmol 2-cyclohexen-1-one (**1a**, 1.2 equiv.), 0.25 mmol **28** (1.0 equiv.), 0.025 mmol catalyst (10 mol% **AM6**, 10 mol% **PA9**) and 0.60 mmol (2.0 equiv.) $NaHCO_3$ in 1.0 mL solvent at 25 °C for 48 hours. [b] Determined by GC-MS analysis. [c] Determined by chiral HPLC analysis using a Chiralpak® AS-H column. Absolute configuration has been determined by measuring the optical rotation and comparing with literature data.

7.2 Base screening

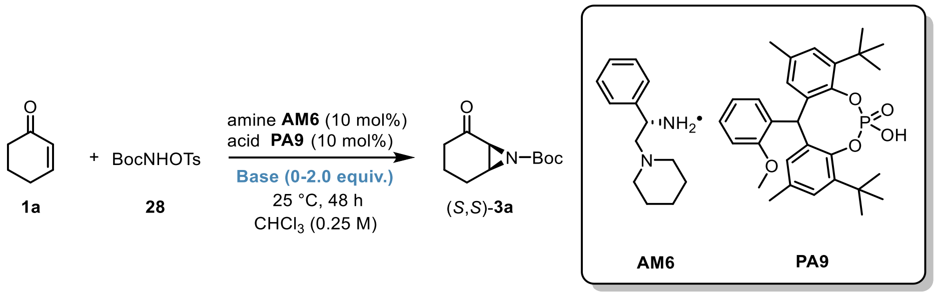

entry[a]	amine	acid	base	conv. [%][b]	ee [%][c]
1			$NaHCO_3$ (0 equiv.)	traces	n.d.
2			$NaHCO_3$ (1.0 equiv.)	36	57
3			$NaHCO_3$ (2.0 equiv.)	98	98
4			Na_2CO_3 (2.0 equiv.)	25	n.d.
5	**AM6**	**PA9**	*i*Pr_2NEt (2.0 equiv.)	traces	n.d.
6			Pyridine (2.0 equiv.)	23	68
7			Lutidine (2.0 equiv.)	traces	n.d
8			Cs_2CO_3 (2.0 equiv.)	5	n.d.
**9**			**NaOAc (2.0 equiv.)**	**98**	**99.5**

[a] Performed with 0.30 mmol 2-cyclohexen-1-one (**1a**, 1.2 equiv.), 0.25 mmol **28** (1.0 equiv.), 0.025 mmol catalyst (10 mol% **AM6**, 10 mol% **PA9**) and 0-0.60 mmol (0-2.0 equiv.) base in 1.0 mL chloroform at 25 °C for 48 hours. [b] Determined by GC-MS analysis. [c] Determined by chiral HPLC analysis using a Chiralpak® AS-H column. Absolute configuration has been determined by measuring the optical rotation and comparing with literature data.

8. General procedure and analytical data for the asymmetric aziridinations

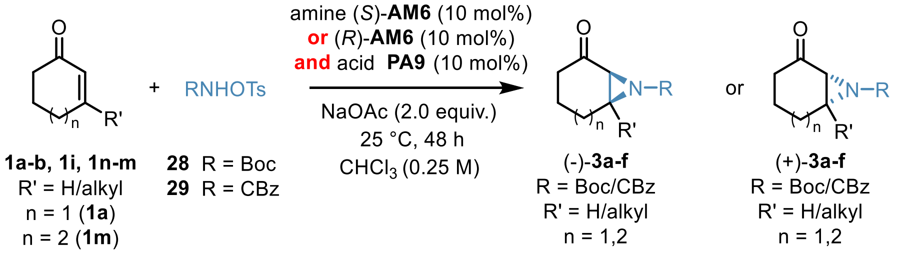

All reactions were performed on a 1.0 mmol scale. In a 20 mL screw cap vial the catalyst was prepared *in situ* by stirring the amine **AM6** (20.4 mg, 10 mol%) and phosphoric acid **PA9** (50.86 mg, 10 mol%) in $CHCl_3$ (4.0 mL, 0.25 M) for 20 min. Then the enone **1a-b**, **1i**, **1n-m** (1.2 mmol, 1.2 equiv.) was added and the reaction mixture was stirred for another 15 min at room temperature. Then, reagent **28**-**29** (1.0 mmol, 1.0 equiv.) and NaOAc (165 mg, 2.0 mmol, 2.0 equiv.) were added. The reaction mixture was stirred at room temperature for 48 h. The crude reaction mixture was washed with brine (1×) and the aqueous phase was extracted with CH_2Cl_2 (3×). The combined organic phases were dried over anhydrous Na_2SO_4 and the solvent was removed under reduced pressure. The crude product was then purified by column chromatography (PE/EE mixtures, vanillin staining agent) to afford the pure aziridines (−)**3a-f** and (+)-**3a-f**.

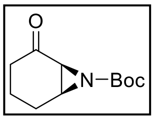

***tert*-Butyl (1*S*,6*S*)-2-oxo-7-azabicyclo[4.1.0]heptane-7-carboxylate, (*S*,*S*)-3a.**[31] Purified by column chromatography (20% EtOAc in PE). White solid (167 mg, 79% yield). $[\alpha]_D^{20}$ = − 143.5 (c 1.0, $CHCl_3$). **^{1}H NMR (400 MHz, $CDCl_3$) δ** 3.08 – 3.05 (m, 1H), 2.88 (d, *J* = 5.8 Hz, 1H), 2.53 – 2.40 (m, 1H), 2.27 – 2.19 (m, 1H), 2.09 – 1.90 (m, 2H), 1.82 – 1.73 (m, 1H), 1.67 – 1.59 (m, 1H), 1.44 (s, 9H). **^{13}C NMR (101 MHz, $CDCl_3$) δ** 204.3, 160.6, 82.2, 43.2, 40.4, 36.9, 27.8, 22.6, 17.3. The **enantiomeric excess** was determined to be 99.5% ee by chiral HPLC (Chiralpak® AS-H column, *n*-Heptane/*i*-PrOH = 95:5, 0.5 mL/min, λ = 220 nm, 25 °C), major enantiomer: t_r = 21.60 min, minor enantiomer: t_r = 23.50 min.

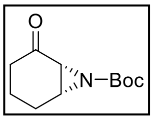

***tert*-Butyl (1*R*,6*R*)-2-oxo-7-azabicyclo[4.1.0]heptane-7-carboxylate, (*R*,*R*)-3a.**[31] Purified by column chromatography (20% EtOAc in PE). $[\alpha]_D^{20}$ = + 106.7 (c 1.0, $CHCl_3$). White solid (166 mg, 79% yield). The **enantiomeric excess** was determined to be 97% ee by chiral HPLC (Chiralpak® AS-H column, *n*-Heptane/*i*-PrOH = 95:5, 0.5 mL/min, λ = 220 nm, 25 °C), minor enantiomer: t_r = 21.69 min, major enantiomer: t_r = 23.12 min.

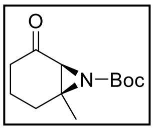

***tert*-Butyl (1*S*,6*S*)-1-methyl-5-oxo-7-azabicyclo[4.1.0]heptane-7-carboxylate**, **(*S*,*S*)-3b.**[31] Purified by column chromatography (20% EtOAc in PE). Colourless oil (175 mg, 78% yield). $[\alpha]_D^{20}$ = – 80.7 (c 1.0, $CHCl_3$). **^{1}H NMR (400 MHz, $CDCl_3$) δ** 2.77 (s, 1H), 2.50 – 2.37 (m, 1H), 2.16 – 2.09 (m, 1H), 2.06 – 1.92 (m, 2H), 1.71 – 1.57 (m, 2H), 1.45 (s, 9H), 1.39 (s, 3H). **^{13}C NMR (101 MHz, $CDCl_3$) δ** 205.6, 159.0, 82.0, 49.68, 47.6, 36.2, 29.3, 28.1, 20.4, 17.5. The **enantiomeric excess** was determined to be 89% ee by chiral HPLC (Chiralpak® AS-H column, *n*-Heptane/ Chiralpak® AS-H column, *n*-Heptane/*i*-PrOH = 90:10, 1 mL/min, λ = 220 nm, 25 °C), minor enantiomer: t_r = 7.70 min, major enantiomer: t_r = 8.52 min.

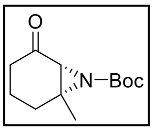

***tert*-Butyl (1*R*,6*R*)-1-methyl-5-oxo-7-azabicyclo[4.1.0]heptane-7-carboxylate**, **(*R*,*R*)-3b.**[31] Purified by column chromatography (20% EtOAc in PE). Colourless oil (172 mg, 76% yield). $[\alpha]_D^{20}$ = + 81.7 (c 1.0, $CHCl_3$). The **enantiomeric excess** was determined to be 88% ee by chiral HPLC (Chiralpak® AS-H column, *n*-Heptane/*i*-PrOH = 90:10, 1 mL/min, λ = 220 nm, 25 °C), major enantiomer: t_r = 7.70 min, minor enantiomer: t_r = 8.65 min.

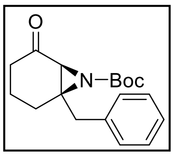

***tert*-Butyl (1*R*,6*S*)-1-Benzyl-5-oxo-7-azabicyclo[4.1.0]heptane-7-carboxylate**, **(*R*,*S*)-3c.**[31] Reaction was performed with 2.0 equiv. $NaHCO_3$ instead of 2.0 equiv. NaOAc on a 0.8 mmol scale. Purified by column chromatography (20% EtOAc in PE). Colourless oil (141 mg, 59% yield). $[\alpha]_D^{20}$ = – 139.1 (c 1.0, $CHCl_3$). **^{1}H NMR (400 MHz, $CDCl_3$) δ** 7.33 - 7.20 (m, 5H), 3.24 (d, *J* = 14.3 Hz, 1H), 3.06 (s, 1H), 2.47 – 2.35 (m, 2H), 2.08 – 2.00 (m, 1H), 1.99 – 1.86 (m, 2H), 1.66 – 1.51 (m, 2H), 1.49 (s, 9H). **^{13}C NMR (101 MHz, $CDCl_3$) δ** 205.1, 158.6, 136.4, 129.5, 128.9, 127.3, 82.4, 51.3, 48.8, 41.4, 36.2, 28.1, 26.2, 17.6. The **enantiomeric excess** was determined to be 88% ee by chiral HPLC (Chiralpak® AS-H column, *n*-Heptane/*i*-PrOH = 90:10, 1 mL/min, λ = 220 nm, 25 °C), minor enantiomer: t_r = 6.45 min, major enantiomer: t_r = 15.17 min.

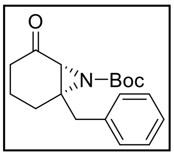

***tert*-Butyl (1*S*,6*R*)-1-Benzyl-5-oxo-7-azabicyclo[4.1.0]heptane-7-carboxylate**, **(*S*,*R*)-3c.**[31] Reaction was performed with 2.0 equiv. $NaHCO_3$ instead of 2.0 equiv. NaOAc on a 0.8 mmol scale. Purified by column chromatography (20% EtOAc in PE). Colourless oil (135 mg, 56% yield). $[\alpha]_D^{20}$ = + 137.5 (c 1.0, $CHCl_3$). The **enantiomeric excess** was determined to be 89% ee by chiral HPLC Chiralpak® AS-H column, *n*-Heptane/*i*-PrOH = 90:10, 1 mL/min, λ = 220 nm, 25 °C), major enantiomer: t_r = 6.43 min, minor enantiomer: t_r = 15.20 min.

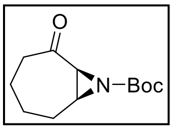

***tert*-butyl (1*S*,7*S*)-2-Oxo-8-azabicyclo[5.1.0]octane-8-carboxylate**, **(*S*,*S*)-3d.**[31] Purified by column chromatography (10% EtOAc in PE). Colourless oil (155 mg, 69% yield). $[\alpha]_D^{20}$ = – 116.13 (c 0.98, CH_2Cl_2). **^{1}H NMR (400 MHz, $CDCl_3$) δ** 2.95 (d, *J* = 5.9, 1H), 2.82

(dd, *J* = 5.9, *J* = 1.6 Hz, 1H), 2.70 – 2.66 (m, 1H), 2.38 – 2.35 (m, 1H), 2.21 - 2.19 (m, 1H), 1.72 – 1.49 (m, 5H), 1.38 (s, 9H). **^{13}C NMR (101 MHz, CDCl$_3$) δ** 209.3, 160.9, 82.0, 47.3, 40.8, 40.4, 27.9, 23.7, 23.5. The **enantiomeric excess** was determined to be 92% ee by chiral HPLC (Chiralpak® AS-H column, *n*-Heptane/*i*-PrOH = 90:10, 1 mL/min, λ = 220 nm, 25 °C), minor enantiomer: t_r = 7.20 min, major enantiomer: t_r = 8.44 min.

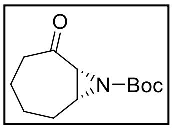

***tert*-butyl (1*R*,7R)-2-Oxo-8-azabicyclo[5.1.0]octane-8-carboxylate, (*R*,*R*)-3d.**[31] Purified by column chromatography (10% EtOAc in PE). Colourless oil (163 mg, 72% yield). The **enantiomeric excess** was determined to be 93% ee by chiral HPLC (Chiralpak® AS-H column, *n*-Heptane/*i*-PrOH = 90:10, 1 mL/min, λ = 220 nm, 25 °C), major enantiomer: t_r = 7.18 min, major enantiomer: t_r = 8.48 min.

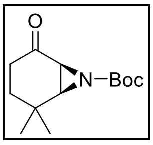

***tert*-Butyl (1*S*,6*S*)-2,2-Dimethyl-5-oxo-7-azabicyclo[4.1.0]heptane-7-carboxylate, (*S*,*S*)-3e.**[31] Purified by column chromatography (15% EtOAc in PE). Colourless oil (175 mg, 76% yield). $[\alpha]_D^{20}$ = – 100.1 (c 1.0, CHCl$_3$). **^{1}H NMR (400 MHz, CDCl$_3$) δ** 2.92 (d, *J* = 5.9, 1H), 2.66 (dd, *J* = 5.9, *J* = 1.6 Hz, 1H), 2.35 (ddd, *J* = 19.1, *J* = 6.5, *J* = 2.4 Hz, 1H), 2.21 – 2.11 (m, 1H), 1.96 – 1.84 (m, 1H), 1.44 (s, 9H), 1.32 – 1.24 (m, 1H), 1.21 (s, 3H), 1.03 (s, 3H). **^{13}C NMR (101 MHz, CDCl$_3$) δ** 204.7, 160.5, 82.2, 50.2, 44.2, 33.8, 30.4, 28.0, 27.7, 23.4. The **enantiomeric excess** was determined to be 93% ee by chiral HPLC (Chiralpak® AS-H column, *n*-Heptane/*i*-PrOH = 90:10, 1 mL/min, λ = 220 nm, 25 °C), major enantiomer: t_r = 6.85 min, minor enantiomer: t_r = 8.56 min.

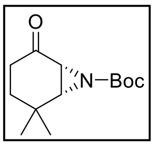

***tert*-Butyl (1*R*,6*R*)-2,2-Dimethyl-5-oxo-7-azabicyclo[4.1.0]heptane-7-carboxylate, (*R*,*R*)-3e.**[31] Purified by column chromatography (15% EtOAc in PE). Colourless oil (182 mg, 75% yield). $[\alpha]_D^{20}$ = + 103.4 (c 1.0, CHCl$_3$). The **enantiomeric excess** was determined to be 92% ee by chiral HPLC (Chiralpak® AS-H column, *n*-Heptane/*i*-PrOH = 90:10, 1 mL/min, λ = 220 nm, 25 °C), minor enantiomer: t_r = 7.35 min, major enantiomer: t_r = 8.94 min.

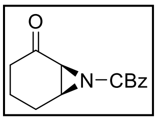

(1*S*,6*S*)-7-(2-Oxo-2-phenyl-1λ^2-ethyl)-7-azabicyclo[4.1.0]heptan-2-one, (*S*,*S*)-3f.[31] Purified by column chromatography (gradient elution, 20% to 33% EtOAc in PE). Colourless oil (174 mg, 71% yield). $[\alpha]_D^{20}$ = – 75.77 (c 1.34, CH$_2$Cl$_2$). **^{1}H NMR (400 MHz, CDCl$_3$)** δ 7.28 - 7.22 (m, 5H), 5.04 (dd, *J* = 12.0 Hz, 14.0 Hz, 2H), 3.06 - 3.05 (m, 1H), 2.89 (d, *J* = 8.0 Hz, 1H), 2.40 - 2.35 (m, 1H), 2.17 - 2.13 (m, 1H), 1.99 - 1.78 (m, 2H), 1.72 - 1.66 (m, 1H), 1.57 - 1.51 (m, 1H). **^{13}C NMR (101 MHz, CDCl$_3$)** δ 203.7, 161.5, 133.4, 128.6, 128.5, 128.3, 68.6, 42.9, 40.6, 36.9, 30.4, 22.5, 17.1. The **enantiomeric excess** was determined to be 93% ee by chiral HPLC (Chiralpak® AS-H column,

n-Heptane/*i*-PrOH = 95:5, 1 mL/min, λ = 220 nm, 25 °C), major enantiomer: t_r = 31.89 min, minor enantiomer: t_r = 49.23 min.

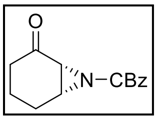

(1*R*,6*R*)-7-(2-Oxo-2-phenyl-1λ^2-ethyl)-7-azabicyclo[4.1.0]heptan-2-one, (*R*,*R*)-3f.[31] Purified by column chromatography (gradient elution, 20% to 33% EtOAc in PE). Colourless oil (180 mg, 73% yield). $[\alpha]_D^{20}$ = + 84.71 (c 0.95, CH_2Cl_2).The **enantiomeric excess** was determined to be 92% ee by chiral HPLC (AS-H column, *n*-Heptane/*i*-PrOH = 95:5, 1 mL/min, λ = 220 nm, 25 °C), minor enantiomer: t_r = 32.34 min, major enantiomer: t_r = 48.69 min.

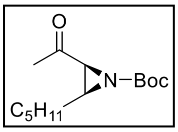

(*tert*-butyl (2*S*,3*S*)-2-Acetyl-3-pentylaziridine-1-carboxylate, (*S*,*S*)-3g.[31] Purified by column chromatography (12% EtOAc in PE). Colourless oil (175 mg, 78% yield). $[\alpha]_D^{20}$ = − 5.3 (c 1.0, CH_2Cl_2). **^{1}H NMR (400 MHz, $CDCl_3$)** δ 2.91 (d, *J* = 2.8 Hz, 1H), 2.60 – 2.56 (m, 1H), 2.20 (s, 3H), 1.54 – 1.51 (m, 1H), 1.43 – 1.34 (m, 12H), 1.27 – 1.22 (m, 4H), 0.82 (t, *J* = 8.0 Hz, 3H). **^{13}C NMR (101 MHz, $CDCl_3$)** δ 202.7, 159.1, 81.7, 46.7, 45.6, 31.3, 29.1, 28.0, 26.5, 22.5, 14.0. The **diastereometic ratio** was 33/1 d.r. based on the ^{1}H NMR spectrum. The **enantiomeric excess** was determined to be 64% ee by chiral HPLC (Chiralpak® IA-3 column, *n*-Hexane/*i*-PrOH = 99:1, 1 mL/min, λ = 235 nm, 25 °C), minor enantiomer: t_r = 7.78 min, major enantiomer: t_r = 11.88 min.

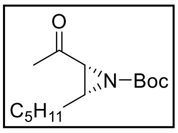

(*tert*-butyl (2*R*,3*R*)-2-Acetyl-3-pentylaziridine-1-carboxylate, (*R*,*R*)-3g.[31] Purified by column chromatography (12% EtOAc in PE). Colourless oil (170 mg, 75% yield). $[\alpha]_D^{20}$ = + 5.5 (c 1.0, CH_2Cl_2). The **diastereometic ratio** was 33/1 d.r. based on the ^{1}H NMR spectrum. The **enantiomeric excess** was determined to be 70% ee by chiral HPLC (Chiralpak® IA-3 column, *n*-Hexane/*i*-PrOH = 99:1, 1 mL/min, λ = 235 nm, 25 °C), major enantiomer: t_r = 7.72 min, minor enantiomer: t_r = 12.14 min.

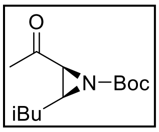

(*tert*-butyl (2*S*,3*S*)-2-Acetyl-3-isobutylaziridine-1-carboxylate, (*S*,*S*)-3h. Purified by column chromatography (10% EtOAc in PE). Colourless oil (164 mg, 68% yield). $[\alpha]_D^{20}$ = − 8.56 (c 1.0, CH_2Cl_2). **^{1}H NMR (400 MHz, $CDCl_3$)** δ 2.91 (d, *J* = 2.8 Hz, 1H), 2.62 – 2.59 (m, 1H), 2.21 (s, 3H), 1.80 – 1.73 (m, 1H), 1.48 – 1.43 (m, 1H), 1.39 (s, 9H), 1.25 – 1.19 (m, 1H), 0.92 – 0.90 (m, 6H). **^{13}C NMR (101 MHz, $CDCl_3$)** δ 202.6, 159.1, 81.7, 46.9, 44.4, 40.3, 29.3, 27.9, 26.9, 22.7, 22.0. The **diastereometic ratio** was 33/1 d.r. based on the ^{1}H NMR spectrum. The **enantiomeric excess** was determined to be 64% ee by chiral HPLC (Chiralpak® IA-3 column, *n*-Hexane/*i*-PrOH = 99:1, 1 mL/min, λ = 235 nm, 25 °C), minor enantiomer: t_r = 7.66 min, major enantiomer: t_r = 12.30 min. **IR ATR (vmax/cm^{-1})** 2958, 1724, 1423, 1149, 802. **HRMS (ESI)** Cald for $C_8H_{16}NO_2$ $[M+H-BOC]^+$ 142.1226, Found 142.1233.

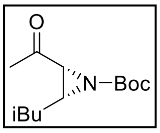

(*tert*-butyl (2*R*,3*R*)-2-Acetyl-3-isobutylaziridine-1-carboxylate, (*R*,*R*)-3h. Purified by column chromatography (10% EtOAc in PE). Colourless oil (170 mg, 70% yield). $[\alpha]_D^{20}$ = + 8.91 (c 1.0, CH_2Cl_2). The **diastereometic ratio** was 33/1 d.r. based on the ^1H NMR spectrum. The **enantiomeric excess** was determined to be 66% ee by chiral HPLC (Chiralpak® IA-3 column, *n*-Hexane/*i*-PrOH = 99:1, 1 mL/min, λ = 235 nm, 25 °C), major enantiomer: t_r = 7.66 min, minor enantiomer: t_r = 12.34 min.

9. General procedure and analytical data for the asymmetric aza-Michael additions

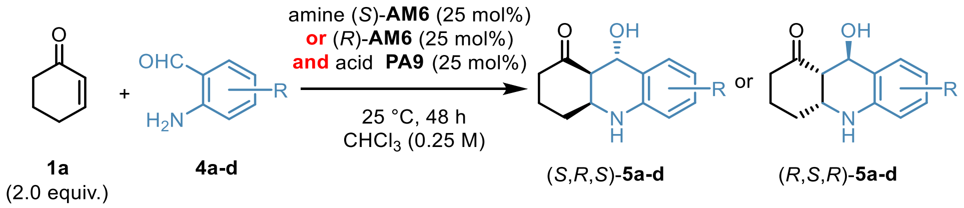

In an 8 mL screw cap vial the catalyst was prepared *in situ* by stirring the amine **AM6** (25 mol%) and phosphoric acid **PA9** (25 mol%) in $CHCl_3$ (0.25 M) for 20 min. Then, 2-cyclohexen-1-one (**1a**, 2.0 equiv.) was added and the reaction mixture was stirred for another 20 min at room temperature. Then aminobenzaldehyde **4a-d** was added in one portion. The reaction mixture was stirred at room temperature for 48 h. After this 15 % *i*-PrOH in *n*-hexane was added and the mixture was placed in an ultrasound bath for 1min. The solid was filtered off, it was successively washed with low amounts of 15% *i*-PrOH in *n*-hexane (3×) (13% *i*-PrOH in *n*-hexane for **5e**) and with pure *n*-hexane (3×) and it was dried *in vacuo* to provide the pure product **5a-d** as white powder.

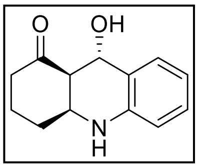

(4a*S*,9*R*,9a*S*)-9-Hydroxy-3,4,4a,9,9a,10-hexahydroacridin-1(2*H*)-one, (*S*,*R*,*S*)-**5a.** Performed with 1.24 mmol 2-aminobenzaldehyde (**4a**). White powder (171 mg, 64% yield). **M.p.** = 197 – 199 °C. $[\alpha]_D^{20}$ = – 178.7 (c 0.66, CH_2Cl_2). **^{1}H NMR (400 MHz, DMSO-*d*$_6$) δ** 7.09 (dd, *J* = 7.6, 1.6 Hz, 1H), 6.91 (ddd, *J* = 8.0, 7.2, 1.6 Hz, 1H), 6.55 – 6.47 (m, 2H), 5.76 (s, 1H), 5.02 (d, *J* = 5.5 Hz, 1H), 4.74 (dd, *J* = 5.5, 2.1 Hz, 1H), 3.84 (q, *J* = 3.2 Hz, 1H), 2.77 (dt, *J* = 2.4, 1.2 Hz, 1H), 2.46 – 2.33 (m, 1H), 2.13 – 1.92 (m, 4H), 1.88 – 1.78 (m, 1H). **^{13}C NMR (101 MHz, DMSO-*d*$_6$) δ** 210.06, 144.74, 130.57, 127.62, 121.84, 115.86, 114.04, 63.00, 53.99, 48.42, 40.52, 28.74, 21.85. The **enantiomeric excess** was determined to be >99% ee by chiral HPLC (Chiralpak® IB column, *n*-Hexane/*i*-PrOH = 85:15, 1 mL/min, λ = 220 nm, 25 °C), minor enantiomer: t_r = 18.61 min, major enantiomer: t_r = 22.48 min. **IR ATR (vmax/cm^{-1})** 3370, 3167, 1692, 1496, 750. **HRMS (ESI)** Cald for $C_{13}H_{15}NO_2$ $[M]^+$ 217.1103, Found 217.1058.

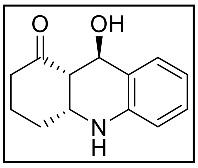

(4a*R*,9*S*,9a*R*)-9-Hydroxy-3,4,4a,9,9a,10-hexahydroacridin-1(2*H*)-one, (*R*,*S*,*R*)-**5a.** Performed with 1.24 mmol 2-aminobenzaldehyde (**4a**). White powder (164 mg, 61% yield). $[\alpha]_D^{20}$ = + 132.9 (c 0.48, CH_2Cl_2). The **enantiomeric excess** was determined to be >99% ee by chiral HPLC (Chiralpak® IB column, *n*-Hexane/*i*-PrOH = 85:15, 1 mL/min, λ = 220 nm, 25 °C), major enantiomer: t_r = 18.67 min, minor enantiomer: t_r = 22.48 min.

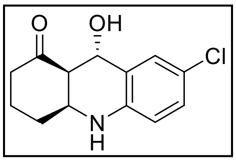

(4a*S*,9*R*,9a*S*)-7-Chloro-9-hydroxy-3,4,4a,9,9a,10-hexahydroacridin-1(2*H*)-one, (*S*,*R*,*S*)-**5b.** Performed with 0.60 mmol 2-amino-5-chlorobenzaldehyde (**4b**). White powder (90 mg, 56% yield). **M.p. =** 184 – 186 °C. $[\alpha]_D^{20}$ = – 130.9 (c 0.80, CH_2Cl_2). **^{1}H NMR (400 MHz, DMSO-*d*$_6$) δ** 7.10 (d, *J* = 2.5 Hz, 1H), 6.94 (dd, *J* = 8.6, 2.6 Hz, 1H), 6.51 (d, *J* = 8.6 Hz, 1H), 6.01 (s, 1H), 5.21 (d, *J* = 5.3 Hz, 1H), 4.71 (dd, *J* = 5.3, 2.3 Hz, 1H), 3.82 (q, *J* = 3.1 Hz, 1H), 2.79 (ddt, *J* = 3.6, 2.3, 1.1 Hz, 1H), 2.46 – 2.35 (m, 1H), 2.08 (ddd, *J* = 14.8, 3.9, 1.8 Hz, 1H), 1.97 (t, *J* = 3.0 Hz, 3H), 1.89 – 1.78 (m, 1H). **^{13}C NMR (101 MHz, DMSO-*d*$_6$) δ** 209.94, 143.57, 129.75, 127.42, 123.45, 118.85, 115.50, 62.71, 53.33, 48.49, 40.46, 28.57, 21.89. The **enantiomeric excess** was determined to be 93% ee by chiral HPLC (Chiralpak® AS-H column, *n*-Hexane/*i*-PrOH = 65:35, 1 mL/min, λ = 220 nm, 25 °C), major enantiomer: t_r = 7.93 min, minor enantiomer: t_r =14.53 min. **IR ATR (vmax/cm^{-1})** 3371, 3192, 1692, 1500, 820. **HRMS (ESI)** Cald for $C_{13}H_{14}ClNO_2$ $[M]^+$ 251.0713, Found 251.0689.

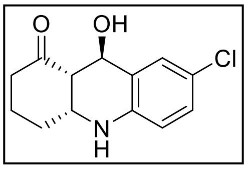

(4a*R*,9*S*,9a*R*)-7-Chloro-9-hydroxy-3,4,4a,9,9a,10-hexahydroacridin-1(2*H*)-one , (*R*,*S*,*R*)-**5b.** Performed with 0.6 mmol 2-amino-5-chlorobenzaldehyde (**4c**). White powder (89 mg, 55% yield). $[\alpha]_D^{20}$ = + 137.0 (c 0.66, CH_2Cl_2). The **enantiomeric excess** was determined to be 91% ee by chiral HPLC (Chiralpak® AS-H column, *n*-Hexane/*i*-PrOH = 65:35, 1 mL/min, λ = 220 nm, 25 °C), minor enantiomer: t_r = 7.82 min, major enantiomer: t_r =13.89 min.

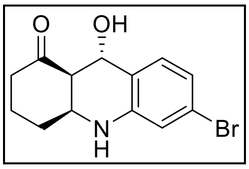

(4a*S*,9*R*,9a*S*)-6-Bromo-9-hydroxy-3,4,4a,9,9a,10-hexahydroacridin-1(2*H*) - (*S*,*R*,*S*)-**5c.** Performed with 0.6 mmol 2-amino-4-bromobenzaldehyde (**4c**). White powder (90 mg, 61% yield). **M.p. =** 183 – 185 °C. $[\alpha]_D^{20}$ = – 118.6 (c 0.62, CH_2Cl_2). **^{1}H NMR (400 MHz, DMSO-*d*$_6$) δ** 7.02 (d, *J* = 8.0 Hz, 1H), 6.70 – 6.61 (m, 2H), 6.12 (s, 1H), 5.13 (d, *J* = 5.4 Hz, 1H), 4.70 (dd, *J* = 5.4, 2.3 Hz, 1H), 3.84 (q, *J* = 2.8, 2.4 Hz, 1H), 2.79 (td, *J* = 2.5, 1.2 Hz, 1H), 2.46 – 2.36 (m, 1H), 2.11 – 2.02 (m, 1H), 1.99 – 1.91 (m, 3H), 1.85 (pt, *J* = 6.9, 4.2, 3.4 Hz, 1H). **^{13}C NMR (101 MHz, DMSO-*d*$_6$) δ** 209.87, 146.30, 132.37, 120.96, 120.58, 118.05, 115.72, 62.59, 53.26, 48.16, 40.48, 28.52, 21.81. The **enantiomeric excess** was determined to be >99% ee by chiral HPLC (Chiralpak® AS-H column, *n*-Hexane/*i*-PrOH = 65:35, 1 mL/min, λ = 220 nm, 25 °C), major enantiomer: t_r = 8.74 min, minor enantiomer: t_r = 12.08 min. **IR ATR (vmax/cm^{-1})** 3368, 3225, 1692, 1607, 1491, 989. **HRMS (ESI)** Cald for $C_{13}H_{14}BrNO_2$ $[M]^+$ 295.0208, Found 295.0199.

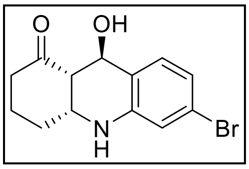

(4a*R*,9*S*,9a*R*)-6-Bromo-9-hydroxy-3,4,4a,9,9a,10-hexahydroacridin-1(2*H*)-one, (*R*,*S*,*R*)-**5c.** Performed with 0.6 mmol 2-amino-4-bromobenzaldehyde (**4c**). White powder (87 mg, 59% yield). $[\alpha]_D^{20}$ = + 125.2 (c 0.74, CH_2Cl_2). The

enantiomeric excess was to be >99% ee by chiral HPLC (Chiralpak® AS-H column, *n*-Hexane/*i*-PrOH = 65:35, 1 mL/min, λ = 220 nm, 25 °C), minor enantiomer: t_r = 9.83 min, major enantiomer: t_r = 12.74 min.

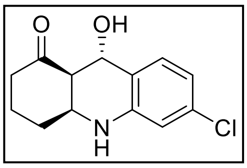

(4a*S*,9*R*,9a*S*)-6-Chloro-9-hydroxy-3,4,4a,9,9a,10-hexahydroacridin-1(2*H*) -one, (*S*,*R*,*S*)-**5d**. Performed with 0.6 mmol 2-amino-4-chlorobenzaldehyde (**4d**). White powder (109 mg, 69% yield). **M.p. =** 172 – 174 °C. $[\alpha]_D^{20}$ = – 185.5 (c 0.483, CH_2Cl_2). **^{1}H NMR (400 MHz, DMSO-d$_6$) δ** 7.08 (d, *J* = 7.8 Hz, 1H), 6.51 (d, *J* = 7.4 Hz, 2H), 6.13 (s, 1H), 5.10 (d, *J* = 5.4 Hz, 1H), 4.71 (dd, *J* = 5.4, 2.3 Hz, 1H), 3.85 (q, *J* = 2.8, 2.4 Hz, 1H), 2.79 (td, *J* = 2.6, 1.3 Hz, 1H), 2.43 (td, *J* = 12.1, 6.1 Hz, 1H), 2.12 – 2.02 (m, 1H), 1.97 (q, *J* = 6.4, 3H), 1.86 (dq, *J* = 8.5, 5.8, 4.8 Hz, 1H). **^{13}C NMR (101 MHz, DMSO-d$_6$) δ** 209.71, 145.96, 131.99, 131.90, 120.57, 115.13, 112.73, 62.46, 53.27, 48.09, 40.41, 28.47, 21.73. The **enantiomeric excess** was determined to be >99% ee by chiral HPLC (Chiralpak® AS-H column, *n*-Hexane/*i*-PrOH = 65:35, 1 mL/min, λ = 220 nm, 25 °C), major enantiomer: t_r = 8.22 min, minor enantiomer: t_r = 11.70 min. **IR ATR (vmax/cm^{-1})** 3366, 3169, 1692, 1613, 1476, 992. **HRMS (ESI)** Cald for $C_{13}H_{15}ClNO_2$ $[M+H]^+$ 252.0786, Found 252.0790.

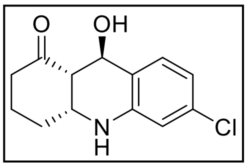

(4a*R*,9*S*,9a*R*)-6-Chloro-9-hydroxy-3,4,4a,9,9a,10-hexahydroacridin-1(2*H*) -one, (*R*,*S*,*R*)-**5d**. Performed with 0.6 mmol 2-amino-4-chlorobenzaldehyde (**4d**). White powder (109 mg, 69% yield). $[\alpha]_D^{20}$ = + 180.0 (c 0.48, CH_2Cl_2). The **enantiomeric excess** was determined to be >99% ee by chiral HPLC (Chiralpak® AS-H column, *n*-Hexane/*i*-PrOH = 65:35, 1 mL/min, λ = 220 nm, 25 °C), minor enantiomer: t_r = 8.27 min, majorenantiomer: t_r = 11.65 min.

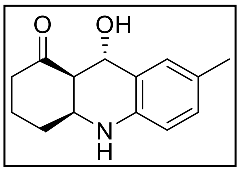

(4a*S*,9*R*,9a*S*)-9-Hydroxy-7-methyl-3,4,4a,9,9a,10-hexahydroacridin-1(2H)-one, (*S*,*R*,*S*)-**5e**. Performed with 0.74 mmol 2-amino-5-methylbenzaldehyde (**4e**). White powder (86 mg, 50% yield). **M.p. =** 183 – 185 °C. $[\alpha]_D^{20}$ = – 178,36 (c 0.304, CH_2Cl_2). **^{1}H NMR (400 MHz, DMSO-d$_6$)** δ 6.92 (d, *J* = 2.2 Hz, 1H), 6.74 (dd, *J* = 8.1, 2.1 Hz, 1H), 6.41 (d, *J* = 8.1 Hz, 1H), 5.57 (s, 1H), 4.95 (d, *J* = 5.6 Hz, 1H), 4.70 (dd, *J* = 5.6, 2.1 Hz, 1H), 3.81 (q, *J* = 3.2 Hz, 1H), 2.75 (dt, *J* = 3.7, 1.7 Hz, 1H), 2.41 (td, *J* = 14.1, 13.3, 6.3 Hz, 1H), 2.15 (s, 3H), 2.11 – 1.89 (m, 4H), 1.83 (ddd, *J* = 11.2, 8.2, 4.3 Hz, 1H). **^{13}C NMR (101 MHz, DMSO-d$_6$)** δ 209.96, 142.35, 130.82, 128.20, 124.03, 121.77, 114.07, 62.91, 54.19, 48.53, 40.44, 28.77, 21.81, 20.15. The **enantiomeric excess** was determined to be >99% ee by chiral HPLC (Chiralpak® AS-H column, *n*-Hexane/*i*-PrOH = 70:30, 1 mL/min, λ = 220 nm, 25 °C), major enantiomer: t_r = 8.99 min, minor enantiomer: t_r = 24.2 min. **IR ATR (vmax/cm^{-1})** 3374, 3194, 1695, 1624, 1457, 990. **HRMS (ESI)** Cald for $C_{14}H_{18}NO_2$ $[M+H]^+$ 232.1332, Found 232.1335.

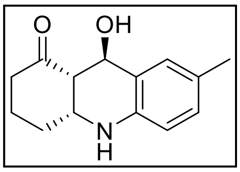

(4a*R*,9*S*,9a*R*)-9-Hydroxy-7-methyl-3,4,4a,9,9a,10-hexahydroacridin-1(2*H*)-one, (*R*,*S*,*R*)-**5e**. Performed with 0.74 mmol 2-amino-5-methylbenzaldehyde (**4e**). White powder (85 mg, 50% yield). $[\alpha]_D^{20} = + 177.97$ (c 0.295, CH_2Cl_2). The **enantiomeric excess** was determined to be >99% ee by chiral HPLC (Chiralpak® AS-H column, *n*-Hexane/*i*-PrOH = 70:30, 1 mL/min, λ = 220 nm, 25 °C), minor enantiomer: t_r = 9.14 min, major enantiomer: t_r = 23.3 min.

10. General procedure and analytical data for the asymmetric Michael-Initiated Ring-Closure/Intramolecular Aldol Sequence

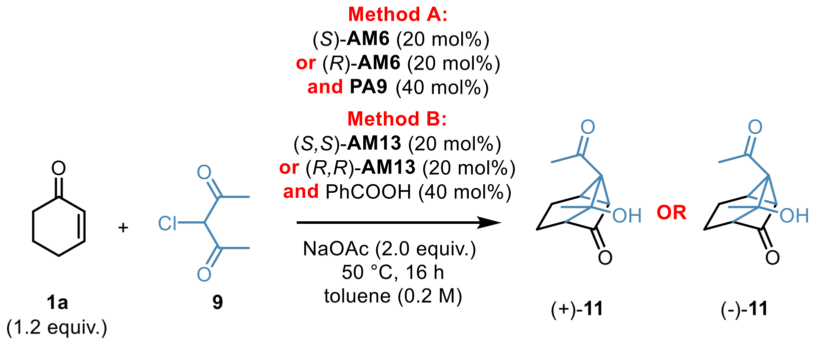

Reactions were performed on a 1.0 mmol scale. In a 20 mL screw cap vial the catalyst was prepared *in situ* by stirring the amine (**AM6** or **AM13**, 20 mol%) and acid (**PA9** or PhCOOH**,** 40 mol%) in anhydrous toluene (0.20 M) for 20 min. Then, 2-cyclohexen-1-one (**1a**, 1.2 equiv.) was added and the reaction mixture was stirred for another 20 min at room temperature. Then 3-chloroacetylacetone (**9,** 1.0 equiv.) and NaOAc (2.0 equiv.) were added and the reaction mixture was stirred at 50 ˚C for 16 hours. After being cooled down to room temperature, the reaction was quenched with H_2O (10 mL) and the aqueous phase was extracted with toluene (2×) and EtOAc (2×). The combined organic phases were washed with saturated $NaHCO_3$ (2×), dried over anhydrous Na_2SO_4 and the solvents were removed *in vacuo*. The crude product was purified by column chromatography (gradient elution, PE/EE mixtures).

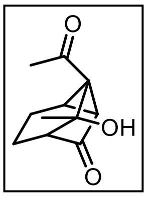

1-Acetyl-8-hydroxy-8-methyltricyclo[3.2.1.0^{2,7}]octan-6-one (−)**-11a.** Purified by column chromatography (33% EtOAc in PE to 100% EtOAc, R_f = 0.35 in PE/EtOAc 1/1). White solid (155 mg, 77% yield). **M.p. =** 85 – 87 °C. $[\alpha]_D^{20}$ = – 114.14 (c 1.02, CH_2Cl_2). **^{1}H NMR (600 MHz, $CDCl_3$)** δ 3.99 (bs, 1H, *O*H), 2.60 (ddt, *J* = 8.5, 3.0, 1.5 Hz, 1H, *H*2), 2.48 (dd, *J* = 8.6, 1.5 Hz, 1H, *H*7), 2.13 – 1.99 (m, 4H, *H*3a, *H*3b, *H*4a, *H*5), 1.99-1.94 (m, 1H, *H*4b), 1.93 (s, 3H, COC*H*$_3$), 1.62 (s, 3H, C*H*$_3$). **^{13}C NMR (150 MHz, $CDCl_3$)** δ 209.79, 206.08, 74.99, 52.43, 52.26, 38.68, 36.29, 25.41, 25.12, 15.47. The product was obtained as a single diastereomer. The **enantiomeric excess** was determined by chiral HPLC to be 85% ee and 95% ee by using [(*S*)-**AM6**][**PA9**] and [(*S*,*S*)-**AM13**][2PhCOOH], respectively (Chiralpak® OJ column, *n*-Hexane/*i*-PrOH = 90:10, 1 mL/min, λ = 220 nm, 25 °C), major enantiomer: t_r = 22.09 min, minor enantiomer: t_r = 26.02 min. **IR ATR (vmax/cm^{-1})** 3045, 2976, 1717, 1682, 1353, 1225, 876. **HRMS (ESI)** Cald for $C_{11}H_{15}O_3$ $[M+H]^+$ 195.1021, Found 195.1018**.**

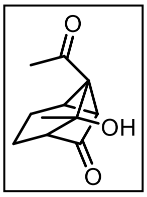

1-Acetyl-8-hydroxy-8-methyltricyclo[3.2.1.0^{2,7}]octan-6-one (+)-**11a.** White solid (152 mg, 79% yield). $[\alpha]_D^{20}$ = + 111.91 (c 1.0, CH_2Cl_2). The product was obtained as a single diastereomer. The **enantiomeric excess** was determined by chiral HPLC to be 87% ee and 97% ee by using [(*R*)-**AM6**][**PA9**] and [(*R*,*R*)-**AM13**][2PhCOOH], respectively (Chiralpak® OJ column, *n*-Hexane/*i*-PrOH = 90 : 10, 1 mL/min, λ = 220 nm, 25 °C), minor enantiomer: t_r = 22.83 min, major enantiomer: t_r = 25.22 min.

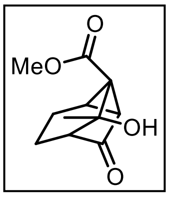

Methyl-8-hydroxy-8-methyl-6-oxotricyclo[3.2.1.0^{2,7}]octane-1-carboxylate (−)-**11b.** Purified by column chromatography (25% EtOAc in PE to 33% EtOAc in PE). Light yellow oil (119 mg, 52% yield). $[\alpha]_D^{20}$ = − 21.30 (c 1.0, CH_2Cl_2). **^{1}H NMR (400 MHz, $CDCl_3$)** δ 3.67 (s, 3H), 3.65 (s, 1H), 2.53 – 2.52 (m, 1H), 2.34 (d, *J* = 8.0 Hz, 1H), 1.97 – 1.87 (m, 5H), 1.56 (s, 3H). **^{13}C NMR (100 MHz, $CDCl_3$)** δ 210.2, 171.1, 74.4, 53.2, 52.2, 44.3, 38.9, 36.3, 25.5, 21.9, 15.1. The **enantiomeric excess** was determined by chiral HPLC to be 95% ee and 99% ee by using [(*S*)-**AM6**][**PA9**] and [(*S*,*S*)-**AM13**][2PhCOOH], respectively (Chiralpak® OJ column, *n*-Hexane/*i*-PrOH = 93:7, 0.8 mL/min, λ = 220 nm, 25 °C), major enantiomer: t_r = 29.66 min, minor enantiomer: t_r = 32.41 min. **IR ATR (vmax/cm^{-1})** 3511, 2954, 1721, 1483, 1221, 871. **HRMS (ESI)** Cald for $C_{11}H_{14}O_4Na$ $[M+Na]^+$ 233.0784, Found 233.0790**.**

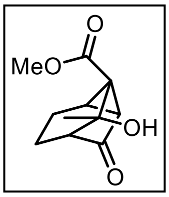

Methyl-8-hydroxy-8-methyl-6-oxotricyclo[3.2.1.0^{2,7}]octane-1-carboxylate (+)-**11b.** Purified by column chromatography (25% EtOAc in PE to 50% EtOAc in PE). Light yellow oil (119 mg, 52% yield). $[\alpha]_D^{20}$ = + 23.1 (c 1.0, CH_2Cl_2). The **enantiomeric excess** was determined by chiral HPLC to be 94% ee and 99% ee by using [(*R*)-**AM6**][**PA9**] and [(*R*,*R*)-**AM13**][2PhCOOH], respectively (Chiralpak® OJ column, *n*-Hexane/*i*-PrOH = 93:7, 0.8 mL/min, λ = 220 nm, 25 °C), minor enantiomer: t_r = 30.45 min, major enantiomer: t_r = 31.73 min.

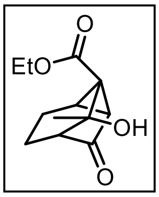

Ethyl-8-hydroxy-8-methyl-6-oxotricyclo[3.2.1.0^{2,7}]octane-1-carboxylate (−)-**11c.** Purified by column chromatography (25% EtOAc in PE to 50% EtOAc in PE). Light yellow oil (112 mg, 50% yield). $[\alpha]_D^{20}$ = − 25.4 (c 1.0, CH_2Cl_2). **^{1}H NMR (400 MHz, $CDCl_3$)** δ 3.67 (s, 3H), 3.65 (s, 1H), 2.53 – 2.52 (m, 1H), 2.34 (d, *J* = 8.0 Hz, 1H), 1.97 – 1.87 (m, 5H), 1.56 (s, 3H). **^{13}C NMR (100 MHz, $CDCl_3$)** δ 210.2, 171.1, 74.4, 53.2, 52.2, 44.3, 38.9, 36.3, 25.5, 21.9, 15.1. The **enantiomeric excess** was determined by chiral HPLC to be 92% ee and 97% ee by using [(*S*)-**AM6**][**PA9**] and [(*S*,*S*)-**AM13**][2PhCOOH], respectively (Chiralpak® OJ column, *n*-Hexane/*i*-PrOH = 93:7, 0.8 mL/min, λ = 220 nm, 25 °C), major enantiomer: t_r = 29.66 min, minor enantiomer: t_r = 32.41 min. **IR ATR (vmax/cm^{-1})** 3511, 2954, 1721, 1483, 1221, 871. **HRMS (ESI)** Cald for $C_{12}H_{17}O_4$ $[M+H]^+$ 225.1122, Found 225.1123**.**

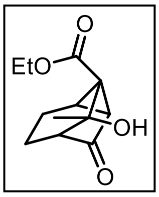

Methyl-8-hydroxy-8-methyl-6-oxotricyclo[3.2.1.0^{2,7}]octane-1-carboxylate (+)-**11c.** Purified by column chromatography (25% EtOAc in PE to 50% EtOAc in PE). Light yellow oil (114 mg, 51% yield). $[\alpha]_D^{20}$ = + 21.5 (c 1.0, CH_2Cl_2). The **enantiomeric excess** was determined by chiral HPLC to be 90% ee and 98% ee by using [(*R*)-**AM6**][**PA9**] and [(*R*,*R*)-**AM13**][2PhCOOH], respectively (Chiralpak® OJ column, *n*-Hexane/*i*-PrOH = 93:7, 0.8 mL/min, λ = 220 nm, 25 °C), minor enantiomer: t_r = 30.45 min, major enantiomer: t_r = 31.73 min.

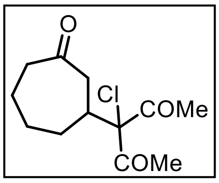

3-Chloro-3-(3-oxocycloheptyl)pentane-2,4-dione (−)-**10b.** Purified by column chromatography (12% EtOAc in PE to 20% EtOAc in PE). Colorless oil (173 mg, 71% yield). $[\alpha]_D^{20}$ = − 8.56 (c 1.0, CH_2Cl_2). **^{1}H NMR (400 MHz, $CDCl_3$)** δ 2.88 – 2.83 (m, 1H), 2.60 – 2.47 (m, 3H), 2.27 – 2.26 (m, 6H), 2.23 – 2.19 (m, 1H), 1.94 – 1.91 (m, 2H), 1.68 – 1.65 (m, 1H), 1.54 – 1.47 (m, 1H), 1.40 – 1.35 (m, 2H). **^{13}C NMR (100 MHz, $CDCl_3$)** δ 211.7, 200.0, 88.7, 45.4, 43.4, 42.0, 32.3, 28.9, 27.4, 24.4. The **enantiomeric excess** was determined by chiral HPLC to be 99% ee by using [(*S*,*S*)-**AM13**][2PhCOOH] (Chiralpak® IA-3 column, *n*-Hexane/*i*-PrOH = 95 : 5, 1 mL/min, λ = 205 nm, 25 °C), major enantiomer: t_r = 13.02 min, minor enantiomer: t_r = 14.78 min. **IR ATR (vmax/cm^{-1})**, 2932, 2861, 1699, 1356, 1154. **HRMS (ESI)** Cald for $C_{12}H_{17}ClO_3Na$ $[M+Na]^+$ 267.0758, Found 267.0762**.**

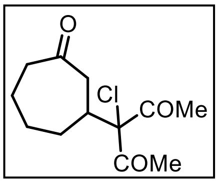

3-Chloro-3-(3-oxocycloheptyl)pentane-2,4-dione (+)-**10b.** Purified by column chromatography (12% EtOAc in PE to 20% EtOAc in PE). Colorless oil (166 mg, 69% yield). $[\alpha]_D^{20}$ = + 8.66 (c 1.0, CH_2Cl_2). The **enantiomeric excess** was determined by chiral HPLC to be 99% ee by using [(*R*,*R*)-**AM13**][2PhCOOH] (Chiralpak® IA-3 column, *n*-Hexane/*i*-PrOH = 95 : 5, 1 mL/min, λ = 205 nm, 25 °C), minor enantiomer: t_r = 13.20 min, major enantiomer: t_r = 14.60 min.

11. Chiral HPLC and chiral GC chromatograms for the asymmetric epoxidations

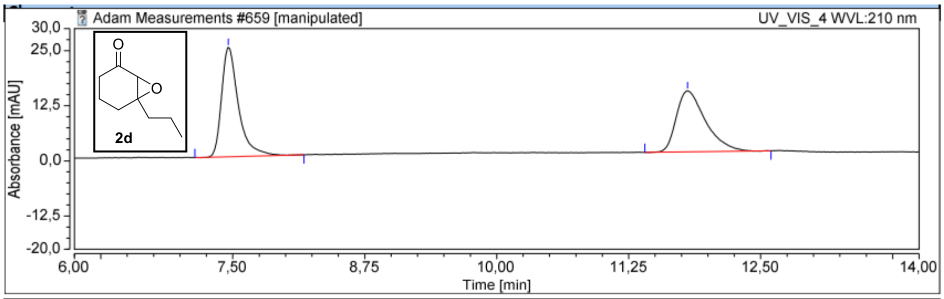

Integration Results

No.	Peak Name	Retention Time min	Area mAU*min	Height mAU	Relative Area %	Relative Height %	Amount n.a.
1		7,458	4,595	24,824	51,04	64,18	n.a.
2		11,807	4,408	13,857	48,96	35,82	n.a.
Total:			9,003	38,681	100,00	100,00	

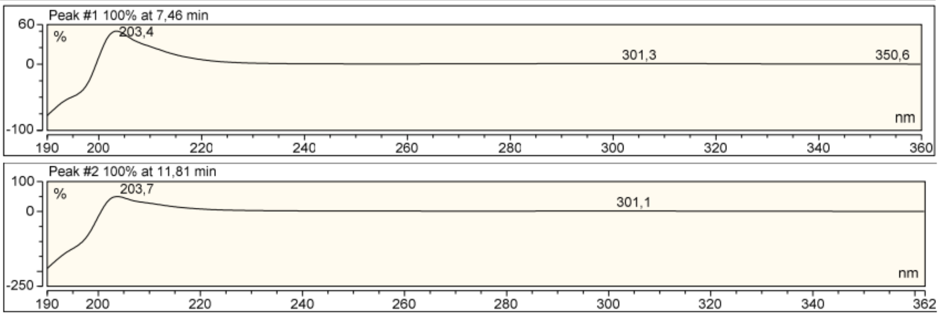

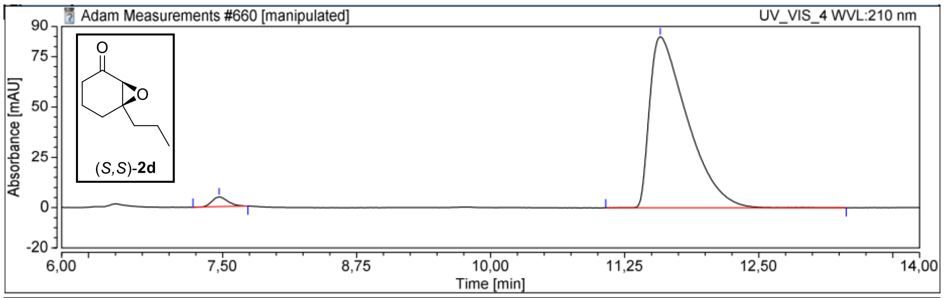

Integration Results

No.	Peak Name	Retention Time min	Area mAU*min	Height mAU	Relative Area %	Relative Height %	Amount n.a.
1		7,468	0,826	4,837	2,34	5,39	n.a.
2		11,582	34,524	84,956	97,66	94,61	n.a.
Total:			35,350	89,793	100,00	100,00	

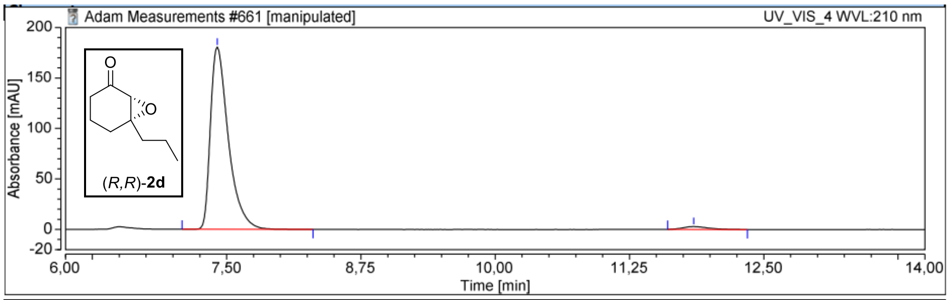

Integration Results

No.	Peak Name	Retention Time min	Area mAU*min	Height mAU	Relative Area %	Relative Height %	Amount n.a.
1		7,412	34,883	180,338	97,84	98,45	n.a.
2		11,848	0,771	2,837	2,16	1,55	n.a.
Total:			35,654	183,174	100,00	100,00	

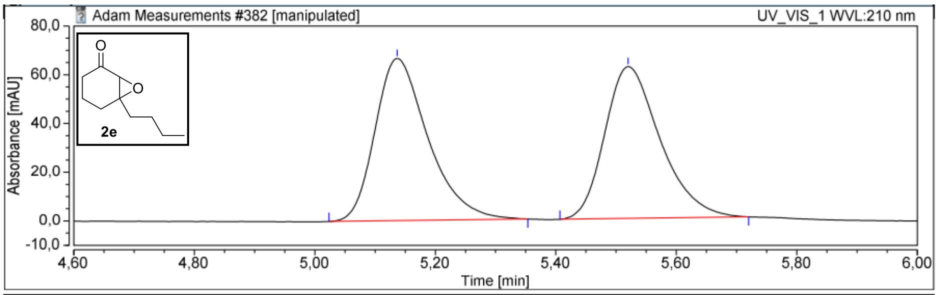

Integration Results

No.	Peak Name	Retention Time min	Area mAU*min	Height mAU	Relative Area %	Relative Height %	Amount n.a.
1		5,137	6,787	66,589	50,55	51,65	n.a.
2		5,520	6,639	62,336	49,45	48,35	n.a.
**Total:**			**13,426**	**128,925**	**100,00**	**100,00**	

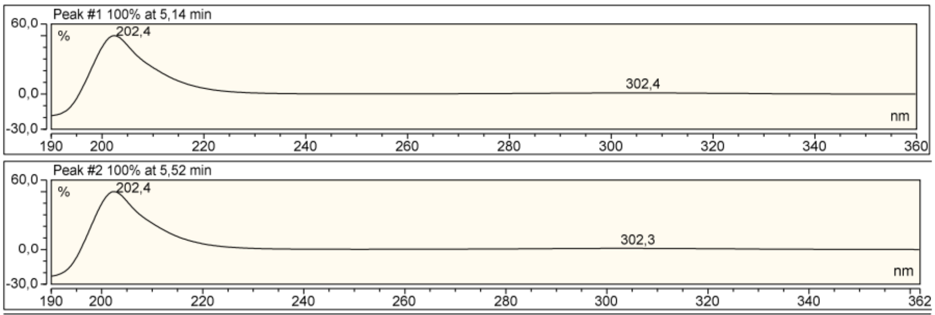

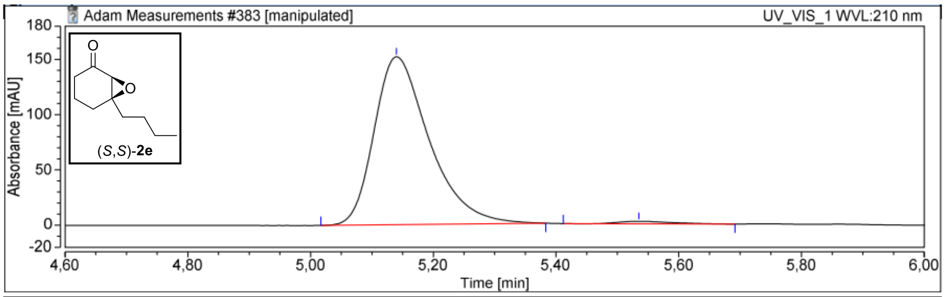

Integration Results

No.	Peak Name	Retention Time min	Area mAU*min	Height mAU	Relative Area %	Relative Height %	Amount n.a.
1		5,140	15,796	151,947	98,64	98,55	n.a.
2		5,535	0,217	2,236	1,36	1,45	n.a.
**Total:**			**16,013**	**154,182**	**100,00**	**100,00**	

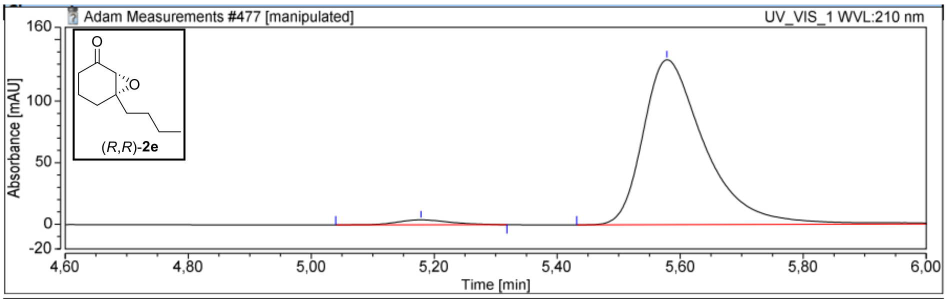

Integration Results

No.	Peak Name	Retention Time min	Area mAU*min	Height mAU	Relative Area %	Relative Height %	Amount n.a.
1		5,178	0,392	4,012	2,44	2,90	n.a.
2		5,578	15,682	134,115	97,56	97,10	n.a.
**Total:**			**16,075**	**138,128**	**100,00**	**100,00**	

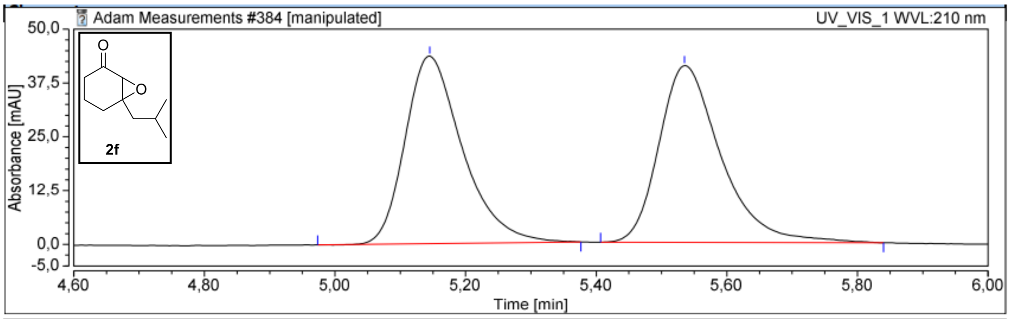

Integration Results							
No.	Peak Name	Retention Time min	Area mAU*min	Height mAU	Relative Area %	Relative Height %	Amount n.a.
1		5,145	4,430	43,569	49,54	51,48	n.a.
2		5,535	4,513	41,069	50,46	48,52	n.a.
**Total:**			**8,943**	**84,638**	**100,00**	**100,00**	

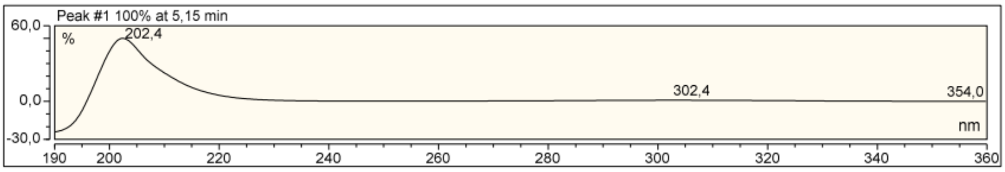

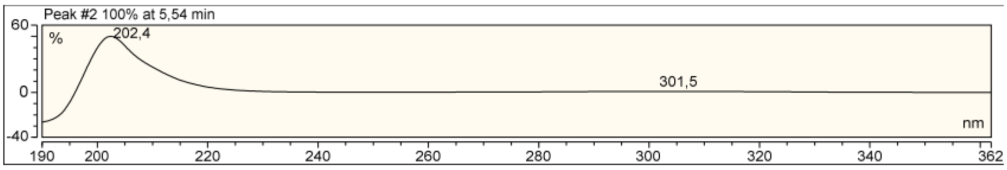

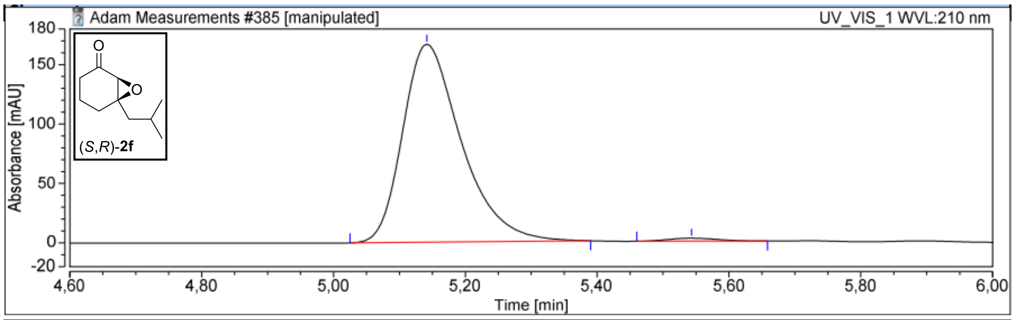

Integration Results							
No.	Peak Name	Retention Time min	Area mAU*min	Height mAU	Relative Area %	Relative Height %	Amount n.a.
1		5,142	17,225	166,593	98,78	98,53	n.a.
2		5,543	0,213	2,477	1,22	1,47	n.a.
**Total:**			**17,438**	**169,070**	**100,00**	**100,00**	

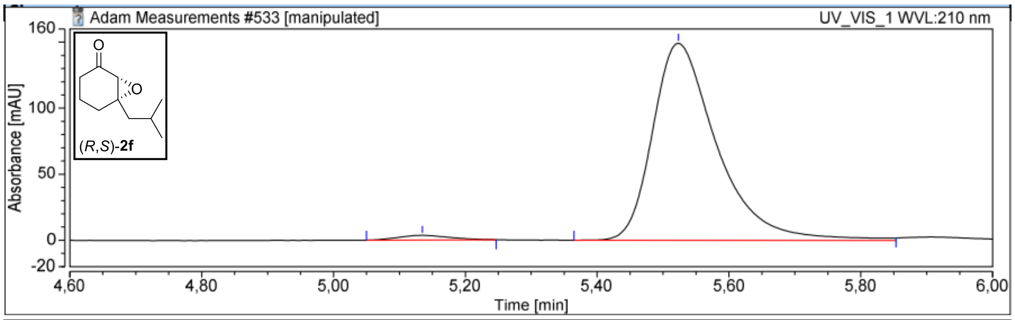

Integration Results							
No.	Peak Name	Retention Time min	Area mAU*min	Height mAU	Relative Area %	Relative Height %	Amount n.a.
1		5,135	0,313	3,488	1,82	2,28	n.a.
2		5,523	16,867	149,230	98,18	97,72	n.a.
**Total:**			**17,180**	**152,718**	**100,00**	**100,00**	

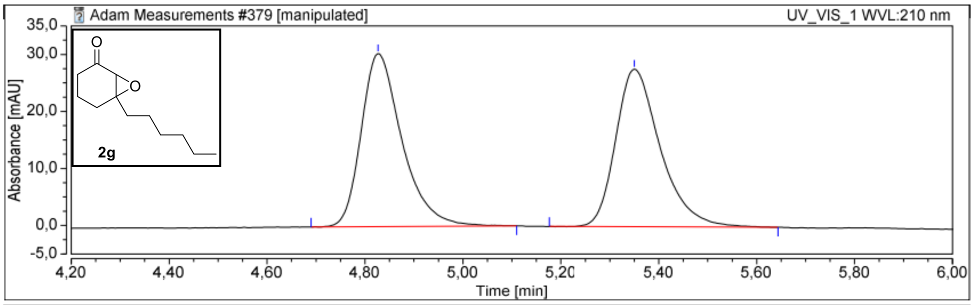

Integration Results							
No.	Peak Name	Retention Time min	Area mAU*min	Height mAU	Relative Area %	Relative Height %	Amount n.a.
1		4,827	2,932	30,374	50,12	52,35	n.a.
2		5,350	2,918	27,647	49,88	47,65	n.a.
Total:			5,851	58,022	100,00	100,00	

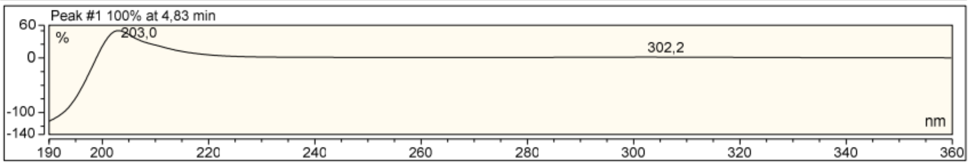

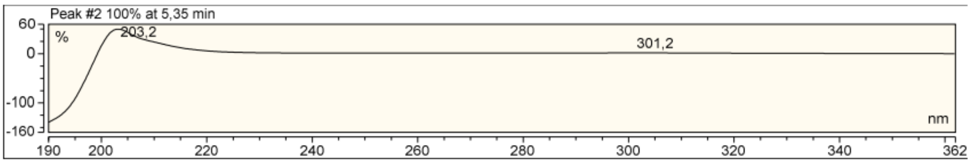

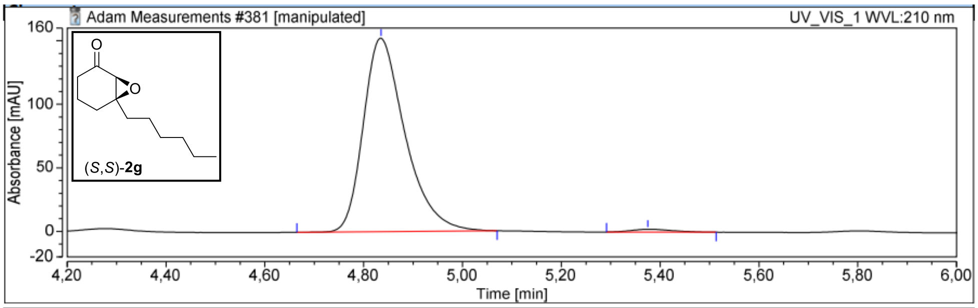

Integration Results							
No.	Peak Name	Retention Time min	Area mAU*min	Height mAU	Relative Area %	Relative Height %	Amount n.a.
1		4,835	14,893	152,260	98,61	98,57	n.a.
2		5,375	0,210	2,202	1,39	1,43	n.a.
Total:			15,103	154,462	100,00	100,00	

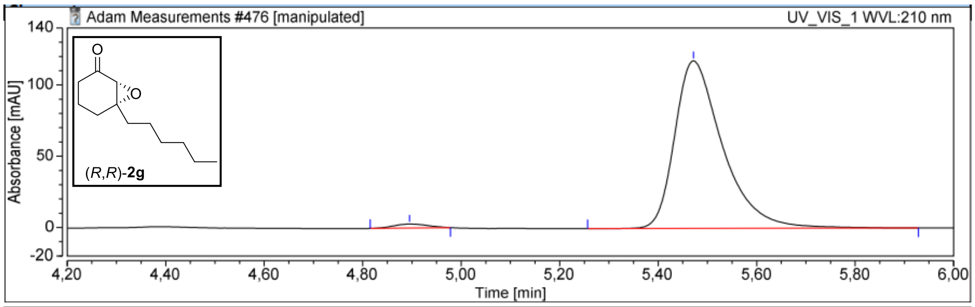

Integration Results							
No.	Peak Name	Retention Time min	Area mAU*min	Height mAU	Relative Area %	Relative Height %	Amount n.a.
1		4,895	0,228	2,842	1,69	2,36	n.a.
2		5,472	13,273	117,672	98,31	97,64	n.a.
Total:			13,501	120,514	100,00	100,00	

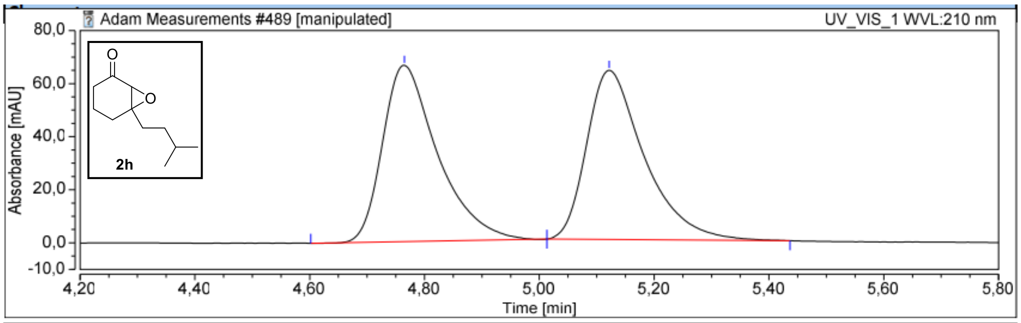

Integration Results							
No.	Peak Name	Retention Time min	Area mAU*min	Height mAU	Relative Area %	Relative Height %	Amount n.a.
1		4,765	7,355	66,478	49,81	51,03	n.a.
2		5,122	7,411	63,786	50,19	48,97	n.a.
**Total:**			**14,766**	**130,264**	**100,00**	**100,00**	

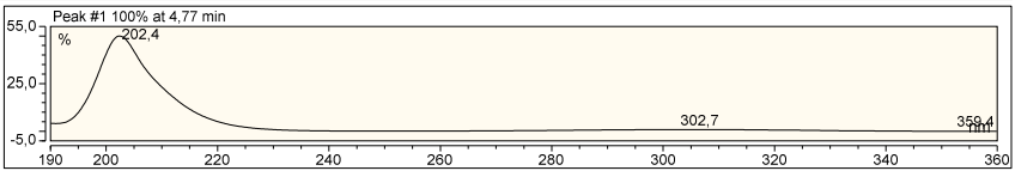

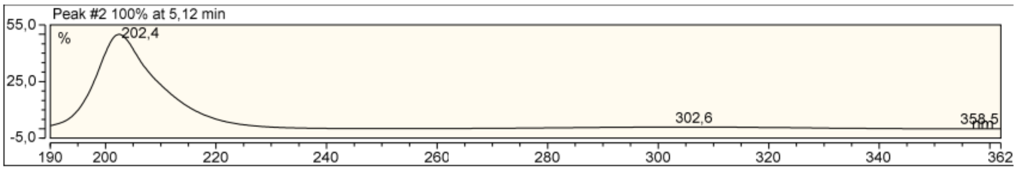

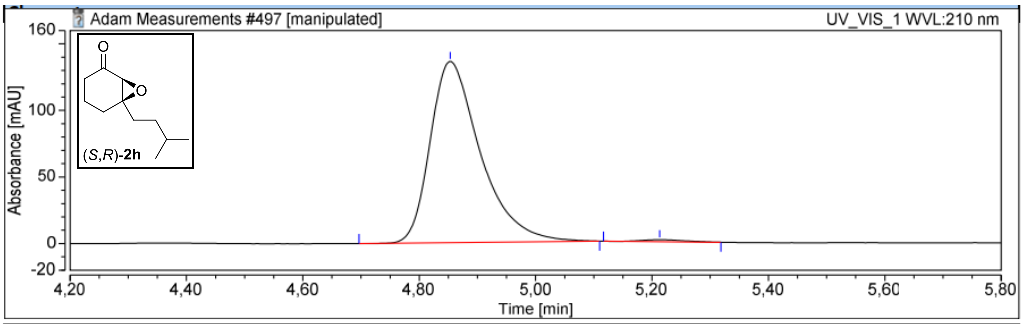

Integration Results							
No.	Peak Name	Retention Time min	Area mAU*min	Height mAU	Relative Area %	Relative Height %	Amount n.a.
1		4,853	13,612	136,137	99,07	98,92	n.a.
2		5,213	0,127	1,487	0,93	1,08	n.a.
**Total:**			**13,739**	**137,623**	**100,00**	**100,00**	

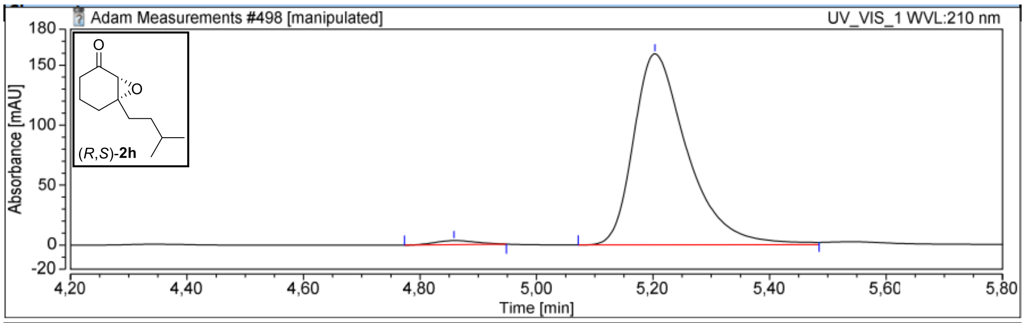

Integration Results							
No.	Peak Name	Retention Time min	Area mAU*min	Height mAU	Relative Area %	Relative Height %	Amount n.a.
1		4,858	0,273	3,372	1,57	2,07	n.a.
2		5,203	17,064	159,392	98,43	97,93	n.a.
**Total:**			**17,336**	**162,764**	**100,00**	**100,00**	

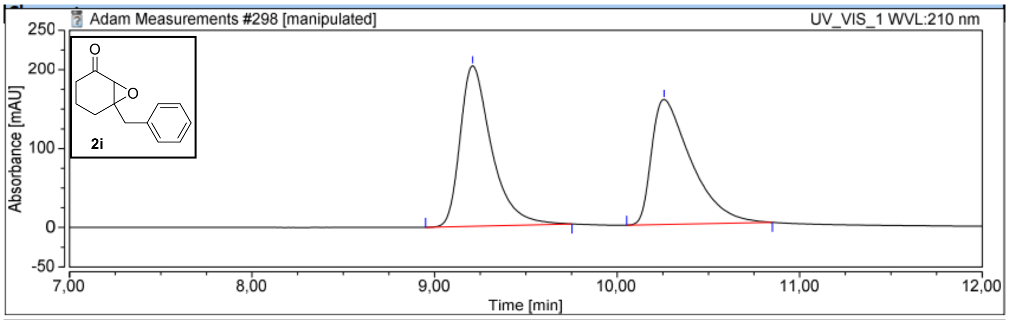

Integration Results

No.	Peak Name	Retention Time min	Area mAU*min	Height mAU	Relative Area %	Relative Height %	Amount n.a.
1		9,208	40,527	203,644	50,41	56,22	n.a.
2		10,257	39,869	158,596	49,59	43,78	n.a.
**Total:**			**80,396**	**362,240**	**100,00**	**100,00**	

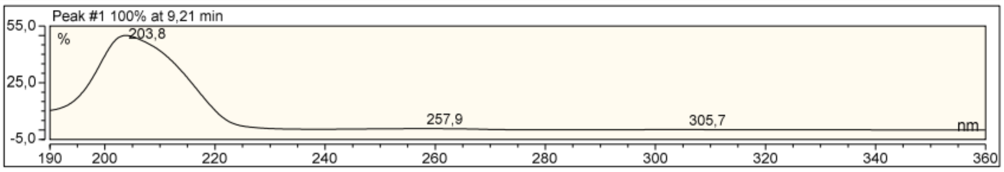

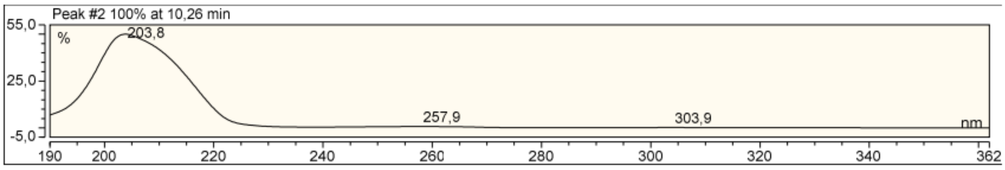

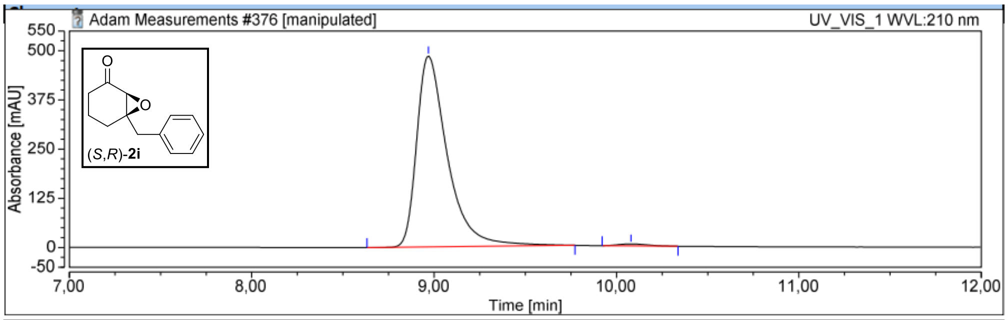

Integration Results

No.	Peak Name	Retention Time min	Area mAU*min	Height mAU	Relative Area %	Relative Height %	Amount n.a.
1		8,968	94,483	484,939	99,03	98,98	n.a.
2		10,080	0,921	4,991	0,97	1,02	n.a.
**Total:**			**95,404**	**489,930**	**100,00**	**100,00**	

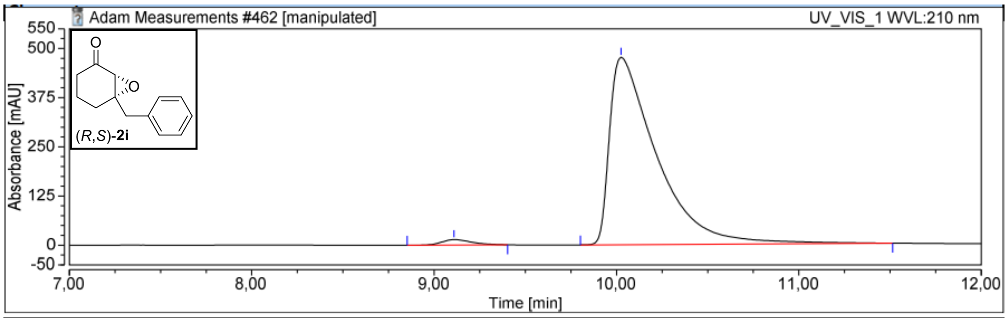

Integration Results

No.	Peak Name	Retention Time min	Area mAU*min	Height mAU	Relative Area %	Relative Height %	Amount n.a.
1		9,108	2,599	13,727	1,79	2,80	n.a.
2		10,025	142,665	476,291	98,21	97,20	n.a.
**Total:**			**145,264**	**490,017**	**100,00**	**100,00**	

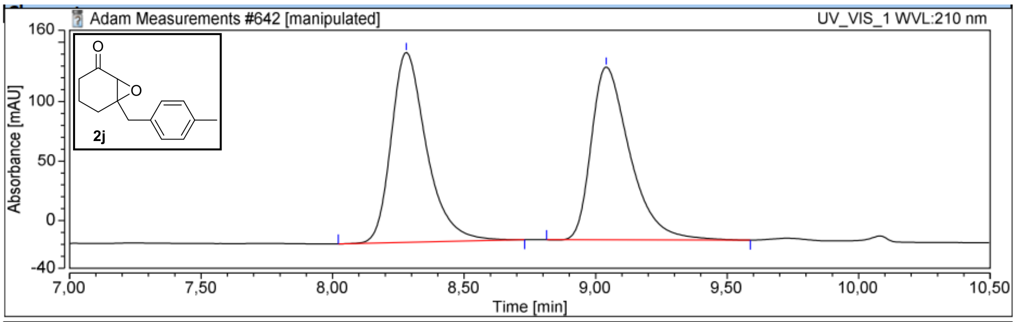

Integration Results							
No.	Peak Name	Retention Time min	Area mAU*min	Height mAU	Relative Area %	Relative Height %	Amount n.a.
1		8,280	24,897	159,683	50,16	52,38	n.a.
2		9,040	24,737	145,187	49,84	47,62	n.a.
Total:			49,634	304,870	100,00	100,00	

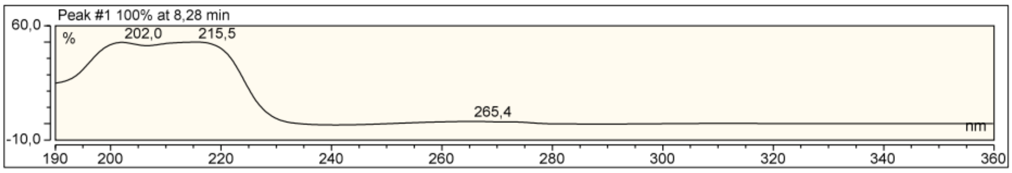

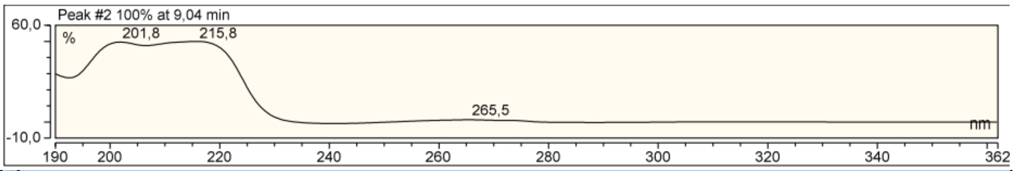

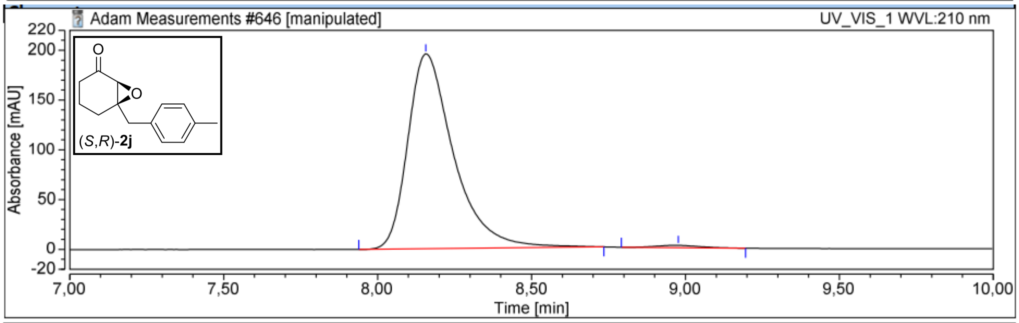

Integration Results							
No.	Peak Name	Retention Time min	Area mAU*min	Height mAU	Relative Area %	Relative Height %	Amount n.a.
1		8,157	33,838	195,881	98,91	98,84	n.a.
2		8,977	0,372	2,301	1,09	1,16	n.a.
Total:			34,210	198,181	100,00	100,00	

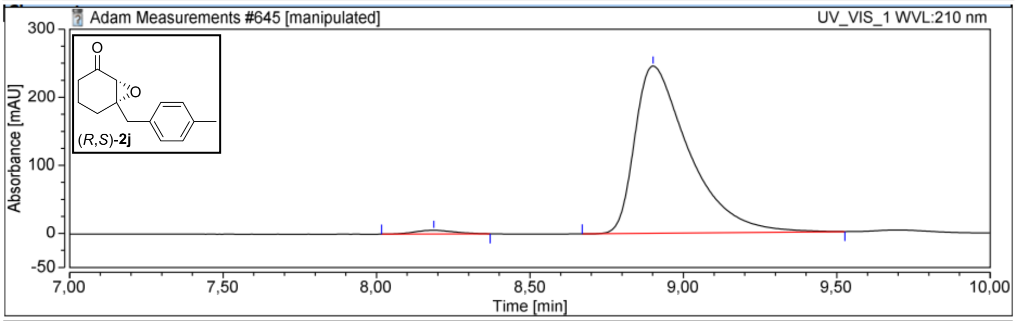

Integration Results							
No.	Peak Name	Retention Time min	Area mAU*min	Height mAU	Relative Area %	Relative Height %	Amount n.a.
1		8,187	0,804	5,406	1,57	2,15	n.a.
2		8,902	50,376	245,891	98,43	97,85	n.a.
Total:			51,179	251,297	100,00	100,00	

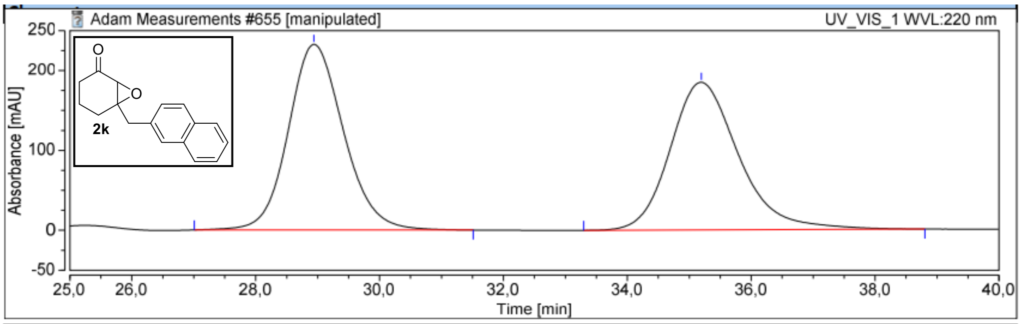

Integration Results							
No.	Peak Name	Retention Time min	Area mAU*min	Height mAU	Relative Area %	Relative Height %	Amount n.a.
1		28,938	240,597	232,494	50,36	55,71	n.a.
2		35,195	237,191	184,809	49,64	44,29	n.a.
**Total:**			**477,788**	**417,303**	**100,00**	**100,00**	

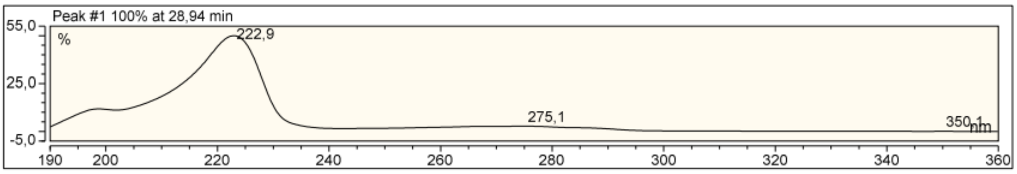

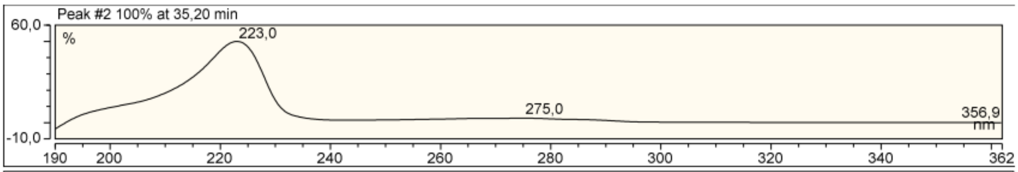

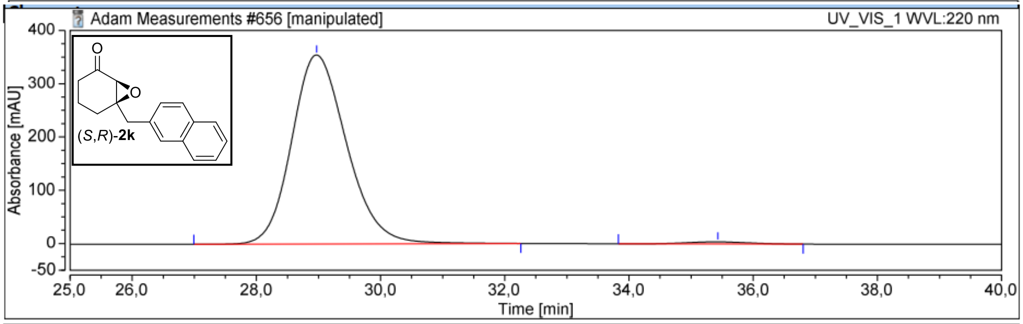

Integration Results							
No.	Peak Name	Retention Time min	Area mAU*min	Height mAU	Relative Area %	Relative Height %	Amount n.a.
1		28,968	363,984	355,021	98,76	98,90	n.a.
2		35,430	4,569	3,950	1,24	1,10	n.a.
**Total:**			**368,553**	**358,971**	**100,00**	**100,00**	

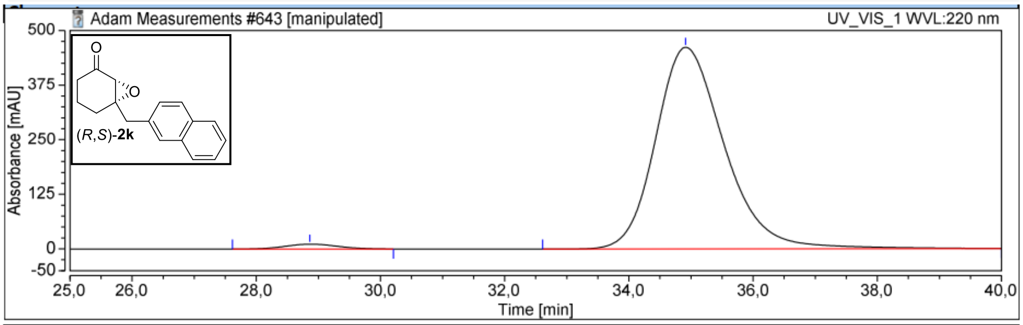

Integration Results							
No.	Peak Name	Retention Time min	Area mAU*min	Height mAU	Relative Area %	Relative Height %	Amount n.a.
1		28,863	11,190	11,318	1,85	2,39	n.a.
2		34,912	592,545	461,747	98,15	97,61	n.a.
**Total:**			**603,735**	**473,065**	**100,00**	**100,00**	

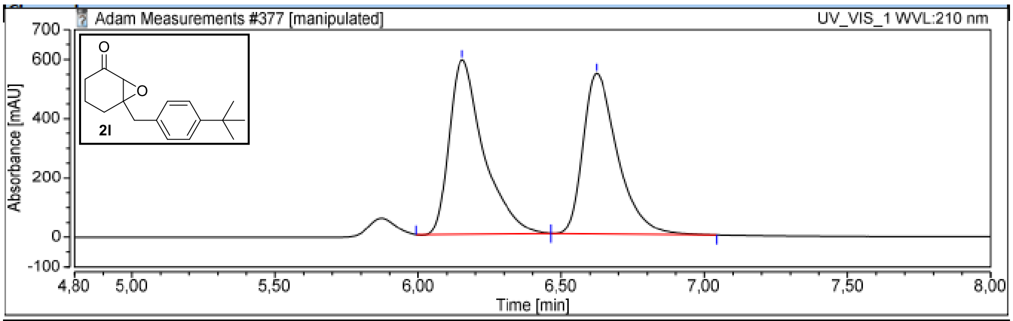

Integration Results							
No.	Peak Name	Retention Time min	Area mAU*min	Height mAU	Relative Area %	Relative Height %	Amount n.a.
1		6,153	81,787	589,335	51,87	52,03	n.a.
2		6,623	75,882	543,317	48,13	47,97	n.a.
Total:			157,669	1132,652	100,00	100,00	

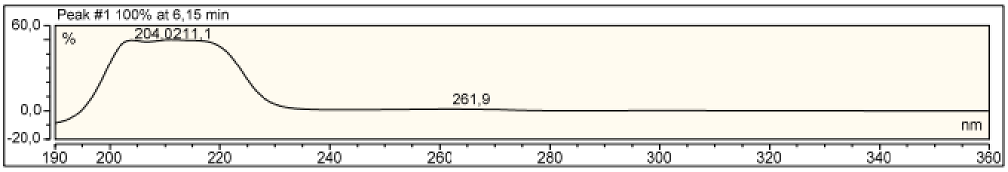

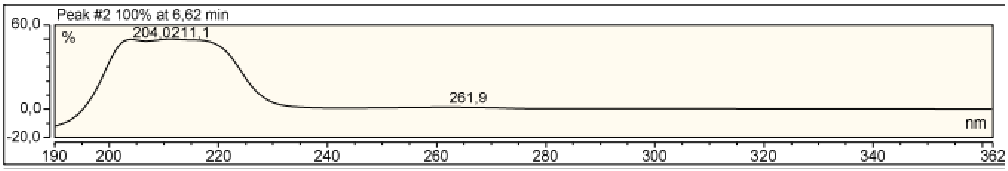

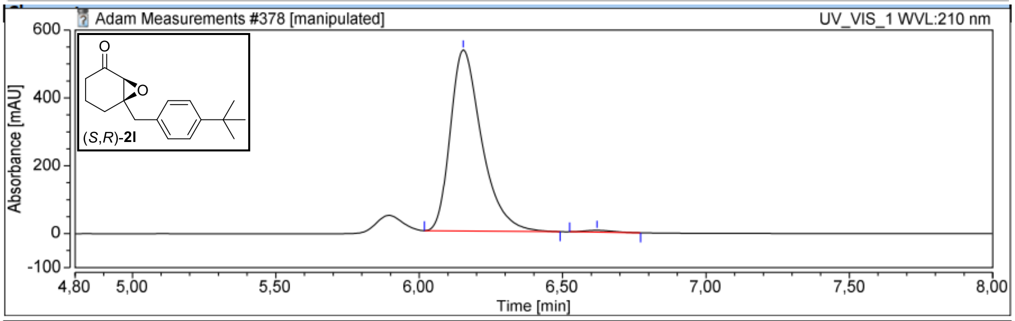

Integration Results							
No.	Peak Name	Retention Time min	Area mAU*min	Height mAU	Relative Area %	Relative Height %	Amount n.a.
1		6,153	67,757	533,581	99,09	98,97	n.a.
2		6,620	0,623	5,530	0,91	1,03	n.a.
Total:			68,380	539,111	100,00	100,00	

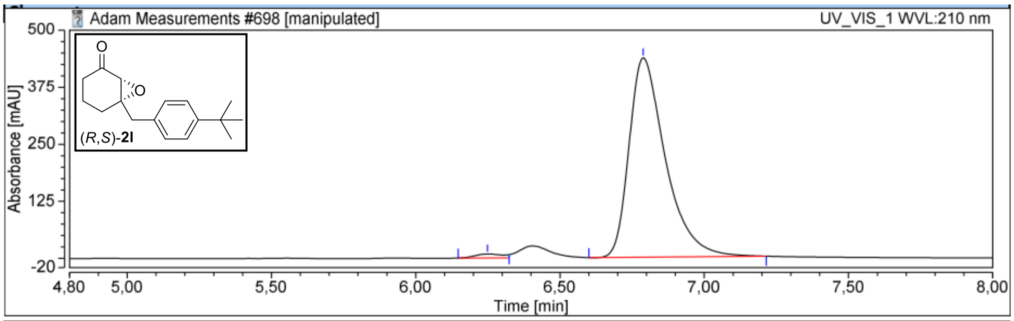

Integration Results							
No.	Peak Name	Retention Time min	Area mAU*min	Height mAU	Relative Area %	Relative Height %	Amount n.a.
1		6,248	0,965	8,695	1,49	1,95	n.a.
2		6,788	63,685	436,689	98,51	98,05	n.a.
Total:			64,651	445,384	100,00	100,00	

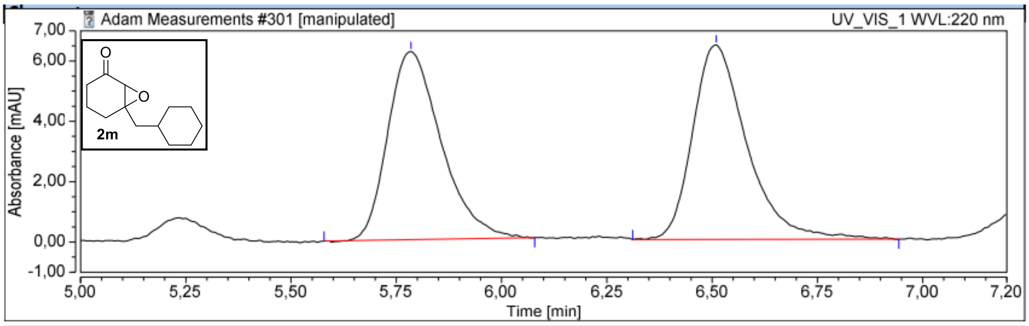

Integration Results

No.	Peak Name	Retention Time min	Area mAU*min	Height mAU	Relative Area %	Relative Height %	Amount n.a.
1		5,785	0,915	6,236	48,69	49,18	n.a.
2		6,510	0,965	6,444	51,31	50,82	n.a.
**Total:**			**1,880**	**12,680**	**100,00**	**100,00**	

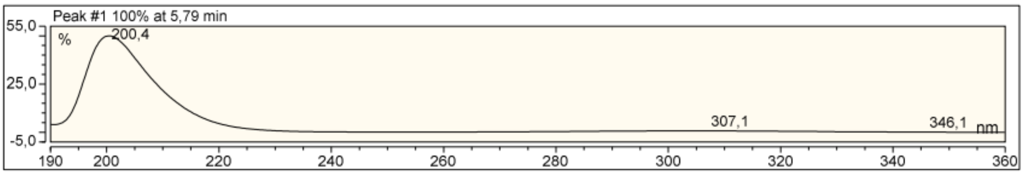

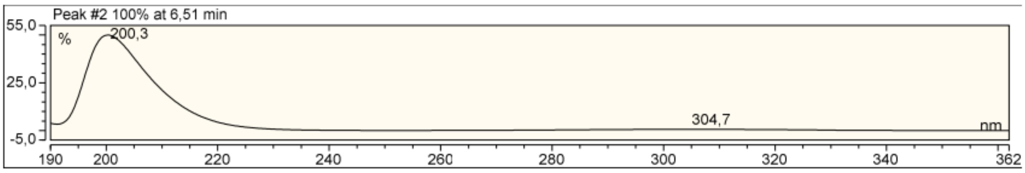

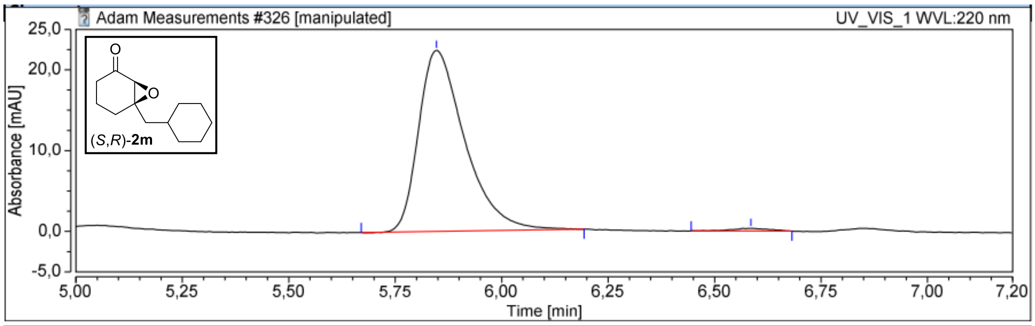

Integration Results

No.	Peak Name	Retention Time min	Area mAU*min	Height mAU	Relative Area %	Relative Height %	Amount n.a.
1		5,847	2,816	22,418	98,83	98,52	n.a.
2		6,585	0,033	0,336	1,17	1,48	n.a.
**Total:**			**2,850**	**22,754**	**100,00**	**100,00**	

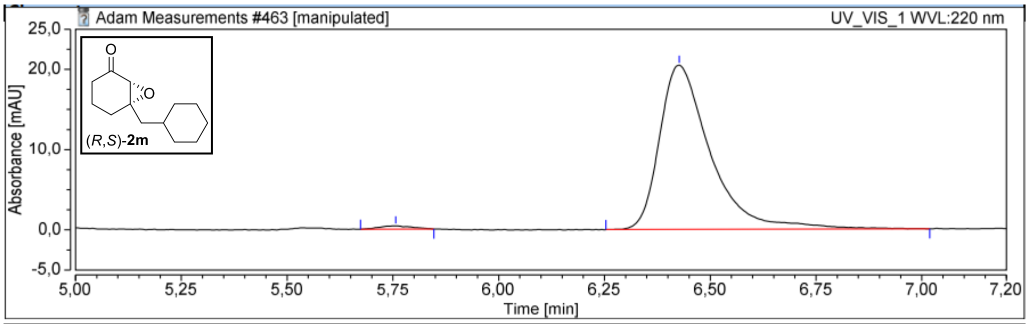

Integration Results

No.	Peak Name	Retention Time min	Area mAU*min	Height mAU	Relative Area %	Relative Height %	Amount n.a.
1		5,757	0,037	0,417	1,29	2,00	n.a.
2		6,427	2,852	20,464	98,71	98,00	n.a.
**Total:**			**2,890**	**20,881**	**100,00**	**100,00**	

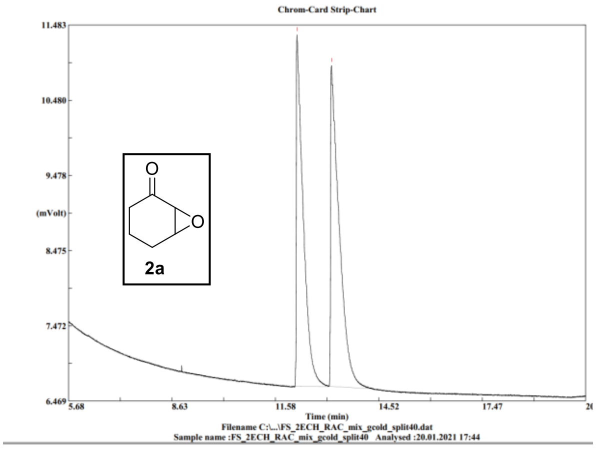

Analysed : 20.01.2021 17:44 Printed : 21.02.2021 14:24
Sample ID : FS_2ECH_RAC_mix_gcold_split40
Channel : Channel A
Analysis Type : UnkNown (Area) Calc. Method : Area %

Peak Number #	Area %	Ret.Time	Area	BC
1	47.3050	12.19	631914	mi
2	52.6950	13.17	703914	mi
Totals	100.0000		1335828	

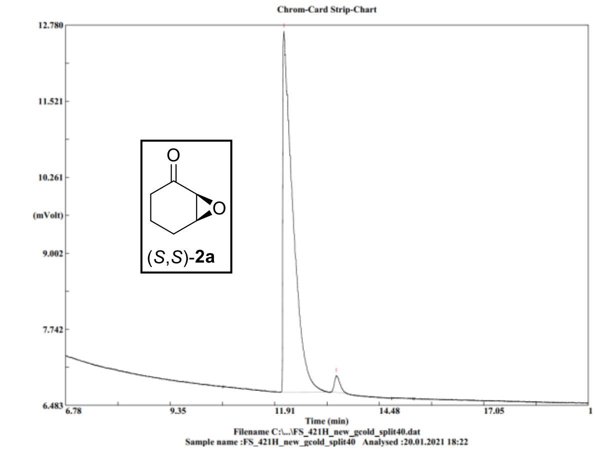

Operator ID : Company Name :
Analysed : 20.01.2021 18:22 Printed : 21.02.2021 14:05
Sample ID : FS_421H_new_gcold_split40
Channel : Channel A
Analysis Type : UnkNown (Area) Calc. Method : Area %

Peak Number #	Area %	Ret.Time	Area	BC
1	97.2837	12.15	1017860	mi
2	2.7163	13.43	28420	mi
Totals	100.0000		1046280	

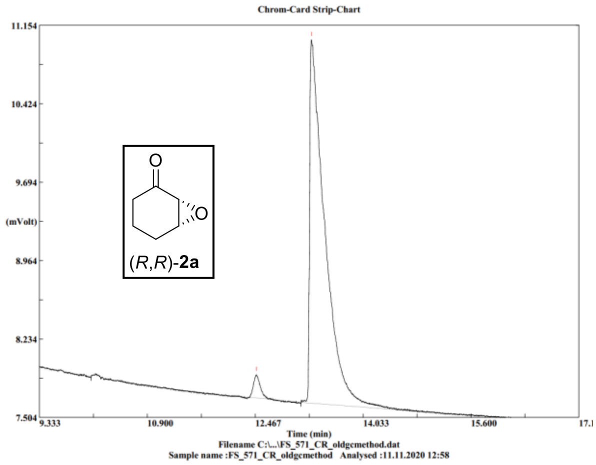

Analysed : 11.11.2020 12:58 Printed : 02.01.2021 14:0
Sample ID : FS_571_CR_oldgcmethod Channel : Channel A
Analysis Type : UnkNown (Area) Calc. Method : Area %

Peak Number #	Area %	Ret.Time	Area	BC
1	2.7457	12.49	14506	mi
2	97.2543	13.29	513798	mi
Totals	100.0000		528304	

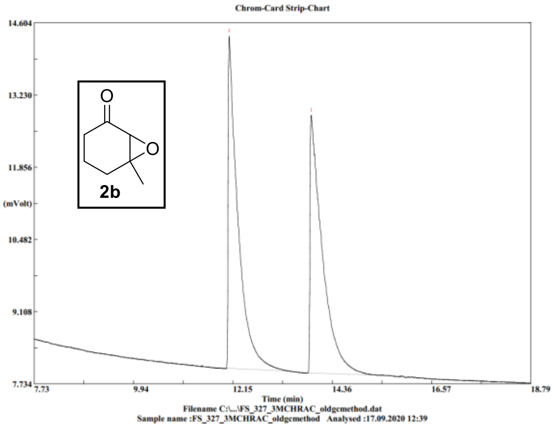

Analysed : 17.09.2020 12:39 Printed : 17.09.2020 13:32
Sample ID : FS_327_3MCHRAC_oldgcmethod
Channel : Channel A
Analysis Type : UnkNown (Area) Calc. Method : Area %

Peak Number #	Area %	Ret.Time	Area	BC
1	50.4849	12.07	944865	mi
2	49.5151	13.90	926713	mi
Totals	100.0000		1871578	

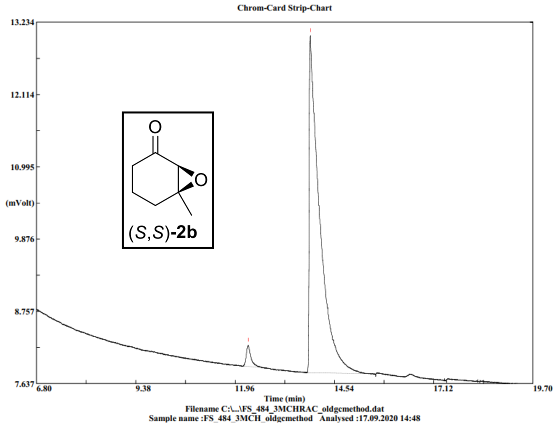

Analysed : 17.09.2020 14:48 Printed : 08.10.2020 16:52
Sample ID : FS_484_3MCH_oldgcmethodChannel : Channel A
Analysis Type : UnkNown (Area) Calc. Method : Area %

Peak Number #	Area %	Ret.Time	Area	BC
1	2.6285	12.30	26364	mi
2	97.3716	13.92	976642	mi
Totals	100.0000		1003006	

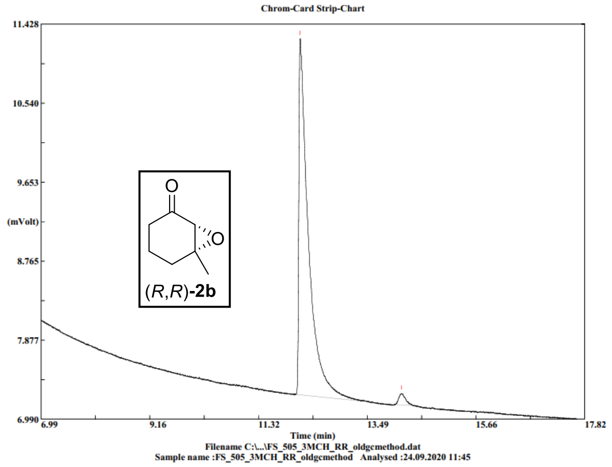

Analysed : 24.09.2020 11:45 Printed : 08.10.2020 16:50
Sample ID : FS_505_3MCH_RR_oldgcmethod
Channel : Channel A
Analysis Type : UnkNown (Area) Calc. Method : Area %

Peak Number #	Area %	Ret.Time	Area	BC
1	97.5301	12.15	527793	mi
2	2.4699	14.17	13366	mi
Totals	100.0000		541159	

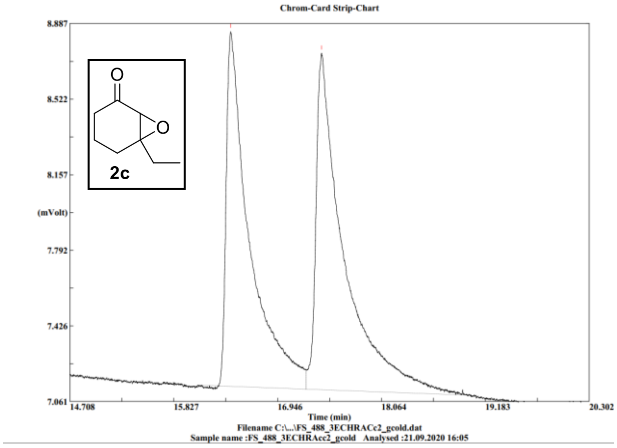

Analysed : 21.09.2020 16:05 Printed : 21.09.2020 16:38
Sample ID : FS_488_3ECHRAcc2_gcold Channel : Channel A
Analysis Type : UnkNown (Area) Calc. Method : Area %

Peak Number #	Area %	Ret.Time	Area	BC
1	47.3646	16.44	297390	mi
2	52.6354	17.42	330484	mi
Totals	100.0000		627874	

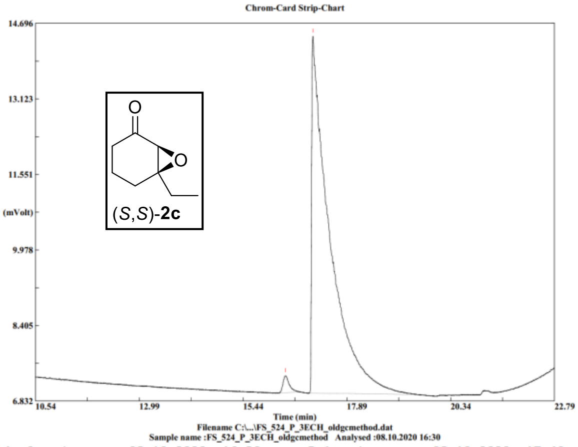

Analysed : 08.10.2020 16:30 Printed : 08.10.2020 17:40
Sample ID : FS_524_P_3ECH_oldgcmethod
Channel : Channel A
Analysis Type : UnkNown (Area) Calc. Method : Area %

Peak Number #	Area %	Ret.Time	Area	BC
1	1.6644	16.44	36903	mi
2	98.3356	17.09	2180270	mi
Totals	100.0000		2217173	

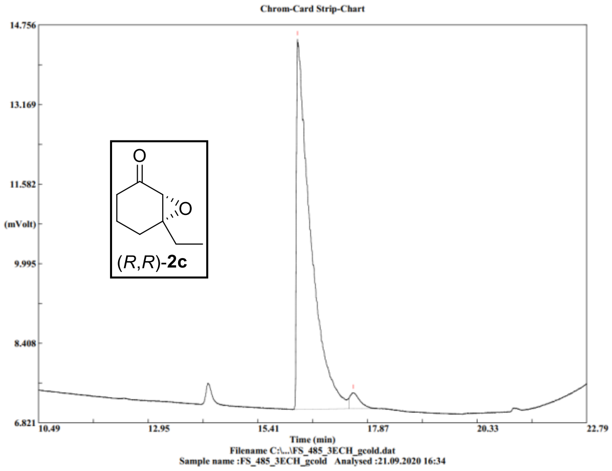

Analysed : 21.09.2020 16:34 Printed : 03.01.2021 13:27
Sample ID : FS_485_3ECH_gcold Channel : Channel A
Analysis Type : UnkNown (Area) Calc. Method : Area %

Peak Number #	Area %	Ret.Time	Area	BC
1	97.0875	16.30	1630936	mi
2	2.9125	17.55	48927	mi
Totals	100.0000		1679862	

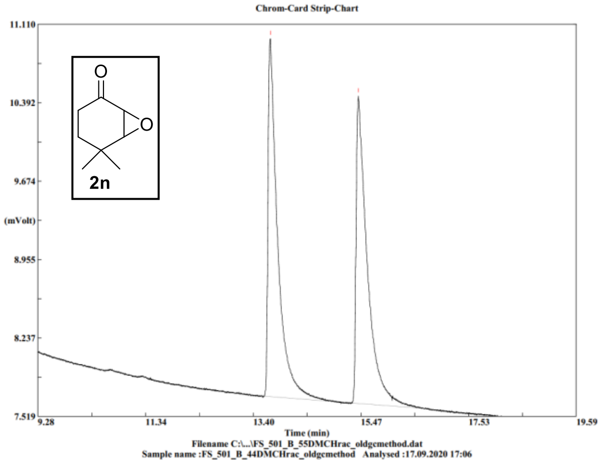

Analysed : 17.09.2020 17:06 Printed : 17.09.2020 20:22
Sample ID : FS_501_B_44DMCHrac_oldgcmethod
Channel : Channel A
Analysis Type : UnkNown (Area) Calc. Method : Area %

Peak Number #	Area %	Ret.Time	Area	BC
1	49.5526	13.73	409531	mi
2	50.4474	15.42	416926	mi
Totals	100.0000		826457	

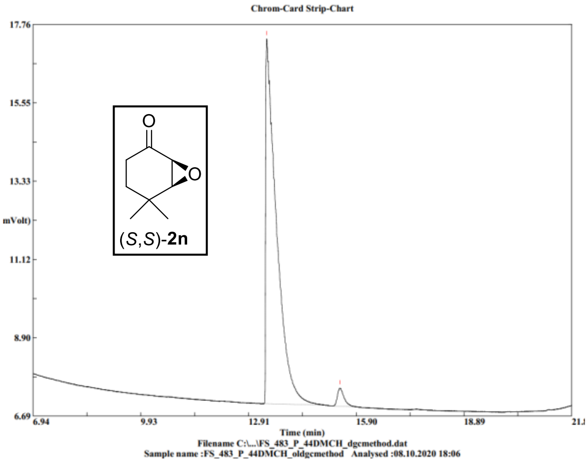

Analysed : 08.10.2020 18:06 Printed : 08.10.2020 22:37
Sample ID : FS_483_P_44DMCH_oldgcmethod
Channel : Channel A
Analysis Type : UnkNown (Area) Calc. Method : Area %

Peak Number #	Area %	Ret.Time	Area	BC
1	97.1759	13.42	2140601	mi
2	2.8241	15.45	62209	mi
Totals	100.0000		2202810	

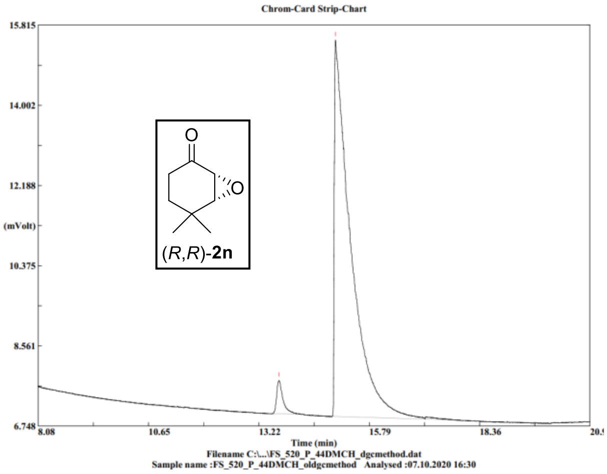

Analysed : 07.10.2020 16:30 Printed : 08.10.2020 16:55
Sample ID : FS_520_P_44DMCH_oldgcmethod
Channel : Channel A
Analysis Type : UnkNown (Area) Calc. Method : Area %

Peak Number #	Area %	Ret.Time	Area	BC
1	3.2889	13.69	75117	mi
2	96.7111	15.00	2208821	mi
Totals	100.0000		2283938	

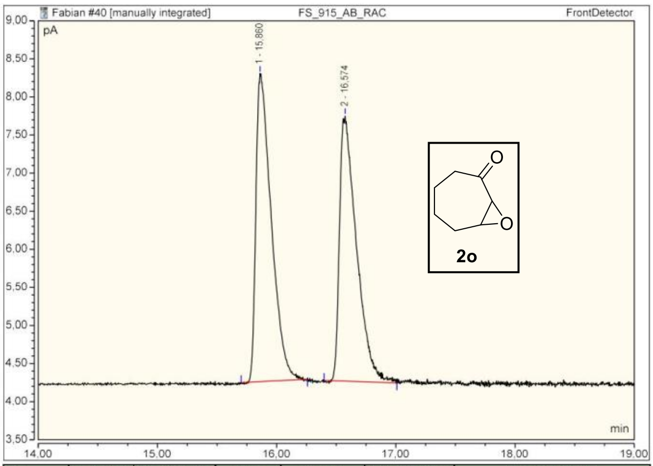

No.	Peak Name	Retention Time min	Area pA*min	Height pA	Relative Area %	Relative Height %
1		15,860	0,602	4,046	51,29	53,74
2		16,574	0,572	3,483	48,71	46,26
**Total:**			**1,174**	**7,530**	**100,00**	**100,00**

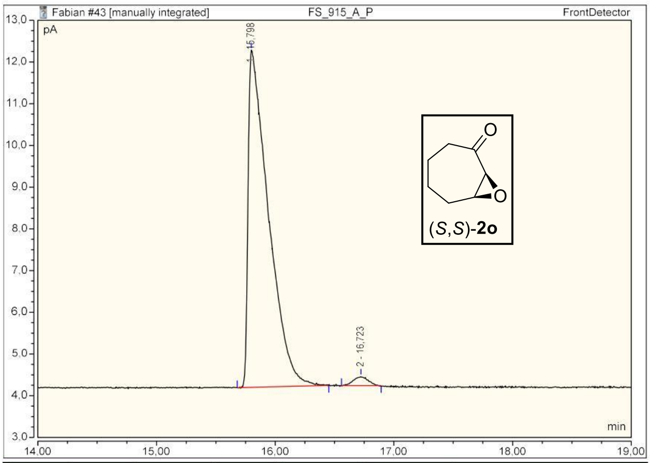

No.	Peak Name	Retention Time min	Area pA*min	Height pA	Relative Area %	Relative Height %
1		15,798	1,593	8,088	98,16	97,33
2		16,723	0,030	0,222	1,84	2,67
**Total:**			**1,623**	**8,310**	**100,00**	**100,00**

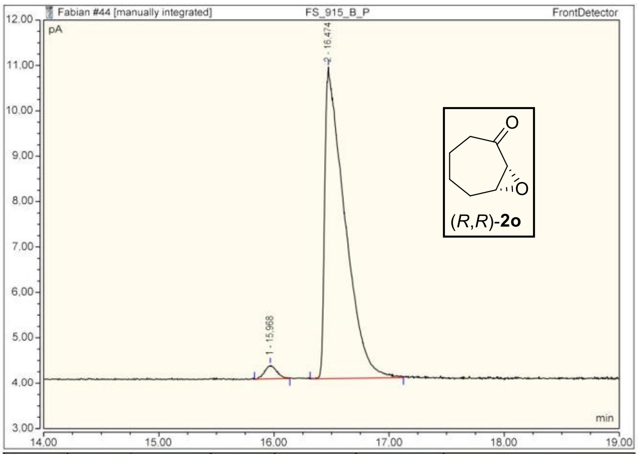

No.	Peak Name	Retention Time min	Area pA*min	Height pA	Relative Area %	Relative Height %
1		15,968	0,036	0,307	2,54	4,27
2		16,474	1,376	6,866	97,46	95,73
**Total:**			**1,411**	**7,172**	**100,00**	**100,00**

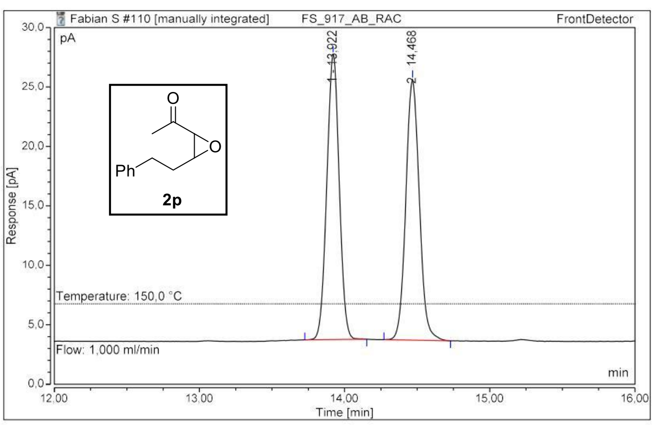

No.	Peak Name	Retention Time min	Area pA*min	Height pA	elative Are %	elative Heig %	Amount n.a.
1		13,922	2,383	24,005	50,19	52,25	n.a.
2		14,468	2,365	21,937	49,81	47,75	n.a.
Total:			4,749	45,942	100,00	100,00	

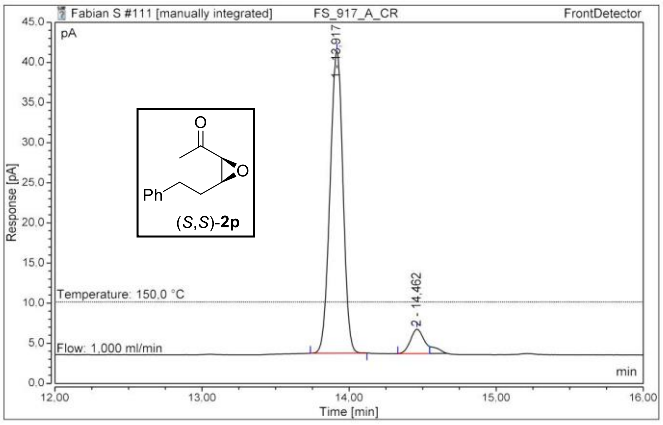

No.	Peak Name	Retention Time min	Area pA*min	Height pA	elative Are %	elative Heig %	Amount n.a.
1		13,917	3,847	37,673	92,30	92,49	n.a.
2		14,462	0,321	3,058	7,70	7,51	n.a.
Total:			4,168	40,732	100,00	100,00	

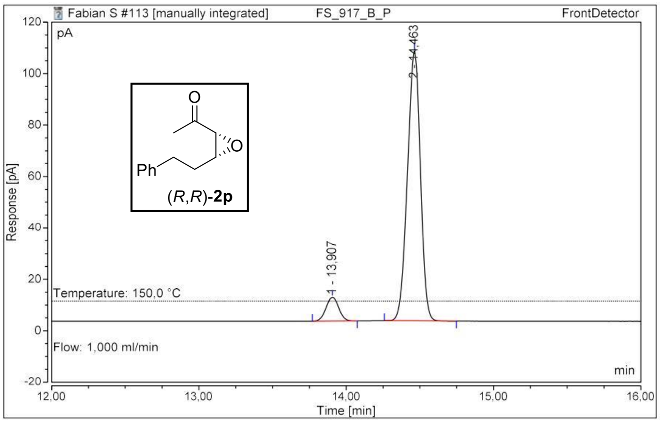

No.	Peak Name	Retention Time min	Area pA*min	Height pA	elative Are %	elative Heig %	Amount n.a.
1		13,907	0,922	9,262	7,94	8,11	n.a.
2		14,463	10,683	104,973	92,06	91,89	n.a.
Total:			11,605	114,235	100,00	100,00	

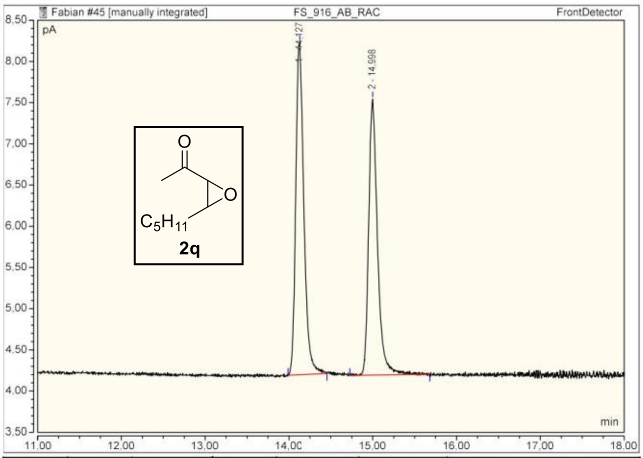

No.	Peak Name	Retention Time min	Area pA*min	Height pA	Relative Area %	Relative Height %
1		14,127	0,453	4,037	52,54	54,66
2		14,998	0,409	3,349	47,46	45,34
**Total:**			**0,862**	**7,387**	**100,00**	**100,00**

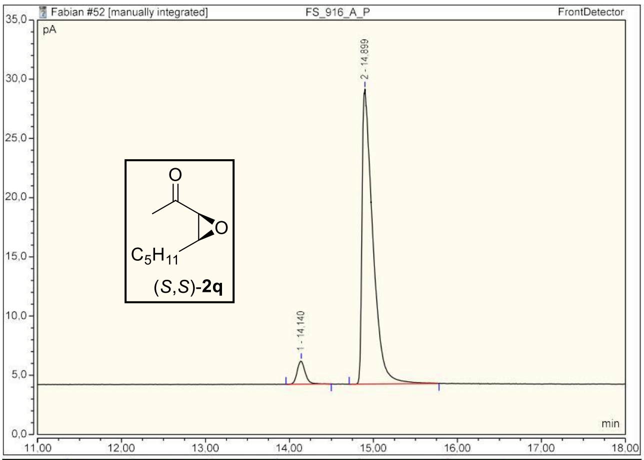

No.	Peak Name	Retention Time min	Area pA*min	Height pA	Relative Area %	Relative Height %
1		14,140	0,215	1,998	5,48	7,42
2		14,899	3,708	24,919	94,52	92,58
**Total:**			**3,923**	**26,917**	**100,00**	**100,00**

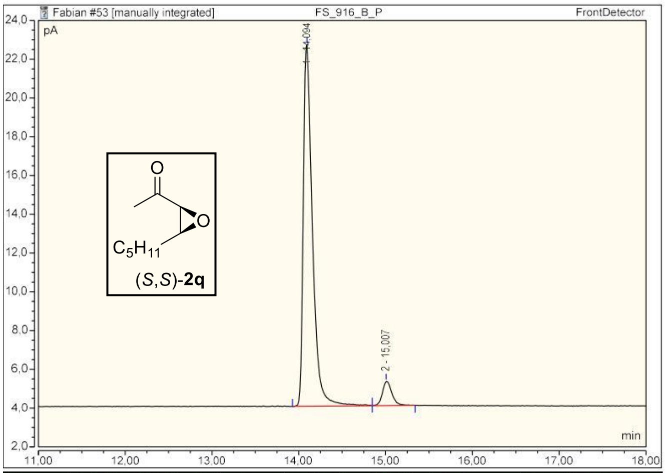

No.	Peak Name	Retention Time min	Area pA*min	Height pA	Relative Area %	Relative Height %
1		14,094	2,276	18,662	93,47	93,69
2		15,007	0,159	1,258	6,53	6,31
**Total:**			**2,435**	**19,920**	**100,00**	**100,00**

12. Chiral HPLC chromatograms for the asymmetric aziridinations

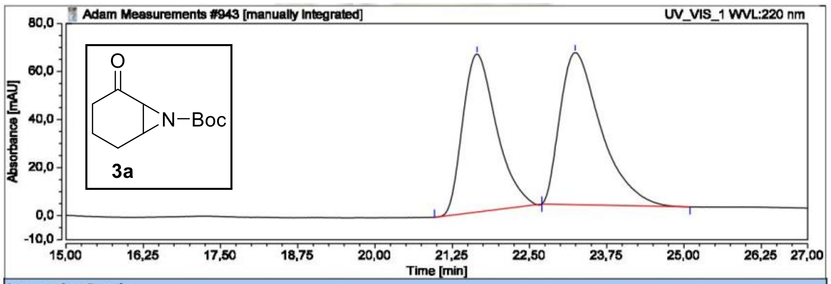

Integration Results

No.	Peak Name	Retention Time min	Area mAU*min	Height mAU	Relative Area %	Relative Height %	Amount n.a.
1		21,652	41,589	65,703	46,00	50,93	n.a.
2		23,238	48,818	63,311	54,00	49,07	n.a.
Total:			90,407	129,013	100,00	100,00	

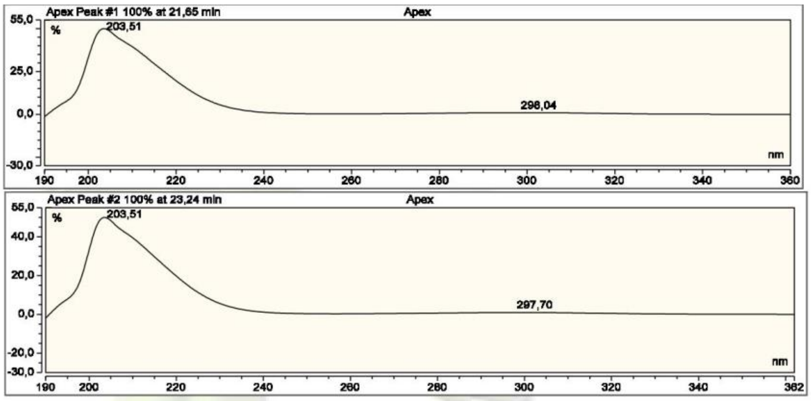

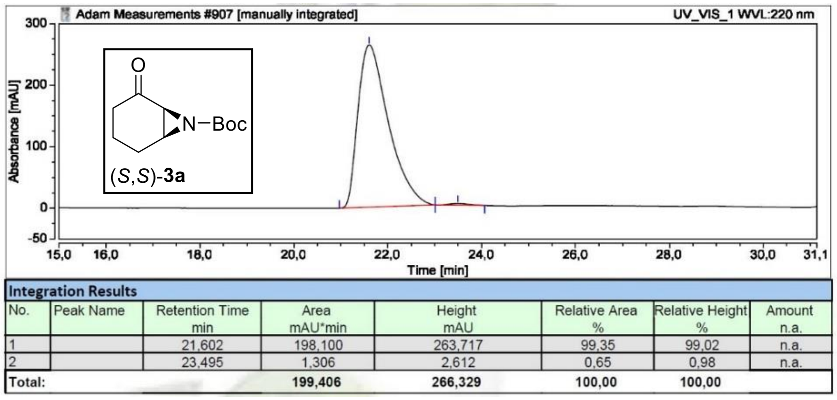

Integration Results

No.	Peak Name	Retention Time min	Area mAU*min	Height mAU	Relative Area %	Relative Height %	Amount n.a.
1		21,602	198,100	263,717	99,35	99,02	n.a.
2		23,495	1,306	2,612	0,65	0,98	n.a.
Total:			199,406	266,329	100,00	100,00	

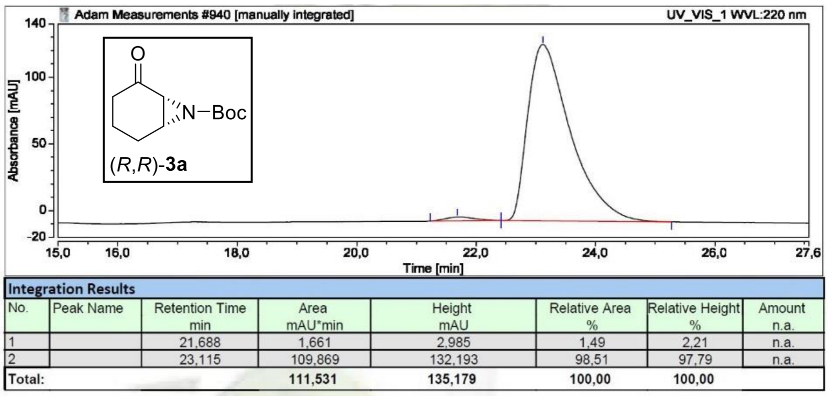

Integration Results

No.	Peak Name	Retention Time min	Area mAU*min	Height mAU	Relative Area %	Relative Height %	Amount n.a.
1		21,688	1,661	2,985	1,49	2,21	n.a.
2		23,115	109,869	132,193	98,51	97,79	n.a.
Total:			111,531	135,179	100,00	100,00	

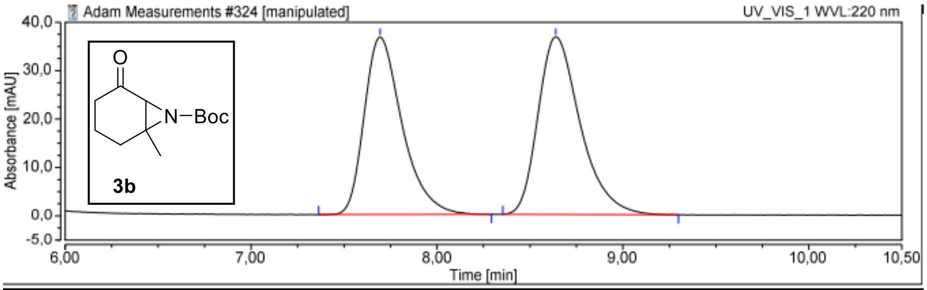

Integration Results

No.	Peak Name	Retention Time min	Area mAU*min	Height mAU	Relative Area %	Relative Height %	Amount n.a.
1		7,693	8,662	36,652	47,23	49,99	n.a.
2		8,638	9,678	36,661	52,77	50,01	n.a.
Total:			18,340	73,313	100,00	100,00	

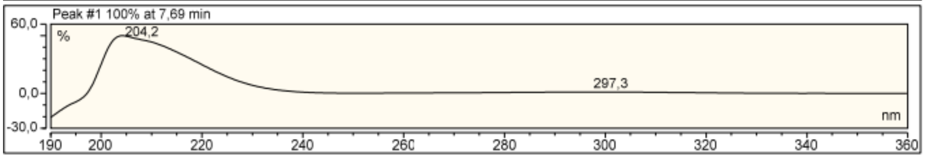

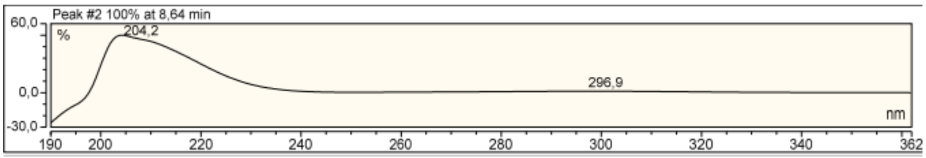

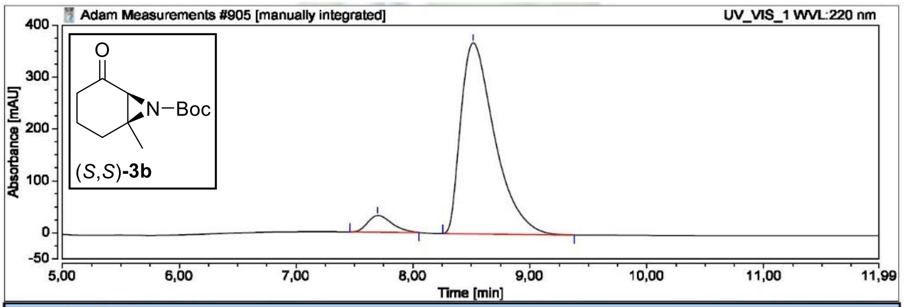

Integration Results

No.	Peak Name	Retention Time min	Area mAU*min	Height mAU	Relative Area %	Relative Height %	Amount n.a.
1		7,700	7,735	32,028	5,89	8,01	n.a.
2		8,515	123,616	367,736	94,11	91,99	n.a.
Total:			131,351	399,764	100,00	100,00	

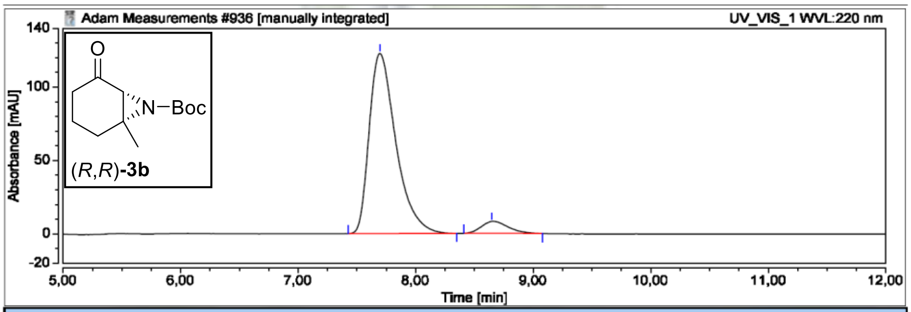

Integration Results

No.	Peak Name	Retention Time min	Area mAU*min	Height mAU	Relative Area %	Relative Height %	Amount n.a.
1		7,695	32,247	122,738	93,76	93,77	n.a.
2		8,650	2,145	8,157	6,24	6,23	n.a.
Total:			34,392	130,894	100,00	100,00	

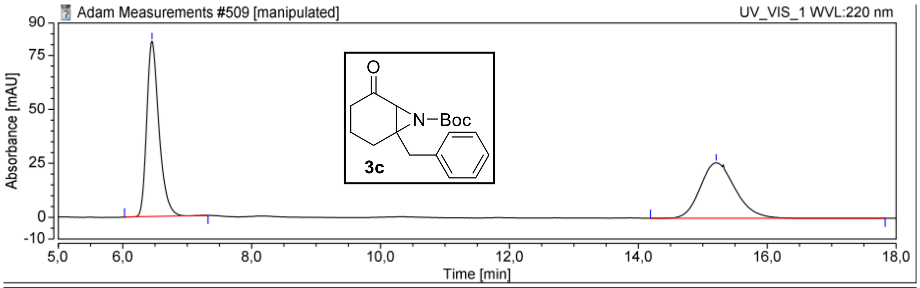

Integration Results

No.	Peak Name	Retention Time min	Area mAU*min	Height mAU	Relative Area %	Relative Height %	Amount n.a.
1		6,452	17,893	81,063	52,73	75,96	n.a.
2		15,207	16,041	25,661	47,27	24,04	n.a.
**Total:**			**33,934**	**106,724**	**100,00**	**100,00**	

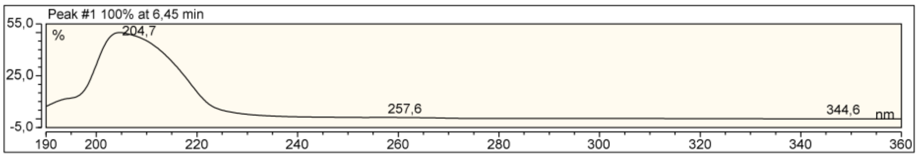

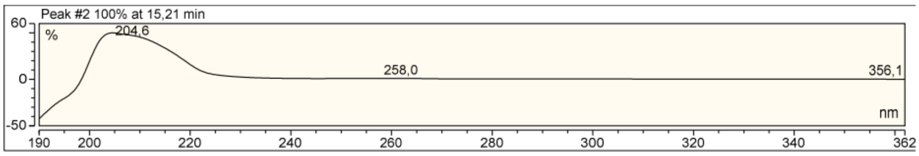

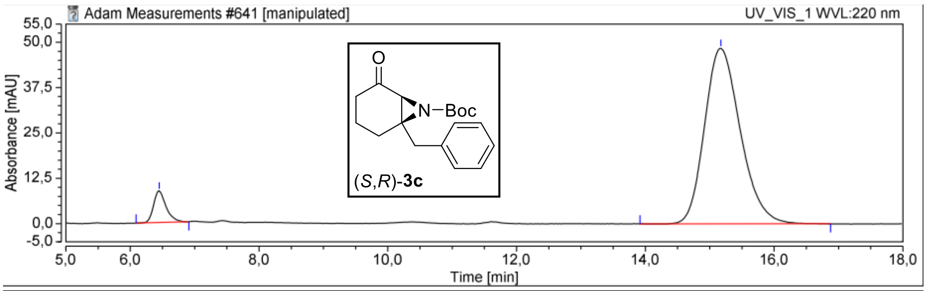

Integration Results

No.	Peak Name	Retention Time min	Area mAU*min	Height mAU	Relative Area %	Relative Height %	Amount n.a.
1		6,448	1,949	8,700	5,89	15,24	n.a.
2		15,172	31,151	48,379	94,11	84,76	n.a.
**Total:**			**33,099**	**57,079**	**100,00**	**100,00**	

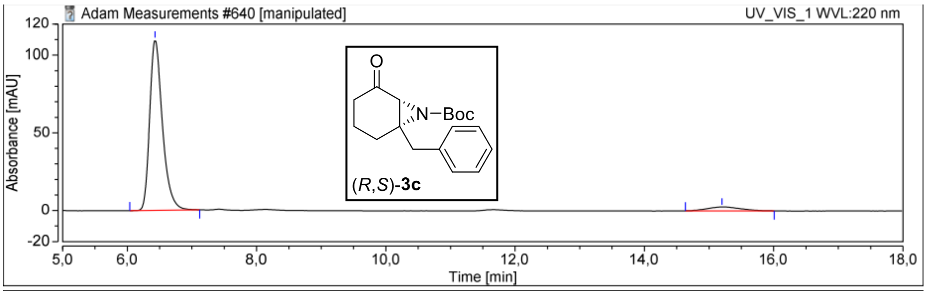

Integration Results

No.	Peak Name	Retention Time min	Area mAU*min	Height mAU	Relative Area %	Relative Height %	Amount n.a.
1		6,430	25,076	109,691	94,45	97,79	n.a.
2		15,200	1,474	2,476	5,55	2,21	n.a.
**Total:**			**26,550**	**112,167**	**100,00**	**100,00**	

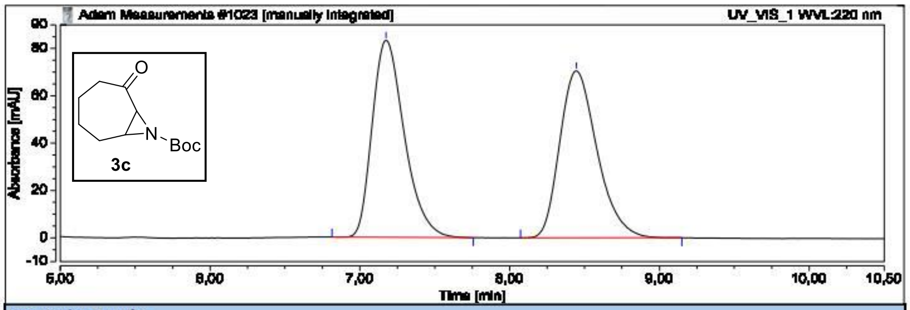

Integration Results							
No.	Peak Name	Retention Time min	Area mAU*min	Height mAU	Relative Area %	Relative Height %	Amount n.a.
1		7,175	20,561	83,238	50,24	54,11	n.a.
2		8,445	20,362	70,580	49,76	45,89	n.a.
Total:			40,923	153,818	100,00	100,00	

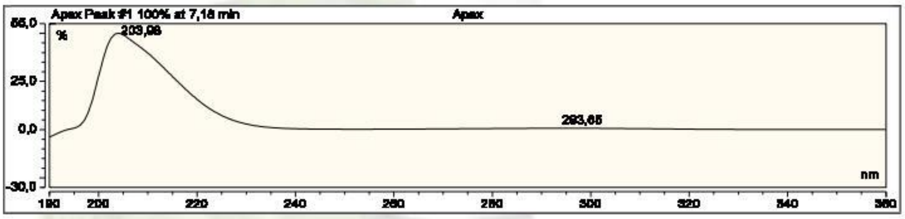

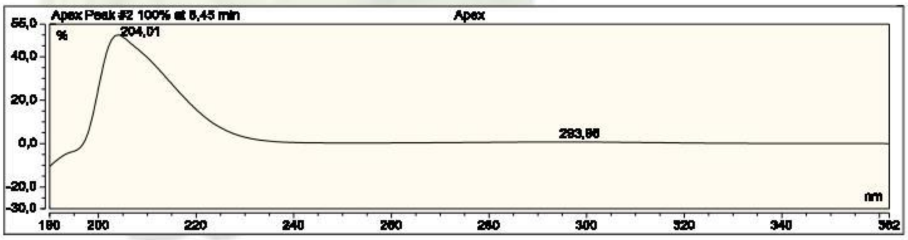

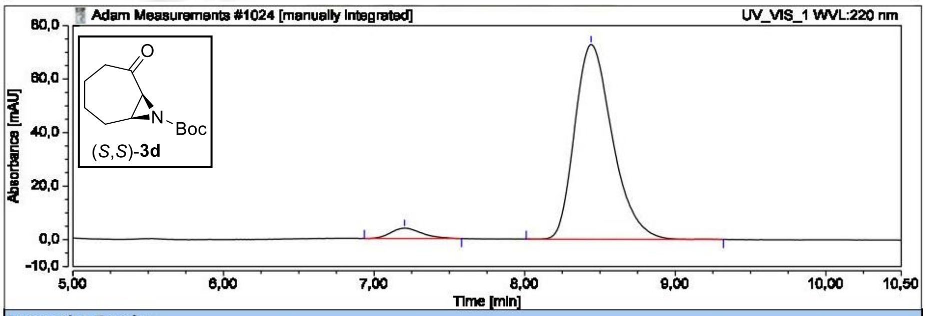

Integration Results							
No.	Peak Name	Retention Time min	Area mAU*min	Height mAU	Relative Area %	Relative Height %	Amount n.a.
1		7,202	0,934	3,886	4,23	5,07	n.a.
2		8,442	21,137	72,797	95,77	94,93	n.a.
Total:			22,071	76,683	100,00	100,00	

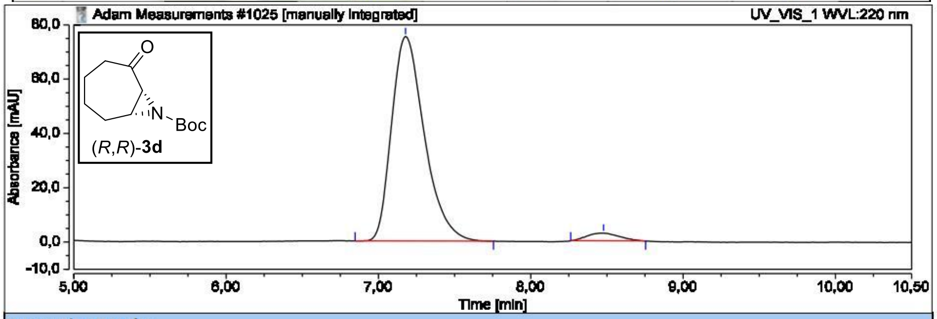

Integration Results							
No.	Peak Name	Retention Time min	Area mAU*min	Height mAU	Relative Area %	Relative Height %	Amount n.a.
1		7,178	18,762	75,330	96,36	96,32	n.a.
2		8,478	0,708	2,881	3,64	3,68	n.a.
Total:			19,470	78,211	100,00	100,00	

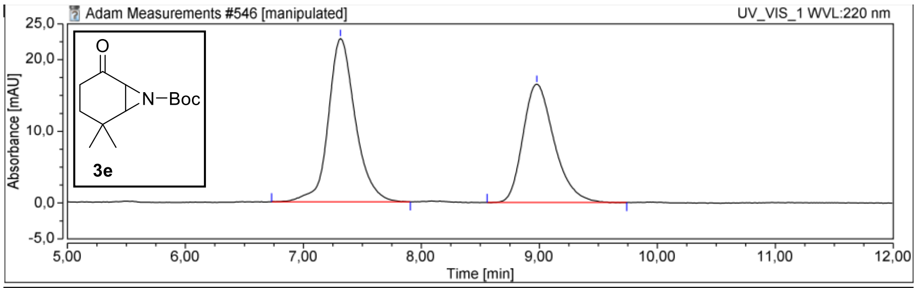

Integration Results							
No.	Peak Name	Retention Time min	Area mAU*min	Height mAU	Relative Area %	Relative Height %	Amount n.a.
1		7,313	6,044	22,816	54,42	58,01	n.a.
2		8,978	5,062	16,513	45,58	41,99	n.a.
Total:			11,107	39,329	100,00	100,00	

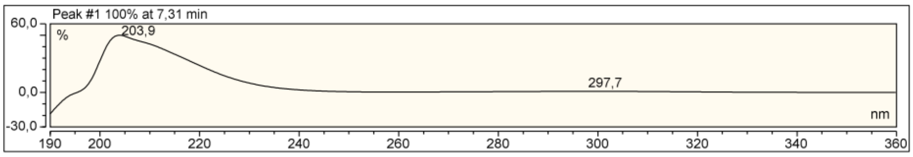

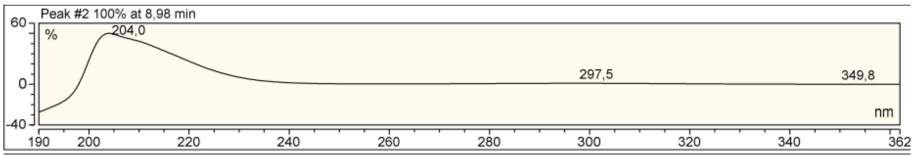

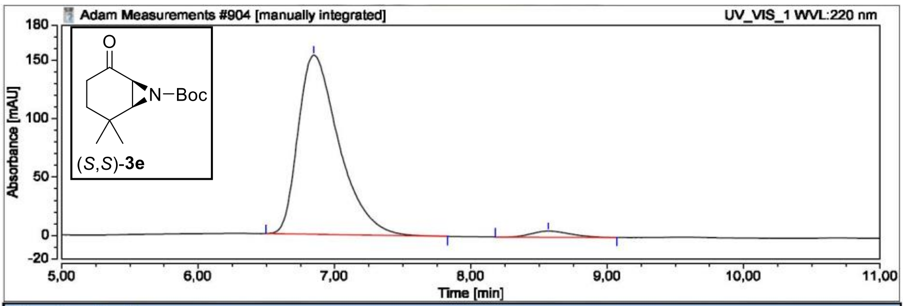

Integration Results							
No.	Peak Name	Retention Time min	Area mAU*min	Height mAU	Relative Area %	Relative Height %	Amount n.a.
1		6,848	53,232	153,311	96,81	96,65	n.a.
2		8,567	1,754	5,307	3,19	3,35	n.a.
Total:			54,987	158,618	100,00	100,00	

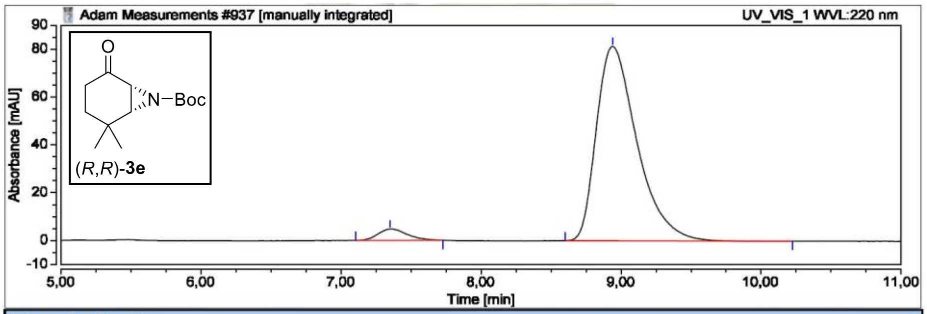

Integration Results							
No.	Peak Name	Retention Time min	Area mAU*min	Height mAU	Relative Area %	Relative Height %	Amount n.a.
1		7,352	1,157	4,802	4,07	5,58	n.a.
2		8,938	27,260	81,282	95,93	94,42	n.a.
Total:			28,417	86,083	100,00	100,00	

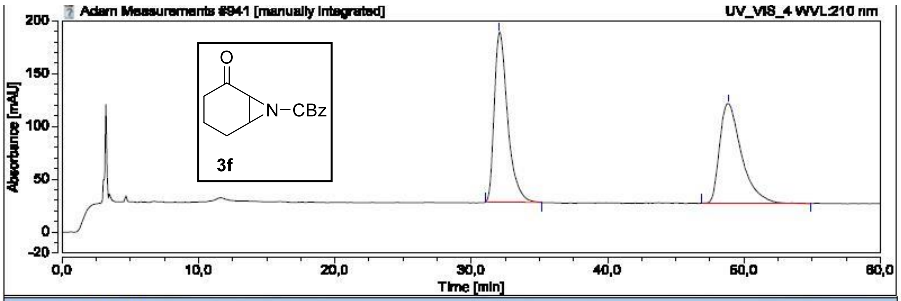

Integration Results							
No.	Peak Name	Retention Time min	Area mAU*min	Height mAU	Relative Area %	Relative Height %	Amount n.a.
1		32,075	182,240	161,429	51,08	63,08	n.a.
2		48,833	174,535	94,465	48,92	36,92	n.a.
Total:			356,775	255,894	100,00	100,00	

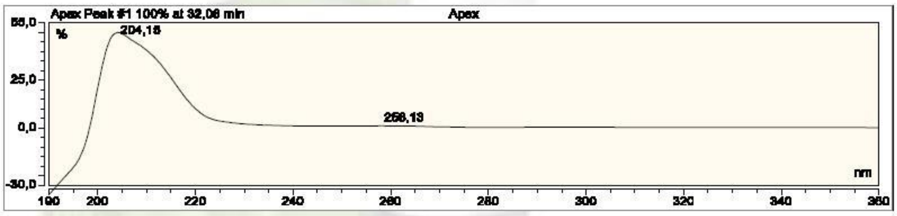

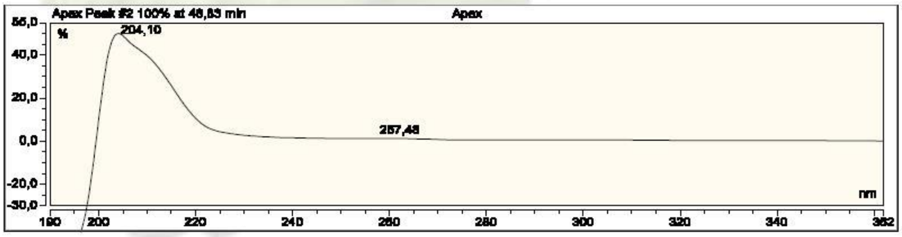

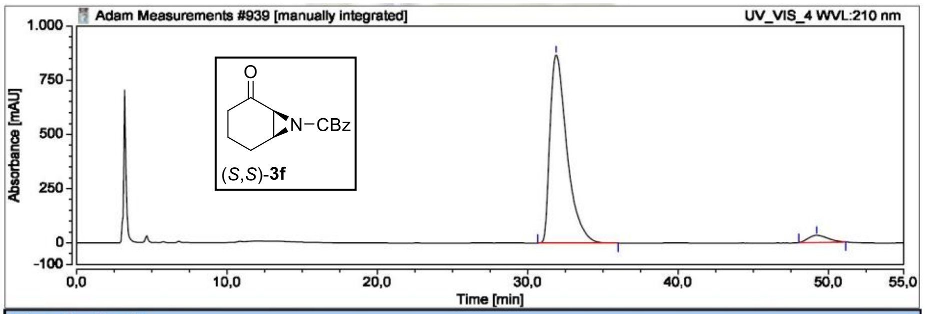

Integration Results							
No.	Peak Name	Retention Time min	Area mAU*min	Height mAU	Relative Area %	Relative Height %	Amount n.a.
1		31,887	1131,020	868,181	95,85	96,39	n.a.
2		49,230	48,947	32,518	4,15	3,61	n.a.
Total:			1179,967	900,699	100,00	100,00	

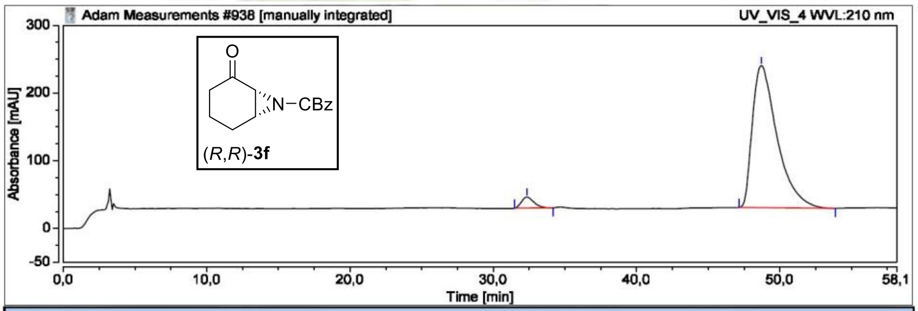

Integration Results							
No.	Peak Name	Retention Time min	Area mAU*min	Height mAU	Relative Area %	Relative Height %	Amount n.a.
1		32,345	16,201	16,417	3,72	7,25	n.a.
2		48,693	418,917	209,865	96,28	92,75	n.a.
Total:			435,117	226,282	100,00	100,00	

Chromatogram

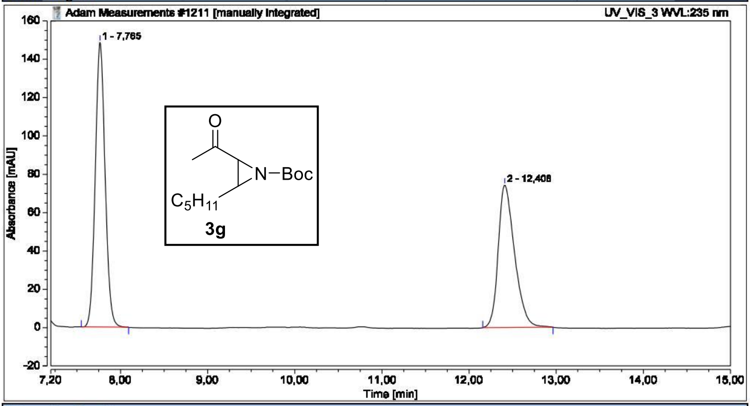

Integration Results

No.	Peak Name	Retention Time min	Area mAU*min	Height mAU	Relative Area %	Relative Height %	Amount
1		7,765	19,483	148,459	54,66	66,63	n.a.
2		12,408	16,164	74,360	45,34	33,37	n.a.
Total:			35,647	222,819	100,00	100,00	

Chromatogram

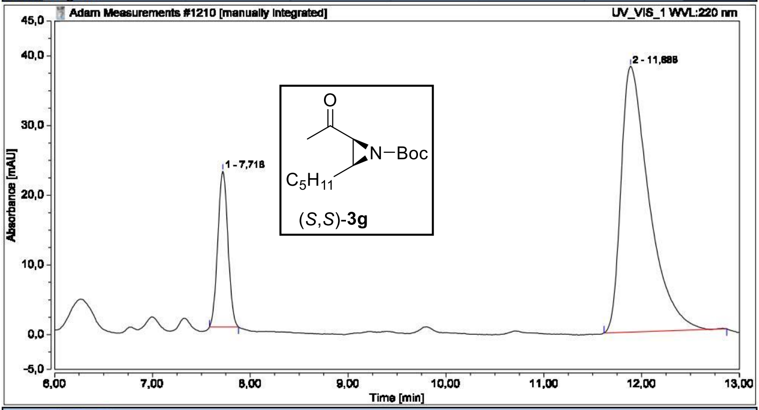

Integration Results

No.	Peak Name	Retention Time min	Area mAU*min	Height mAU	Relative Area %	Relative Height %	Amount
1		7,718	2,678	22,368	17,41	36,97	n.a.
n.a.	1	n.a.	n.a.	n.a.	n.a.	n.a.	n.a.
2		11,888	12,702	38,142	82,59	63,03	n.a.
Total:			15,379	60,509	100,00	100,00	

Chromatogram

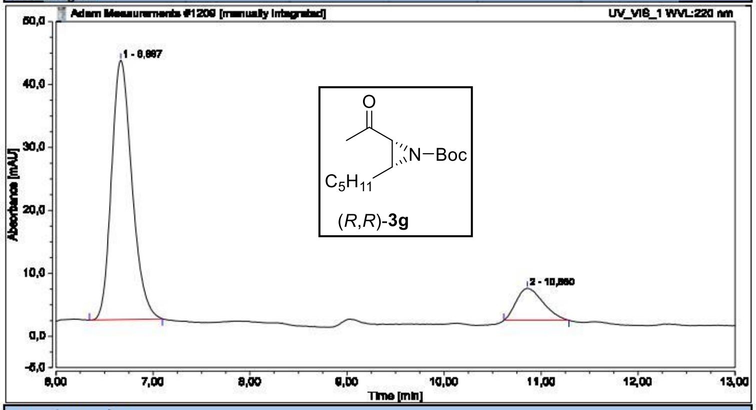

Integration Results

No.	Peak Name	Retention Time min	Area mAU*min	Height mAU	Relative Area %	Relative Height %	Amount
1		6,667	10,050	41,182	86,10	89,04	n.a.
n.a.	1	n.a.	n.a.	n.a.	n.a.	n.a.	n.a.
2		10,860	1,622	5,067	13,90	10,96	n.a.
Total:			11,672	46,250	100,00	100,00	

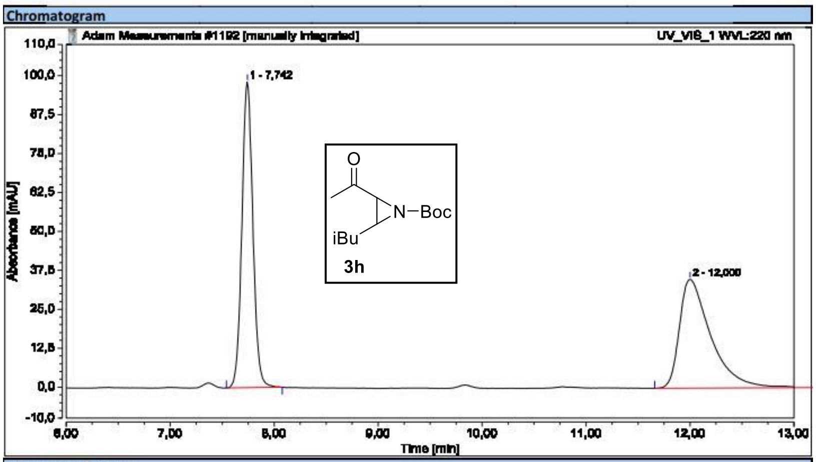

Integration Results

No.	Peak Name	Retention Time min	Area mAU*min	Height mAU	Relative Area %	Relative Height %	Amount
1		7,742	12,006	98,132	49,66	73,76	n.a.
n.a.	1	n.a.	n.a.	n.a.	n.a.	n.a.	n.a.
2		12,000	12,168	34,906	50,34	26,24	n.a.
Total:			24,174	133,038	100,00	100,00	

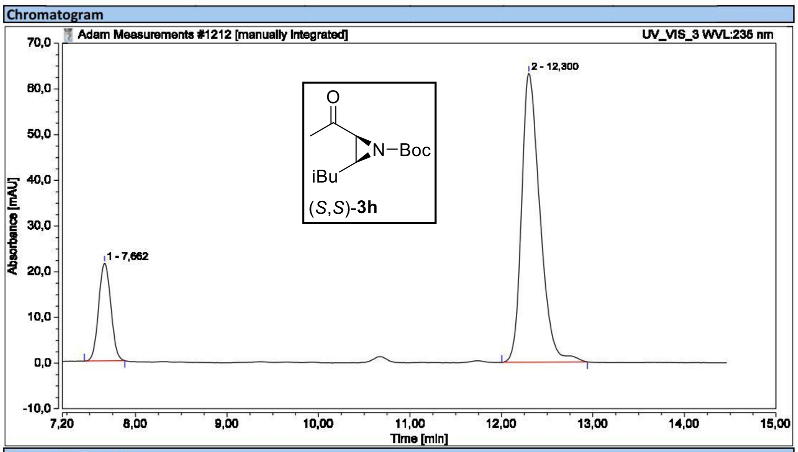

Integration Results

No.	Peak Name	Retention Time min	Area mAU*min	Height mAU	Relative Area %	Relative Height %	Amount
1		7,662	3,293	21,402	18,29	25,29	n.a.
2		12,300	14,712	63,214	81,71	74,71	n.a.
Total:			18,005	84,616	100,00	100,00	

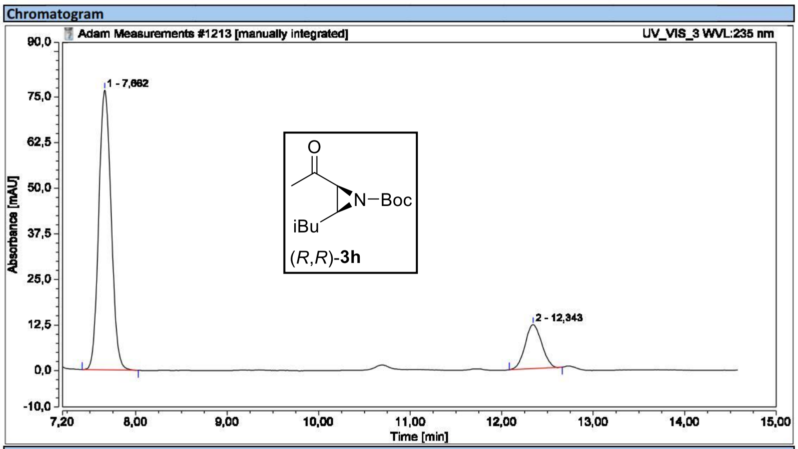

Integration Results

No.	Peak Name	Retention Time min	Area mAU*min	Height mAU	Relative Area %	Relative Height %	Amount
1		7,662	12,010	76,685	82,62	86,41	n.a.
2		12,343	2,526	12,057	17,38	13,59	n.a.
Total:			14,536	88,742	100,00	100,00	

13. Chiral HPLC chromatograms for the asymmetric Aza-Michael/aldol reactions

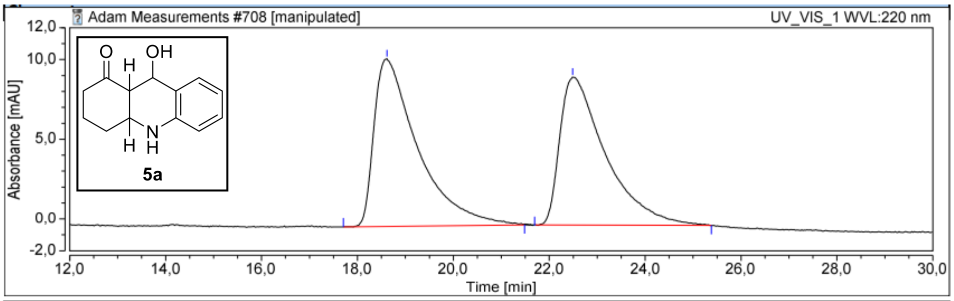

Integration Results							
No.	Peak Name	Retention Time min	Area mAU*min	Height mAU	Relative Area %	Relative Height %	Amount n.a.
1		18,615	11,080	10,513	51,95	53,10	n.a.
2		22,487	10,249	9,285	48,05	46,90	n.a.
**Total:**			**21,329**	**19,798**	**100,00**	**100,00**	

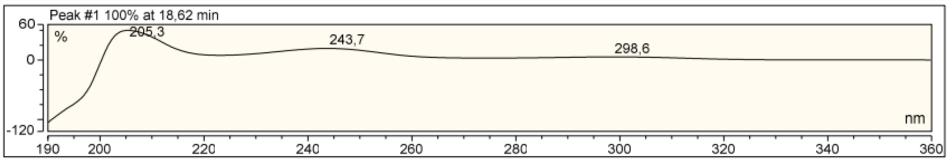

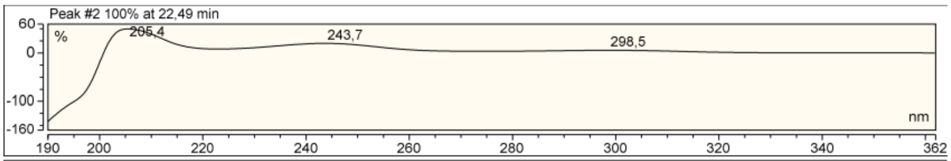

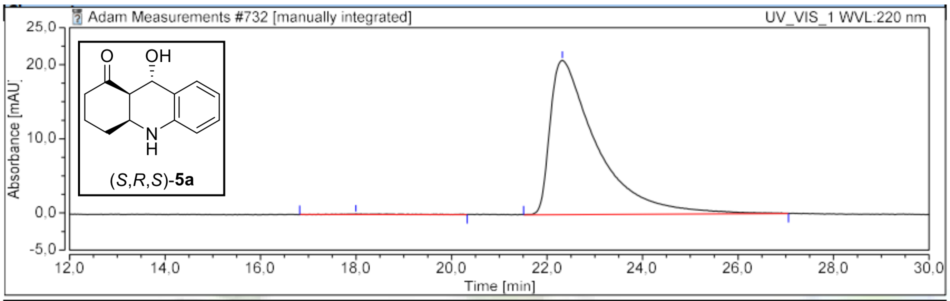

Integration Results							
No.	Peak Name	Retention Time min	Area mAU*min	Height mAU	Relative Area %	Relative Height %	Amount n.a.
1		17,995	0,077	0,085	0,33	0,40	n.a.
2		22,322	23,598	20,827	99,67	99,60	n.a.
**Total:**			**23,676**	**20,912**	**100,00**	**100,00**	

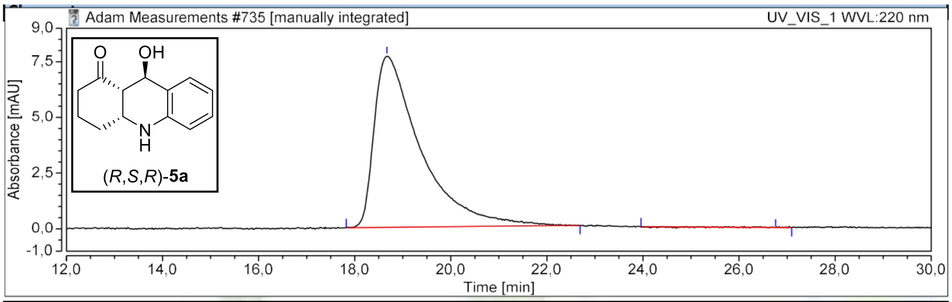

Integration Results							
No.	Peak Name	Retention Time min	Area mAU*min	Height mAU	Relative Area %	Relative Height %	Amount n.a.
1		18,672	8,605	7,694	99,87	99,56	n.a.
2		26,758	0,011	0,034	0,13	0,44	n.a.
**Total:**			**8,616**	**7,728**	**100,00**	**100,00**	

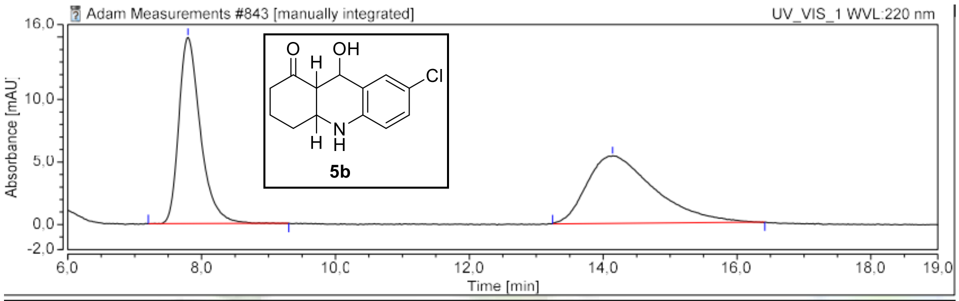

Integration Results							
No.	Peak Name	Retention Time<br>min	Area<br>mAU*min	Height<br>mAU	Relative Area<br>%	Relative Height<br>%	Amount<br>n.a.
1		7,795	5,544	14,898	46,85	73,40	n.a.
2		14,140	6,290	5,399	53,15	26,60	n.a.
**Total:**			**11,834**	**20,298**	**100,00**	**100,00**	

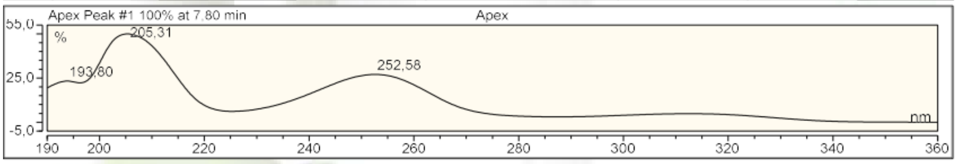

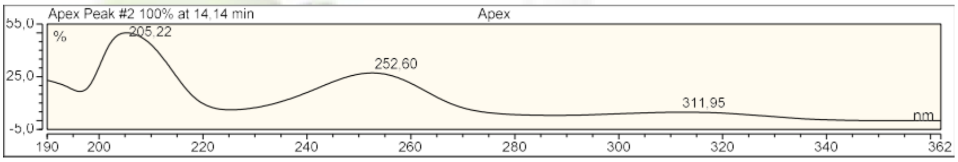

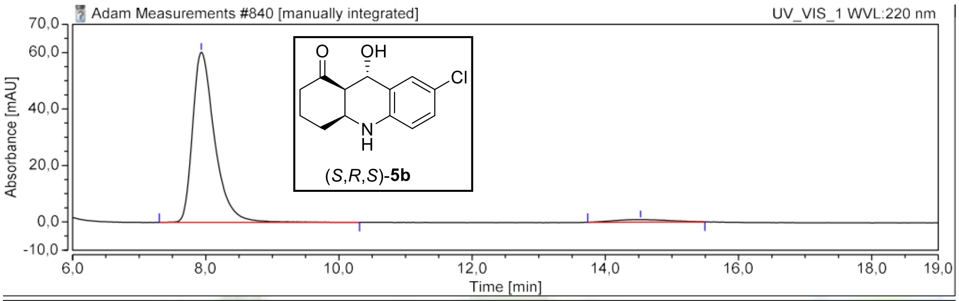

Integration Results							
No.	Peak Name	Retention Time<br>min	Area<br>mAU*min	Height<br>mAU	Relative Area<br>%	Relative Height<br>%	Amount<br>n.a.
1		7,933	23,159	60,331	96,45	98,51	n.a.
2		14,530	0,854	0,913	3,55	1,49	n.a.
**Total:**			**24,012**	**61,244**	**100,00**	**100,00**	

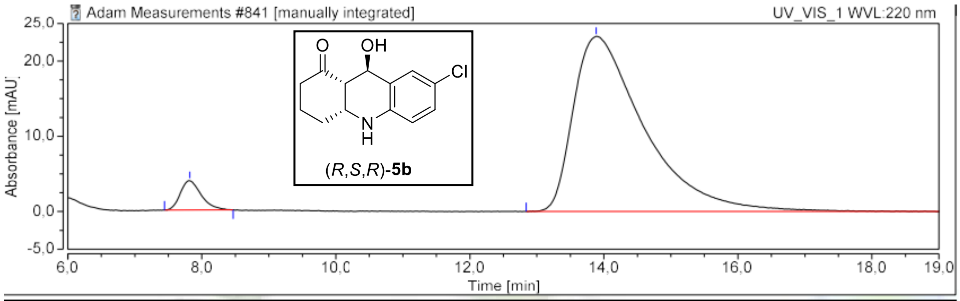

Integration Results							
No.	Peak Name	Retention Time<br>min	Area<br>mAU*min	Height<br>mAU	Relative Area<br>%	Relative Height<br>%	Amount<br>n.a.
1		7,818	1,398	3,912	4,68	14,38	n.a.
2		13,885	28,503	23,293	95,32	85,62	n.a.
**Total:**			**29,902**	**27,204**	**100,00**	**100,00**	

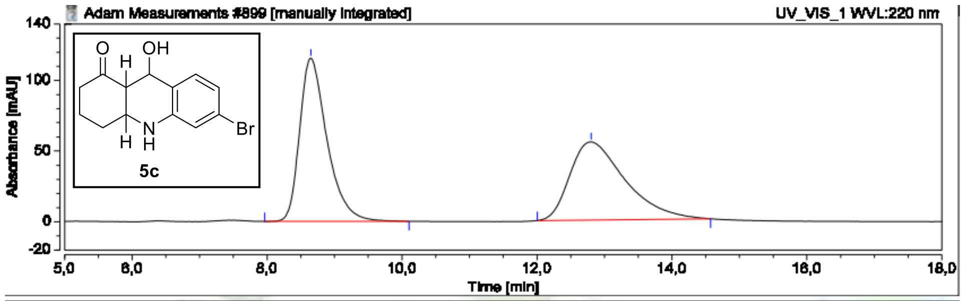

Integration Results

No.	Peak Name	Retention Time min	Area mAU*min	Height mAU	Relative Area %	Relative Height %	Amount n.a.
1		8,648	54,332	115,562	50,92	67,64	n.a.
2		12,802	52,375	55,290	49,08	32,36	n.a.
Total:			106,706	170,852	100,00	100,00	

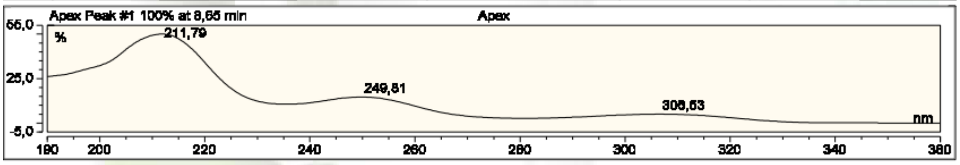

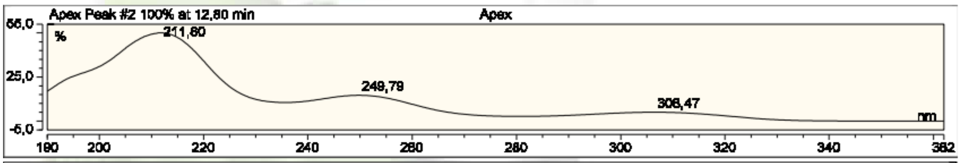

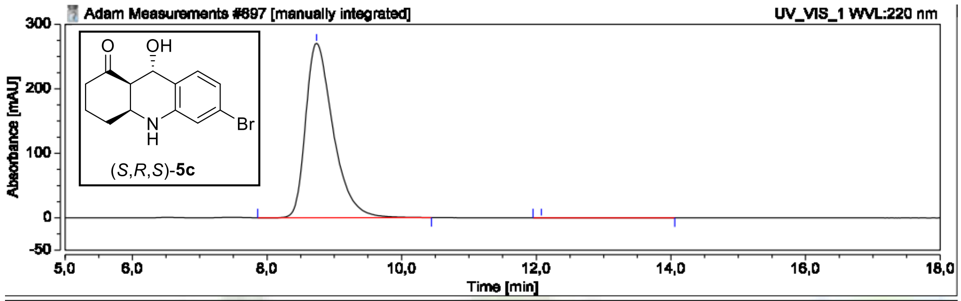

Integration Results

No.	Peak Name	Retention Time min	Area mAU*min	Height mAU	Relative Area %	Relative Height %	Amount n.a.
1		8,737	127,712	270,429	99,99	99,99	n.a.
2		12,077	0,015	0,018	0,01	0,01	n.a.
Total:			127,727	270,447	100,00	100,00	

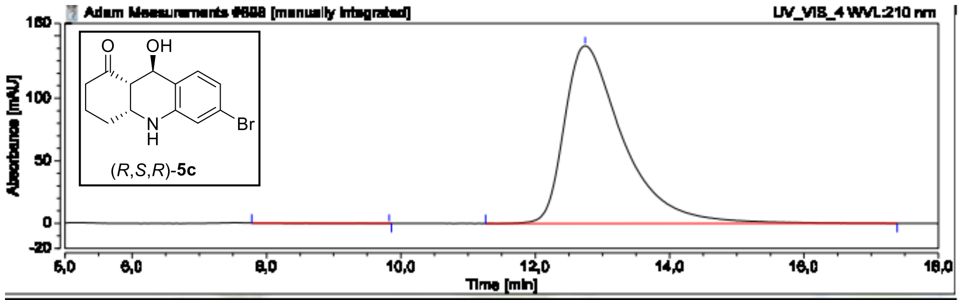

Integration Results

No.	Peak Name	Retention Time min	Area mAU*min	Height mAU	Relative Area %	Relative Height %	Amount n.a.
1		9,825	0,054	0,027	0,04	0,02	n.a.
2		12,742	143,659	142,132	99,96	99,98	n.a.
Total:			143,714	142,159	100,00	100,00	

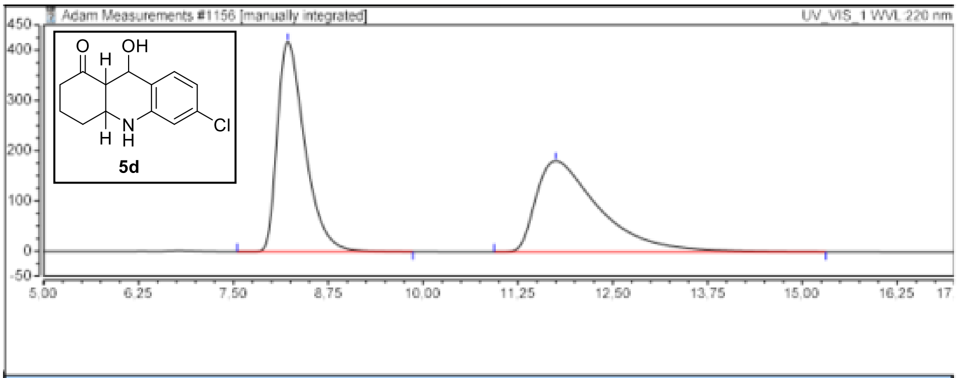

Integration Results							
No.	Peak Name	Retention Time min	Area mAU*min	Height mAU	Relative Area %	Relative Height %	Amount n.a.
1		8,217	176,197	417,600	50,19	69,77	n.a.
2		11,748	174,889	180,952	49,81	30,23	n.a.
Total:			351,087	598,552	100,00	100,00	

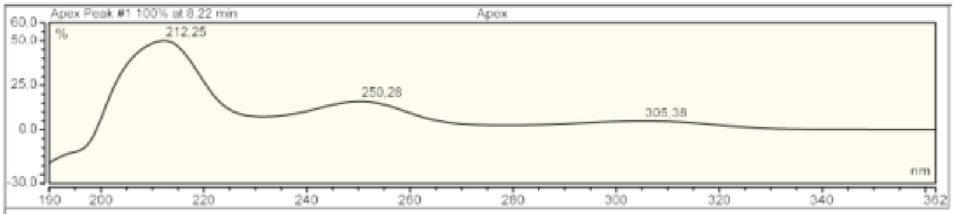

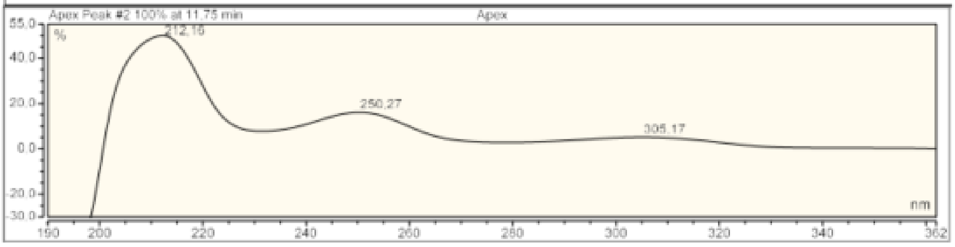

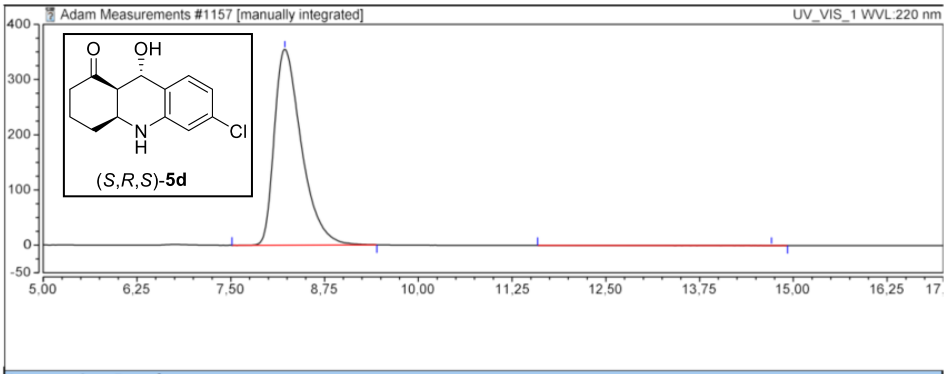

Integration Results							
No.	Peak Name	Retention Time min	Area mAU*min	Height mAU	Relative Area %	Relative Height %	Amount n.a.
1		8,220	148,851	354,859	99,95	99,99	n.a.
2		14,708	0,070	0,048	0,05	0,01	n.a.
Total:			148,922	354,907	100,00	100,00	

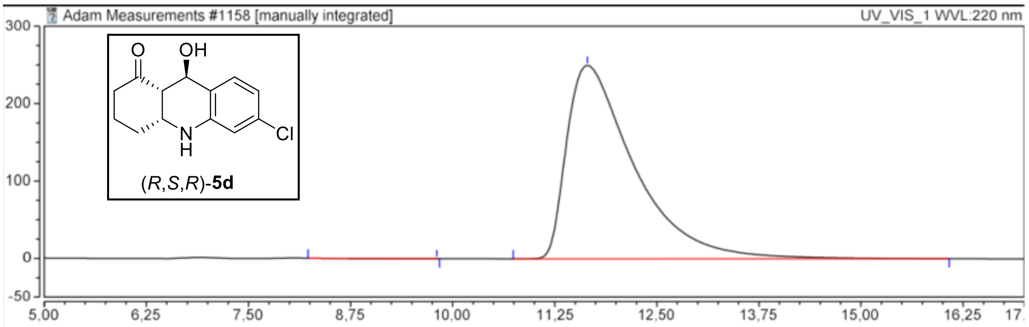

Integration Results

No.	Peak Name	Retention Time min	Area mAU*min	Height mAU	Relative Area %	Relative Height %	Amount n.a.
1		9,807	0,299	0,066	0,12	0,03	n.a.
2		11,648	239,830	249,853	99,88	99,97	n.a.
**Total:**			**240,130**	**249,918**	**100,00**	**100,00**	

Chromatogram

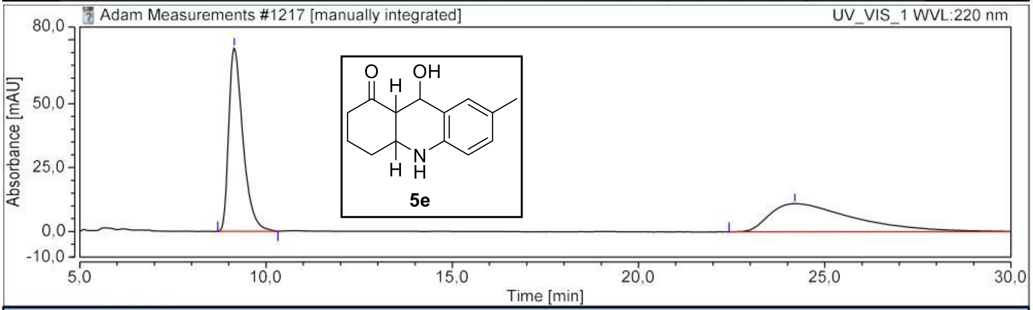

Integration Results

No.	Peak Name	Retention Time min	Area mAU*min	Height mAU	Relative Area %	Relative Height %	Amount n.a.
1		9,145	31,759	71,805	52,60	86,68	n.a.
2		24,197	28,614	11,034	47,40	13,32	n.a.
**Total:**			**60,373**	**82,839**	**100,00**	**100,00**	

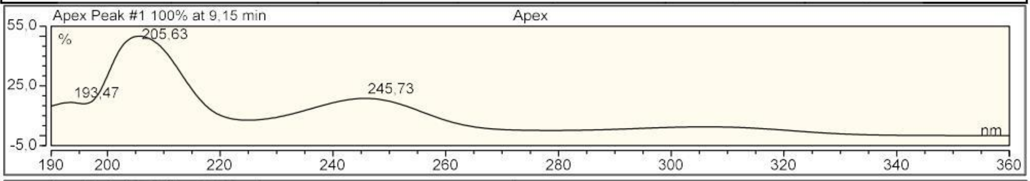

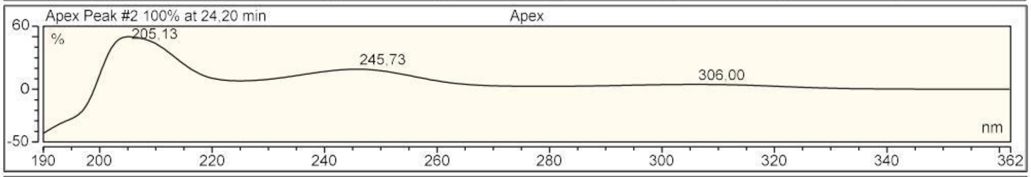

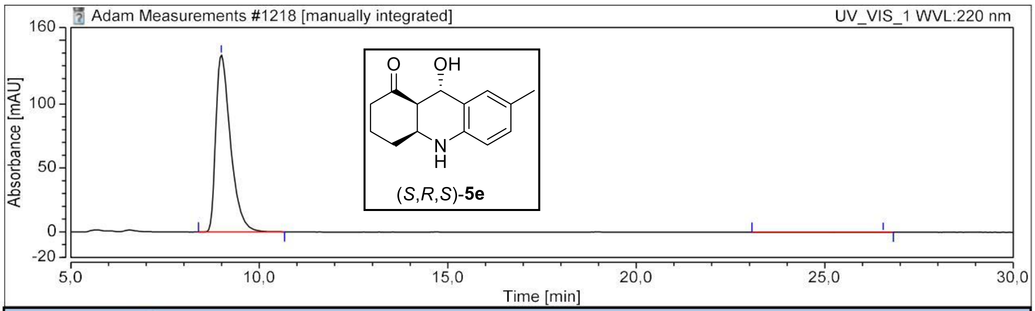

Integration Results							
No.	Peak Name	Retention Time min	Area mAU*min	Height mAU	Relative Area %	Relative Height %	Amount n.a.
1		8,990	62,774	138,581	99,93	99,96	n.a.
2		26,548	0,042	0,054	0,07	0,04	n.a.
**Total:**			**62,815**	**138,635**	**100,00**	**100,00**	

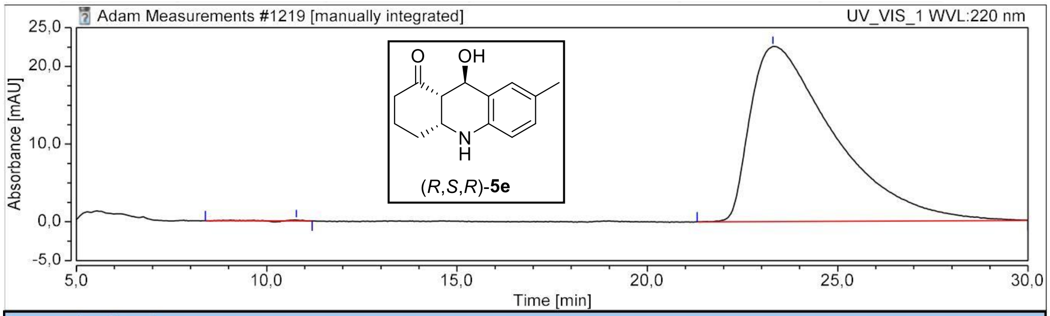

Integration Results							
No.	Peak Name	Retention Time min	Area mAU*min	Height mAU	Relative Area %	Relative Height %	Amount n.a.
1		10,780	0,067	0,156	0,12	0,69	n.a.
2		23,300	57,614	22,542	99,88	99,31	n.a.
**Total:**			**57,681**	**22,698**	**100,00**	**100,00**	

14. Chiral HPLC chromatograms for the asymmetric Michael-Initiated Ring Closure/intramolecular aldol sequence

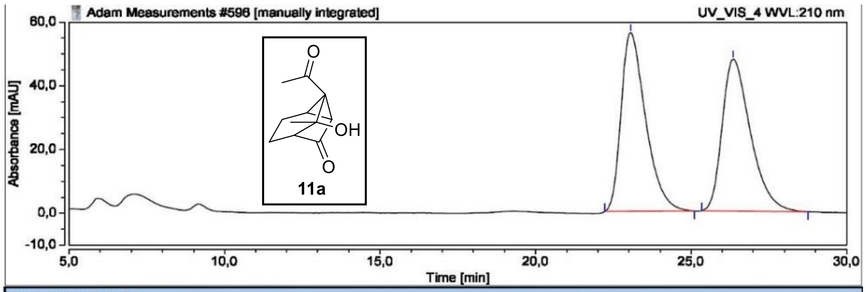

Integration Results							
No.	Peak Name	Retention Time min	Area mAU*min	Height mAU	Relative Area %	Relative Height %	Amount n.a.
1		23,055	51,224	56,148	50,46	54,09	n.a.
2		26,350	50,286	47,652	49,54	45,91	n.a.
Total:			101,511	103,800	100,00	100,00	

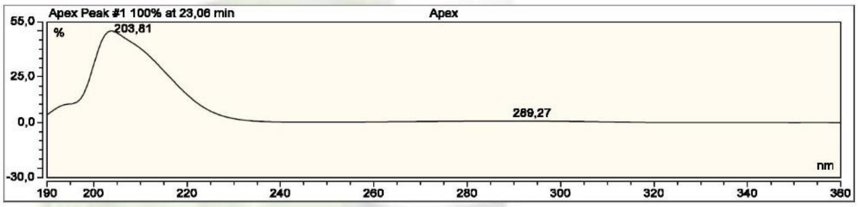

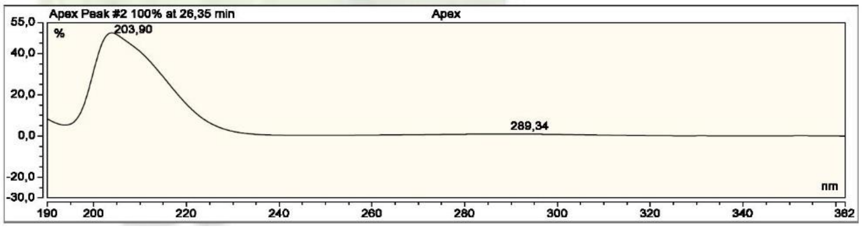

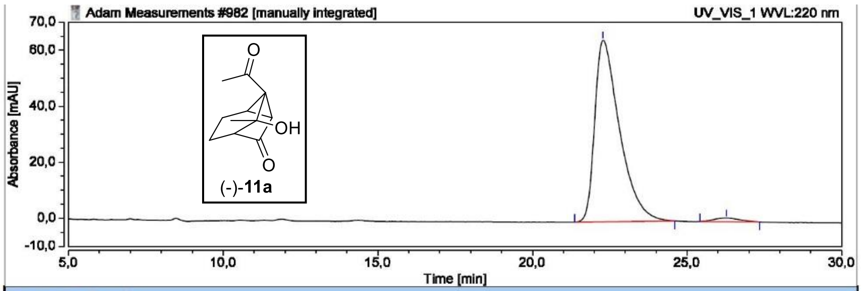

Integration Results							
No.	Peak Name	Retention Time min	Area mAU*min	Height mAU	Relative Area %	Relative Height %	Amount n.a.
1		22,285	60,385	64,902	97,92	97,95	n.a.
2		26,267	1,281	1,357	2,08	2,05	n.a.
Total:			61,666	66,258	100,00	100,00	

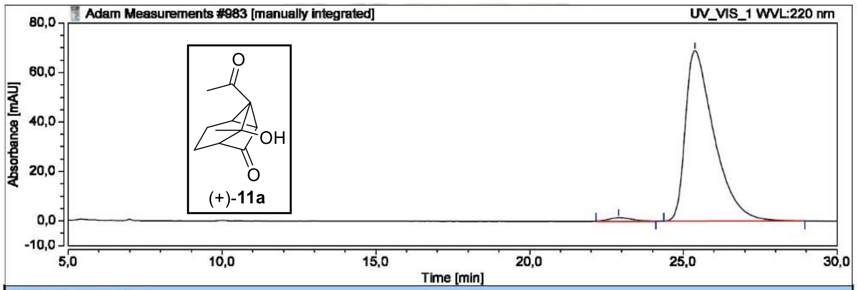

Integration Results							
No.	Peak Name	Retention Time min	Area mAU*min	Height mAU	Relative Area %	Relative Height %	Amount n.a.
1		22,888	1,190	1,412	1,57	2,01	n.a.
2		25,372	74,789	68,946	98,43	97,99	n.a.
Total:			75,980	70,358	100,00	100,00	

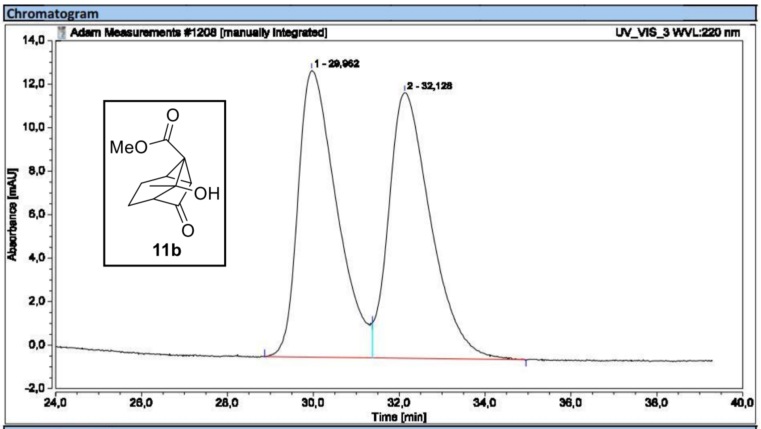

Integration Results

No.	Peak Name	Retention Time min	Area mAU*min	Height mAU	Relative Area %	Relative Height %	Amount
1		29,962	13,735	13,195	49,84	51,92	n.a.
2		32,128	13,821	12,221	50,16	48,08	n.a.
Total:			27,556	25,417	100,00	100,00	

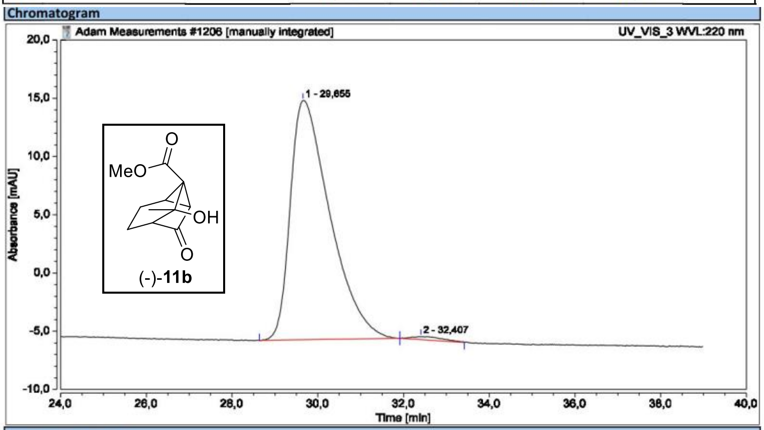

Integration Results

No.	Peak Name	Retention Time min	Area mAU*min	Height mAU	Relative Area %	Relative Height %	Amount
1		29,655	21,989	20,568	98,93	98,63	n.a.
2		32,407	0,237	0,286	1,07	1,37	n.a.
Total:			22,227	20,854	100,00	100,00	

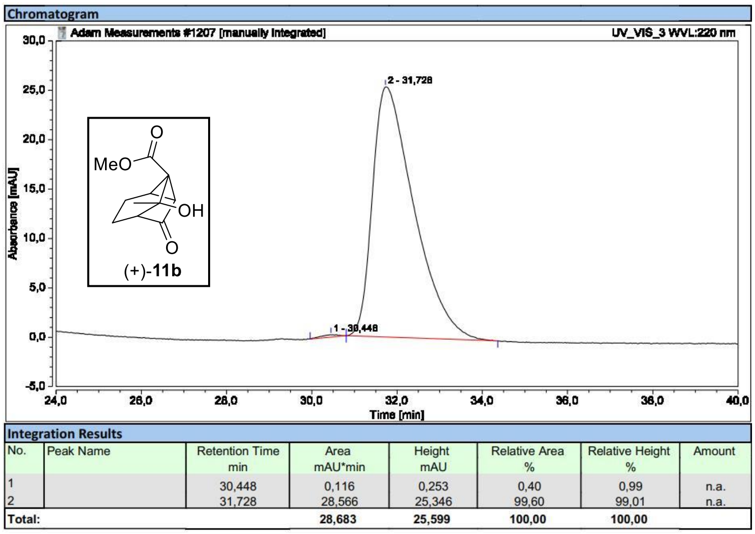

Integration Results

No.	Peak Name	Retention Time min	Area mAU*min	Height mAU	Relative Area %	Relative Height %	Amount
1		30,448	0,116	0,253	0,40	0,99	n.a.
2		31,728	28,566	25,346	99,60	99,01	n.a.
Total:			28,683	25,599	100,00	100,00	

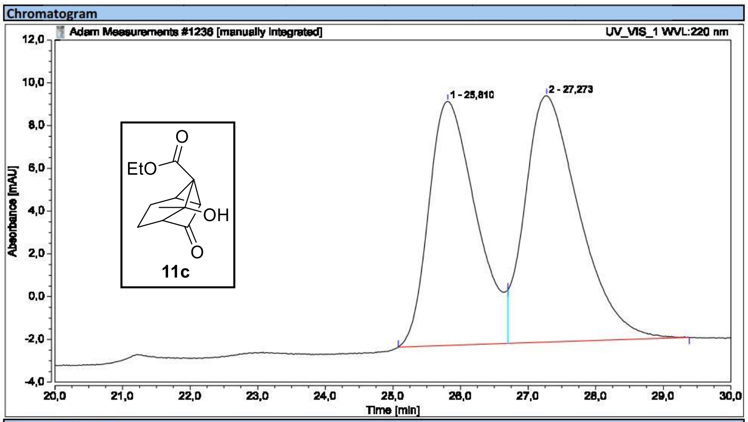

Integration Results

No.	Peak Name	Retention Time min	Area mAU*min	Height mAU	Relative Area %	Relative Height %	Amount
n.a.	1	n.a.	n.a.	n.a.	n.a.	n.a.	n.a.
1		25,810	9,238	11,420	46,24	49,76	n.a.
2		27,273	10,738	11,529	53,76	50,24	n.a.
Total:			19,976	22,950	100,00	100,00	

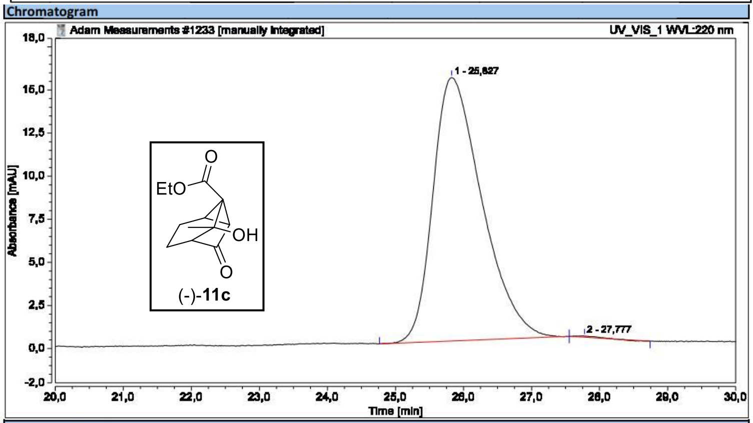

Integration Results

No.	Peak Name	Retention Time min	Area mAU*min	Height mAU	Relative Area %	Relative Height %	Amount
n.a.	1	n.a.	n.a.	n.a.	n.a.	n.a.	n.a.
1		25,827	12,754	15,297	99,85	99,46	n.a.
2		27,777	0,019	0,083	0,15	0,54	n.a.
Total:			12,774	15,381	100,00	100,00	

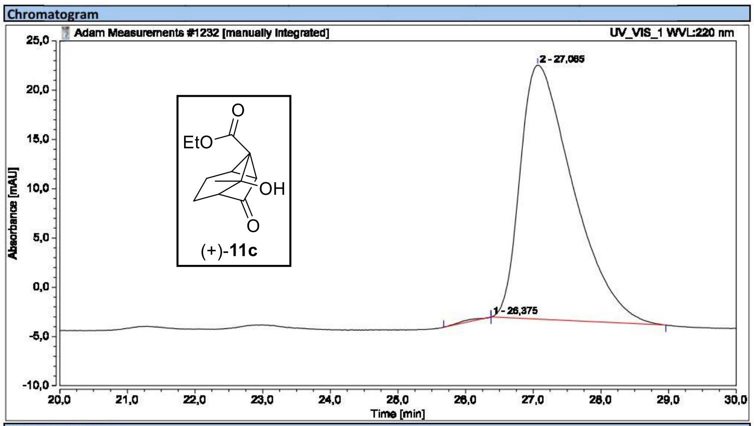

Integration Results

No.	Peak Name	Retention Time min	Area mAU*min	Height mAU	Relative Area %	Relative Height %	Amount
n.a.	1	n.a.	n.a.	n.a.	n.a.	n.a.	n.a.
1		26,375	0,073	0,000	0,31	0,00	n.a.
2		27,065	23,477	25,768	99,69	100,00	n.a.
Total:			23,550	25,768	100,00	100,00	

Chromatogram

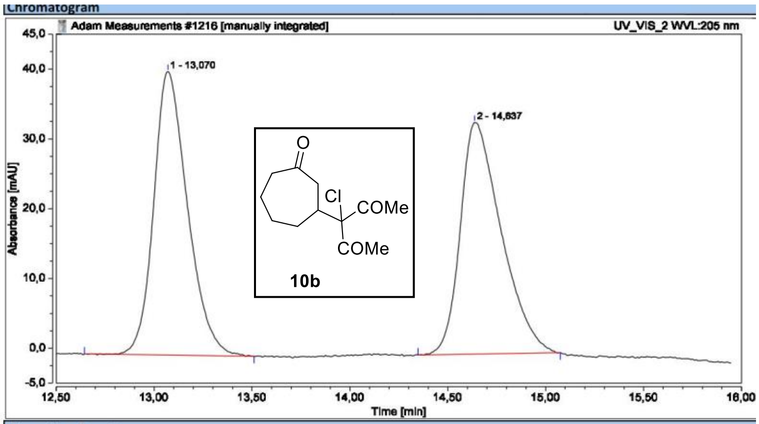

Integration Results

No.	Peak Name	Retention Time min	Area mAU*min	Height mAU	Relative Area %	Relative Height %	Amount n.a.
1		13,070	8,141	40,617	50,30	55,04	n.a.
2		14,637	8,045	33,175	49,70	44,96	n.a.
Total:			16,186	73,793	100,00	100,00	

Chromatogram

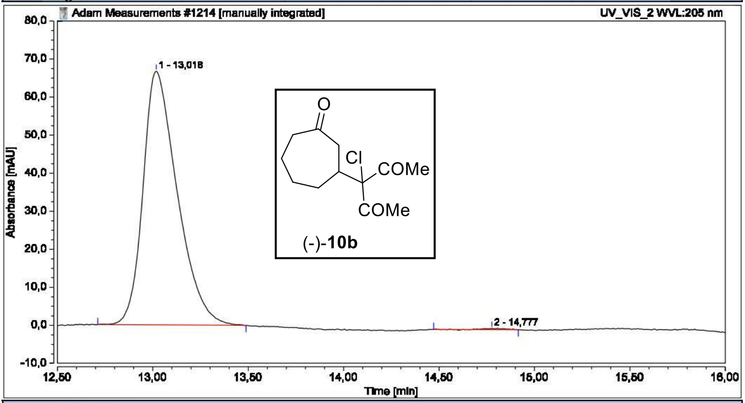

Integration Results

No.	Peak Name	Retention Time min	Area mAU*min	Height mAU	Relative Area %	Relative Height %	Amount
1		13,018	13,877	66,661	99,76	99,50	n.a.
2		14,777	0,034	0,335	0,24	0,50	n.a.
Total:			13,911	66,996	100,00	100,00	

Chromatogram

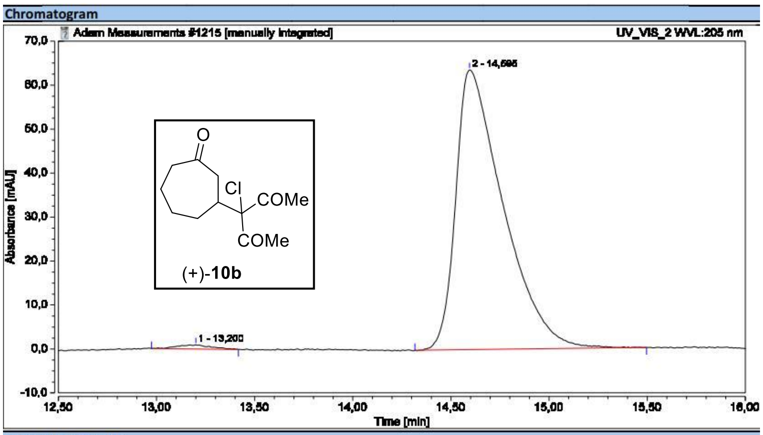

Integration Results

No.	Peak Name	Retention Time min	Area mAU*min	Height mAU	Relative Area %	Relative Height %	Amount
1		13,200	0,185	0,953	1,03	1,48	n.a.
2		14,595	17,860	63,620	98,97	98,52	n.a.
Total:			18,045	64,573	100,00	100,00	

15. NMR spectra of chiral diamines

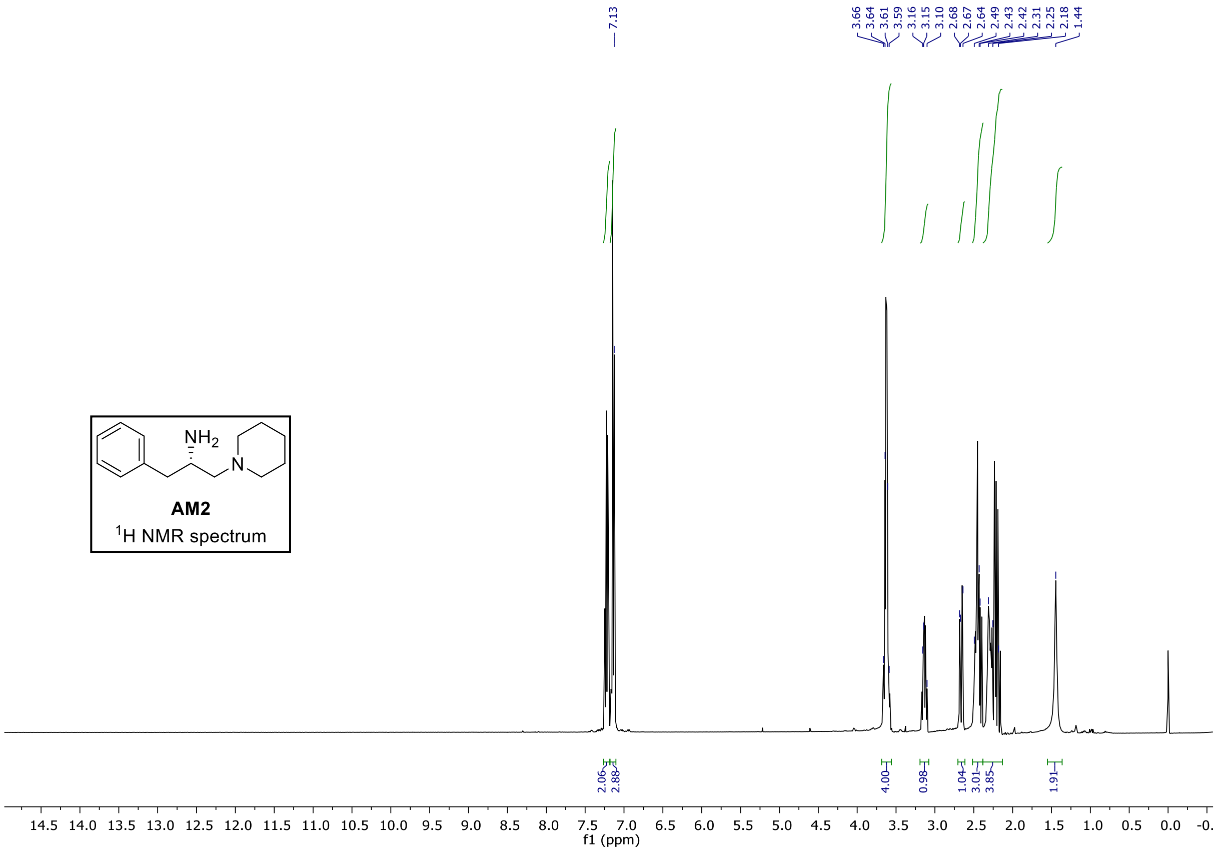

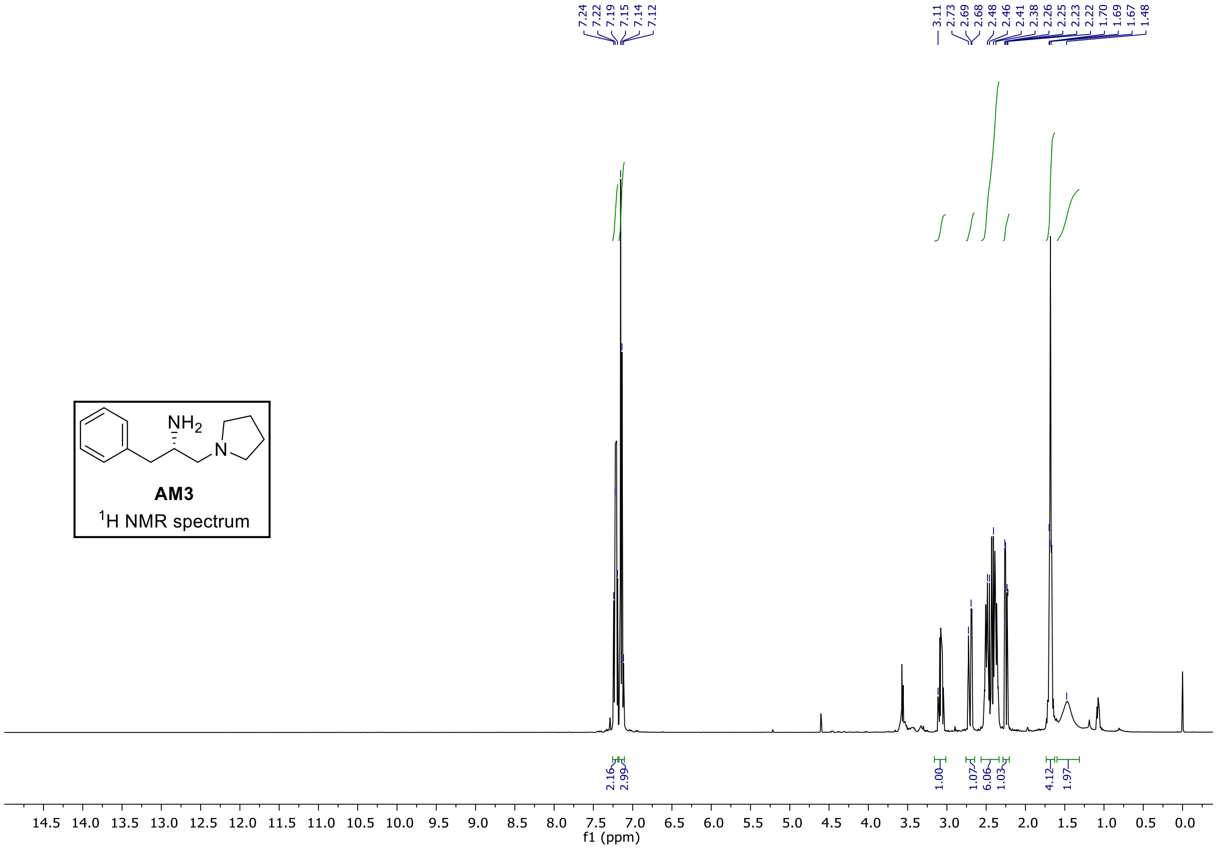

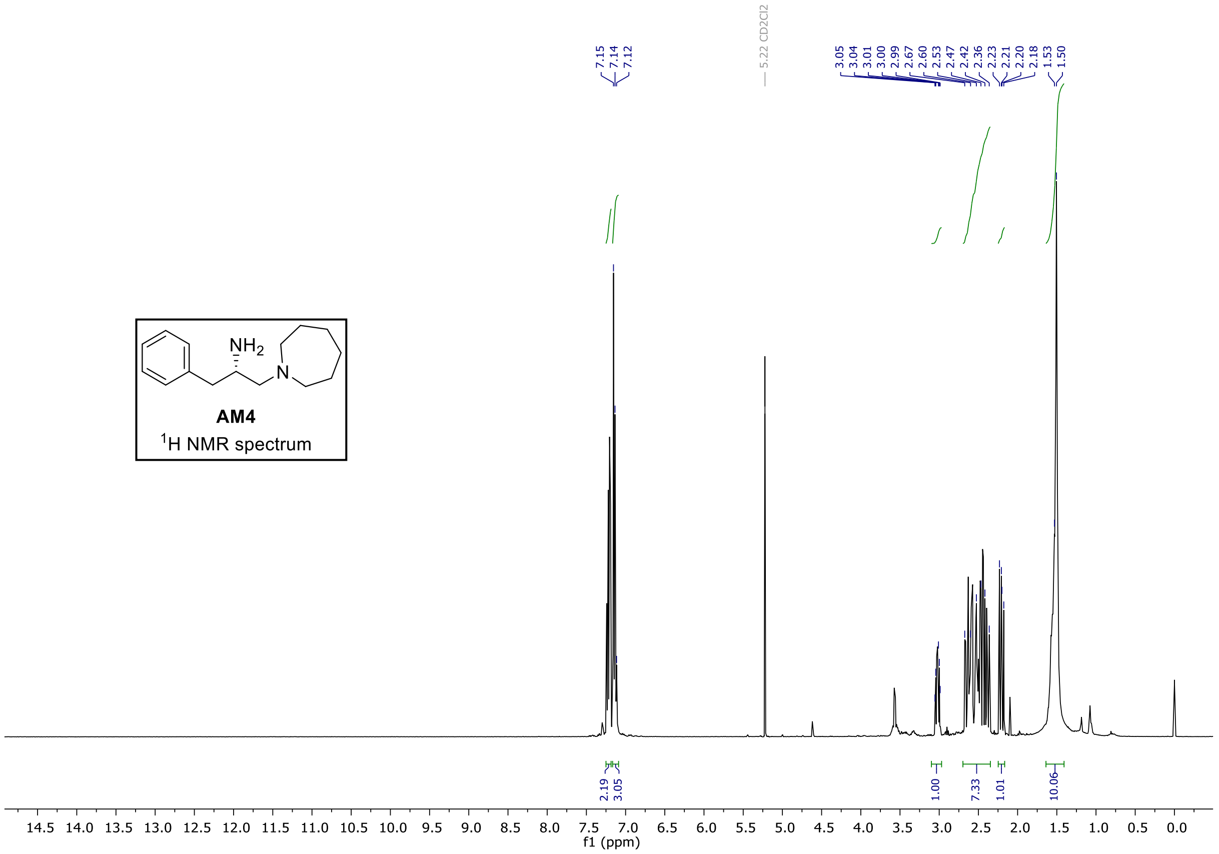
NH2
N
AM4
1H NMR spectrum
5.22 CD2Cl2
f1 (ppm)

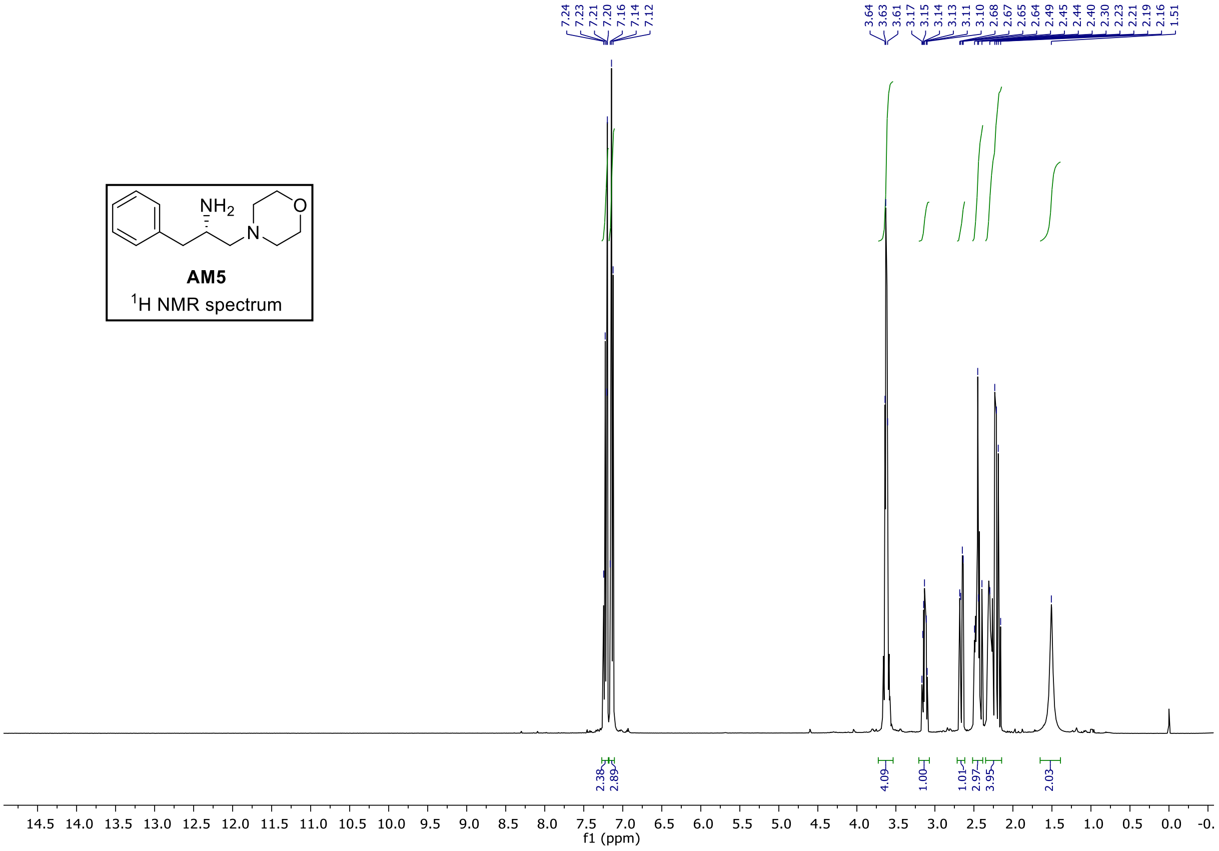
NH2
N
O
AM5
1H NMR spectrum
f1 (ppm)

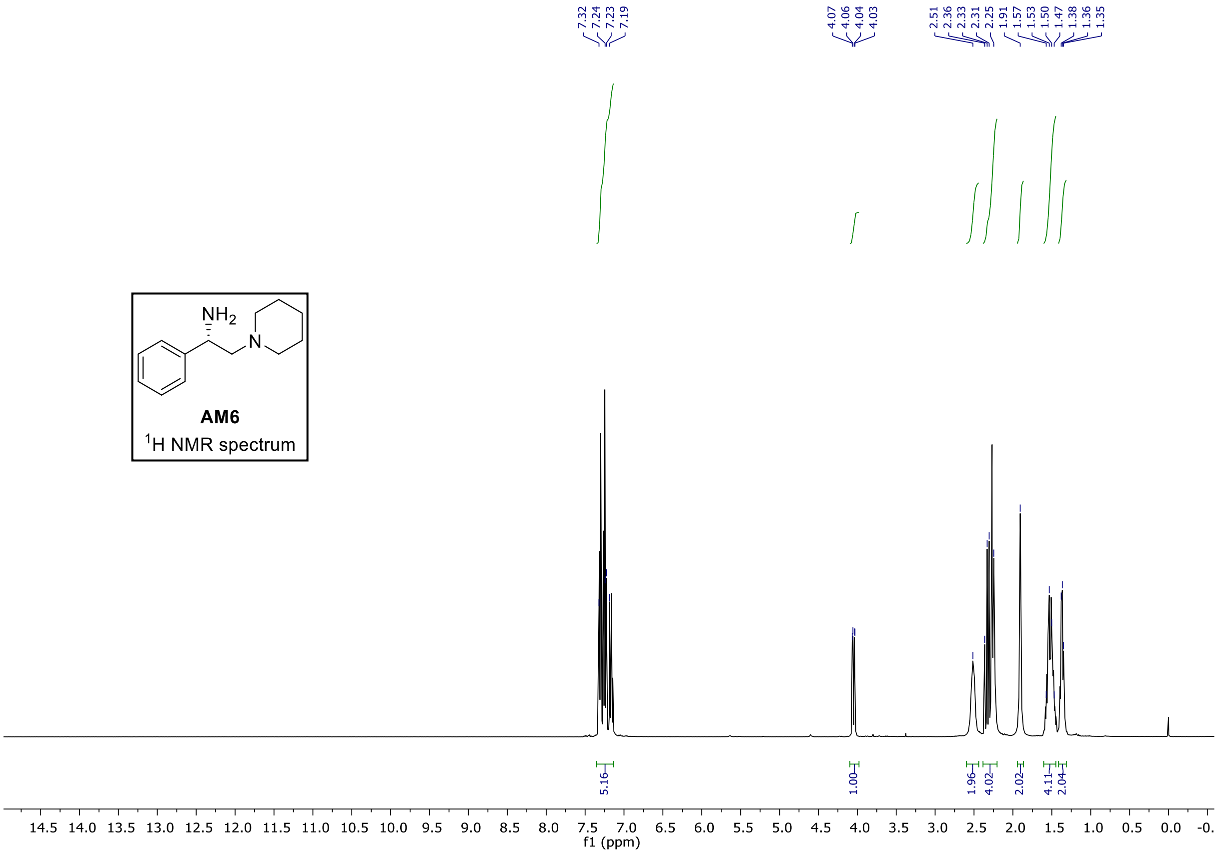
7.32
7.24
7.23
7.19
4.07
4.06
4.04
4.03
2.51
2.36
2.33
2.31
2.25
1.91
1.57
1.53
1.50
1.47
1.38
1.36
1.35
NH2
N
AM6
1H NMR spectrum
5.16
1.00
1.96
4.02
2.02
4.11
2.04
f1 (ppm)

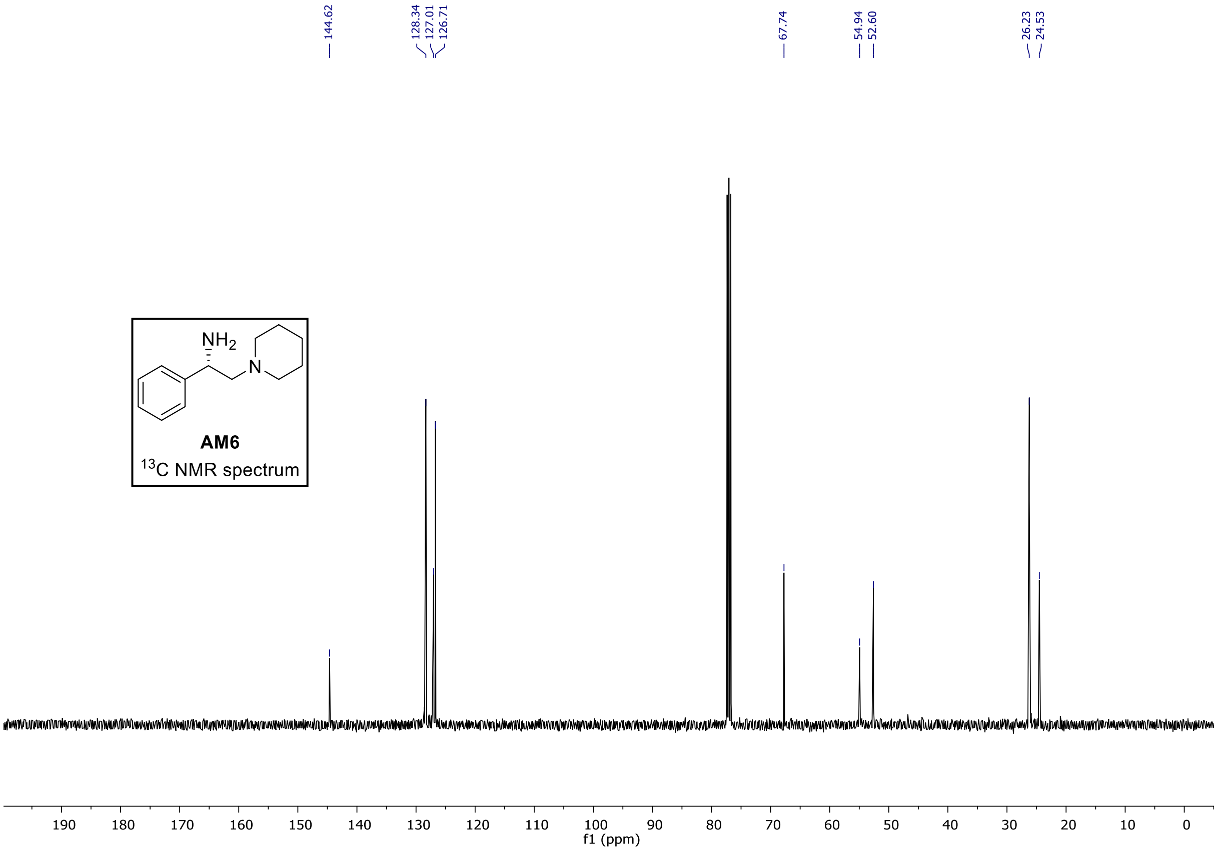
144.62
128.34
127.01
126.71
67.74
54.94
52.60
26.23
24.53
NH2
N
AM6
13C NMR spectrum
f1 (ppm)

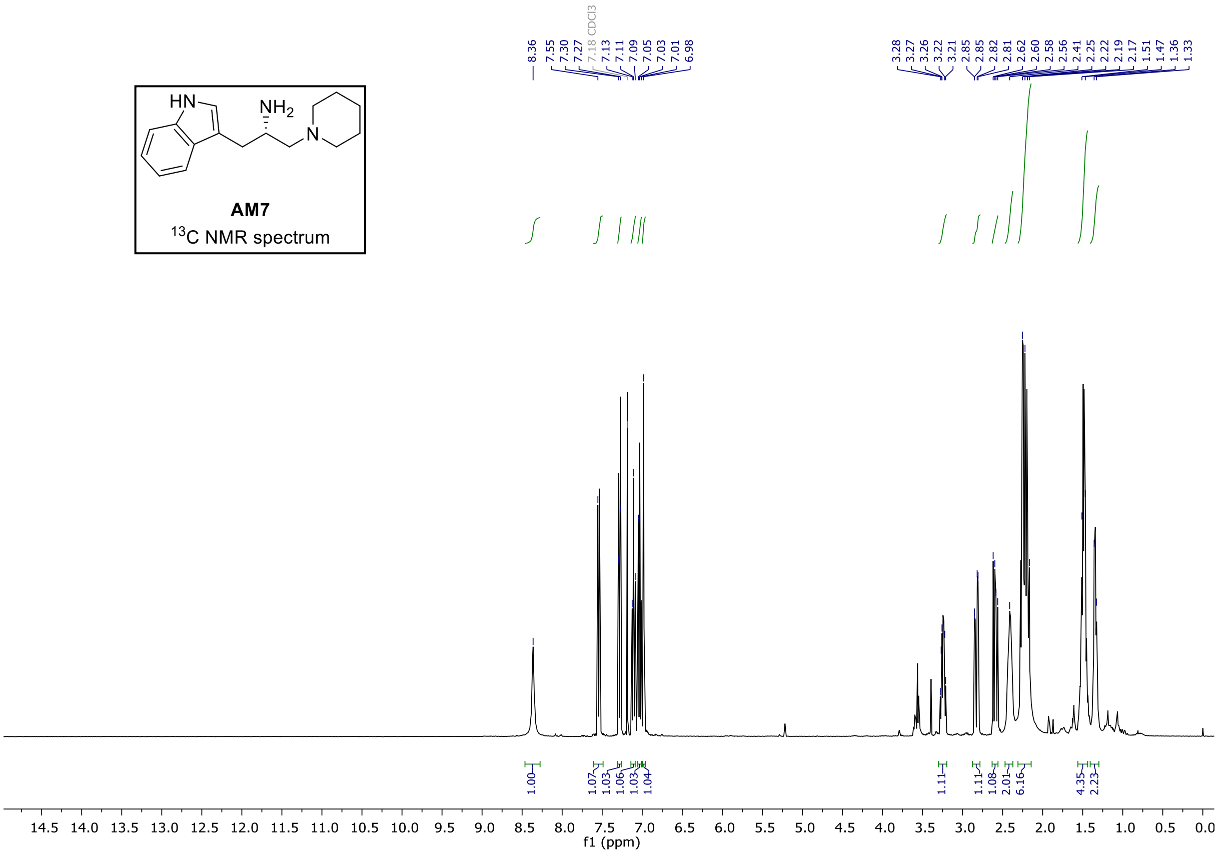

AM7
^{13}C NMR spectrum
8.36
7.55
7.30
7.27
7.18 CDCl3
7.13
7.11
7.09
7.05
7.03
7.01
6.98
3.28
3.27
3.26
3.22
3.21
2.85
2.85
2.82
2.81
2.62
2.60
2.58
2.56
2.41
2.25
2.22
2.19
2.17
1.51
1.47
1.36
1.33
1.00
1.07
1.03
1.06
1.03
1.04
1.11
1.11
1.08
2.01
6.16
4.35
2.23
f1 (ppm)

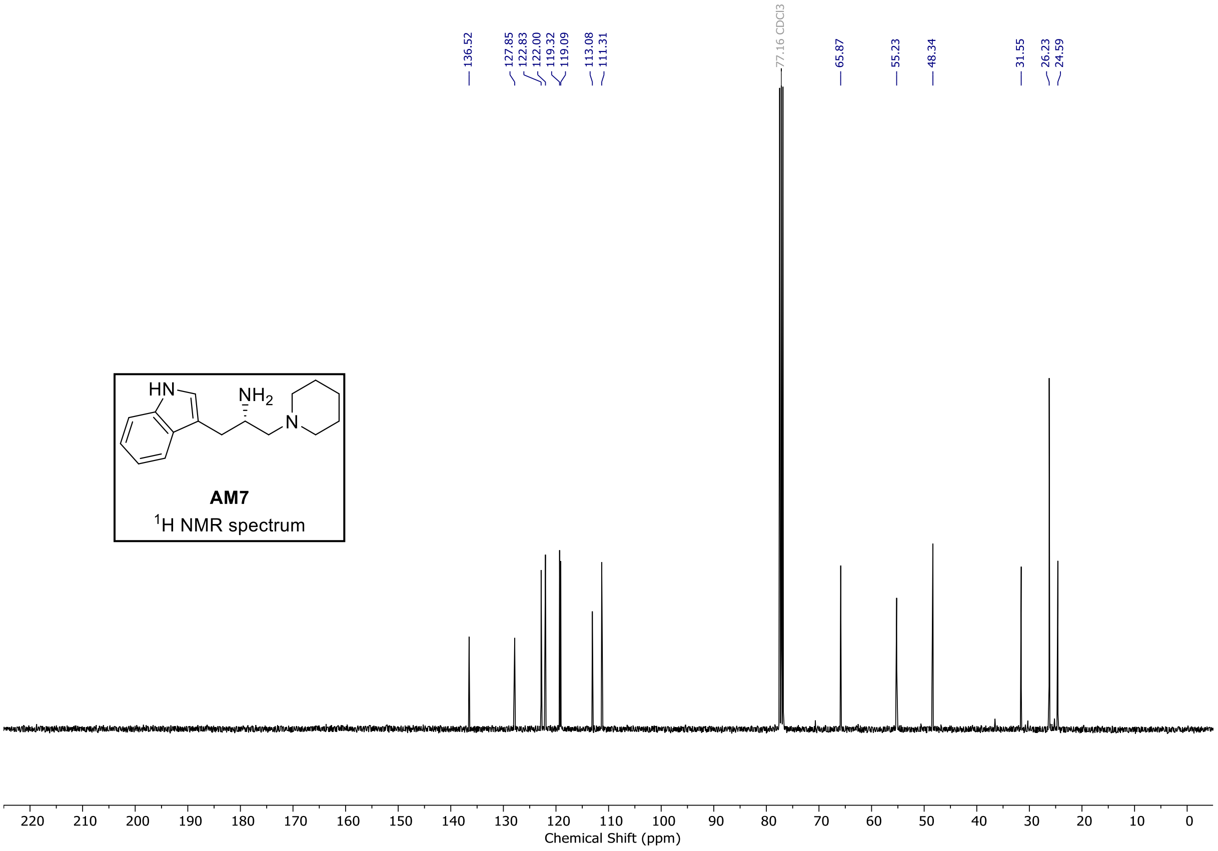

AM7
^{1}H NMR spectrum
136.52
127.85
122.83
122.00
119.32
119.09
113.08
111.31
77.16 CDCl3
65.87
55.23
48.34
31.55
26.23
24.59
Chemical Shift (ppm)

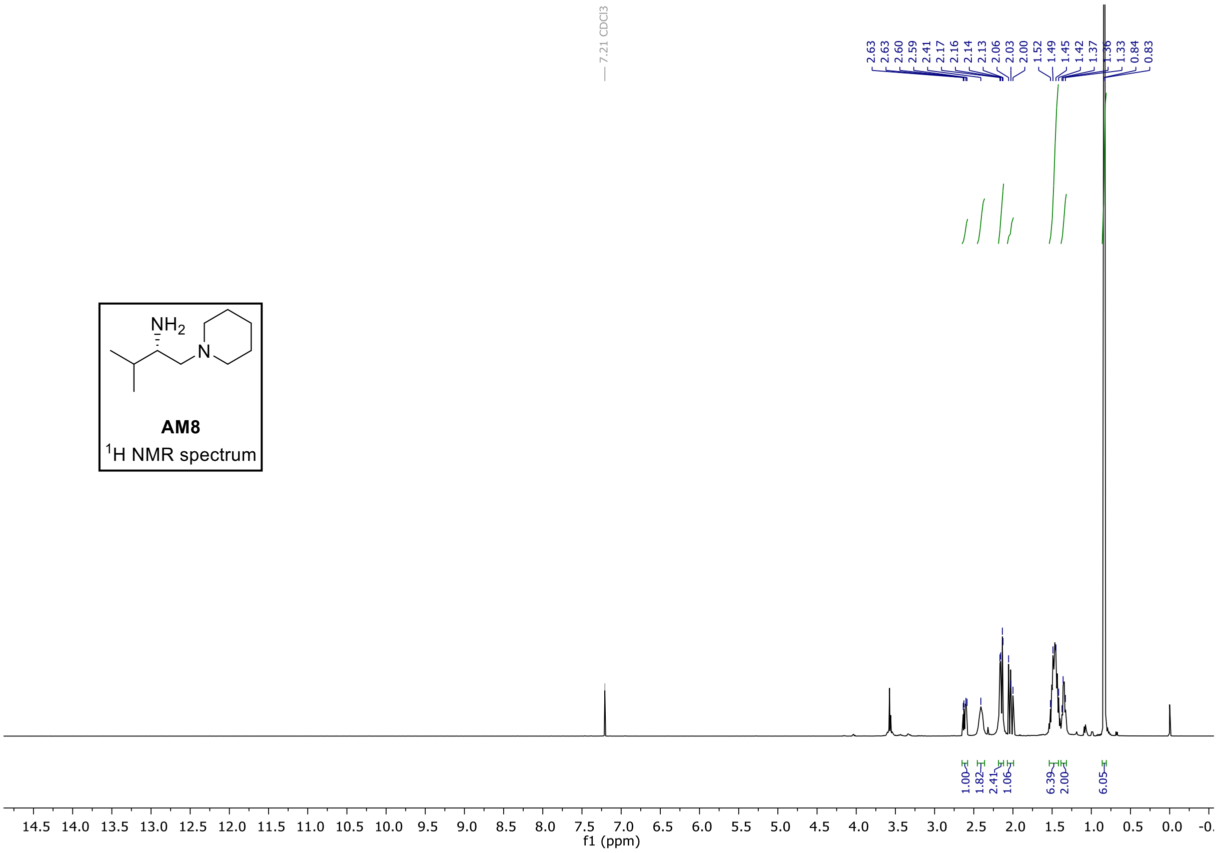
AM8
^{1}H NMR spectrum
7.21 CDCl3
2.63
2.63
2.60
2.59
2.41
2.17
2.16
2.14
2.13
2.06
2.03
2.00
1.52
1.49
1.45
1.42
1.37
1.36
1.33
0.84
0.83
1.00
1.82
2.41
1.06
6.39
2.00
6.05
f1 (ppm)

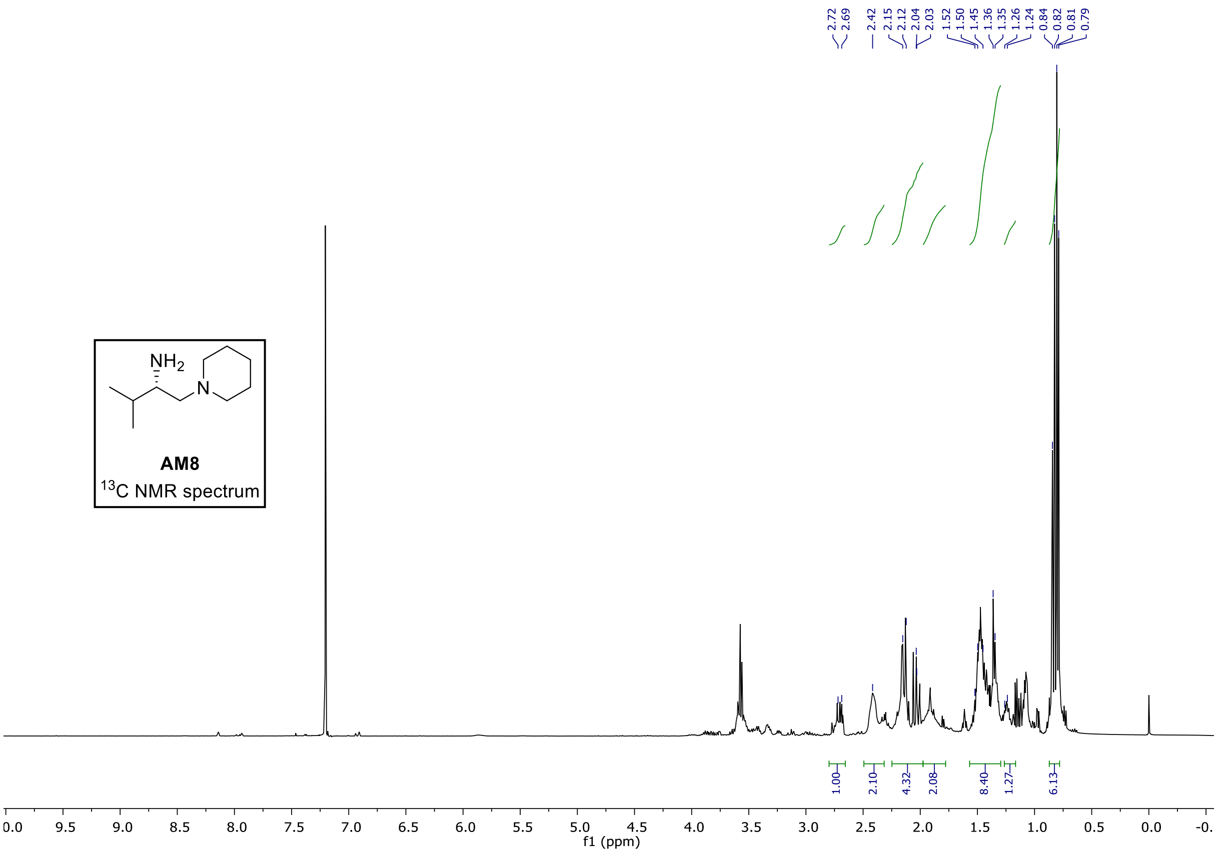
AM8
^{13}C NMR spectrum
2.72
2.69
2.42
2.15
2.12
2.04
2.03
1.52
1.50
1.45
1.36
1.35
1.26
1.24
0.84
0.82
0.81
0.79
1.00
2.10
4.32
2.08
8.40
1.27
6.13
f1 (ppm)

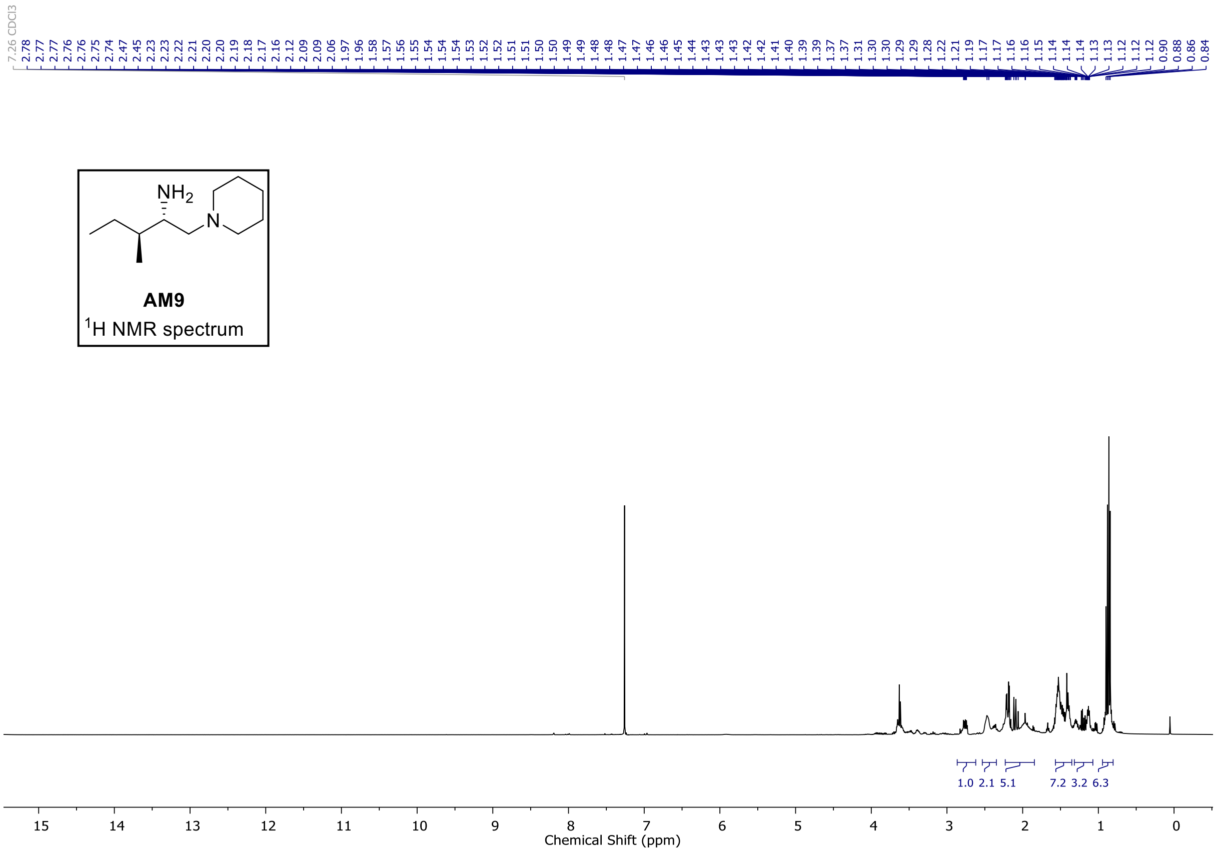
7.26 CDCl3
2.78 2.77 2.77 2.76 2.75 2.74 2.47 2.45 2.23 2.23 2.22 2.21 2.20 2.20 2.19 2.18 2.17 2.16 2.12 2.09 2.09 2.06 1.97 1.96 1.58 1.57 1.56 1.55 1.54 1.54 1.54 1.53 1.52 1.52 1.51 1.51 1.50 1.50 1.49 1.49 1.48 1.48 1.47 1.47 1.46 1.46 1.45 1.44 1.43 1.43 1.43 1.42 1.42 1.41 1.40 1.39 1.39 1.37 1.37 1.31 1.30 1.30 1.29 1.29 1.28 1.22 1.21 1.19 1.17 1.17 1.16 1.16 1.15 1.14 1.14 1.14 1.13 1.13 1.12 1.12 1.12 0.90 0.88 0.86 0.84
NH2
N
AM9
1H NMR spectrum
1.0 2.1 5.1 7.2 3.2 6.3
15 14 13 12 11 10 9 8 7 6 5 4 3 2 1 0
Chemical Shift (ppm)

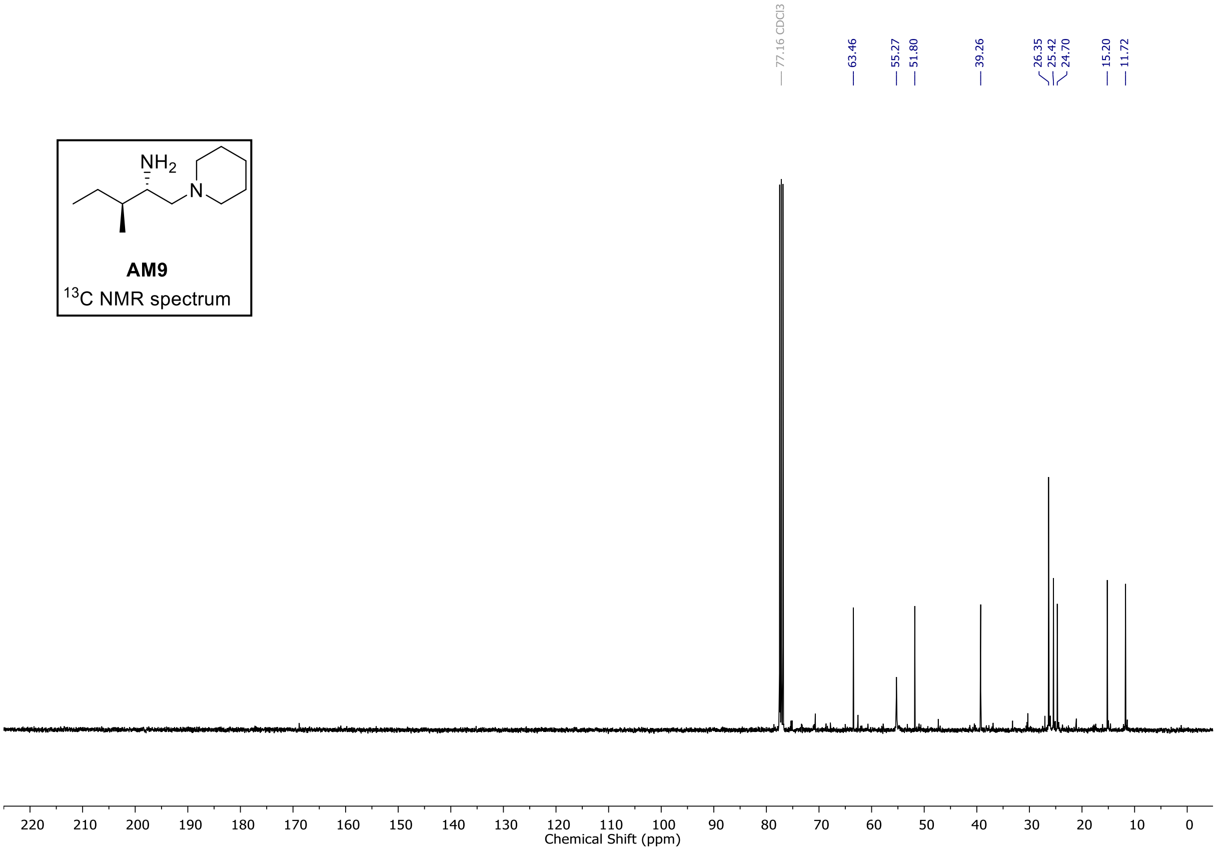
77.16 CDCl3
63.46
55.27
51.80
39.26
26.35
25.42
24.70
15.20
11.72
NH2
N
AM9
13C NMR spectrum
220 210 200 190 180 170 160 150 140 130 120 110 100 90 80 70 60 50 40 30 20 10 0
Chemical Shift (ppm)

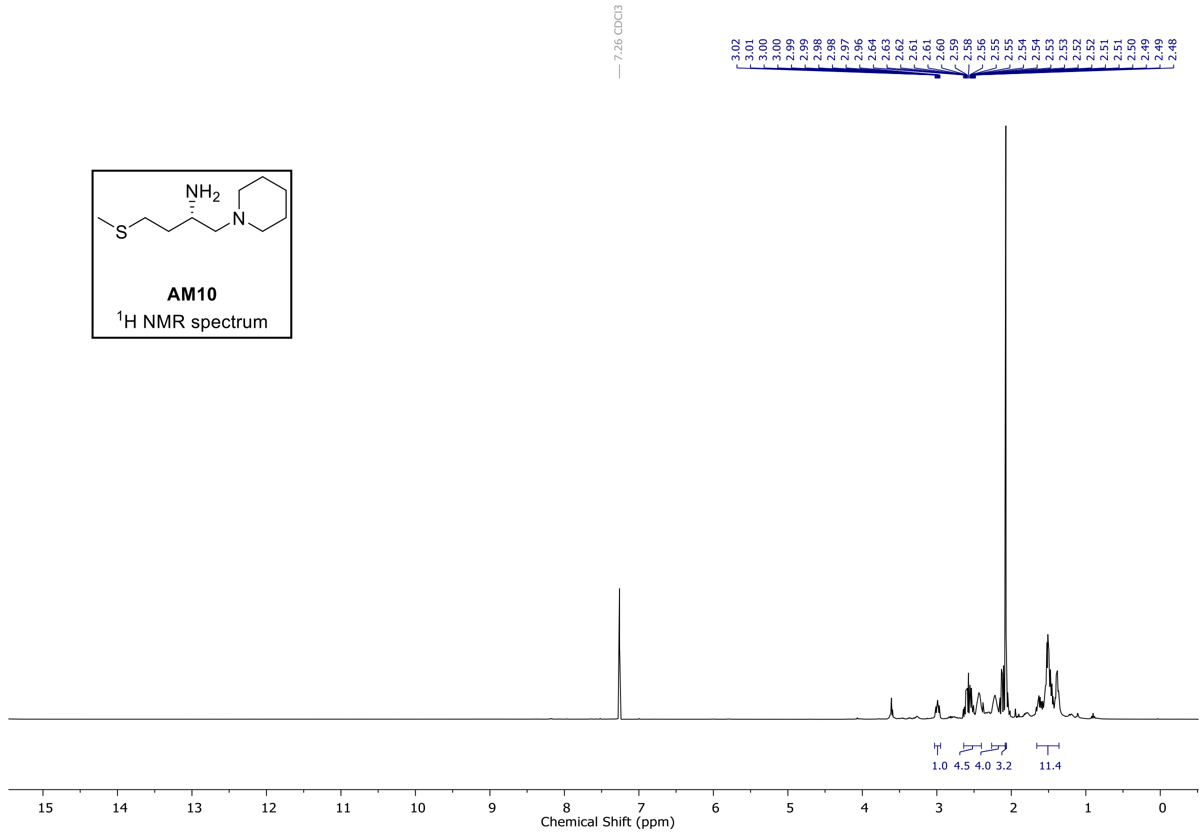
NH2
S
N
AM10
1H NMR spectrum
7.26 CDCl3
3.02
3.01
3.00
2.99
2.99
2.98
2.98
2.97
2.96
2.64
2.63
2.62
2.61
2.61
2.60
2.59
2.58
2.56
2.55
2.55
2.54
2.54
2.53
2.53
2.52
2.52
2.51
2.50
2.49
2.48
1.0 4.5 4.0 3.2
11.4
15 14 13 12 11 10 9 8 7 6 5 4 3 2 1 0
Chemical Shift (ppm)

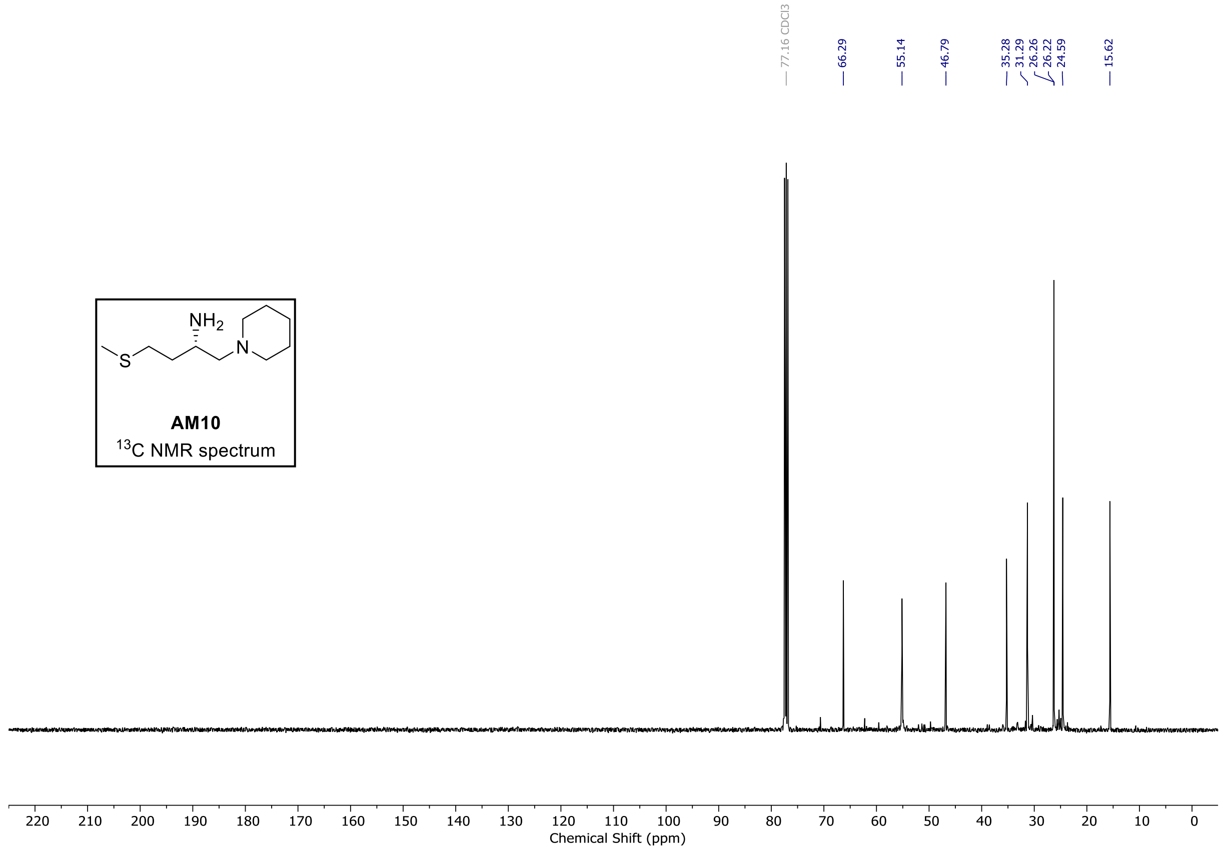
NH2
S
N
AM10
13C NMR spectrum
77.16 CDCl3
66.29
55.14
46.79
35.28
31.29
26.26
26.22
24.59
15.62
220 210 200 190 180 170 160 150 140 130 120 110 100 90 80 70 60 50 40 30 20 10 0
Chemical Shift (ppm)

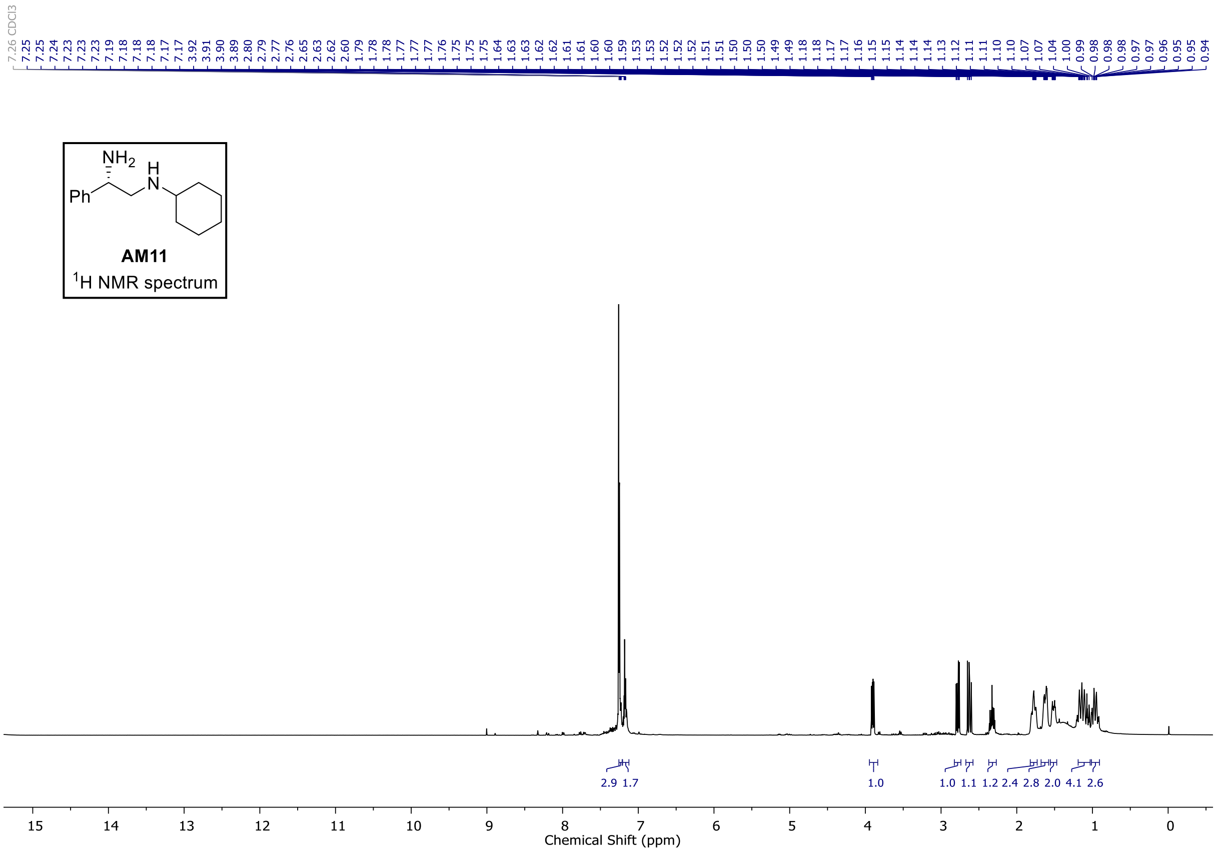
7.26 CDCl3
7.25 7.25 7.24 7.23 7.23 7.23 7.19 7.18 7.18 7.18 7.17 7.17 3.92 3.91 3.90 3.89 2.80 2.79 2.77 2.76 2.65 2.63 2.62 2.60 1.79 1.78 1.78 1.77 1.77 1.77 1.76 1.75 1.75 1.75 1.64 1.63 1.63 1.62 1.62 1.61 1.61 1.60 1.60 1.59 1.53 1.53 1.53 1.52 1.52 1.52 1.51 1.51 1.50 1.50 1.50 1.49 1.49 1.18 1.18 1.17 1.17 1.16 1.15 1.15 1.14 1.14 1.14 1.13 1.12 1.11 1.11 1.10 1.10 1.07 1.04 1.00 0.99 0.98 0.98 0.98 0.97 0.97 0.96 0.95 0.95 0.94
NH2
H
N
Ph
AM11
1H NMR spectrum
2.9 1.7
1.0
1.0 1.1 1.2 2.4 2.8 2.0 4.1 2.6
15 14 13 12 11 10 9 8 7 6 5 4 3 2 1 0
Chemical Shift (ppm)

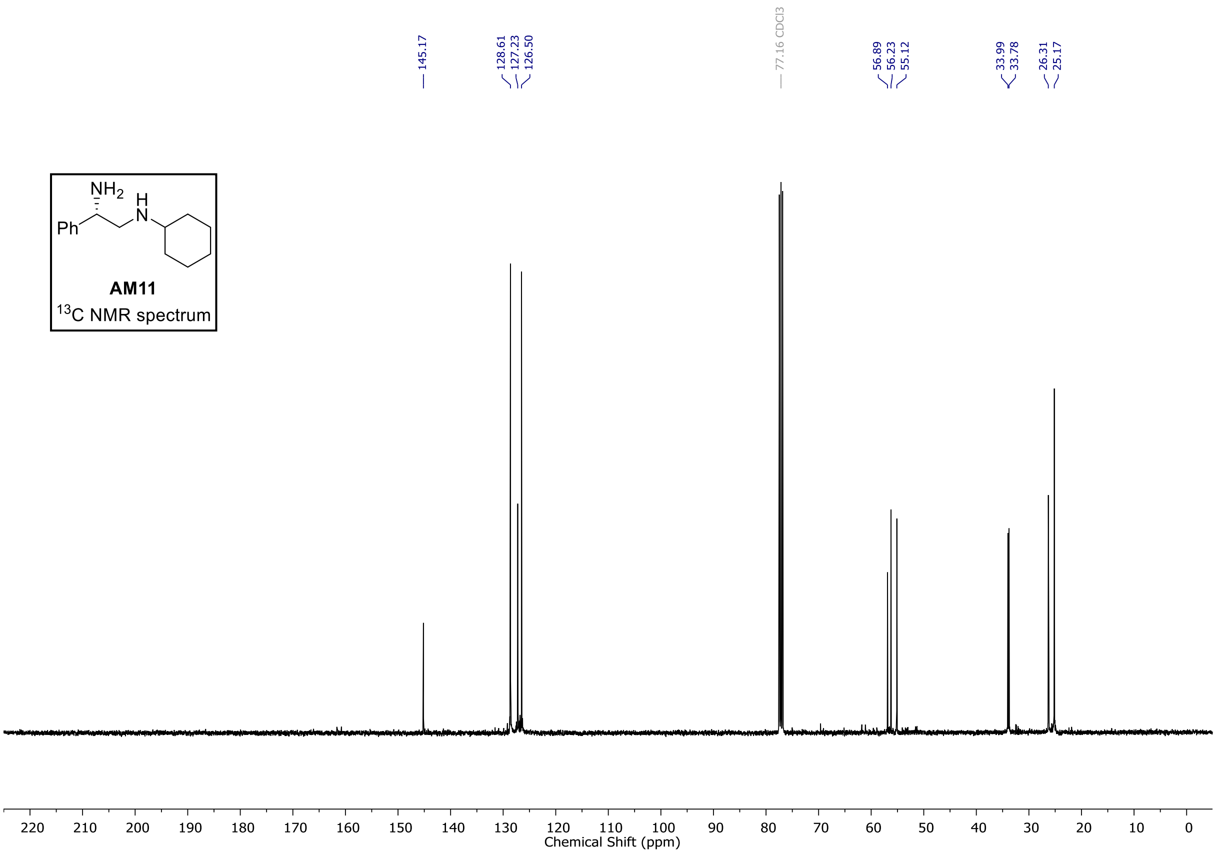
145.17
128.61 127.23 126.50
77.16 CDCl3
56.89 56.23 55.12
33.99 33.78
26.31 25.17
NH2
H
N
Ph
AM11
13C NMR spectrum
220 210 200 190 180 170 160 150 140 130 120 110 100 90 80 70 60 50 40 30 20 10 0
Chemical Shift (ppm)

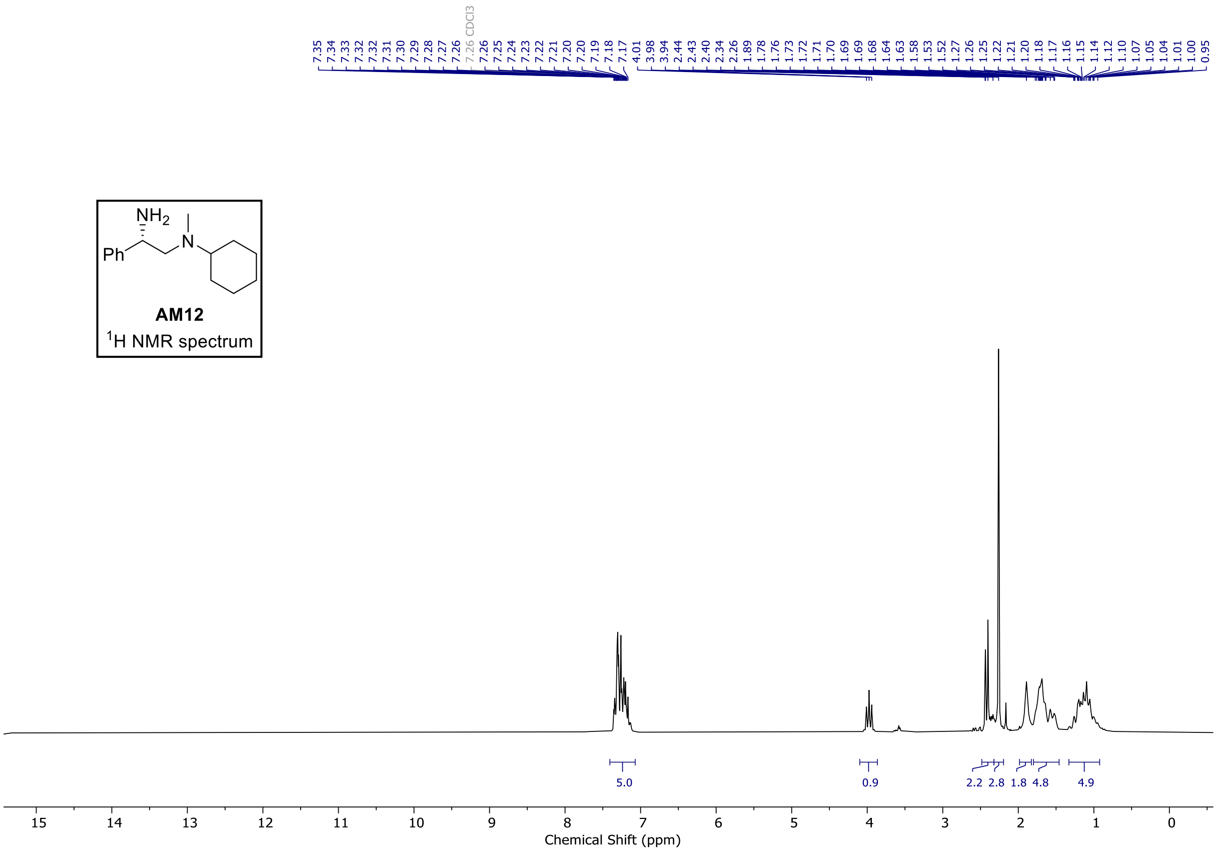
NH2
Ph
N
AM12
1H NMR spectrum
Chemical Shift (ppm)

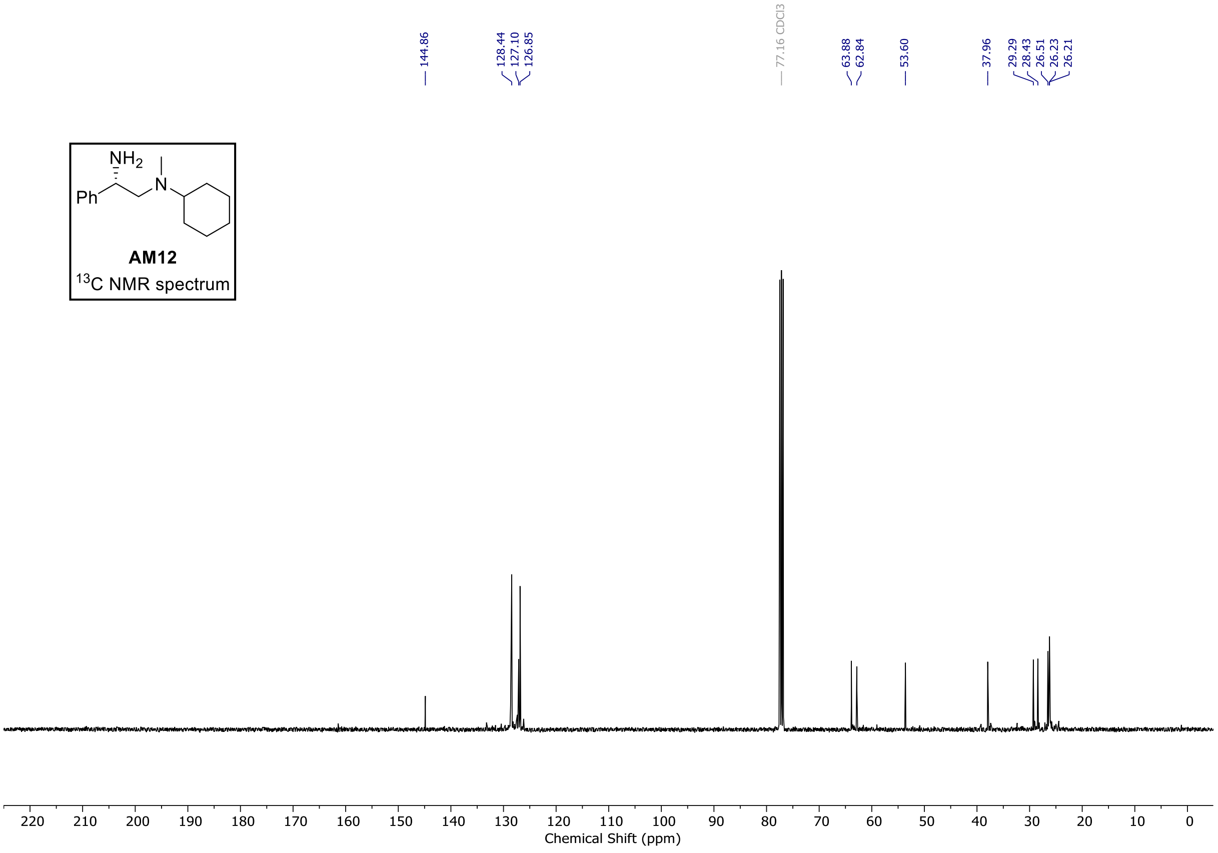
NH2
Ph
N
AM12
13C NMR spectrum
Chemical Shift (ppm)

16. NMR spectra of phosphoric acids and intermediates

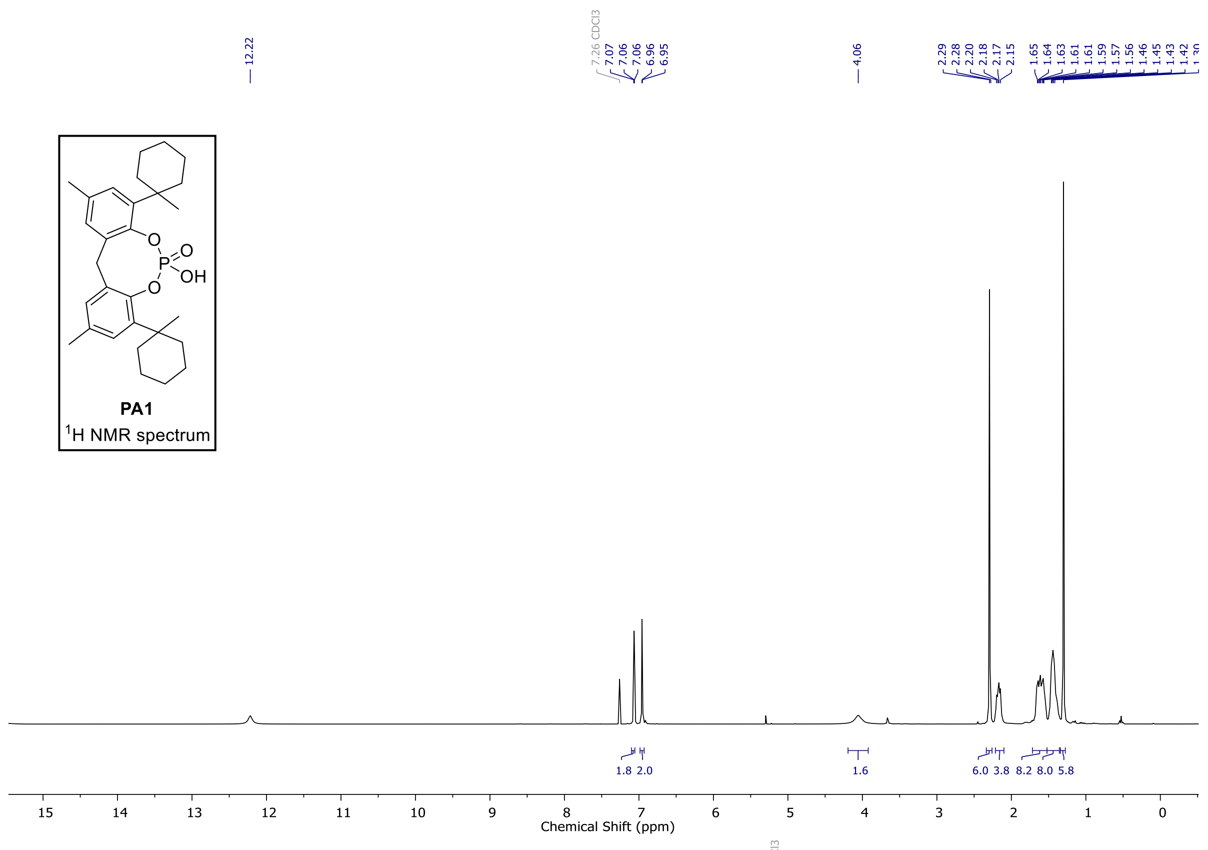

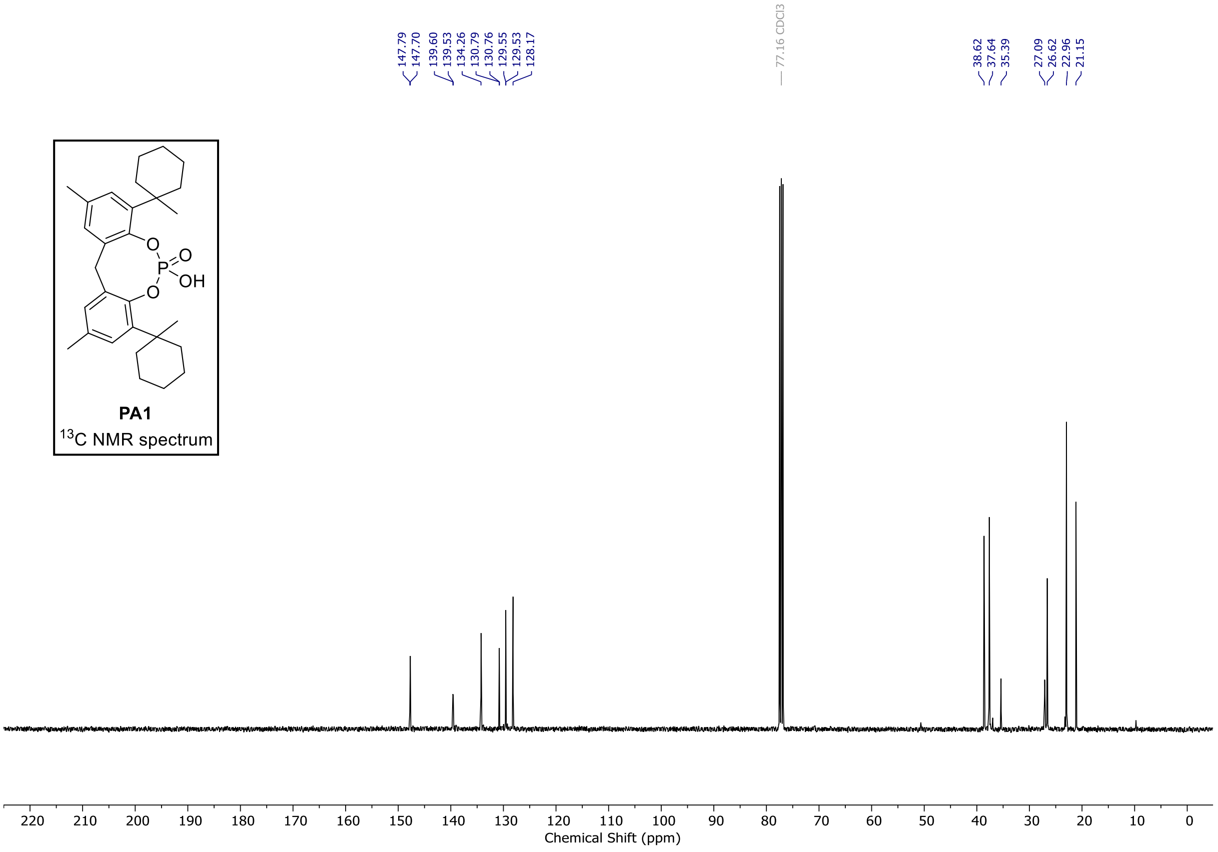

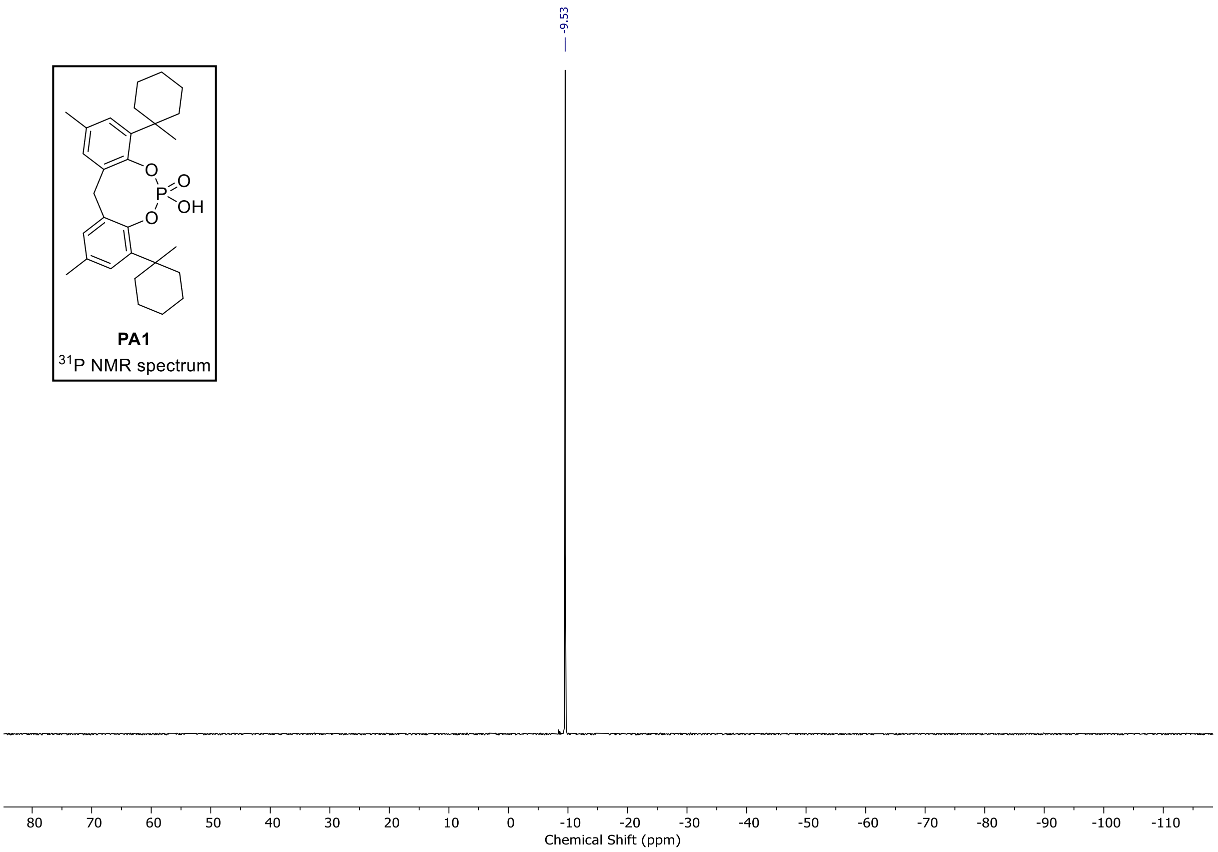

-9.53
PA1
31P NMR spectrum
80 70 60 50 40 30 20 10 0 -10 -20 -30 -40 -50 -60 -70 -80 -90 -100 -110
Chemical Shift (ppm)

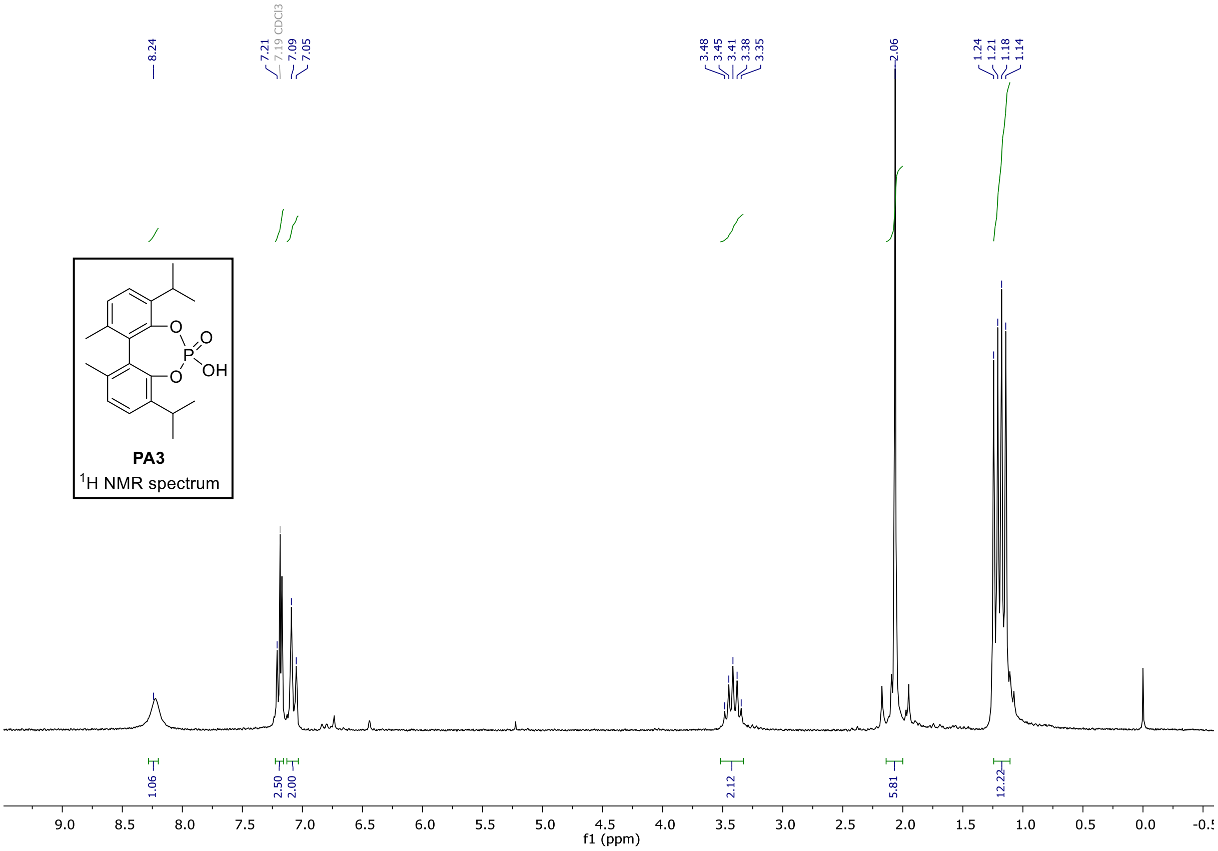

8.24
7.21
7.19 CDCl3
7.09
7.05
3.48
3.45
3.41
3.38
3.35
2.06
1.24
1.21
1.18
1.14
PA3
1H NMR spectrum
1.06
2.50
2.00
2.12
5.81
12.22
9.0 8.5 8.0 7.5 7.0 6.5 6.0 5.5 5.0 4.5 4.0 3.5 3.0 2.5 2.0 1.5 1.0 0.5 0.0
f1 (ppm)

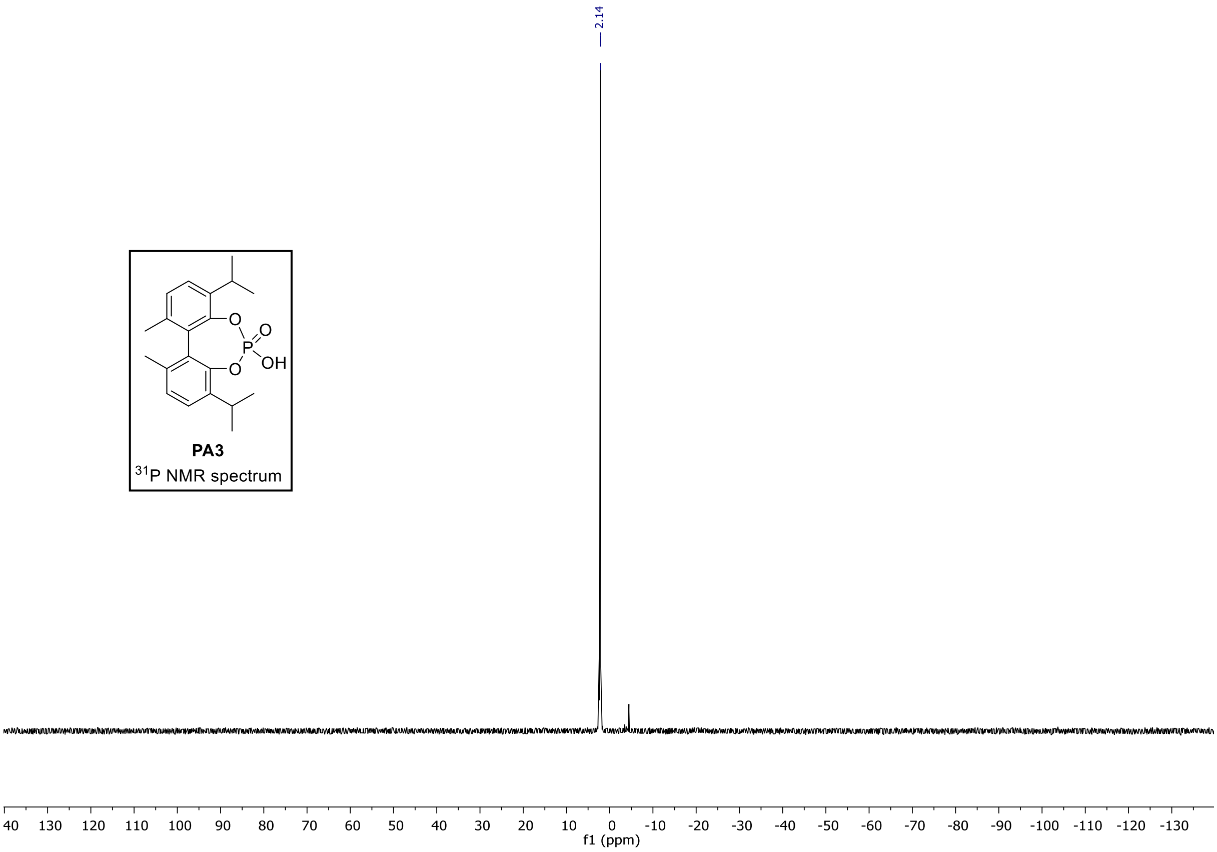
2.14
O
P
O
OH
O
PA3
31P NMR spectrum
40 130 120 110 100 90 80 70 60 50 40 30 20 10 0 -10 -20 -30 -40 -50 -60 -70 -80 -90 -100 -110 -120 -130
f1 (ppm)

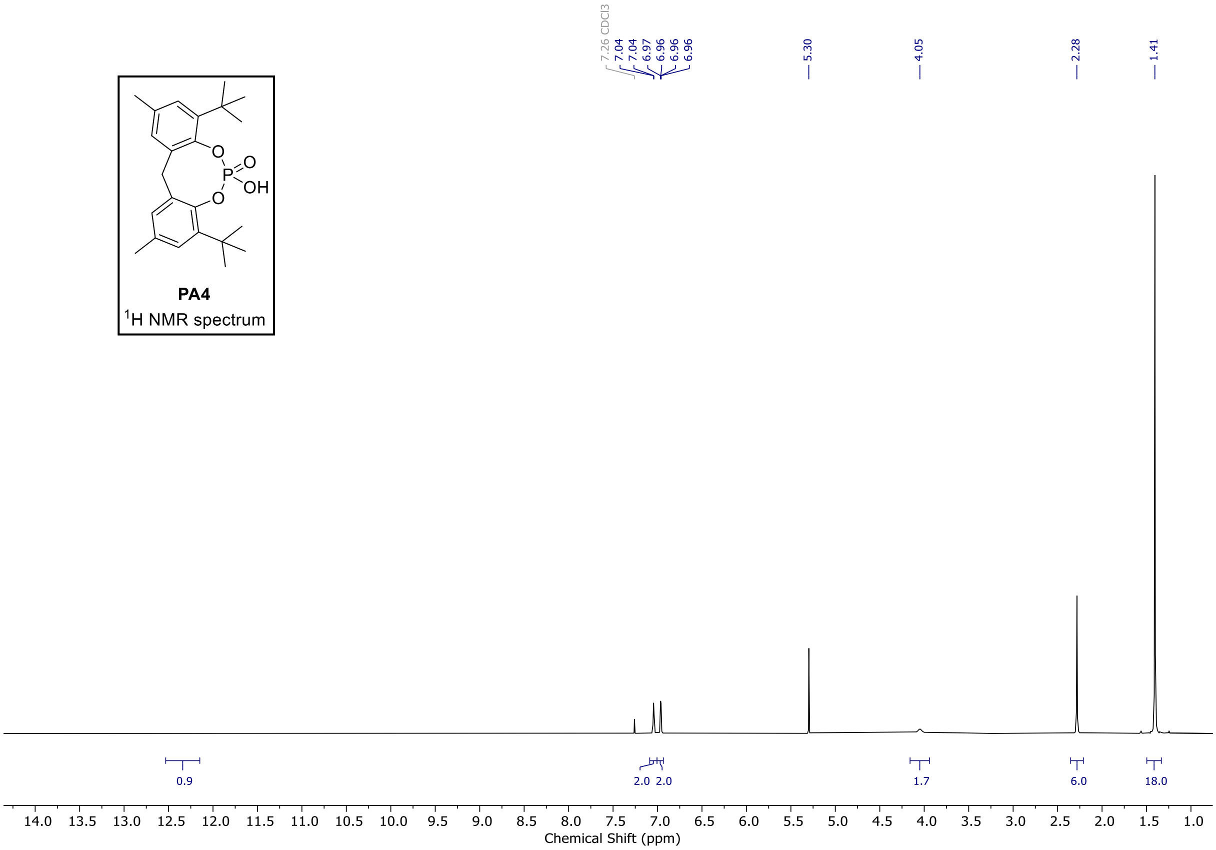
7.26 CDCl3
7.04
7.04
6.97
6.96
6.96
6.96
5.30
4.05
2.28
1.41
O
P
O
OH
O
PA4
1H NMR spectrum
0.9
2.0 2.0
1.7
6.0
18.0
14.0 13.5 13.0 12.5 12.0 11.5 11.0 10.5 10.0 9.5 9.0 8.5 8.0 7.5 7.0 6.5 6.0 5.5 5.0 4.5 4.0 3.5 3.0 2.5 2.0 1.5 1.0
Chemical Shift (ppm)

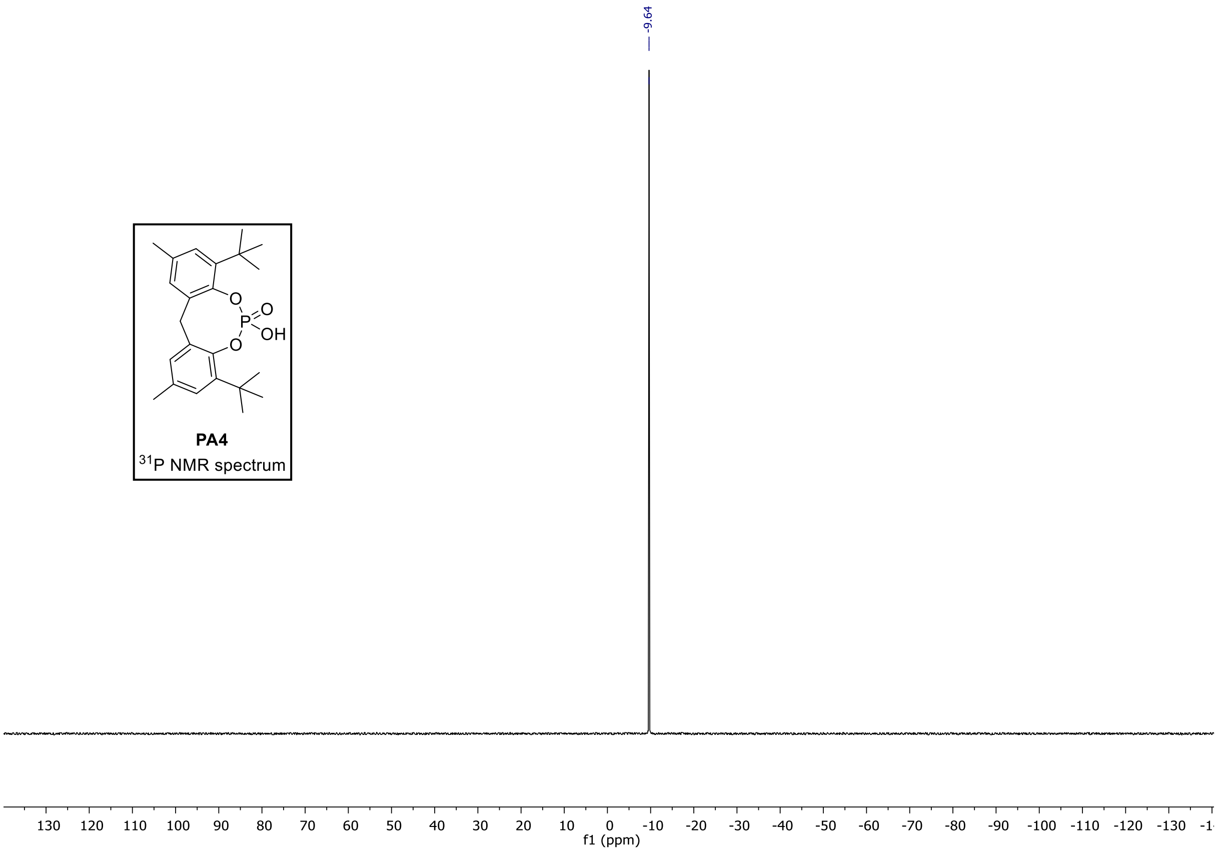

-9.64
O
P
O
OH
O
PA4
31P NMR spectrum
130 120 110 100 90 80 70 60 50 40 30 20 10 0 -10 -20 -30 -40 -50 -60 -70 -80 -90 -100 -110 -120 -130
f1 (ppm)

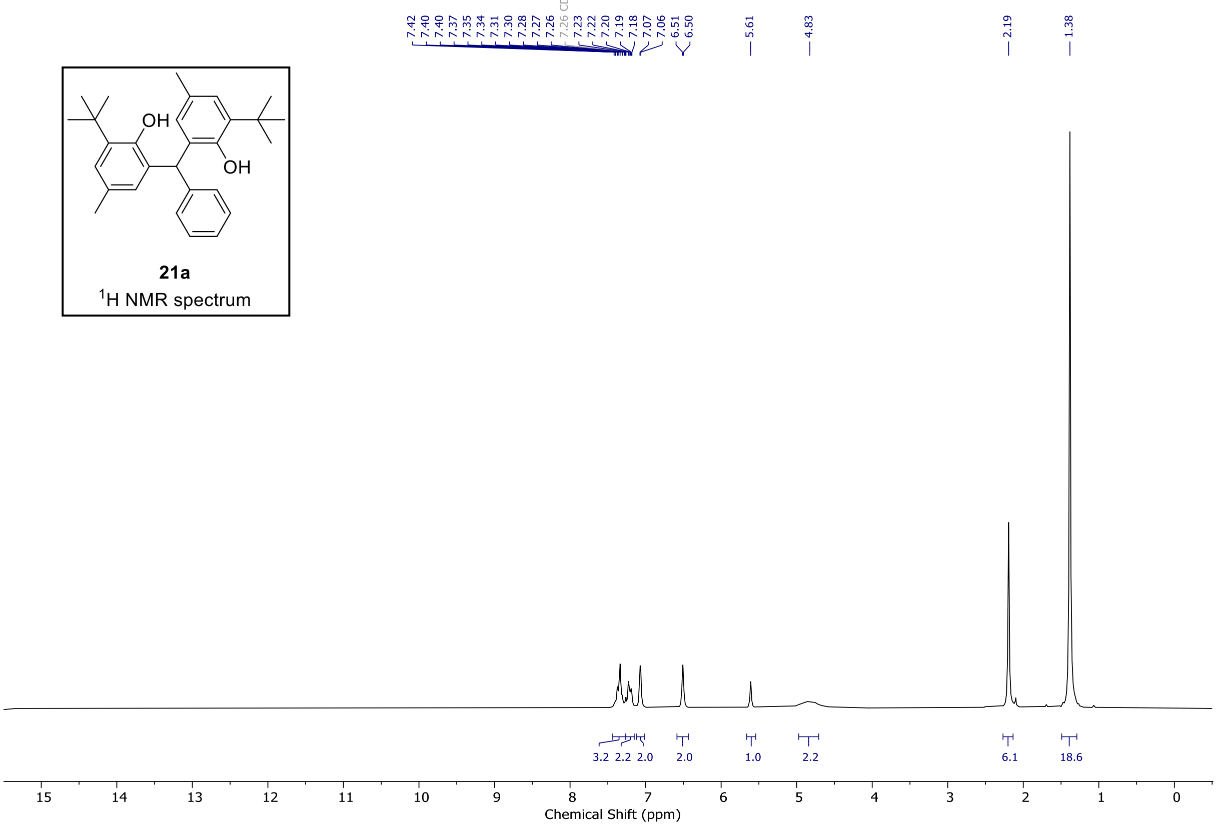

7.42 7.40 7.40 7.37 7.35 7.34 7.31 7.30 7.28 7.27 7.26 7.26 CDCl3 7.23 7.22 7.20 7.19 7.18 7.07 7.06 6.51 6.50
5.61
4.83
2.19
1.38
OH
OH
21a
1H NMR spectrum
3.2 2.2 2.0
2.0
1.0
2.2
6.1
18.6
15 14 13 12 11 10 9 8 7 6 5 4 3 2 1 0
Chemical Shift (ppm)

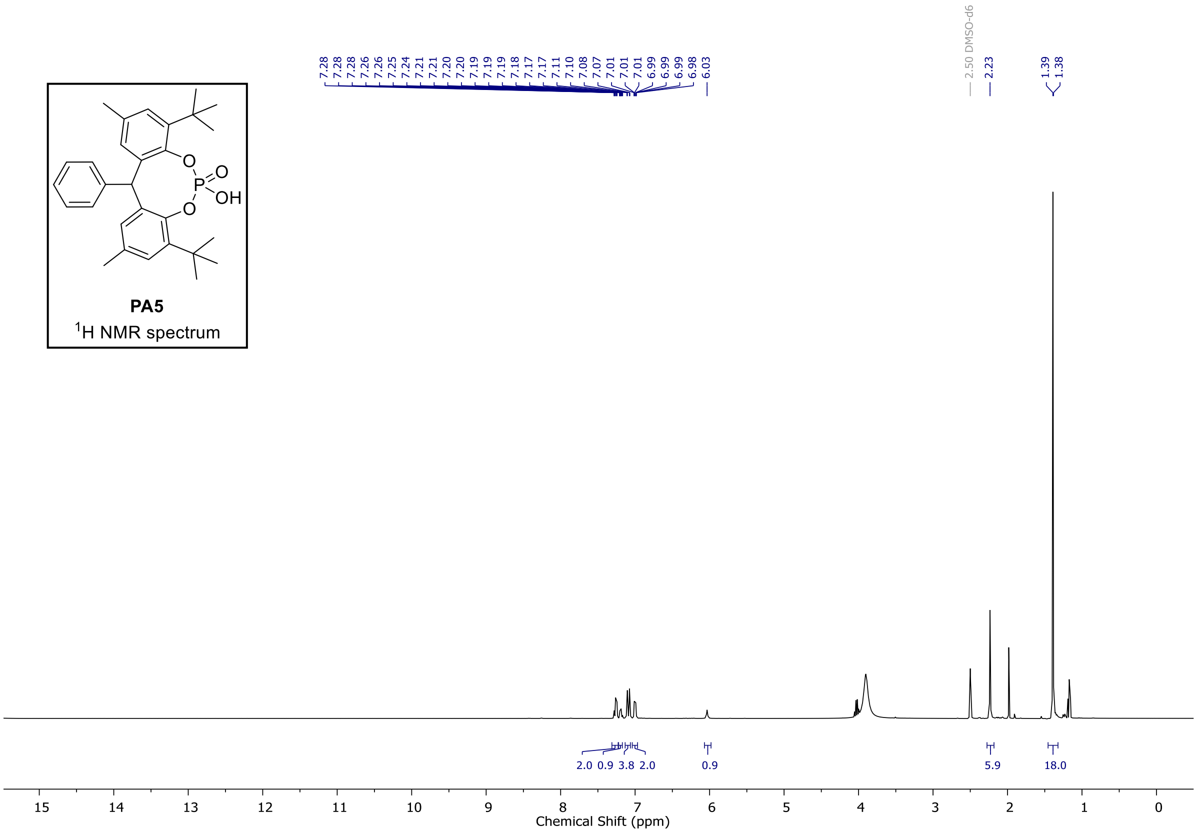
PA5
1H NMR spectrum
7.28 7.28 7.28 7.26 7.26 7.25 7.24 7.21 7.21 7.20 7.20 7.19 7.19 7.19 7.18 7.17 7.17 7.11 7.10 7.08 7.07 7.01 7.01 7.01 6.99 6.99 6.99 6.98 6.03
2.50 DMSO-d6
2.23
1.39 1.38
2.0 0.9 3.8 2.0
0.9
5.9
18.0
Chemical Shift (ppm)

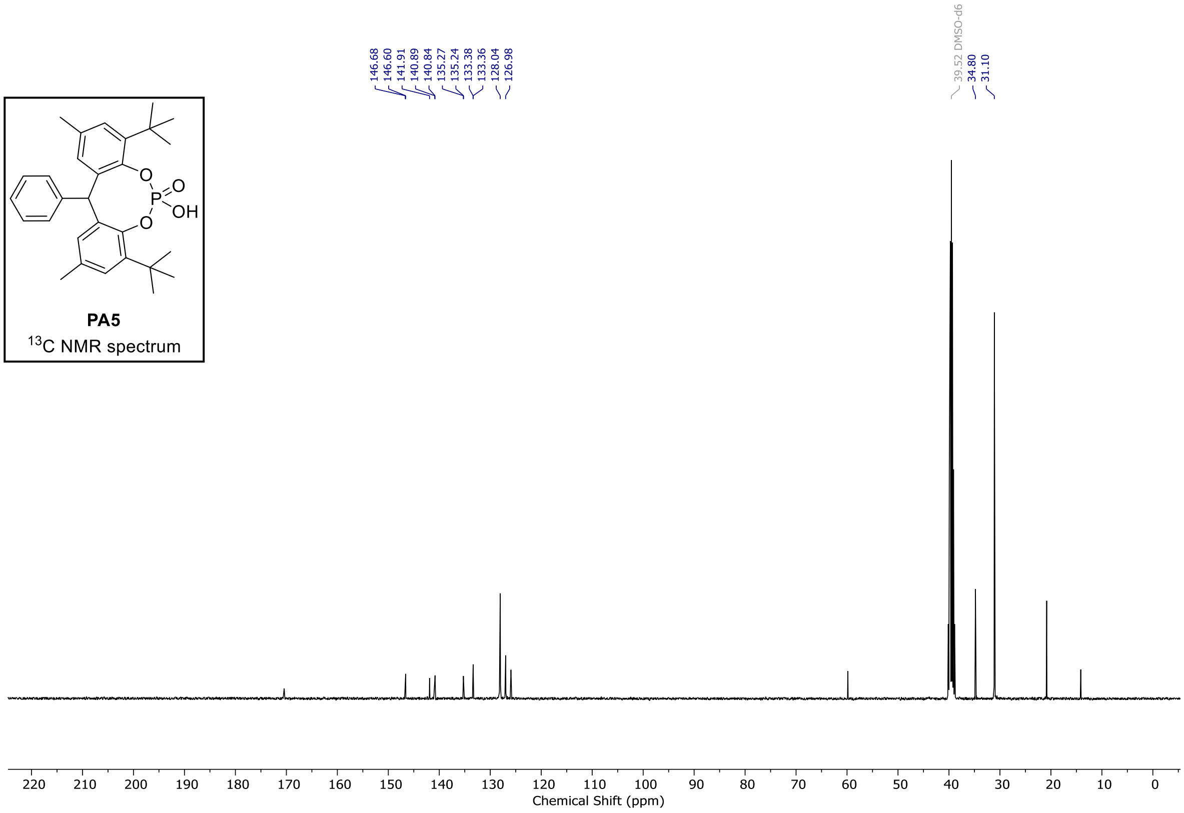
PA5
13C NMR spectrum
146.68 146.60 141.91 140.89 140.84 135.27 135.24 133.38 133.36 128.04 126.98
39.52 DMSO-d6
34.80
31.10
Chemical Shift (ppm)

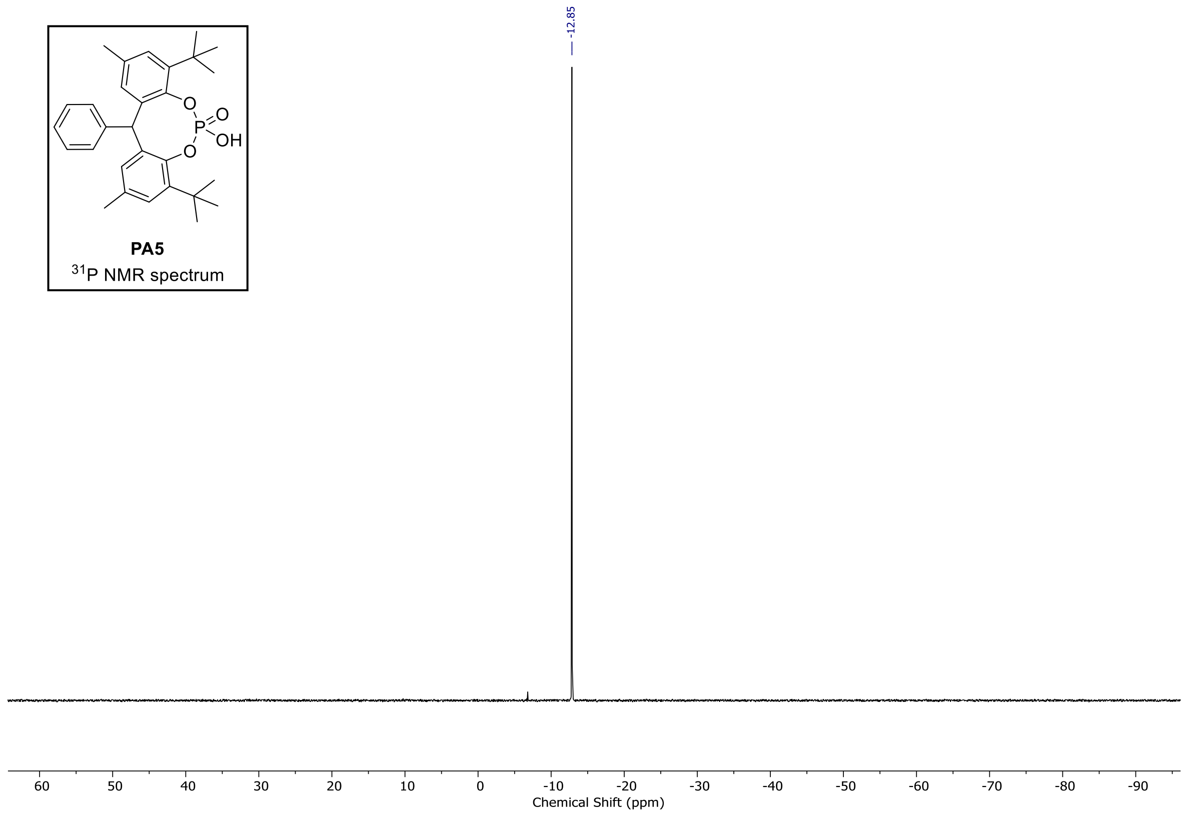
-12.85
PA5
31P NMR spectrum
60 50 40 30 20 10 0 -10 -20 -30 -40 -50 -60 -70 -80 -90
Chemical Shift (ppm)

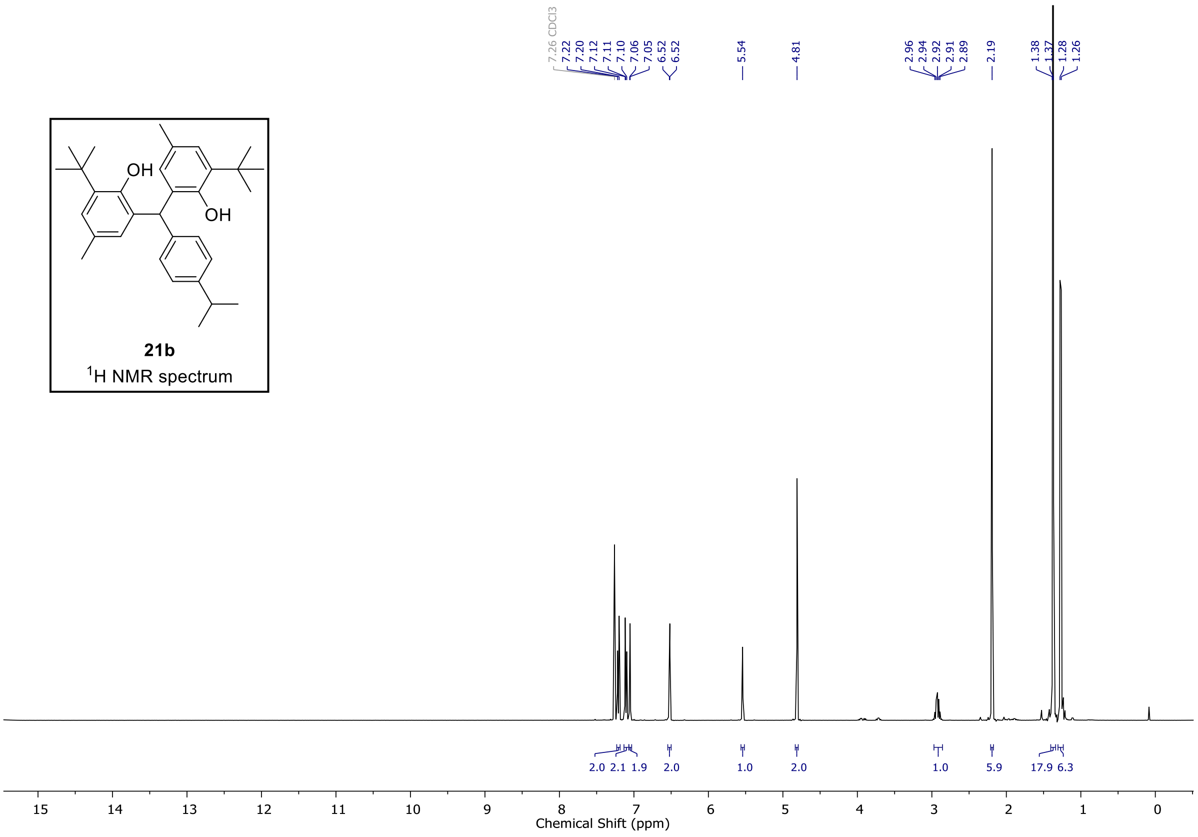
7.26 CDCl3
7.22 7.20 7.12 7.11 7.10 7.06 7.05 6.52 6.52
5.54
4.81
2.96 2.94 2.92 2.91 2.89
2.19
1.38 1.37 1.28 1.26
OH
OH
21b
1H NMR spectrum
2.0 2.1 1.9 2.0 1.0 2.0 1.0 5.9 17.9 6.3
15 14 13 12 11 10 9 8 7 6 5 4 3 2 1 0
Chemical Shift (ppm)

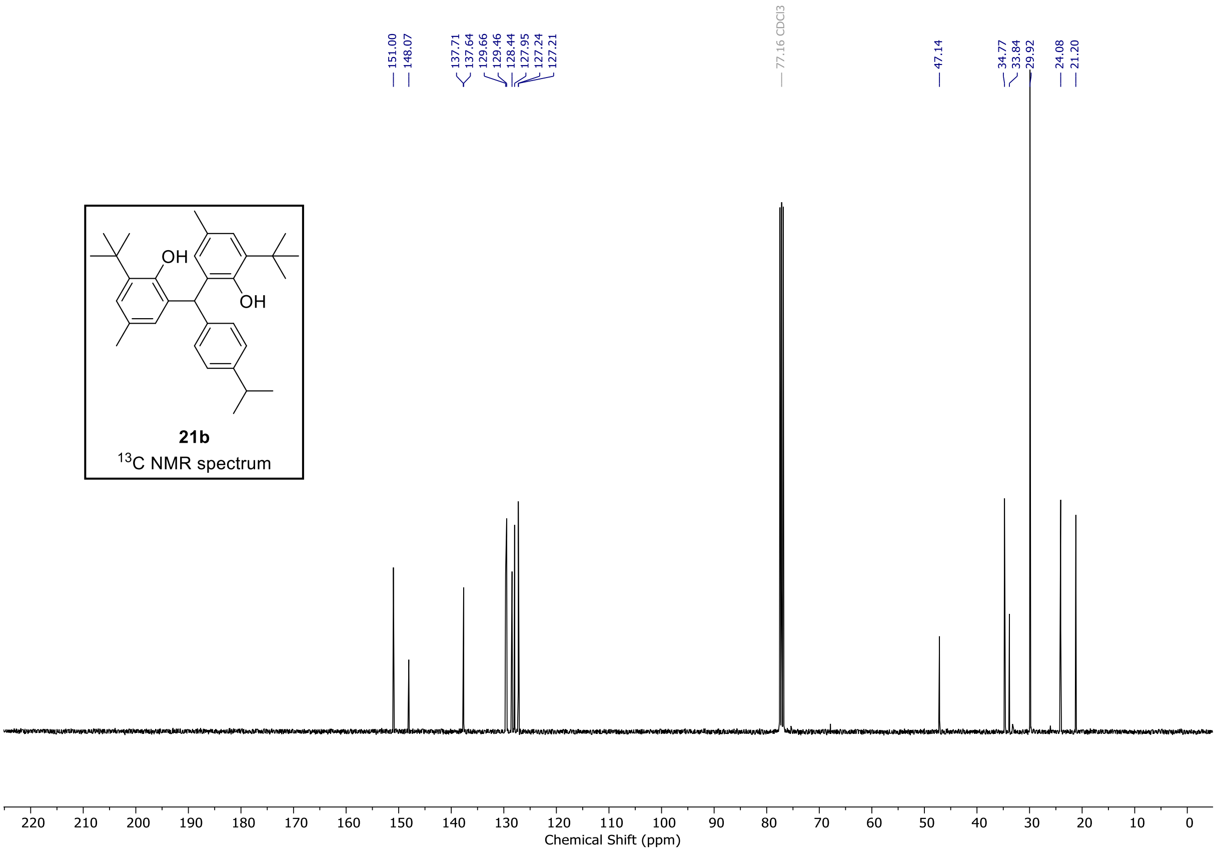
151.00
148.07
137.71
137.64
129.66
129.46
128.44
127.95
127.24
127.21
77.16 CDCl3
47.14
34.77
33.84
29.92
24.08
21.20
OH
OH
21b
13C NMR spectrum
Chemical Shift (ppm)

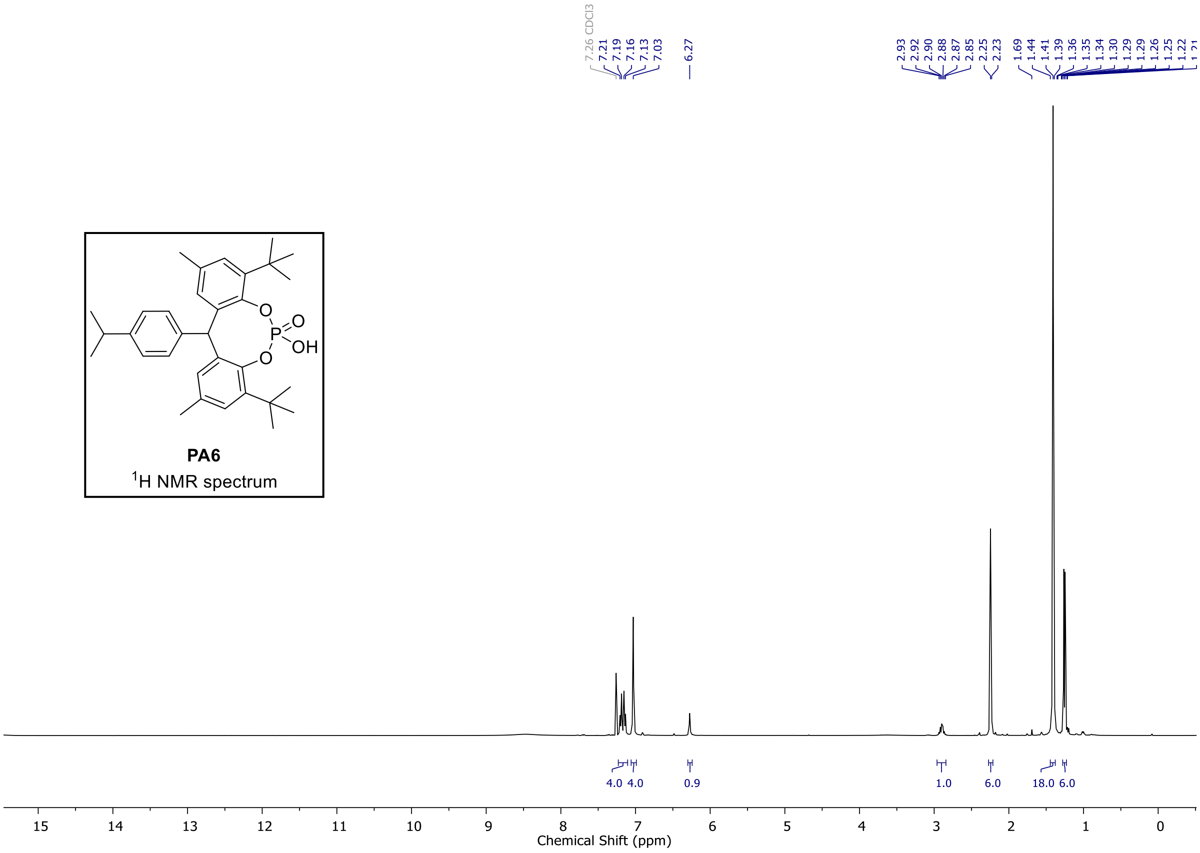
7.26 CDCl3
7.21
7.19
7.16
7.13
7.03
6.27
2.93
2.92
2.90
2.88
2.87
2.85
2.25
2.23
1.69
1.44
1.41
1.39
1.36
1.35
1.34
1.30
1.29
1.29
1.26
1.25
1.22
O
P
O
OH
PA6
1H NMR spectrum
4.0
4.0
0.9
1.0
6.0
18.0
6.0
Chemical Shift (ppm)

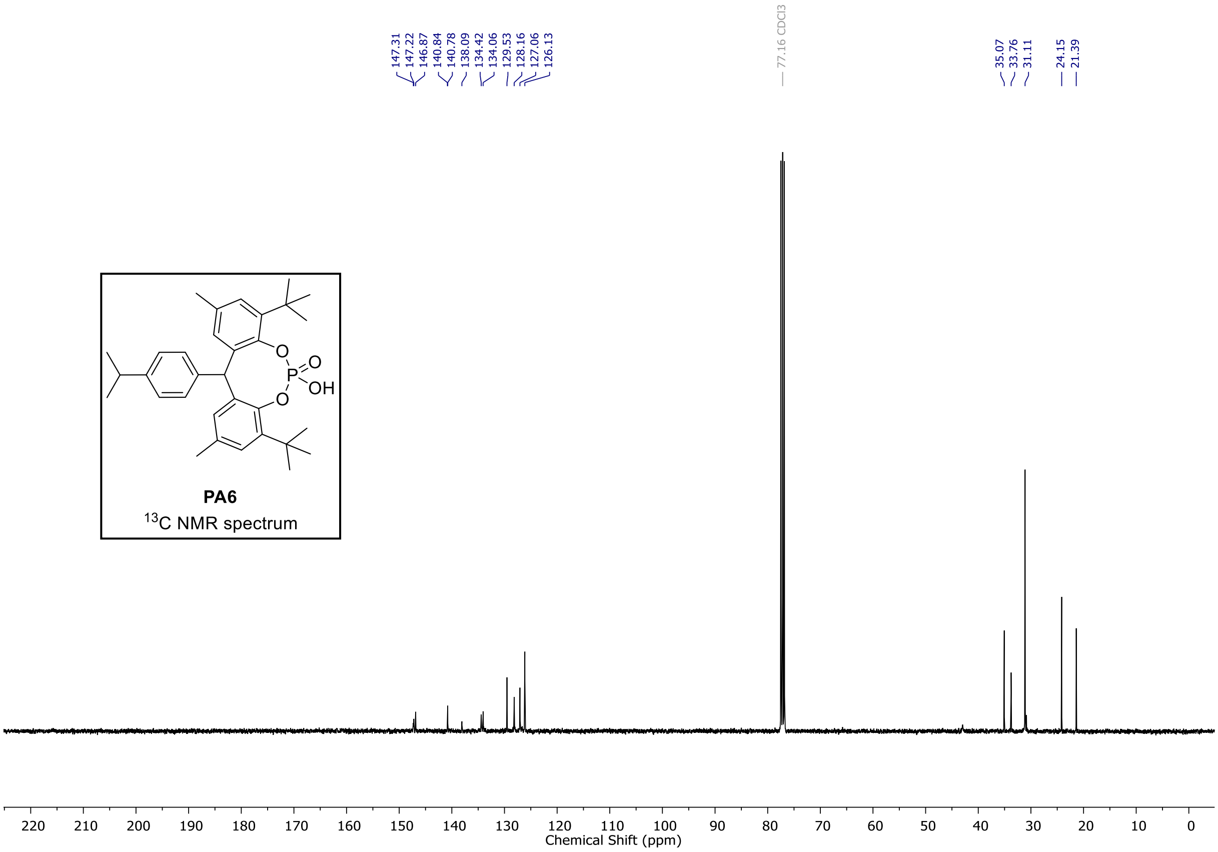
147.31
147.22
146.87
140.84
140.78
138.09
134.42
134.06
129.53
128.16
127.06
126.13
77.16 CDCl3
35.07
33.76
31.11
24.15
21.39
O
P
O
OH
O
PA6
13C NMR spectrum
Chemical Shift (ppm)

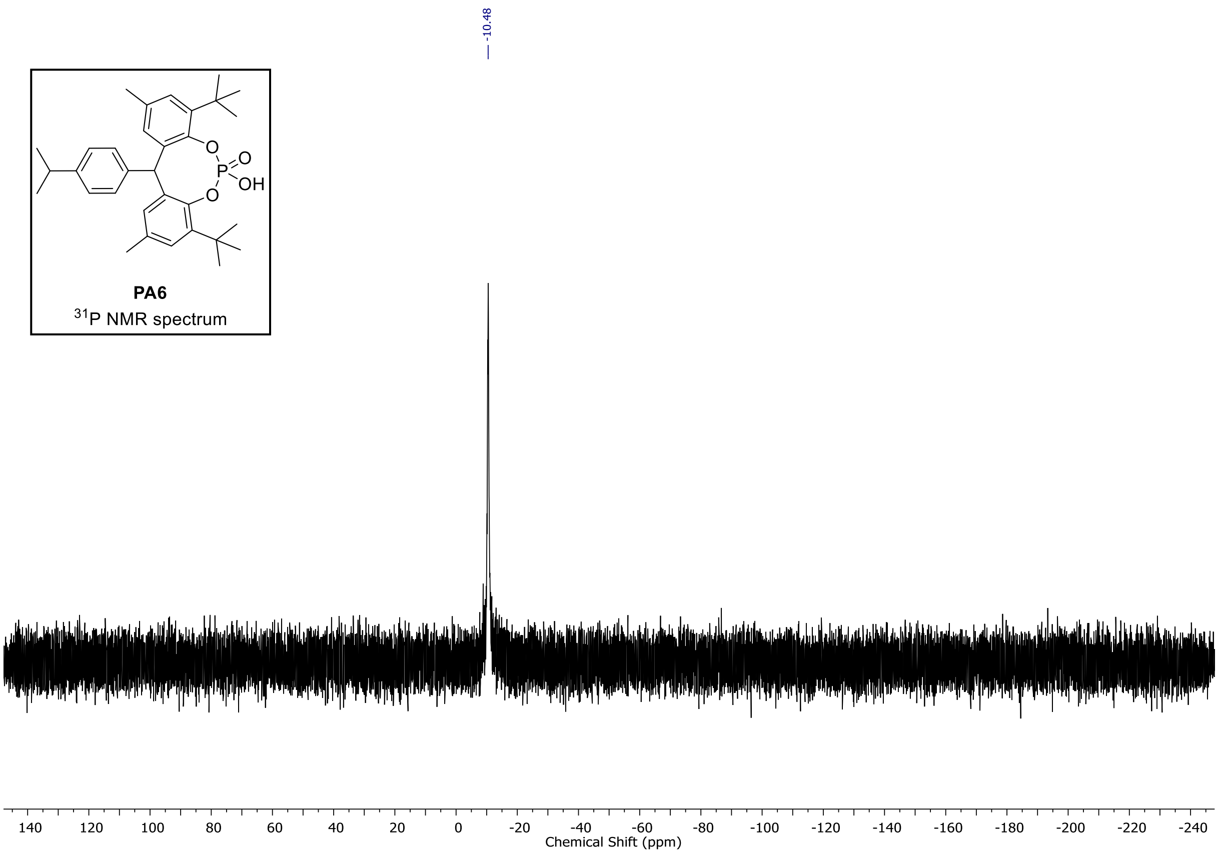
-10.48
O
P
O
OH
O
PA6
31P NMR spectrum
Chemical Shift (ppm)

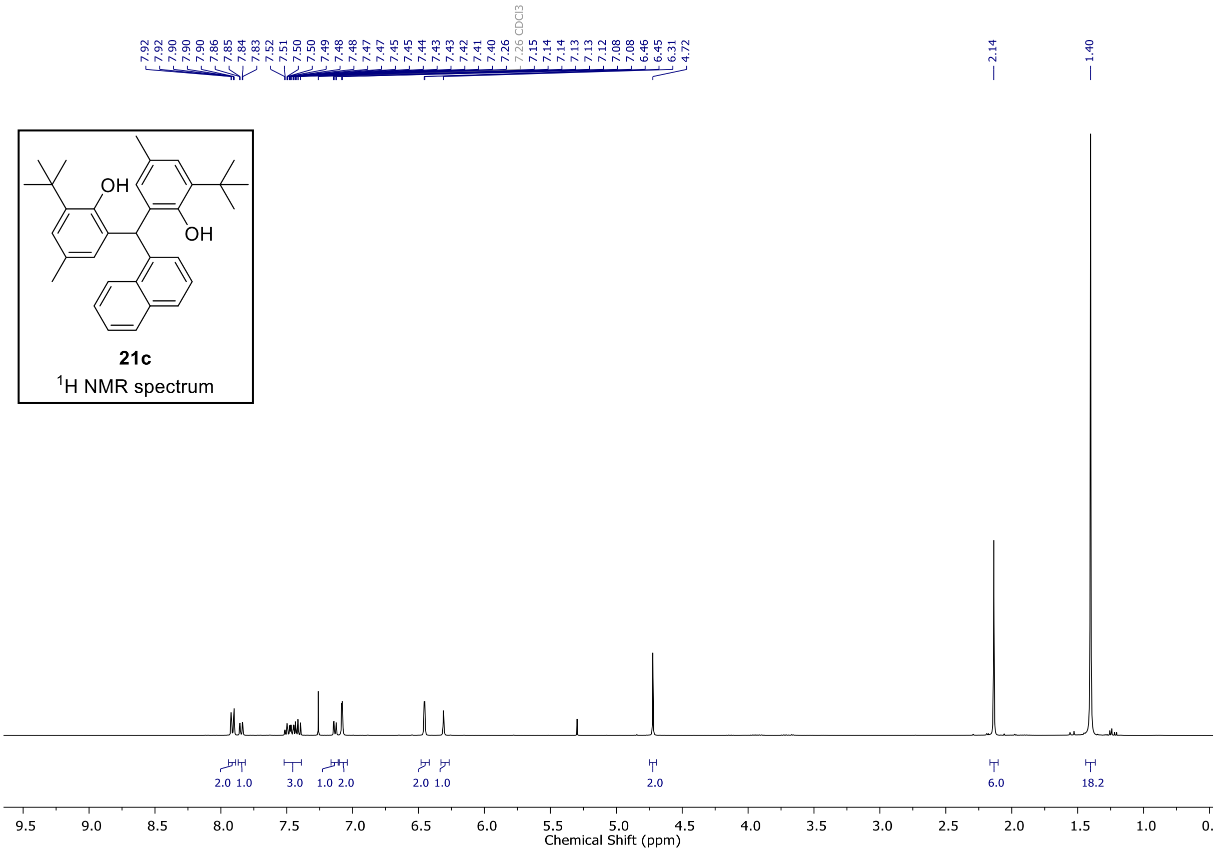
7.92
7.92
7.90
7.90
7.90
7.86
7.85
7.84
7.83
7.52
7.51
7.50
7.50
7.49
7.48
7.48
7.47
7.47
7.45
7.45
7.44
7.43
7.43
7.42
7.41
7.40
7.26 CDCl3
7.15
7.14
7.14
7.13
7.13
7.12
7.08
7.08
6.46
6.45
6.31
4.72
2.14
1.40
OH
OH
21c
1H NMR spectrum
2.0 1.0
3.0
1.0 2.0
2.0 1.0
2.0
6.0
18.2
Chemical Shift (ppm)

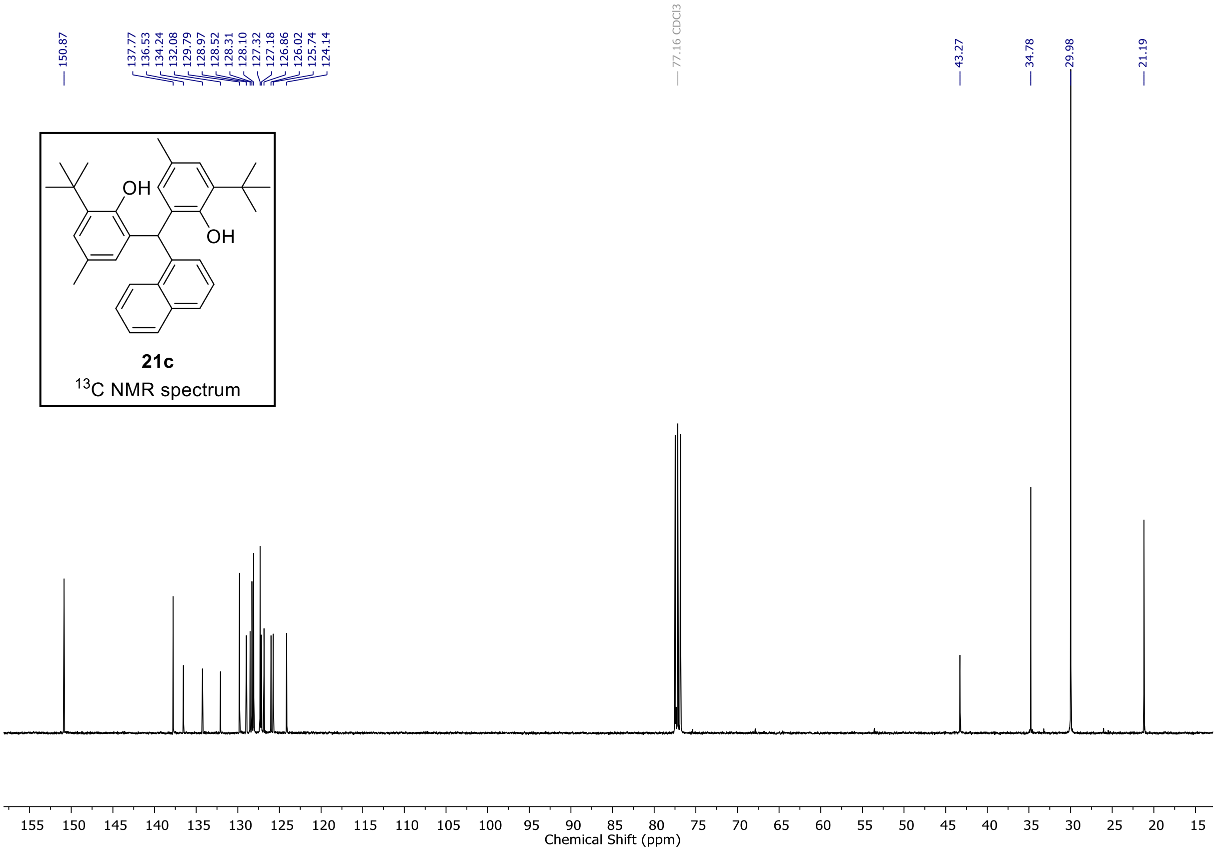
150.87
137.77
136.53
134.24
132.08
129.79
128.97
128.52
128.31
128.10
127.32
127.18
126.86
126.02
125.74
124.14
77.16 CDCl3
43.27
34.78
29.98
21.19
OH
OH
21c
13C NMR spectrum
Chemical Shift (ppm)

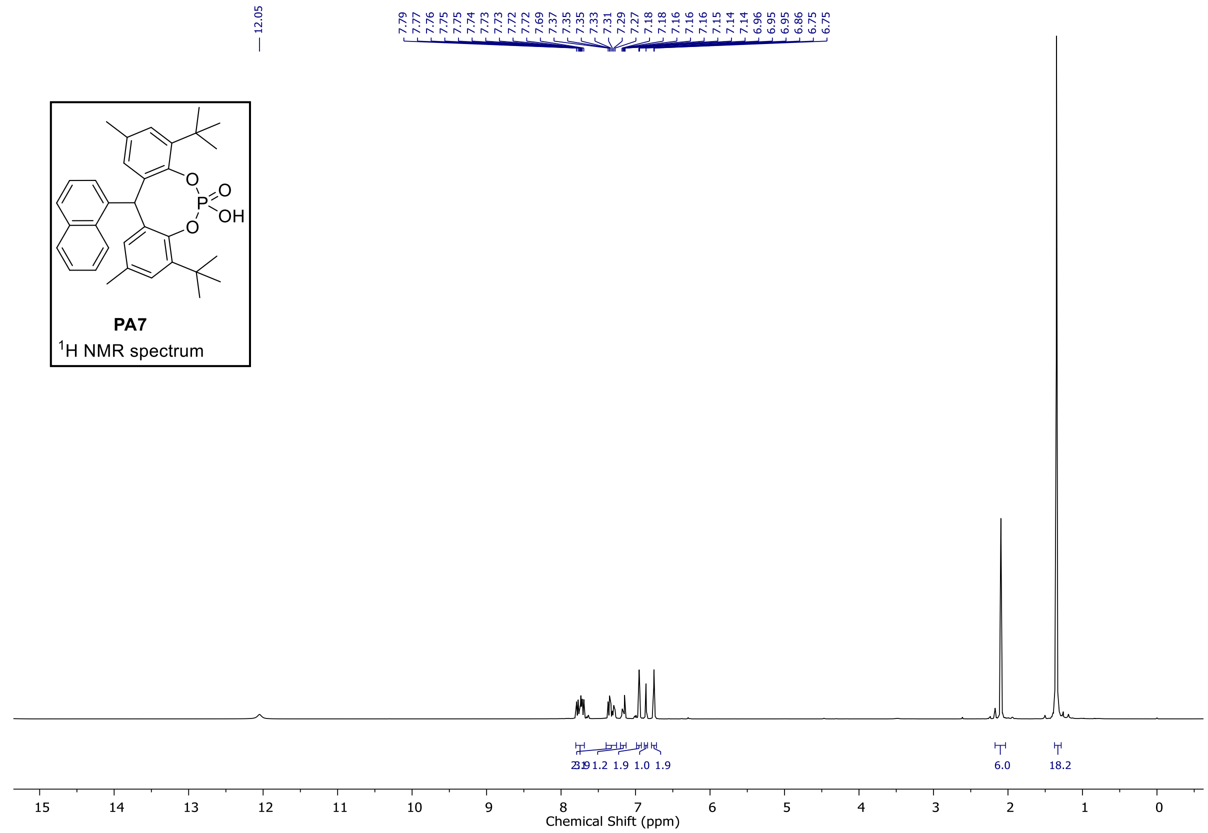
12.05
7.79 7.77 7.76 7.75 7.75 7.74 7.73 7.73 7.72 7.72 7.69 7.37 7.35 7.35 7.33 7.31 7.29 7.27 7.18 7.18 7.16 7.16 7.15 7.14 7.14 6.96 6.95 6.95 6.86 6.75 6.75
O
P
O
OH
O
PA7
^{1}H NMR spectrum
1.2 1.9 1.0 1.9
6.0
18.2
Chemical Shift (ppm)

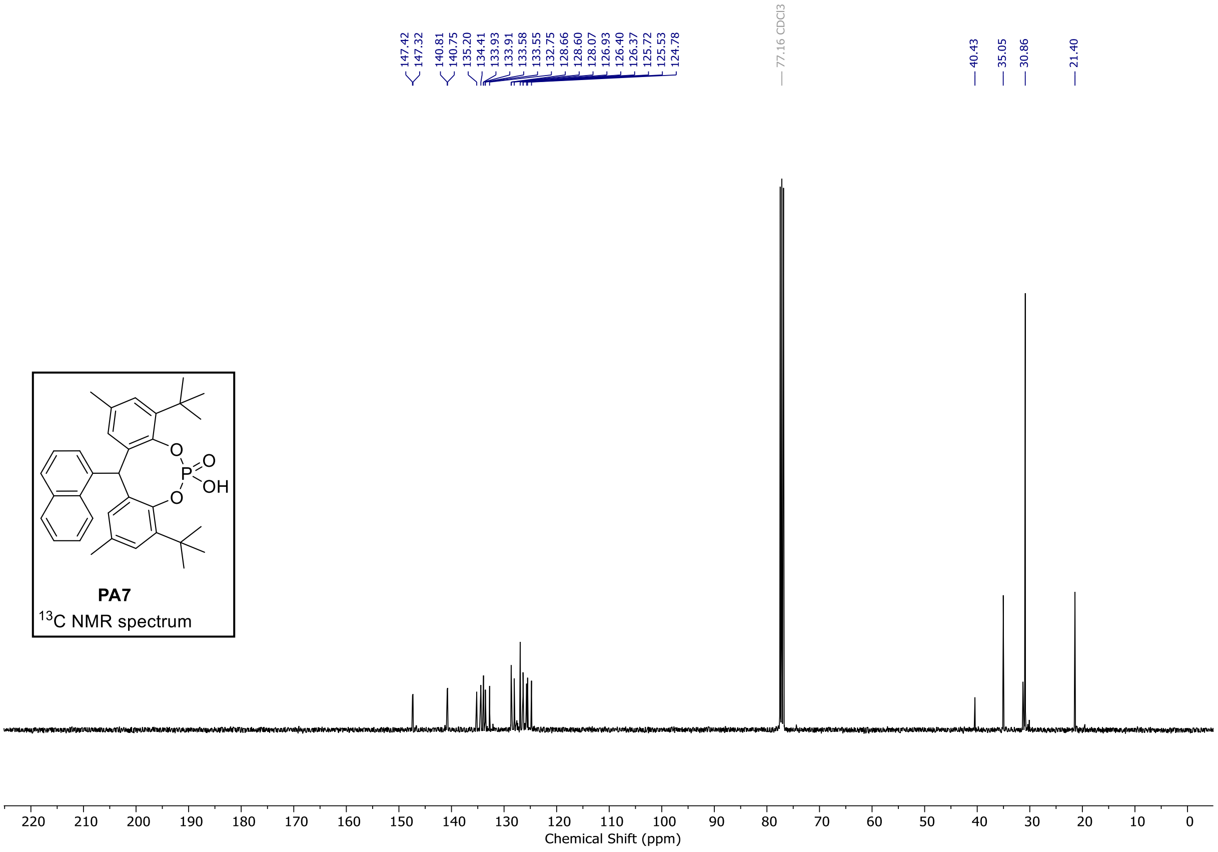
147.42 147.32 140.81 140.75 135.20 134.41 133.93 133.91 133.58 133.55 132.75 128.66 128.60 128.07 126.93 126.40 126.37 125.72 125.53 124.78
77.16 CDCl3
40.43 35.05 30.86 21.40
O
P
O
OH
O
PA7
^{13}C NMR spectrum
Chemical Shift (ppm)

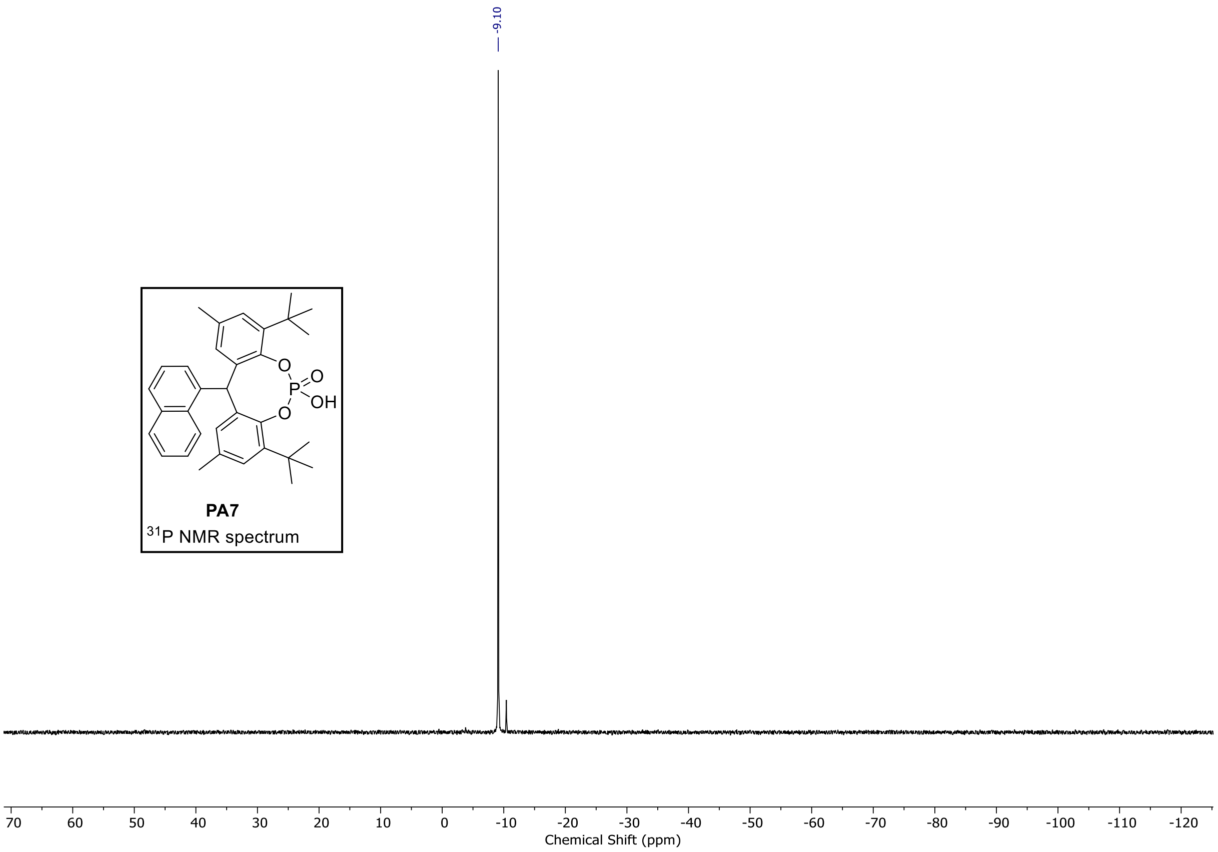
-9.10
PA7
31P NMR spectrum
70 60 50 40 30 20 10 0 -10 -20 -30 -40 -50 -60 -70 -80 -90 -100 -110 -120
Chemical Shift (ppm)

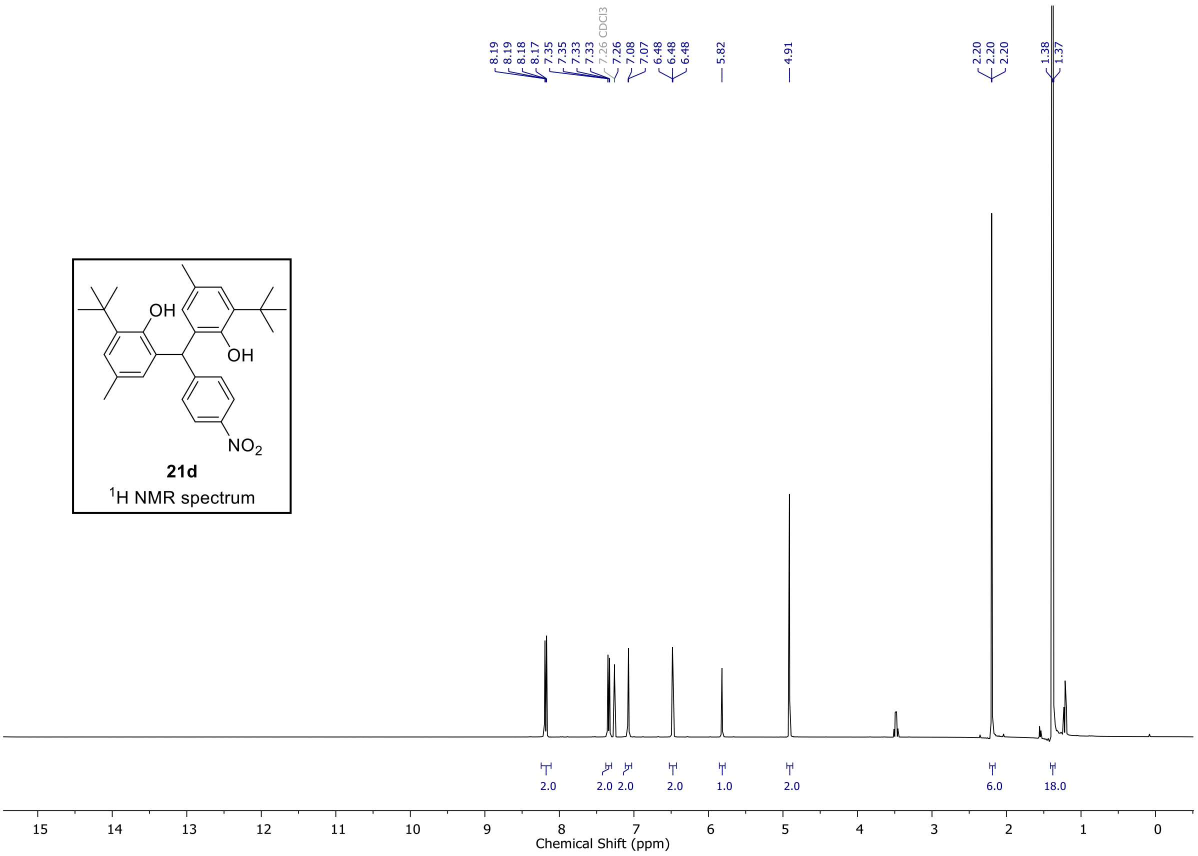
8.19 8.19 8.18 8.17 7.35 7.35 7.33 7.33 7.26 CDCl3 7.26 7.08 7.07 6.48 6.48 6.48 5.82 4.91 2.20 2.20 2.20 1.38 1.37
OH
OH
NO2
21d
1H NMR spectrum
2.0 2.0 2.0 2.0 1.0 2.0 6.0 18.0
15 14 13 12 11 10 9 8 7 6 5 4 3 2 1 0
Chemical Shift (ppm)

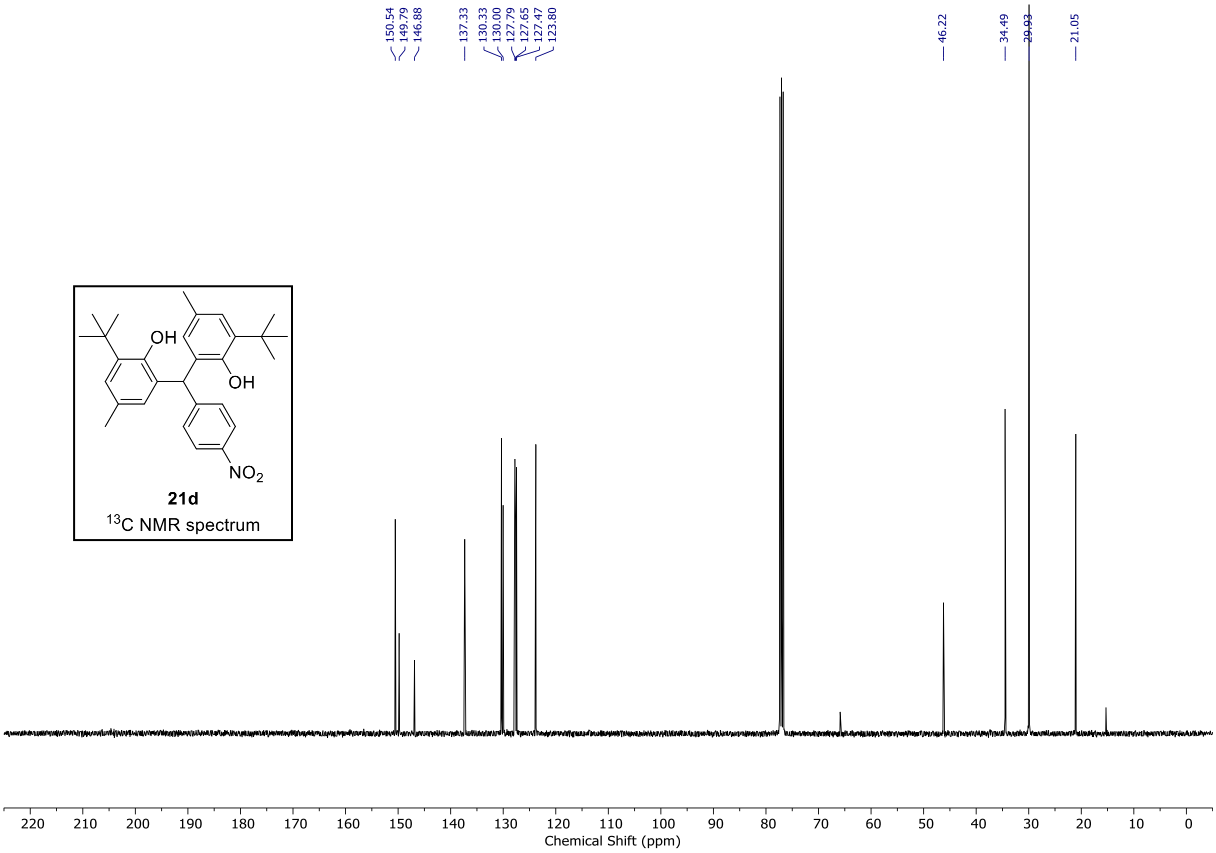
21d
^{13}C NMR spectrum
Chemical Shift (ppm)

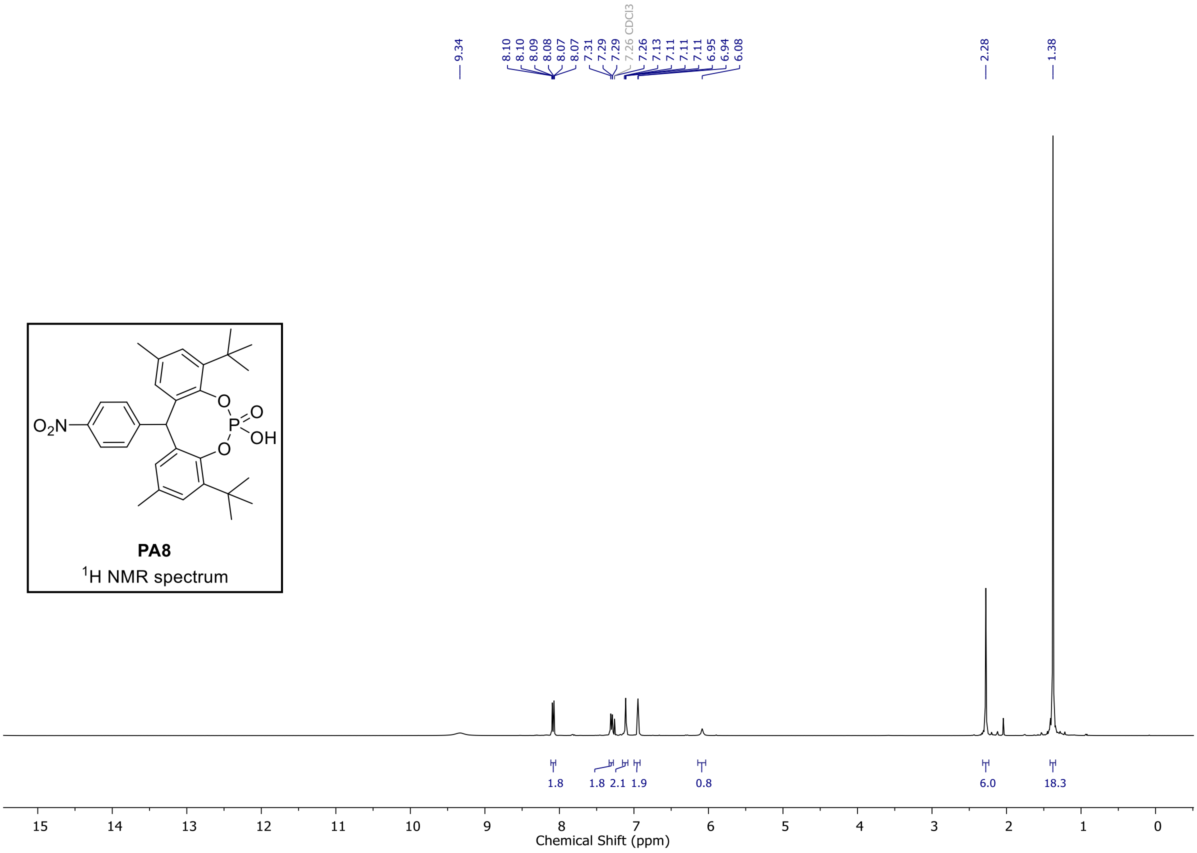
PA8
^{1}H NMR spectrum
Chemical Shift (ppm)

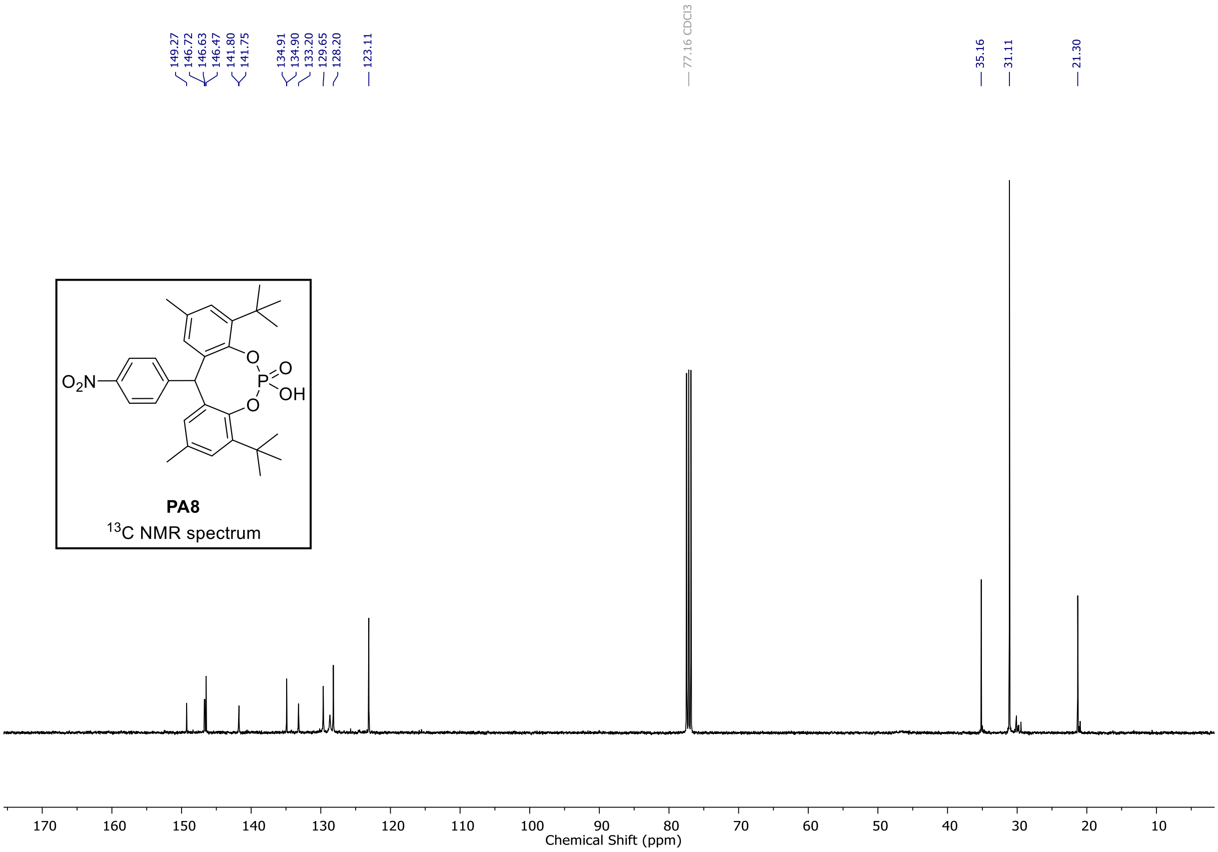
149.27
146.72
146.63
146.47
141.80
141.75
134.91
134.90
133.20
129.65
128.20
123.11
77.16 CDCl3
35.16
31.11
21.30
O_2N
O
P
O
OH
O
PA8
^{13}C NMR spectrum
Chemical Shift (ppm)

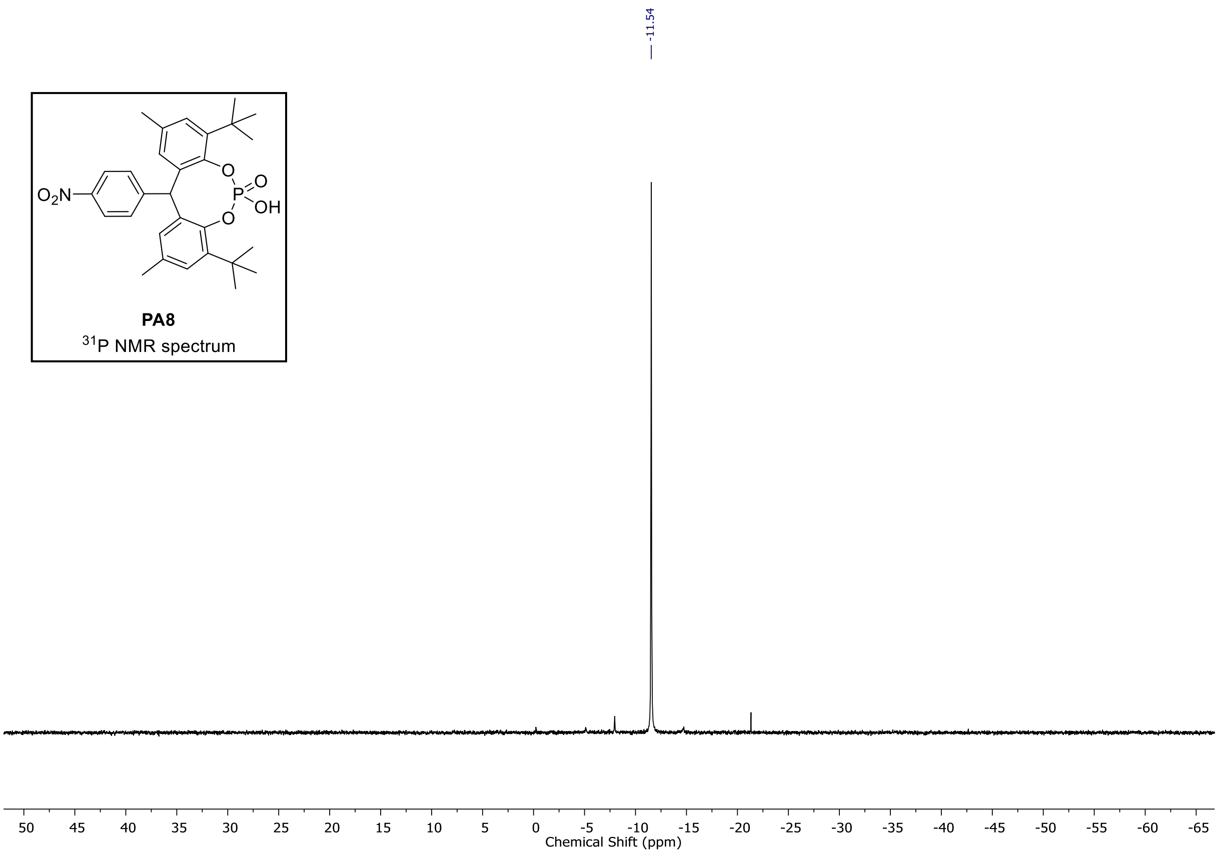
-11.54
O_2N
O
P
O
OH
O
PA8
^{31}P NMR spectrum
Chemical Shift (ppm)

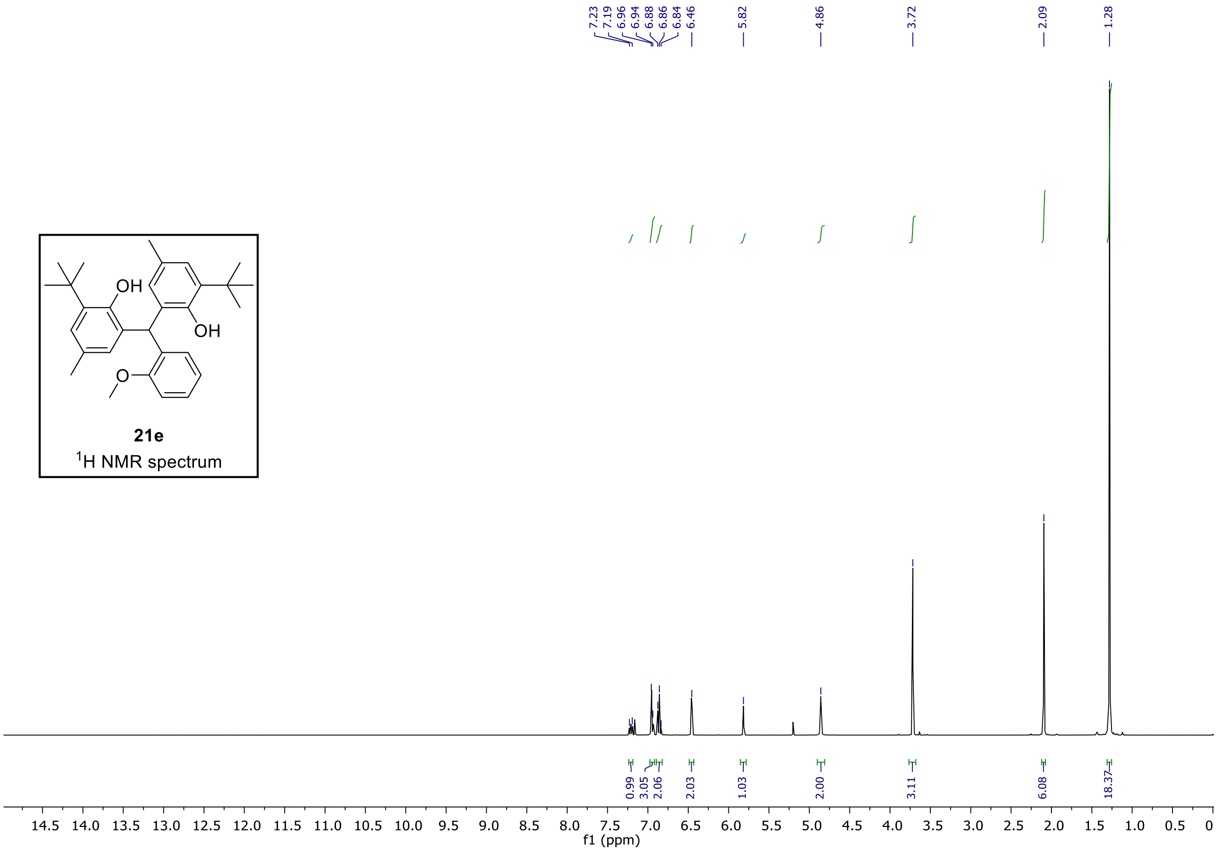
7.23
7.19
6.96
6.94
6.88
6.86
6.84
6.46
5.82
4.86
3.72
2.09
1.28
OH
OH
O
21e
1H NMR spectrum
0.99
3.05
2.06
2.03
1.03
2.00
3.11
6.08
18.37
14.5 14.0 13.5 13.0 12.5 12.0 11.5 11.0 10.5 10.0 9.5 9.0 8.5 8.0 7.5 7.0 6.5 6.0 5.5 5.0 4.5 4.0 3.5 3.0 2.5 2.0 1.5 1.0 0.5 0
f1 (ppm)

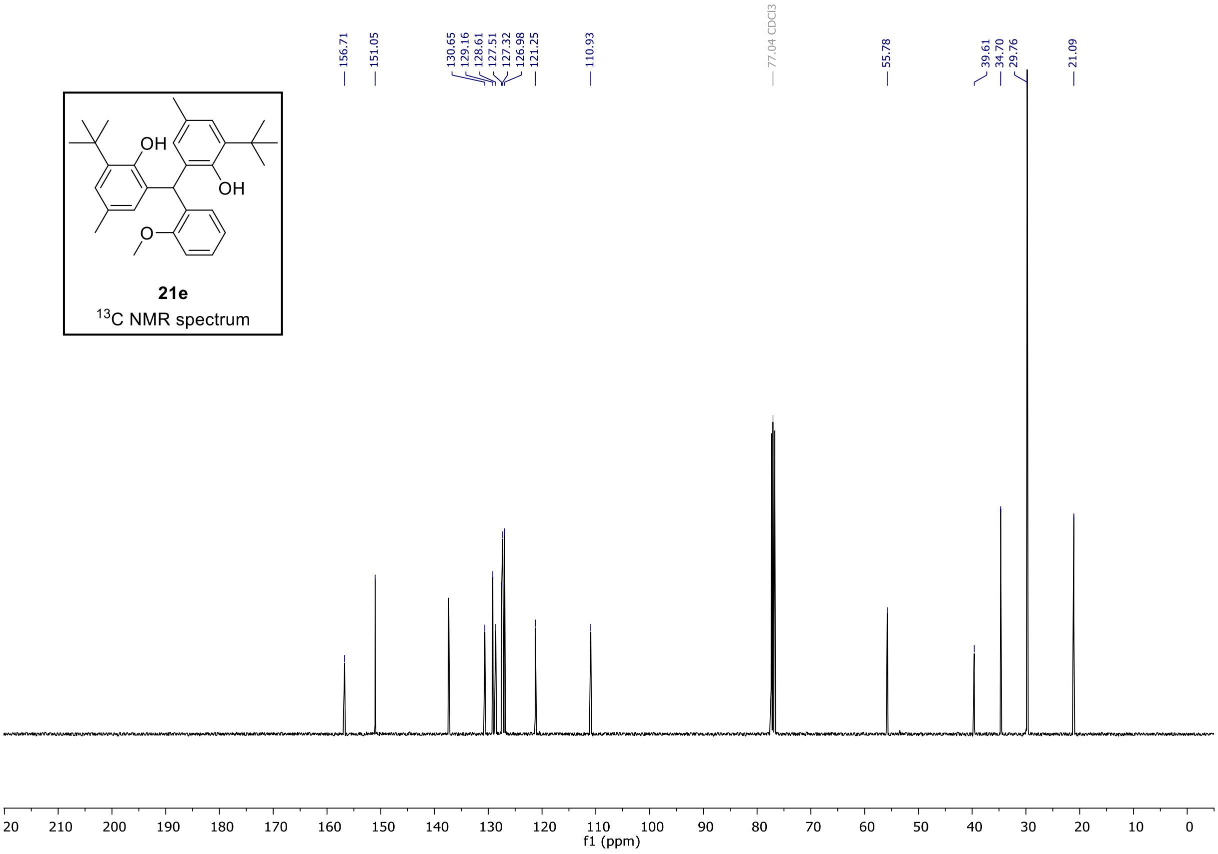
156.71
151.05
130.65
129.16
128.61
127.51
127.32
126.98
121.25
110.93
77.04 CDCl3
55.78
39.61
34.70
29.76
21.09
OH
OH
O
21e
13C NMR spectrum
20 210 200 190 180 170 160 150 140 130 120 110 100 90 80 70 60 50 40 30 20 10 0
f1 (ppm)

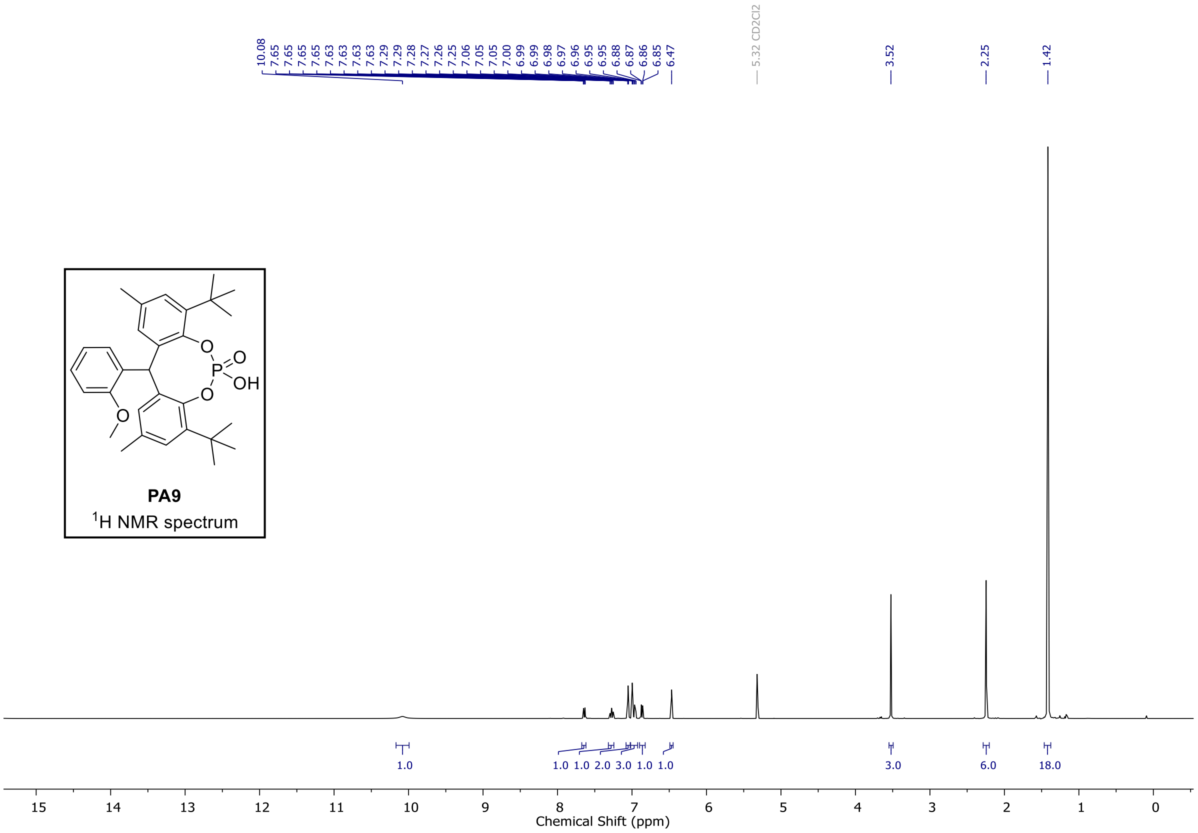
PA9
¹H NMR spectrum
Chemical Shift (ppm)

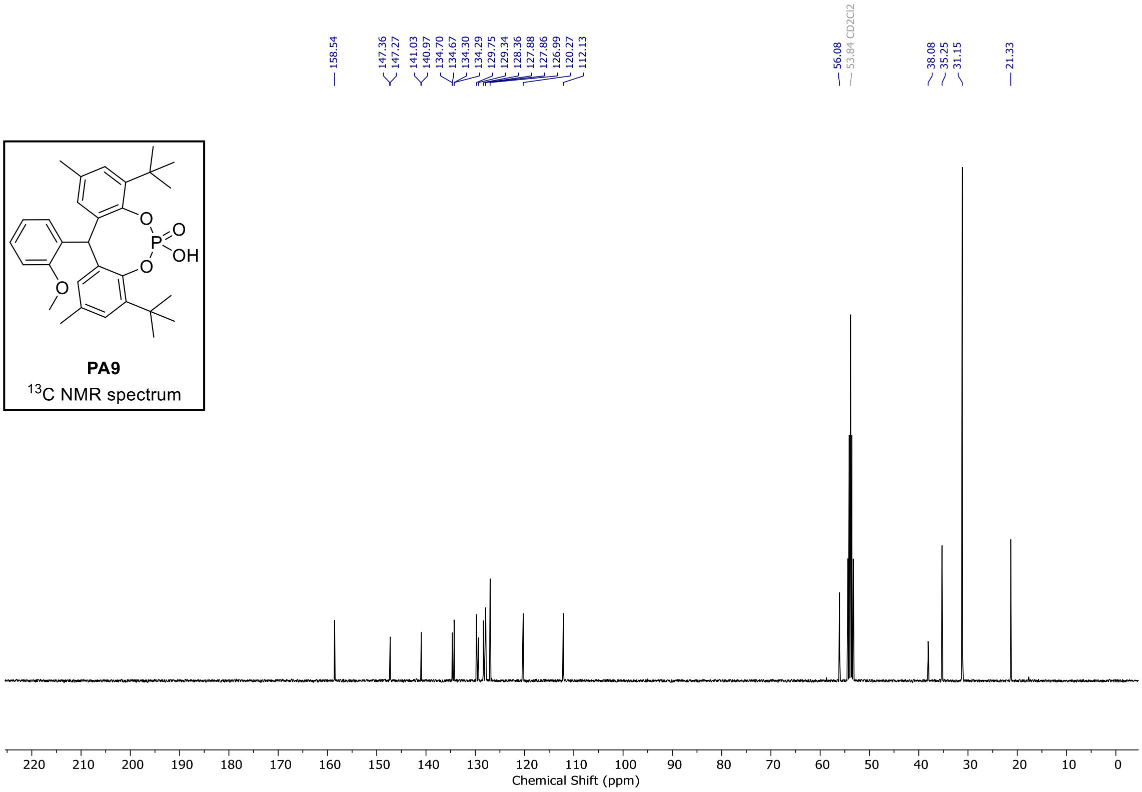
PA9
¹³C NMR spectrum
Chemical Shift (ppm)

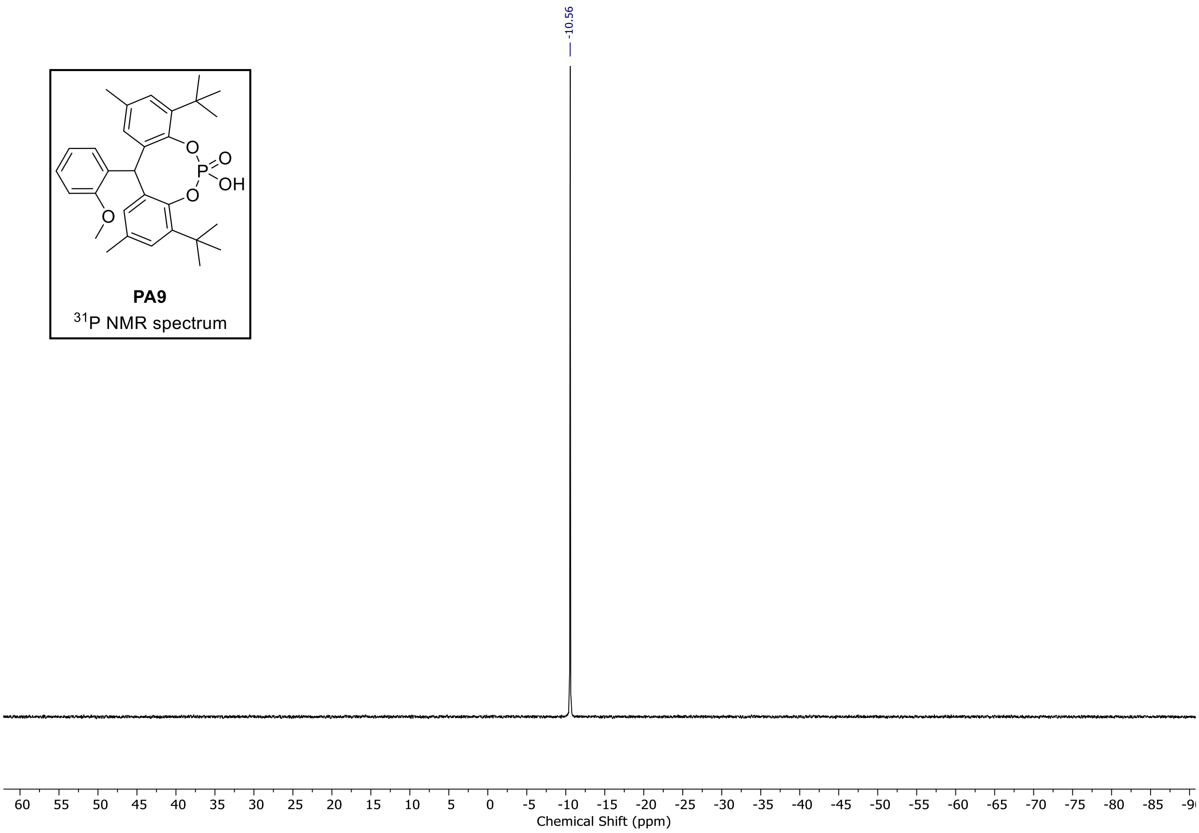

PA9

^{31}P NMR spectrum

17. NMR spectra of literature unknown enone substrates

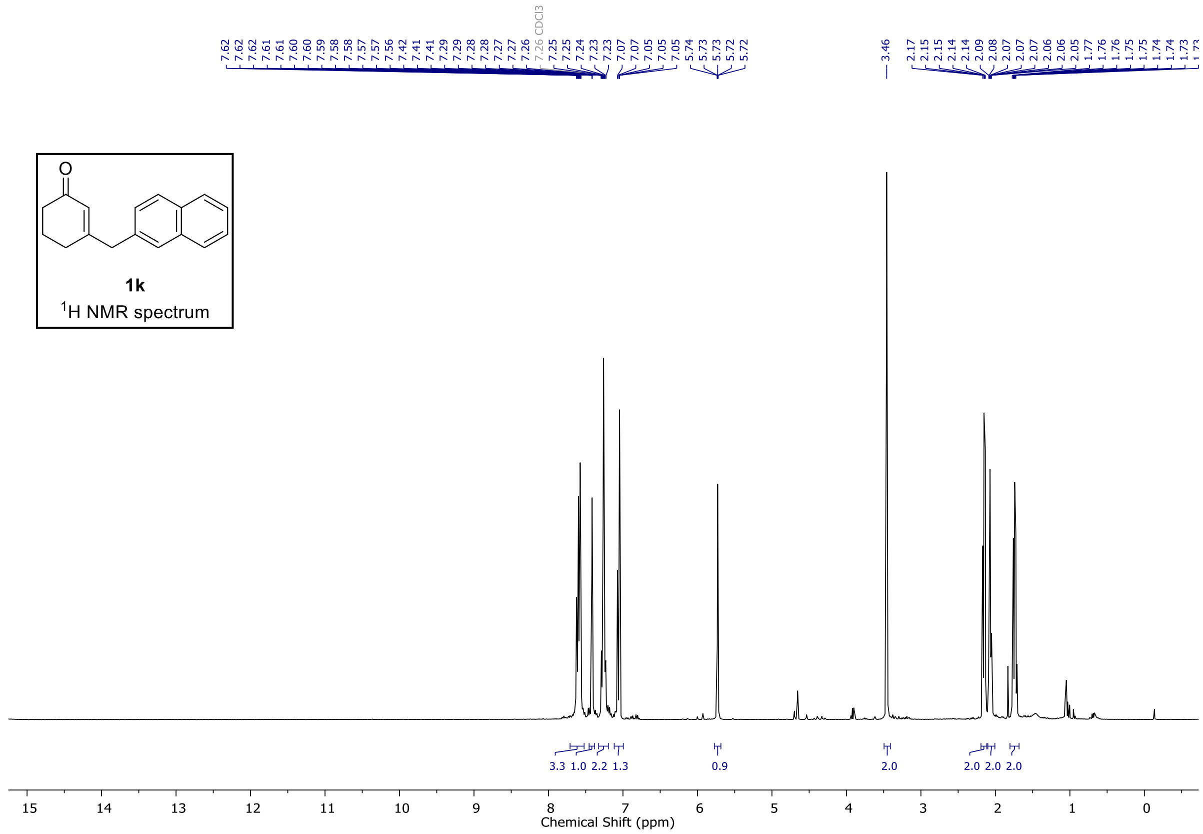

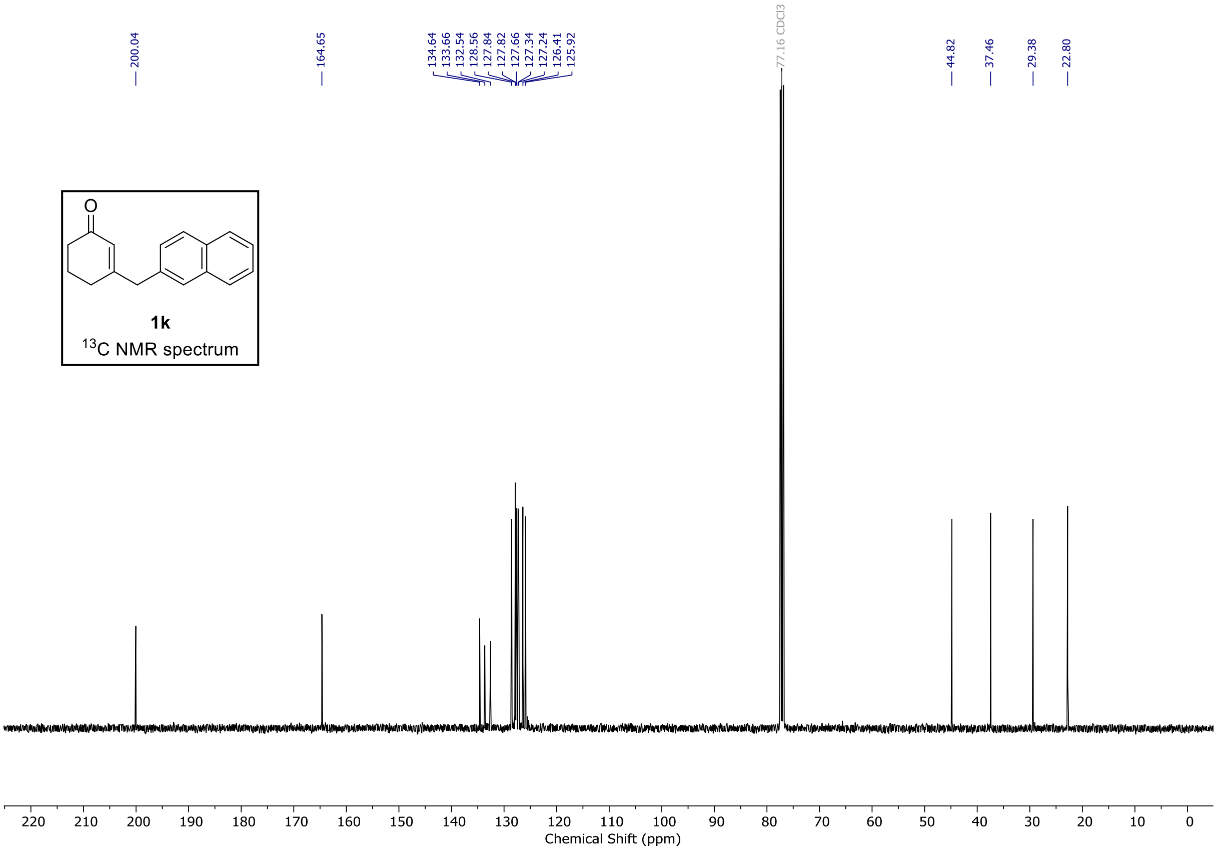

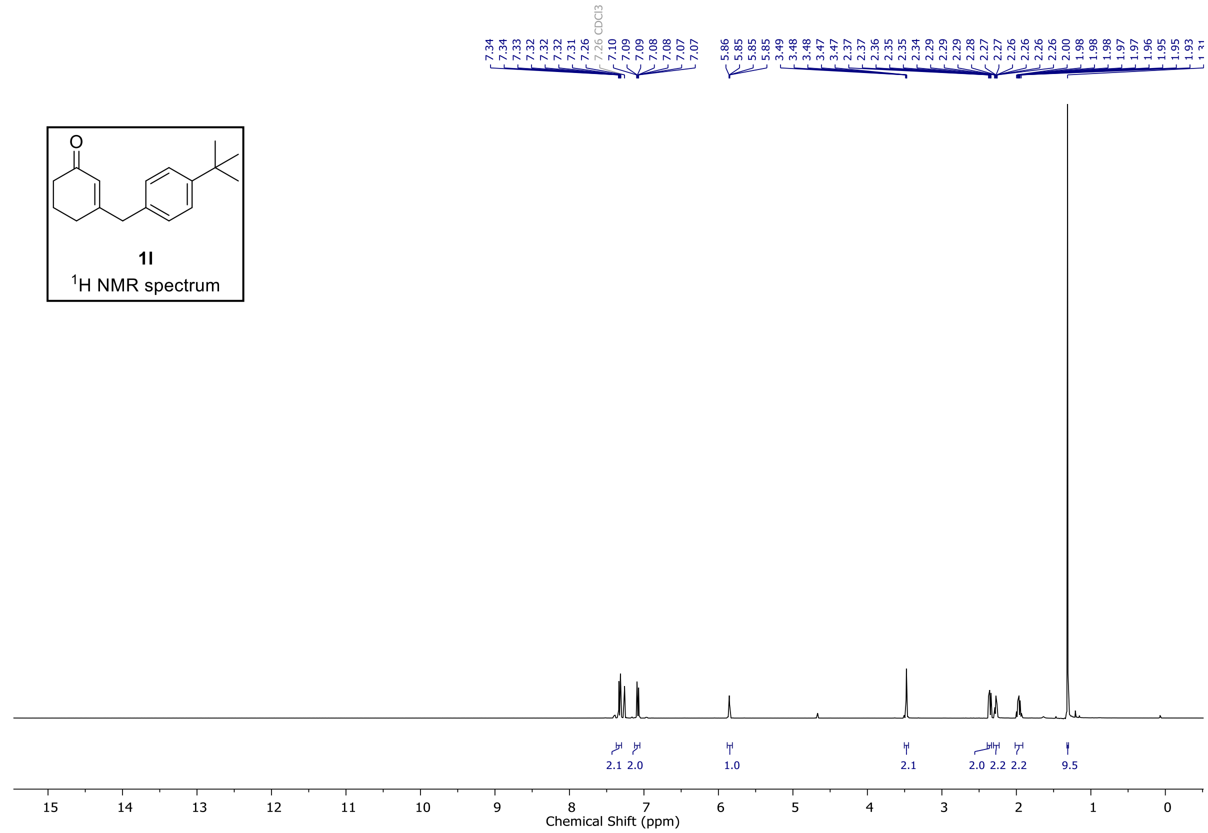

1l
^{1}H NMR spectrum
Chemical Shift (ppm)

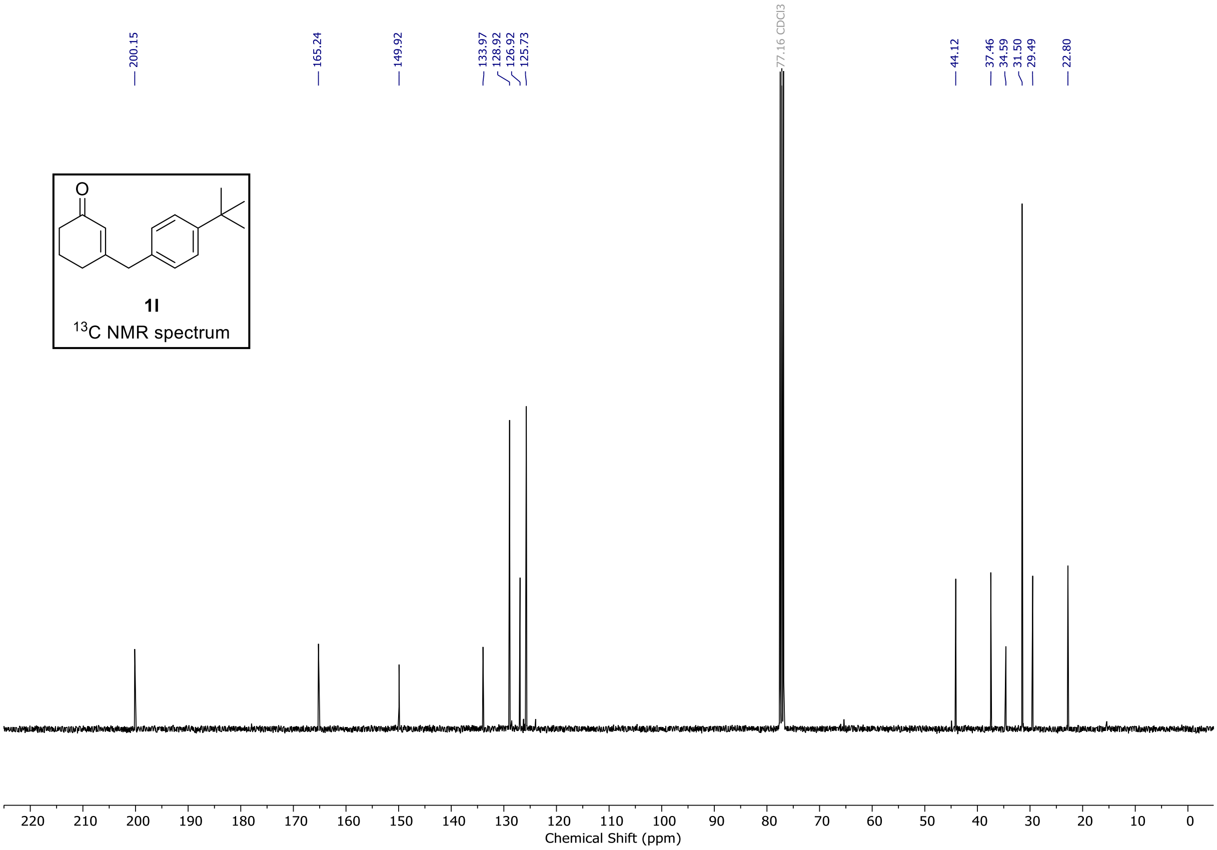

1l
^{13}C NMR spectrum
200.15
165.24
149.92
133.97
128.92
126.92
125.73
77.16 CDCl3
44.12
37.46
34.59
31.50
29.49
22.80
Chemical Shift (ppm)

18. NMR spectra for the asymmetric epoxidations

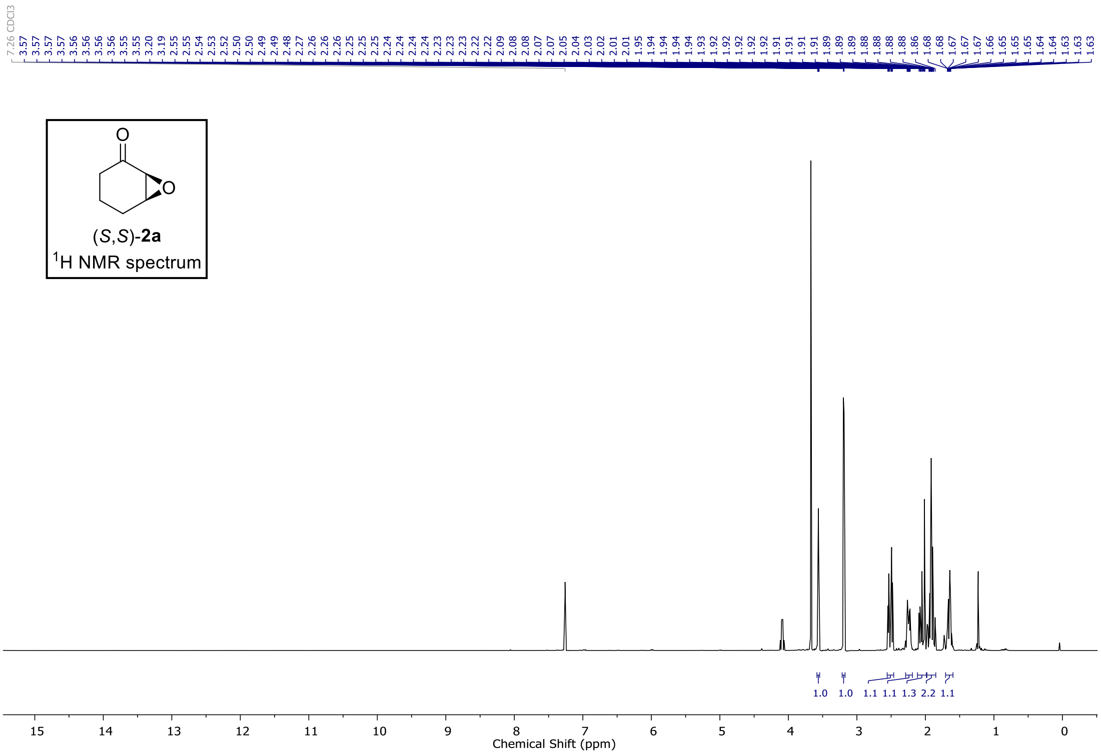

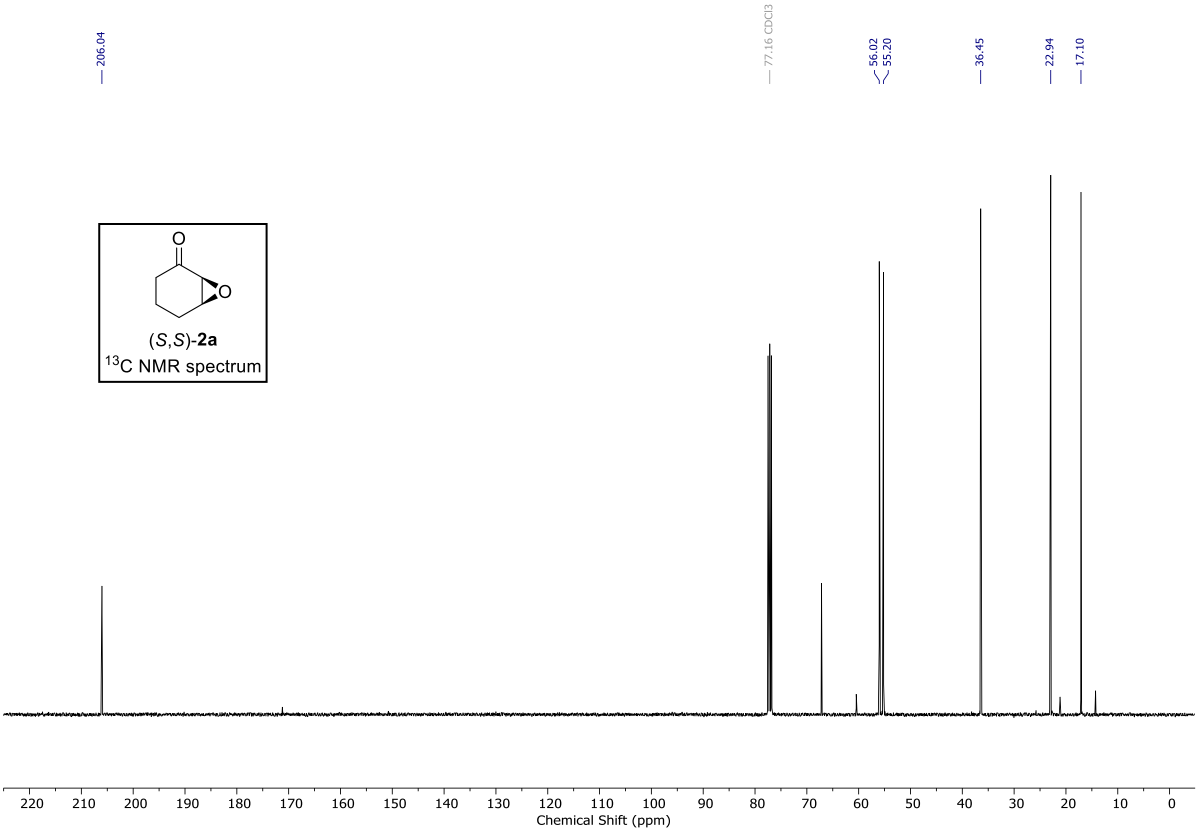

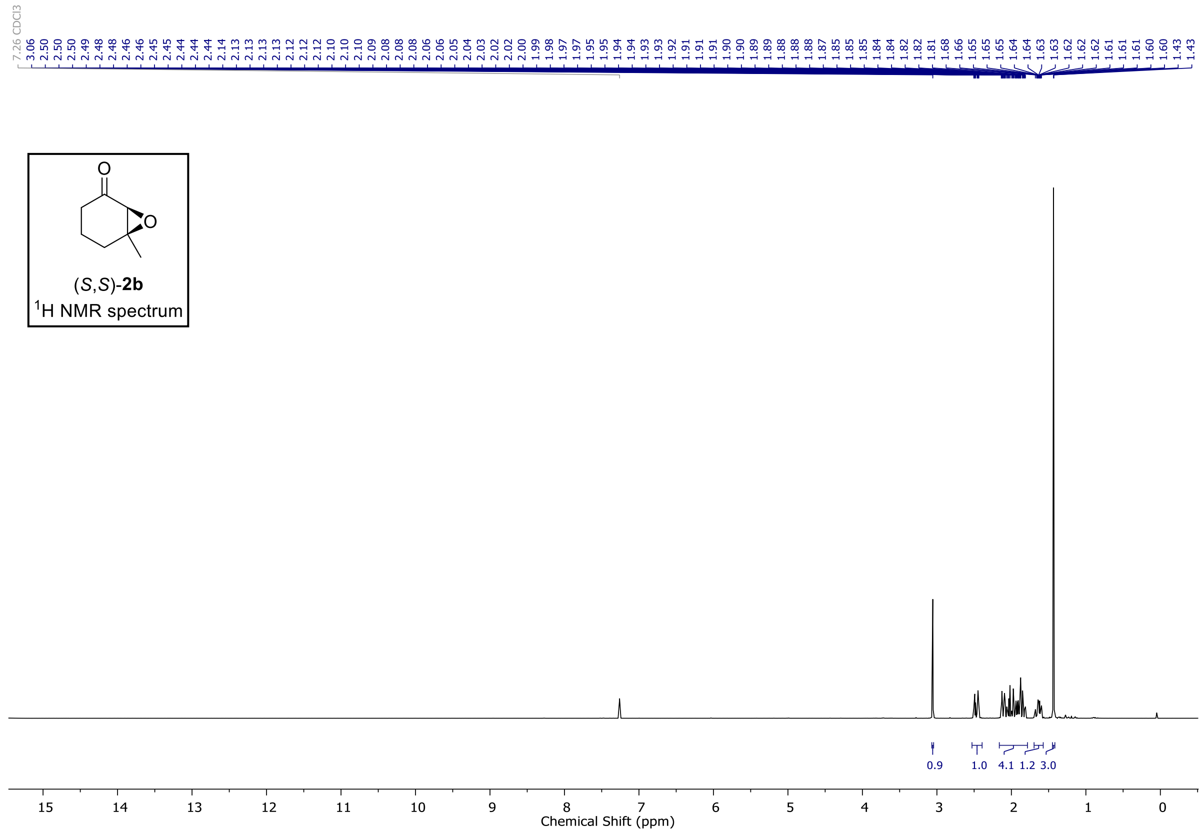
(S,S)-2b
1H NMR spectrum
Chemical Shift (ppm)
0.9
1.0
4.1
1.2
3.0
7.26 CDCl3
3.06

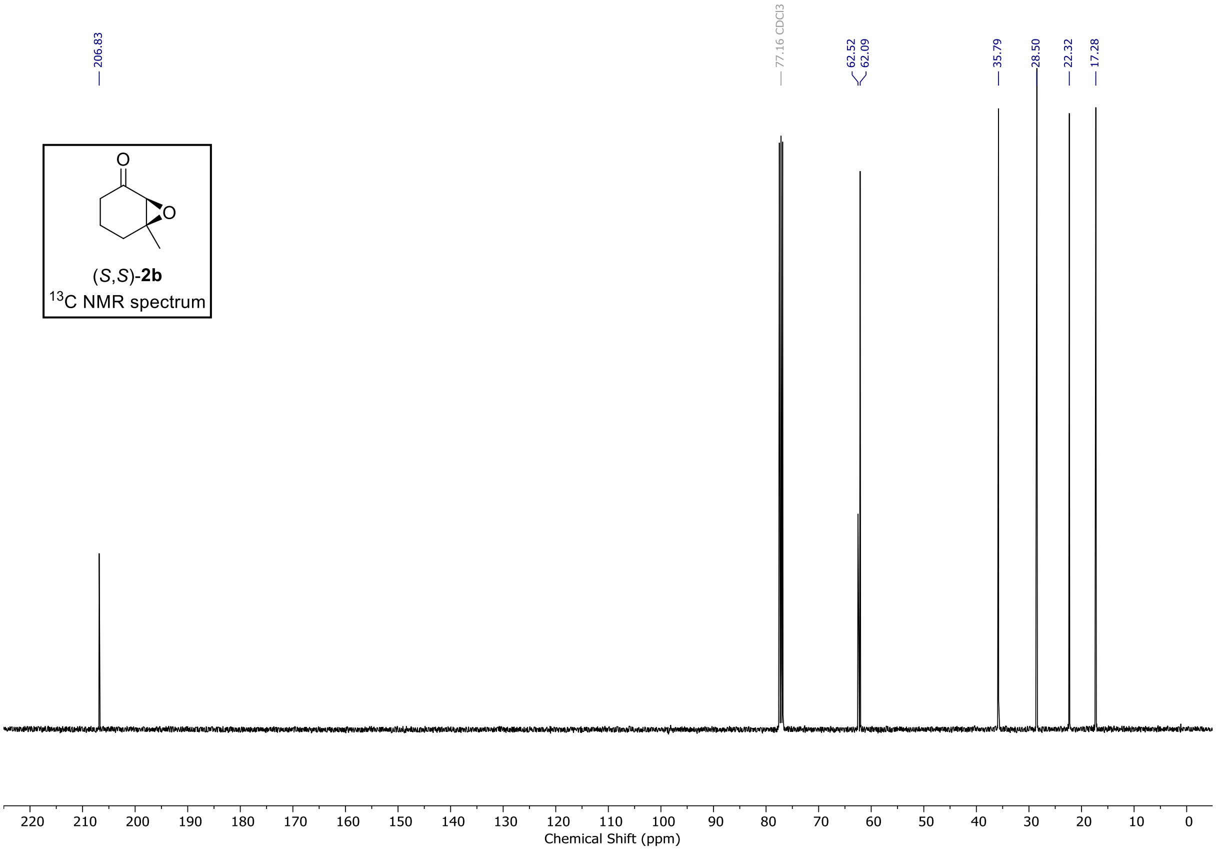
(S,S)-2b
13C NMR spectrum
Chemical Shift (ppm)
206.83
77.16 CDCl3
62.52
62.09
35.79
28.50
22.32
17.28

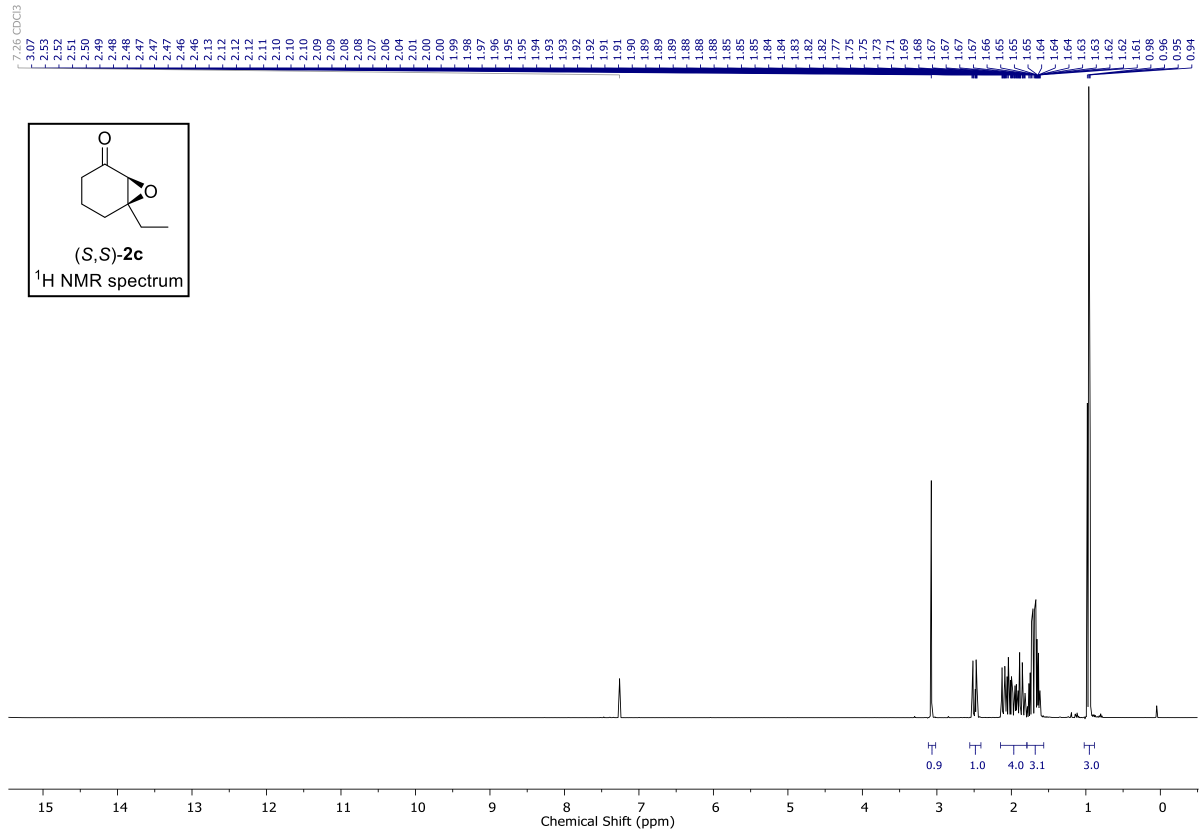
(S,S)-2c
1H NMR spectrum
Chemical Shift (ppm)

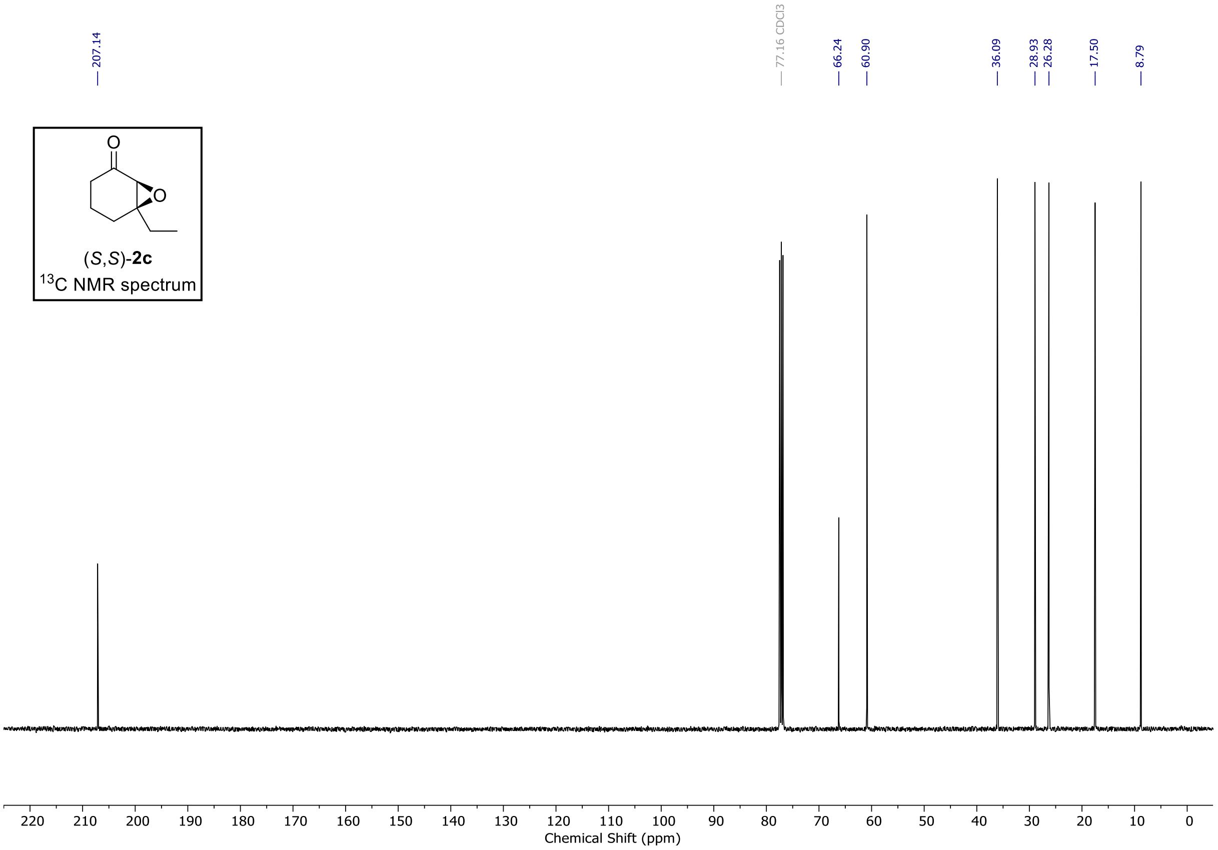
207.14
77.16 CDCl3
66.24
60.90
36.09
28.93
26.28
17.50
8.79
(S,S)-2c
13C NMR spectrum
Chemical Shift (ppm)

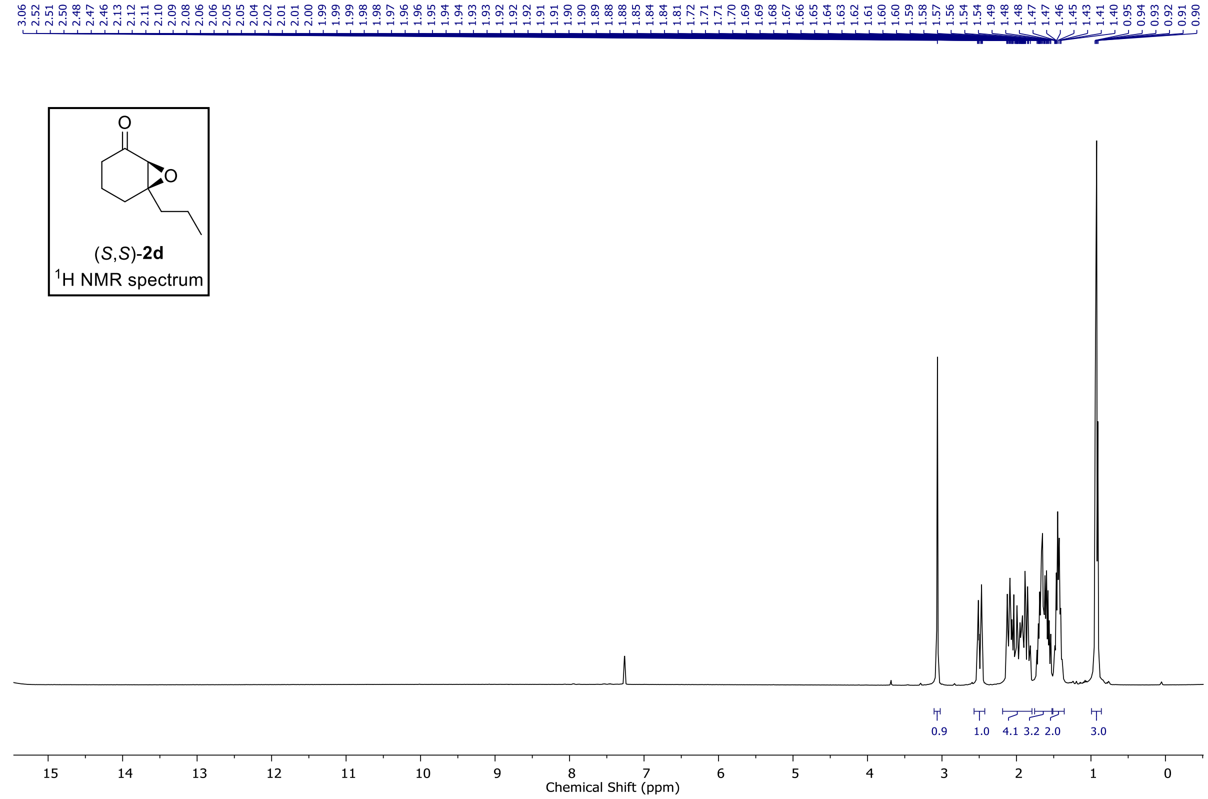
3.06
2.52
2.51
2.50
2.48
2.47
2.46
2.13
2.12
2.11
2.10
2.09
2.08
2.06
2.06
2.05
2.05
2.04
2.02
2.01
2.00
1.99
1.99
1.99
1.98
1.98
1.97
1.96
1.96
1.95
1.94
1.94
1.93
1.93
1.92
1.92
1.92
1.91
1.91
1.90
1.90
1.89
1.88
1.88
1.85
1.84
1.84
1.81
1.72
1.71
1.71
1.70
1.69
1.69
1.68
1.67
1.66
1.65
1.64
1.63
1.62
1.61
1.60
1.60
1.59
1.58
1.57
1.56
1.54
1.54
1.49
1.48
1.48
1.47
1.46
1.45
1.43
1.41
1.40
0.95
0.94
0.93
0.92
0.91
0.90
O
O
(S,S)-2d
1H NMR spectrum
0.9
1.0
4.1
3.2
2.0
3.0
15
14
13
12
11
10
9
8
7
6
5
4
3
2
1
0
Chemical Shift (ppm)

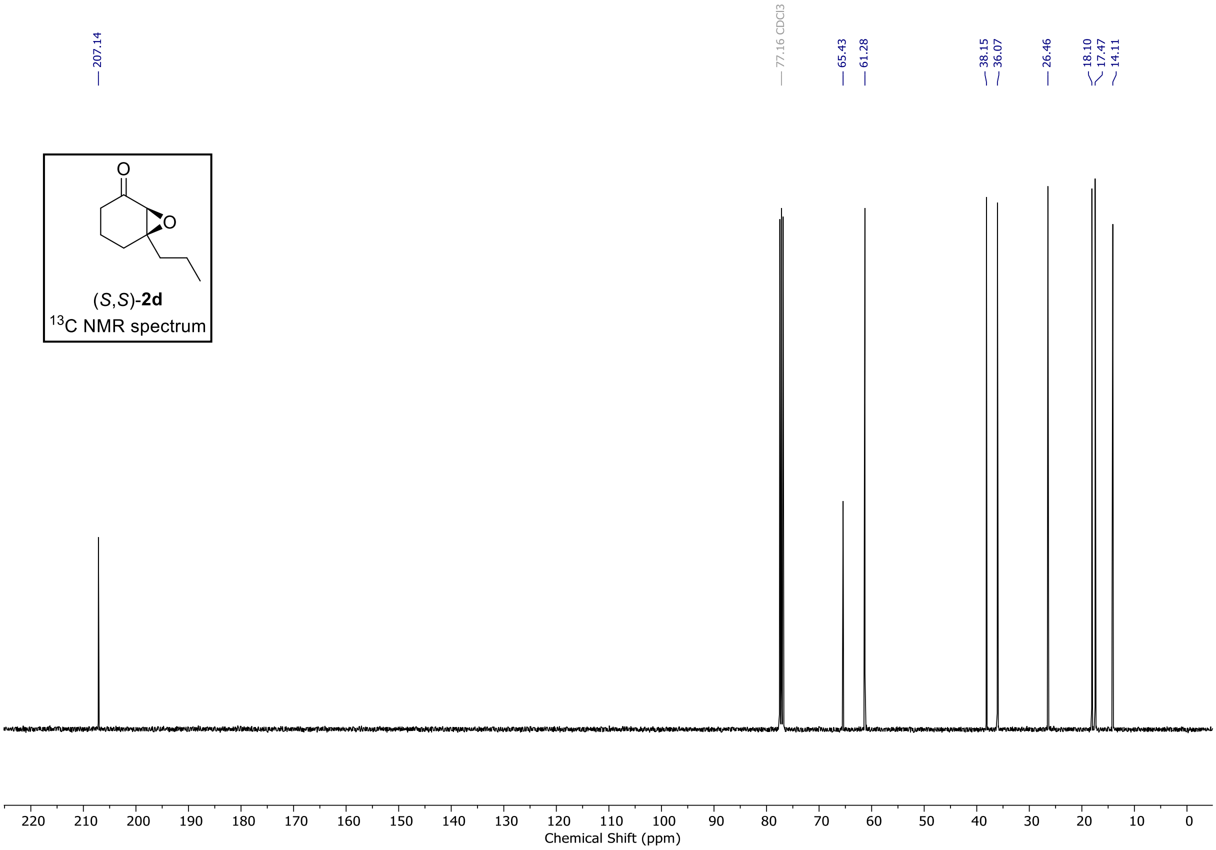
207.14
77.16 CDCl3
65.43
61.28
38.15
36.07
26.46
18.10
17.47
14.11
O
O
(S,S)-2d
13C NMR spectrum
220
210
200
190
180
170
160
150
140
130
120
110
100
90
80
70
60
50
40
30
20
10
0
Chemical Shift (ppm)

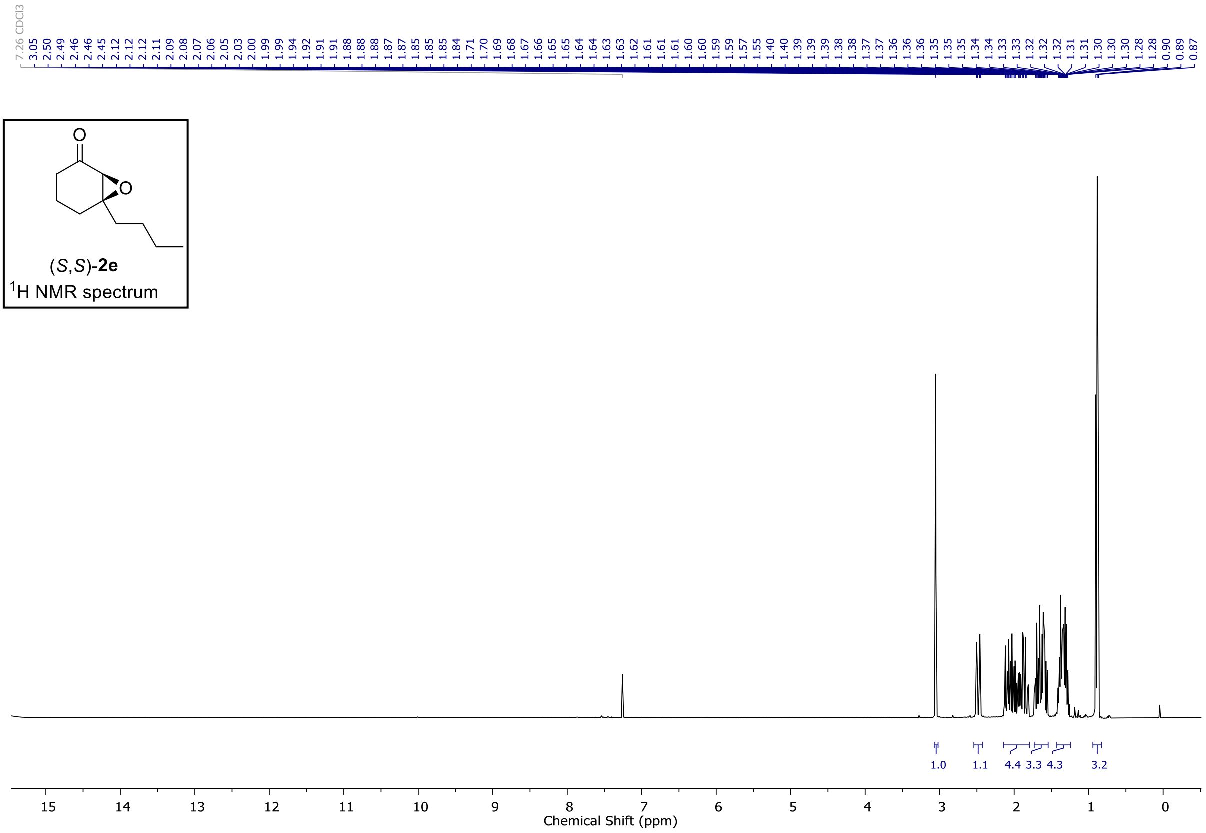
7.26 CDCl3
3.05 2.50 2.49 2.46 2.46 2.45 2.12 2.12 2.12 2.11 2.09 2.08 2.07 2.06 2.05 2.03 2.00 1.99 1.99 1.94 1.92 1.91 1.91 1.91 1.88 1.88 1.88 1.87 1.85 1.85 1.85 1.84 1.71 1.70 1.69 1.68 1.67 1.66 1.65 1.65 1.64 1.64 1.63 1.63 1.62 1.61 1.61 1.61 1.60 1.60 1.59 1.59 1.57 1.55 1.40 1.40 1.39 1.39 1.39 1.38 1.38 1.37 1.37 1.36 1.36 1.35 1.35 1.35 1.34 1.33 1.33 1.32 1.32 1.32 1.31 1.31 1.30 1.30 1.30 1.28 0.90 0.89 0.87
O
O
(S,S)-2e
1H NMR spectrum
1.0
1.1
4.4 3.3 4.3
3.2
15 14 13 12 11 10 9 8 7 6 5 4 3 2 1 0
Chemical Shift (ppm)

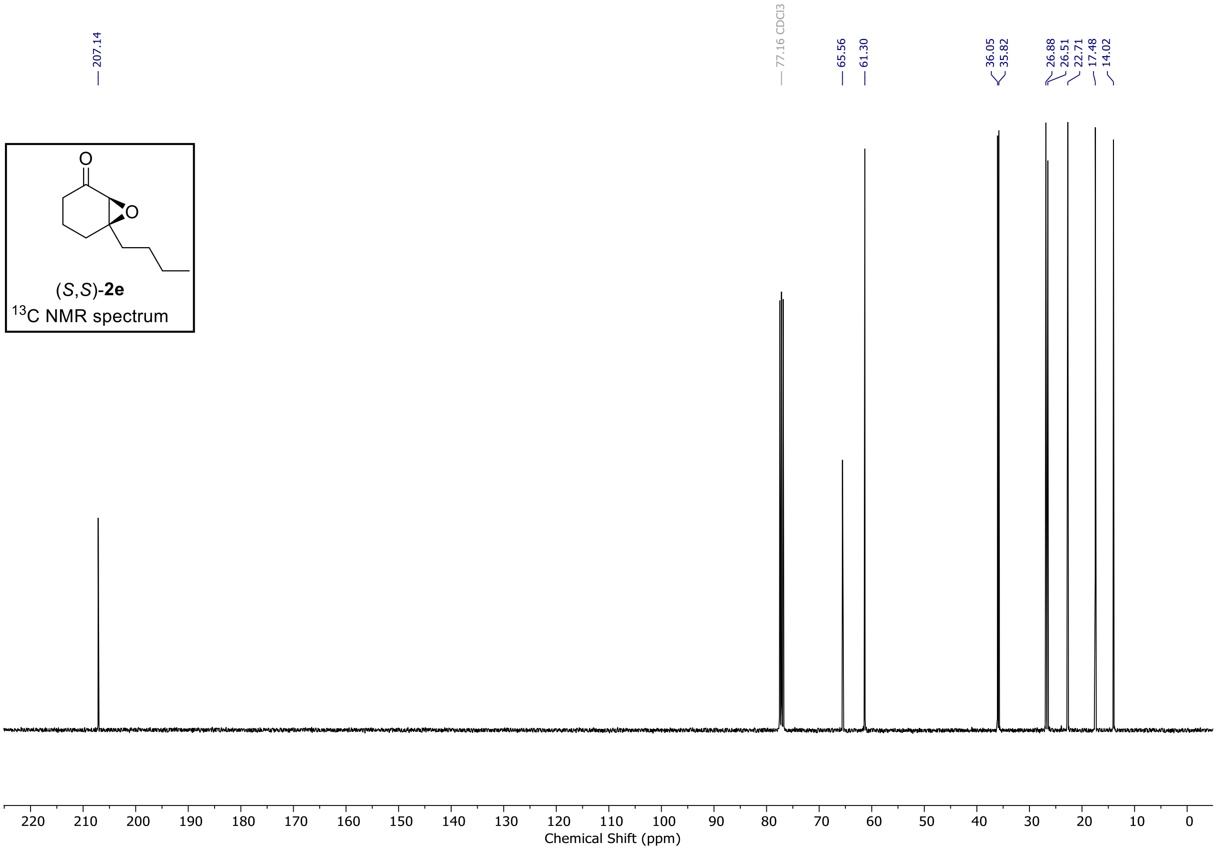
207.14
77.16 CDCl3
65.56
61.30
36.05
35.82
26.88
26.51
22.71
17.48
14.02
O
O
(S,S)-2e
13C NMR spectrum
220 210 200 190 180 170 160 150 140 130 120 110 100 90 80 70 60 50 40 30 20 10 0
Chemical Shift (ppm)

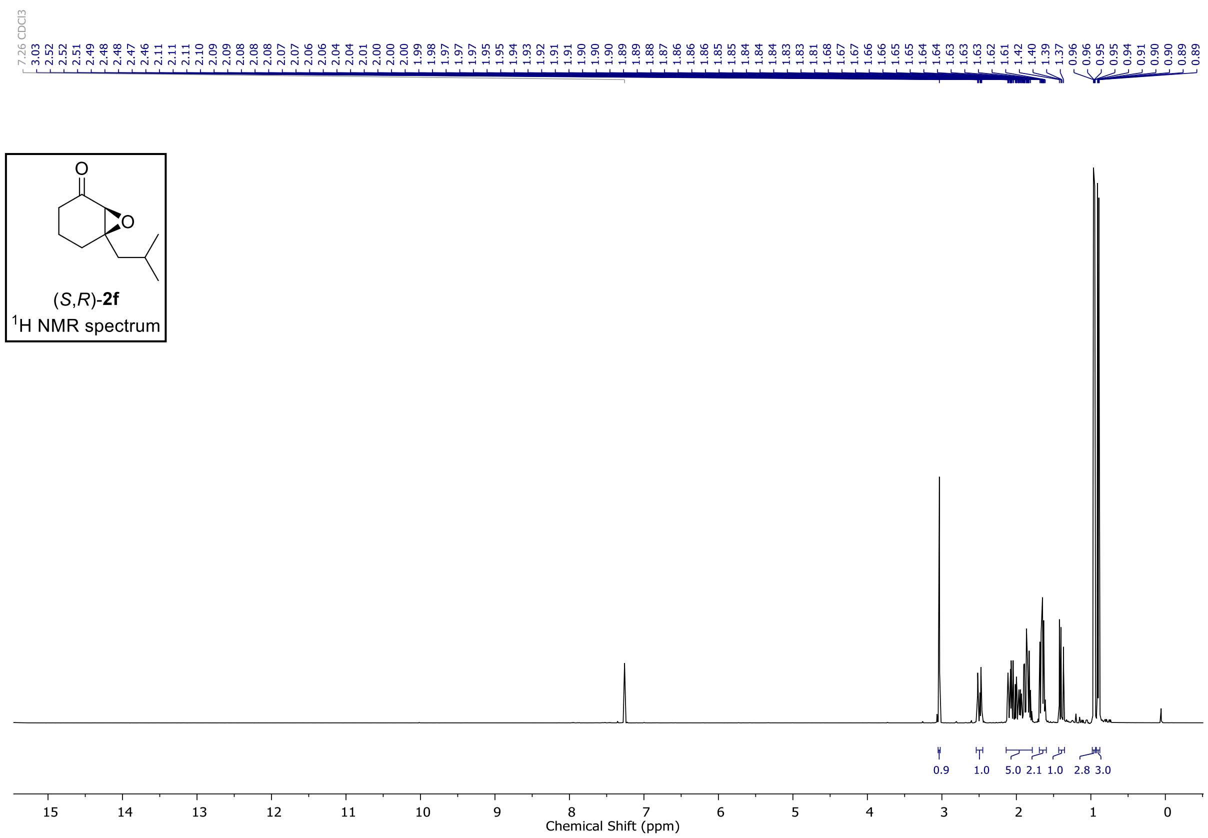
7.26 CDCl3
3.03
2.52
2.52
2.51
2.49
2.48
2.48
2.47
2.46
2.11
2.11
2.11
2.10
2.09
2.09
2.08
2.08
2.08
2.07
2.07
2.06
2.06
2.04
2.04
2.01
2.00
2.00
1.99
1.98
1.97
1.97
1.97
1.95
1.95
1.94
1.93
1.92
1.91
1.91
1.90
1.90
1.90
1.89
1.89
1.88
1.87
1.86
1.86
1.85
1.85
1.84
1.84
1.84
1.83
1.83
1.81
1.68
1.67
1.67
1.66
1.66
1.65
1.65
1.64
1.64
1.63
1.63
1.63
1.62
1.61
1.42
1.40
1.39
1.37
0.96
0.96
0.95
0.95
0.94
0.91
0.90
0.90
0.89
0.89
O
O
(S,R)-2f
^{1}H NMR spectrum
0.9
1.0
5.0
2.1
1.0
2.8
3.0
15
14
13
12
11
10
9
8
7
6
5
4
3
2
1
0
Chemical Shift (ppm)

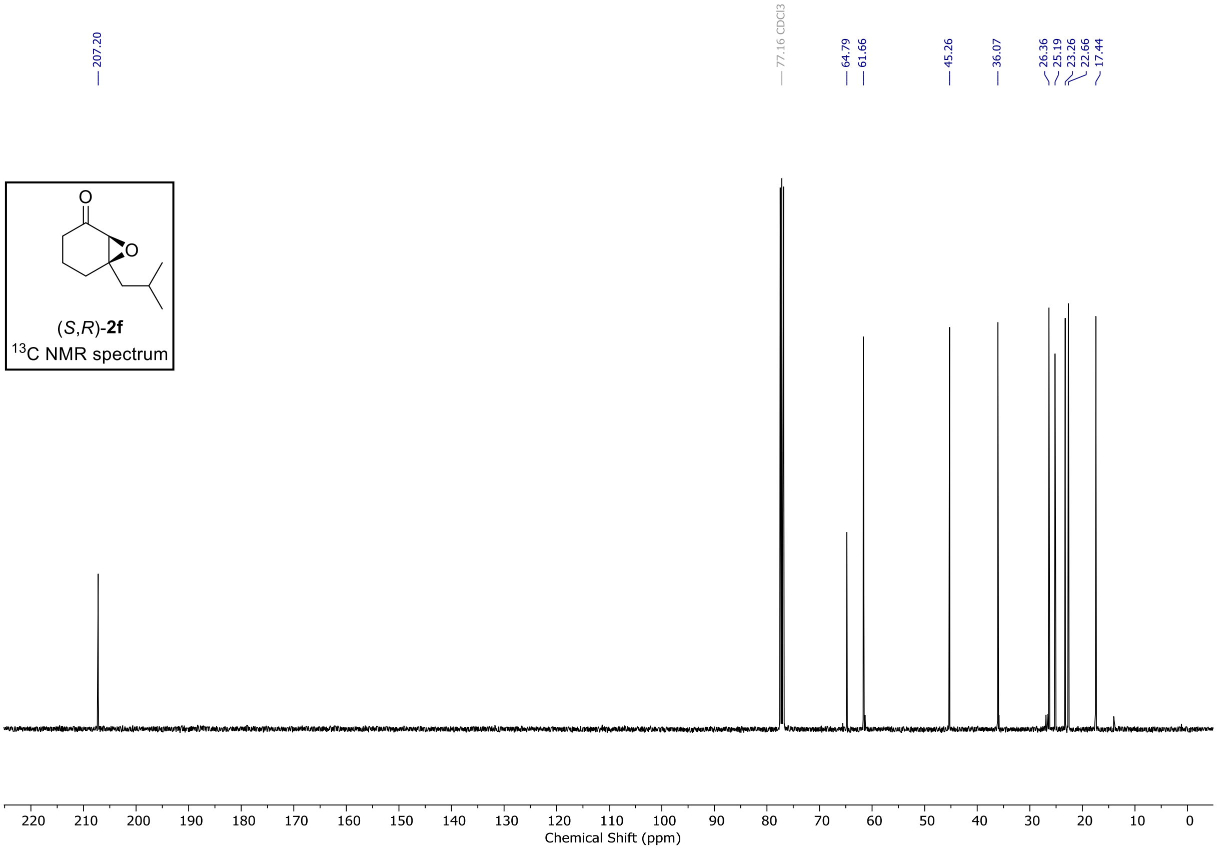
207.20
77.16 CDCl3
64.79
61.66
45.26
36.07
26.36
25.19
23.26
22.66
17.44
O
O
(S,R)-2f
^{13}C NMR spectrum
220
210
200
190
180
170
160
150
140
130
120
110
100
90
80
70
60
50
40
30
20
10
0
Chemical Shift (ppm)

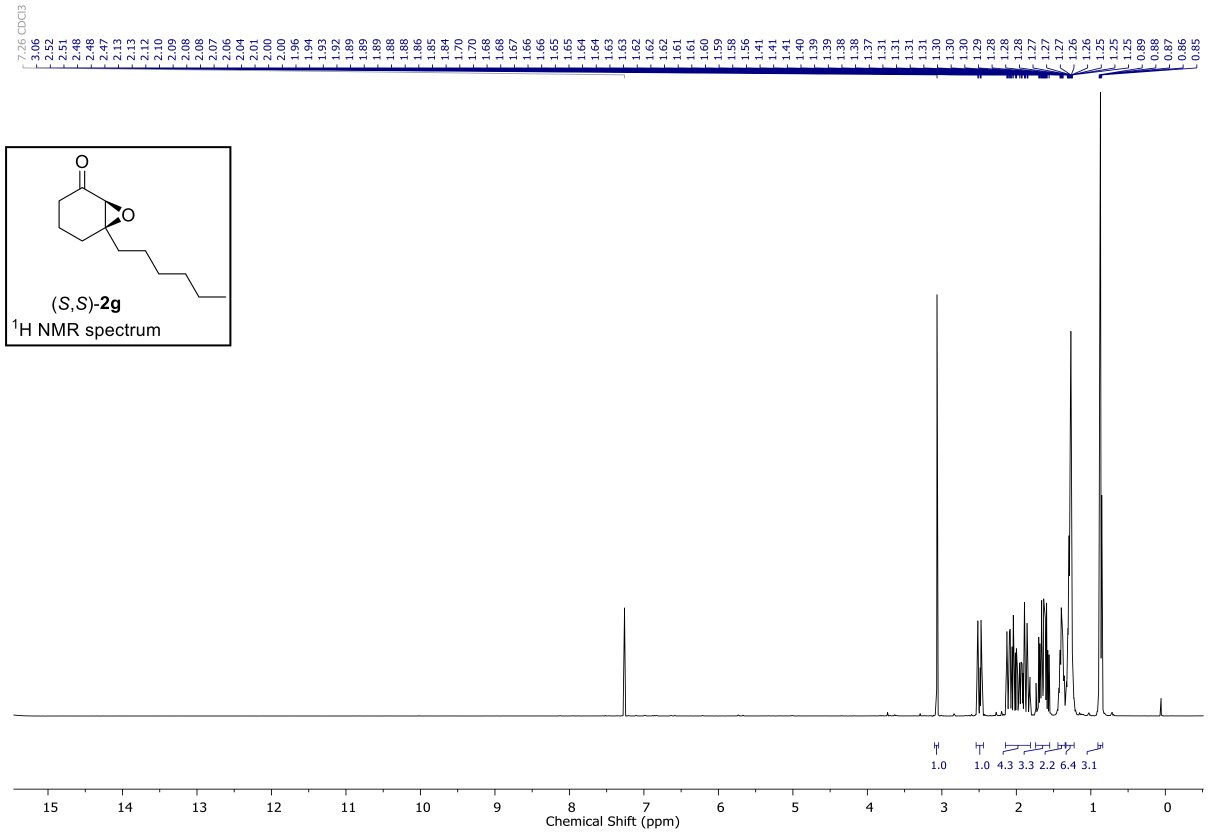
(S,S)-2g
1H NMR spectrum
Chemical Shift (ppm)

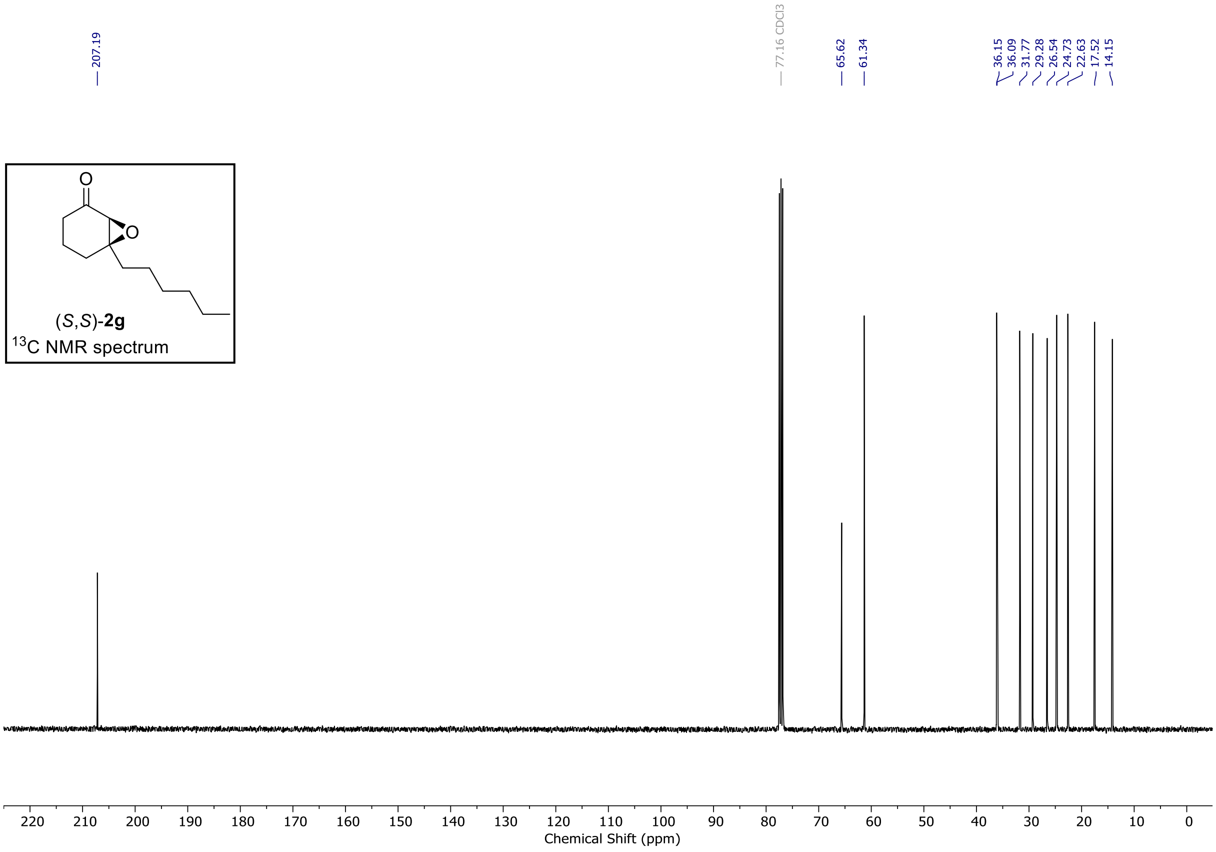
(S,S)-2g
13C NMR spectrum
207.19
77.16 CDCl3
65.62
61.34
36.15
36.09
31.77
29.28
26.54
24.73
22.63
17.52
14.15
Chemical Shift (ppm)

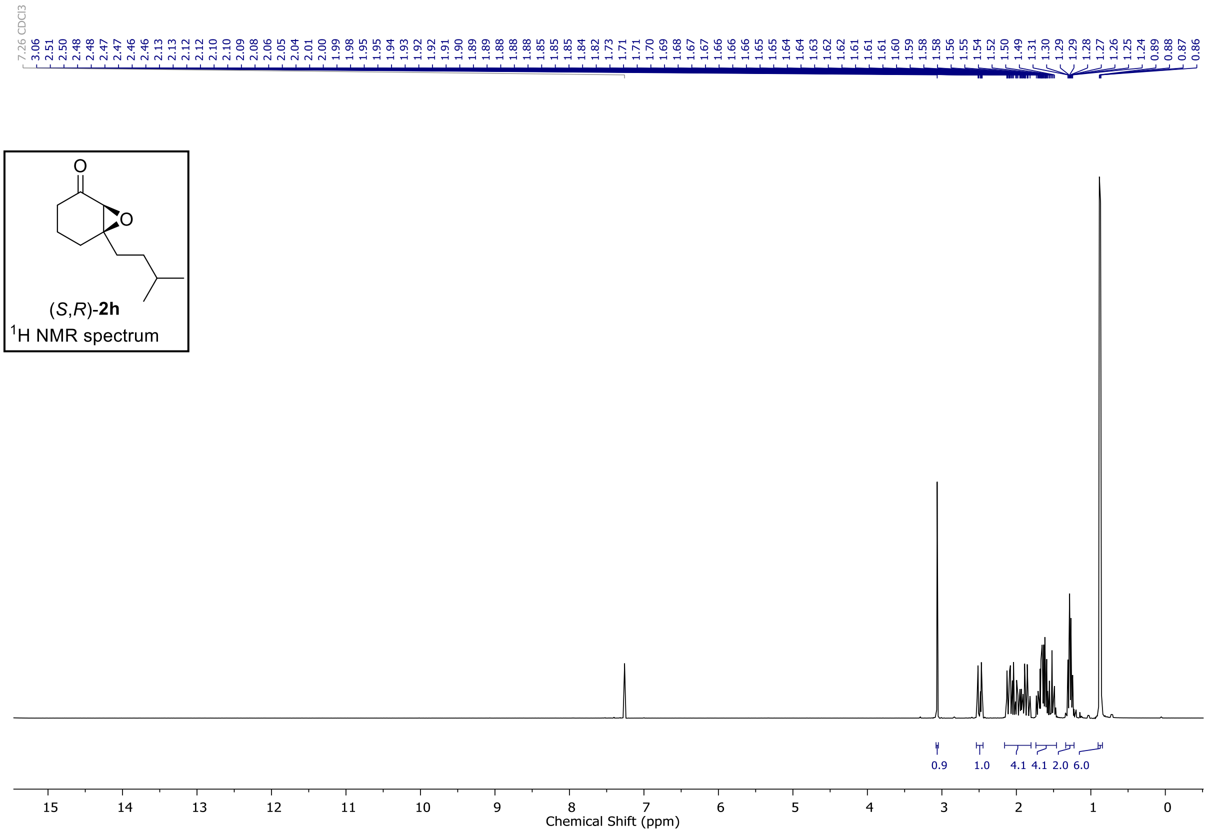
(S,R)-2h
1H NMR spectrum
Chemical Shift (ppm)

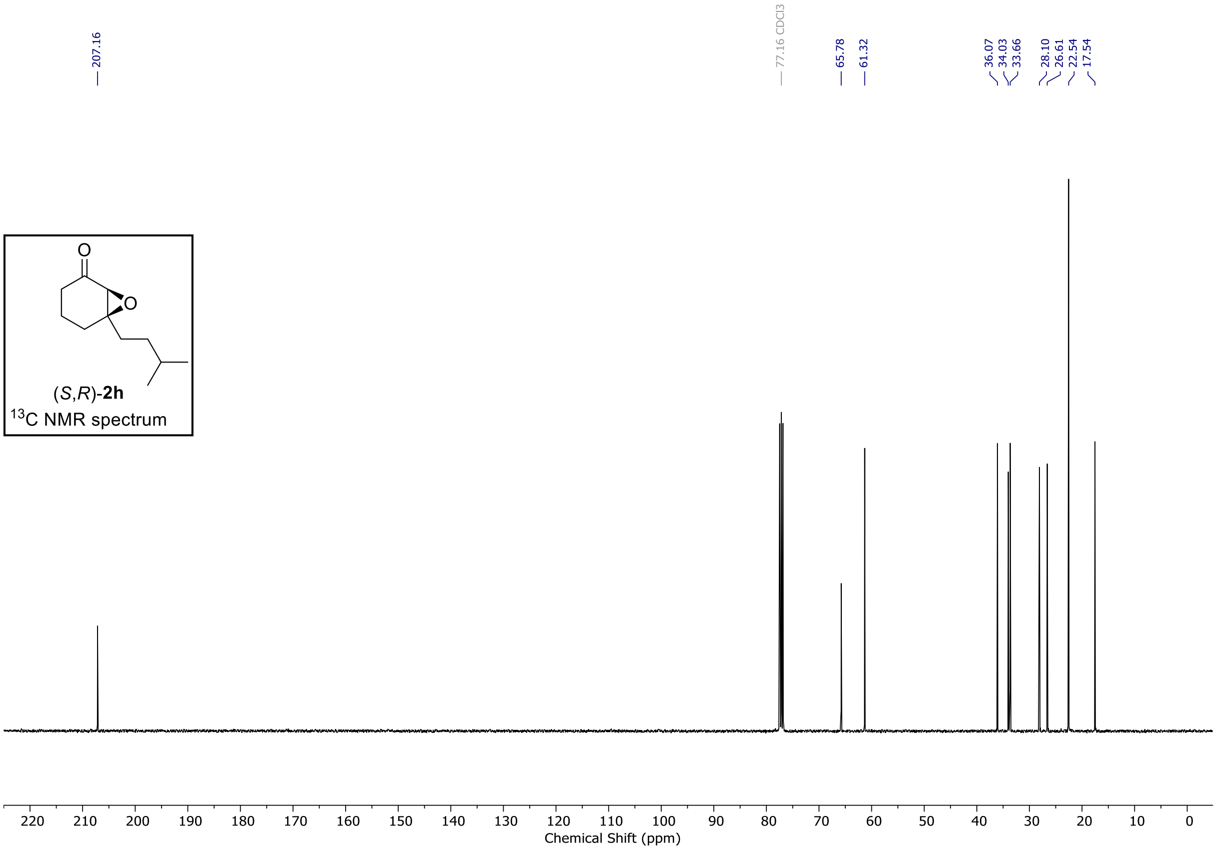
(S,R)-2h
13C NMR spectrum
207.16
77.16 CDCl3
65.78
61.32
36.07
34.03
33.66
28.10
26.61
22.54
17.54
Chemical Shift (ppm)

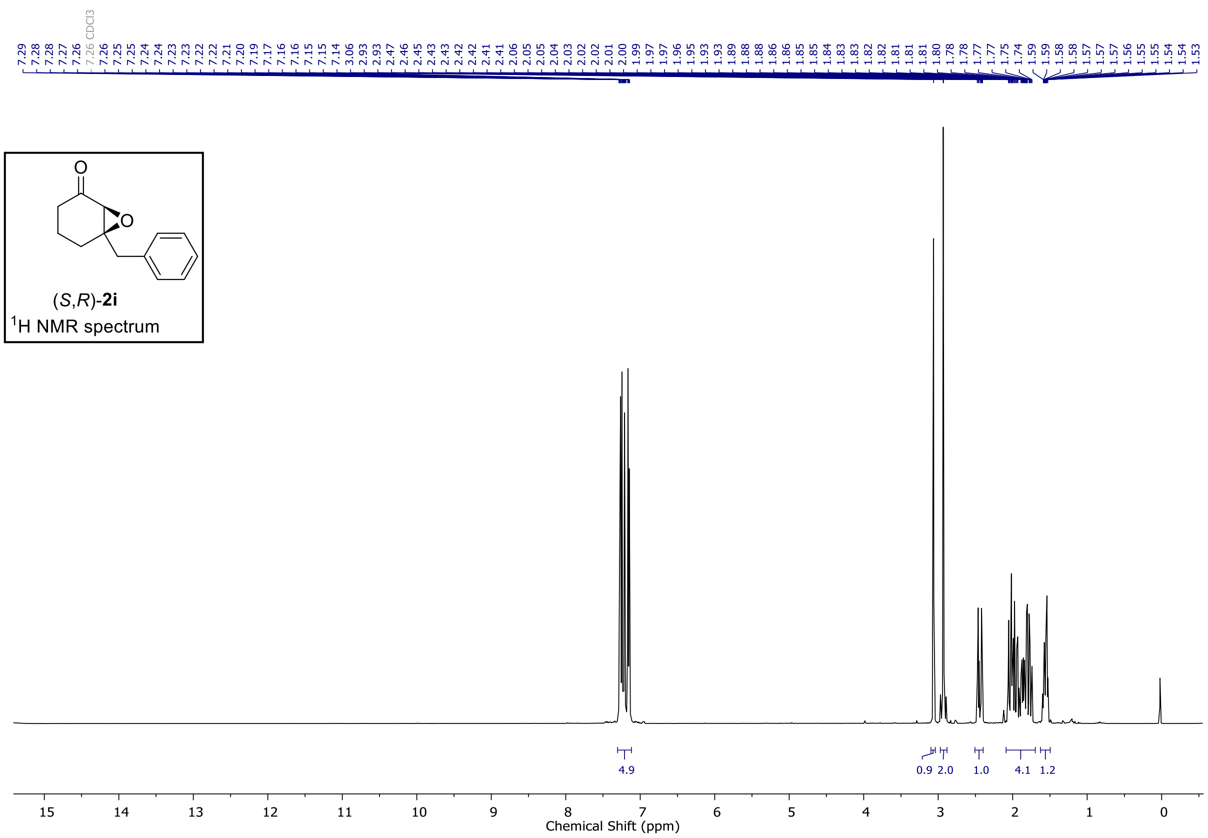

7.29 7.28 7.28 7.27 7.26 7.26 CDCl3 7.26 7.25 7.25 7.24 7.24 7.23 7.23 7.22 7.22 7.21 7.20 7.19 7.17 7.16 7.16 7.15 7.15 7.14 3.06 2.93 2.93 2.93 2.47 2.46 2.45 2.43 2.43 2.42 2.42 2.41 2.41 2.06 2.05 2.05 2.04 2.03 2.02 2.02 2.01 2.00 1.99 1.97 1.97 1.96 1.95 1.93 1.93 1.89 1.88 1.88 1.86 1.86 1.85 1.85 1.84 1.83 1.83 1.82 1.82 1.81 1.81 1.81 1.80 1.78 1.78 1.77 1.77 1.75 1.74 1.59 1.59 1.58 1.58 1.57 1.57 1.57 1.56 1.55 1.55 1.54 1.54 1.53
O
O
(S,R)-2i
1H NMR spectrum
4.9
0.9 2.0
1.0
4.1 1.2
15 14 13 12 11 10 9 8 7 6 5 4 3 2 1 0
Chemical Shift (ppm)

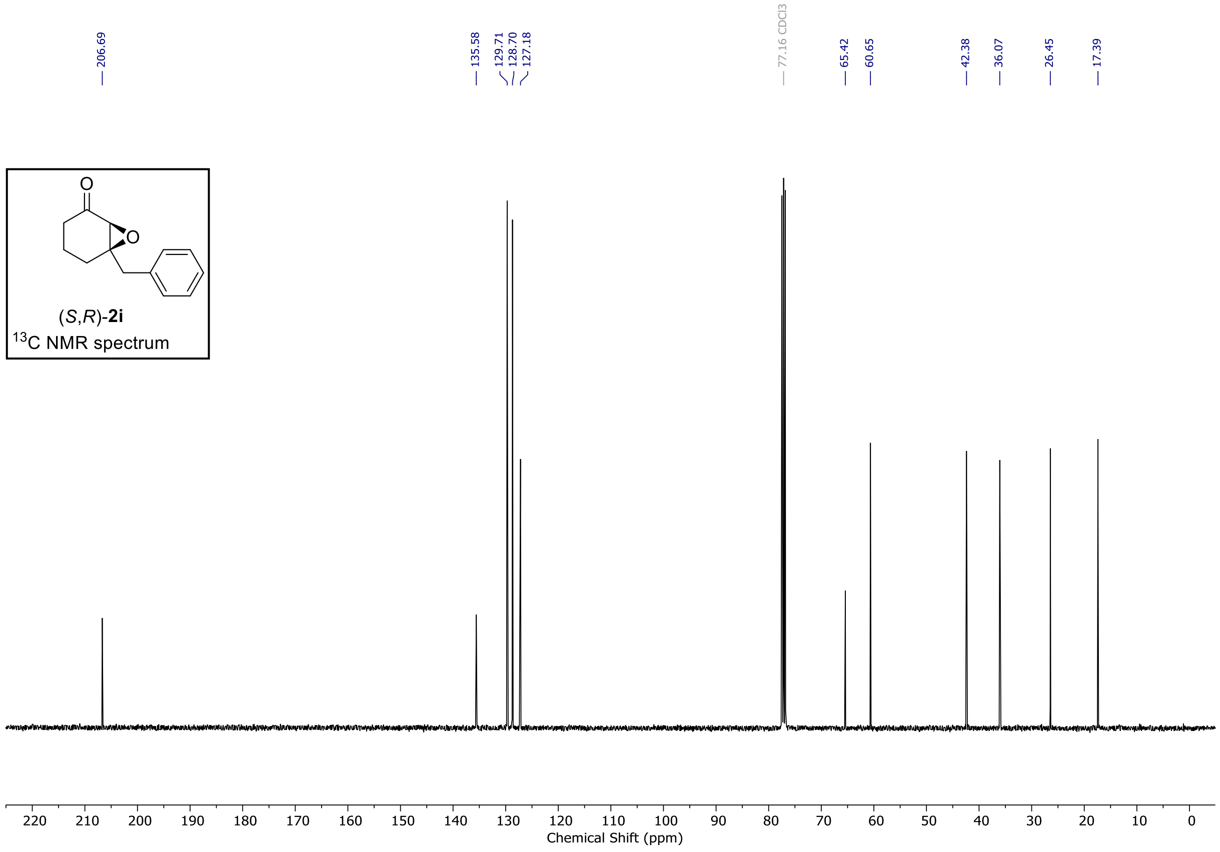

206.69
135.58 129.71 128.70 127.18
77.16 CDCl3
65.42 60.65
42.38 36.07 26.45 17.39
O
O
(S,R)-2i
13C NMR spectrum
220 210 200 190 180 170 160 150 140 130 120 110 100 90 80 70 60 50 40 30 20 10 0
Chemical Shift (ppm)

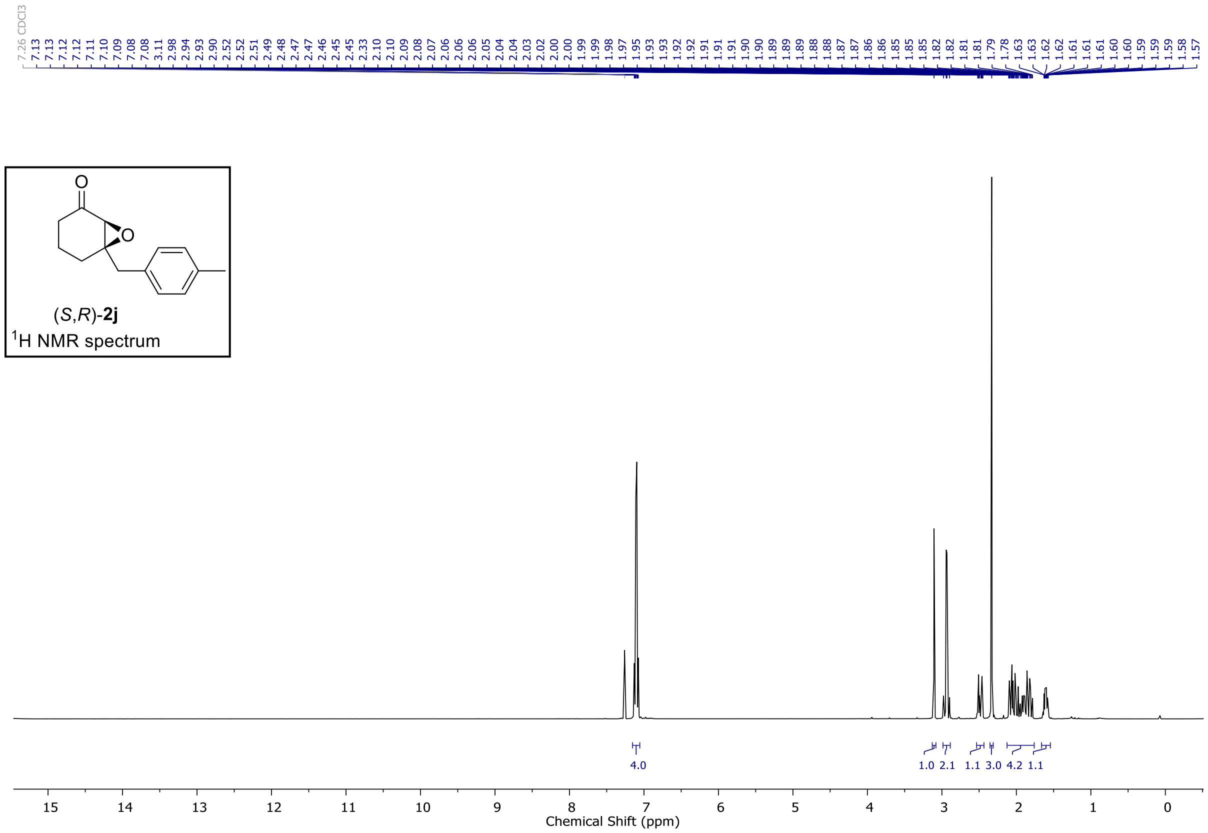
7.26 CDCl3
7.13 7.13 7.12 7.12 7.11 7.10 7.09 7.08 7.08 3.11 2.98 2.94 2.93 2.90 2.52 2.52 2.51 2.49 2.48 2.47 2.47 2.46 2.45 2.45 2.33 2.10 2.10 2.09 2.08 2.07 2.06 2.06 2.06 2.05 2.04 2.04 2.03 2.02 2.00 2.00 1.99 1.99 1.98 1.97 1.95 1.93 1.93 1.93 1.92 1.92 1.91 1.91 1.90 1.90 1.89 1.89 1.89 1.88 1.88 1.87 1.87 1.86 1.86 1.85 1.85 1.85 1.82 1.82 1.81 1.81 1.79 1.78 1.63 1.63 1.62 1.61 1.61 1.60 1.59 1.59 1.59 1.58 1.57
O
O
(S,R)-2j
1H NMR spectrum
4.0
1.0 2.1 1.1 3.0 4.2 1.1
15 14 13 12 11 10 9 8 7 6 5 4 3 2 1 0
Chemical Shift (ppm)

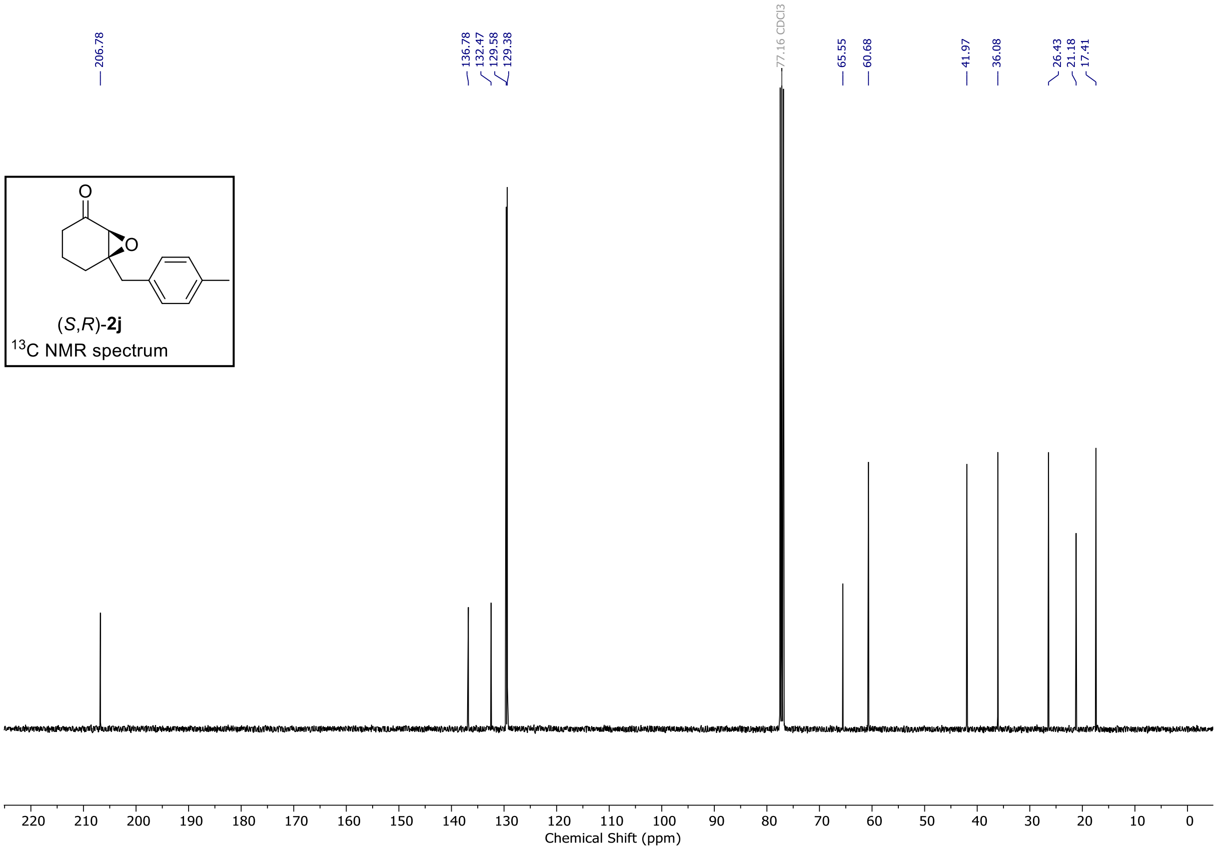
206.78
136.78 132.47 129.58 129.38
77.16 CDCl3
65.55 60.68
41.97 36.08
26.43 21.18 17.41
O
O
(S,R)-2j
13C NMR spectrum
220 210 200 190 180 170 160 150 140 130 120 110 100 90 80 70 60 50 40 30 20 10 0
Chemical Shift (ppm)

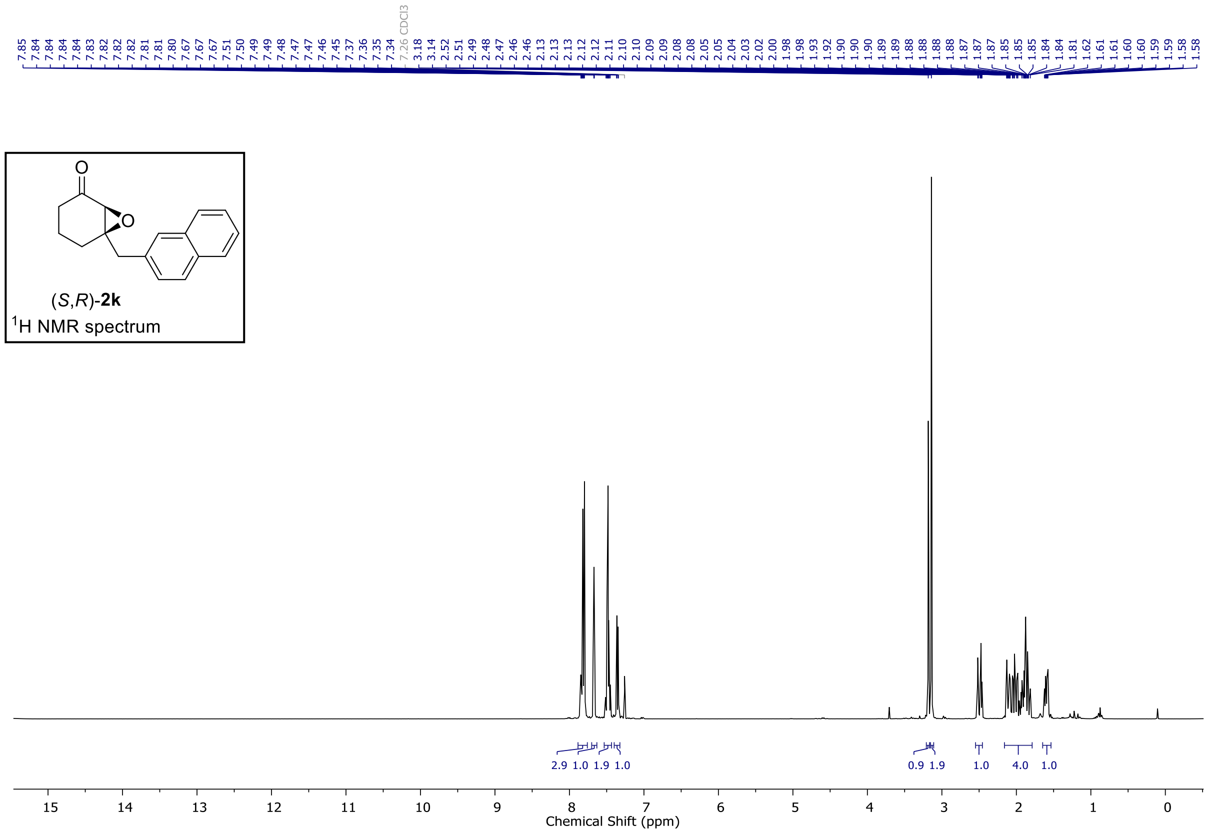
7.85 7.84 7.84 7.84 7.83 7.82 7.82 7.82 7.81 7.81 7.80 7.67 7.67 7.67 7.67 7.51 7.50 7.49 7.49 7.48 7.47 7.47 7.46 7.45 7.37 7.36 7.35 7.34 7.26 CDCl3 3.18 3.14 2.52 2.51 2.49 2.48 2.47 2.46 2.46 2.13 2.13 2.13 2.12 2.12 2.11 2.10 2.10 2.09 2.09 2.08 2.08 2.05 2.05 2.04 2.03 2.02 2.00 1.98 1.98 1.93 1.92 1.90 1.90 1.90 1.89 1.89 1.88 1.88 1.88 1.88 1.87 1.87 1.85 1.85 1.84 1.81 1.62 1.61 1.61 1.60 1.60 1.59 1.58
O
O
(S,R)-2k
1H NMR spectrum
2.9 1.0 1.9 1.0
0.9 1.9
1.0
4.0
1.0
15 14 13 12 11 10 9 8 7 6 5 4 3 2 1 0
Chemical Shift (ppm)

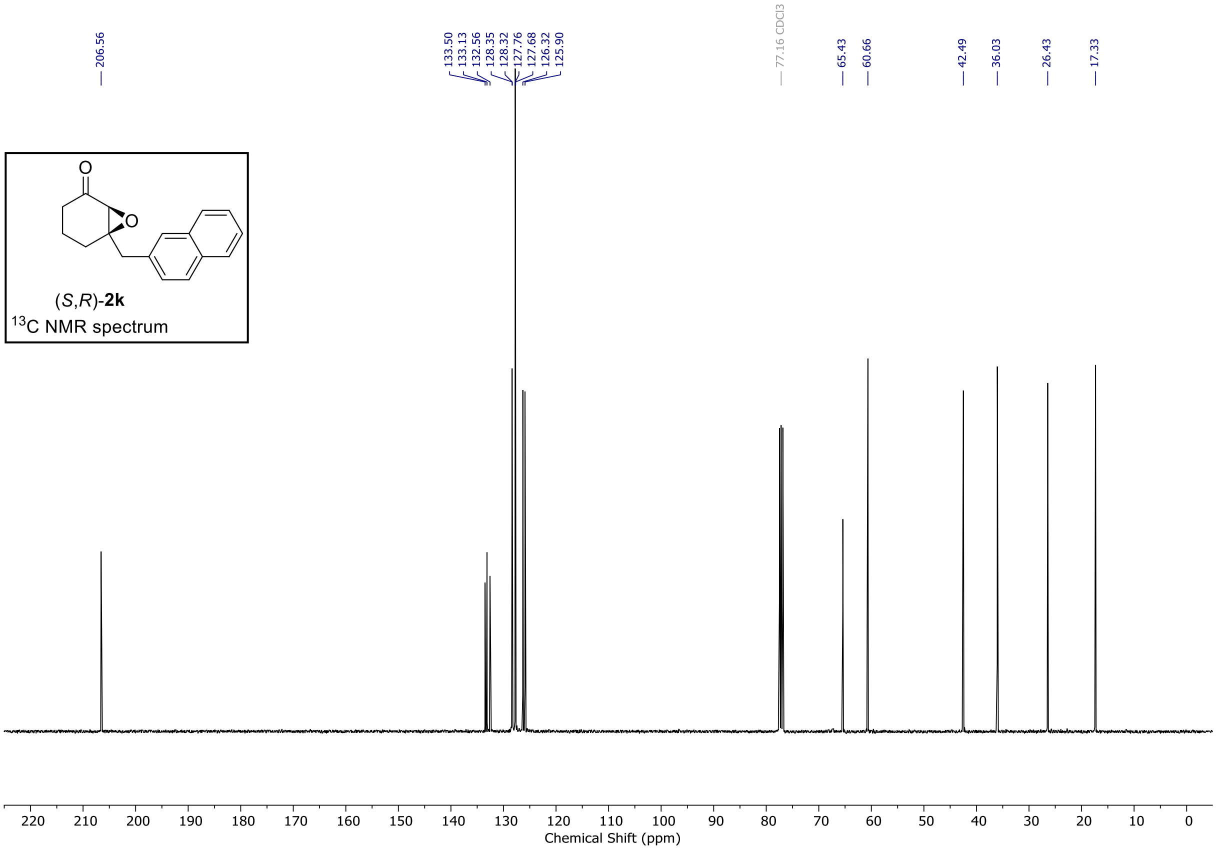
206.56
133.50 133.13 132.56 128.35 128.32 127.76 127.68 126.32 125.90
77.16 CDCl3
65.43 60.66
42.49 36.03 26.43 17.33
O
O
(S,R)-2k
13C NMR spectrum
220 210 200 190 180 170 160 150 140 130 120 110 100 90 80 70 60 50 40 30 20 10 0
Chemical Shift (ppm)

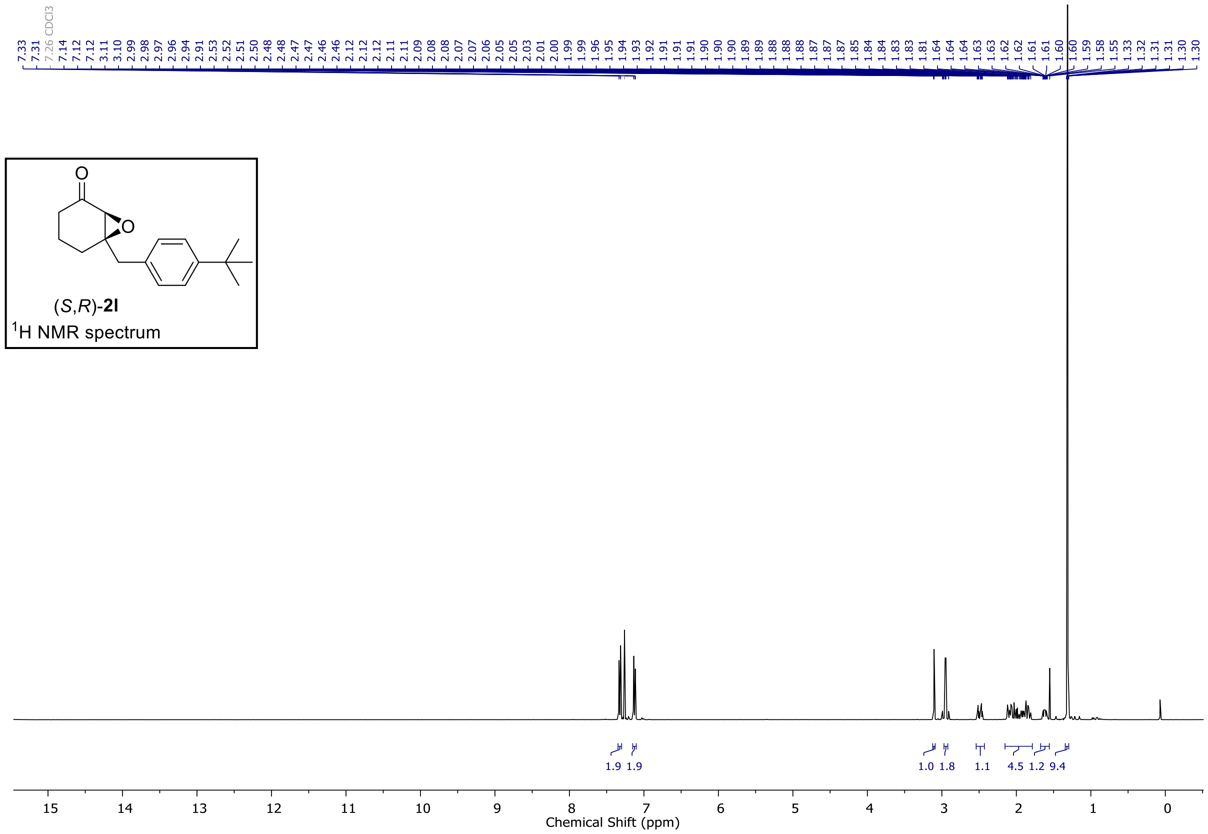
(S,R)-2l
1H NMR spectrum
Chemical Shift (ppm)

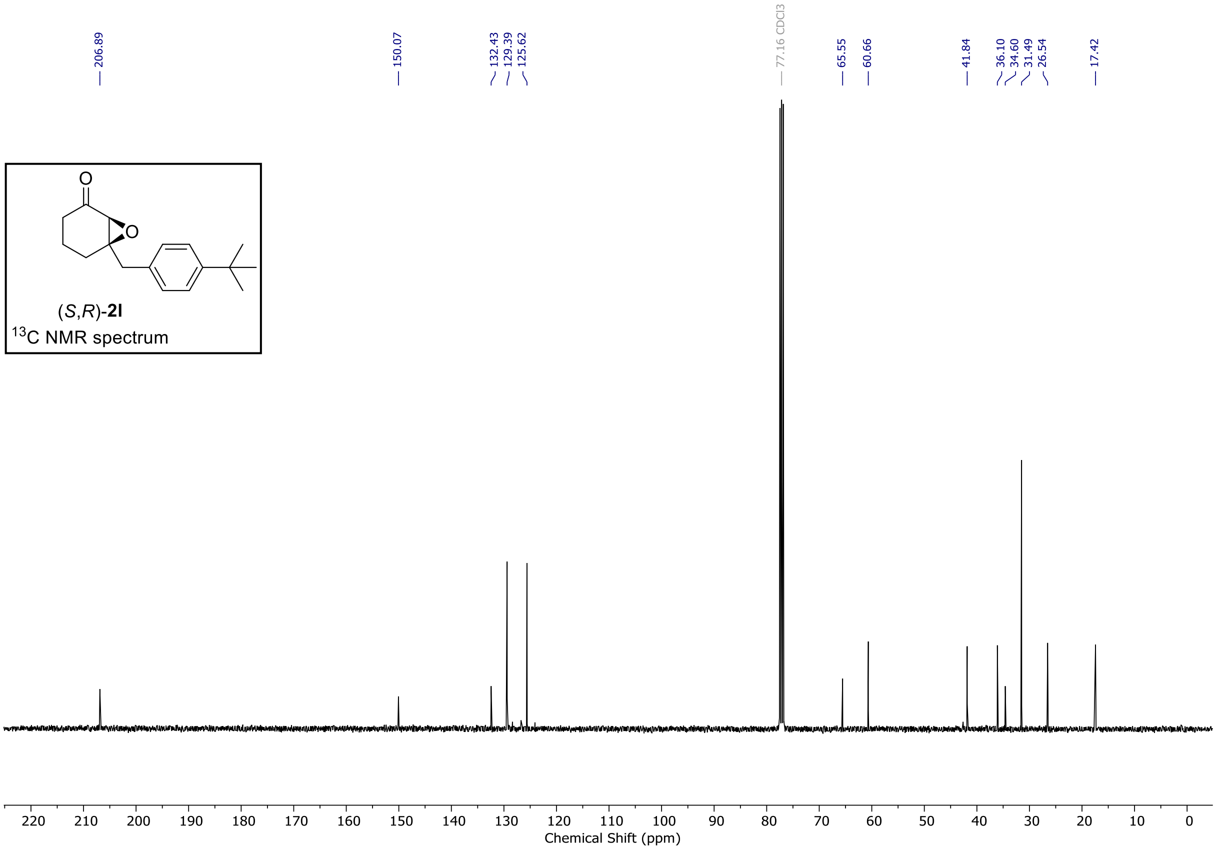
(S,R)-2l
13C NMR spectrum
Chemical Shift (ppm)

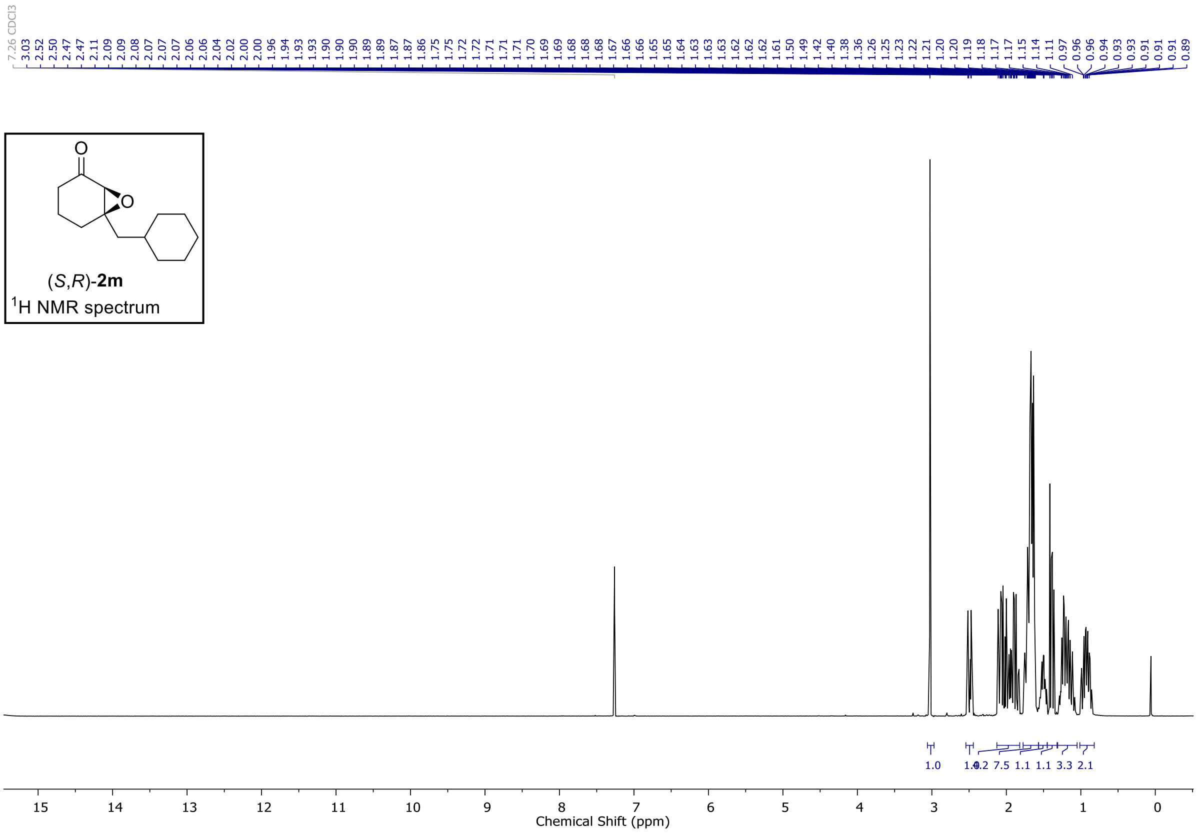
(S,R)-2m
1H NMR spectrum
Chemical Shift (ppm)

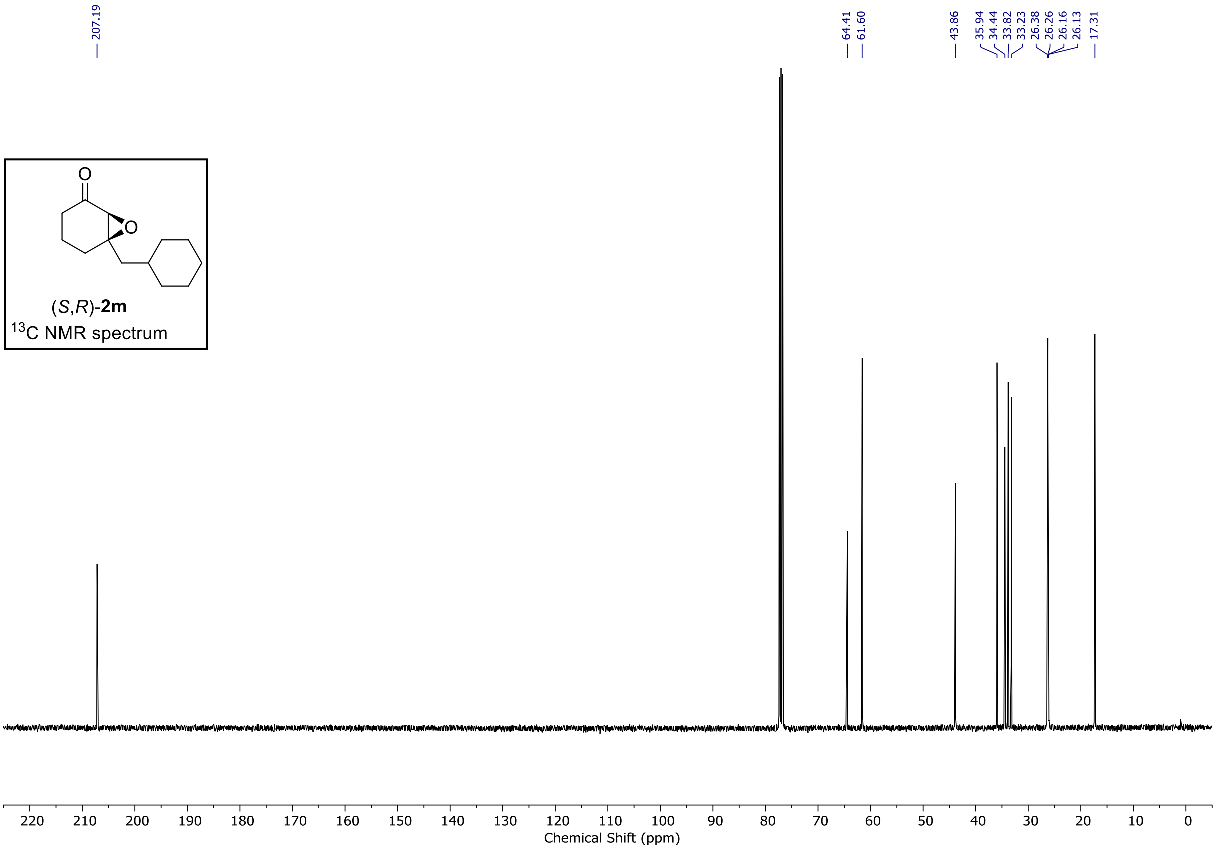
(S,R)-2m
13C NMR spectrum
207.19
64.41
61.60
43.86
35.94
34.44
33.82
33.23
26.38
26.26
26.16
26.13
17.31
Chemical Shift (ppm)

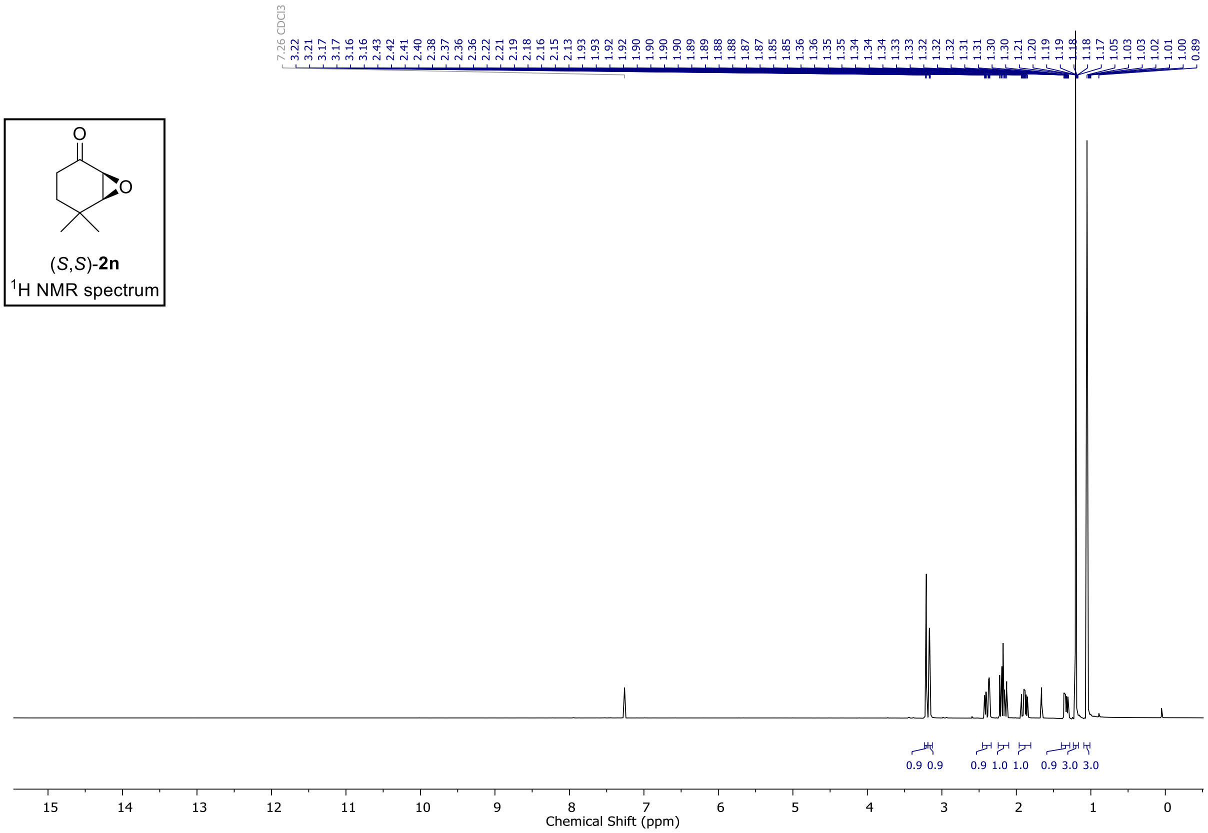
(S,S)-2n
1H NMR spectrum
Chemical Shift (ppm)

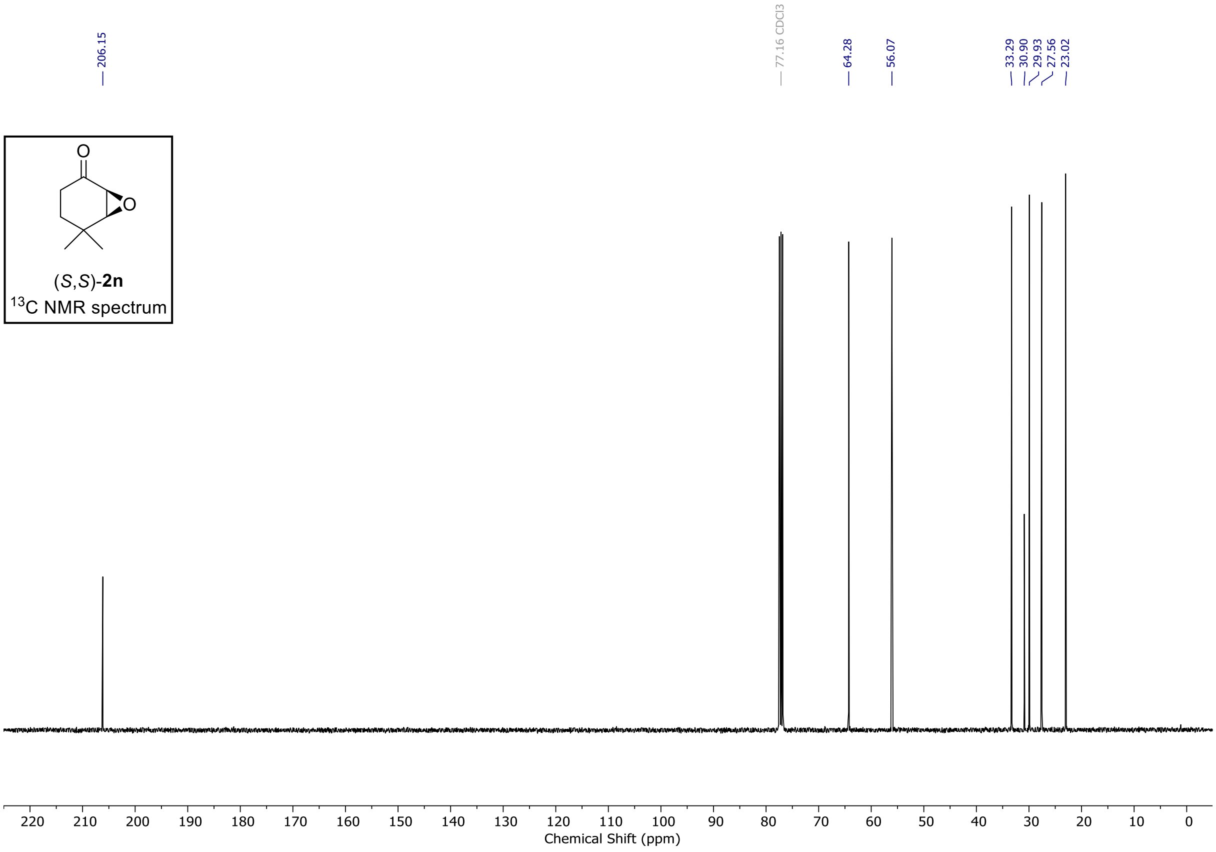
(S,S)-2n
13C NMR spectrum
206.15
77.16 CDCl3
64.28
56.07
33.29
30.90
29.93
27.56
23.02
Chemical Shift (ppm)

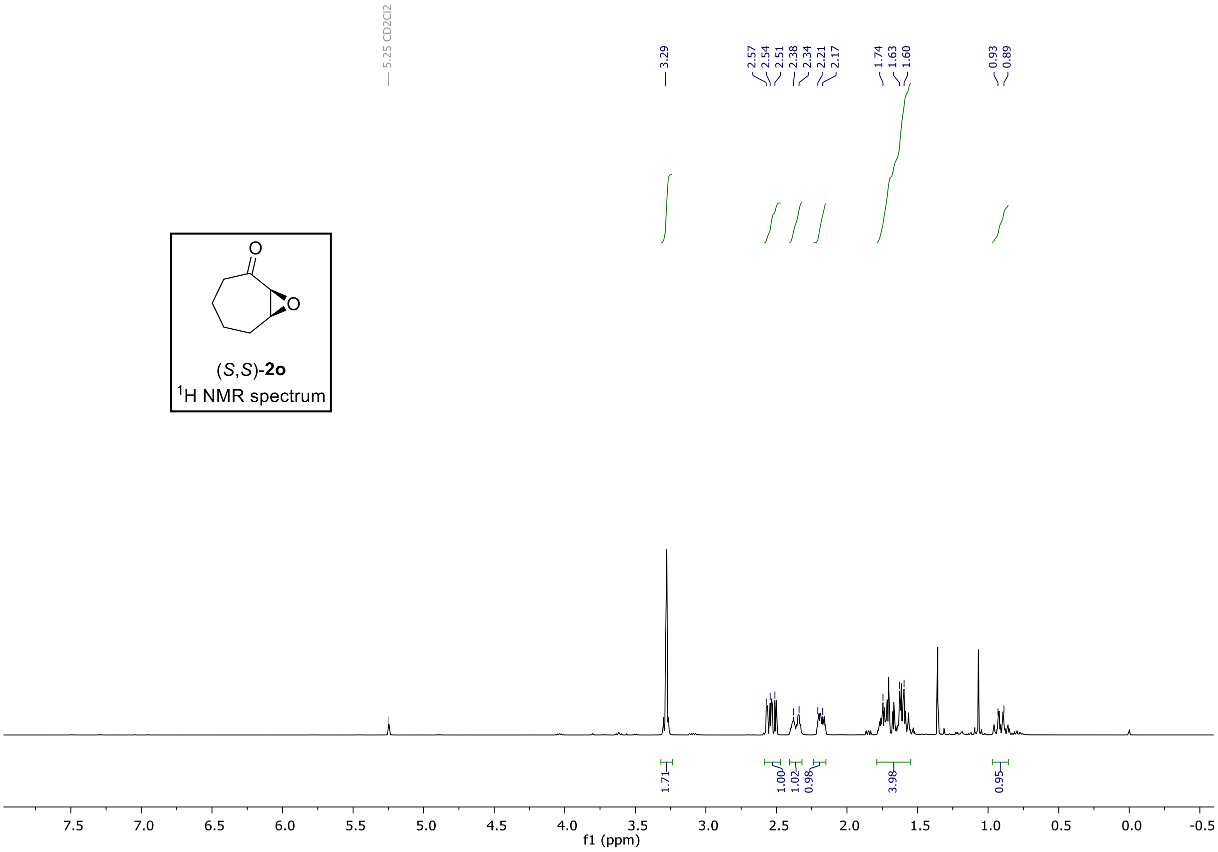
5.25 CD2Cl2
3.29
2.57
2.54
2.51
2.38
2.34
2.21
2.17
1.74
1.63
1.60
0.93
0.89
O
O
(S,S)-2o
1H NMR spectrum
1.71
1.00
1.02
0.98
3.98
0.95
7.5
7.0
6.5
6.0
5.5
5.0
4.5
4.0
3.5
3.0
2.5
2.0
1.5
1.0
0.5
0.0
-0.5
f1 (ppm)

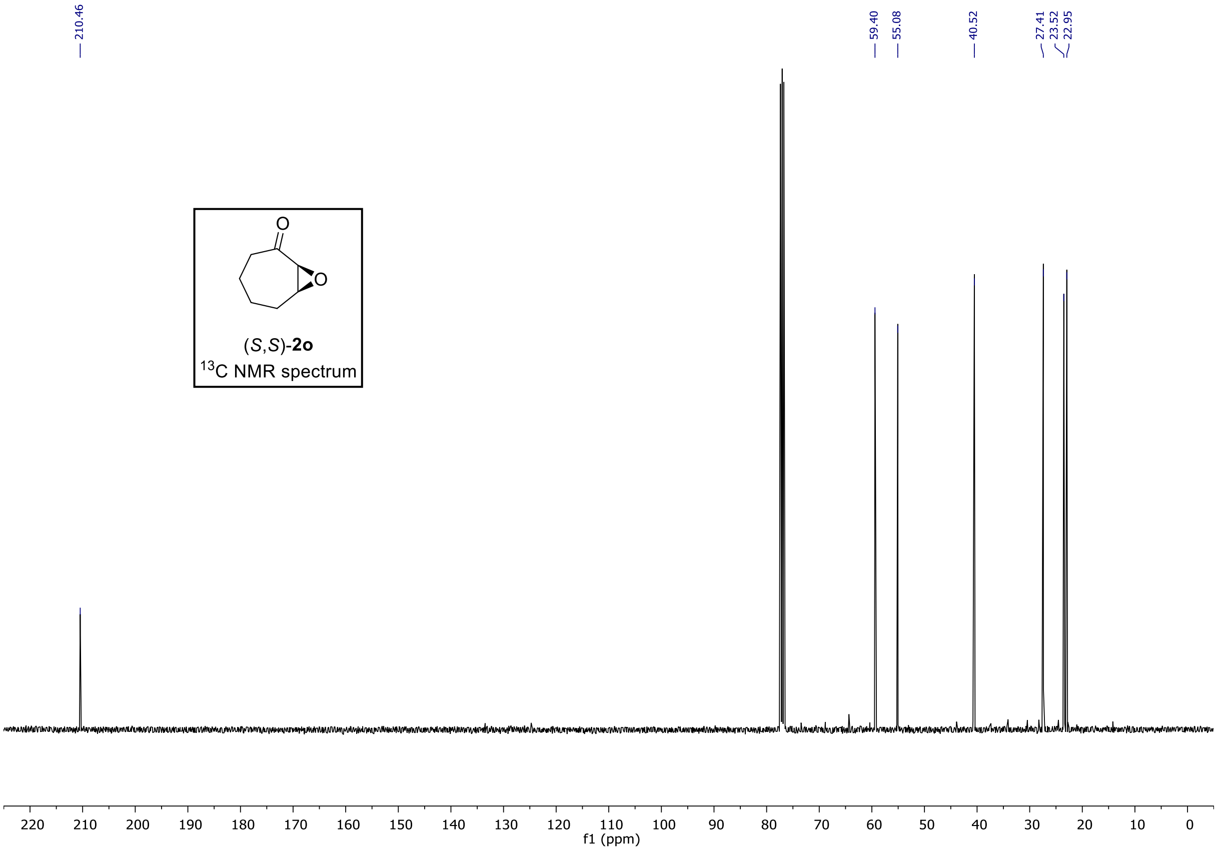
210.46
59.40
55.08
40.52
27.41
23.52
22.95
O
O
(S,S)-2o
13C NMR spectrum
220
210
200
190
180
170
160
150
140
130
120
110
100
90
80
70
60
50
40
30
20
10
0
f1 (ppm)

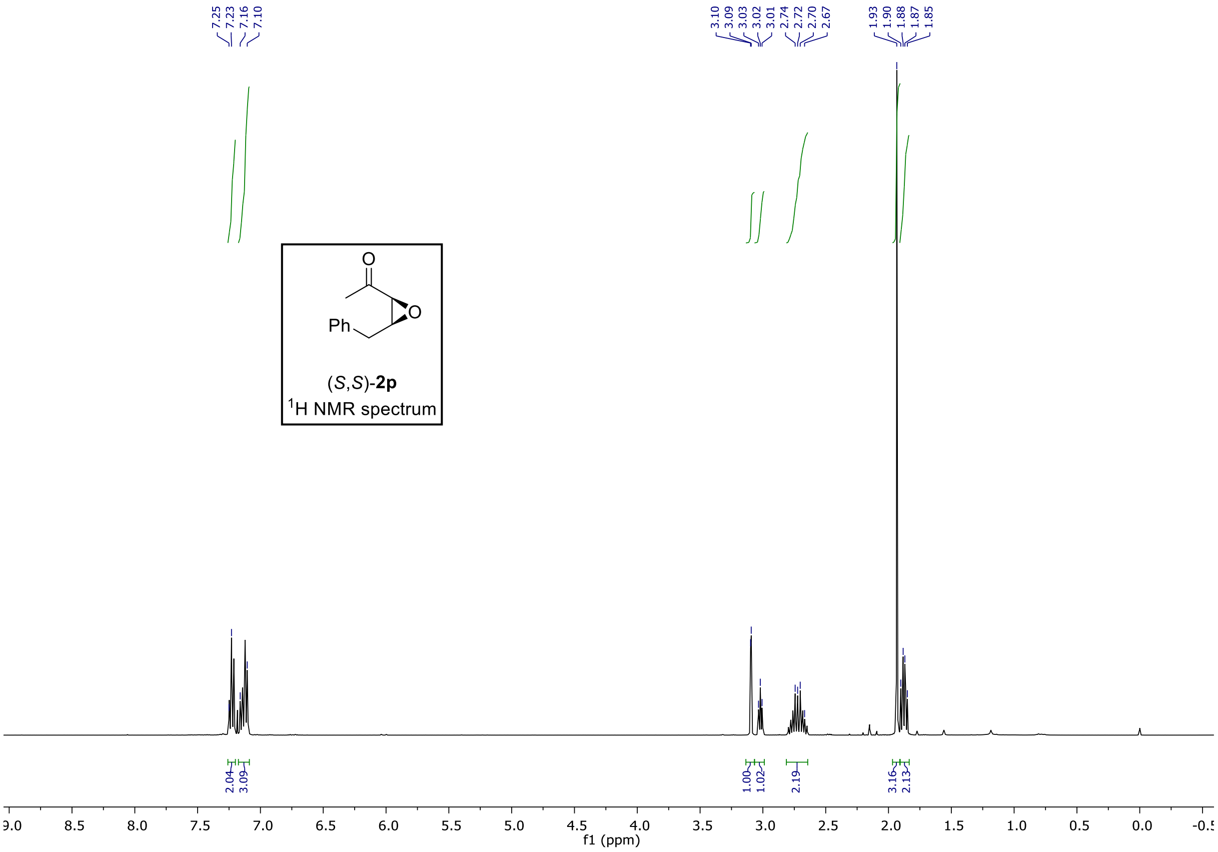
7.25
7.23
7.16
7.10
3.10
3.09
3.03
3.02
3.01
2.74
2.72
2.70
2.67
1.93
1.90
1.88
1.87
1.85
O
O
Ph
(S,S)-2p
1H NMR spectrum
2.04
3.09
1.00
1.02
2.19
3.16
2.13
f1 (ppm)

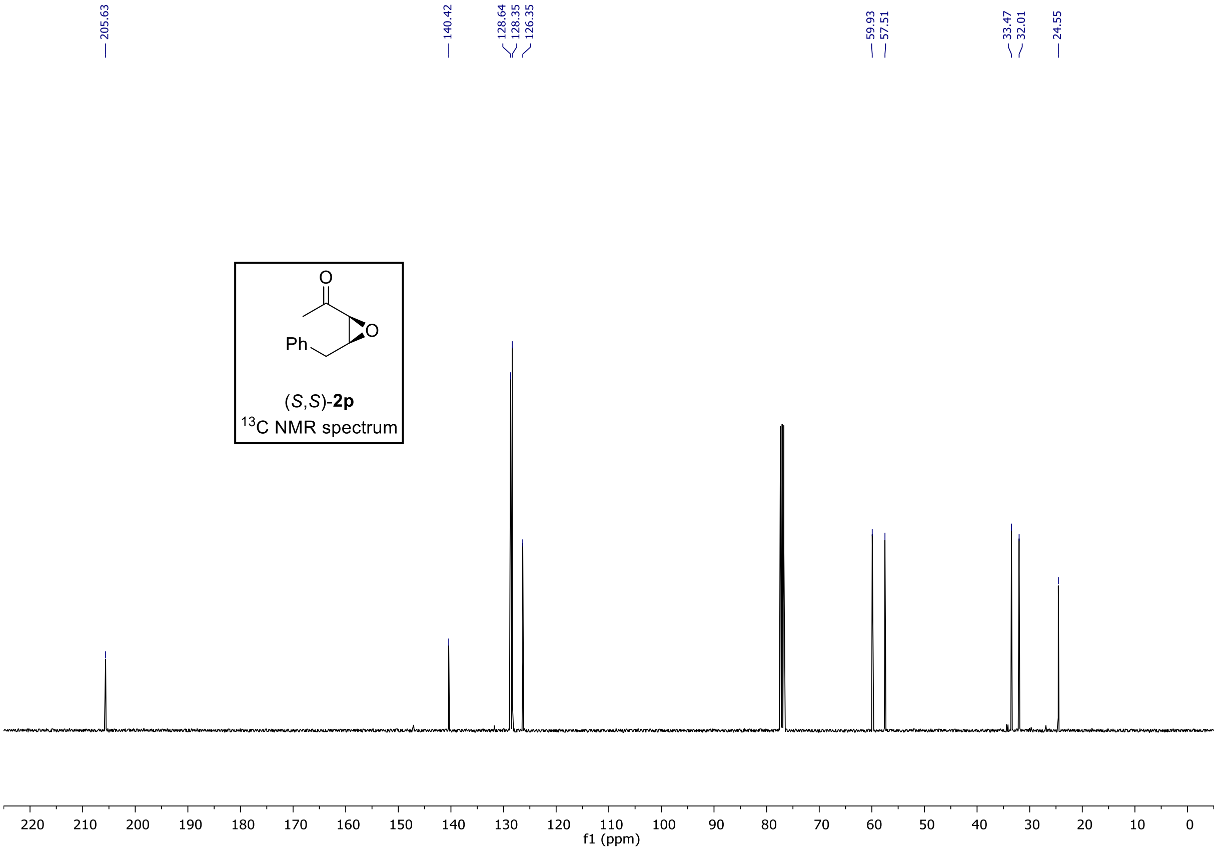
205.63
140.42
128.64
128.35
126.35
59.93
57.51
33.47
32.01
24.55
O
O
Ph
(S,S)-2p
13C NMR spectrum
f1 (ppm)

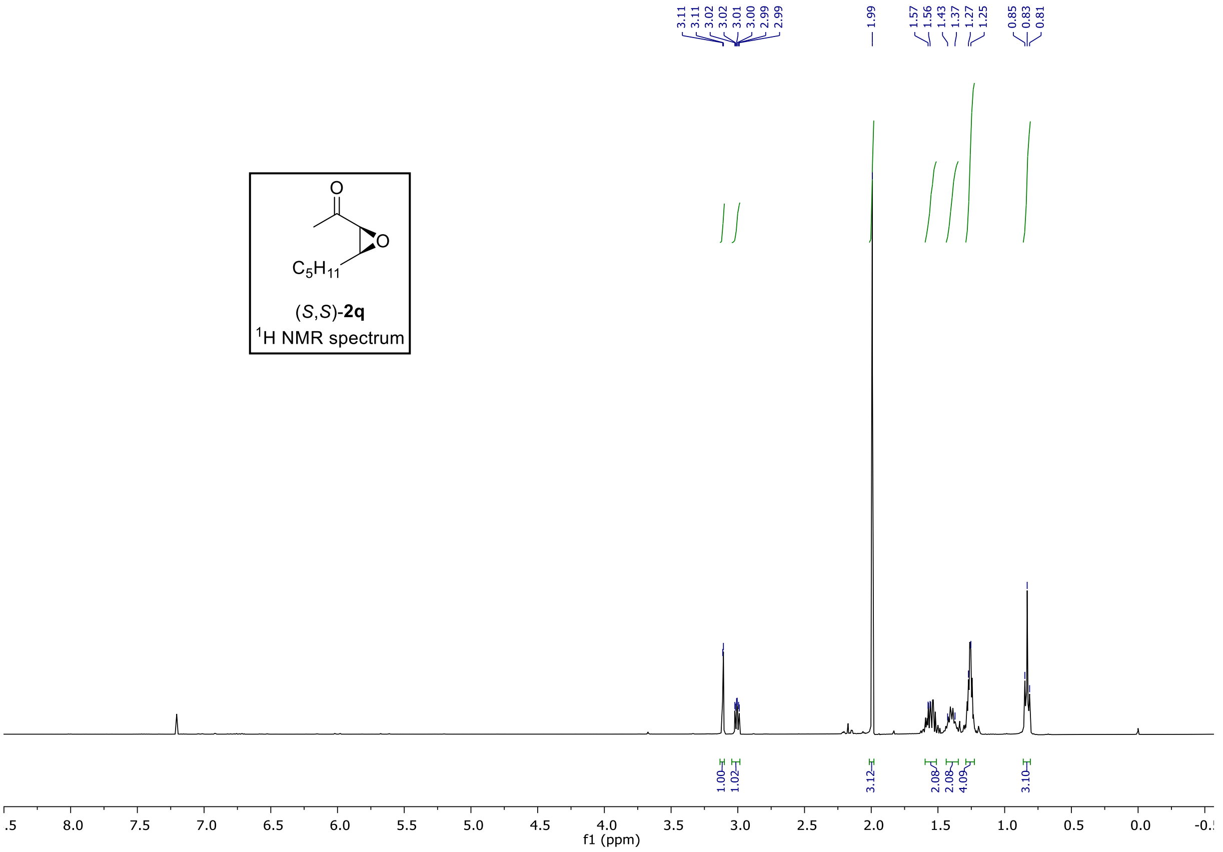
(S,S)-2q
1H NMR spectrum
3.11
3.11
3.02
3.02
3.01
3.00
2.99
2.99
1.99
1.57
1.56
1.43
1.37
1.27
1.25
0.85
0.83
0.81
1.00
1.02
3.12
2.08
2.08
4.09
3.10
f1 (ppm)

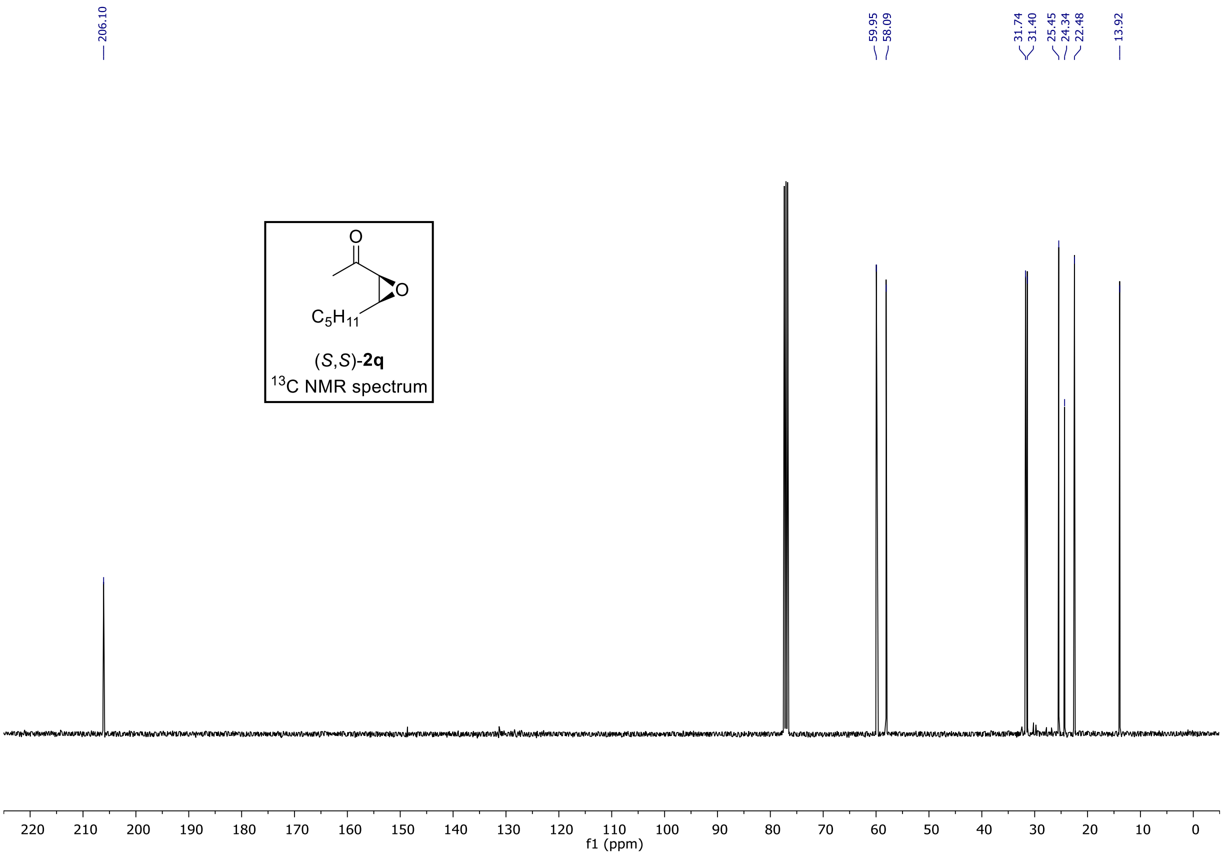
(S,S)-2q
13C NMR spectrum
206.10
59.95
58.09
31.74
31.40
25.45
24.34
22.48
13.92
f1 (ppm)

19. NMR spectra for the asymmetric aziridinations

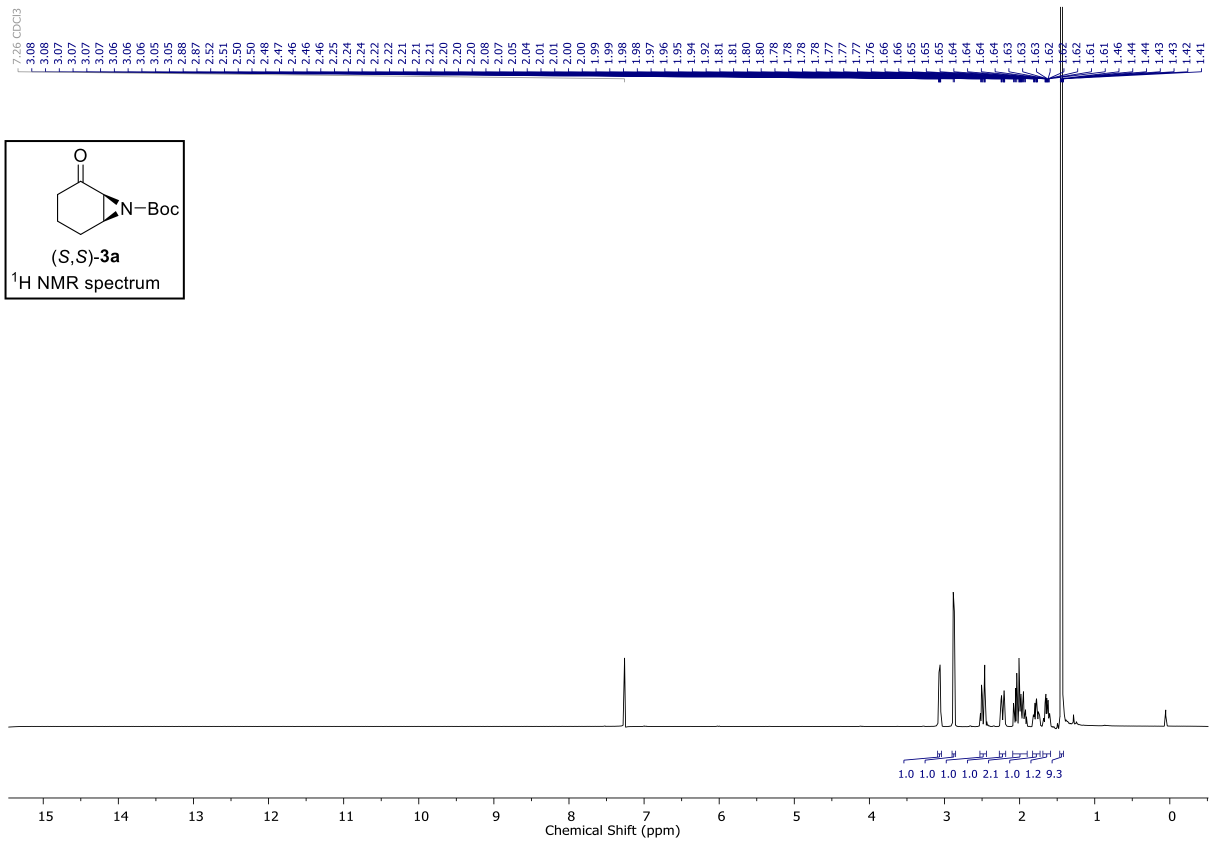

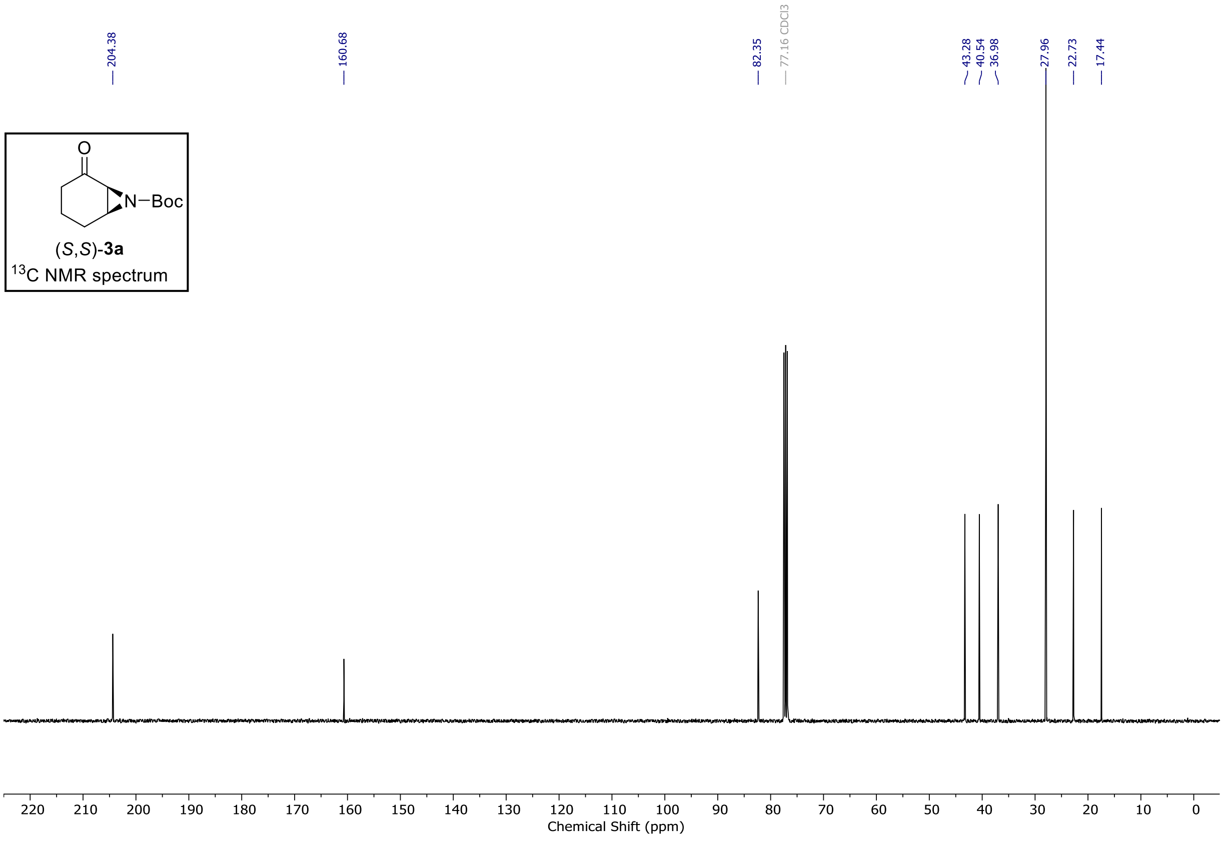

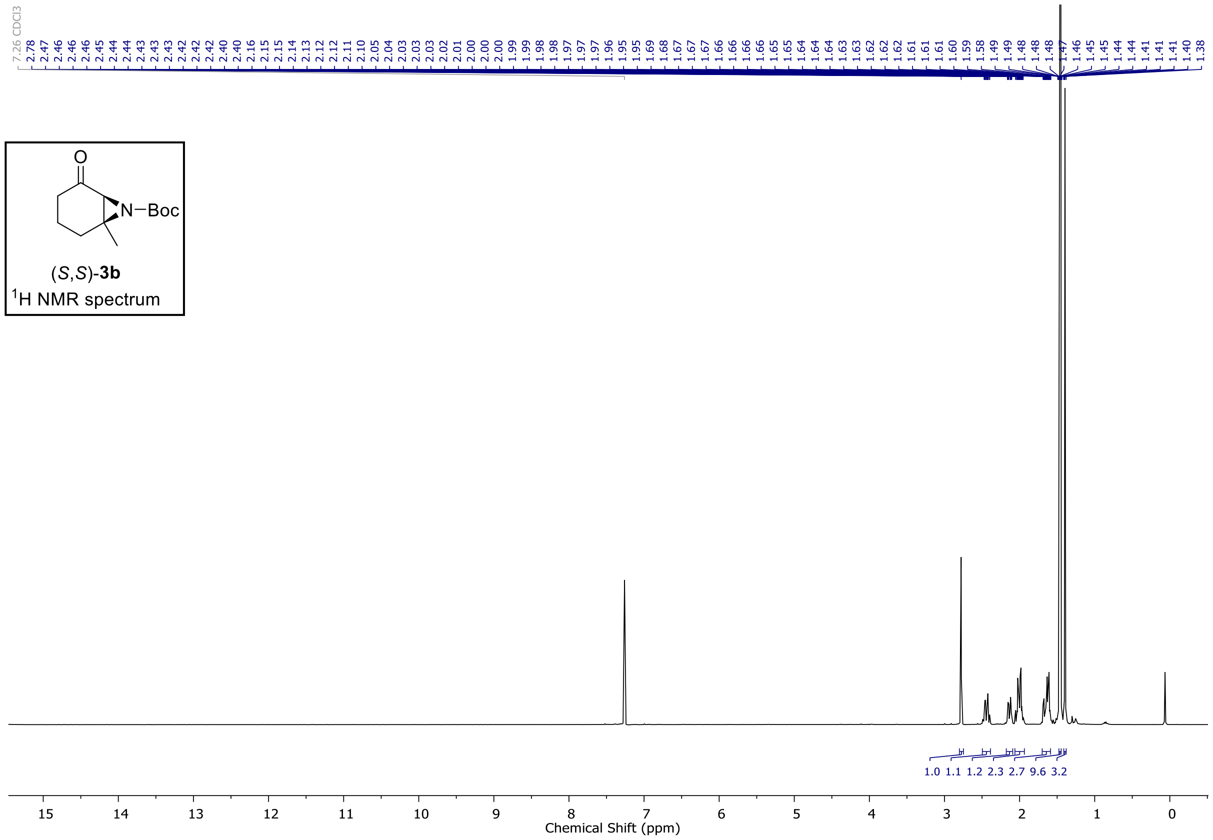
N−Boc
(S,S)-3b
1H NMR spectrum
Chemical Shift (ppm)

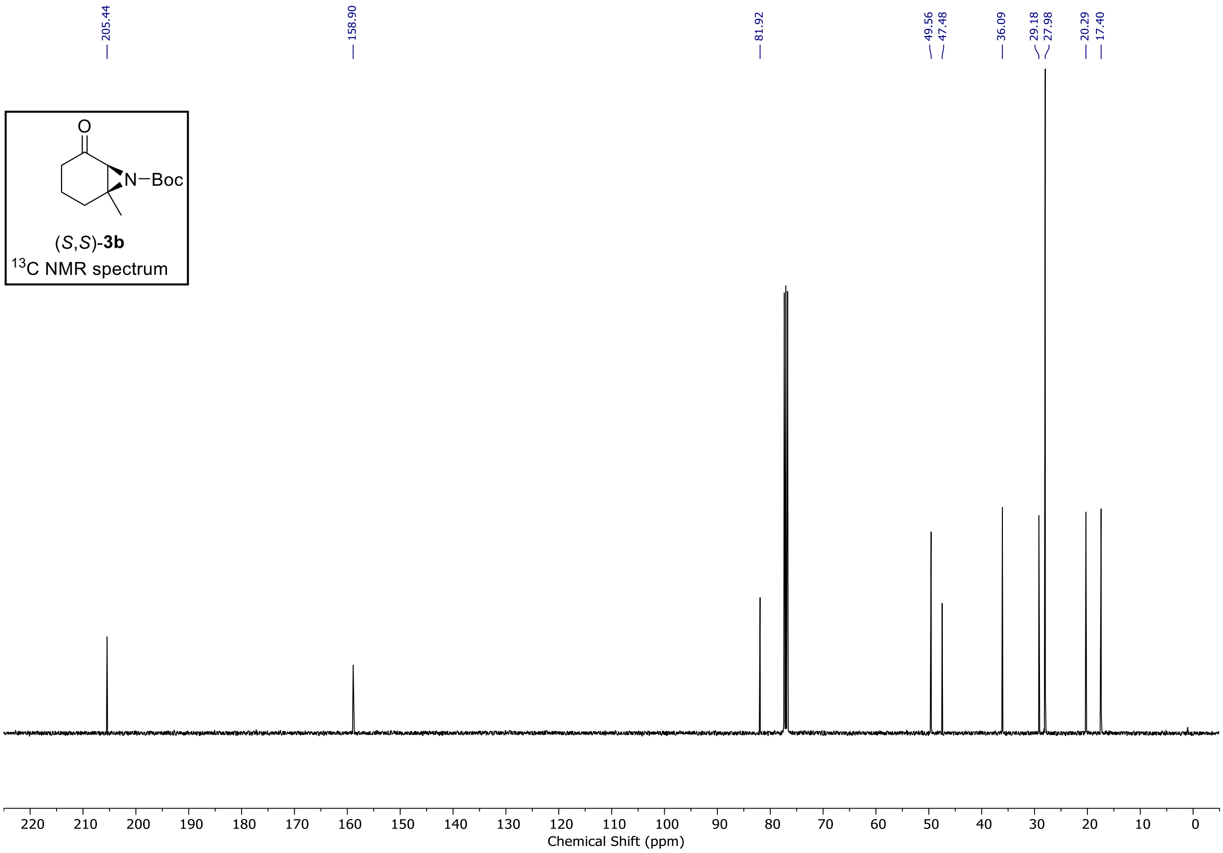
N−Boc
(S,S)-3b
13C NMR spectrum
205.44
158.90
81.92
49.56
47.48
36.09
29.18
27.98
20.29
17.40
Chemical Shift (ppm)

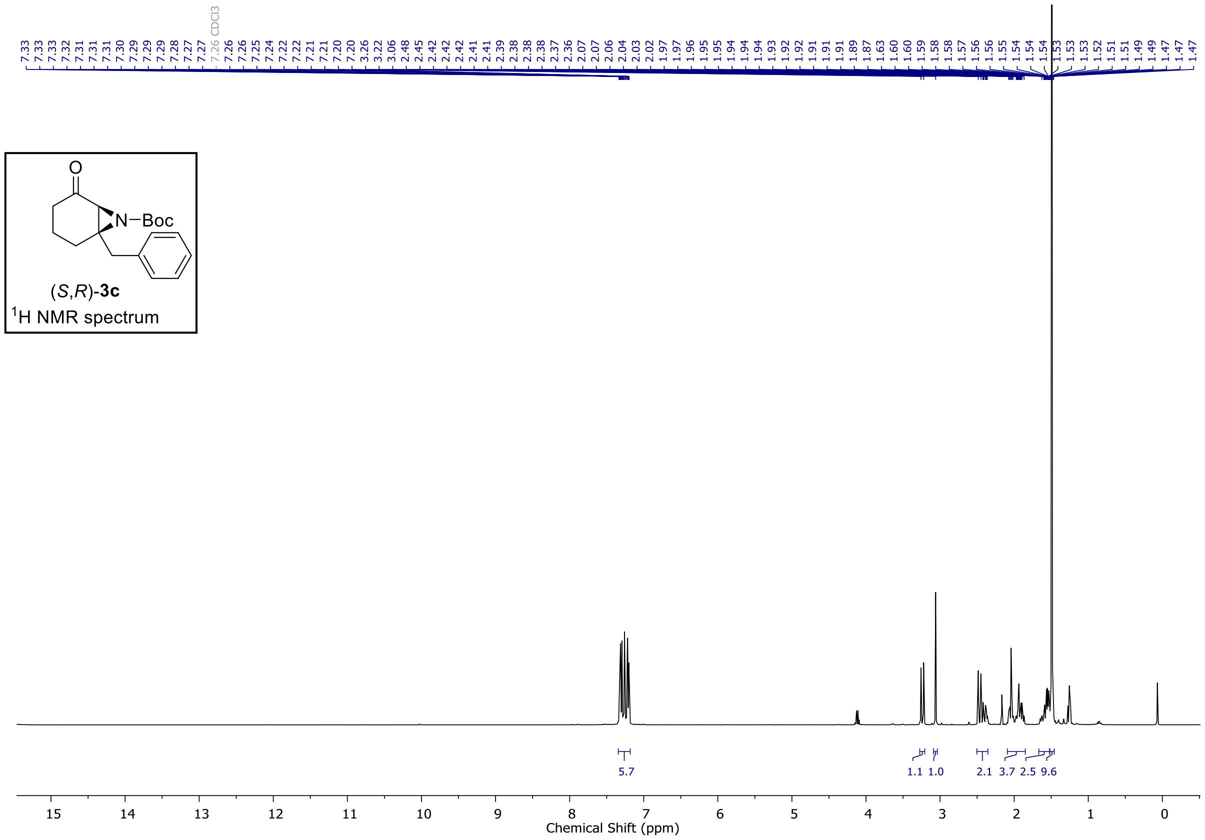
7.33 7.33 7.33 7.32 7.31 7.31 7.31 7.30 7.29 7.29 7.29 7.28 7.27 7.27 7.26 CDCl3 7.26 7.26 7.25 7.24 7.22 7.22 7.21 7.21 7.20 7.20 3.26 3.22 3.06 2.48 2.45 2.42 2.42 2.42 2.41 2.41 2.39 2.38 2.38 2.38 2.37 2.36 2.07 2.06 2.04 2.03 2.02 1.97 1.97 1.96 1.95 1.95 1.94 1.94 1.93 1.92 1.92 1.91 1.91 1.91 1.89 1.87 1.63 1.60 1.59 1.58 1.58 1.57 1.56 1.56 1.55 1.54 1.54 1.54 1.53 1.53 1.53 1.52 1.51 1.49 1.47 1.47
O
N−Boc
(S,R)-3c
1H NMR spectrum
5.7
1.1 1.0
2.1 3.7 2.5 9.6
15 14 13 12 11 10 9 8 7 6 5 4 3 2 1 0
Chemical Shift (ppm)

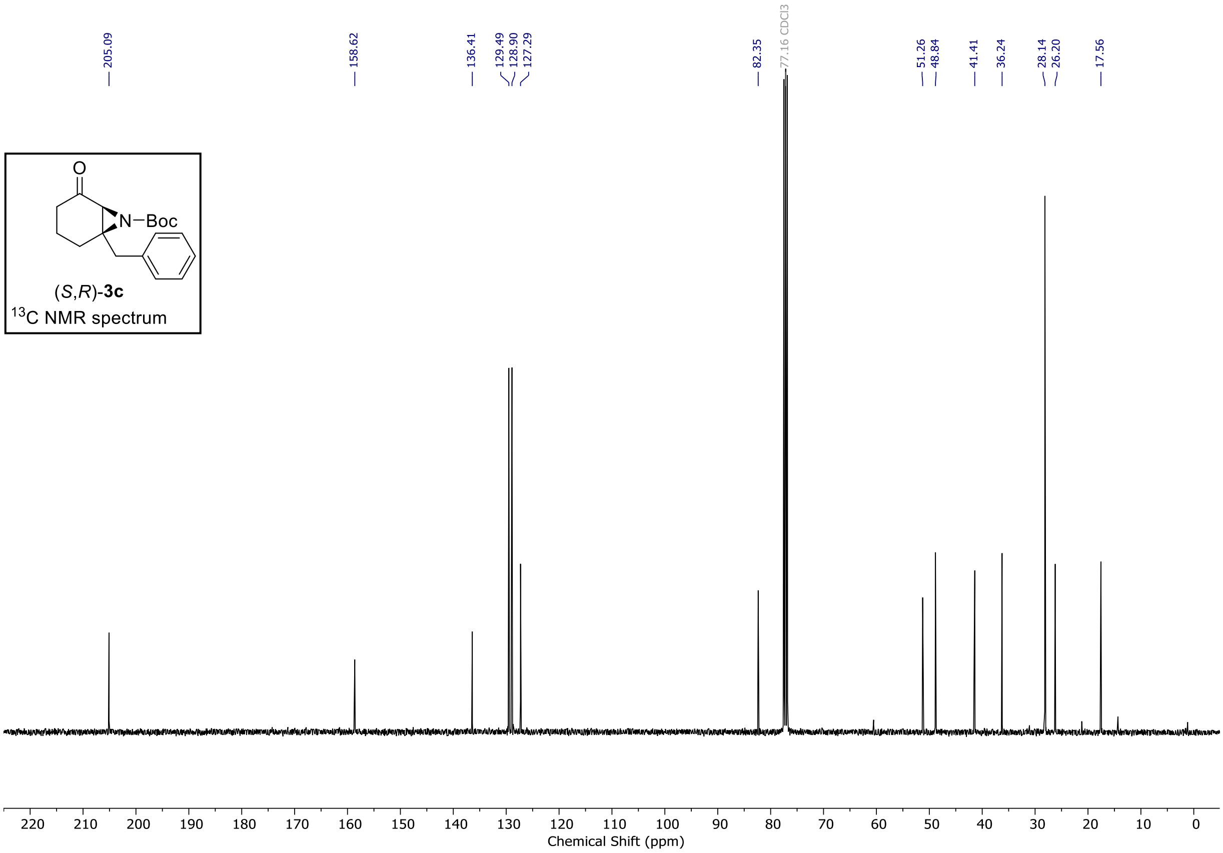
205.09
158.62
136.41
129.49 128.90 127.29
82.35
77.16 CDCl3
51.26 48.84
41.41
36.24
28.14 26.20
17.56
O
N−Boc
(S,R)-3c
13C NMR spectrum
220 210 200 190 180 170 160 150 140 130 120 110 100 90 80 70 60 50 40 30 20 10 0
Chemical Shift (ppm)

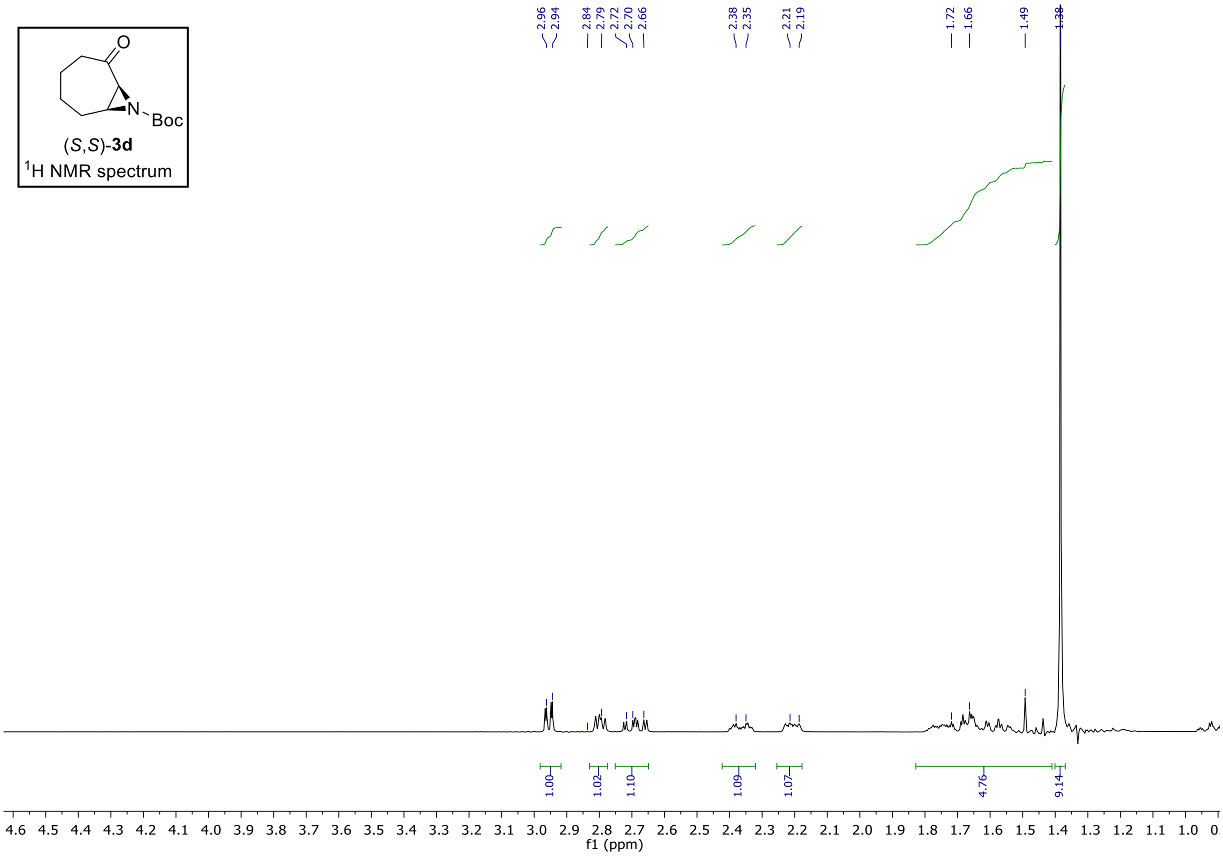
(S,S)-3d
1H NMR spectrum
Boc
2.96 2.94 2.84 2.79 2.72 2.70 2.66 2.38 2.35 2.21 2.19 1.72 1.66 1.49 1.38
1.00 1.02 1.10 1.09 1.07 4.76 9.14
f1 (ppm)

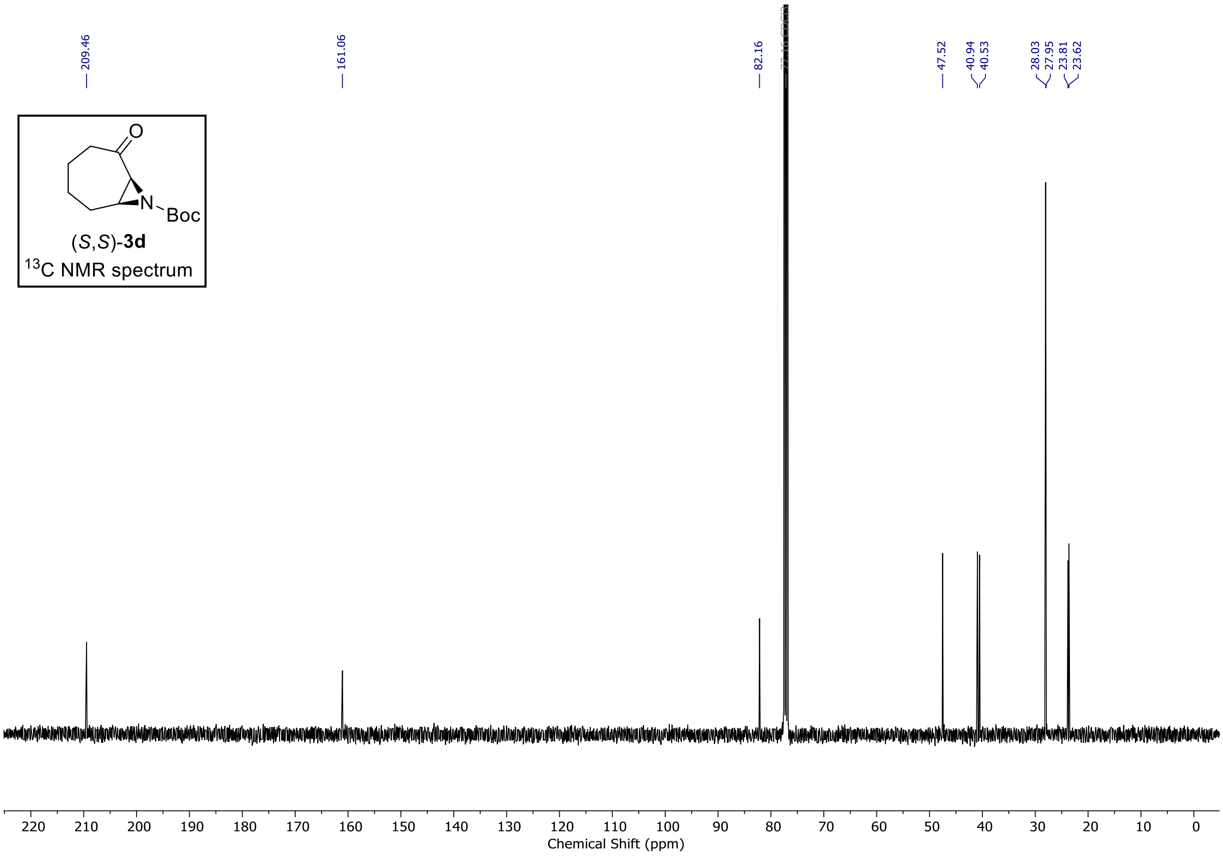
(S,S)-3d
13C NMR spectrum
Boc
209.46 161.06 82.16 47.52 40.94 40.53 28.03 27.95 23.81 23.62
Chemical Shift (ppm)

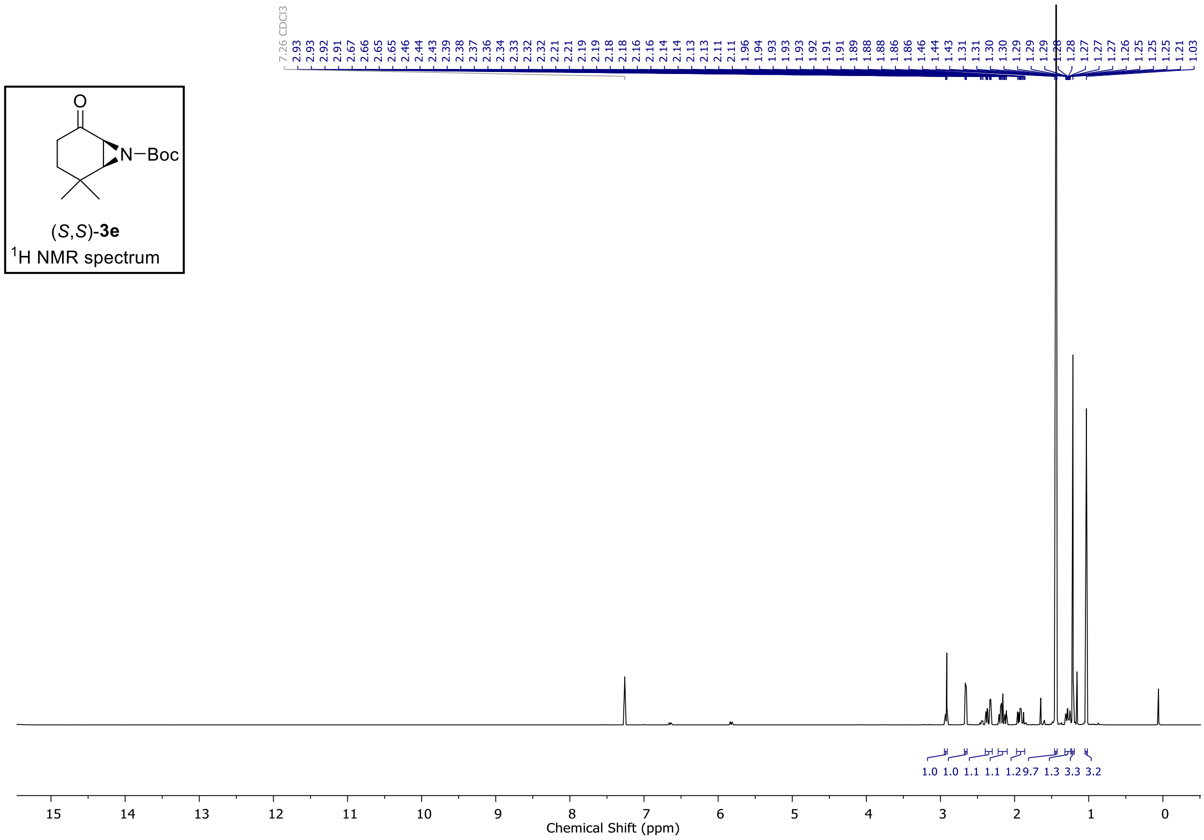
(S,S)-3e
1H NMR spectrum
N−Boc
Chemical Shift (ppm)

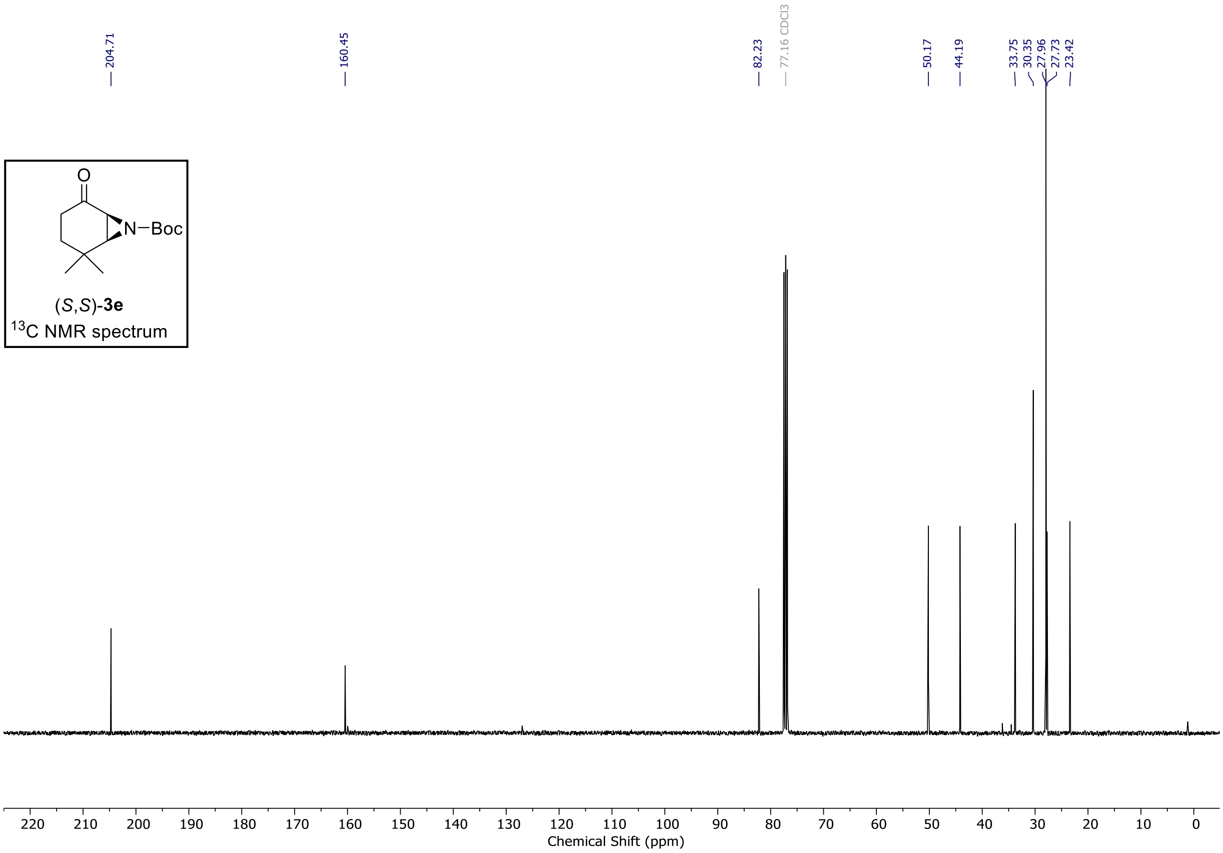
(S,S)-3e
13C NMR spectrum
N−Boc
204.71
160.45
82.23
77.16 CDCl3
50.17
44.19
33.75
30.35
27.96
27.73
23.42
Chemical Shift (ppm)

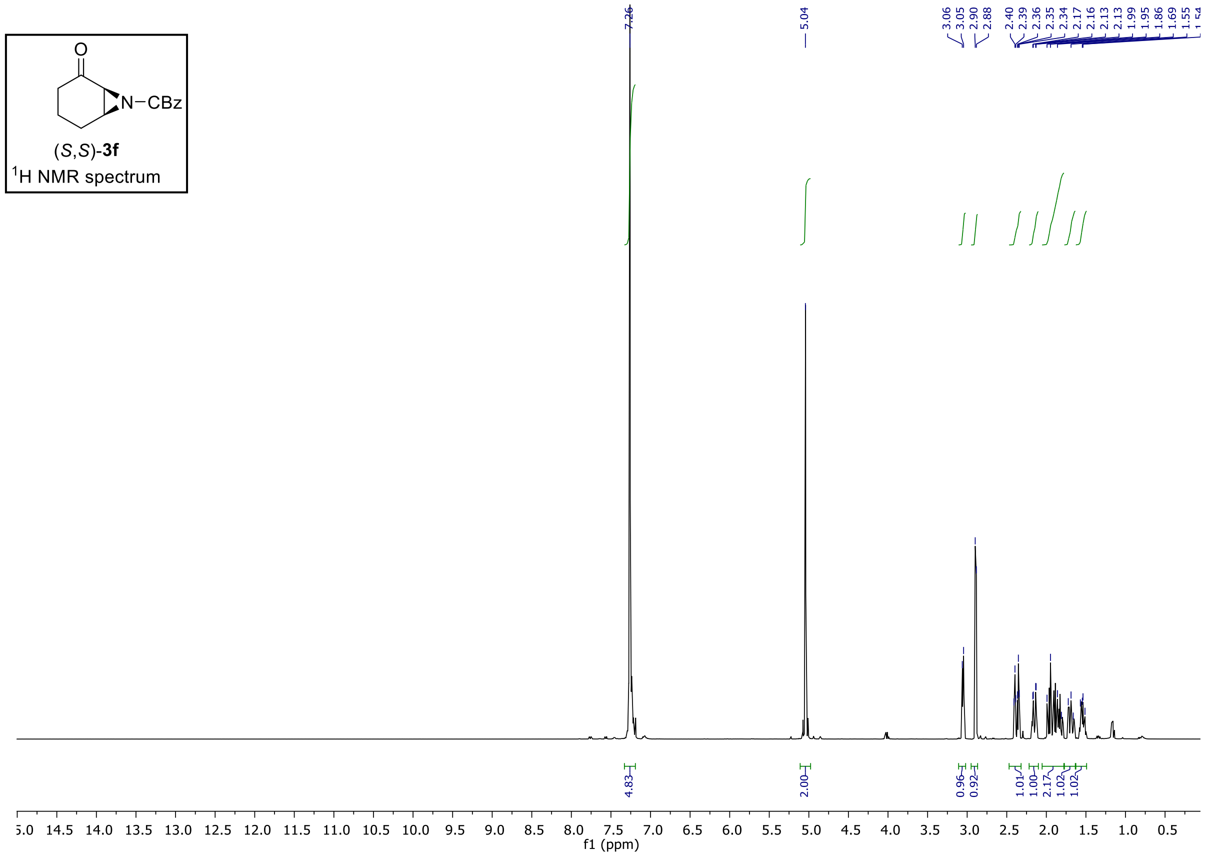
(S,S)-3f
¹H NMR spectrum
N−CBz
O
f1 (ppm)

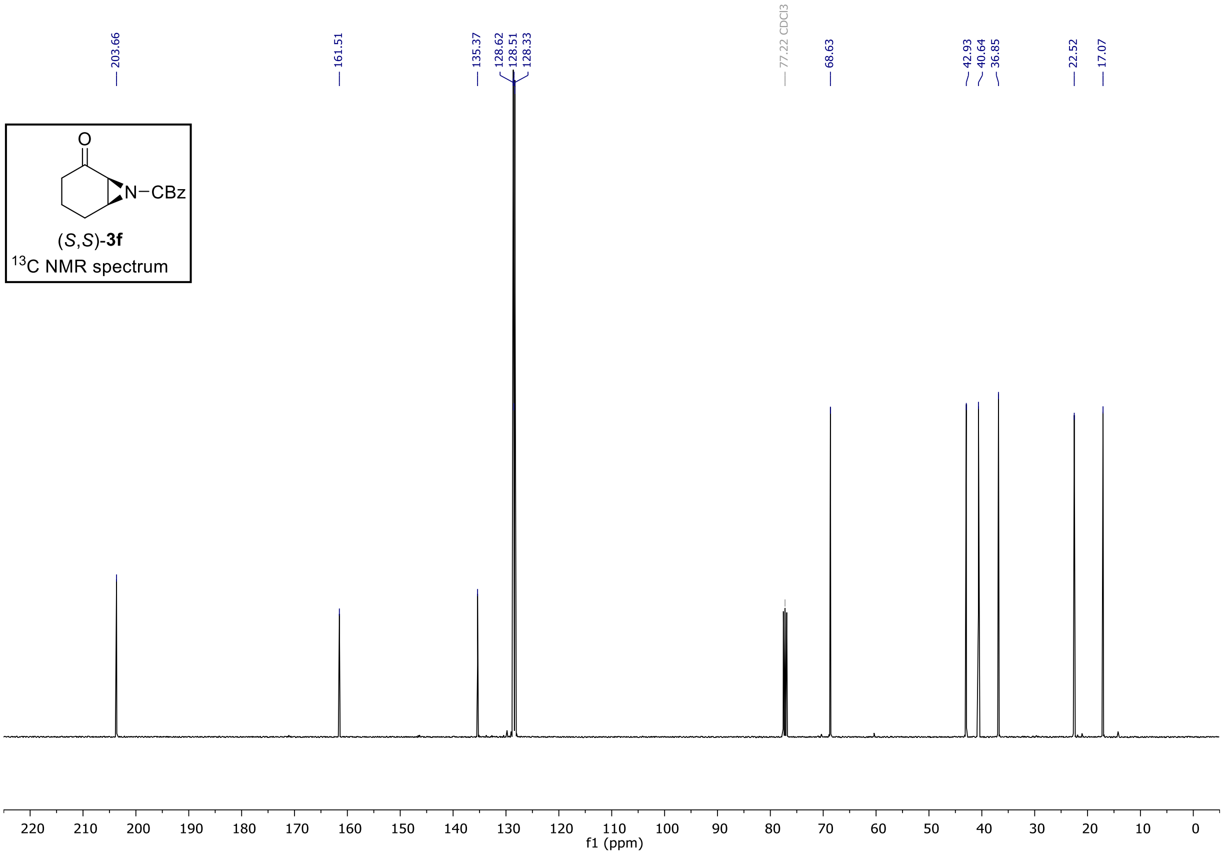
(S,S)-3f
¹³C NMR spectrum
N−CBz
O
203.66
161.51
135.37
128.62
128.51
128.33
77.22 CDCl3
68.63
42.93
40.64
36.85
22.52
17.07
f1 (ppm)

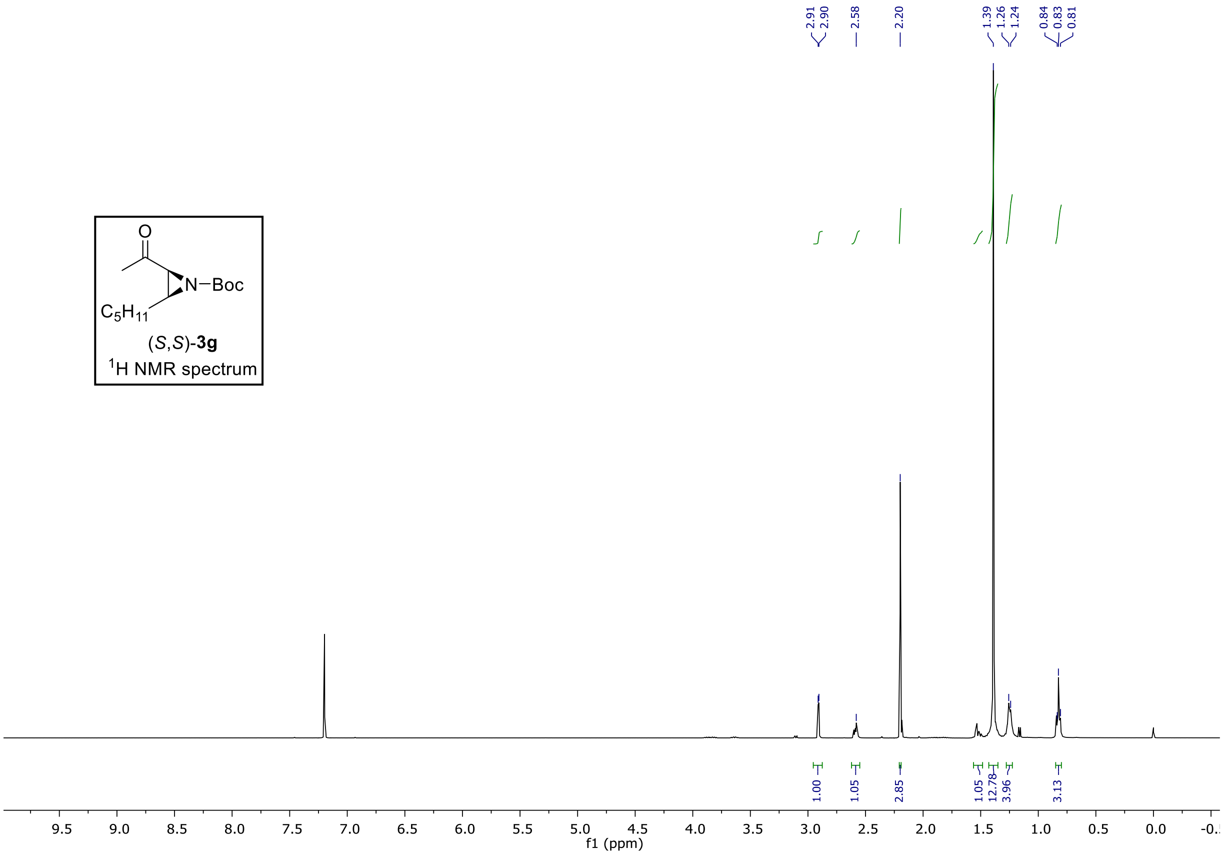
2.91
2.90
2.58
2.20
1.39
1.26
1.24
0.84
0.83
0.81
O
N−Boc
C_5H_{11}
(S,S)-3g
1H NMR spectrum
1.00
1.05
2.85
1.05
12.78
3.96
3.13
9.5 9.0 8.5 8.0 7.5 7.0 6.5 6.0 5.5 5.0 4.5 4.0 3.5 3.0 2.5 2.0 1.5 1.0 0.5 0.0
f1 (ppm)

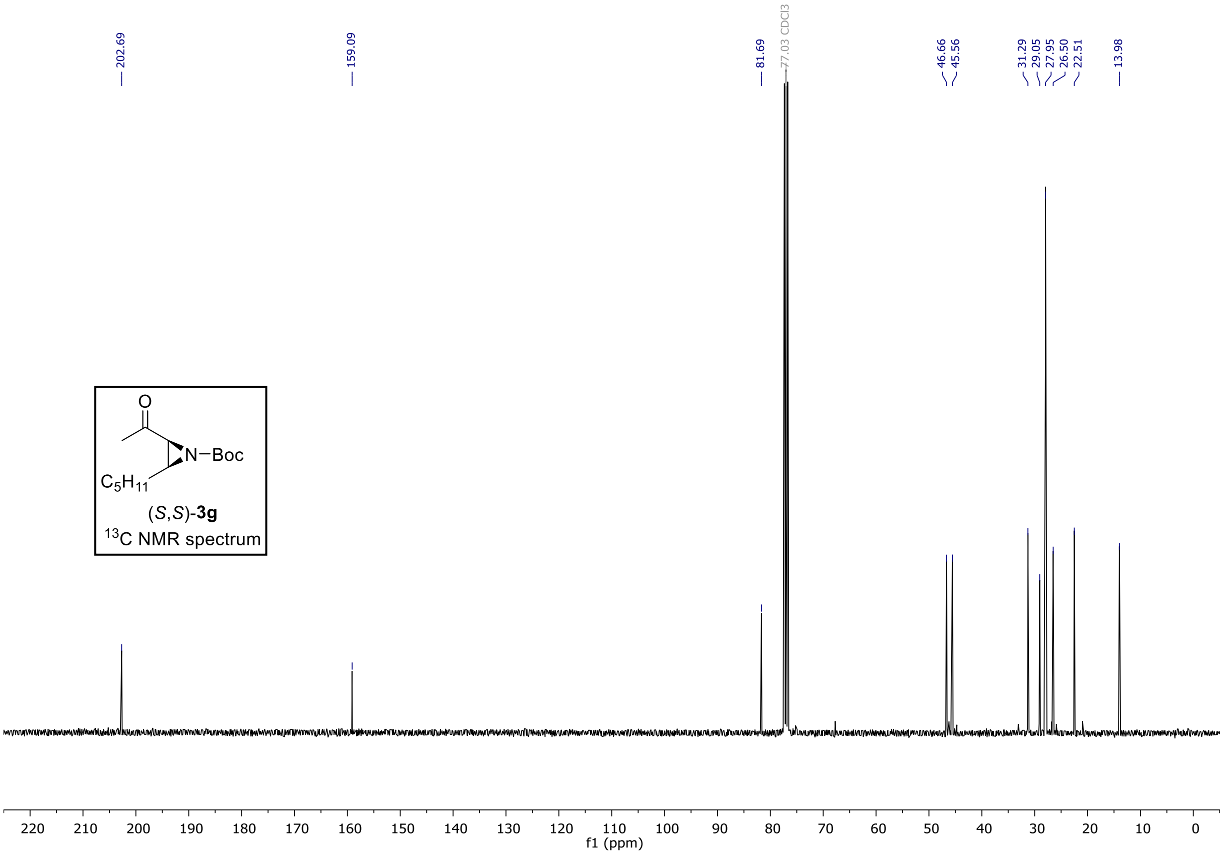
202.69
159.09
81.69
77.03 CDCl3
46.66
45.56
31.29
29.05
27.95
26.50
22.51
13.98
O
N−Boc
C_5H_{11}
(S,S)-3g
13C NMR spectrum
220 210 200 190 180 170 160 150 140 130 120 110 100 90 80 70 60 50 40 30 20 10 0
f1 (ppm)

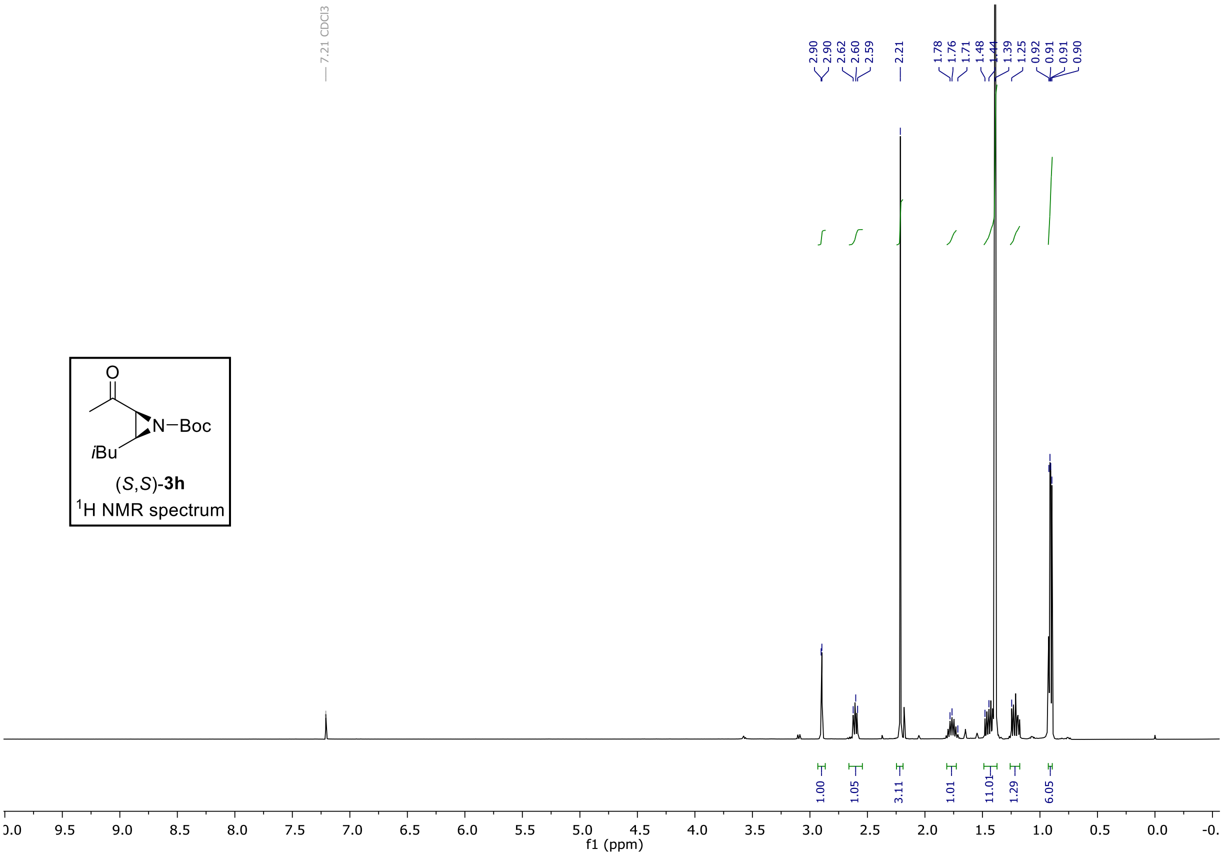
7.21 CDCl3
2.90
2.90
2.62
2.60
2.59
2.21
1.78
1.76
1.71
1.48
1.44
1.39
1.25
0.92
0.91
0.91
0.90
N−Boc
iBu
(S,S)-3h
1H NMR spectrum
1.00
1.05
3.11
1.01
11.01
1.29
6.05
f1 (ppm)

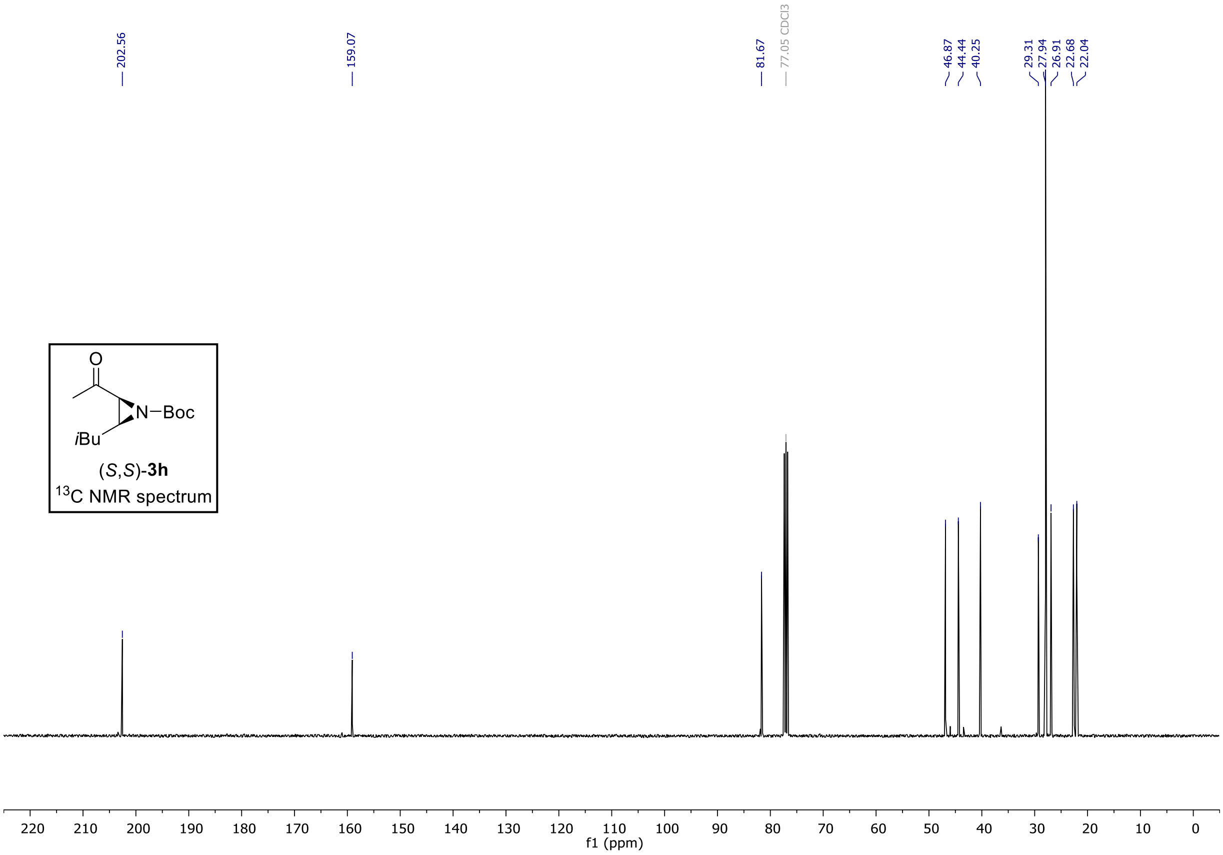
202.56
159.07
81.67
77.05 CDCl3
46.87
44.44
40.25
29.31
27.94
26.91
22.68
22.04
N−Boc
iBu
(S,S)-3h
13C NMR spectrum
f1 (ppm)

20. NMR spectra of asymmetric Aza-michael/aldol reactions

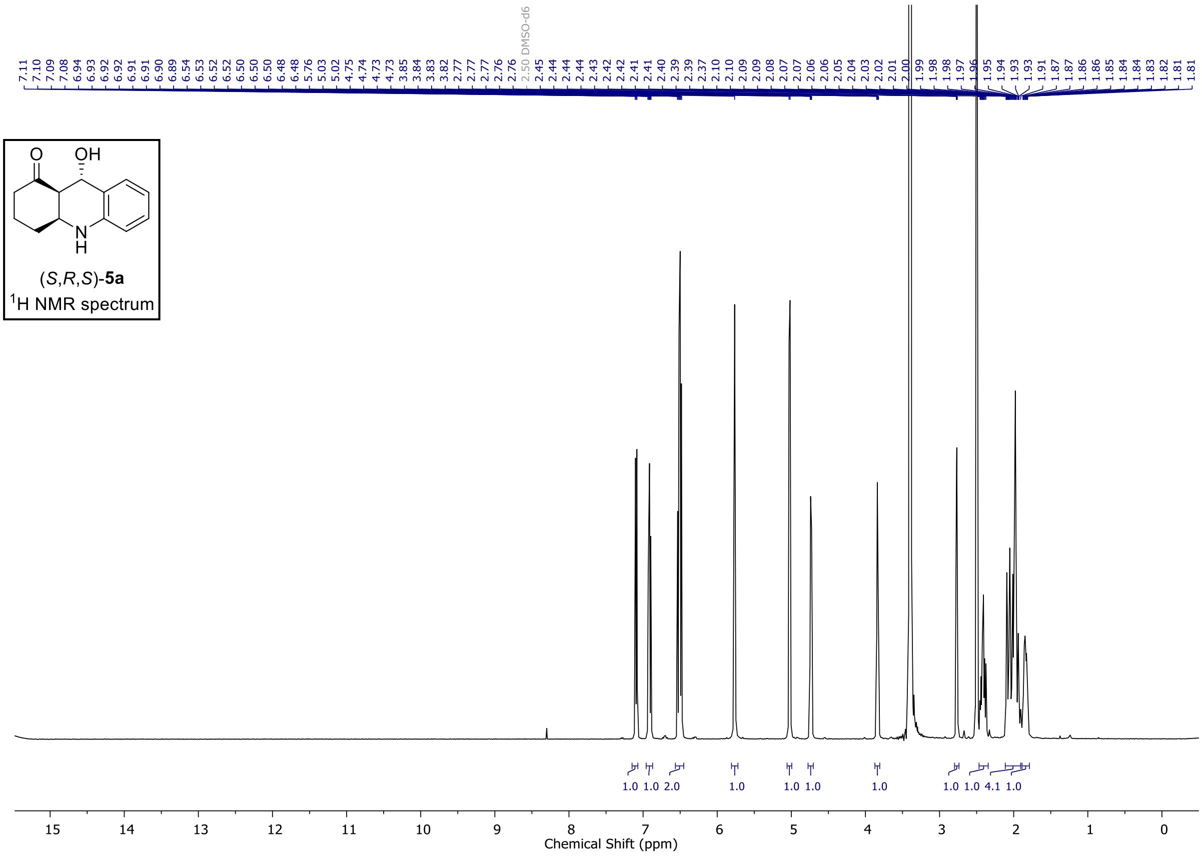

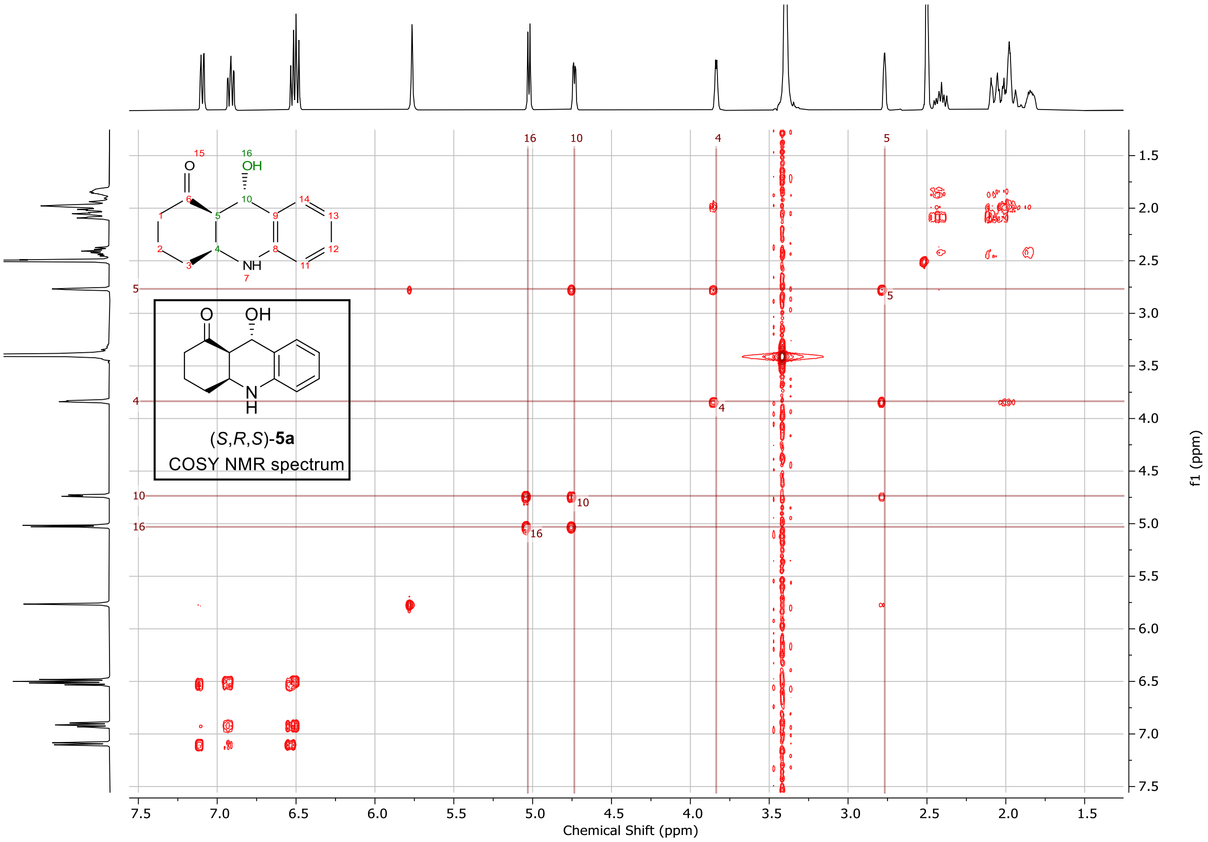

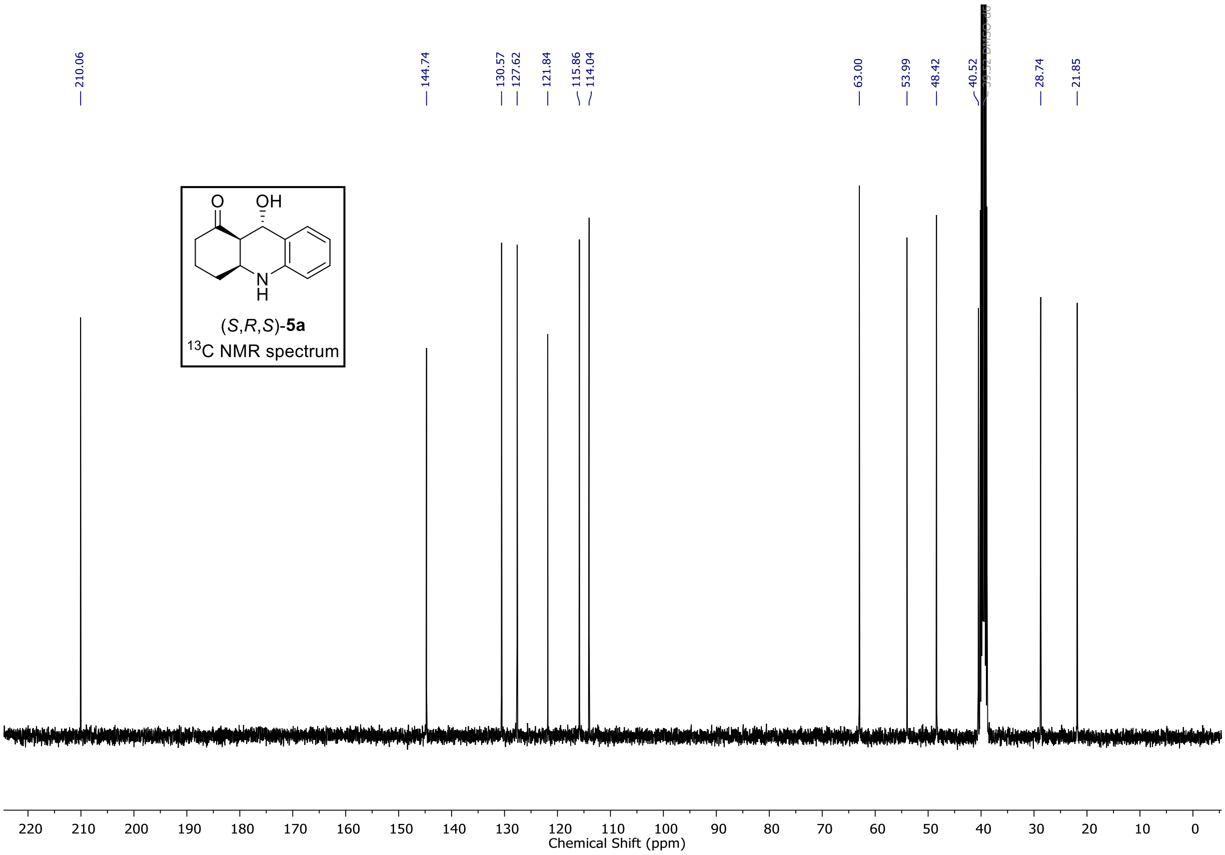
210.06
144.74
130.57
127.62
121.84
115.86
114.04
63.00
53.99
48.42
40.52
28.74
21.85
(S,R,S)-5a
13C NMR spectrum
Chemical Shift (ppm)

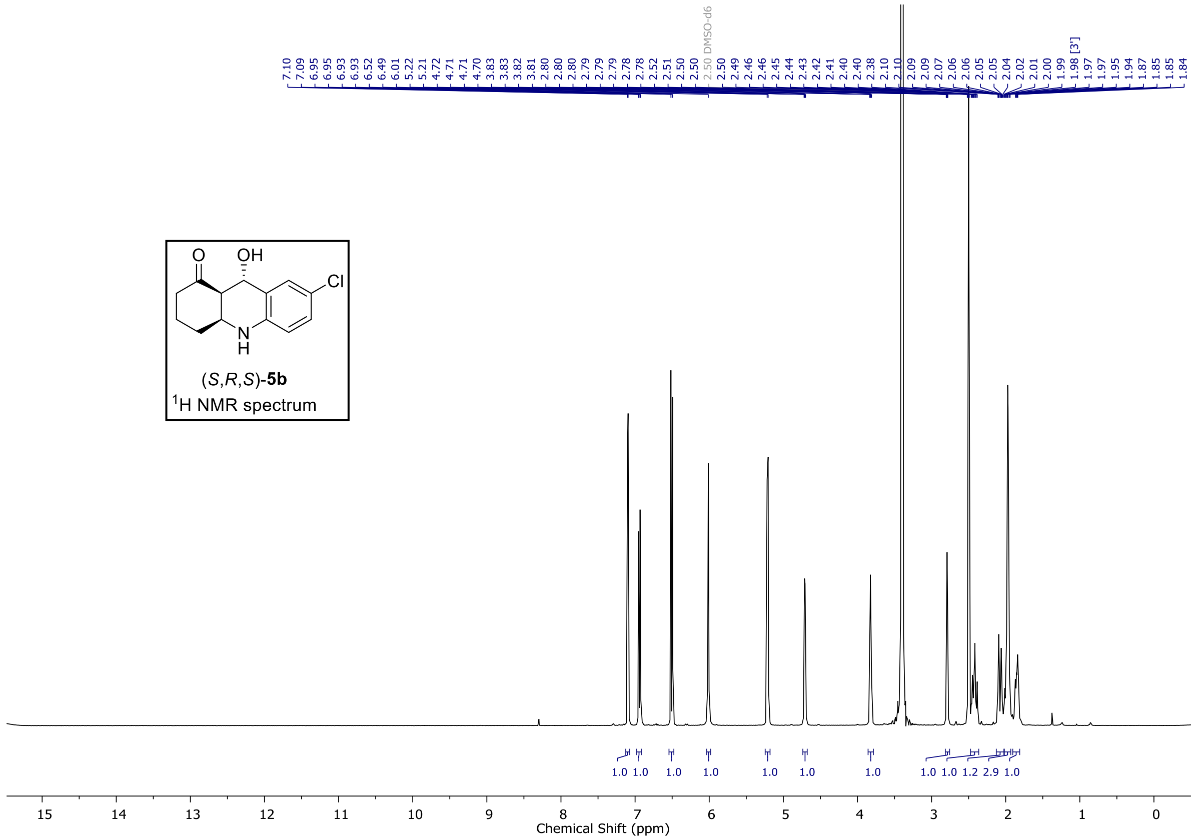
2.50 DMSO-d6
(S,R,S)-5b
1H NMR spectrum
Chemical Shift (ppm)

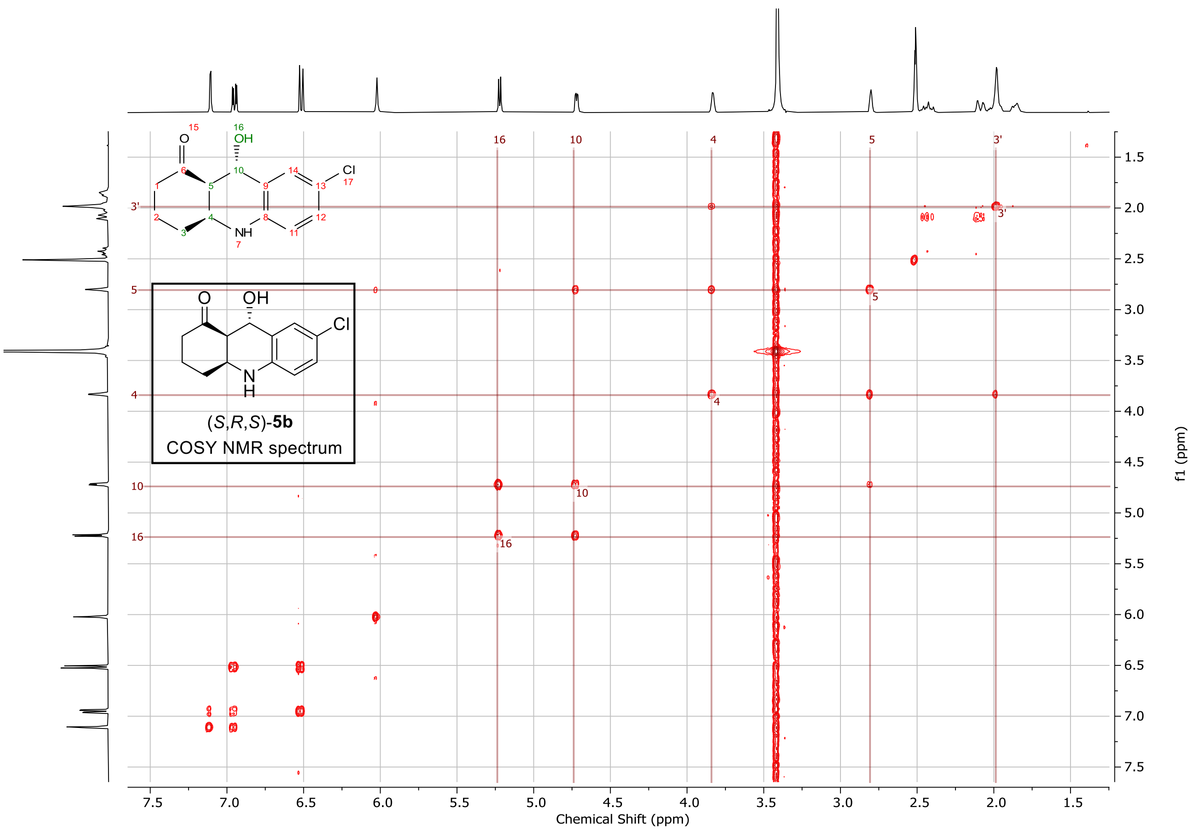
(S,R,S)-5b
COSY NMR spectrum
Chemical Shift (ppm)
f1 (ppm)

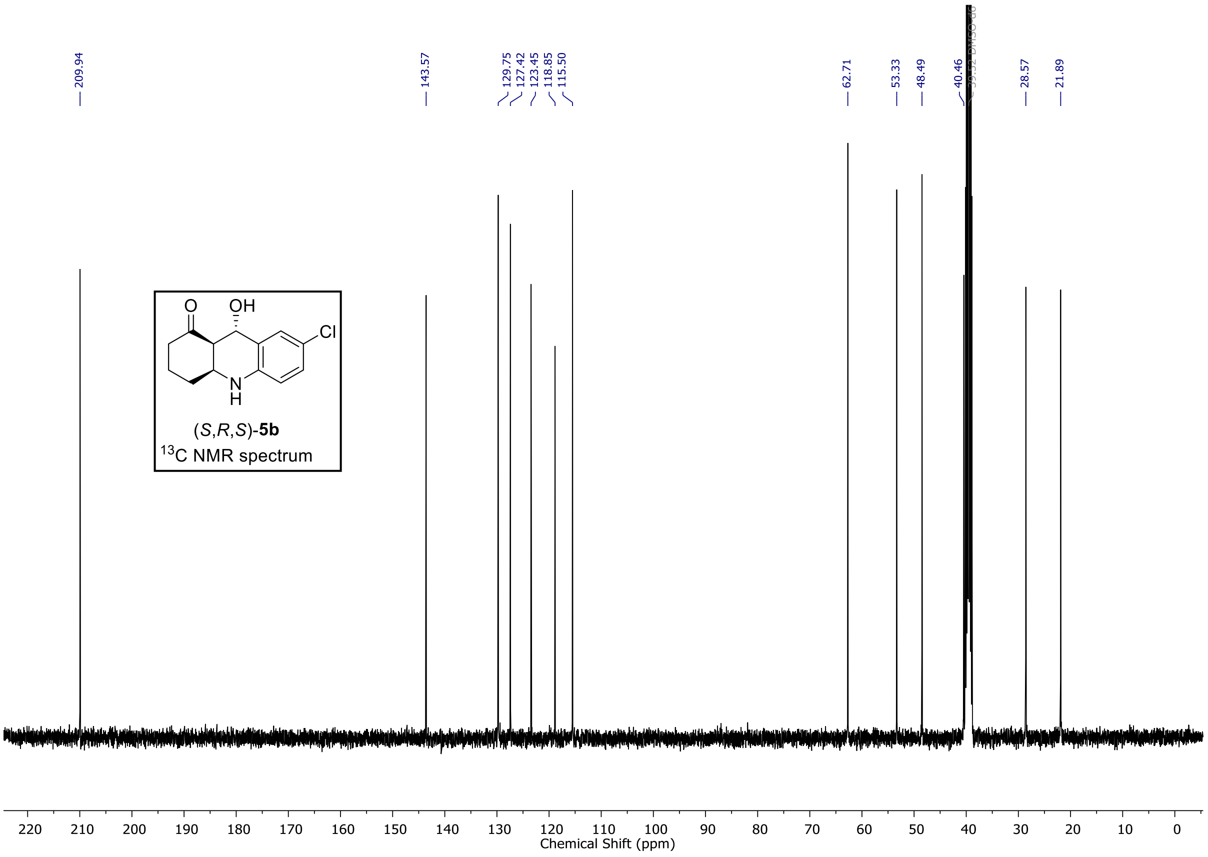
209.94
143.57
129.75
127.42
123.45
118.85
115.50
62.71
53.33
48.49
40.46
28.57
21.89
(S,R,S)-5b
13C NMR spectrum
Chemical Shift (ppm)

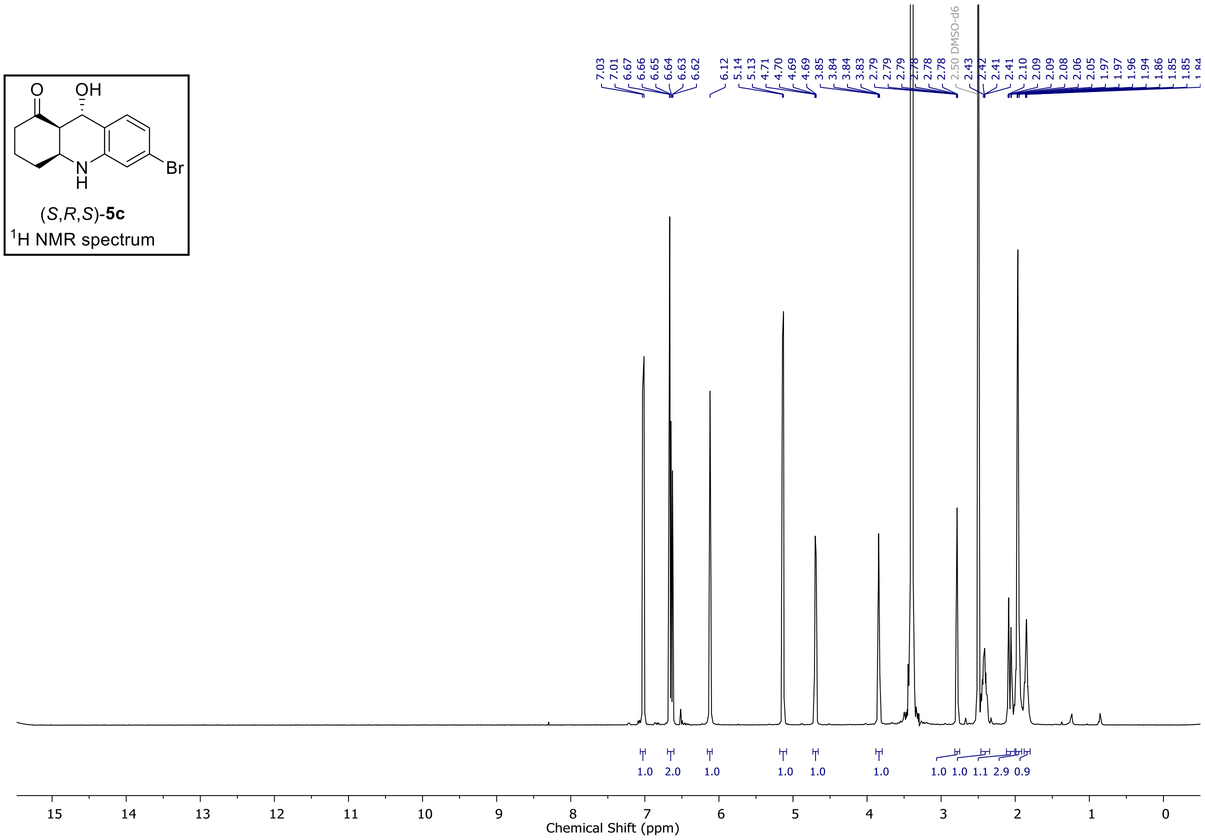
(S,R,S)-5c
1H NMR spectrum
2.50 DMSO-d6
Chemical Shift (ppm)

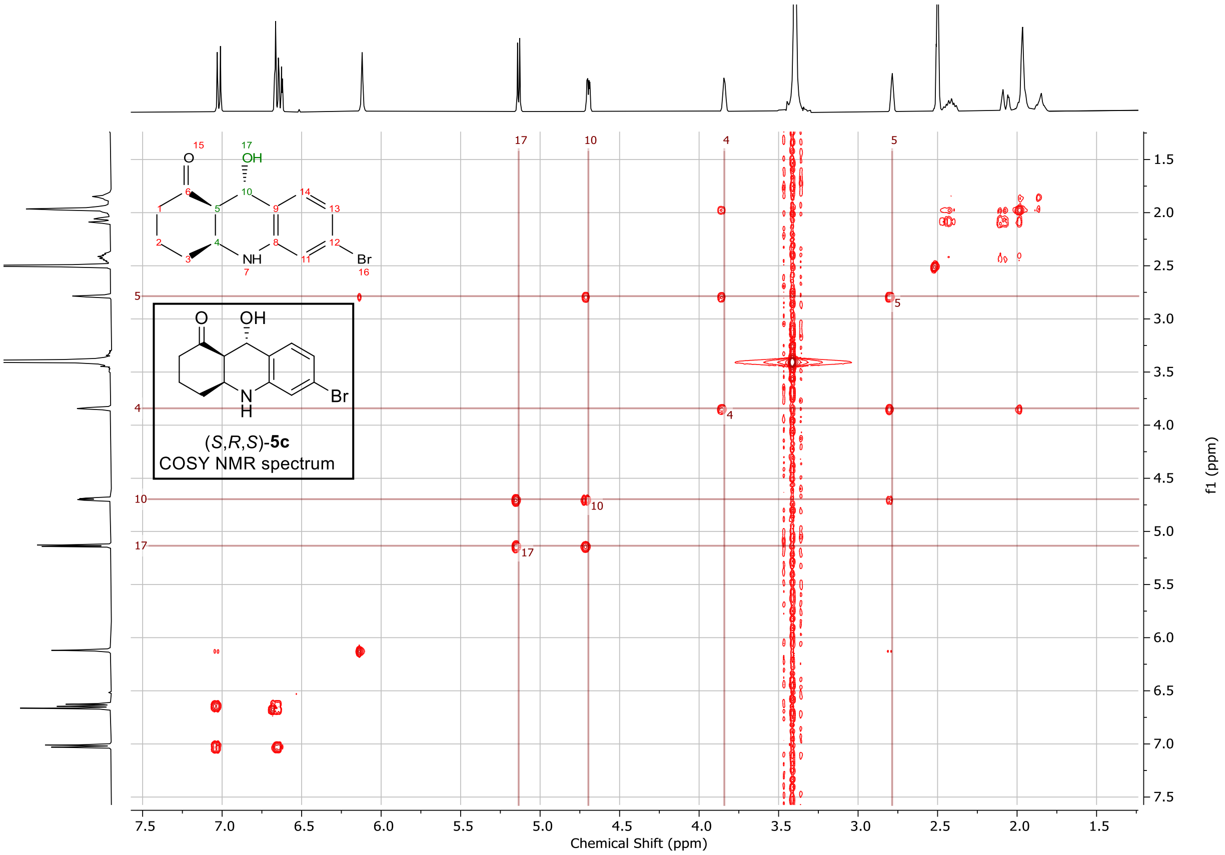
(S,R,S)-5c
COSY NMR spectrum
Chemical Shift (ppm)
f1 (ppm)

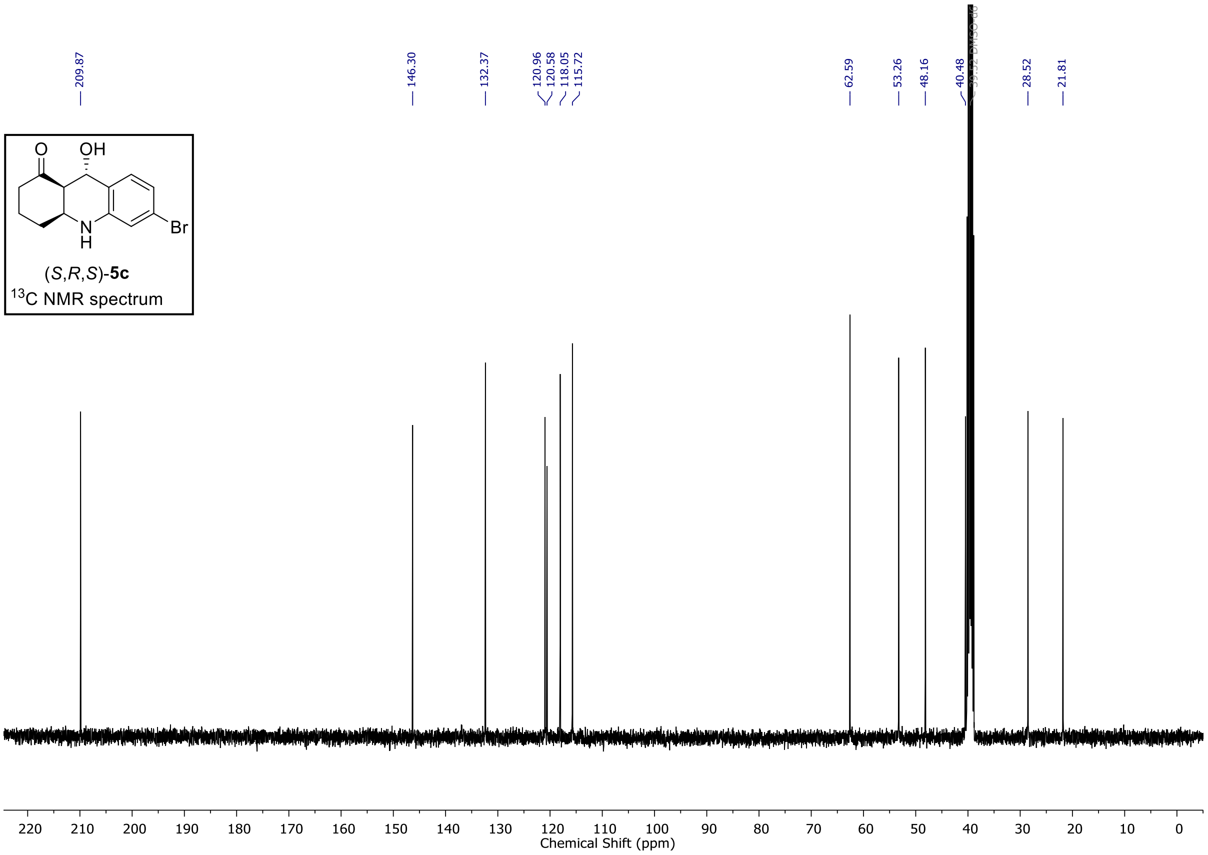
209.87
146.30
132.37
120.96
120.58
118.05
115.72
62.59
53.26
48.16
40.48
28.52
21.81
O
OH
N
H
Br
(*S*,*R*,*S*)-**5c**
^{13}C NMR spectrum
Chemical Shift (ppm)

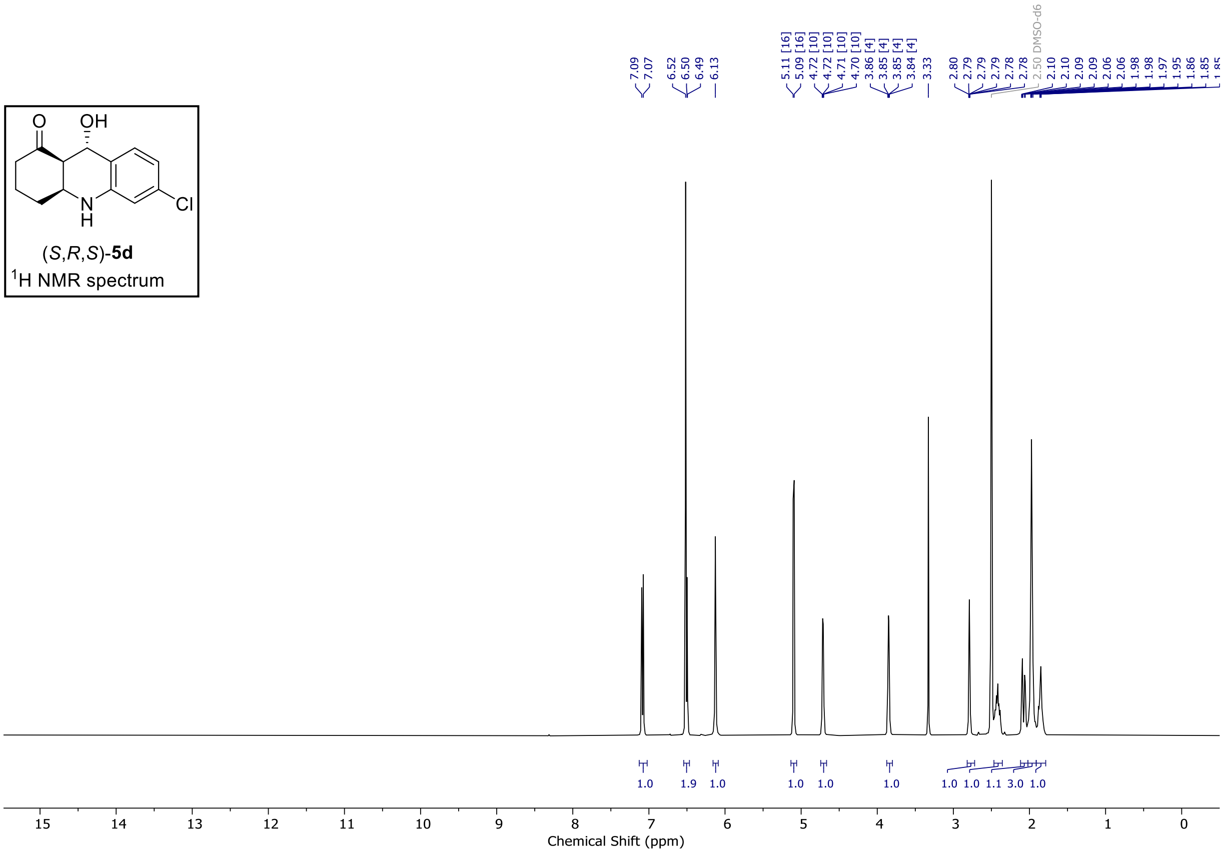
7.09
7.07
6.52
6.50
6.49
6.13
5.11 [16]
5.09 [16]
4.72 [10]
4.72 [10]
4.71 [10]
4.70 [10]
3.86 [4]
3.85 [4]
3.85 [4]
3.84 [4]
3.33
2.80
2.79
2.79
2.79
2.78
2.50 DMSO-d6
2.10
2.10
2.09
2.09
2.06
2.06
1.98
1.98
1.97
1.95
1.86
1.85
O
OH
N
H
Cl
(*S*,*R*,*S*)-**5d**
^{1}H NMR spectrum
1.0 1.9 1.0 1.0 1.0 1.0 1.0 1.0 1.1 3.0 1.0
Chemical Shift (ppm)

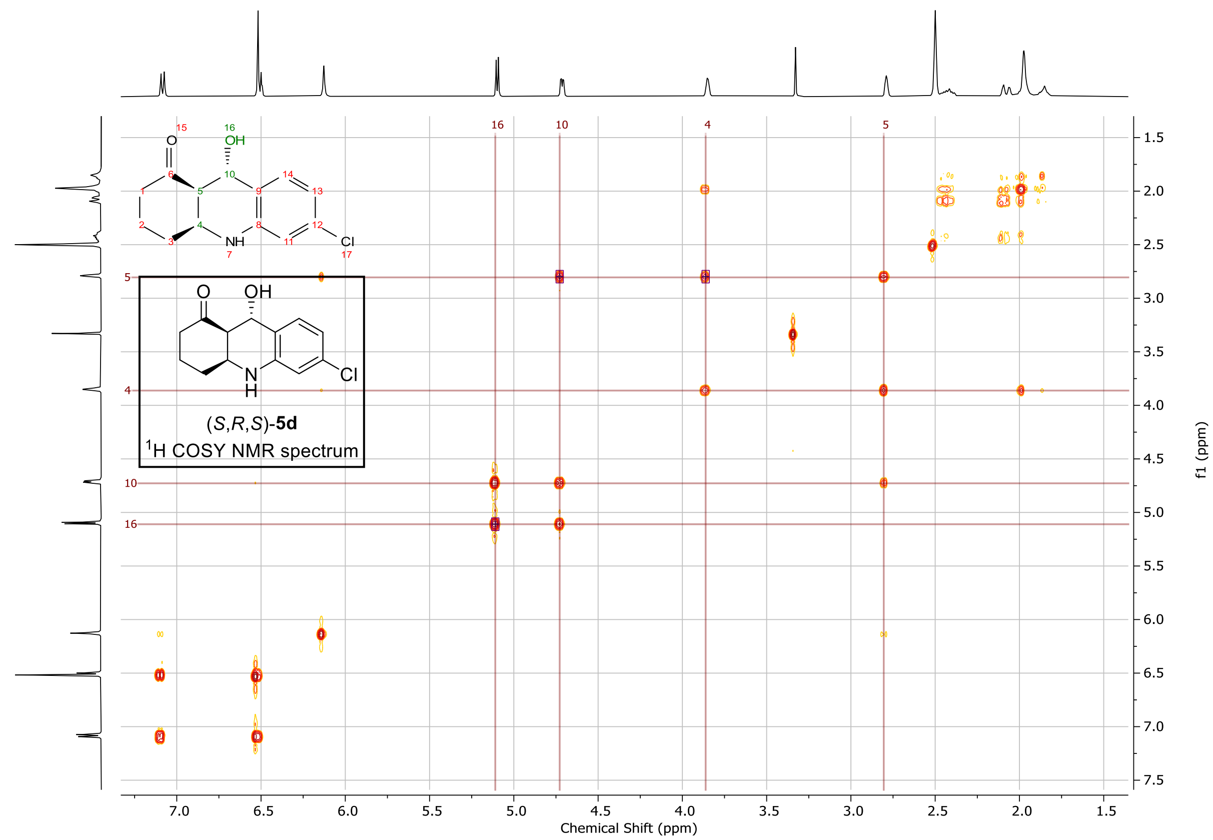
(S,R,S)-5d
^{1}H COSY NMR spectrum
Chemical Shift (ppm)
f1 (ppm)

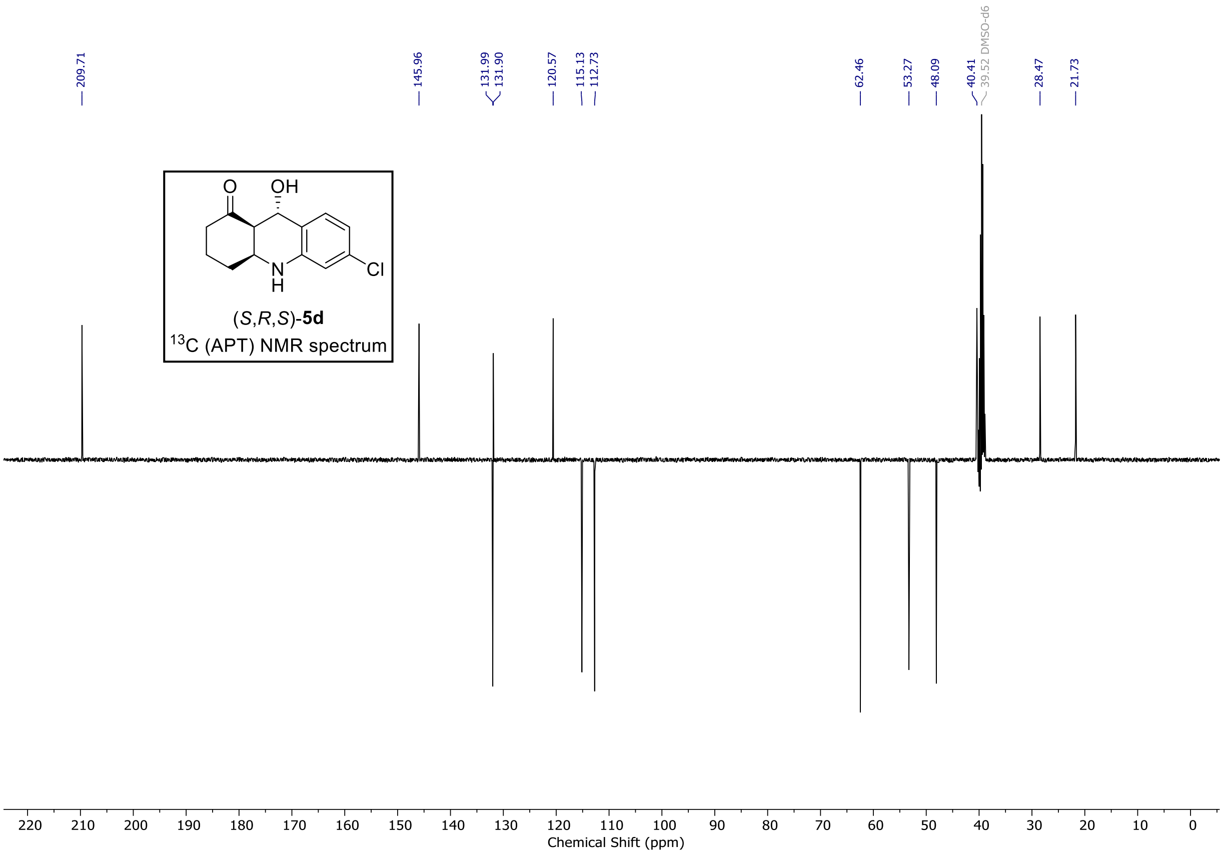
209.71
145.96
131.99
131.90
120.57
115.13
112.73
62.46
53.27
48.09
40.41
39.52 DMSO-d6
28.47
21.73
(S,R,S)-5d
^{13}C (APT) NMR spectrum
Chemical Shift (ppm)

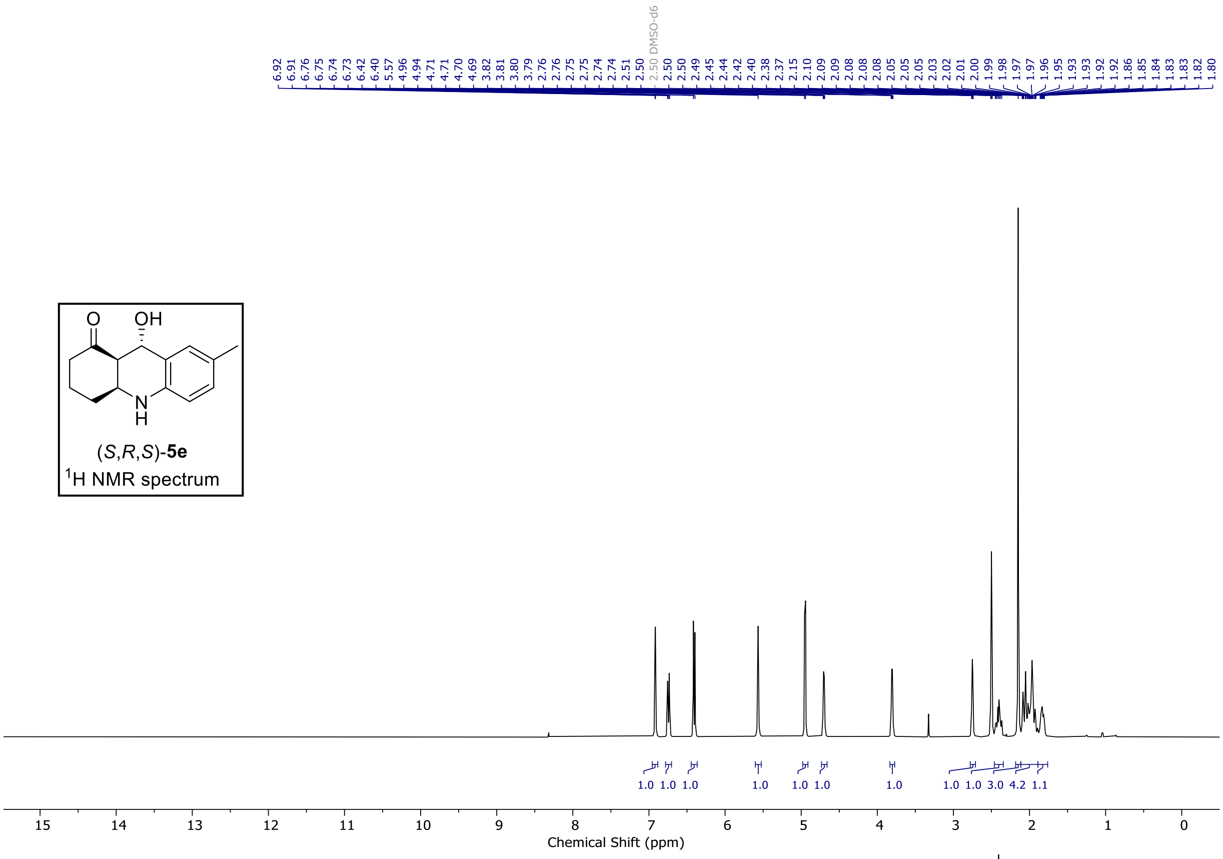
6.92 6.91 6.76 6.75 6.74 6.73 6.42 6.40 5.57 4.96 4.94 4.71 4.71 4.70 4.69 3.82 3.81 3.80 3.79 2.76 2.76 2.75 2.75 2.74 2.74 2.51 2.50 2.50 2.50 2.49 2.45 2.44 2.42 2.40 2.38 2.37 2.15 2.10 2.09 2.08 2.08 2.08 2.05 2.05 2.05 2.03 2.02 2.01 2.00 1.99 1.98 1.97 1.97 1.96 1.95 1.93 1.93 1.92 1.92 1.86 1.85 1.84 1.83 1.83 1.82 1.80
2.50 DMSO-d6
O
OH
N
H
(S,R,S)-5e
1H NMR spectrum
1.0 1.0 1.0 1.0 1.0 1.0 1.0 1.0 1.0 3.0 4.2 1.1
Chemical Shift (ppm)

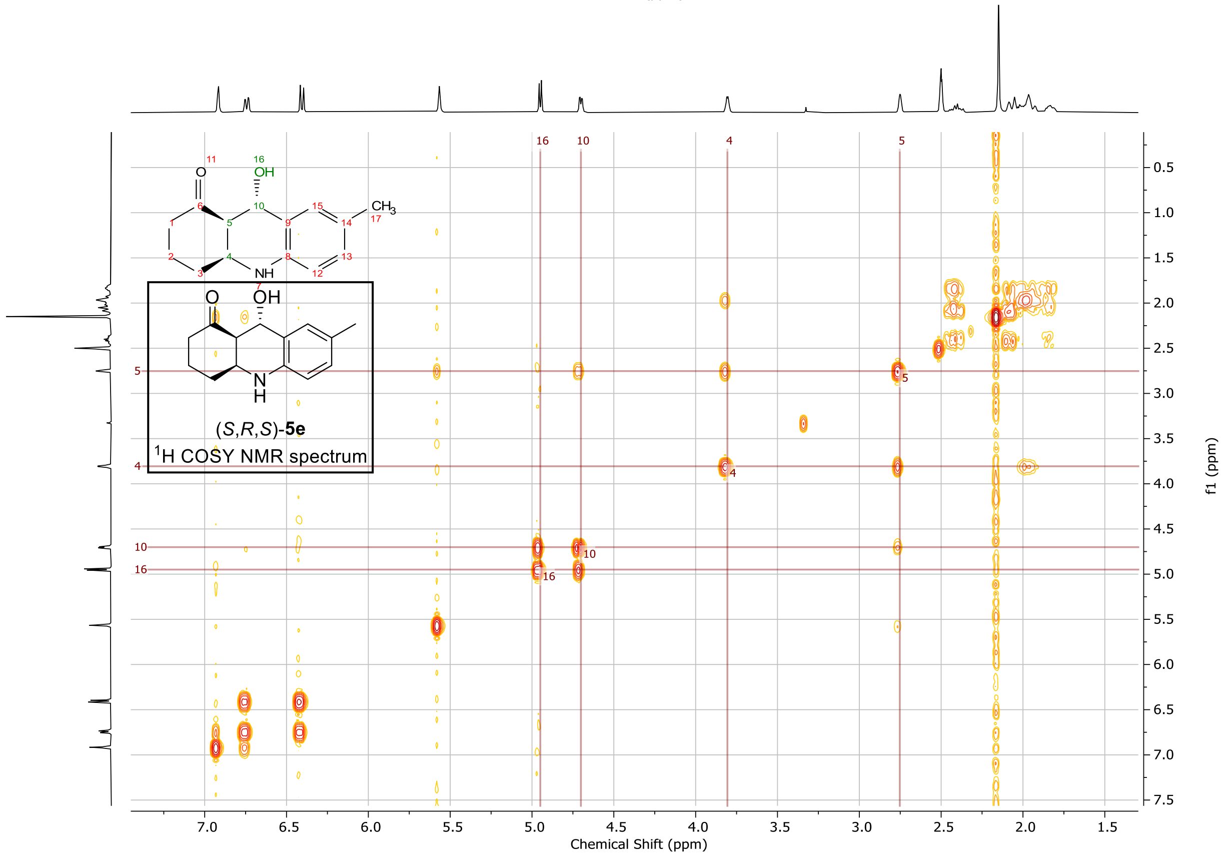
(S,R,S)-5e
1H COSY NMR spectrum
Chemical Shift (ppm)
f1 (ppm)

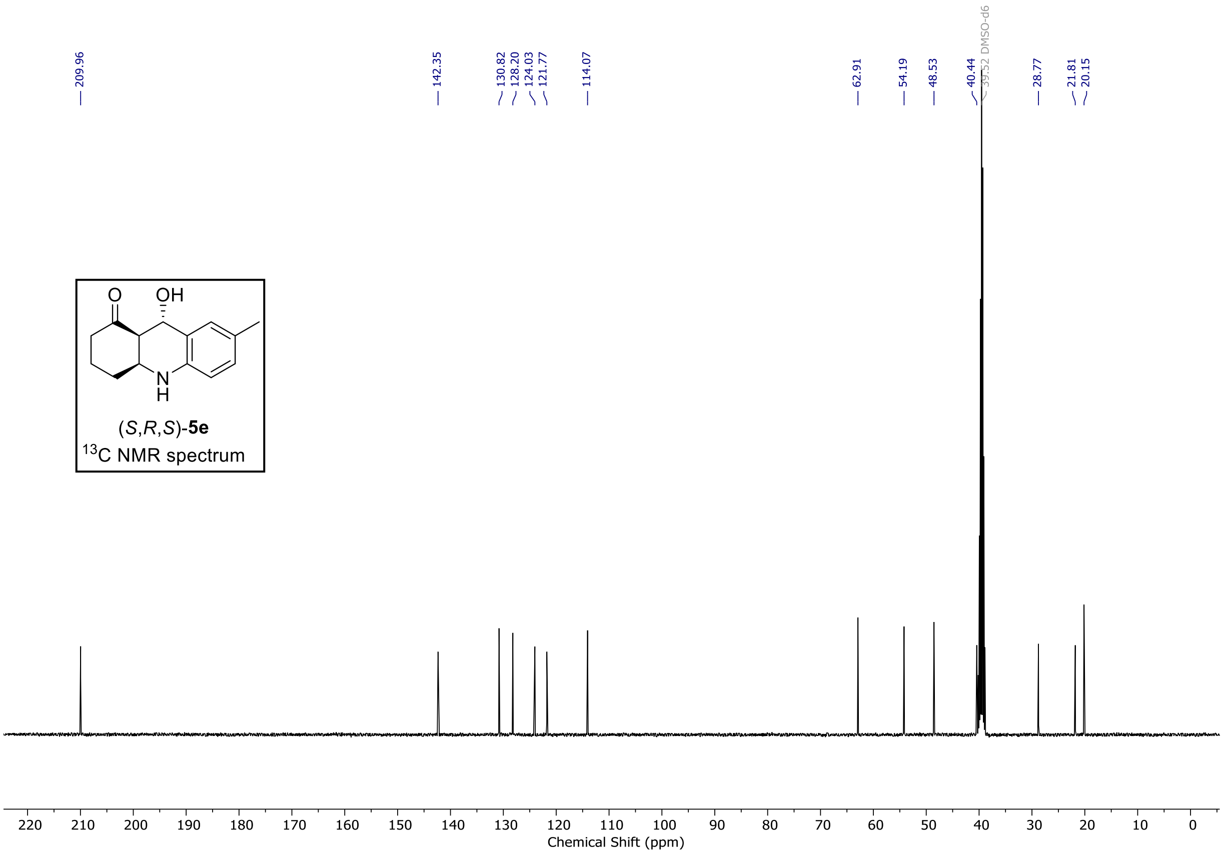

209.96
142.35
130.82
128.20
124.03
121.77
114.07
62.91
54.19
48.53
40.44
39.52 DMSO-d6
28.77
21.81
20.15
O
OH
N
H
(S,R,S)-5e
13C NMR spectrum
220
210
200
190
180
170
160
150
140
130
120
110
100
90
80
70
60
50
40
30
20
10
0
Chemical Shift (ppm)

21. NMR spectra of asymmetric Michael-induced Ring-Closure/aldol reaction sequence

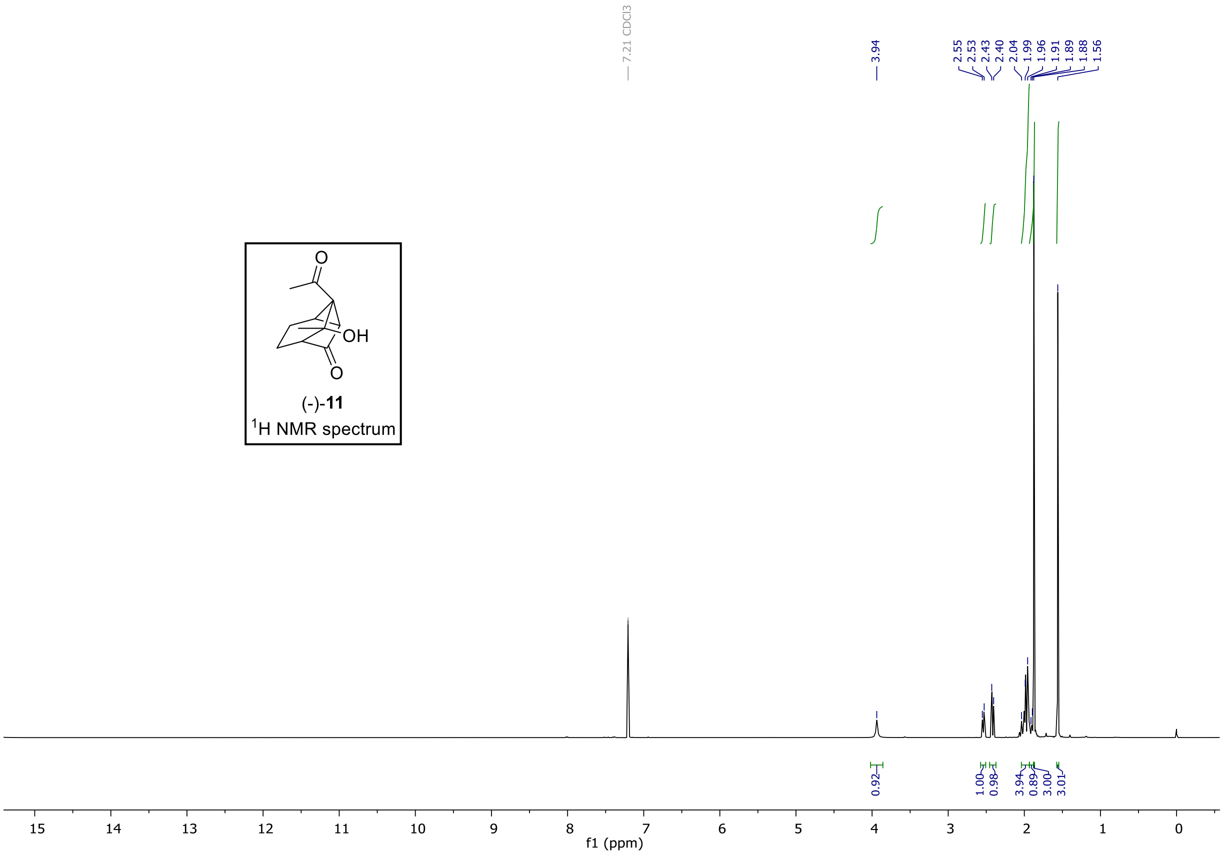

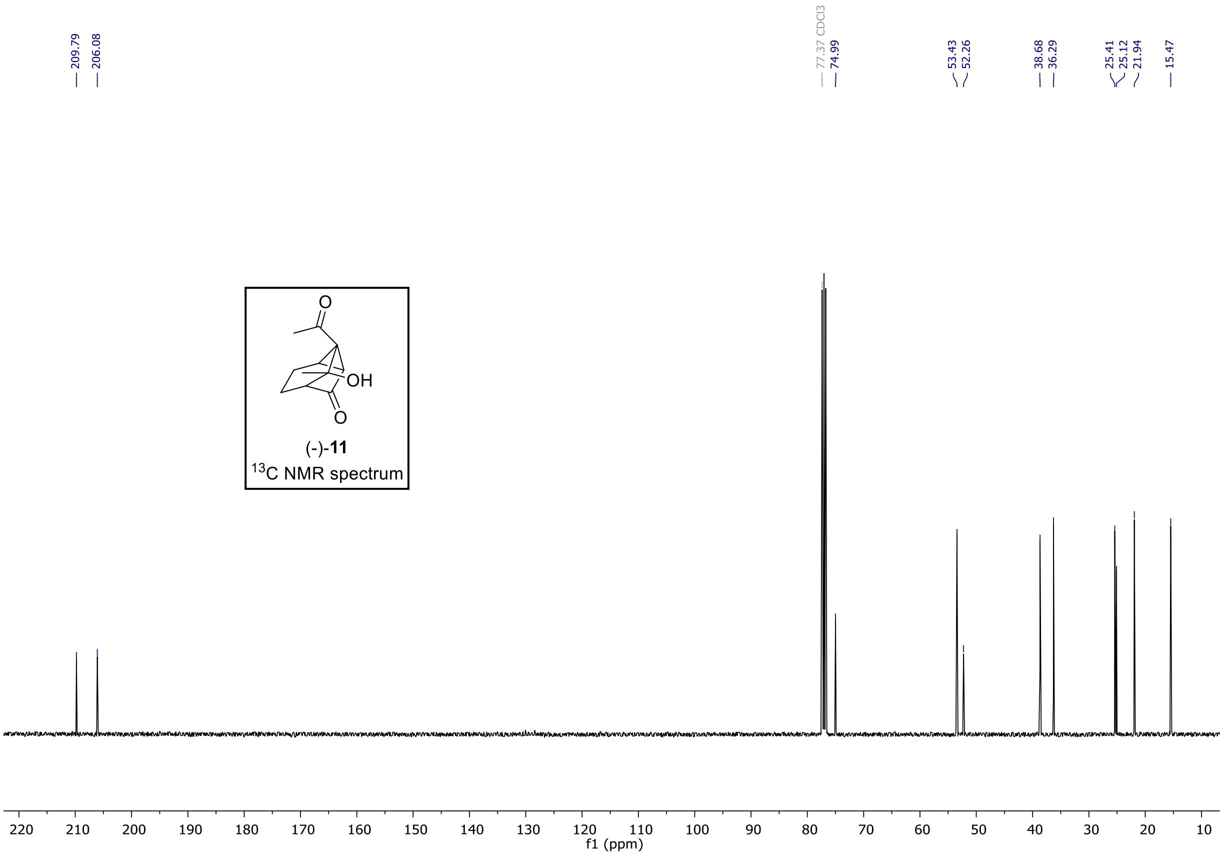

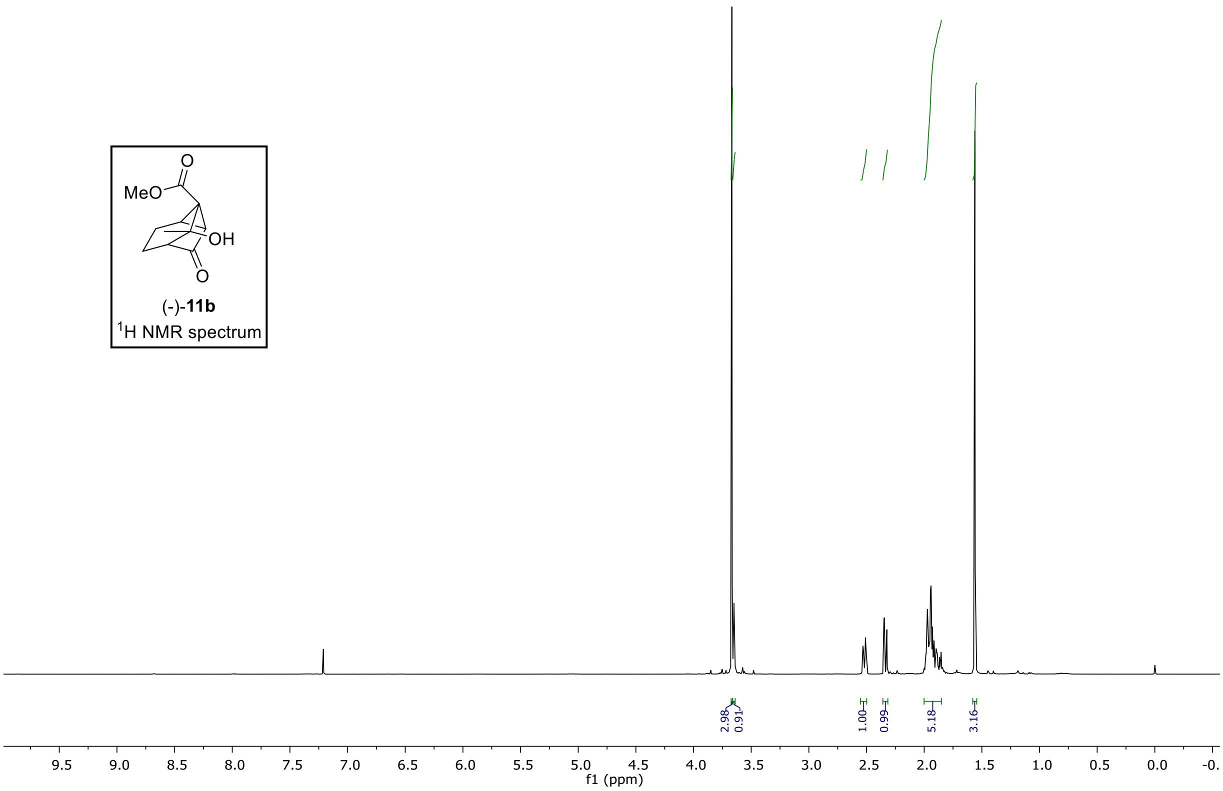
MeO
O
OH
O
(-)-11b
1H NMR spectrum
2.98
0.91
1.00
0.99
5.18
3.16
9.5
9.0
8.5
8.0
7.5
7.0
6.5
6.0
5.5
5.0
4.5
4.0
3.5
3.0
2.5
2.0
1.5
1.0
0.5
0.0
-0.
f1 (ppm)

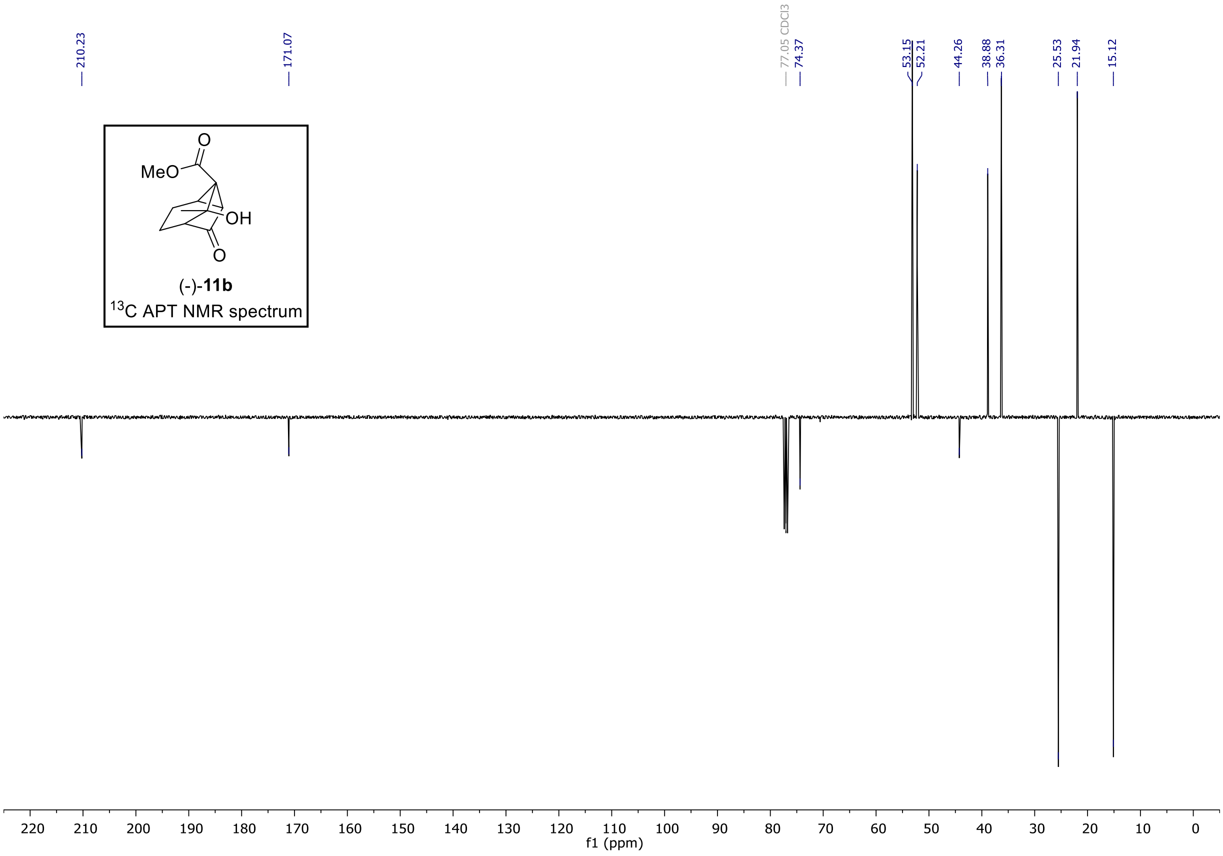
210.23
171.07
77.05 CDCl3
74.37
53.15
52.21
44.26
38.88
36.31
25.53
21.94
15.12
MeO
O
OH
O
(-)-11b
13C APT NMR spectrum
220
210
200
190
180
170
160
150
140
130
120
110
100
90
80
70
60
50
40
30
20
10
0
f1 (ppm)

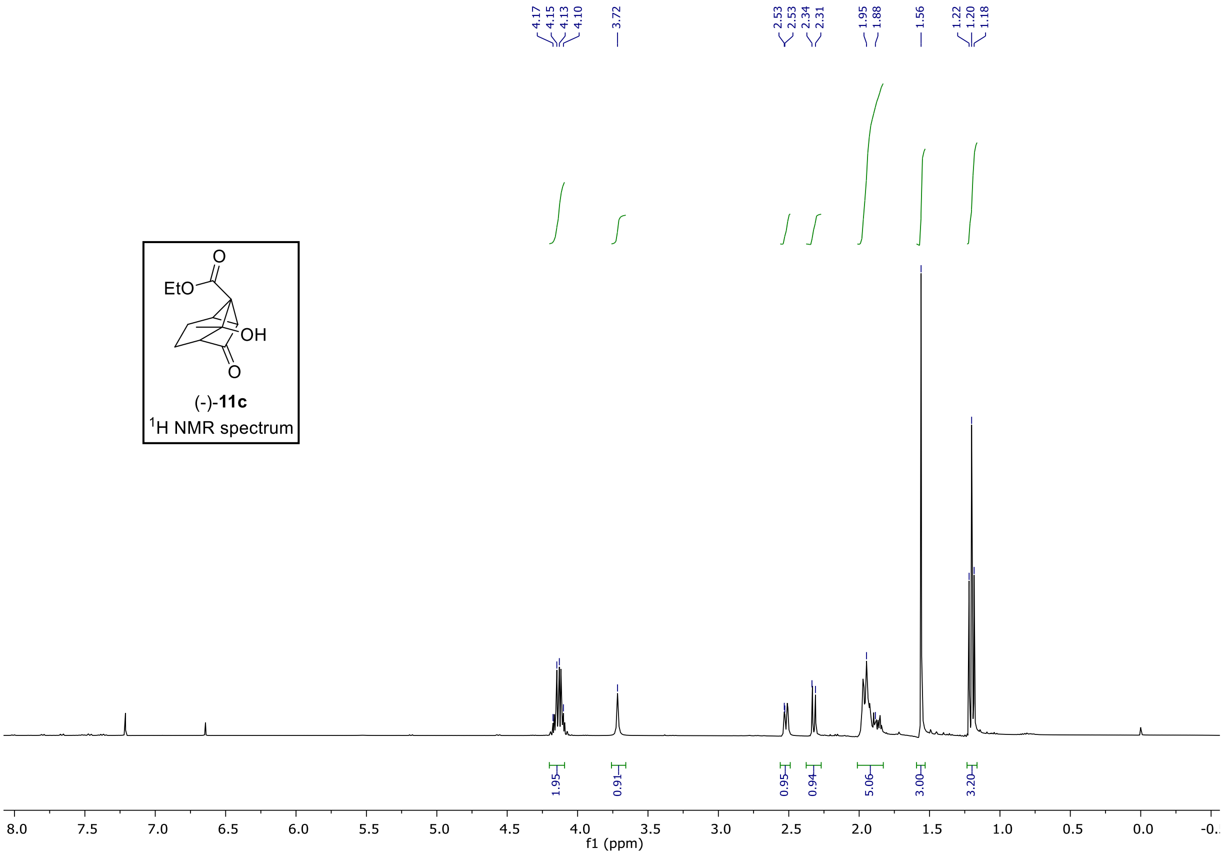
4.17
4.15
4.13
4.10
3.72
2.53
2.53
2.34
2.31
1.95
1.88
1.56
1.22
1.20
1.18
EtO
O
OH
O
(-)-11c
1H NMR spectrum
1.95
0.91
0.95
0.94
5.06
3.00
3.20
8.0
7.5
7.0
6.5
6.0
5.5
5.0
4.5
4.0
3.5
3.0
2.5
2.0
1.5
1.0
0.5
0.0
f1 (ppm)

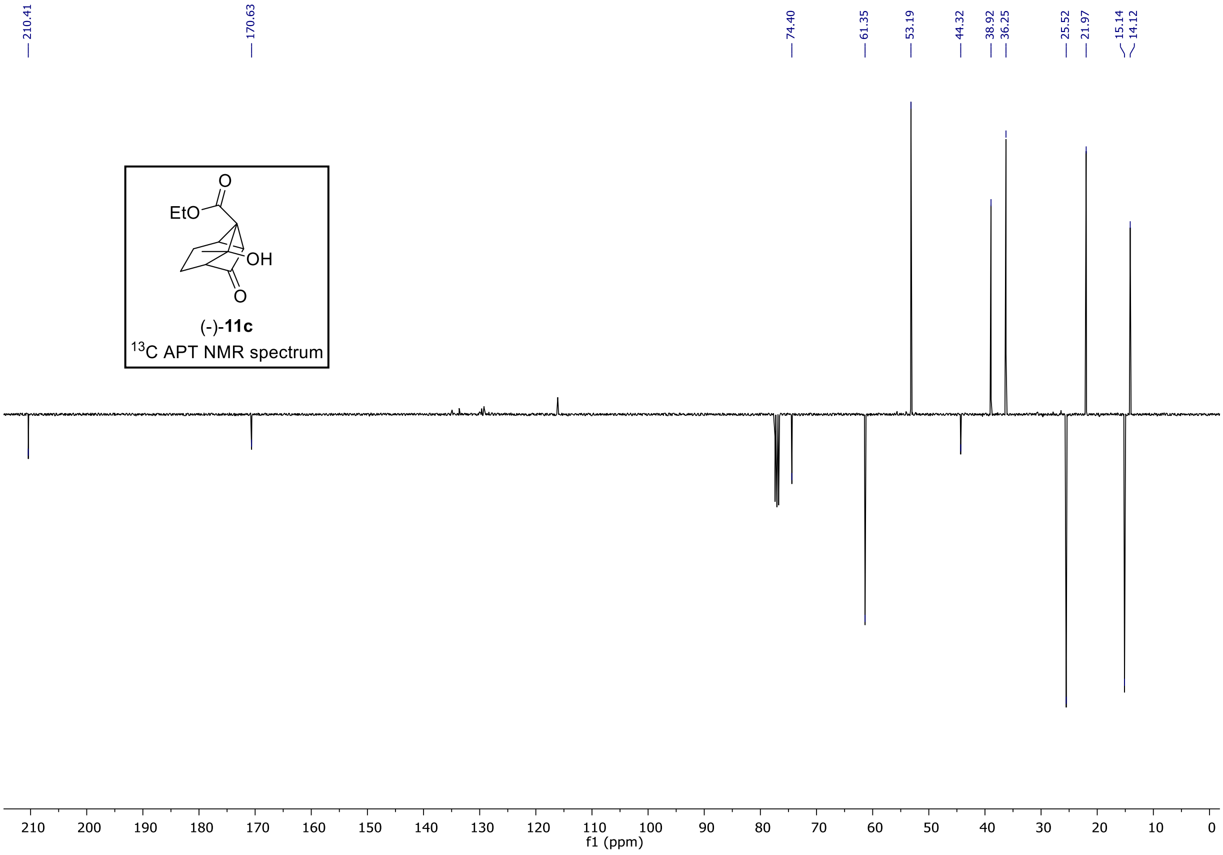
210.41
170.63
74.40
61.35
53.19
44.32
38.92
36.25
25.52
21.97
15.14
14.12
EtO
O
OH
O
(-)-11c
13C APT NMR spectrum
210
200
190
180
170
160
150
140
130
120
110
100
90
80
70
60
50
40
30
20
10
0
f1 (ppm)

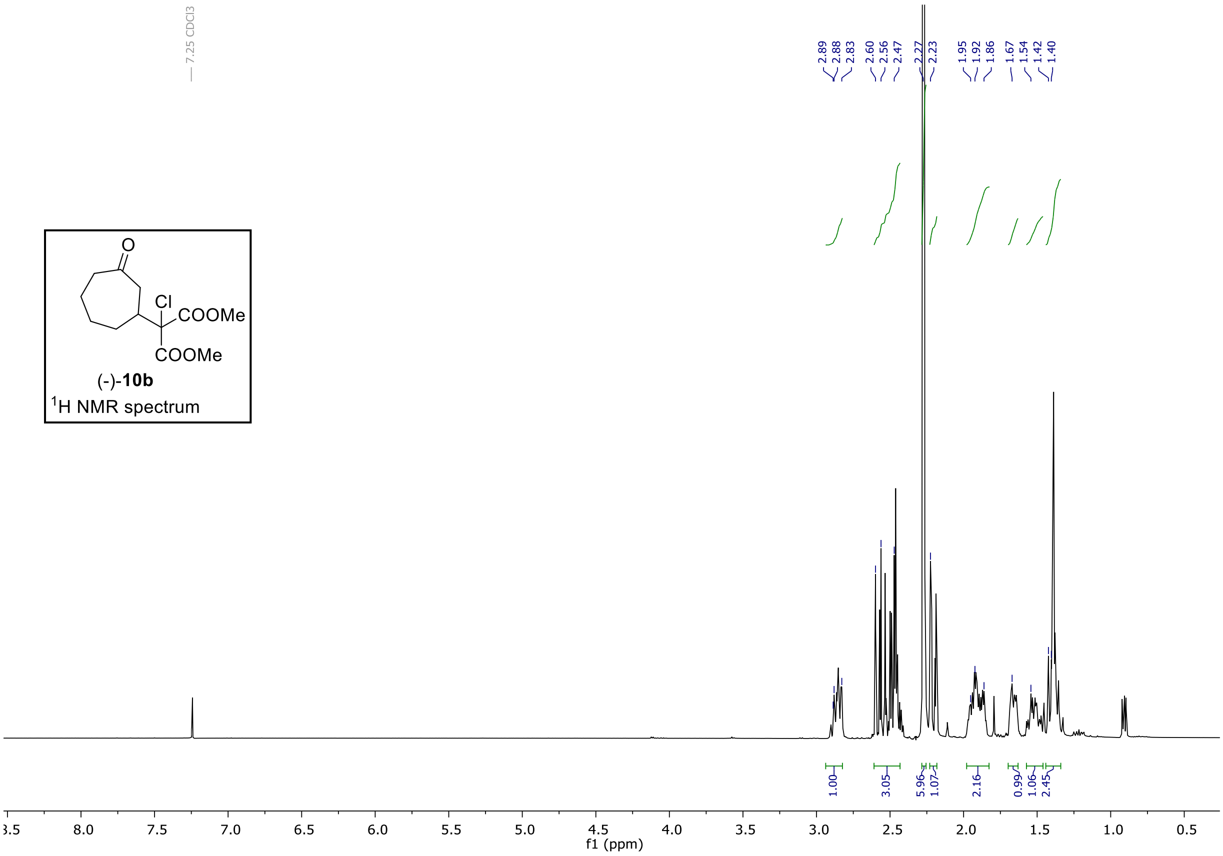

7.25 CDCl3
2.89
2.88
2.83
2.60
2.56
2.47
2.27
2.23
1.95
1.92
1.86
1.67
1.54
1.42
1.40
Cl
COOMe
COOMe
O
(-)-10b
1H NMR spectrum
1.00
3.05
5.96
1.07
2.16
0.99
1.06
2.45
f1 (ppm)

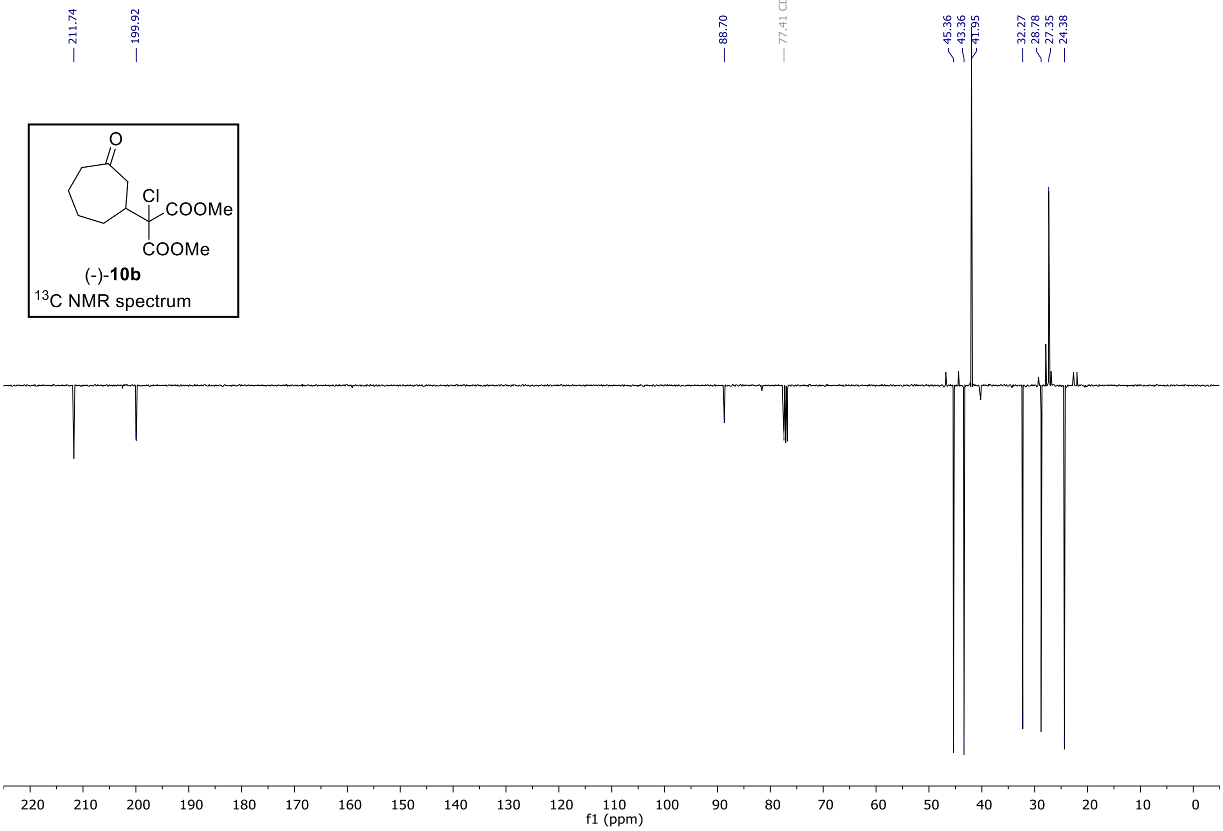

211.74
199.92
88.70
77.41 CDCl3
45.36
43.36
41.95
32.27
28.78
27.35
24.38
O
Cl
COOMe
COOMe
(-)-10b
13C NMR spectrum
f1 (ppm)

22. Extended 1D/2D-NMR experiments supporting the structure of compound 11

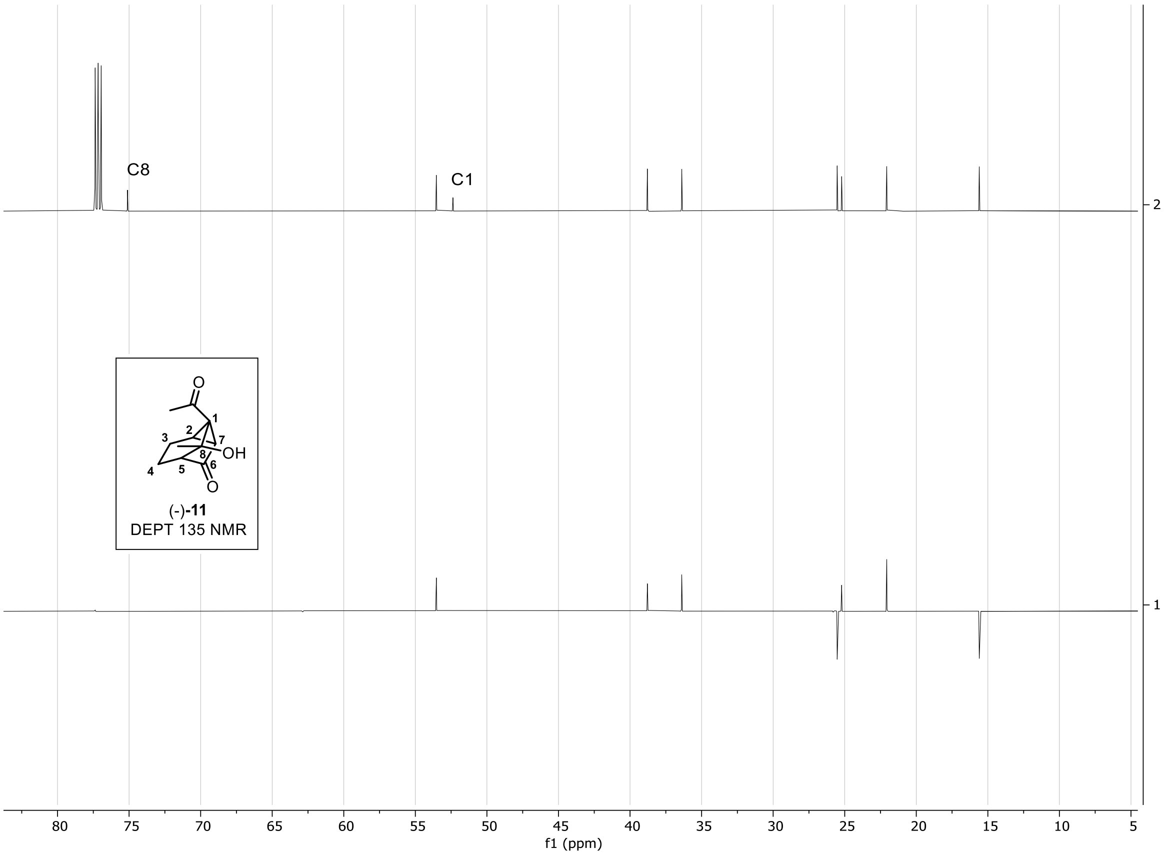

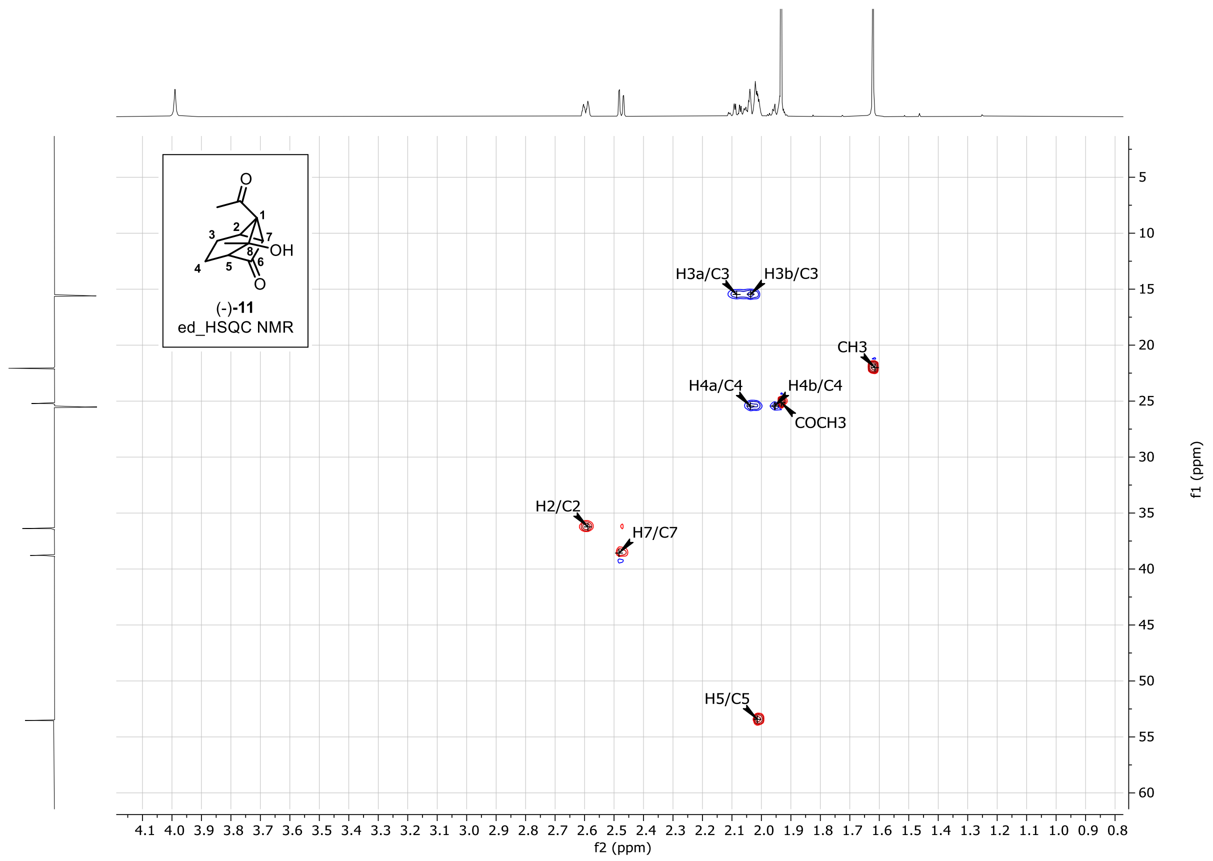

Comparison between the standard C-spectrum and a DEPT-135 experiment identified two CH_2 groups (C2, C4) and besides two carbonyl groups 2 further quaternary signals in the aliphatic region at 75.11 (C8) and 53.37 (C1) ppm. The signal at 75.11 ppm clearly suggesting the vicinity to a strongly withdrawing group/atom like the hydroxyl group. Along with the phase sensitive HSQC spectrum, two CH_3 groups with significantly different chemical environment were identified, one bearing with the typical shifts for an acetyl groups, further assigned as $COCH_3$ and CH_3. Three CH-groups were identified as unambiguous $^1H/^{13}C$ shift dupels, corresponding to the 2, 5 and 7-position. This analysis already pointed to an additional bond formation in line with some sort of aldol-type chemistry, additionally to the targeted cyclopropanation transformation. The HSQC also identified the broad singlet at 3.99 ppm as a proton attached to a hetero-atom; considering the starting chemical structure of the starting material most likely an OH group. Measurement of a ^1H NMR in DMSO-d_6 confirmed the isolated singlet.

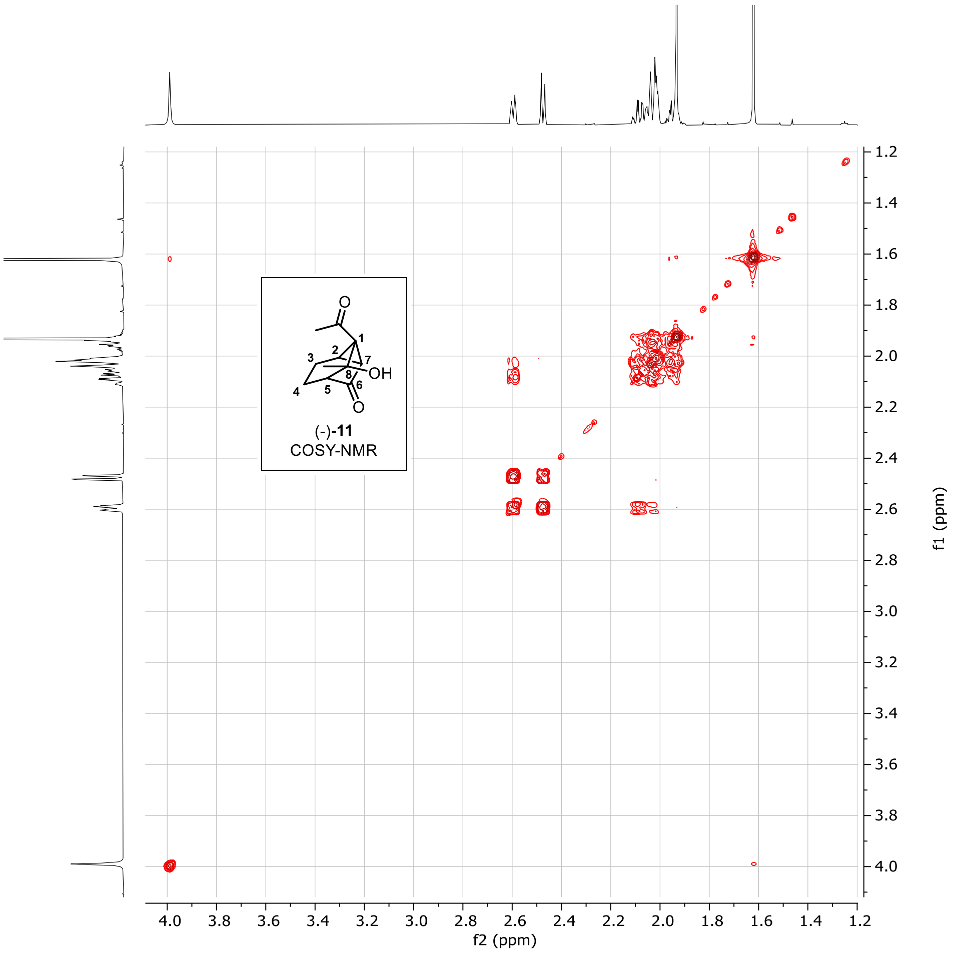

H,H-COSY-correlation

The H,H-COSY spectrum confirmed the two CH_2 groups identified in the HSQC and pointed towards the H7-H2-H3a,b spin system and in line with correlation to the H4a/H4b system, which however is in the crowded region of the spectrum (1.9 - 2.15 ppm). Consequently, also the putative correlation between H5 and H4a/H4b could not be resolved due to complete overlap of the signals as is also visible in the

HSQC spectrum. Further, a weak correlation of the OH group with the isolated CH_3 group is also observed, consistent with structure and assignment.

HMBC-Correlation

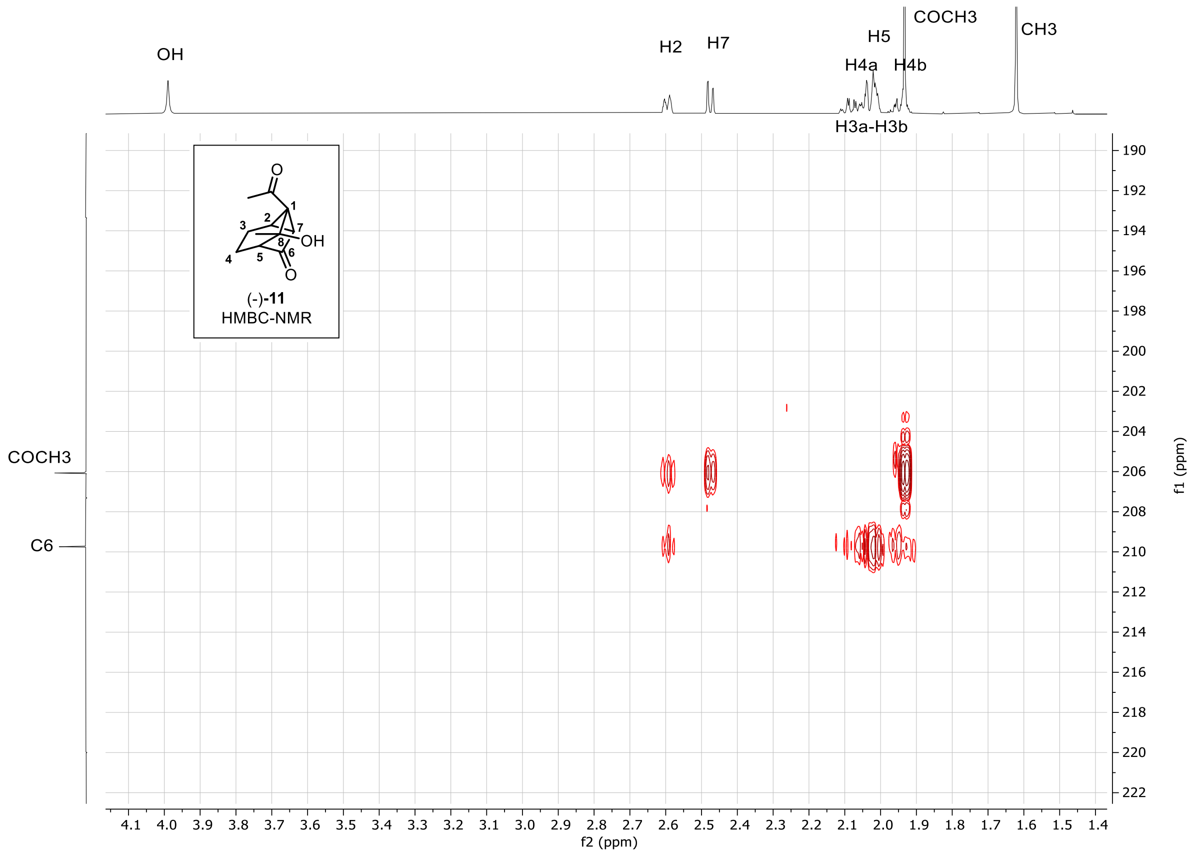

Due to the similar ^{1}H-shifts of many signals the HMBC-correlation is complex but nonetheless allows confirmation of the suggested structure. Only selected correlations are pointed out here. It clearly allowed the assignment of the two carbonyl groups to the remaining acetyl group (206 ppm) based on correlation to the $COCH_3$ signal and the other carbonyl group in the ring-system (210 ppm). All observed correlations over two and particularly three bonds (C6-H2, H4, H5 and **C**OCH_3 to H2, H7) from these carbonyls are consistent with the structure. Further, in the HMBC the vicinity of the hydroxyl-group and the isolated methyl group to both quaternary carbons (C1, C8) is clearly established as well as the additional correlation of the methyl group to the C5-position. Importantly, there are also no correlations conflicting with the suggested structure, which was later also confirmed via the crystal structure.

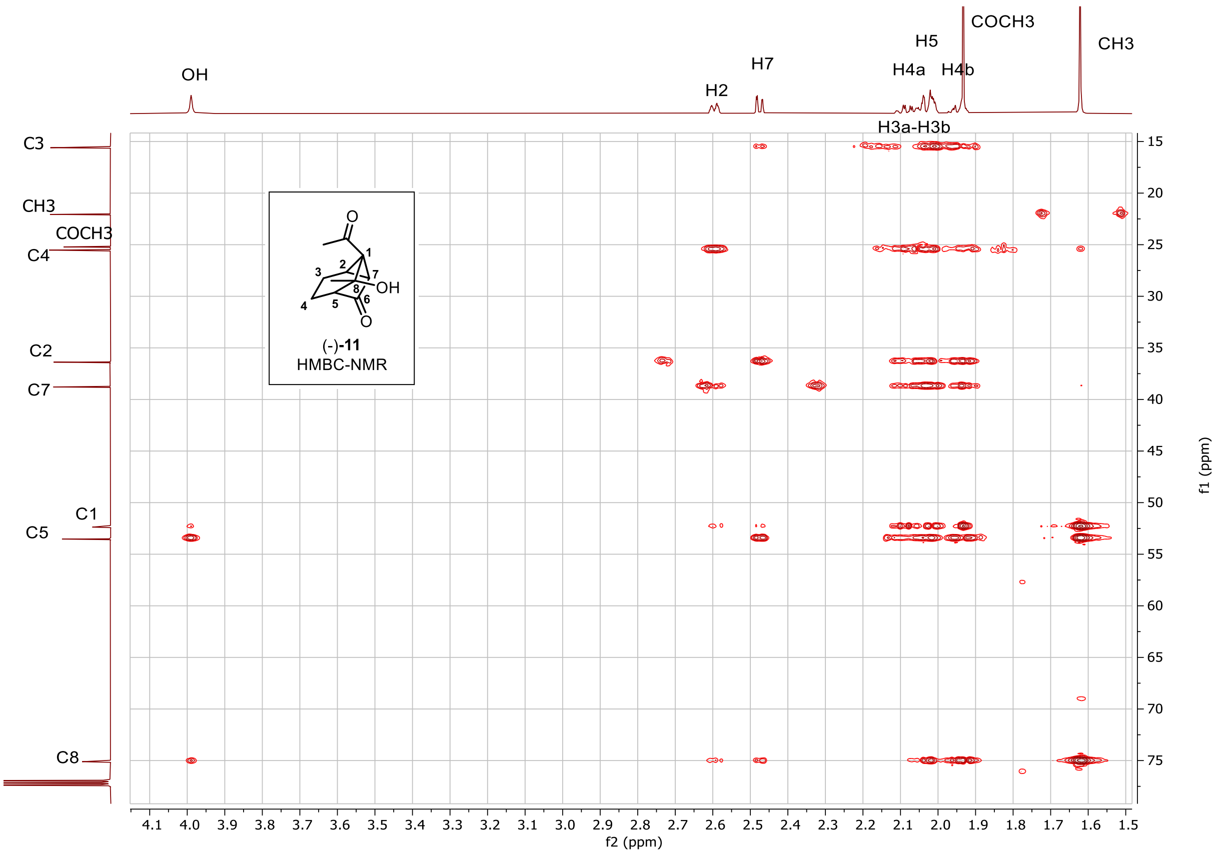

In respect to the relative stereochemistry at C8, both the NOESY (not shown) and ROESY-correlation exhibit a clear correlation between the methyl group at 1.62 ppm (but not the OH group) and all CH and CH_2 groups of the ring system, indicating that the methyl groups must be in the *endo*-position in respect to the former cyclohexyl ring system which was also later confirmed in the crystal structure.

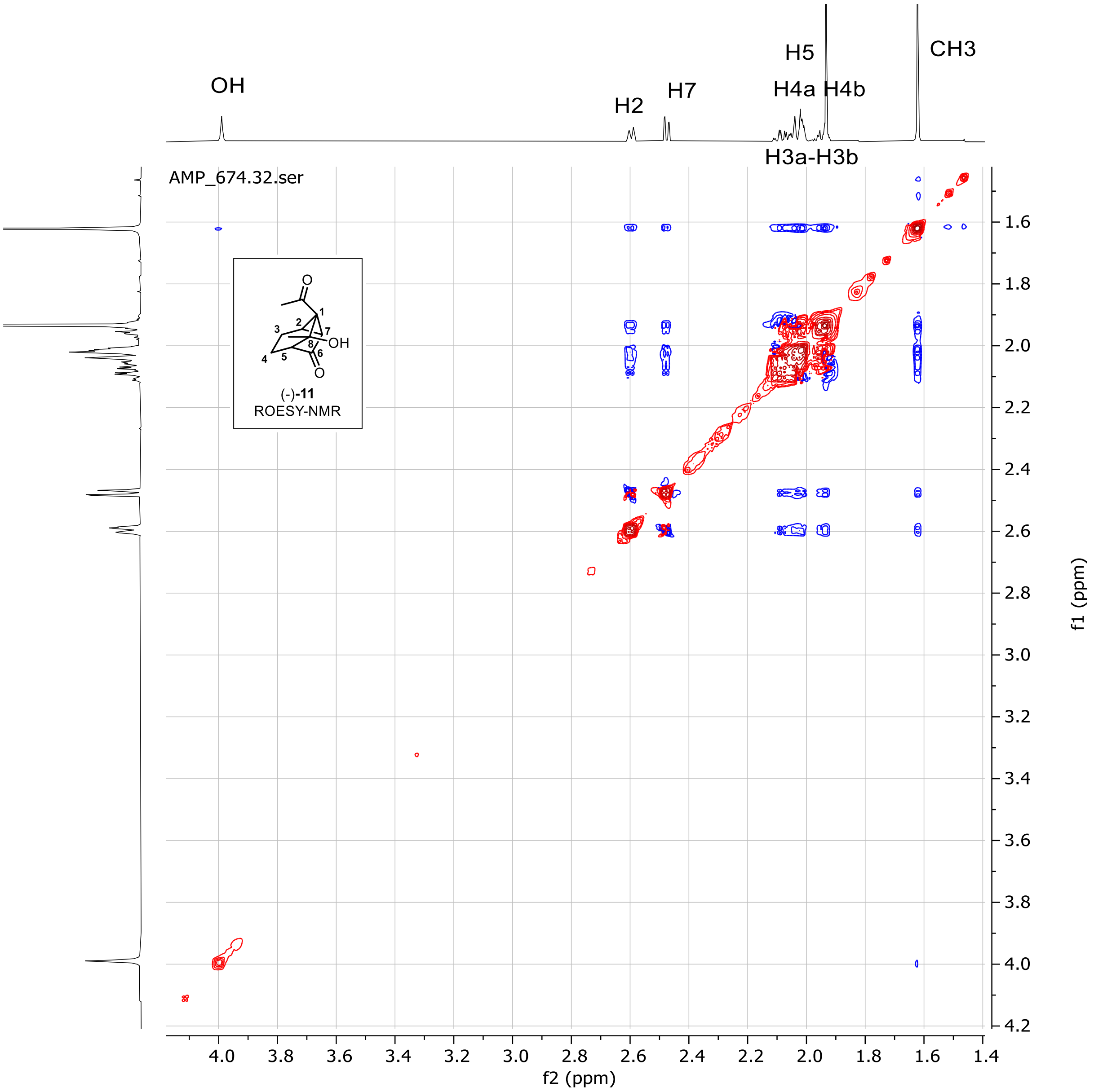
OH
H2
H7
H5
H4a H4b
CH3
H3a-H3b
AMP_674.32.ser
(-)-11
ROESY-NMR
f1 (ppm)
f2 (ppm)

23. NMR Studies for Phosphoric Acids

In order to probe our hypothesis whether the phosphoric acids **PA5**-**PA9** can eventually form stable enantiomers under the reaction conditions, ^{31}P NMR studies of the salts [(*S*)-**AM6**][**PA3**] and [(*S*)-**AM6**][**PA9**] have been carried out at different temperatures.

The ^{31}P NMR spectra of the salts [(*S*)-**AM6**][**PA3**] resulted in complete peak splitting already when being measured at 27 ˚C, indicating the formation of stable diastereomers in chiral environment, therefore suggesting that the acid **PA3** is chiral and it can form stable, distinguishable enantiomers at room temperature as a result of its steric hindrance (Figures S1-S2).

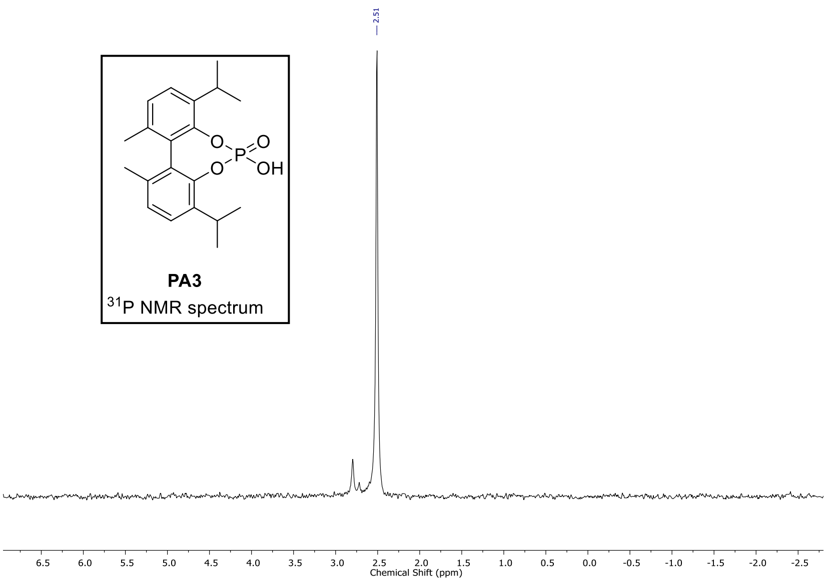

Figure S1. ^{31}P NMR spectrum of the phosphoric acid **PA3** at 27 ˚C.

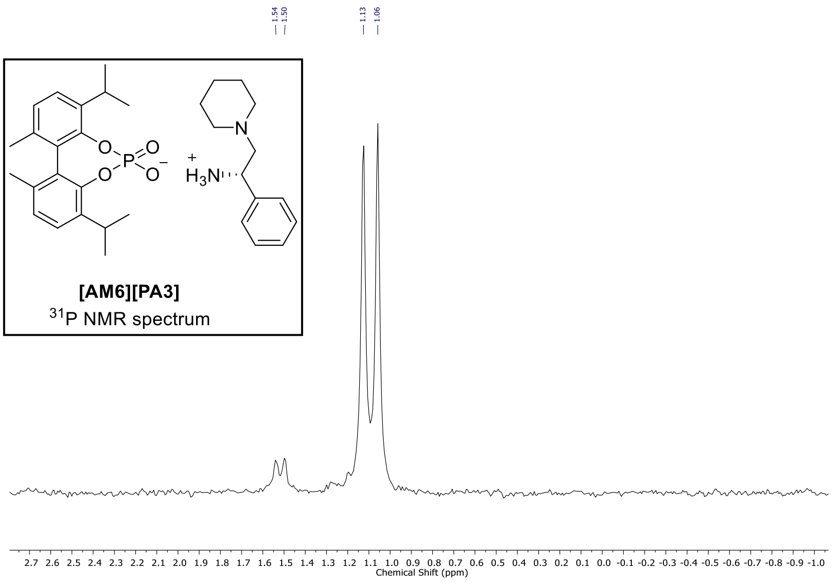

Figure S2. ^{31}P NMR spectrum of the salt [(*S*)-**AM6**][**PA3**] at 27 ˚C.

In contrast, no peak splitting in the NMR spectra of [(*S*)-**AM6**][**PA9**] could be observed at room temperature, suggesting that such ion-paired catalysts based on flexible phosphoric acids might indeed act as a single stereoisomer under the reaction conditions (Figures S3-S4). Measuring the same compounds both at −30 ˚C and − 13 ˚C resulted in no change in this phenomenon (Figures S5-S6).

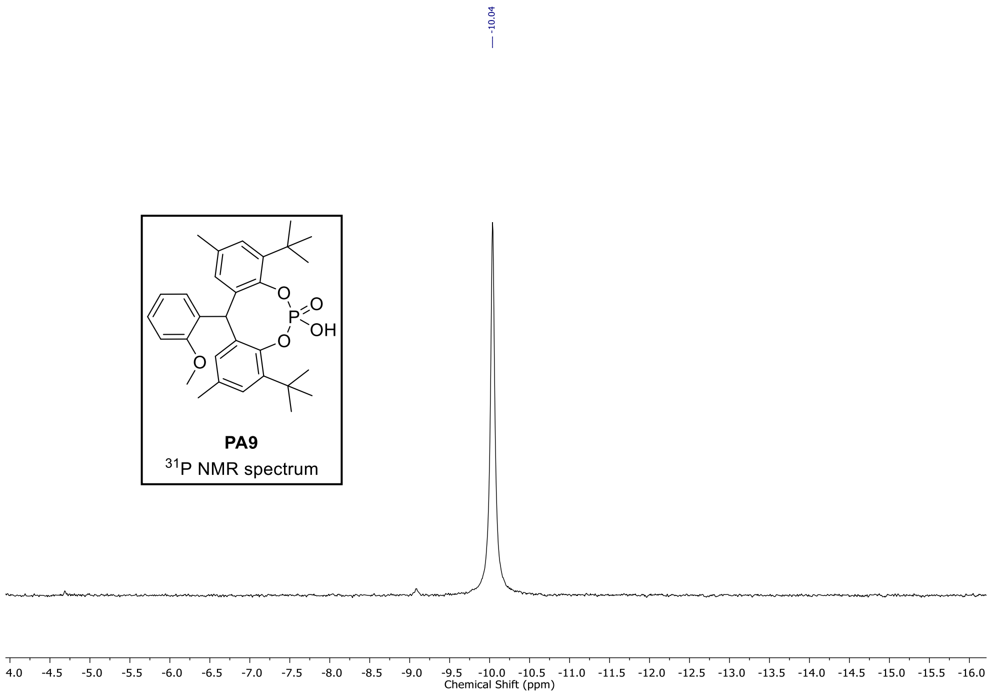

Figure S3. ^{31}P NMR spectrum of the phosphoric acid **PA9** at 27 ˚C.

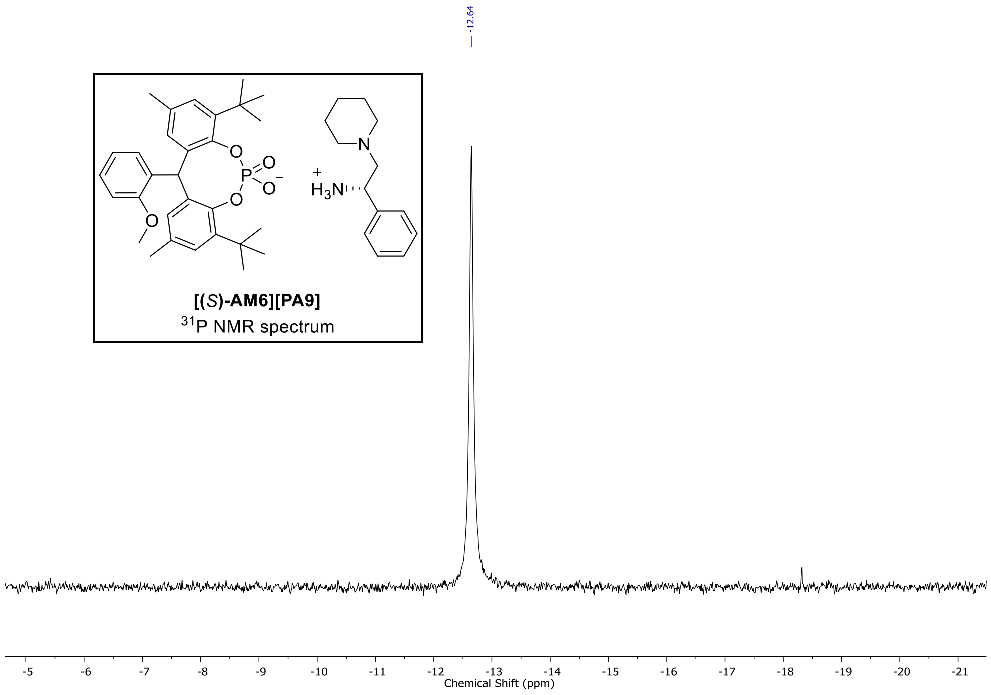

Figure S4. ^{31}P NMR spectrum of the salt [(*S*)-**AM6**][**PA9**] at 27 ˚C.

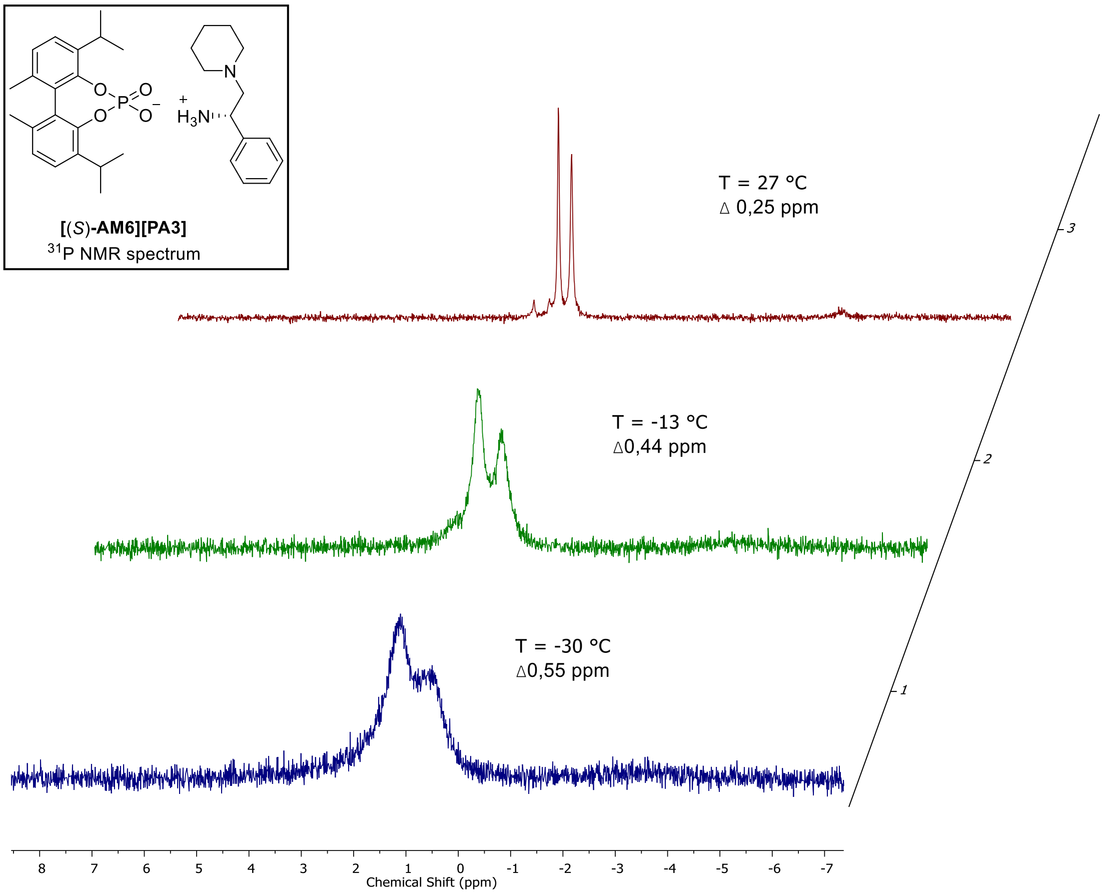

Figure S5. ^{31}P NMR spectrum of the salt [(*S*)-**AM6**][**PA3**] at different temperatures.

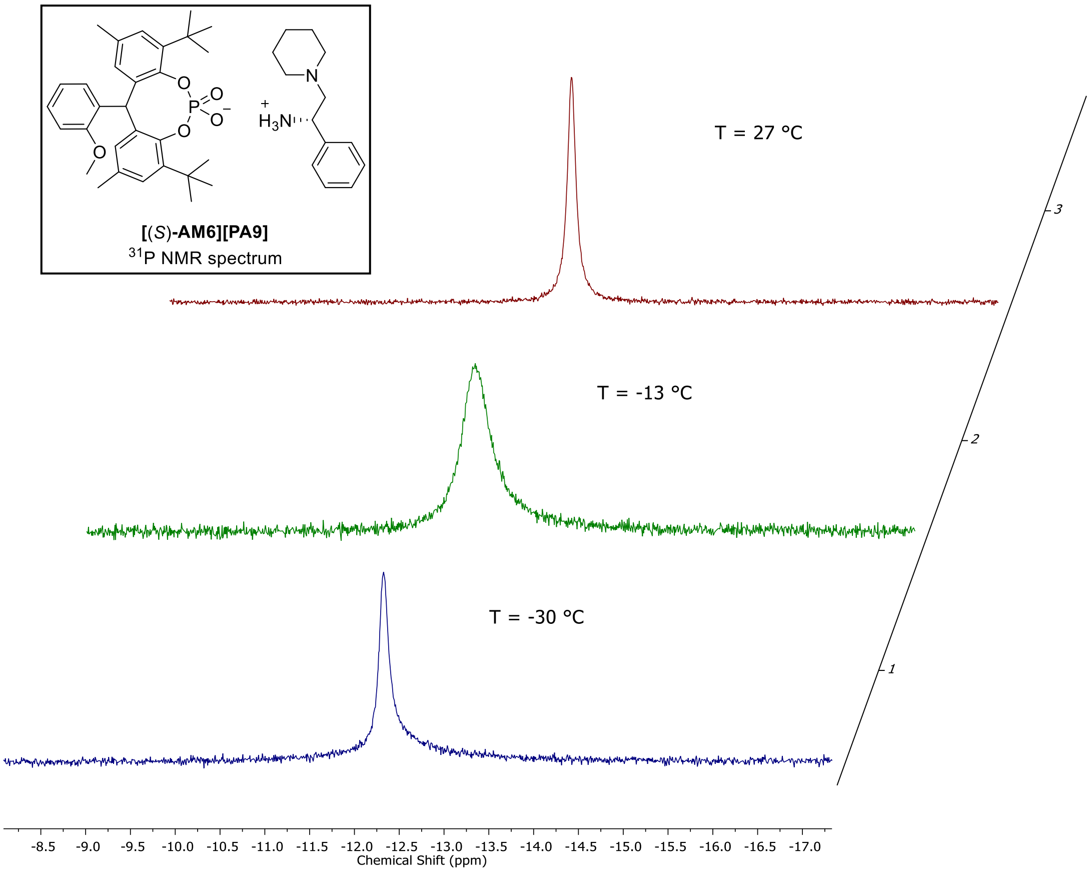

Figure S6. ^{31}P NMR spectrum of the salt [(*S*)-**AM6**][**PA9**] at different temperatures.

In order to further study this phenomenon, the salts of racemic **BINOL-P** and **PA9** with different amines (**AM2**-**AM6**) have been analyzed, respectively. While the former phosphoric acid readily formed stable diastereomers, no peak splitting was observed in any cases when using **PA9** (Figures S7-S8). Even the use of cinchonidine – which was already proven to be efficient for the resolution of chiral phosphoric acids[20] – resulted in no change in this phenomenon (Figures S9-S12).

All these NMR results suggests, that – with the exception of **PA3** – the phosphoric acids of our study can not make stable enantiomers at room temperature, and therefore their salts can act as single stereoisomeric catalyst. This might help avoiding the generation of match-mismatched scenarios and provide an easy access to both product antipodes retaining the enantioselectivity.

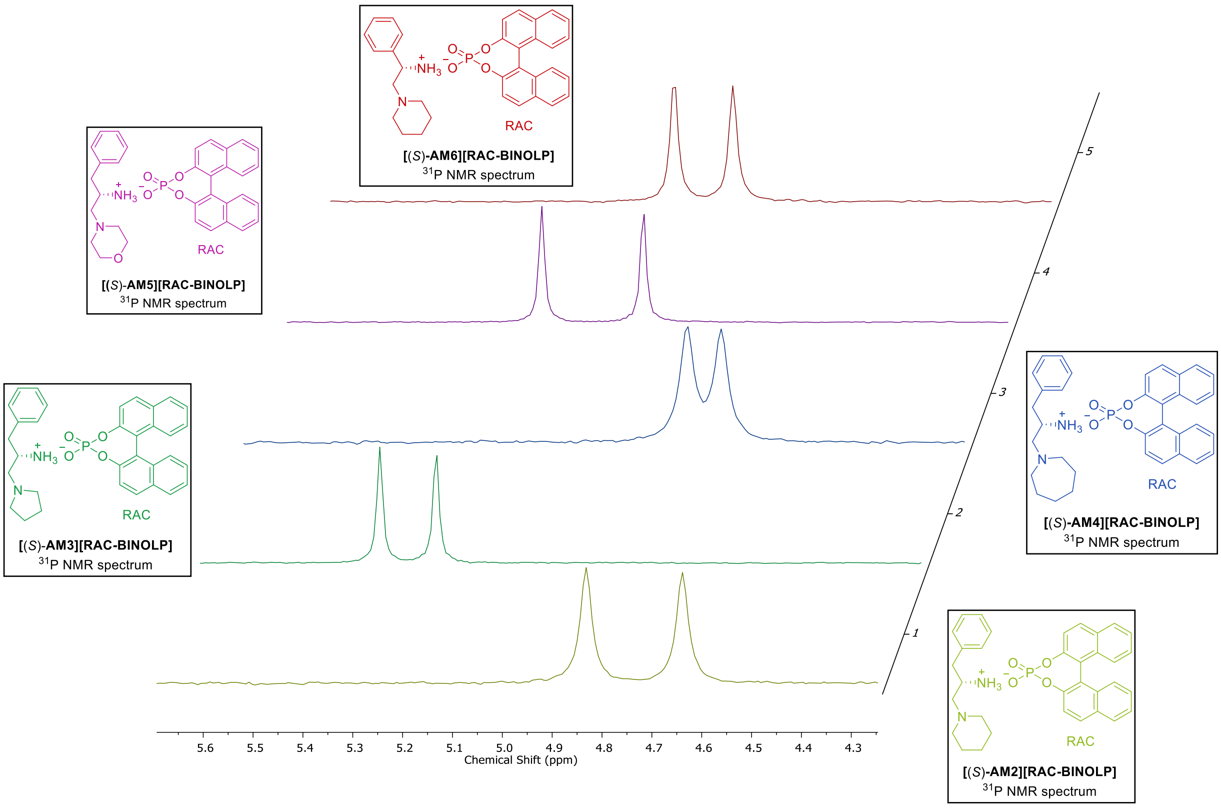

Figure S7. ^{31}P NMR spectra of different diastereomeric salts of chiral amines (*S*)-**AM2**-**AM6** and RAC-**BINOL-P** at room temperature.

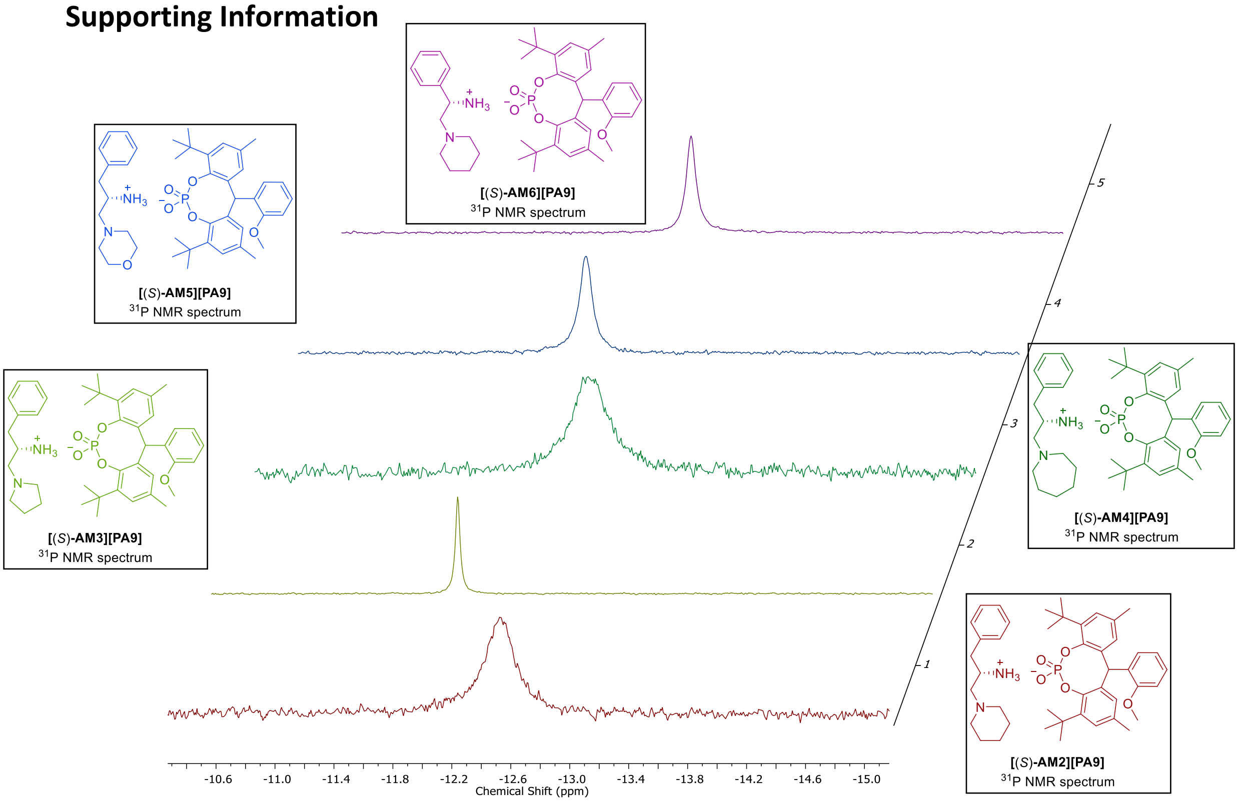

Figure S8. ^{31}P NMR spectra of different salts with chiral amines (*S*)-**AM2**-**AM6** and **PA9** at room temperature, showing no diastereomer formation.

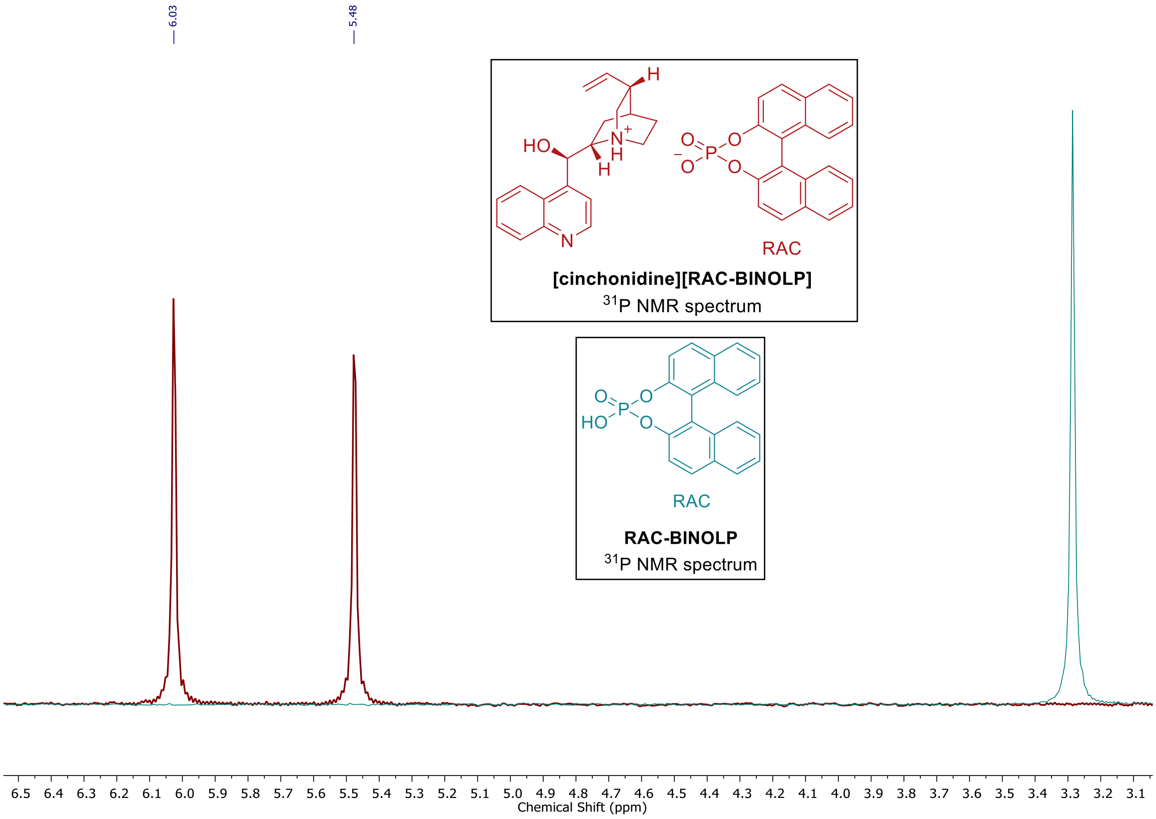

Figure S9. ^{31}P NMR spectra of the salt [cinchonidine][RAC-**BINOLP]** and the phosphoric acid RAC-**BINOLP** at room temperature.

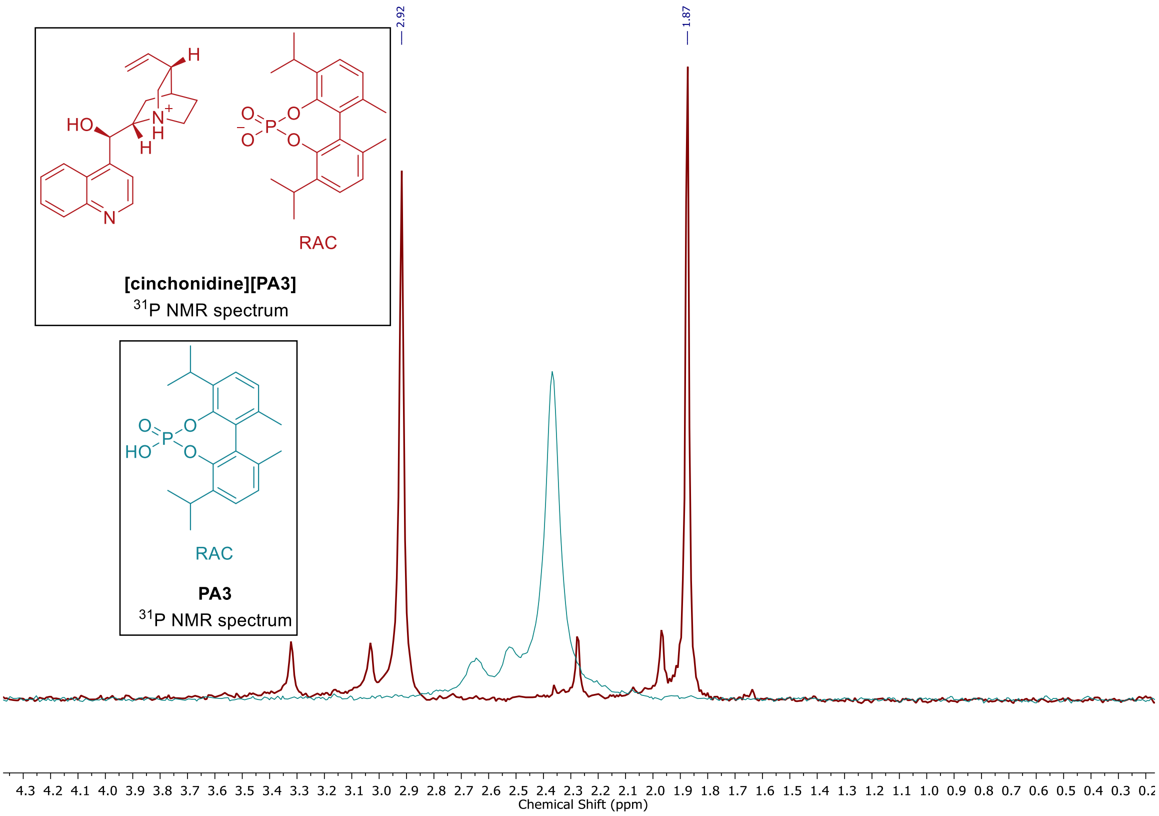

Figure S10. ^{31}P NMR spectra of the salt [cinchonidine]**[PA3]** and the phosphoric acid **PA3** at room temperature.

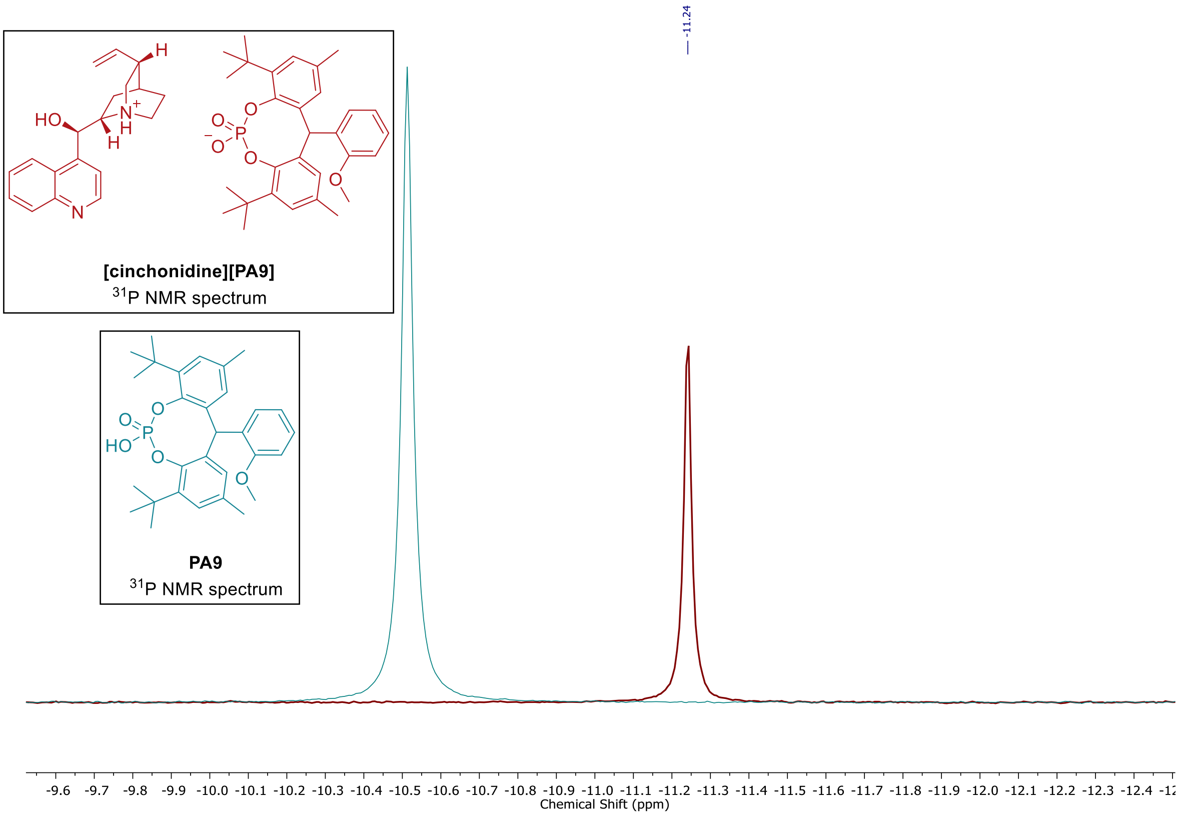

Figure S11. ^{31}P NMR spectra of the salt [cinchonidine]**[PA9]** and the phosphoric acid **PA9** at room temperature showing no diastereomer formation.

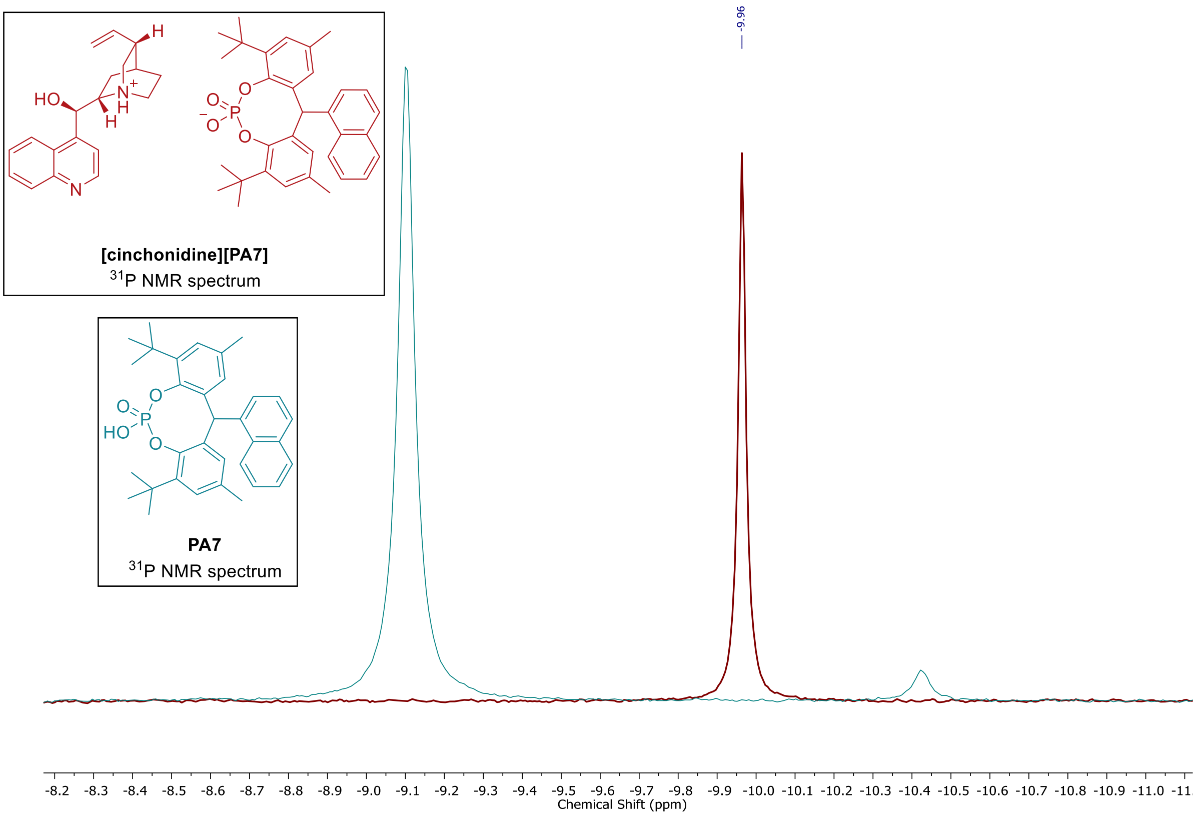

Figure S12. ^{31}P NMR spectrum of the salt [cinchonidine]**[PA7]** and the phosphoric acid **PA7** at room temperature showing no diastereomer formation.

24. XRD data for compounds 5c, 11a and PA9

Methodology

Single crystals of **5c**, **11a** and of the methanol solvate and dioxane disolvate hydrate of **PA9**, *viz*. **PA9-methanol** and **PA9-2dioxane-H_2O**, were pre-selected under a polarising microscope, embedded in perfluorinated polyether and mounted on MiTeGen MicroLoops®. X-ray diffraction data of all crystals were collected at 100 K on a Bruker Kappa APEX-II CCD diffractometer working with graphite-monochromated Mo–Kα radiation (λ = 0.71073 Å) and using narrow ω-scan frames. Corrections for absorption were applied with the multi-scan approach using SADABS.[32] The structures were solved with SHELXT and refined on F^2 with SHELXL.[33] All non-hydrogen atoms were refined with anisotropic displacement parameters. H atoms were placed in calculated positions and thereafter treated as riding, except the H atoms bonded to O atoms that were located from difference Fourier maps and refined freely.

Crystallographic data of (*S*,*R*,*S*)**5c**: $C_{13}H_{14}BrNO_2$; M_r = 296.16, colourless plate, 0.16 × 0.08 × 0.02 mm^3, trigonal, space group $P3_2$ (no. 145), a = 13.3743(8) Å, c = 6.0715(4) Å, V = 940.52(13) Å^3, Z = 3, d_x = 1.569 g/cm^3, T = 100 K. 13390 reflections collected (ϑ_{max} = 30.3°) and merged to 3721 independent data with $I>2\sigma(I)$ (R_{int} = 0.039); final R indices (all data) R_1 = 0.0360, wR_2 = 0.0547, GooF 1.018, 162 parameters, Flack parameter -0.018(5).

Crystallographic data of (-)-**11a**: $C_{11}H_{14}O_3$; M_r = 194.22, colourless fragment, 0.40 × 0.40 × 0.20 mm^3, monoclinic, space group $P2_1$ (no. 4), a = 7.622(2) Å, b = 7.839(2) Å, c = 8.088(2) Å, β = 97.683(6)°, V = 478.9(2) Å^3, Z = 2, d_x = 1.347 g/cm^3, T = 100 K. 5229 reflections collected (ϑ_{max} = 30.3°) and merged to 2778 independent data with $I>2\sigma(I)$ (R_{int} = 0.036); final R indices (all data) R_1 = 0.0571, wR_2 = 0.1540, GooF 1.087, 133 parameters; Flack parameter 0.2(10).

Crystallographic data of **PA9-methanol**: $C_{30}H_{36}O_5P,CH_5O$; M_r = 540.61, colourless block, 0.50 × 0.35 × 0.15 mm^3, triclinic, space group $P\bar{1}$ (no. 2), a = 8.5523(14) Å, b = 13.3715(18) Å, c = 13.6488(18) Å, α = 101.057(9)°, β = 94.356(9)°, γ = 100.883(10)°, V = 1494.1(4) Å^3, Z = 2, d_x = 1.202 g/cm^3, T = 100 K. 44750 reflections collected (ϑ_{max} = 33.1°) and merged to 11293 independent data with $I>2\sigma(I)$ (R_{int} = 0.027); final R indices (all data) R_1 = 0.0471, wR_2 = 0.1126, GooF 1.062, 361 parameters.

Crystallographic data of **PA9-2dioxane-H_2O**: $C_{30}H_{37}O_5P,2(C_4H_8O_2),H_2O$; M_r = 702.79, colourless fragment, 0.30 × 0.30 × 0.20 mm^3, triclinic, space group $P\bar{1}$ (no. 2), a = 10.3044(9) Å, b = 13.1948(11) Å, c = 15.2341(13) Å, α = 92.285(3)°, β = 103.859(3)°, γ = 112.366(2)°, V = 1839.8(3) Å^3, Z = 2, d_x = 1.269 g/cm^3, T = 100 K. 24690 reflections collected (ϑ_{max} = 25.0°) and merged to 6476 independent

data with $I>2\sigma(I)$ ($R_{int} = 0.079$); final R indices (all data) $R_1 = 0.0913$, $wR_2 = 0.1590$, GooF 1.025, 463 parameters.

Deposition numbers CCDC 2112845 for **5c**, CCDC 2112843 for **11**, CCDC 2112844 for **PA9-methanol** and CCDC 2112846 for **PA9-2dioxane-H_2O** contain the supplementary crystallographic data in CIF format. These data can be obtained free of charge from The Cambridge Crystallographic Data Centre via http://www.ccdc.cam.ac.uk/data_request/cif.

Crystal structures of 5c, 11a, PA9-methanol and PA9-2dioxane-H_2O

In all crystal structures, classical hydrogen bonds are the dominant intermolecular interactions. In **5c**, medium-strong N–H…$O_{carbonyl}$ and strong O–H…$O_{hydroxyl}$ interactions lead to the formation of supramolecular columns running parallel to [001]. Weak Br…Br interactions (3.5 Å) are present between the columns (Figure S13).

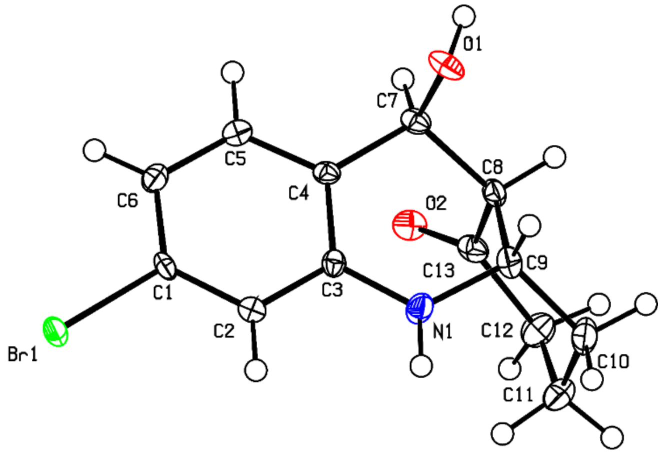

Figure S13. Molecular structure of (*S*,*R*,*S*)-**5c** with atoms shown as displacement ellipsoids at the 50% probability level. H atoms are represented by spheres of arbitrary radius.

In **11a**, adjacent molecules are linked through O–H…O$_{carbonyl}$ hydrogen bonds of medium strength into chains extending parallel to [101] (Figure S14).

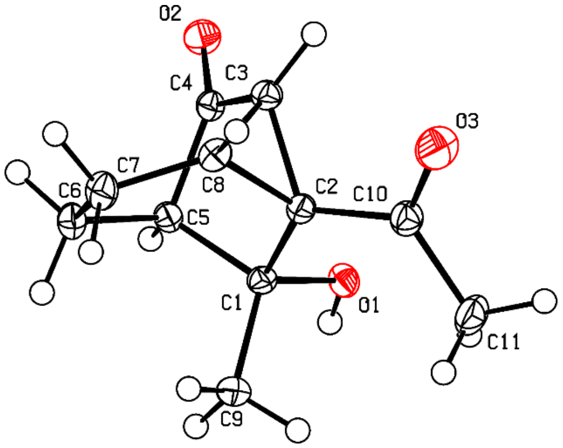

Figure S14. Molecular structure of (-)-**11** with atoms shown as displacement ellipsoids at the 50% probability level. H atoms are represented by spheres of arbitrary radius.

In **PA9-methanol**, a very strong symmetrical hydrogen bond is present between the phosphoric acid OH group and the O atom of the methanol solvate molecule. An additional very strong and likewise symmetrical O–H…O hydrogen bond between the methanol OH group and an O atom of the phosphoric acid moiety leads to a centrosymmetric ring motif with graph-set notation $R^4_4(12)$. In **PA9-2dioxane-H$_2$O**, again a very strong but asymmetrical hydrogen bond is present between the phosphoric acid moiety and the water molecule, and two more O–H…O hydrogen bonds of medium strengths between the water molecule and an dioxane solvent molecule and a phosphoric acid O atom, respectively, are present. The water molecules function as bridges between a **PA9** entity and a dioxane solvent molecule, leading to a hydrogen-bonded dimer (**PA9**-dioxane-H$_2$O)$_2$ with ring-set motif $R^4_4(12)$. The second dioxane solvent molecule is only weakly bound through C–H…O interactions (Figures S15 and S16).

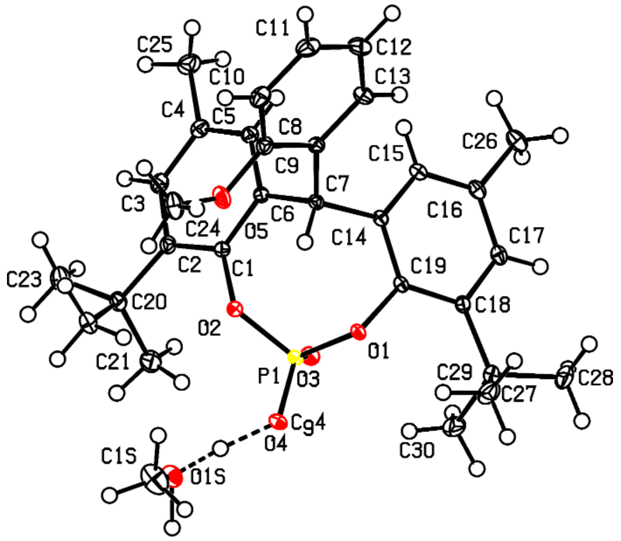

Figure S15. Molecular structure of (**PA9-methanol**) with atoms shown as displacement ellipsoids at the 50% probability level. H atoms are represented by spheres of arbitrary radius. The very strong hydrogen bonds between **PA9** and the methanol molecule is shown as dashed lines.

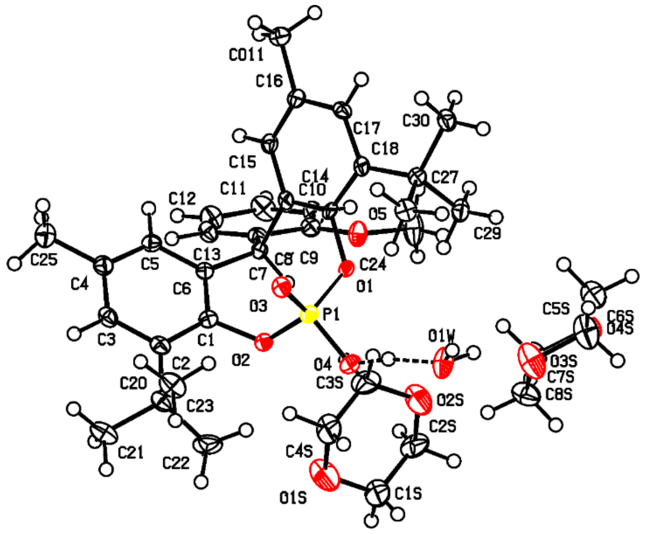

Figure S16. Molecular structure of (**PA9-2dioxane-H_2O**) with atoms shown as displacement ellipsoids at the 50% probability level. H atoms are represented by spheres of arbitrary radius. The very strong hydrogen bond between **PA9** and the water molecule is shown as dashed lines.

24. Bibliography

(1) Yu, G.; Wang, S.; Wang, K.; Hu, Y.; Hu, H. A Novel Approach to 1,2-Dihydro-2-Oxo-3-Pyridinecarboxylic Ester via Aromatization Induced by Deamidation. *Synthesis (Stuttg).* **2004**, No. 7, 1021–1028. https://doi.org/10.1055/s-2004-822325.

(2) Haraguchi, N.; Takenaka, N.; Najwa, A.; Takahara, Y.; Mun, M. K.; Itsuno, S. Synthesis of Main-Chain Ionic Polymers of Chiral Imidazolidinone Organocatalysts and Their Application to Asymmetric Diels-Alder Reactions. *Adv. Synth. Catal.* **2018**, *360* (1), 112–123. https://doi.org/10.1002/adsc.201701016.

(3) Martins, R. de S.; Pereira, M. P.; de Castro, P. P.; Bombonato, F. I. Design and Preparation of a Novel Prolinamide-Based Organocatalyst for the Solvent-Free Asymmetric Aldol Reaction. *Tetrahedron* **2020**, *76* (5), 130855. https://doi.org/10.1016/j.tet.2019.130855.

(4) Katritzky, A. R.; Fali, C. N.; Li, J.; Ager, D. J.; Prakash, I. Synthesis of 1-(T -Butoxycarbonyl)Benzotriazole and 1-(p -Methoxybenzyloxycarbonyl)Benzotriazole and Their Use in the Protection of Amino Acids. *Synth. Commun.* **1997**, *27* (9), 1623–1630. https://doi.org/10.1080/00397919708006101.

(5) Sestito, S.; Daniele, S.; Nesi, G.; Zappelli, E.; Di Maio, D.; Marinelli, L.; Digiacomo, M.; Lapucci, A.; Martini, C.; Novellino, E.; Rapposelli, S. Locking PDK1 in DFG-out Conformation through 2-Oxo-Indole Containing Molecules: Another Tools to Fight Glioblastoma. *Eur. J. Med. Chem.* **2016**, *118*, 47–63. https://doi.org/10.1016/j.ejmech.2016.04.003.

(6) Zhang, E.; Bai, P. Y.; Cui, D. Y.; Chu, W. C.; Hua, Y. G.; Liu, Q.; Yin, H. Y.; Zhang, Y. J.; Qin, S.; Liu, H. M. Synthesis and Bioactivities Study of New Antibacterial Peptide Mimics: The Dialkyl Cationic Amphiphiles. *Eur. J. Med. Chem.* **2018**, *143*, 1489–1509. https://doi.org/10.1016/j.ejmech.2017.10.044.

(7) Karmakar, A.; Basha, M.; Venkatesh Babu, G. T.; Botlagunta, M.; Malik, N. A.; Rampulla, R.; Mathur, A.; Gupta, A. K. Tertiary-Butoxycarbonyl (Boc) – A Strategic Group for N-Protection/Deprotection in the Synthesis of Various Natural/Unnatural N-Unprotected Aminoacid Cyanomethyl Esters. *Tetrahedron Lett.* **2018**, *59* (48), 4267–4271. https://doi.org/10.1016/j.tetlet.2018.10.041.

(8) Hatano, M.; Miyamoto, T.; Ishihara, K. Highly Active Chiral Phosphoramide−Zn(II) Complexes as Conjugate Acid−Base Catalysts for Enantioselective Organozinc Addition to Ketones. *Org. Lett.* **2007**, *9* (22), 4535–4538. https://doi.org/10.1021/ol702074a.

(9) Ando, A.; Shioiri, T. Enantioselective Synthesis of β-Hydroxy-α-Methyl Carbonyl Compounds by Aldol Reaction. *Tetrahedron* **1989**, *45* (16), 4969–4988. https://doi.org/10.1016/S0040-4020(01)81078-4.

(10) Khatik, G. L.; Kumar, V.; Nair, V. A. Reversal of Selectivity in Acetate Aldol Reactions of N -Acetyl-(S)-4-Isopropyl-1-[(R)-1-Phenylethyl]Imidazolidin-2-One. *Org. Lett.* **2012**, *14* (10), 2442–2445. https://doi.org/10.1021/ol300949s.

(11) Li, P.; Chai, Z.; Zhao, S.-L.; Yang, Y.-Q.; Wang, H.-F.; Zheng, C.-W.; Cai, Y.-P.; Zhao, G.; Zhu, S.-Z. Highly Enantio- and Diastereoselective Synthesis of α-Trifluoromethyldihydropyrans Using a Novel Bifunctional Piperazine-Thiourea Catalyst. *Chem. Commun.* **2009**, No. 47, 7369. https://doi.org/10.1039/b915210e.

(12) Li, J.; Luo, S.; Cheng, J. Chiral Primary−Tertiary Diamine Catalysts Derived From Natural Amino Acids for Syn -Aldol Reactions of Hydroxy Ketones. *J. Org. Chem.* **2009**, *74* (4), 1747–1750. https://doi.org/10.1021/jo802557p.

(13) Zhou, Y.; Liu, Q.; Gong, Y. Camphor-Derived C1-Symmetric Chiral Diamine Organocatalysts for Asymmetric Michael Addition of Nitroalkanes to Enones. *Org. Biomol. Chem.* **2012**, *10* (37), 7618. https://doi.org/10.1039/c2ob25922b.

(14) Rossiter, B. E.; Eguchi, M.; Miao, G.; Swingle, N. M.; Hernández, A. E.; Vickers, D.; Fluckiger, E.; Greg Patterson, R.; Vásavi Reddy, K. Enantioselective Conjugate Addition to Cyclic Enones with Scalemic Lithium Organo(Amido)Cuprates, Part IV. Relationship between Ligand Structure and Enantioselectivity. *Tetrahedron* **1993**,

49 (5), 965–986. https://doi.org/10.1016/S0040-4020(01)86278-5.

(15) Kumar, A.; Singh, S.; Kumar, V.; Singh Chimni, S. Asymmetric Syn-Selective Direct Aldol Reaction of Protected Hydroxyacetone Catalyzed by Primary Amino Acid Derived Bifunctional Organocatalyst in the Presence of Water. *Org. Biomol. Chem.* **2011**, *9* (8), 2731. https://doi.org/10.1039/c0ob00898b.

(16) Davis, T. J.; Balsells, J.; Carroll, P. J.; Walsh, P. J. Optimization of Asymmetric Catalysts Using Achiral Ligands: Metal Geometry-Induced Ligand Asymmetry. *Org. Lett.* **2001**, *3* (14), 2161–2164. https://doi.org/10.1021/ol016003d.

(17) Fareghi-Alamdari, R.; Golestanzadeh, M.; Agend, F.; Zekri, N. Regiospecific, One-Pot, and Pseudo-Five-Component Synthesis of 6,6′-(Arylmethylene)Bis(2-(Tert -Butyl)4-Methylphenol) Antioxidants Using Highly Sulfonated Multi-Walled Carbon Nanotubes under Solvent-Free Conditions. *Can. J. Chem.* **2013**, *91* (10), 982–991. https://doi.org/10.1139/cjc-2013-0160.

(18) Hsueh, M.-L.; Huang, B.-H.; Lin, C.-C. Reactions of 2,2′-(2-Methoxybenzylidene)Bis(4-Methyl-6- Tert -Butylphenol) with Trimethylaluminum: Novel Efficient Catalysts for "Living" and "Immortal" Polymerization of ε-Caprolactone. *Macromolecules* **2002**, *35* (15), 5763–5768. https://doi.org/10.1021/ma020574j.

(19) Grant-Overton, S.; Buss, J. A.; Smith, E. H.; Gutierrez, E. G.; Moorhead, E. J.; Lin, V. S.; Wenzel, A. G. Efficient Microwave Method for the Oxidative Coupling of Phenols. *Synth. Commun.* **2015**, *45* (3), 331–337. https://doi.org/10.1080/00397911.2014.956370.

(20) Gutierrez, E. G.; Moorhead, E. J.; Smith, E. H.; Lin, V.; Ackerman, L. K. G.; Knezevic, C. E.; Sun, V.; Grant, S.; Wenzel, A. G. Electron-Withdrawing, Biphenyl-2,2′-diol-Based Compounds for Asymmetric Catalysis. *Eur. J. Org. Chem.* **2010**, *1* (16), 3027–3031. https://doi.org/10.1002/ejoc.201000070.

(21) Kumara Swamy, K. C.; Kumaraswamy, S.; Kommana, P. Very Strong C−H···O, N−H···O, and O−H···O Hydrogen Bonds Involving a Cyclic Phosphate. *J. Am. Chem. Soc.* **2001**, *123* (50), 12642–12649. https://doi.org/10.1021/ja010713x.

(22) Martin, N. J. A.; List, B. Highly Enantioselective Transfer Hydrogenation of α,β- Unsaturated Ketones. *J. Am. Chem. Soc.* **2006**, *128* (41), 13368–13369. https://doi.org/10.1021/ja065708d.

(23) Shintani, R.; Takeda, M.; Nishimura, T.; Hayashi, T. Chiral Tetrafluorobenzobarrelenes as Effective Ligands for Rhodium-Catalyzed Asymmetric 1,4-Addition of Arylboroxines to β,β-Disubstituted α,β-Unsaturated Ketones. *Angew. Chem. Int. Ed.* **2010**, *49* (23), 3969–3971. https://doi.org/10.1002/anie.201000467.

(24) Yasui, N.; Mayne, C. G.; Katzenellenbogen, J. A. Preparation of o -Fluorophenols from Nonaromatic Precursors: Mechanistic Considerations for Adaptation to Fluorine-18 Radiolabeling. *Org. Lett.* **2015**, *17* (22), 5540–5543. https://doi.org/10.1021/acs.orglett.5b02640.

(25) Palais, L.; Alexakis, A. Copper-Catalyzed Asymmetric Conjugate Addition with Chiral SimplePhos Ligands. *Chem. - A Eur. J.* **2009**, *15* (40), 10473–10485. https://doi.org/10.1002/chem.200901577.

(26) LARCHEVÊQUE, M.; VALETTE, G.; CUVIGNY, T. Hyperbasic Media; Synthesis of 1-Alkyl-3-Oxocyclohexenes from 1,5-Diketones. *Synthesis (Stuttg).* **1977**, *1977* (06), 424–427. https://doi.org/10.1055/s-1977-24428.

(27) Wang, X.; Reisinger, C. M.; List, B. Catalytic Asymmetric Epoxidation of Cyclic Enones. *J. Am. Chem. Soc.* **2008**, *130* (19), 6070–6071. https://doi.org/10.1021/ja801181u.

(28) Saini, G.; Mondal, A.; Kapur, M. Palladium-Mediated Remote Functionalization in γ- and ε-Arylations and Alkenylations of Unblocked Cyclic Enones. *Org. Lett.* **2019**, *21* (22), 9071–9075. https://doi.org/10.1021/acs.orglett.9b03462.

(29) Danishefsky, S.; Zimmer, A. The Direction of Base-Catalyzed Aldol Cyclization of 1,5 Diketones. *J. Org. Chem.* **1976**, *41* (26), 4059–4064. https://doi.org/10.1021/jo00888a002.

(30) Cussó, O.; Cianfanelli, M.; Ribas, X.; Klein Gebbink, R. J. M.; Costas, M. Iron Catalyzed Highly Enantioselective Epoxidation of Cyclic Aliphatic Enones with Aqueous H 2 O 2. *J. Am. Chem. Soc.* **2016**, *138* (8), 2732–2738. https://doi.org/10.1021/jacs.5b12681.

(31) De Vincentiis, F.; Bencivenni, G.; Pesciaioli, F.; Mazzanti, A.; Bartoli, G.; Galzerano, P.; Melchiorre, P. Asymmetric Catalytic Aziridination of Cyclic Enones. *Chem. - Asian J.* **2010**, *5* (7), 1652–1656. https://doi.org/10.1002/asia.201000040.

(32) Krause, L.; Herbst-Irmer, R.; Sheldrick, G. M.; Stalke, D. Comparison of Silver and Molybdenum Microfocus X-Ray Sources for Single-Crystal Structure Determination. *J. Appl. Crystallogr.* **2015**, *48* (1), 3–10. https://doi.org/10.1107/S1600576714022985.

(33) Sheldrick, G. M. SHELXT – Integrated Space-Group and Crystal-Structure Determination. *Acta Crystallogr. Sect. A Found. Adv.* **2015**, *71* (1), 3–8. https://doi.org/10.1107/S2053273314026370.
